# Insights into the multi-chromosomal mitochondrial genome structure of the xero-halophytic plant *Haloxylon Ammodendron* (C.A.Mey.) Bunge ex Fenzl

**DOI:** 10.1186/s12864-024-10026-6

**Published:** 2024-01-29

**Authors:** Lulu Yang, Jia Liu, Wenjun Guo, Zehan Zheng, Yafei Xu, Houjun Xia, Tian Xiao

**Affiliations:** 1 Department of Cell Biology and Genetics, School of Basic Medical Sciences, Shenzhen University Medical School, Shenzhen University, Shenzhen, Guangdong 518055 China; 2 Biomedical Research Center, Tongji University Suzhou Institute, Suzhou, Jiangsu 215101 China; 3 State Key Laboratory of Oncology in South China, Guangdong Key Laboratory of Nasopharyngeal Carcinoma Diagnosis and Therapy, Sun Yat-sen University Cancer Center, Guangzhou, Guangdong 510060 China; 4 Center for Cancer Immunology, Faculty of Pharmaceutical Sciences, Shenzhen Institute of Advanced Technology, Chinese Academy of Sciences, Guangdong, 518055 China

**Keywords:** *Haloxylon*, Xerophytic plant, Mitochondrial genome, Multi-chromosome, DNA migration

## Abstract

**Background:**

*Haloxylon ammodendron* holds significance as an ecological plant, showcasing remarkable adaptability to desert conditions, halophytic environments, and sand fixation. With its potential for carbon sequestration, it emerges as a promising candidate for environmental sustainability. Furthermore, it serves as a valuable C4 plant model, offering insights into the genetic foundations of extreme drought tolerance. Despite the availability of plastid and nuclear genomes, the absence of a mitochondrial genome (mitogenome or mtDNA) hinders a comprehensive understanding of its its mtDNA structure, organization, and phylogenetic implications.

**Results:**

In the present study, the mitochondrial genome of *H. ammodendron* was assembled and annotated, resulting in a multi-chromosomal configuration with two circular chromosomes. The mtDNA measured 210,149 bp in length and contained 31 protein-coding genes, 18 tRNA and three rRNA. Our analysis identified a total of 66 simple sequence repeats along with 27 tandem repeats, 312 forward repeats, and 303 palindromic repeats were found. Notably, 17 sequence fragments displayed homology between the mtDNA and chloroplast genome (cpDNA), spanning 5233 bp, accounting for 2.49% of the total mitogenome size. Additionally, we predicted 337 RNA editing sites, all of the C-to-U conversion type. Phylogenetic inference confidently placed *H. ammodendron* in the Amaranthacea family and its close relative, *Suaeda glacum*.

**Conclusions:**

*H. ammodendron* mtDNA showed a multi-chromosomal structure with two fully circularized molecules. This newly characterized mtDNA represents a valuable resource for gaining insights into the basis of mtDNA structure variation within Caryophyllales and the evolution of land plants, contributing to their identification, and classification.

**Supplementary Information:**

The online version contains supplementary material available at 10.1186/s12864-024-10026-6.

## Background

*H. ammodendron* is a small C4 perennial tree belonging to the Amaranthaceae family, formerly known as Chenopodiaceae [1]. The genus *Haloxylon* encompasses approximately 11 species, distributed from Iran to Mongolia and Northwestern China [2]. Referred to as the King of psammophytic woody plants, *H. ammodendron* demonstrates remarkable adaptability to severe environmental conditions, including desert conditions and sandstorms [3]. Widely found in the deserts of northwest China, it plays a crucial role in environmental protection through wind control, carbon sequestration, and sand fixation [4]. However, due to increasing overexploitation and the dramatic climate change occurring in central Asia, *Haloxylon* plants face a threat [5].

The declining numbers of these plants and the subsequent ecological impacts have sparked significant interest in genetic research on *Haloxylon* resources. Long et al. [6] provided the first transcriptome resource of *H. ammodendron* to investigate genes that confer drought stress resistance to the plant. For a thorough understanding of the resilience of *H. ammodendron* to drought stress, Gao et al. [7] conducted a complete transcriptome analysis under osmotic conditions, paired with evaluations of physiological factors such as proline, betaine, soluble sugar levels, and peroxidase activity. Using cpDNA genes (*trnS-trnG* and *trnV*) and ITS (Internal transcribed spacer) (ITS1-ITS4) sequences, Chen at al [8]. assessed 420 individuals from 36 populations of *Haloxylon* for their genetic diversity and historical demography. Suo et al. [9] utilized SSR marker-derived DNA markers to distinguish *H. ammodendron* and *Haloxylon persicum* species. Later, the cpDNA of *H. ammodendron* and *H. persicum* were assembled using short-read sequencing data. Recently, a high-quality chromosome-level assembly of *H. ammodendron* was released [10], paving the way for an in-depth investigation of genes of interest concerning its high tolerance to drought and salinity. Despite the availability of these genomic resources, the mitochondrial genome (mitogenome or mtDNA) of *H. ammodendron* is lacking, hampering mtDNA-based structural and functional evolutionary studies.

The mitogenome, often referred to as the cell’s powerhouse, plays a crucial role in cellular energy production [11]. Housed in the mitochondria, this distinct genome is responsible for producing essential proteins for oxidative phosphorylation, leading to the creation of adenosine triphosphate (ATP)– the primary energy currency of the cell [12, 13]. High variations in mtDNA size coupled with structural complexity, are the major factors slowing the pace of fully and accurately assembled mitogenome projects. For example, mitogenome sizes vary over 40-fold in the single genus Silene, from 253 kbp in *Silene latifolia* to more than 11 Mbp in *Silene conica* [14]. Variation in repetitive sequences, the incorporation of foreign sequences, and gain or loss of non-coding regions are among the key factors contributing to genome size variability [15, 16].

Furthermore, while a single circular mitogenome is common in angiosperms, various configurations may occur, including linear, fragmented, loop-like, branched, and multi-chromosomal structures [11]. The dynamic nature of mtDNA presents challenges in fully assembling the genomes of certain species compared to cpDNA assembly.

However, advancements in long-read sequencing technologies have made the assembly of plant mitochondrial genomes more achievable, facilitating comparative studies of plant mitogenome structure and evolutionary implications [17, 18]. In the present study, short and long-read sequencing data were used to generate the first mtDNA of *H. ammodendron*. The characterization of repeats content, sequence collinearity, DNA migration and RNA editing sites were explored.

## Results

### Genome assembly and annotation

**Fig. 1:**
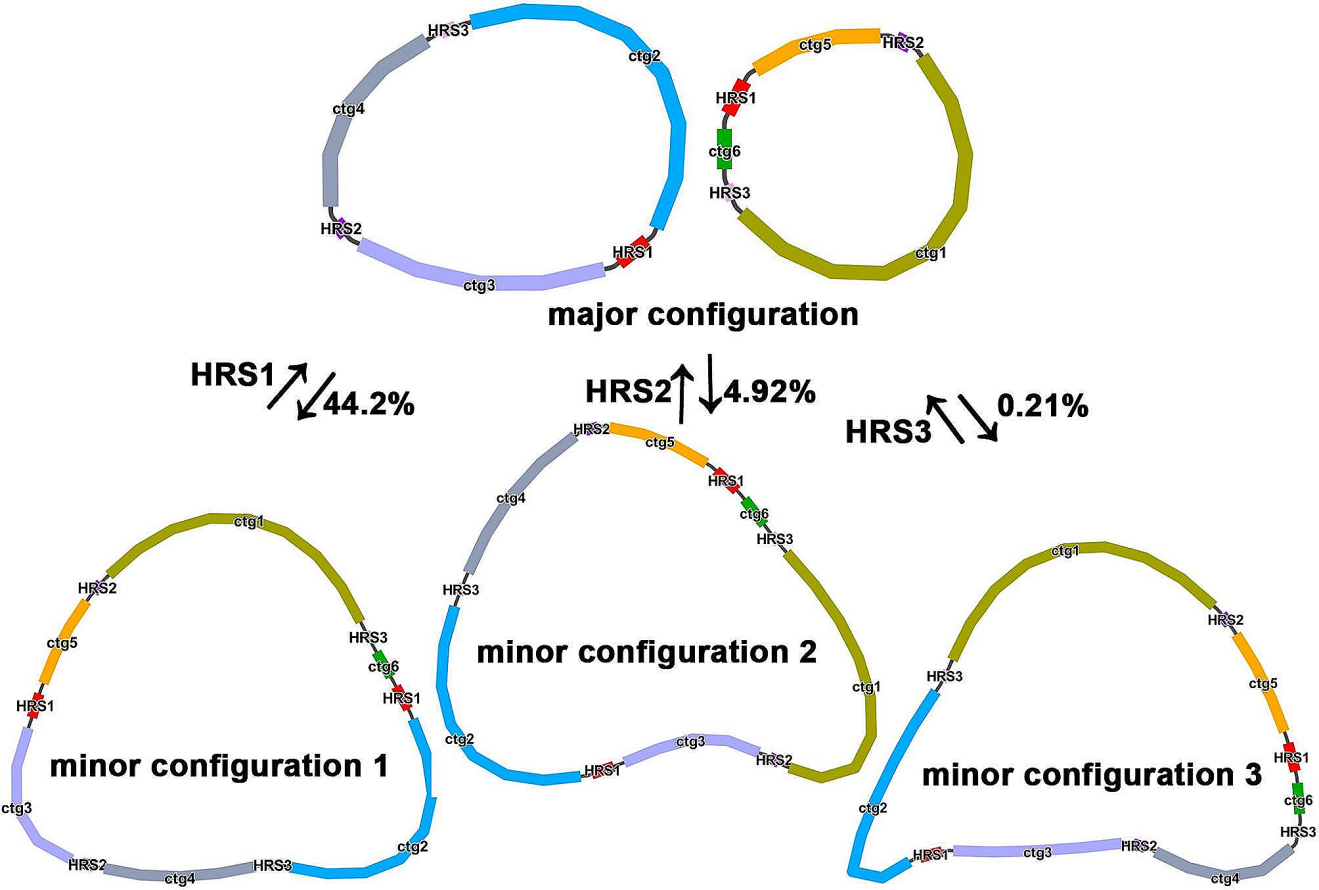
Diagram of mitogenome recombination. The major configuration of *Haloxylon ammodendron* mitogenome is two independent circular mtDNA, with three pairs of repeats that can mediate gene recombination: HRS1, HRS2 and HRS3. According to the supported long-reads, the frequencies of mitogenome recombination mediated by HRS1, HRS2 and HRS3 were 44.2%, 4.92%, and 0.21%, respectively

In this study, we utilized Illumina short-reads and PacBio HiFi long-reads to assemble the mtDNA of *H. ammodendron*. Importantly, the data was not generated within this study; instead, we sourced the Illumina short-reads and PacBio long-reads from a previously conducted study on the nuclear genome of the species. Specifically, we accessed 49.4 Gbp of clean short reads (Table S1) from the NCBI SRA database under accession number SRR17127859. The assembly was facilitated using the GetOrganelle software, version 1.7.5 [19]. In parallel, the PacBio dataset comprised 22.6 Gbp of long-reads, also retrieved from NCBI SRA under the accession SRR17129371. When visualizing the short-read assembly graph using Bandage version 0.8.1 [20], an intricate and circular multi-branch mtDNA structure was assembled encompassing nodes or contigs (Fig. 1). These nodes were delineated by overlapping regions depicted by black lines on the assembly graph. Overlapping regions were resolved by aligning associated branch nodes with the PacBio HiFi dataset. Due to the presence of repeats, recombinant sequences could occur, leading to different configurations. Therefore, four potential genomic paths were inferred. Paths 1–1 and 1–2 were considered major configurations, while paths 2 − 1 and 2–2 represented minor configurations (Fig. 1, Table S2, Table S3). The major configuration was obtained by extending 2000 bp on both ends based on the repetitive sequence. As for the minor configuration, the 2000 bp extension was not supported by the long-reads data (Fig. 1, Table S2, Table S3). Consequently, two fully circular sequences representing the mtDNA of *H. ammodendron*, were obtained (Fig. 1). The two circularized contigs, namely Chromosome 1 and Chromosome 2, exhibited similar GC content (44%) spanning 121,403 bp and 88,746 bp respectively (Table 1).

**Table 1:** Basic statistics of *H. ammodendron* mitochondrial genome

Contigs	Type	Length	GC content
Chromosome 1–2	Branched	210,149 bp	44.42%
Chromosome 1	circular	121,403 bp	44.58%
Chromosome 2	circular	88,746 bp	44.21%

The annotation of the *H. ammodendron* mtDNA is summarized in Table 2. A total of 31 protein-coding genes, 18 tRNA and three rRNA were predicted. Among the protein-coding genes, 24 core genes typically found in angiosperms [21], and seven variably present genes were identified. The core genes include five ATP synthase genes (*atp1*, *atp4*, *atp6*, *atp8*, and *atp9*), nine NADH dehydrogenase genes (*nad1*, *nad2*, *nad3*, *nad4*, *nad4L*, *nad5*, *nad6*, *nad7*, and *nad9*), four cytochrome c biogenesis genes (*ccmB*, *ccmC*, *ccmFC*, and *ccmFN*), three cytochrome c oxidase genes (*cox1*, *cox2*, and *cox3*), one membrane transport protein gene (*mttB*), one mature enzyme gene (*matR*), and one ubiquinol-cytochrome c reductase gene (*cob*). The non-core genes include one ribosomal large subunit gene (*rpl5*), five ribosomal small subunit genes (*rps3*, *rps4*, *rps7*, *rps12*, *rps13*), and one succinate dehydrogenase gene (*sdh4*). Regarding tRNA, three were in duplicate copy (*trnC-GCA*, *trnG-GCC*, *trnP-UGG*), while one was in triplicate copy (*trnfM-CAU*). Among the three rRNA, *rrn26* was present in duplicate copy.

**Table 2:** Gene content information in *H. ammodendron* mitochondrial genome

Group of genes		Name of genes
Core genes	ATP synthase	*atp1*, *atp4*, *atp6*, *atp8*, *atp9*
	NADH dehydrogenase	*nad1, nad2, nad3, nad4(×2), nad4L, nad5, nad6, nad7, nad9*
	Cytochrome c biogenesis	*ccmB*, *ccmC*, *ccmFC*, *ccmFN*
	Ubiquinol cytochrome c reductase	*Cob*
	Cytochrome c oxidase	*cox1*, *cox2*, *cox3*
	Maturases	*matR*
	Transport membrane protein	*mttB*
Variable genes	Large subunit of ribosome	*rpl5*
	Small subunit of ribosome	*rps3*, *rps4*, *rps7*, *rps12*, *rps13*
	Succinate dehydrogenase	*sdh4*
rRNA genes	Ribosome RNA	*rrn5*, *rrn18*, *rrn26* (×2)
tRNA genes	Transfer RNA	*trnC-GCA(×2), trnD-GUC, trnE-UUC, trnFGAA, trnfM-CAU (×3), trnG-GCC (×2), trnHGUG, trnI-CAU, trnK-UUU, trnM-CAU, trnNGUU, trnP-UGG (×2), trnQ-UUG, trnS-GCU, trnS-GGA, trnS-UGA, trnW-CCA, trnY-GUA*

### Gene transfer

**Fig. 2:**
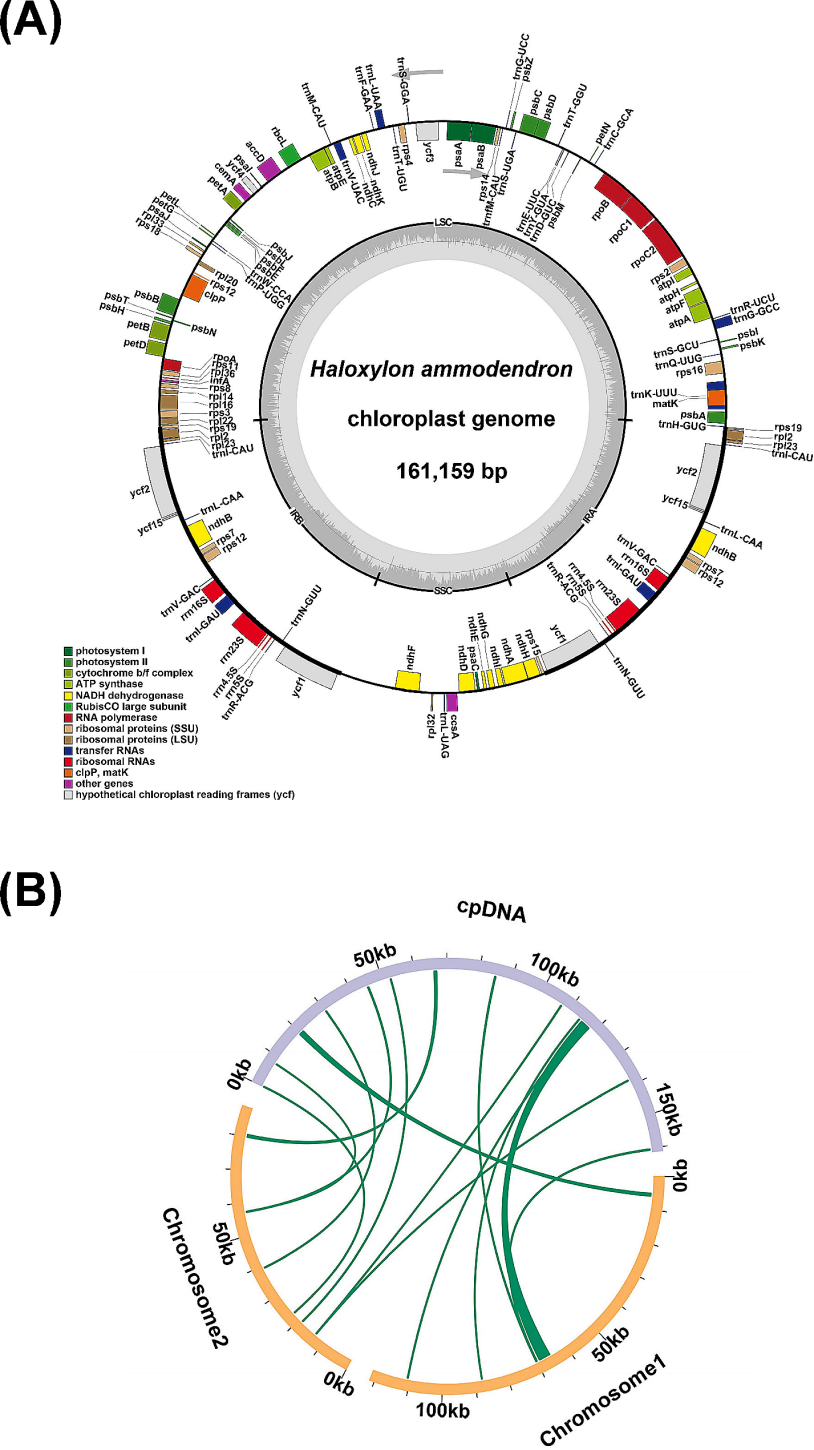
*H. ammodendron* plastome overview and sequence similarity. **(A)** Depiction of the *H. ammodendron* plastome, providing a visual representation of its organization and key features. **(B)** Illustration highlighting sequence similarity between the mitochondrial and chloroplast organelles. Yellow arcs denote regions of the mitochondrial genome, while the purple arc signifies the chloroplast genome. The interconnecting green lines emphasize homologous segments shared between the two genomes

Putative gene transfer between organelles was unveiled through alignment of the newly assembled cpDNA (Fig. 2A) with the mtDNA of *H. ammodendron*. Homologous sequence fragments between the organelles are illustrated in Fig. 2B. According to the sequence similarity analysis, 17 DNA fragments were identified as homologous between the mtDNA and cpDNA, with a total length of 5233 bp, accounting for 2.49% of the mtDNA (Table S4). Among them, the longest fragment measured 1158 bp in length. By annotating these homologous sequences, eight complete genes were found on the 17 homologous fragments, including one protein-coding gene (*petG*) and seven tRNA genes (*trnD-GUC*, *trnH-GUG*, *trnM-CAU*, *trnN-GUU*, *trnP-UGG*, *trnS-GCU*, *trnW-CCA*). Additionally, we investigated the mtDNA trace in the nuclear genome (Table S5). The longest alignment, with a length of 15 Kbp, was identified on chromosome 1 (Table S5).

### Repeat sequences analysis

**Fig. 3:**
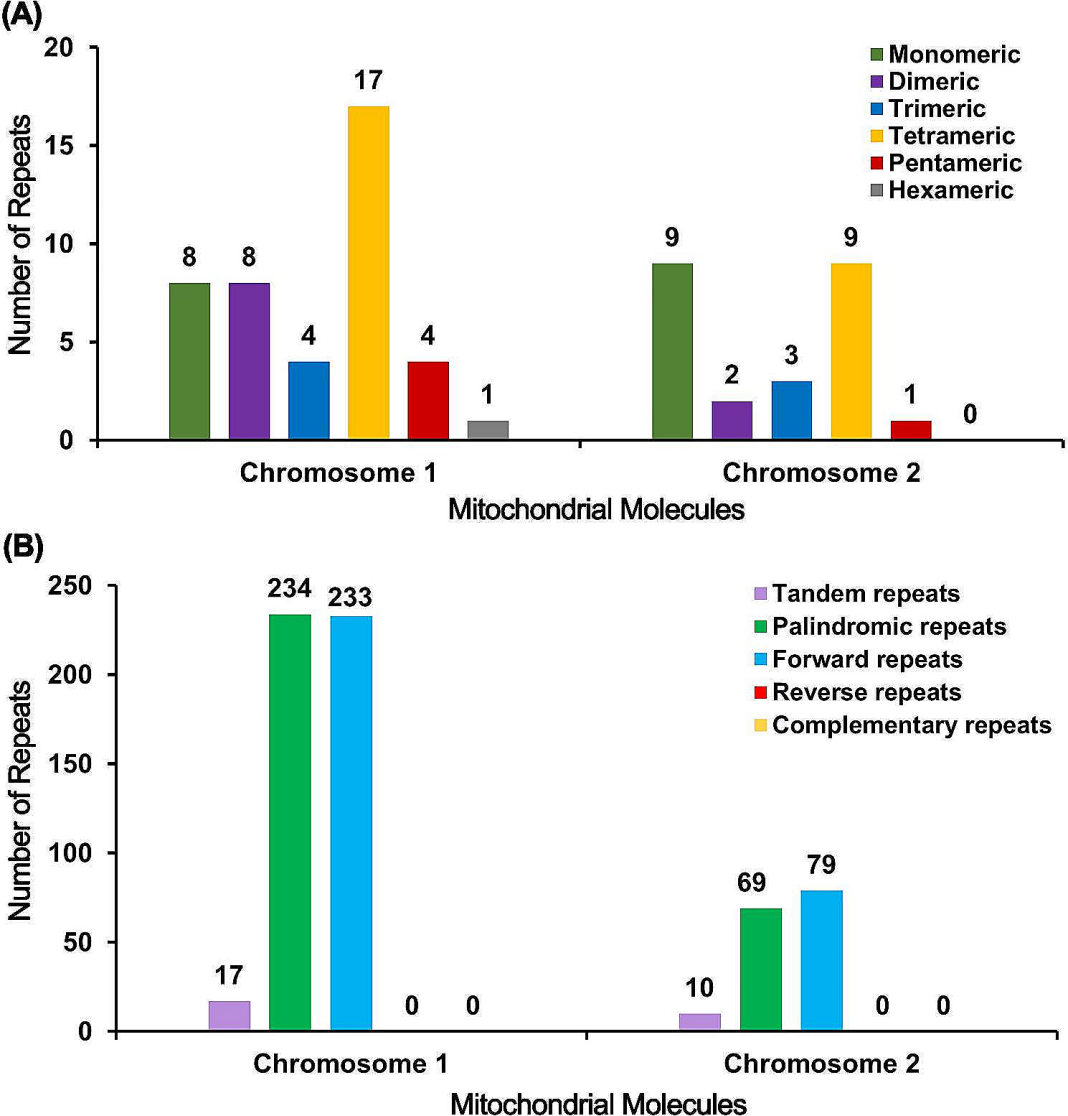
Repeat patterns in the *H. ammodendron* mitochondrial genome. (**A**) Chart showing the count of Simple Sequence Repeats (SSRs) distributed in chromosome 1 and chromosome 2. (**B**) Visualization of the number of tandem and dispersed repeats found in both chromosomes, offering insight into the genome’s complexity and evolutionary history

Microsatellites were screened for each set of chromosomes in the *H. ammondendron* mtDNA (Fig. 3A). For chromosome 1, a total of 42 simple sequence repeats (SSRs) were identified, comprising eight monomeric SSRs, eight dimeric SSRs, four trimeric SSRs, 17 tetrameric SSRs, four tetrameric SSRs, and one hexameric SSRs (Fig. 3A). Among the SSRs, the repeat motifs (A)_10_, (GAAA)_3_ and (CT)_5_ were found to be prevalent (Table S6). Seventeen tandem repeat sequences were identified, exhibiting a matching identity greater than 70% and lengths ranging from 14 to 31 bp (Fig. 3B, Table S7). Dispersed repeat sequences in chromosome 1 were also examined, detecting 467 repeat sequence pairs with a length equal to or greater than 30 bp. Among these, 234 pairs were palindromic repeats, 233 were forward repeats, and one was found to be a reverse repeat type. The longest observed palindromic repeat sequence spanned 123 bp, while the longest forward repeat sequence was 175 bp in length (Table S8).

In chromosome 2, 24 SSRs were detected (Fig. 3A). Among these SSRs, nine were monomeric, two were dimeric, three were trimeric, nine were tetrameric, and one was the pentameric type. No hexameric SSRs were found in this chromosome. The most prominent SSR motif was thymine (T)_10,_ among others (Table S7). A total of 10 tandem repeat sequences with a matching identity greater than 78% and lengths ranging from 18 to 42 bp were identified (Fig. 3B, Table S8). Dispersed repeat sequences in chromosome 2 were also examined, detecting 148 pairs of repeat sequences with a length equal to or greater than 30 bp. Among these, 69 pairs were palindromic repeats, and 79 were forward repeats. No reverse or complement repeats were detected in both chromosomes. The longest observed palindromic repeat sequence spanned 56 bp, while the longest forward repeat sequence was 114 bp in length (Table S8).

### RNA editing

**Fig. 4:**
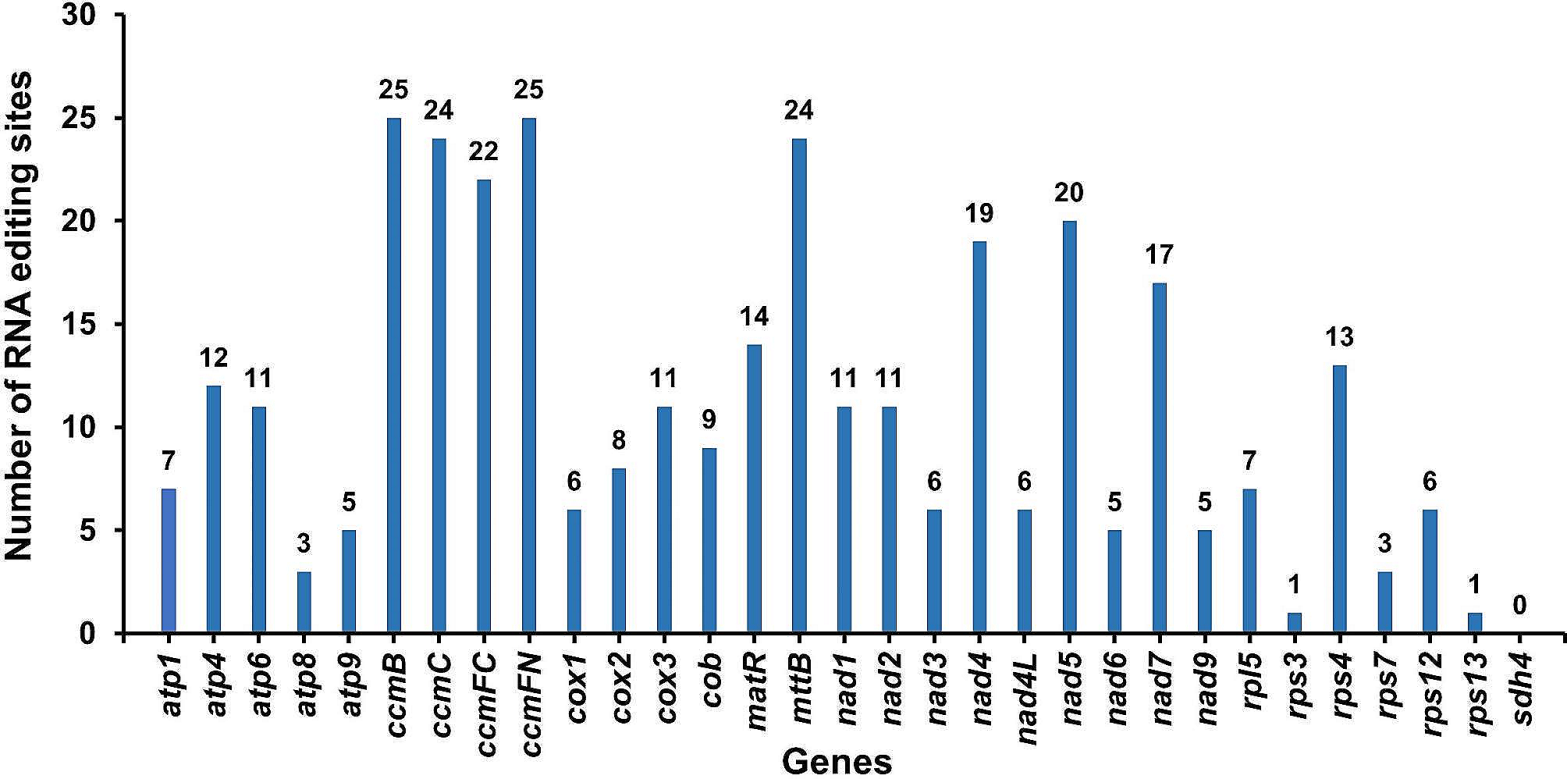
RNA editing sites in *H. ammodendron* mitochondrial genes. This figure presents a comprehensive count of RNA editing sites discovered within 31 mitochondrial protein-coding genes of *H. ammodendron*, shedding light on post-transcriptional modifications and their potential implications

RNA editing, a widespread phenomenon in land plants, entails modifications within the coding region of the transcript involving the addition, loss, or conversion of nucleotides [22]. The current study identified 337 RNA editing sites, all of which were cytidine to uridine (C to U) conversion types. The predicted RNA editing sites in various genes are shown in Fig. 4. The *ccmB* and *ccmFN* genes exhibited the highest number (25) of RNA editing sites. Conversely, no RNA editing sites were observed in the *sdh4* gene. The amino acid transition exhibiting the highest abundance was from serine to leucine, comprising 19.58% (66 sites) of the observed transitions (Table S9).

### Codon usage

**Fig. 5:**
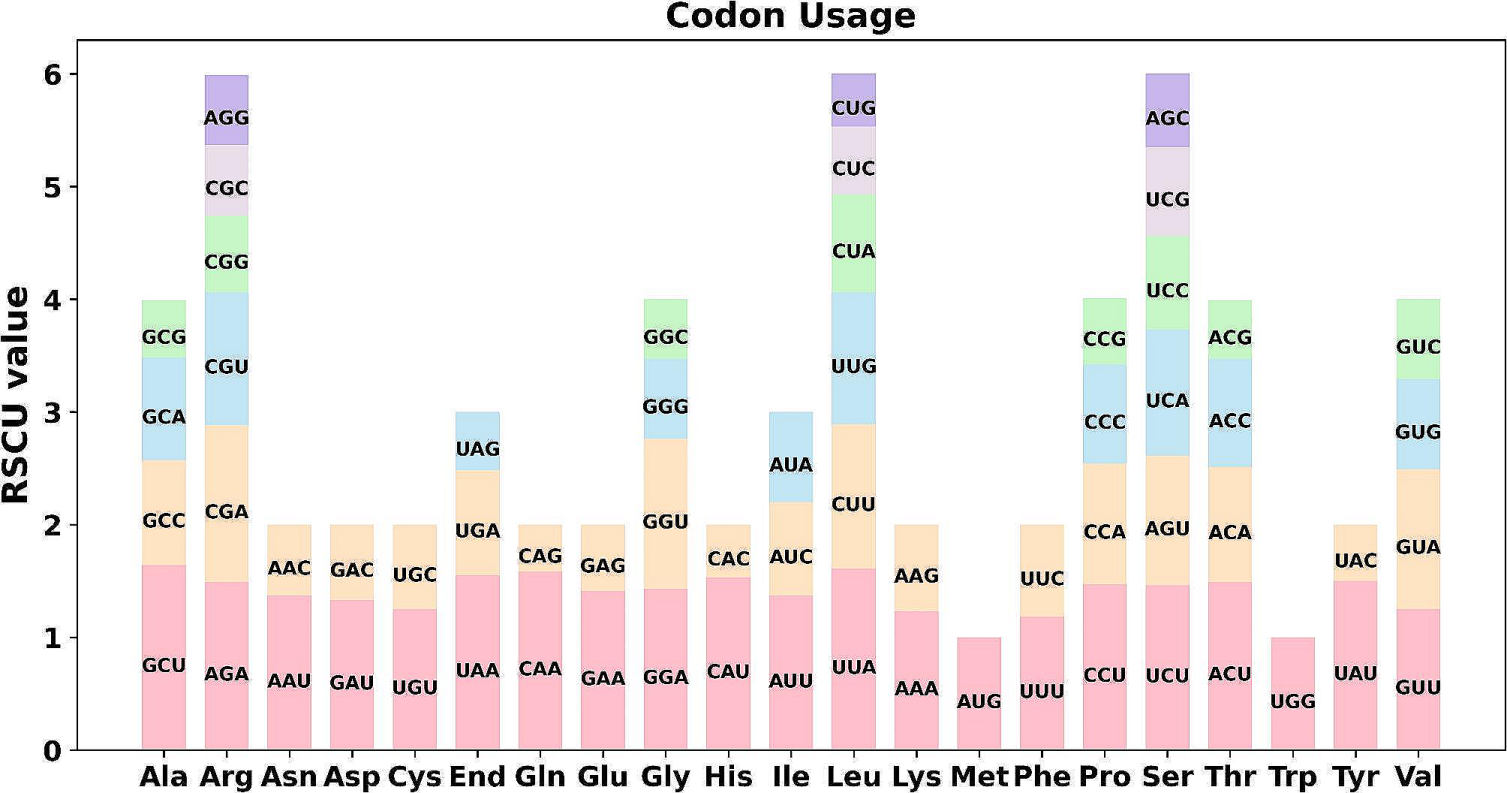
Codon usage preferences in *H. ammodendron*‘s mitochondrial genome. Graphical representation illustrating the Relative Synonymous Codon Usage (RSCU) values. This showcases the preferences of certain codons encoding the same amino acid, hinting at evolutionary pressures and translational optimization

The codon usage pattern of the *H. ammodendron* mtDNA was presented in Fig. 5. Codons exhibiting a relatively synonymous codon usage (RSCU) value greater than one were regarded as being favored by the corresponding amino acids. Apart from the start codon AUG and the tryptophan codon UGG, both of which had an RSCU value of 1, there were widespread codon usage preferences in the *H. ammodendron* mitochondrial protein-coding genes (Fig. 5). For example, alanine (Ala) showed a strong preference for the codon GCU, with the highest RSCU value of 1.64. In addition, leucine (Leu) preferred the codon UUA, with an RSCU value of 1.61 (Table S10).

### Phylogenetic analysis and collinearity analysis

**Fig. 6:**
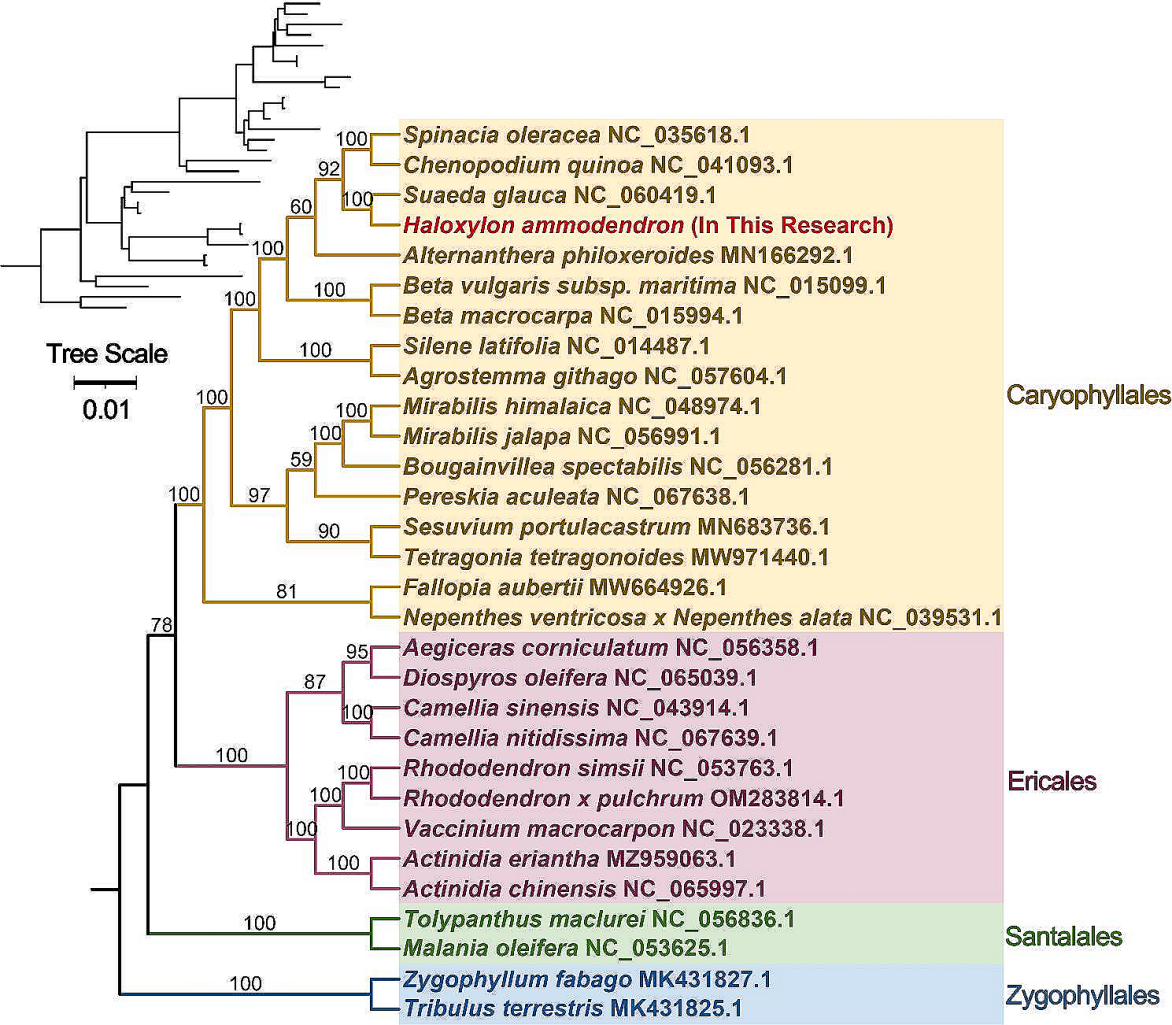
Phylogenetic placement of *H. ammodendron* within Caryophyllales. A Maximum Likelihood phylogenetic tree that delineates the evolutionary relationship of *H. ammodendron* with other species in the Caryophyllales order. Node labels display support values, with maximum-likelihood bootstrap values offering a measure of the tree’s reliability

**Fig. 7:**
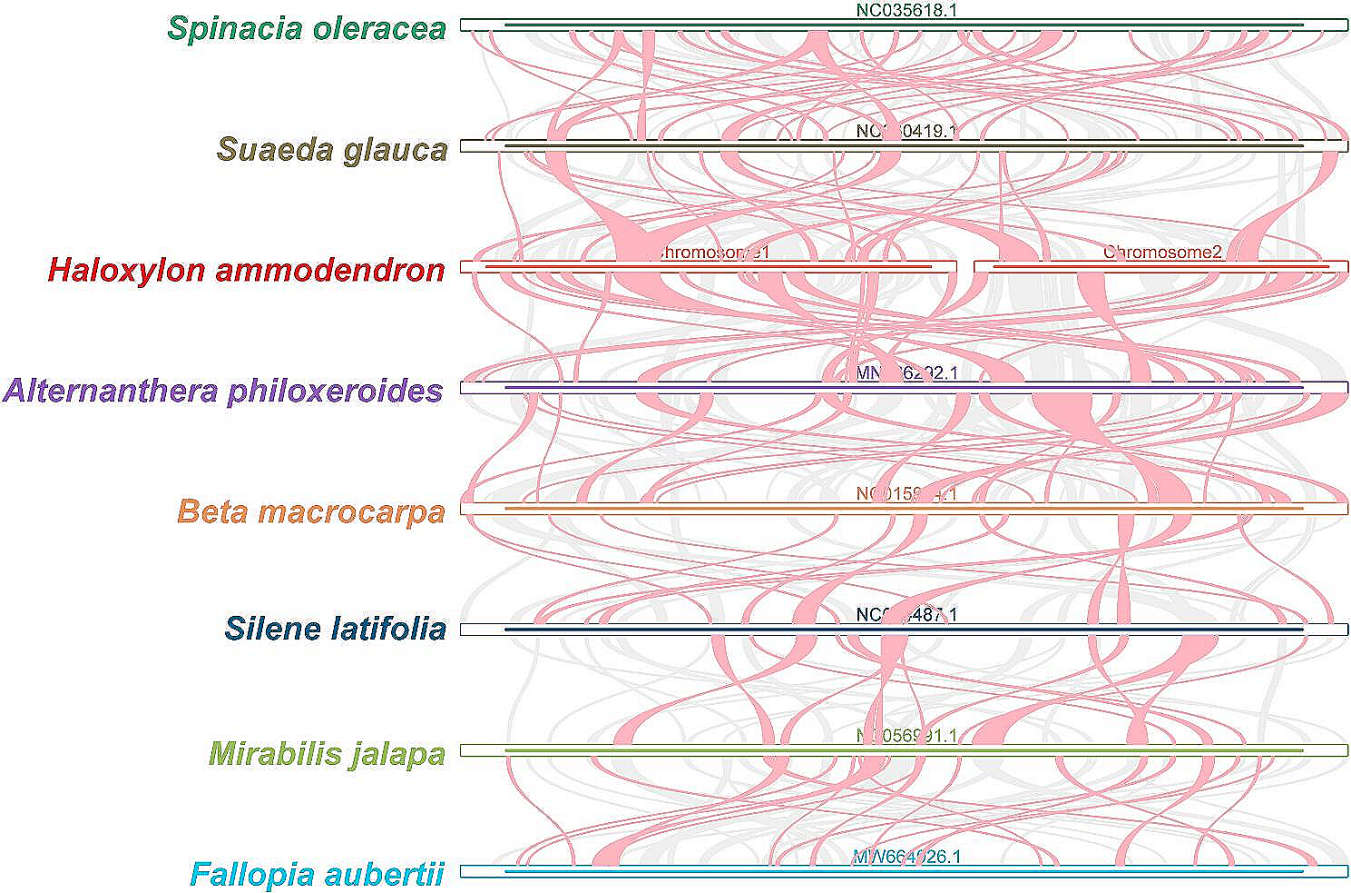
Comparative collinearity in caryophyllales mitogenomes. A landscape view showcasing the genome collinearity between *H. ammodendron* and its Caryophyllales counterparts. The diagram helps in understanding genomic rearrangements, evolutionary events, and conservation levels among these species

The phylogenetic tree based on mtDNA (Fig. 6) was constructed using a maximum likelihood approach, incorporating a diverse set of 30 species representing various orders (Table S11). The resulting tree topology aligns with the most recent classification the Angiosperm Phylogeny Group (APG) provided. *H. ammodendron* and the Amaranthaceae species *Spinacia oleracea*, *Chenopodium quinoa*, and *Suaeda glauca* were placed within the Caryophyllales order. Interestingly, *H. ammodendron* exhibited a close phylogenetic relationship with *S. glauca*, another member of the Amaranthaceae family, widely distributed in the arid areas of northwest China [23]. These results were robustly supported by Bayesian phylogenetic tree inference (Figure S1). To identify both conserved and unique mitochondrial regions among Caryophyllales species, we performed pairwise alignments using *Spinacia oleracea* (NC_035618.1), *S. glauca* (NC_060419.1), *Alternanthera philoxeroides* (MN166292.1), *Beta macrocarpa* (NC_015994.1), *Silene latifolia* (NC_014487.1), *Mirabilis jalapa* (NC_056991.1), and *Fallopia aubertii* (MW664926.1) mtDNA (Fig. 7). The alignment results were provided in Fig. 7 and Table S12. Although collinear blocks were noticeable within Caryoplyllales species, their length is relatively short, indicating a non-conservative mitogenome structure. It is worth pointing out that the homologous block arrangement is inconsistent between *H. ammodendron* and its congeners, implying an evolutionary mtDNA rearrangement occurrence. Additionally, some blank regions were observed in *H. ammodendron*, representing species-specific sequences lacking homology with other species.

## Discussion

In land plants, mtDNA has undergone extensive genomic reorganization and gene arrangements, resulting in rapid structural evolution. Studying plant mtDNA offers a path into their structural organization, genetic diversity, and evolutionary dynamics, which have implications for understanding their functional role in biological processes such as plant growth, energy production, and respiration.

In the present study, we took advantage of both short- and long-read sequencing data to assemble the first mtDNA of *H. ammodendron*. The assembly mitogenome length was 210,149 bp, which is about 50% shorter than *S. glauca* (NC_060419.1, assembly size: 474,330 bp), and *P. aculeate* (NC_067638.1, assembly size: 515,187 bp) but in similar range with *M. jalapa* (NC_056991.1, assembly size: 267,334 bp), all belonging to the same order: Caryophyllales. The observed genome size variation could be imputable to several factors, including repeat elements density, intragenic regions gain or loss, and the introgression of foreign DNA [24–26].

Repeats elements such as microsatellites, tandem and dispersed repeats are prone to recombination leading to isomeric sequence sets in the mitogenome [14, 27, 28]. Therefore, the repeats dynamism could impact DNA maintenance and evolution of the plant mtDNA [16]. Unusually sized repeats within plant mtDNA exert significant influence, marked by their excessive size relative to coding capacity, a low mutation rate in genes, and a substantial rearrangement rate [16]. Besides, ubiquitous short tandem repeats play pivotal roles in mtDNA evolution, contributing to tandem duplications and structural variations [29]. It is worth to mention that homologous recombination, facilitated by rich repeated sequences in plant mtDNA, emerges as a major evolutionary driver [30]. Additionally, repeat-mediated recombination stands out as a highly active and substantial contributor to structural rearrangements in mtDNA [31].

One of the complexities of the mtDNA is the variability in molecular conformation. Several configurations have been documented, including circular, linear, sigma-like, fragmented, and multi-chromosomal structures [30]. Here we reported a multi-chromosomal mitogenome architecture that consists of two circular molecules. Similar findings were reported in other Caryophyllales species, such as *Fagopyrum esculentum* (10 circular chromosomes) [32], and *Fallopia multiflora* (two circular chromosomes) [33]. In the same plant order, a multi-chromosomal architecture with more than 50 chromosomes was also found for one of the symbolic plants, *Silene conica*, well known for its massive mitogenome spanning 11 Mb [14]. However, the close relatives *S. glauca* (NC_060419.1) [18] and *Chenopodium quinoa* (NC_041093.1) [17] present a single molecule in a fully circularized conformation based on long reads data-based assembly. The origin and maintenance of the multi-ring structure in plant mtDNA are areas of ongoing research [34–39]. It is believed that mechanisms such as recombination and rearrangement events, possibly mediated by repeated sequences [40–42], could play a role in generating and maintaining this phenomenon. The balance between the recombination and replication of mitochondrial rings was proposed as a key aspect that could influence the occurrence of mtDNA multi-chromosome structure [43, 44]. Besides, gain or loss of chromosome sets is suggested to be one of the key biological events leading to the diversity of mtDNA structures across different organisms [35], providing insights into the mechanisms driving the mitochondrial evolution.

DNA transfer among chloroplasts and mitochondria allows genetic exchange between cellular compartments and can serve as a driver for expansion or contraction of mitochondrial genomes in plants. Our study detected sequence transfer between the cpDNA and the mtDNA, indicating possible horizontal or intracellular gene transfer [45]. Sequences exchange between mtDNA and cpDNA was also noted for other Caryophyllales members including *Mammillaria huitzilopochtli* [46].

RNA editing is a post-transcriptional mechanism in higher plant organelles, contributing to improved protein folding [47]. In angiosperm mitochondria, extensive cytidine to uridine RNA editing occurred, playing a crucial role in transcript expression [48]. Interestingly, all protein-coding genes analyzed from *H. ammomendron* mtDNA showed C- to-U conversion, which may affect the start or end positions of the coding genes. It is worth noting that proteins translated from edited RNAs could play a critical function in an environment change context, resulting in plant acclimatization and survival capabilities [49]. Indeed, *H. ammodendron* is well known for its remarkable tolerance to saline and dry environments [50, 51].

The maximum likelihood phylogenetic inference resulted in a tree with strong support values, placing *H. ammodendron* in the close relative taxon *S. glauca*. Our tree topology is in perfect agreement with the recent results based on the Angiosperm Phylogeny Group (APG IV) classification [52], ITS [53], plastid markers [54], the recent nuclear whole genome sequence data [10], and the Bayesian phylogenetic tree indicating the reliability of the mitogenome-based tree.

## Conclusions

In the present study, the mtDNA of *H. ammodendron* was characterized. The mtDNA exhibited a multi-chromosomal conformation with two circular molecules with a total length of 210,149 bp. It contains 31 protein-coding genes, 18 tRNA and 3 rRNA. A set of 17 sequence fragments were found homologous between the mtDNA and cpDNA, spanning 5,233 bp, accounting for 2.49% of the mitogenome. RNA editing assessment revealed that the protein-coding genes were mainly subjected to cytidine-to-uridine conversion. The phylogenetic inference showed a close relationship with *S. glauca*. The mtDNA resource provided by this study laid a foundation for further investigations into the comparative evolution and functional role of the *H. ammodendron* mtDNA.

## Methods

### Assembly and annotation

Before the mtDNA assembly, short-reads data were trimmed using fastp v0.23.4 [55]. To assemble the *H. ammodendron* mitogenome, a hybrid approach encompassing both short and long-read sequencing data was implemented. Firstly, using the Illumina short-reads data, a draft mtDNA assembly was conducted with GetOrganelle v1.7.5 [19] with the following settings:-R 15 -k 21,45,65,85,105 -F embplant_mt. The resulting genome graph sketch was then visualized using Bandage v. 0.8.1 [20], manually inspected and curated for node junctions. Using minimap2.2.26 (r1175), we mapped PacBio HiFi data onto the initial assembly to address conflictual nodes and repetitive regions [56].. In cases where multiple alternative connections exist at branching nodes, preference was given to connections supported by long reads. The obtained assembly was validated by mapping both short and long reads to ensure the connectivity and consistency of the assembly.

The assembly was annotated with GeSeq online tool (https://chlorobox.mpimp-golm.mpg.de/geseq.html#) [57] with the following parameters: *Arabidopsis thaliana* (NC_037304), *Nicotiana tabacum* (NC_ 006581), *Glycine max* (NC_020455), and *Suaeda glauca* (NC_060419.1) were set as reference mitogenomes; BLAT search (protein search identity = 25, rRNA, tRNA, DNA search identity = 85) for coding protein genes, rRNA and tRNA prediction. Additionally, tRNAscan-SE v2.0.7 [58] was used for tRNA prediction with a cut-off score for reporting tRNAs = 15. The annotated mtDNA was visualized using Apollo v2.7.0 [59] for manual correction. The genome map was rendered using the Organellar Genome DRAW (OGDRAW) tool [60].

### Repeat sequence analysis

The identification of repetitive sequences, including microsatellite sequence repeats (SSRs), tandem repeats, and dispersed repeats, was performed using MISA (https://webblast.ipk-gatersleben.de/misa/) [61], TRF (https://tandem.bu.edu/trf/trf.unix.help.html) [62], and the REPuter (https://bibiserv.cebitec.uni-bielefeld.de/reputer/) [63] web servers respectively.

### RNA editing and codon usage bias analyses

The protein-coding sequences of the mitochondrial genome of *H. ammodendron* were extracted using the PhyloSuite toolbox [64]. Prediction of RNA editing sites was performed using the online tool PREPACT3 (http://www.prepact.de/) [65], while MEGA v7.0 software [66] was employed to calculate the relative synonymous codon usage (RSCU).

### Collinearity analysis

Using the BLASTN v2.10.1 + tool, we conducted pairwise alignments to assess sequence similarity between *H. ammodendron* and its related species (*Spinacia oleracea*, *Suaeda glauca*, *Alternanthera philoxeroides*, *Beta macrocarpa*, *Silene latifolia*, *Mirabilis jalapa*, *Fallopia aubertii*) [67]. Homologous sequences exceeding 500 bp were retained as conserved collinear blocks for constructing the multiple synteny plot with the help of MCscanX implemented in TBTools v1.098746 [68].

### Gene transfer analysis

To detect the putative gene transfer between cpDNA and mtDNA, we performed a *de novo* assembly and annotation of the cpDNA of our sample using GetOrganelle v1.7.5 [19] and GeSeq web server [57], respectively. Then, an alignment of organelles was performed using BLAST v2.10.1+ [67] with the following settings: E-value ≤ 1E-10, matching rate ≥ 70%, and matching length ≥ 40 bp. Utilizing the genome of *H. ammodendron*, we applied a similar approach by aligning the mtDNA onto the nuclear genome to investigate the sequence transfer between the mtDNA and the nuclear genome.

### Phylogenetic analysis

To infer the mtDNA-based phylogenetic tree, a set of 15 conserved genes (*atp1*, *atp4*, *atp6*, *atp8*, *ccmB*, *ccmC*, *ccmFC*, *ccmFN*, *cox2*, *matR*, *nad1*, *nad2*, *nad3*, *nad5*, and *nad6*) from 30 species belonging to four orders (Caryophyllales, Ericales, Santalales, and Zygopgyllales) (Table S10) were extracted from each mtDNA and aligned using MAFFT v7.505 [69]. Poorly aligned sequence regions were trimmed with trimAl v 1.4.1 [70]. The resulting data matrix was then concatenated with PhyloSuite [64] (Supplementary file 1), and the tree was constructed using IQ-TREE v2.0.3 following the Maximum Likelihood approach with the GTR + I + G model [64]. To assess the tree topology, a Bayesian method was performed using MrBayes v3.2.6 tool [71] with the following settings: number of substitution types: 6; Model: 4by4; rates variation across sites: invgamma; number of generations: 10,000, sample frequency: 10; Burnin: 250. The tree was rendered with the Interactive Tree of Life tool (iTOL) v5.0 [72].

### Electronic supplementary material

Below is the link to the electronic supplementary material.

**Supplementary Material 1: Supplementary Table 1.** Haloxylon ammodendron sequence reads archive information of short and long reads data. **Supplementary Table 2. **Using long-reads to validate potential recombinant DNA sequences based on BLASTn. **Supplementary Table 3.** The percentage of different genome configurations identified by long-reads in the Haloxylon ammodendron mitogenome. **Supplementary Table 4. **Results of sequence similarity analysis between chloroplast and mitochondrial genomes of Haloxylon ammondendron. **Supplementary Table 5.** Alignment of nuclear and mitochondrial genomes of Haloxylon ammodendron. **Supplementary Table 6.** Microsatellite repeats detected on Haloxylon ammodendron mitogenome. **Supplementary Table 7.** Tandem repeats results identified in Haloxylon ammodendron mitogenome. **Supplementary Table 8.** Dispersed repeats results detected in Haloxylon ammodendron mitogenome. **Supplementary Table 9.** RNA editing sites and conversion type within Haloxylon ammodendron mitochondrial genome. **Supplementary Table 10.** Relative synonymous codon usage of various amino acids in the Haloxylon ammodendron mitochondrial genome. **Supplementary Table 11.** List of species employed in the phylogenetic tree construction. **Supplementary Table 12.** Collinearity analysis results within the selected Caryophyllales species

**Supplementary Material 2: Supplementary Figure 1.** Bayesian-based phylogenetic tree. The Bayesian tree inference was conducted using the MrBayes tool based on a conserved gene set. Node support values are highlighted in red. The tree was inferred using the 4by4 model. The tree was visualized using etetoolkit online viewer http://etetoolkit.org/treeview/

**Supplementary Material 3: Supplementary file 1.** Alignment matrix used for the phylogenetic tree inference

## Data Availability

The data underpinning the conclusions drawn in this study are accessible in both the main manuscript and its supplementary information appendices. The PacBio Hi-Fi and Illumina sequencing reads are retrievable via the Sequence Read Archive accession numbers SRR17129371 and SRR17127859, respectively. Furthermore, the assembled sequences corresponding to chromosome 1 and chromosome 2 of the mitogenome have been submitted to the National Center for Biotechnology Information and are retrievable under the GenBank accession numbers OR296702 and OR296703, respectively.
