# Supplementary material for: Insights into the multi-chromosomal mitochondrial genome structure of the xero-halophytic plant Haloxylon Ammodendron (C.A.Mey.) Bunge ex Fenzl
Source: BMC Genomics. 2024 Jan 29;25:123. doi: 10.1186/s12864-024-10026-6 (PMC10823707; doi:10.1186/s12864-024-10026-6)
Supplement: Supplementary file 2 — Supplementary Material 2: Supplementary Figure 1. Bayesian-based phylogenetic tree. The Bayesian tree inference was conducted using the MrBayes tool based on a conserved gene set. Node support values are highlighted in red. The tree was inferred using the 4by4 model. The tree was visualized using etetoolkit online viewer http://etetoolkit.org/treeview/ [file 12864_2024_10026_MOESM2_ESM.docx]

**Supplementary Figures**


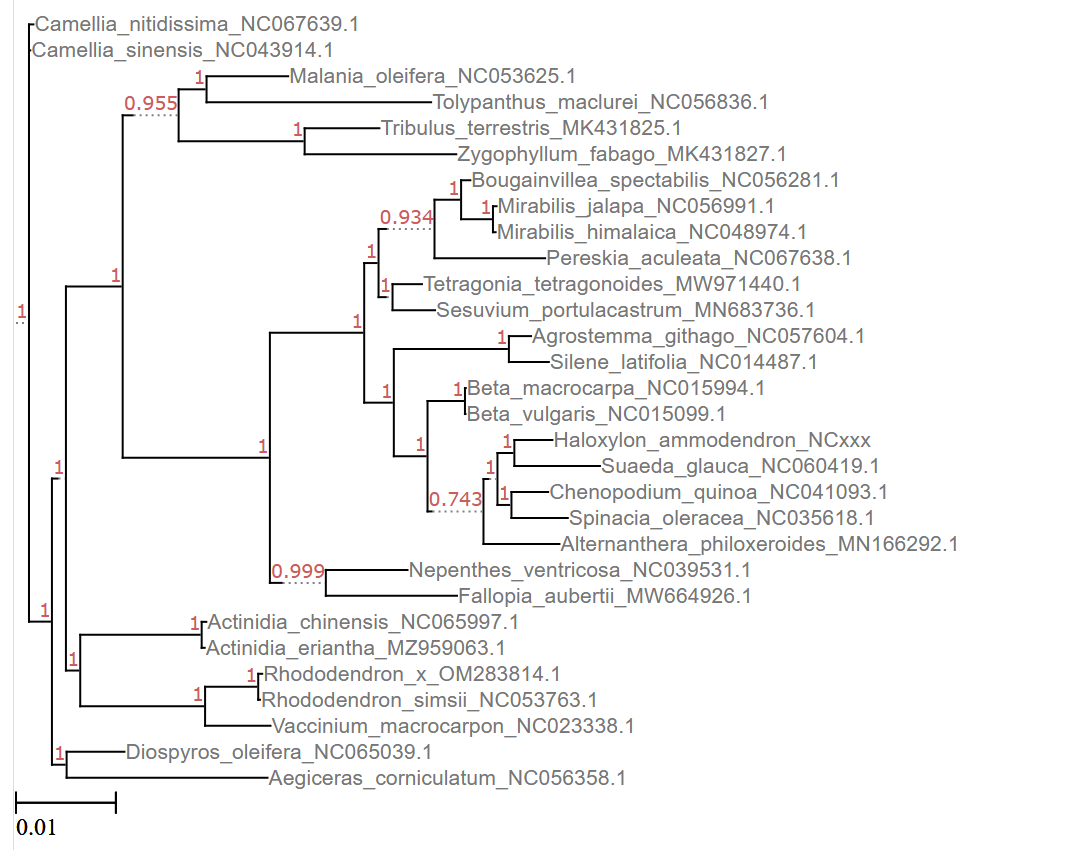


**Figure S1.** Bayesian-based phylogenetic tree. The Bayesian tree inference was conducted using the MrBayes tool based on a conserved gene set. Node support values are highlighted in red. The tree was inferred using the 4by4 model. The tree was visualized using etetoolkit online viewer http://etetoolkit.org/treeview/.
