# Supplementary material for: Insights into the multi-chromosomal mitochondrial genome structure of the xero-halophytic plant Haloxylon Ammodendron (C.A.Mey.) Bunge ex Fenzl
Source: BMC Genomics. 2024 Jan 29;25:123. doi: 10.1186/s12864-024-10026-6 (PMC10823707; doi:10.1186/s12864-024-10026-6)
Supplement: Supplementary file 3 — Supplementary Material 3: Supplementary file 1. Alignment matrix used for the phylogenetic tree inference [file 12864_2024_10026_MOESM3_ESM.docx]

**Supplementary file 1** : Alignment matrix used for the phylogenetic tree inference

>Camellia nitidissima NC067639.1

-TGATACTTTCTGTTTTGTCAAGCCCTGCTTTGGTCTCTGGTTTGATGGTTGTACGTGCTAAAAATCCGG

TACATTCCGTTTCGTTTCCCATCCCAGTCTTTCGCGACACTTCAGGTTTACTTCTTTTGTTAGGTCTCGA

CTTCTTCGCTATGATCTTCCCAGTAGTTCATATAGGAGCTATAGCCGTTTCATTCCTATTCGTTGTTATG

ATGTTCCATATTCAAATAGCGGAGATTCACGAAGAAGTATTGCGCTATTTACCAGTGAGTGGTATTATTG

GACTGATCTTTTGGTGGGAAATGTTCTTCATTTTAGATAATGAAAGCATTCCATTACTACCAACCCAAAG

AAATACGACCTCTCTGAGATATACGGTTTATGCCGGAAAGGTACGAAGTTGGACTAATTTGGAAACATTG

GGCAATTTACTTTATACCTACTATTCCGTCTGGTTTTTGGTTCCTAGTCTTATTTTATTAGTAGCCATGA

TTGGGGCTATAGTACTGACTATGCATAGGACTACTAA------GGTGAAAAGACAGGATGTATTCCGACG

AAATGCTATTGATTCTAGGAGGACTATAATGAGGAGGACGACAGACC--TGTCAATATATGAATTGTTTC

ATTATTCGTTATTTCCGGGTCTTTTCGTTGCATTCACTTACAACAAGAAACAACCACCAGTGTTTGGTGC

AGCACCTGCATTTTGGTGCATTCTTCTTTCTTTCCTTGGTCTTTCGTTCCGTCATATTCCTAATAACTTA

TCCAATTACAACGTATTAACTGCTAATGCACCTTTCTTTTATCAAATCTCAGGGACATGGTCTAATCATG

AGGGTAGTATTTTATCATGGTGTCGGATCCTAAGTTTTTATGGATTTCTTCTTTGTTACCGGGGTCGACC

CCAAAGCCATAATGTCTCAAAACGAGGAGGCCATAGAGAAACTCTTTTTTATTCCTTTGTCTTGAACTTC

GTGAAGAACTCCATTCTACCTCTCCCTCGTTACGAACAAAAAAGTGGGGCT------GCGCCCCAGTTGT

ACACTCCCTTCGTTCTACGAA---CCCTTGTTGATTCTGAACTTCGTTCGCGAAGGAACCGGACTTTTGA

CGGGCCAGCCCTTTTTTATGCGCCGCTTTACCCTGAAAGGAAAATGAGCTTTGCTCCTCTGGGCGCTAGG

CGCTCCCGTGGTTCGCGAGAAGGAAAAAGGACTCATCCTTTGTTGCATCTGGCACGAGATGATAAAGAGA

GAGCTTCGTCTATCGATGAACAGCGGATTGACGGAGCTCTTGGCATTGCTTTGTTTTTCTCTCCTTTCCT

ATCAGCGAGTTCCGATCCTTTTGTTCGAAATTTCTTCGTTCGTACCGAACCGCTTGCAGAATCAAATCCT

GTTCCACAAGATCCTATATCAGCTATACATCCTCCTTGCATTTATGCCGGAGACGTCGCCAGTGCTATGG

GCTTTGGCTTATGTAGATCAAAAATGATGAATGGGATTGCGGCACTCCACTCGCCGCCAATGCGGAAGGA

TGCCGCCGAAAAGAATGGAACGCTGTTTCGCTCTGCTGGATGCGTCGGATCCCGTATAACAAGCGAGCTC

TTTACCCTCAAATTCAAACATGTGGGCGCAAAATGCTATCCTGCTCTATTCTTACGTAGCAATAGAAGCC

TGCTT---ATGCTGCTTCGGCGGCGCTTTTTCGCCTTCTCTTCGCTCTGGACAGGAGCGCTAGTGGACGC

GGGGAGGGAGCA------GGCGAAGCCTGTCG------TTCGTAATGGAAAGAAAGATACCACTACTTCG

CCTCTTTGTTGGACCGCCGGCGCGAACACAGTGGTCTCTGACCAGGACCAGGAACCAATTCGAATTTGGA

TCTTGACATGTCGGTGGTTTTTAACCGTAGGCATCTTGCCAGGAAGTTGGTGGGCTCATCATGAATTAGG

TCGGGGTGGCTGGTGGTTTCGGGATCCCGTAGAAAATGCTTCTTTTATGCCTCGGGTATTAGCCACAGCT

CGTATTCATTCAGTAATTCTACCCCTTCTTCATTCTTGGACCTCGCTTCTTAATATTGTGACTCTTCCAT

GCTGTGTCTCAGGAACCTCTTCAATACGGTCCGGATTGCTAGCTCCCGTTCATAGTTTTGCTACAGATGA

TACACGAGGAATCTTTTTATGGCGGTTCTTCCTTCTAATGACCGGCATATCTATGATTCTTTTCTCCCAG

ATGAAGCAGCAGGCATCGGTCCGTAGAACCTATAAAAAAGAGATGGTTGTGGCGCGAAGTACTCTTGTGC

ACCTACGT--ATGATTGTTCTAGAATGGCGATTCCTCACAATTGCTCCTTGTGATGCAGCAGAACCATGG

CAATTAGGATCTCAAGACGCAGCAACACCTATGATGCAAGGAATAATAGACTTACATCACGATATCTTTT

TCTTCCTCATTCTGATTTTGGTTTTCGTATCACGGATCTTGGTTCGCGCTTTATGGCATTTCCACTATAA

AAAAAATCCAATCCCGCAAAGGATTGTTCATGGAACTACTATCGAGATTCTTCGGACCATATTTCCTAGT

ATCATCCCGATGTTCATTGCTATACCATCATTTGCTCTGTTATACTCAATGGATGAGGTAGTAGTAGATC

CAGCCATTACTATCAAAGCTATTGGACATCAATGGTATCGGACTTATGAGTATTCGGACTATAACAGTTC

CGATGAACAGTCACTCACTTTTGACAGTTATACGATTCCAGAAGATGATCCAGAATTGGGTCAATCACGT

TTATTAGAAGTGGACAATAGAGTGGTTGTACCAGCCAAAACTCATCTACGTATTATTGTAACACCTGCTG

ATGTACCTCATAGTTGGGCTGTACCTTCCTCAGGTGTCAAATGTGATGCTGTACCTGGTCGTTTAAATCA

GACCTCTATTTCGGTACAACCAAAAGGAGTTTACTATGGTCAGTGCAGTGAGATTTGTGGAACTAATCAT

GCCTTTACGCG-----------------------------------------------------------

------------------------TGAGACGACTCTTTCTTGAACTATATCATAAACAGATCTTCCCCTC

CACACCAATCACGAGTTTTTCTCCATTCCTCTCGTATATCGTCGTAACGCCCTTAATGCTAGGTTTTGAA

AAAGACTTTTCATGTCATTCCCATTTAGGTCCGATTCGGATCCCTCCGTTGTTTCCTTTTCCTTCCGCAC

CTTTTCCTCGAAATGAGAAAGAAGATGGTACACTTGAATTGTATTATTTAAGTGCTTATTGCTTGCCAAA

GATCCTACTTCTACAATTGGTAGGTCACCGGGTTATTCAAATAAGTCGTGTTTTCCGTGGTTTTCCCATG

TTACAACTTCCGTACCAATTCGGTCGATCCGGAATGGATCGGTTAAACATTCTATTAGGGAGCCTGGTCT

TGACTCTTCTGTGTGGTATTCATTCTCGTTCGGCTCTTGGAATCACATCCAGCAGTGGTTGGAACAGCTC

GCAAAATCCAACCACTTCACCTACTTCATTGCCCCCAACCGTTTCTCGTACCTCTATTGAAACAGAATGG

TTTCATGTTCTTTCATCGATTGGTTATTCCTCTCCGTTCGTATCTCTTTTTCCAATTTCGGTCTCGATTA

GTTTACAAGATTGA-TGTCCGTTTCGTTATTACAACCTTC-----TTTTTTGATGTCAAAGACCAGAAGC

TACGCGCAAATTCTCATTGGATCTCGGTTGTTCTTAACAGCGATGGCTATTCATTTAAGTCTTCGGGTAG

CACCACTAGATCTTCAACAAGGTGGAAATTCTCGTATTCCGTATGTACATGTTCCTGCGGCTCGGATGAG

TATTCTTGTTTATATCGCTACGGCTATAAACACTTTCTTGTTCCTATTAACAAAACATCCCCTTTTTCTT

CGCTCTTCCGGAACCGGTACAGAAATGGGTGCATTTTCTACGTTGTTTACCTTAGTTACTGGGGGGTTTC

GGGGAAGACCTATGTGGGGCACCTTTTGGGTGTGGGATGCTCGTTTAACCTCTGTATTCATCTCGTTCCT

TATTTACCTGGGTGCACTGCGTTTTCAAAAGCTTCCTGTCGAACCGGCTCCTATTTCAATCCGTGCTGGA

CCGATCGATATACCAATAATAAAGTCTTCAGTCAACTGGTGGAATACATCGCATCAACCTGGGAGCATTA

GCCGATCTGGTACATCAATACATGTTCCTATGCCCATTCCAATCTTGTCTAACTTTGCTAACTCCCCCTT

CTCAACCCGTATCTTGTTCGTTCTGGAAACACGTCTTCCTATTCCATCTTTTCTCGAATCTCCTTTAACG

GAAGAAATAGAAGCTCGAGAAGGAATACC-GCAGGCTAGAAAGATGCTATTTGCTGCTATTCTATCTATT

TGTGCATCAAGTTCGAAGAAGATCTCAATCTATAATGAAGAAATGATAGTAGCTCGTTGTTTTATAGGCT

TTATCATATTCAGTCGGAAGAGTTTAGGTAAGACTTTCAAAGTGACTCTCGACGGGAGAATCCAGGCTAT

TCAGGAAGAATCGCAGCAATTCCCCAATCCTAACGAAGTAGTTCCTCCGGAATCCAATGAACAACAACGA

TTACTTAGGATCAGCTTGCGAATTTGTGGCACCGTAGTAGAATCATTACCAATGGCACGCTGTGCGCCTA

AGTGCGAAAAGACAGTGCAAGCTTTGTTATGCCGAAACCTAAATGTTAAGTCAGCAACACTTCCAAATGC

CACTTCTTCCCGTCGCATCCGTCTTCAGGACGATCTAGTCACAGGTTTTCACTTCTCAGTGAGTGAAAGA

TTTGTCCCCGGGTGTACGTTGAAAGCTTCTATAGTAGAACTCATTCGAGAGGGCTTGGCGGTCTTAAGAA

TGGTTCGGGTGGGGGGTTTCTCTT-TGAAAGAGGCGATCAGAATGGTACTCGAATCCATTTACGATCCCG

AGTTTCCAGACACATCGCACTTCCGCTCGGGTCGAGGCTGCCACGCGGCCCTAAGACGGATCAAAGAAGA

GTGGGGAACCTCTCGCTGGTTTTTGGAATTCGACATCAGGAAGTGTTTTCACACCATCGACCGACATCGA

CTCATCTCAATCTTTAAGGAAGAGATCGACGATCCCAAGTTCTTTTACTCCATTCAGAAAGTCTTTTCCG

CCGGACGACTCGTAGGAGGTGAGAAGGGCCCTTACTCCGTTCCACACAGTGTACTACTATCGGCCCTACC

AGGCAACATCTACCTACACAAGCTCGATCAGGAGATAGGGAGGATCCGACAGAAGTACGAAATTCCGATT

GTTCAGAGAATCAGATCGGTTCTATTAAGGACAGGTCGTATTGATGACCAAGAAAACTCTGGAGAAGAAG

CAATGGAAATCTCTCCCAGAGCTGCGGAACTAACGACTCTATTAGAAAGTAGAATTACCAACTTTTACAC

GAATTTTCAAGTGGATGAGATCGGTCGAGTGGTCTCAGTTGGAGATGGGATTGCACGTGTTTATGGATTG

AACGAGATTCAAGCCGGGGAAATGGTTGAATTTGCCAGCGGTGTGAAAGGAATAGCGTTGAATCTTGAGA

ATGAGAATGTAGGGATTGTTGTCTTTGGTAGTGATACCGCTATTAAAGAAGGAGATCTTGTCAAGCGCAC

TGGATCTATTGTGGATGTTCCTGCGGGAAAGGCTATGCTAGGGCGTGTGGTCGACGCGTTGGGAGTACCT

ATTGATGGAAGAGGGTCTCTAAGCGATCACGAGCGAAGACGTGTCGAAGTGAAAGCCCCTGGGATTATTG

AACGTAAATCTGTGCACGAGCCTATGCAAACAGGGTTAAAGGCGGTAGATAGCCTCGTTCCTATAGGCCG

TGGTCAACGAGAACTTATAATCGGGGATCGACAAACTGGAAAAACAGCTATTGCTATCGATACCATATTA

AACCAAAAGCAAATGAACTCAAGGGGCACCTCTGAGAGTGAGACATTGTATTGTGTCTATGTAGCGATTG

GACAGAAACGCTCAACTGTGGCACAATTAGTTCAAATTCTTTCAGAAGCGAATGCTTTGGAATATTCCAT

TCTTGTAGCAGCCACCGCTTCGGATCCTGCTCCTCTGCAATTTCTGGCCCCATATTCTGGGTGTGCCATG

GGGGAATATTTCCGCGATAATGGAATGCACGCATTAATAATATATGATGATCTTAGTAAACAGGCGGTGG

CATATCGACAAATGTCATTATTGTTACGCCGACCACCAGGCCGTGAGGCTTTCCCAGGGGATGTTTTCTA

TTTACATTCCCGTCTCTTAGAAAGAGCCGCTAAACGATCGGACCAGACAGGCGCAGGTAGCTTGACCGCC

TTACCCGTCATTGAAACACAAGCTGGAGACGTATCGGCCTATATTCCCACCAATGTGATCCCCATTACTG

ATGGACAAATCTGTTCGGAAACAGAGCTCTTTTATCGCGGAATTAGACCTGCTATTAACGTCGGCTTATC

TGTCAGTCGCGTCGGGTCTGCCGCTCAGTTGAAAGCTATGAAACAAGTCTGCGGTAGTTCAAAACTGGAA

TTGGCACAATATCGCGAAGTGGCCGCCCTTGCTCAATTTGGGTCAGACCTTGATGCTGCGACTCAGGCAT

TACTCAATAGAGGTGCAAGGCTTACAGAAGTCCCGAAACAACCACAATATGCACCACTTCCAATTGAAAA

ACAAATTCTAGTCATTTATGCAGCTGTCAATGGATTCTGTGATCGAATGCCATTAGAAAAAATTTCTCAA

TATGAGAGAGCCATTCCAAGTAGTGTAAAACCAGAATTACTACAATCTCTTTTAGAAAAAGGTGGGTTAA

CTAACGAAAGAAAGATGGAACCAGATGCATTCTTAAGAGAAAGTGCTTTGCCTTACCTATGATGCAAGAA

AGAATGG-CATCCAACGCAAAGCGGCCTTTCATTCCCTTGTTTCGTCGTGGCACACCCCCCCCACAAGCA

CCCCCCGGCTCAGGGGGGACCAGAAAACGCCTTTCGTTTTCCCCCCTTCGTCGGCCCTTGCCGCCTTCCT

TAACAAGCCCTCGAGCCTCCTTTGCGCCGCCTTCCTCATAGAAGCCGCTGGGTTGACCCCGAAGGCCGAA

TTCTATGGTAGAGAAGGCTGTAATAATAATTGGGCCATGAGAGACTTTATTAAGTATTGCAAAAGAAAGG

GCCTGCTGATAGAGCTGGGCGGGGAGGCGATACTAGTTATCAGGTCAGAGAGGCGCCTGGCCCGTAAGCT

GGCCCCCTTAAAAACCCATTACTTAATAAGGATTTGTTACGCGCGATATGCCGACGACTCACTACTGGGA

ATCGTGGGTGCCGTAGAGCTTCTCATAGAAATACAAAAACGTATCGCCCACTTCCTACAATCCGGCCTGA

ACCTTTGGGTAAGCTCTGCAGGATCAACAACAATAACTGCACGGAGTACGGTAGAATTCCTCGGTACGAT

CATTCGGGAAGTCCCTCCGAGGACGACTCCCATACAATTCTTGCGAGAGCTGGAGAAGCGTCTACGGGTA

AAGCACCGTATCCATATAACTGCTTGCCACCTACGCTCCGCCATCCATTCCAAGTTTAGGAACCTAGGGA

ATAGTATCCCGATCAAACAGCTGACGAAGGGGATGAGCGGAACAGGGAGTCTACTGGACGCGGTTCAACT

AGCGGAGACTCTTGGAACAGCTGGAGTAAGAAGTCCCCAAGTGAGCGTATTATGGGGGACCGTCAAGCAC

ATCCGGCAGGGATCAAGGGGGATCTCGTTGTTGCATAGCTCAGGTCGGAGCAAGGTGCCATCGGACGTTC

AACGGGCAGTCTCACGATCGGGCACTCATGCCCGGAAGTTGTC---ATTGTATACTCCCGCGGGTCGGAA

GGCGGCGGGGGAAGGAGGGGGACACTGGGCGAGATCTATCAGCAGCGAATTCCCCATACAAATAGAGGCG

CCTATCAAAAAGATACTCCGAAGGCTTCGGGATCGAGGTATCATTAGCCGAAGAAGACCCTGGCCAATCC

ACGTGGCCTGCTTGACGAACGTCAGCGACGGAGACATCGTAAATTGGTCCGCGGGCATTGCGATAAGTCC

TCTGTCCTACTACAGGTGCCGCGACAACCTTTACCAAGTCCGAACGATTGTCGACCACCAGATCCGCTGG

TCTGCAATATTCACCCCGGCCCACAAGCACAAATCCTCGGCGCGGAATATAATCCCAAAGTACTCCAAAG

ACTCAAATATAGTAAATCAAGAAGGTGGTAAGACCCTTGCAGAGTTCCCCAACAGCATAGAGCTTGGGAA

GCTCGGACCCGGTCAAGATCC-GAACAACAAGGAGCACTCAACTACTA-----------------ATGGT

CCAACTACATAACTTTTTCTTTTTCATTACTTCCATGGTCGTGCCTCGTGGCACGGCAGCACCCGTACTA

TTGAAATGGTTCGTCAGTAGAGATGTTCCCACAGGTGCCCCTTCTTCCAATGGTACTATAATTCCTATTC

CTATCCCTTCATTCCCTCTTTTGGTCTATCTACATTCCAGGAAATTCATACGCTCCACGGACGGAGCAAA

AAGTGGAGTCTTGGTCAGAGCAAGCCGCCCTATTCTAT------TACCAGACATAATTGGGAGAAGCTCA

TCCGAAACTAGAGCAAGAAACGCCTCATTTCGTTTTGTTCCTGTTCTTCATTTCCTTCTTCTTCAATCCA

A------GGGGGACTTCTCATATTTAGAATCTTTCTGCGGTGTGCTCCGTTTACTATTCTTTCGTACTTT

CTTCTTTTTACCACGCGATAGGTCAGCGAAGGGTGAGCGGGCGCGGAGAAGGAAAGGCCAAACACTTCGG

CCTAAC------GGGAATGAGCAACGACGAAATGACAAGATGAGGTGCCCCGGGCATCCCCATT------

TAGA---AAGAAGGATCGAAGGTTTTGGGCCTGTAGCTTTCCCCGTCCCCCCTTCGTCGGGTGGTCCTTG

TGTGGGGGGTGCGCCACCAGAAATCGGGCTTGAAGCTCTCGCCTTACCAACGAGCCGACAGCTGATGGCT

GTTGGTCACGACTACTACCAAAAAGCTCCAATGAAGATGAATATTTCACATGGAGGAGTGTGCATCTGTA

TGTTGGGTGTTCTTCTG----TACATAGCTGTTCCAGCTGAAATACTTGGAATAATTCTACCACTTCTAC

TGGGAGTAGCCTTTTTAGTGCTAGCTGAACGTAAAGTAATGGCTTTTGTGCAACGTCGAAAGGGTCCTGA

TGTAGTGGGATCGTTTGGATTGTTACAACCTCTAGCAGATGGTTTGAAATTGATTCTAAAAGAACCTATT

TCACCAAGTAGTGCTAATTTCTCCCTTTTTAGAATGGCTCCAGTGGCTACATTTATGTTAAGTCTGGTCG

CTCGGGCCGTTGTACCTTTTGATTATGGTATGGTATTGTCAGATCCGAACATAGGGCTACTTTATTTGTT

TGCCATATCTTCGCTAGGTGTTTATGGAATTATTATAGCAGGTCGGTCTAGTATTTATTATATACGCTTA

GCGAAAAGAATGTTTTTTGATACACCTAGGACATGGATTCTATATGAACCAATGGATCGTGACAAGTCGT

TACTACTAGCAATGACTTCCTCTTTCATTACTTCATTCTTTCCATATCCCTCTCCTTTGTTCTCAGTTAC

TCATCAAATGGCACTCAGTTCATATCTTTA--TGTCAGAATTTTCACCTATTTGTATCTATTTAGTGATC

AGTCCGCTAGTTTCTTTGATCCCACTCGGTGTTCCTTTTCCATTTGCTTCCAATAGTTCGACCTATCCAG

AAAAATTGTCGGCCTACGAATGTGGTTTCGATCCTTCCGGTGATGCCAGAAGTCGTTTTGATATACGATT

TTATCTTGTTTCAATTTTATTTATTATCCCTGATCCGGAAGTAACCTTTTCCTTTCCTTGGGCAGTACCT

CCCAACAAGATTGATCCGTTTGGATCTTGGTCCATGATGGCCTTTTTATTGATTTTGACGATTGGATCTC

TCTATGAATGGAAAAGGGGTGCTTCGGATCGGGAATAA-AAAGTGTTTCTTACGATTACGCCCAACAGCC

CACTTGAGCAATTTGCCATTCTCCCATTGATTCCTATGAATATAGGAAACTTGTATTTCTCATTCACAAA

TCCATCTTTGTTTATGCTGCTAACTCTCAGTTTGGTCCTACTTCTGGTTCATTTTGTTACTAAAAACGGA

GGAGGAAACTCAGTACCAAATGCTTGGCAATCCTTGGTAGAGCTTATTTATGATTTCGTGCCGAACCCGG

TAAACGAACAAATAGGTGGTCTTTCCGGAAATGTTAAACAAAAGTTTTCCCCTCGCATCTCGGTTACTTT

TACTTTTTCGTTATTTCGTAATCCCCAGGGTATGATACCTTATAGCTTCACAGTTACAAGTCATTTTCTC

ATTACTTTGGGTCTCTCATTTTCTCTTTTTATTGGCATTACTATAGTGGGATTTCAAAGAAATGGGCTTC

ATTTTTTAAGCTTATCATTACCCGCTGGAGTCCCACTGCCGTTAGCACCTTTTTTAGTACTCCTTGAGCT

AATCCCTCATTGTTTTCGCGCATTAAGCCCAGGAATACGTTTATTTGCTAATATGATGGCCGGTCATAGT

TCAGTAAAGATTTTAAGTGGGTCCGCTTGGACTATGCTATGTATGAATGATCTTTTATATTTCATAGGAG

ATCTTGGTCCTTTATTTATAGTTCTTGCATTAACCGGTCCGGAATTAGGTGTAGCTATATCACAAGCTCA

TGTTTCTACGATCTCAATCTGTATTTACTTGAATGATGCTACAAATCTCCATCAAAG-------------

----------------ATATGTGGGCACCTGATATCTATGAGGGTTCACCCACCCCGGTTACAGCATTCC

TTTCTATTGCGCCTAAAATATCTATTTCTGCTAATATTTCACGTGTTTCTATTTATGGTTCCTATGGAGC

TACATTGCAACAAATCTTCTTTTTCTGCAGCATTGCTTCTATGATCTTAGGAGCACTGGCCGCCATGGCC

CAAACGAAAGTCAAAAGACTTCTAGCTCATAGTTCAATTGGACATGTAGGTTATATTCGTACTGGTTTCT

CATGTGGAACCATAGAAGGAATTCAATCACTACTAATTGGTATCTTTATTTATGCATCAATGACGATAGA

TGCATTCGCCATAGTTTCAGCATTACGGCAAACCCGTGTCAAATATATAGCGGATTTGGGCGCTCTAGCC

AAAACGAATCCTATTTCGGCTATTACCTTCTCCATTACTATGTTCTCATACGCAGGAATACCCCCGTTAG

CCGGCTTTTGTAGCAAATTCTATTTGTTCTTCGCCGCTTTGGGTTGTGGGGCTTACTTCCTAGCCCCAGT

GGGAGTAGTGACTAGCGTTATAGGTCGT--GTTCGATAGCCCGACCGTAGTGATGTTAATTGTGGTTACA

TCCATAAGTAGCTTGGTCCATCTTTATTCCATTTCATATATGTCTGAGGATCCGCATAGCCCTCGATTTA

TGTGTTATTTATCCATTCCTACTTTTTTTATGCCAATGTTGGTGACTGGAGATAACTCTCTTCAATTATT

CCTGGGATGGGAGGGAGTAGGTCTTGCTTCATATTTGTTAATTCATTTCTGGTTTACACGACTTCAGGCA

GATAAAGCAGCTATAAAAGCTATGCCTGTCAATCGAGTAGGTGATTTTGGATTAGCTCCTGGGATTTCGG

GTTGTTTTACTCTCTTTCAAACAGTAGACTTTTCAACCATTTTTGCTTGTGCTAGTGCCCCCAGAAATTC

TTGGATTTCTCGCAATATGAGATTGAATGCCATAACTCTTATTTGTATTTTACTTCTTATTGGTGCTGTT

GGGAAATCTGCACAGATAGGATCGCATACTTGGTCACCCGATGCTATGGAGGGTCCCACTCCAGTATCCG

CTTTGATTCATGCAGCTACTATGGTAACAGCTGGCGTTTTCATGATAGCAAGGTGCTCCCCTTTATTTGA

ATACCCACCTACGGCTTTGATTGTTATTACTTTTGCAGGAGCTACGACGTCATTCCTTGCGGCAACCACT

GGAATATTACAGAACGATCTAAAGAGGGTCATAGCTTATTCAACTTGCAGTCAATTAGGCTATATGATCT

TTGCTTGCGGCATCTCTAACTATTCGGTTAGCGTCTTTCACTTAATGAATCACGCGTTTTTCAAAGCATT

ACTCTTCCTGAGTGCAGGTTCGGTGATTCATGCCATGTCGGATGAGCAAGATATGCGGAAGATGGGGGGG

CTTGCCTCCTCGTTCCCTTTTACCTATGCCATGATGCTCATGGGCAGCTTATCTCTAATTGGATTTCCTT

TTCTAACTGGATTTTATTCCAAAGATGTGATCTTAGAGCTCGCTTACACTAAGTATACCATCAGTGGGAA

CTTTGCTTTCTGGTTGGGAAGTGTCTCTGTCCTTTTCACTTCTTATTACTCTTTTCGTTCACTTTTTCTA

ACATTTCTAGTACCAACTAATTCATTCGGGCGAGACATCTTACGATGTCATGATGCGCCCATTCCTATGG

CCATTCCTTTAATACTTCTGGCTCTCGGGAGTCTCTTTGTAGGATACTTGGCCAAACTAACACAAAGAAG

ATACAGTTCACTCAACGATTGCCTTTGGGTTCCGAACTCCATATGGGGAAGGAGCGTTGTTGTTTGCGAG

GTCTCGATCATTTACATGGACCCACTTTTCATTCCATTTGTGGGAATTTGATGATCTATAAACCGTCCTT

AACGAACGATCGGCTCATGTT------TGAGCATGATGAATCACTTCGTGCCGACCTGTTGCCAATAAAC

TTTCCGGCCTCATATGAGAATGGAAAACTGGAGCATTTTCTGCATCGGTGGATGAAGAATCGCGAACATA

ATAATTTCTGGTTGACCATGTTCCCAGAAAAAAGATACTTTCGAGAAACGACGAGTACGACTGAAGTGGC

TATACATACAAATCCATTTACGGATCTATATGCTTCGATTGGAACTGGAAGTTCCAGAACAGGCGGCTGG

TATACCACCATAATGAAACTGCCTTTTCTTTTTTTTATTTGGATAGGATTTATGTTGGCTTCGTTGGGAG

GCTCGCGTAGTTTGTTACGTCAGCTCCAAAAGGATAAGTTGCGTTGGAATCGAGAAAGTTCCGTGG----

AGTTCATAATTGCATA-CCAATTTTTGGGCCAATTCCCCCTTCGTACTACCAAAAAATGAGATTCTTGCC

GAATCCGAGTTTGCTGCTCCAACCATTACCAAACTAATACCTATTCCGTTTAGTACTTCAGGTGCTTCTG

TTGCGTATAATGTAAATCCCGTAGCGGATCAATTCC---------------AACGAGCCTTTCAAACTAG

TACTTTTTGTAATCGACTCTATAGCTTCTTCAATAAACGCTGGTTCTTCGATCAAGTTTTGAATGACTTT

CTAGTCAGATCGTTCCTGCGTTTCGGATATGAAGTCTCATTCGAAGCTTTAGACAAAGGTGCTATTGAGA

TATTGGGCCCTTATGGTATCTCGTACACATTCCGACGATTGGCCGAGCGAATAAGTAAACTTCAAAGTGG

ATTTGT-TTGTTCCATGATCTATGGGTCTACTGGAGCTACCCACTTCGATCAATTAGCCAAGATTTTGAC

CGGATACGAAATCACTGGTGCTCGATCTAGTGGTATTTTTATGGGGATTCTATCTATCGCTGTAGGATCC

CTATTCAAGATCACTGCAGTTCCTTTT--GGTCCATGCACATCGCTCTCTCCAGGAGGTTGGCCGCCTAT

CCTAGATCTTCCCATTTTCAAGAAGATCCCGGGCTCGATCCGGTTTAGTATCAAGGTGATTCTTTTTCTG

TTTCTATATATATGGGTCCGTGCAGCATTTCCACGATATCGTTATGATCAATTAATGGGACTTGGCCGGA

AAGTGTTCTTGCCTCTATCATTAGCTCGGGTAGTCGCCGTTTCTGGTGTTTTAGTCACCTTTCAATGGCT

CCCTTAAATGCCTCAACTGGATAAATTGACTTATTTCACACAATTCTTCTGGTCATGCCTTTTCCTCTTT

ACTTTCTATATTCCCATATGCAATGATGGAGATGGAGTACTTGGGATCAGCAGAATTCTAAAACTACGGA

ACCAACTGGTTTCACACCGGGAGAACAACATCCGGA------GCAACGACCCCAAGAGTTTGGAAGATAT

CTTGCTAAAAGGTTTTAGCACCGGTGTATCCTATATGTACTCCAGTTTATTCGAAGTATCCCAATGGTGT

AACGCCGTCGACTTATTGGGAAAAAGGAGGAAAATCACTTTGATCTCTTGTTTCGGAGAAATAAGTGGCT

CACGAGGAATGGAAAGAAACATATTCTATTTGATCTCGAAGTCCTCATATAGCACTTC------------

---TTCCAATCCTGAATGGGGGATCCCCTGTAGGAATGACATAATGCTAATCCATGTTCCACACGGCCAA

GGAAGCATCGTTTTTTAA-----

>Camellia sinensis NC043914.1

ATGATACTTTCTGTTTTGTCAAGCCCTGCTTTGGTCTCTGGTTTGATGGTTGTACGTGCTAAAAATCCGG

TACATTCCGTTTCGTTTCCCATCCCAGTCTTTCGCGACACTTCAGGTTTACTTCTTTTGTTAGGTCTCGA

CTTCTTCGCTATGATCTTCCCAGTAGTTCATATAGGAGCTATAGCCGTTTCATTCCTATTCGTTGTTATG

ATGTTCCATATTCAAATAGCGGAGATTCACGAAGAAGTATTGCGCTATTTACCAGTGAGTGGTATTATTG

GACTGATCTTTTGGTGGGAAATGTTCTTCATTTTAGATAATGAAAGCATTCCATTACTACCAACCCAAAG

AAATACGACCTCTCTGAGATATACGGTTTATGCCGGAAAGGTACGAAGTTGGACTAATTTGGAAACATTG

GGCAATTTACTTTATACCTACTATTCCGTCTGGTTTTTGGTTCCTAGTCTTATTTTATTAGTAGCCATGA

TTGGGGCTATAGTACTGACTATGCATAGGACTACTAA------GGTGAAAAGACAGGATGTATTCCGACG

AAATGCTATTGATTCTAGGAGGACTATAATGAGGAGGACGACAGAC---TGTCAATATATGAATTGTTTC

ATTATTCGTTATTTCCGGGTCTTTTCGTTGCATTCACTTACAACAAGAAACAACCACCAGTGTTTGGTGC

AGCACCTGCATTTTGGTGCATTCTTCTTTCTTTCCTTGGTCTTTCGTTCCGTCATATTCCTAATAACTTA

TCCAATTACAACGTATTAACTGCTAATGCACCTTTCTTTTATCAAATCTCAGGGACATGGTCTAATCATG

AGGGTAGTATTTTATCATGGTGTCGGATCCTAAGTTTTTATGGATTTCTTCTTTGTTACCGGGGTCGACC

CCAAAGCCATAATGTCTCAAAACGAGGAGGCCATAGAGAAACTCTTTTTTATTCCTTTGTCTTGAACTTC

GTGAAGAACTCCATTCTACCTCTCCCTCGTTACGAACAAAAAAGTGGGGCT------GCGCCCCAGTTGT

ACACTCCCTTCGTTCTACGAA---CCCTTGTTGATTCTGAACTTCGTTCGCGAAGGAACCGGACTTTTGA

CGGGCCAGCCCTTTTTTATGCGCCGCTTTACCCTGAAAGGAAAATGAGCTTTGCTCCTCTGGGCGCTAGG

CGCTCCCGTGGTTCGCGAGAAGGAAAAAGGACTCATCCTTTGTTGCATCTGGCACGAGATGATAAAGAGA

GAGCTTCGTCTATCGATGAACAGCGGATTGACGGAGCTCTTGGCATTGCTTTGTTTTTCTCTCCTTTCCT

ATCAGCGAGTTCCGATCCTTTTGTTCGAAATTTCTTCGTTCGTACCGAACCGCTTGCAGAATCAAATCCT

GTTCCACAAGATCCTATATCAGCTATACATCCTCCTTGCATTTATGCCGGAGACGTCGCCAGTGCTATGG

GCTTTGGCTTATGTAGATCAAAAATGATGAATGGGATTGCGGCACTCCACTCGCCGCCAATGCGGAAGGA

TGCCGCCGAAAAGAATGGAACGCTGTTTCGCTCTGCTGGATGCGTCGGATCCCGTATAACAAGCGAGCTC

TTTACCCTCAAATTCAAACATGTGGGCGCAAAATGCTATCCTGCTCTATTCTTACGTAGCAATAGAAGCC

TGCTT---ATGCTGCTTCGGCGGCGCTTTTTCGCCTTCTCTTCGCTCTGGACAGGAGCGCTAGTGGACGC

GGGGAGGGAGCA------GGCGAAGCCTGTCG------TTCGTAATGGAAAGAAAGATACCACTACTTCG

CCTCTTTGTTGGACCGCCGGCGCGAACACAGTGGTCTCTGACCAGGACCAGGAACCAATTCGAATTTGGA

TCTTGACATGTCGGTGGTTTTTAACCGTAGGCATCTTGCCAGGAAGTTGGTGGGCTCATCATGAATTAGG

TCGGGGTGGCTGGTGGTTTCGGGATCCCGTAGAAAATGCTTCTTTTATGCCTCGGGTATTAGCCACAGCT

CGTATTCATTCAGTAATTCTACCCCTTCTTCATTCTTGGACCTCGCTTCTTAATATTGTGACTCTTCCAT

GCTGTGTCTCAGGAACCTCTTCAATACGGTCCGGATTGCTAGCTCCCGTTCATAGTTTTGCTACAGATGA

TACACGAGGAATCTTTTTATGGCGGTTCTTCCTTCTAATGACCGGCATATCTATGATTCTTTTCTCCCAG

ATGAAGCAGCAGGCATCGGTCCGTAGAACCTATAAAAAAGAGATGGTTGTGGCGCGAAGTACTCTTGTGC

ACCTACGT--ATGATTGTTCTAGAATGGCGATTCCTCACAATTGCTCCTTGTGATGCAGCAGAACCATGG

CAATTAGGATCTCAAGACGCAGCAACACCTATGATGCAAGGAATAATAGACTTACATCACGATATCTTTT

TCTTCCTCATTCTGATTTTGGTTTTCGTATCACGGATCTTGGTTCGCGCTTTATGGCATTTCCACTATAA

AAAAAATCCAATCCCGCAAAGGATTGTTCATGGAACTACTATCGAGATTCTTCGGACCATATTTCCTAGT

ATCATCCCGATGTTCATTGCTATACCATCATTTGCTCTGTTATACTCAATGGATGAGGTAGTAGTAGATC

CAGCCATTACTATCAAAGCTATTGGACATCAATGGTATCGGACTTATGAGTATTCGGACTATAACAGTTC

CGATGAACAGTCACTCACTTTTGACAGTTATACGATTCCAGAAGATGATCCAGAATTGGGTCAATCACGT

TTATTAGAAGTGGACAATAGAGTGGTTGTACCAGCCAAAACTCATCTACGTATTATTGTAACACCTGCTG

ATGTACCTCATAGTTGGGCTGTACCTTCCTCAGGTGTCAAATGTGATGCTGTACCTGGTCGTTTAAATCA

GACCTCTATTTCGGTACAACGAGAAGGAGTTTACTATGGTCAGTGCAGTGAGATTTGTGGAACTAATCAT

GCCTTTACGCCTATCGTCGTAGAAGCTGTTTCTAGGAAAGATTATGGTTCTCGGGTATCTAATCAATTAA

TCCCCCAAAC-------------ATGAGACGACTCTTTCTTGAACTATATCATAAACAGATCTTCCCCTC

CACACCAATCACGAGTTTTTCTCCATTCCTCTCGTATATCGTCGTAACGCCCTTAATGCTAGGTTTTGAA

AAAGACTTTTCATGTCATTCCCATTTAGGTCCGATTCGGATCCCTCCGTTGTTTCCTTTTCCTTCCGCAC

CTTTTCCTCGAAATGAGAAAGAAGATGGTACACTTGAATTGTATTATTTAAGTGCTTATTGCTTGCCAAA

GATCCTACTTCTACAATTGGTAGGTCACCGGGTTATTCAAATAAGTCGTGTTTTCCGTGGTTTTCCCATG

TTACAACTTCCGTACCAATTCGGTCGATCCGGAATGGATCGGTTAAACATTCTATTAGGGAGCCTGGTCT

TGACTCTTCTGTGTGGTATTCATTCTCGTTCGGCTCTTGGAATCACATCCAGCAGTGGTTGGAACAGCTC

GCAAAATCCAACCACTTCACCTACTTCATTGCCCCCAACCGTTTCTCGTACCTCTATTGAAACAGAATGG

TTTCATGTTCTTTCATCGATTGGTTATTCCTCTCCGTTCGTATCTCTTTTTCCAATTTCGGTCTCGATTA

GTTTACAAGATTG-ATGTCCGTTTCGTTATTACAACCTTC-----TTTTTTGATGTCAAAGACCAGAAGC

TACGCGCAAATTCTCATTGGATCTCGGTTGTTCTTAACAGCGATGGCTATTCATTTAAGTCTTCGGGTAG

CACCACTAGATCTTCAACAAGGTGGAAATTCTCGTATTCCGTATGTACATGTTCCTGCGGCTCGGATGAG

TATTCTTGTTTATATCGCTACGGCTATAAACACTTTCTTGTTCCTATTAACAAAACATCCCCTTTTTCTT

CGCTCTTCCGGAACCGGTACAGAAATGGGTGCATTTTCTACGTTGTTTACCTTAGTTACTGGGGGGTTTC

GGGGAAGACCTATGTGGGGCACCTTTTGGGTGTGGGATGCTCGTTTAACCTCTGTATTCATCTCGTTCCT

TATTTACCTGGGTGCACTGCGTTTTCAAAAGCTTCCTGTCGAACCGGCTCCTATTTCAATCCGTGCTGGA

CCGATCGATATACCAATAATAAAGTCTTCAGTCAACTGGTGGAATACATCGCATCAACCTGGGAGCATTA

GCCGATCTGGTACATCAATACATGTTCCTATGCCCATTCCAATCTTGTCTAACTTTGCTAACTCCCCCTT

CTCAACCCGTATCTTGTTCGTTCTGGAAACACGTCTTCCTATTCCATCTTTTCTCGAATCTCCTTTAACG

GAAGAAATAGAAGCTCGAGAAGGAATAC-TGCAGGCTAGAAAGATGCTATTTGCTGCTATTCTATCTATT

TGTGCATCAAGTTCGAAGAAGATCTCAATCTATAATGAAGAAATGATAGTAGCTCGTTGTTTTATAGGCT

TTATCATATTCAGTCGGAAGAGTTTAGGTAAGACTTTCAAAGTGACTCTCGACGGGAGAATCCAGGCTAT

TCAGGAAGAATCGCAGCAATTCCCCAATCCTAACGAAGTAGTTCCTCCGGAATCCAATGAACAACAACGA

TTACTTAGGATCAGCTTGCGAATTTGTGGCACCGTAGTAGAATCATTACCAATGGCACGCTGTGCGCCTA

AGTGCGAAAAGACAGTGCAAGCTTTGTTATGCCGAAACCTAAATGTTAAGTCAGCAACACTTCCAAATGC

CACTTCTTCCCGTCGCATCCGTCTTCAGGACGATCTAGTCACAGGTTTTCACTTCTCAGTGAGTGAAAGA

TTTGTCCCCGGGTGTACGTTGAAAGCTTCTATAGTAGAACTCATTCGAGAGGGCTTGGCGGTCTTAAGAA

TGGTTCGGGTGGGGGGTTTCTCT-ATGAAAGAGGCGATCAGAATGGTACTCGAATCCATTTACGATCCCG

AGTTTCCAGACACATCGCACTTCCGCTCGGGTCGAGGCTGCCACGCGGCCCTAAGACGGATCAAAGAAGA

GTGGGGAACCTCTCGCTGGTTTTTGGAATTCGACATCAGGAAGTGTTTTCACACCATCGACCGACATCGA

CTCATCTCAATCTTTAAGGAAGAGATCGACGATCCCAAGTTCTTTTACTCCATTCAGAAAGTCTTTTCCG

CCGGACGACTCGTAGGAGGTGAGAAGGGCCCTTACTCCGTTCCACACAGTGTACTACTATCGGCCCTACC

AGGCAACATCTACCTACACAAGCTCGATCAGGAGATAGGGAGGATCCGACAGAAGTACGAAATTCCGATT

GTTCAGAGAATCAGATCGGTTCTATTAAGGACAGGTCGTATTGATGACCAAGAAAACTCTGGAGAAGAAG

C--TGGAAATCTCTCCCAGAGCTGCGGAACTAACGACTCTATTAGAAAGTAGAATTACCAACTTTTACAC

GAATTTTCAAGTGGATGAGATCGGTCGAGTGGTCTCAGTTGGAGATGGGATTGCACGTGTTTATGGATTG

AACGAGATTCAAGCCGGGGAAATGGTTGAATTTGCCAGCGGTGTGAAAGGAATAGCGTTGAATCTTGAGA

ATGAGAATGTAGGGATTGTTGTCTTTGGTAGTGATACCGCTATTAAAGAAGGAGATCTTGTCAAGCGCAC

TGGATCTATTGTGGATGTTCCTGCGGGAAAGGCTATGCTAGGGCGTGTGGTCGACGCGTTGGGAGTACCT

ATTGATGGAAGAGGGTCTCTAAGCGATCACGAGCGAAGACGTGTCGAAGTGAAAGCCCCTGGGATTATTG

AACGTAAATCTGTGCACGAGCCTATGCAAACAGGGTTAAAGGCGGTAGATAGCCTCGTTCCTATAGGCCG

TGGTCAACGAGAACTTATAATCGGGGATCGACAAACTGGAAAAACAGCTATTGCTATCGATACCATATTA

AACCAAAAGCAAATGAACTCAAGGGGCACCTCTGAGAGTGAGACATTGTATTGTGTCTATGTAGCGATTG

GACAGAAACGCTCAACTGTGGCACAATTAGTTCAAATTCTTTCAGAAGCGAATGCTTTGGAATATTCCAT

TCTTGTAGCAGCCACCGCTTCGGATCCTGCTCCTCTGCAATTTCTGGCCCCATATTCTGGGTGTGCCATG

GGGGAATATTTCCGCGATAATGGAATGCACGCATTAATAATATATGATGATCTTAGTAAACAGGCGGTGG

CATATCGACAAATGTCATTATTGTTACGCCGACCACCAGGCCGTGAGGCTTTCCCAGGGGATGTTTTCTA

TTTACATTCCCGTCTCTTAGAAAGAGCCGCTAAACGATCGGACCAGACAGGCGCAGGTAGCTTGACCGCC

TTACCCGTCATTGAAACACAAGCTGGAGACGTATCGGCCTATATTCCCACCAATGTGATCCCCATTACTG

ATGGACAAATCTGTTCGGAAACAGAGCTCTTTTATCGCGGAATTAGACCTGCTATTAACGTCGGCTTATC

TGTCAGTCGCGTCGGGTCTGCCGCTCAGTTGAAAGCTATGAAACAAGTCTGCGGTAGTTCAAAACTGGAA

TTGGCACAATATCGCGAAGTGGCCGCCCTTGCTCAATTTGGGTCAGACCTTGATGCTGCGACTCAGGCAT

TACTCAATAGAGGTGCAAGGCTTACAGAAGTCCCGAAACAACCACAATATGCACCACTTCCAATTGAAAA

ACAAATTCTAGTCATTTATGCAGCTGTCAATGGATTCTGTGATCGAATGCCATTAGAAAAAATTTCTCAA

TATGAGAGAGCCATTCCAAGTAGTGTAAAACCAGAATTACTACAATCTCTTTTAGAAAAAGGTGGGTTAA

CTAACGAAAGAAAGATGGAACCAGATGCATTCTTAAGAGAAAGTGCTTTGCCTTACCTATGATGCAAGAA

AGAAT--GCATCCAACGCAAAGCGGCCTTTCATTCCCTTGTTTCGTCGTGGCACACCCCCCCCACAAGCA

CCCCCCGGCTCAGGGGGGACCAGAAAACGCCTTTCGTTTTCCCCCCTTCGTCG------GCCGCCTTCCT

TAACAAGCCCTCGAGCCTCCTTTGCGCCGCCTTCCTCATAGAAGCCGCTGGGTTGACCCCGAAGGCCGAA

TTCTATGGTAGAGAAGGCTGTAATAATAATTGGGCCATGAGAGACTTTATTAAGTATTGCAAAAGAAAGG

GCCTGCTGATAGAGCTGGGCGGGGAGGCGATACTAGTTATCAGGTCAGAGAGGCGCCTGGCCCGTAAGCT

GGCCCCCTTAAAAACCCATTACTTAATAAGGATTTGTTACGCGCGATATGCCGACGACTCACTACTGGGA

ATCGTGGGTGCCGTAGAGCTTCTCATAGAAATACAAAAACGTATCGCCCACTTCCTACAATCCGGCCTGA

ACCTTTGGGTAAGCTCTGCAGGATCAACAACAATAACTGCACGGAGTACGGTAGAATTCCTCGGTACGAT

CATTCGGGAAGTCCCTCCGAGGACGACTCCCATACAATTCTTGCGAGAGCTGGAGAAGCGTCTACGGGTA

AAGCACCGTATCCATATAACTGCTTGCCACCTACGCTCCGCCATCCATTCCAAGTTTAGGAACCTAGGGA

ATAGTATCCCGATCAAACAGCTGACGAAGGGGATGAGCGGAACAGGGAGTCTACTGGACGCGGTTCAACT

AGCGGAGACTCTTGGAACAGCTGGAGTAAGAAGTCCCCAAGTGAGCGTATTATGGGGGACCGTCAAGCAC

ATCCGGCAGGGATCAAGGGGGATCTCGTTGTTGCATAGCTCAGGTCGGAGCAAGGTGCCATCGGAC----

--CGGGCAGTCTCACGATCGGGCACTCATGCCCGGAAGTTGTC---ATTGTATACTCCCGCGGGTCGGAA

GGCGGCGGGGGAAGGAGGGGGACACTGGGCGAGATCTATCAGCAGCGAATTCCCCATACAAATAGAGGCG

CCTATCAAAAAGATACTCCGAAGGCTTCGGGATCGAGGTATCATTAGCCGAAGAAGACCCTGGCCAATCC

ACGTGGCCTGCTTGACGAACGTCAGCGACGGAGACATCGTAAATTGGTCCGCGGGCATTGCGATAAGTCC

TCTGTCCTACTACAGGTGCCGCGACAACCTTTACCAAGTCCGAACGATTGTCGACCACCAGATCCGCTGG

TCTGCAATATTCACCCCGGCCCACAAGCACAAATCCTCGGCGCGGAATATAATCCCAAAGTACTCCAAAG

ACTCAAATATAGTAAATCAAGAAGGTGGTAAGACCCTTGCAGAGTTCCCCAACAGCATAGAGCTTGGGAA

GCTCGGACCCGGTCAAGATCC-GAACAACAAGGAGCACTCAACTACT-------------------TGGT

CCAACTACATAACTTTTTCTTTTTCATTACTTCCATGGTCGTGCCTCGTGGCACGGCAGCACCCGTACTA

TTGAAATGGTTCGTCAGTAGAGATGTTCCCACAGGTGCCCCTTCTTCCAATGGTACTATAATTCCTATTC

CTATCCCTTCATTCCCTCTTTTGGTCTATCTACATTCCAGGAAATTCATACGCTCCACGGACGGAGCAAA

AAGTGGAGTCTTGGTCAGAGCAAGCCGCCCTATTCTAT------TACCAGACATAATTGGGAGAAGCTCA

TCCGAAACTAGAGCAAGAAACGCCTCATTTCGTTTTGTTCCTGTTCTTCATTTCCTTCTTCTTCAATCCA

A------GGGGGACTTCTCATATTTAGAATCTTTCTGCGGTGTGCTCCGTTTACTATTCTTTCGTACTTT

CTTCTTTTTACCACGCGATAGGTCAGCGAAGCGTGAGCGGGCGCGGAGAAGGAAAGGCCAAACACTTCGG

CCTAAC------GGGAATGAGCAACGACGAAATGACAAGATGAGGTGCCCCGGGCATCCCCATT------

TAGA---AAGAAGGATCGAAGGTTTTGGGCCTGTAGCTTTCCCCGTCCCCCCTTCGTCGGGTGGTCCTTG

TGTGGGGGGTGCGCCACCAGAAATCGGGCTTGAAGCTCTCGCCTTACCAACGAGCCGACAGCTGATGGCT

GTTGGTCACGACTACTACCAAAAAGCTCCAATGAAGATGAATATTTCACATGGAGGAGTGTGCATCTGTA

TGTTGGGTGTTCTTCTGT---TACATAGCTGTTCCAGCTGAAATACTTGGAATAATTCTACCACTTCTAC

TGGGAGTAGCCTTTTTAGTGCTAGCTGAACGTAAAGTAATGGCTTTTGTGCAACGTCGAAAGGGTCCTGA

TGTAGTGGGATCGTTTGGATTGTTACAACCTCTAGCAGATGGTTTGAAATTGATTCTAAAAGAACCTATT

TCACCAAGTAGTGCTAATTTATCCCTTTTTAGAATGGCTCCAGTGGCTACATTTATGTTAAGTCTGGTCG

CTCGGGCCGTTGTACCTTTTGATTATGGTATGGTATTGTCAGATCCGAACATAGGGCTACTTTATTTGTT

TGCCATATCTTCGCTAGGTGTTTATGGAATTATTATAGCAGGTCGGTCTAGTATTTATTATATACGCTTA

GCGAAAAGAATGTTTTTTGATACACCTAGGACATGGATTCTATATGAACCAATGGATCGTGACAAGTCGT

TACTACTAGCAATGACTTCCTCTTTCATTACTTCATTCTTTCCATATCCCTCTCCTTTGTTCTCAGTTAC

TCATCAAATGGCACTCAGTTCATATCTTTA-ATGTCAGAATTTTCACCTATTTGTATCTATTTAGTGATC

AGTCCGCTAGTTTCTTTGATCCCACTCGGTGTTCCTTTTCCATTTGCTTCCAATAGTTCGACCTATCCAG

AAAAATTGTCGGCCTACGAATGTGGTTTCGATCCTTCCGGTGATGCCAGAAGTCGTTTTGATATACGATT

TTATCTTGTTTCAATTTTATTTATTATCCCTGATCCGGAAGTAACCTTTTCCTTTCCTTGGGCAGTACCT

CCCAACAAGATTGATCCGTTTGGATCTTGGTCCATGATGGCCTTTTTATTGATTTTGACGATTGGATCTC

TCTATGAATGGAAAAGGGGTGCTTCGGATCGGGAATA-AAAAGTGTTTCTTACGATTACGCCCAACAGCC

CACTTGAGCAATTTGCCATTCTCCCATTGATTCCTATGAATATAGGAAACTTGTATTTCTCATTCACAAA

TCCATCTTTGTTTATGCTGCTAACTCTCAGTTTGGTCCTACTTCTGGTTCATTTTGTTACTAAAAACGGA

GGAGGAAACTCAGTACCAAATGCTTGGCAATCCTTGGTAGAGCTTATTTATGATTTCGTGCCGAACCCGG

TAAACGAACAAATAGGTGGTCTTTCCGGAAATGTTAAACAAAAGTTTTCCCCTCGCATCTCGGTTACTTT

TACTTTTTCGTTATTTCGTAATCCCCAGGGTATGATACCTTATAGCTTCACAGTTACAAGTCATTTTCTC

ATTACTTTGGGTCTCTCATTTTCTCTTTTTATTGGCATTACTATAGTGGGATTTCAAAGAAATGGGCTTC

ATTTTTTAAGCTTCTCATTACCCGCTGGAGTCCCACTGCCGTTAGCACCTTTTTTAGTACTCCTTGAGCT

AATCCCTCATTGTTTTCGCGCATTAAGCCCAGGAATACGTTTATTTGCTAATATGATGGCCGGTCATAGT

TCAGTAAAGATTTTAAGTGGGTCCGCTTGGACTATGCTATGTATGAATGATCTTTTATATTTCATAGGAG

ATCTTGGTCCTTTATTTATAGTTCTTGCATTAACCGGTCCGGAATTAGGTGTAGCTATATCACAAGCTCA

TGTTTCTACGATCTCAATCTGTATTTACTTGAATGATGCTACAAATCTCCATCAAA--------------

----------------ATATGTGGGCACCTGATATCTATGAGGGTTCACCCACCCCGGTTACAGCATTCC

TTTCTATTGCGCCTAAAATATCTATTTCTGCTAATATTTCACGTGTTTCTATTTATGGTTCCTATGGAGC

TACATTGCAACAAATCTTCTTTTTCTGCAGCATTGCTTCTATGATCTTAGGAGCACTGGCCGCCATGGCC

CAAACGAAAGTCAAAAGACTTCTAGCTCATAGTTCAATTGGACATGTAGGTTATATTCGTACTGGTTTCT

CATGTGGAACCATAGAAGGAATTCAATCACTACTAATTGGTATCTTTATTTATGCATCAATGACGATAGA

TGCATTCGCCATAGTTTCAGCATTACGGCAAACCCGTGTCAAATATATAGCGGATTTGGGCGCTCTAGCC

AAAACGAATCCTATTTCGGCTATTACCTTCTCCATTACTATGTTCTCATACGCAGGAATACCCCCGTTAG

CCGGCTTTTGTAGCAAATTCTATTTGTTCTTCGCCGCTTTGGGTTGTGGGGCTTACTTCCTAGCCCCAGT

GGGAGTAGTGACTAGCGTTATAGGTCGT--GTTCGATAGCCCGACCGTAGTGATGTTAATTGTGGTTACA

TCCATAAGTAGCTTGGTCCATCTTTATTCCATTTCATATATGTCTGAGGATCCGCATAGCCCTCGATTTA

TGTGTTATTTATCCATTCCTACTTTTTTTATGCCAATGTTGGTGACTGGAGATAACTCTCTTCAATTATT

CCTGGGATGGGAGGGAGTAGGTCTTGCTTCATATTTGTTAATTCATTTCTGGTTTACACGACTTCAGGCA

GATAAAGCAGCTATAAAAGCTATGCCTGTCAATCGAGTAGGTGATTTTGGATTAGCTCCTGGGATTTCGG

GTTGTTTTACTCTCTTTCAAACAGTAGACTTTTCAACCATTTTTGCTTGTGCTAGTGCCCCCAGAAATTC

TTGGATTTCTCGCAATATGAGATTGAATGCCATAACTCTTATTTGTATTTTACTTCTTATTGGTGCTGTT

GGGAAATCTGCACAGATAGGATCGCATACTTGGTCACCCGATGCTATGGAGGGTCCCACTCCAGTATCCG

CTTTGATTCATGCAGCTACTATGGTAACAGCTGGCGTTTTCATGATAGCAAGGTGCTCCCCTTTATTTGA

ATACCCACCTACGGCTTTGATTGTTATTACTTTTGCAGGAGCTACGACGTCATTCCTTGCGGCAACCACT

GGAATATTACAGAACGATCTAAAGAGGGTCATAGCTTATTCAACTTGCAGTCAATTAGGCTATATGATCT

TTGCTTGCGGCATCTCTAACTATTCGGTTAGCGTCTTTCACTTAATGAATCACGCGTTTTTCAAAGCATT

ACTCTTCCTGAGTGCAGGTTCGGTGATTCATGCCATGTCGGATGAGCAAGATATGCGGAAGATGGGGGGG

CTTGCCTCCTCGTTCCCTTTTACCTATGCCATGATGCTCATGGGCAGCTTATCTCTAATTGGATTTCCTT

TTCTAACTGGATTTTATTCCAAAGATGTGATCTTAGAGCTCGCTTACACTAAGTATACCATCAGTGGGAA

CTTTGCTTTCTGGTTGGGAAGTGTCTCTGTCCTTTTCACTTCTTATTACTCTTTTCGTTCACTTTTTCTA

ACATTTCTAGTACCAACTAATTCATTCGGGCGAGACATCTTACGATGTCATGATGCGCCCATTCCTATGG

CCATTCCTTTAATACTTCTGGCTCTCGGGAGTCTCTTTGTAGGATACTTGGCCAAA-TAACACAAAGAAG

ATACAGTTCACTCAACGATTGCCTTTGGGTTCCGAACTCCATATGGGGAAGGAGCGTTGTTGTTTGCGAG

GTCTCGATCATTTACATGGACCCACTTTTCATTCCATTTGTGGGAATTTGATGATCTATAAACCGTCCTT

AACGAACGATCGGCTCATGTT------TGAGCATGATGAATCACTTCGTGCCGACCTGTTGCCAATAAAC

TTTCCGGCCTCATATGAGAATGGAAAACTGGAGCATTTTCTGCATCG------GAAGAATCGCGAACATA

ATAATTTCTGGTTGACCATGTTCCCAGAAAAAAGATACTTTCGAGAAACGACGAGTACGACTGAAGTGGC

TATACATACAAATCCATTTACGGATCTATATGCTTCGATTGGAACTGGAAGTTCCAGAACAGGCGGCTGG

TATACCACCATAATGAAACTGCCTTTTCTTTTTTTTATTTGGATAGGATTTATGTTGGCTTCGTTGGGAG

GCTCGCGTAGTTTGTTACGTCAGCTCCAAAAGGATAAGTTGCGTTGGAATCGAGAAAGTTCCGTGG----

AGTTCATAATTGCATAA-CAATTTTTGGGCCAATTCCCCCTTCGTACTACCAAAAAATGAGATTCTTGCC

GAATCCGAGTTTGCTGCTCCAACCATTACCAAACTAATACCTATTCCGTTTAGTACTTCAGGTGCTTCTG

TTGCGTATAATGTAAATCCCGTAGCGGATCAATTCC---------------AACGAGCCTTTCAAACTAG

TACTTTTTGTAATCGACTCTATAGCTTCTTCAATAAACGCTGGTTCTTCGATCAAGTTTTGAATGACTTT

CTAGTCAGATCGTTCCTGCGTTTCGGATATGAAGTCTCATTCGAAGCTTTAGACAAAGGTGCTATTGAGA

TATTGGGCCCTTATGGTATCTCGTACACATTCCGACGATTGGCCGAGCGAATAAGTAAACTTCAAAGTGG

ATTTGTTTTGTTCCATGATCTATGGGTCTACTGGAGCTACCCACTTCGATCAATTAGCCAAGATTTTGAC

CGGATACGAAATCACTGGTGCTCGATCTAGTGGTATTTTTATGGGGATTCTATCTATCGCTGTAGGATCC

CTATTCAAGATCACTGCAGTTCCTTTT-TGGTCCATGCACATCGCTCTCTCCAGGAGGTTGGCCGCCTAT

CCTAGATCTTCCCATTTTCAAGAAGATCCCGGGCTCGATCCGGTTTAGTATCAAGGTGATTCTTTTTCTG

TTTCTATATATATGGGTCCGTGCAGCATTTCCACGATATCGTTATGATCAATTAATGGGACTTGGCCGGA

AAGTGTTCTTGCCTCTATCATTAGCTCGGGTAGTCGCCGTTTCTGGTGTTTTAGTCACCTTTCAATGGCT

CCCTTA-ATGCCTCAACTGGATAAATTCACTTATTTCACACAATTCTTCTGGTCATGCCTTTTCCTCTTT

ACTTTCTATAT-----------------------------------------------------------

----------------------------------------------------------------------

----------------------------------------------------------------------

----------------------------------------------------------------------

----------------------------------------------------------------------

----------------------------------------------------------------------

-----------------------

>Malania oleifera NC053625.1

ATGATACTTTCTGTTTCGTCGAGCCCTGCTTTGGTCTCTGGTTTGATGGTTGCACGTGCTAAAAATCCGG

TACATTCCGTTTCGTTTCCCATCCCAGTCTTTCGCGACACTTCAGGTTTACTTCTTTTGTTAGGTCTCGA

CTTCTTCGCTATGATCTTCCCAGTAGTTTATATAGGAGCTATAGCCGTTTCATTCCTATTCGTTGTTATG

ATGTTCCATATTCAAATAGCGGAGACTCACGAAGAAGTATTGCGCTATTTACCAGTGAGTGGTATTATTG

GACTGATCCTTTGGTGGGAAATGTTCTTCATTTTAGATAATGAAACCATTCCATTACTACCAACCCAAAG

AAATACGACCTCTCTGAGATATACGGTTTATGCCGGAAAGGTACGAAGTTGGACTAATTTGGAAACATTG

GGCAATTTACTTTATACCTACTATTCCGTCTGGTTTTTGGTTCCTAGTCTTATTTTATTAGTAGCCATGA

TTGGGGCTATAGTACTTACTATGCATAGGACTACTAA------GGTGAAAAGACAGGATGTATTCCGACG

AAATGCAATTGATTCTAGGAGGACTATAATGAGGAGGACGACAGAC---TGTCAATATATGAATTGTTTC

ATTATTCGTTATTTCCGGGTCTTTTCGTTGCATTCACTTACAACAAGAAACAACCACCAGCGTTTGGTGC

AGCACCTGCATTTTGGTGCATTCTTCTTTCTTTCCTTGGTCTTTCGTTCCGTCATATTCCTAATAACTTA

TCCAATTACAACGTATTAACCGCTAATGCACCTTTCTTTTATCAAATCTCAGGGACATGGTCTAATCATG

AGGGTAGTATTTTATCATGGTGTCGGATCCCAAGTTTTTATGGATTCCTTCTTTGTTACCGGGGTCGCCC

CAAAAGCCATAATGTCTCAAAACGAGAAGGCCATAGAGAAACTCTTTTTTATTCCTTTGTCTCGAACTTC

GTGAAGAACTCCATTCTATCTCTCCCTCGTTACGAACAAAAAAGTGGGGCT------GCGCCCCAGTTGT

ACACTCCCTTCGTTTTACGAA---CCCTTGTTGATTCTGAACTTCGTTCGCGAAGGAACCGGACTTTTGA

CGGGCCAGCCCTTTTTTATGCGCCGCTTTACCCTGAAAGGAAAATGCGCTTTGATCCTCTGGGCGCTAGG

CGCTCCCGTGGTTCGCGAGAAGGAAAAAGGACTCATCCTTTGTTGCATCTGGCACGAGATGATAAAGAGA

GAGCTTCGTCTATCGATGAACAGCGGATTGATGGAGCTCTTGGCATTGCTTTGTTTTTCTCTCCTTTCCT

ATCAGCGAGTTCCGATCCTTTTGTTCGAAATTTCTTCGTTCGTACCGAACCGCTTGCAGAATCAAATCCT

GTTCCACAAGATCCTATATCAGCTATACATCCTCCTTGCATTTATGCCGGAGACGTCGCCAGTGCTATGG

GCTTTGGCTTATGTAGATCAAAAATGATGAATGGGATTGTGGCACTCCACTCGCCGCCAATGCGGAAGGA

TGCCGCCGAAAAGAATGGAACGCTGCTTCGCTCTGCTGGATGCGTCGGATCCCTTATAACAAGCGAGCTC

TTTACCCTCAAATTCAAAGATGTGGGCGCAAAATGCTATCCTGCTCTATTGTTGCGTAGCAATAGAAGCC

TGCTC---ATGCTGCTTCGGCGGCGCTTTTTCGCCTTCTCTTCGCTCTGGACAGGAGTGCTAGTGGACAC

GGGGAGGGAGCA------GGCGAAGCGTGTCG------TTCGTAATGGAAAGAAAGATACCACTACTTCG

CCTCTTTGTTGGACCGCCGGCGCGAACACAGTGGTCTCTGACCAGGACCAGGAACCAATTCGAATTTGGA

TCTTGACATGTCGGTGGTTTTTAACCGTAGGCATCTTGCCAGGAAGTTGGTGGGCTCATCATGAATTAGG

TCGGGGTGGCTGGTGGTTTCGGGATCCCGTAGAAAATGCTTCTTTTATGCCTCGGGTATTAGCCACAGCT

CGTATTCATTCAGTAATTATACCCCTTCTTCATTCTTGGACCTCGCTTCTTAATATTGTGACTCTTCCAT

GCTGTGTCTCAGGAACCTCTTCAATACGGTCCGGATTGCTAGCTCCCGTTCATAGTTTTGCTACAGATGA

TACACGAGGAATCTTTTTATGGCGGTTCTTCCTTCTAATGACCGGCATATCTATGATTCTTTTCTCCCAG

ATGAAGCAGCAGGCATCGGTCCGTAAAACCTATAAAAAAGAGATGGTTGTGGCGCGAAGTACTCTTGTGC

ACCTACGT--ATGATTGTTCTAGAATGGCTATTCCTCACAATTGCTCCTTGTGATGCAGCGGAACCATGG

CAATTAGGATCTCAAGACGCAGCAACACCTATGATGCAAGGAATAATGGACTTACATCACGATATCTTTT

TCTTCCTCATTCTGATTTTTGTTTTCGTATCACGGATCTTGGTTCGCGCTTTATGGCATTTCAACTATAA

AAAAAATCCAATCCCGCAAAGGATTGTTCATGGAACTACTATCGAGATTCTTCGGACCATATTTCCTAGT

ATCATCCCGATGTTCATTGCTATACCATCATTTGCTCTGTTATACTCAATGGACGAGGTAGTAGTAGATC

CAGCCATTACTATCAAAGCTATTGGACATCAATGGTATCGGAG---------------------------

----------------------------------------------------------------------

----------------------------------------------------------------------

----------------------------------------------------------------------

----------------------------------------------------------------------

----------------------------------------------------------------------

-----------------------ATGAGACGACTCTTTCTTGAACTATATCATAAACAGATCTTCCCCTT

CACACCAATCACGAGTTTTTCTCCATTCCTCTCGTATATCGTCGTAACGCCCTTAATGCTAGGTTTTGAA

AAAGACTTTTCATGTCATTCCCATTTAGGTCCGATTCGGATCCCTCCGTTGTTTCCTTTTCCTCCCGCAC

CTTTTCCTCGAAATGAGAAAGAAGATGGTACACTCGAATTGTATTATTTAAGTGCTTATTGCTTGCCAAA

GATCCTACTTCTACAATTGGTAGGTTACCGGGTTATTCAAATAAGTCGTGTTCTCCGCGGTTTTCCCATG

TTACAACTTCCGTACCAATTTGGTCGATCCGGAATGGATCGGTTAAACATTCCATTAGGGAGCCTGGTCT

TGACTCTTCTGTGTGGTATTCATTCTCGTTCGGCTCTTGGAATCACATCCAGCAGTGGTTGGAACAGCTC

GCAAAATCCAACCACTTCACCTACTTCATTACCCCTAACCTTTTCTCGTACCTCTATTGAAACAGAATGG

TTTCATGTTCCTTCATCGATTGGTTATTCCTCTCCGTTCGTATCTCTTTCTCCAATTTCGGTCTCGATTA

GTTCACAAGATTG-ATGTCCATTTCGTTATTACAACCTTA-----TTTTTGGATGTCAAAGACCAGAAGC

TACGCGCAAATTCTCATTGGATCTCGGTTGTTCTTAACTGCGATGGCTATTCATTTTAGTCTTCGGGTAG

CACCACTAGATCTTCAACAAGGTGGAAATTCTCGTATTCCGTATGTACATGTTCCTGCGGCTCGGATGAG

TATTCTTGTTTATATCGCTACGGCTATAAACACTTATTTTTTCCTATTAACAAAACATCCCCTTTTTCTT

CGCTCTTCCGGAACCGGTACAGAAATGGGTGCTTTTTCTACGTTGTTTACCTTAGTTACTGGGGGGTTTC

GGGGAAGGCCTATGTGGGGCACCTTTTGGGTGTGGGATGCTCGTTTAACCTCTGTATTCATCTCGTTCCT

TATTTACCTGGGTGCACTGCGTTTTCAAAAGCTTCCTGTCGAACCGGCTCCTATTTCAATCCGTGCTGGA

CCGATCGATATACCAATAATCAAGTCTTCAGTCAACTGGTGGAATACATCGCATCAACCTGGGAGCATTA

GCCGATCTGGTACATCAATACATGTTCCTATGCCCATTCCAATCTTGTCCAACTTTGCTAACTCCCCCTT

CTCAACCCGTATCTTGTTCGTTCTGGAAACACGTCTTCCTATTCCATCTTTTCTCGAATCTCCTTTAACG

GAAGAAATAGAAGCTCGAGAAGGAATAC--GCAGGCTAGAAAGATGTTATTTGCTGCTATTCCATCTATT

TGTGCATCAAGTTCGAAGAAGATCTCAATCTATAATGAAGAAATGATAGTAGCTCGTTGTTTTATAGGCT

TTATCATATTTAGTCGGAAGAGTTTAGGTAAGACTTTCAAAGTGACTCTCGACGGGAGAATCCAGGCTAT

TCAGGAAGAATCGCAGCAATTCCCCAATCCTAACGAAGTAGTTCCTCCGGAATCCAATGAACAACAACGA

TTACTTAGGATCAGTTTGCGAATTTGTGGCACCGTAGTAGAATCATTACCAATGGCACGCTGTGCGCCTA

AGTGCGAAAAGACAGTGCAAGCTTTGTTATGCCGAAACCTAAATGTTAAGTCAGCAACACTTCCAAATGC

CACTTCTTCCCGTCGCATCCGTCTTCAGGACGATCTAGTCACAGGTTTTCACTTCTCAGTGAGTGAAAGA

TTTGTCCCCGGTTCTACGTTGAAAGCTTCTATAGTAGAACTCATTCGAGAGGGCTTGGTGGTCTTAAGAA

TGGTTCGGGTGGGGGGTTCTCTT--TGAAGGAGGCGATCAGAATGGTACCCGAATCCATTTACGATCCCG

AGTTTCCAGACACATCGCACTTCCGCTCGGGTCGAGGCTGCCACTCGGCCCTAAGACGGATCAAAGAAGA

GTGGGGAACCTCTCGCTGGTTTTTGGAATTCGACATCAGGAAGTGTTTTCACACCATCGACCGACATCGA

CTCATCCCAATCTTTAAGGAAGAGATCGACGATCCCAAGTGCTTTTACCCCATTCAGAAAGTCTTTTCCG

CCGGACGACTCGTAGGAGGTGAGAAGGGCCCTTATTCCGTCCCACACAGTGTACTACTATCGGCCCTACC

AGGCAACATCTACCTACACAAGCTCGATCAGGAGATAGGGAGGATCCGACAGAAGTACGAAATTCCGATT

GTTCAGAGAATTAAATCGGTTCTATTAAGGACAGGTCGTATTGATGACCAAGAAAACTCTGGAGAAGAAG

CAATGGAATTCTCTCCCAGAGCTGCGGAACTAACGACTCTATTAGAAAGTAGAATTACCAACTTTTACAC

GAATTTGAAAGTGGATGAGATCGGTCGAGTGGTCTCAGTTGGAGATGGGATTGCACGTGTTTATGGATTG

AACGAGATTCAAGCTGGGGAAATGGTTGAATTTGCCAGCGGTGTGAAAGGAATAGCGTTGAATCTTGAGA

ATGAGAATGTAGGGATTGTTGTCTTTGGTAGTGATACCGCTATTAAAGAAGGAGATCTTGTCAAGCGCAC

TGGATCTATTGTGGATGTTCCTGCGGGAAAGGCTATGCTAGGGCGTGTGGTCGACGCGTTGGGAGTACCT

ATTGATGGAAGAGGGGCTCTAAGCGATCACGAGCGAAGACGTGTCGAAGTGAAAGCCCCTGGGATTATTG

AACGTAAATCTGTGCACGAGCCTATGCAAACAGGGTTAAAAGCGGTAGATAGCCTCGTTCCTATAGGCCG

TGGTCAACGAGAACTTATAATCGGGGACCGACAAACTGGAAAAACGGCTATTGCTATCGATACCATATTA

AACCAAAAGCAAATGAACTCAAGGGCCACCTCTGAGAGTGAGACATTGTATTGTGTCTATGTAGCGATTG

GACAGAAAGGCTCAACTGTGGCACAATTAGTTCAAATTCTTTCAGAAGCGAATGCTTTGGAATATTCCAT

TCTTGTAGCAGCCACCGCTTCGGATCCTGCTCCTCTGCAATTTCTGGCCCCATATTCTGGGTGTGCCATG

GGGGAATATTTCCGCGATAATGGAATGCACGCATTAATAATCTATGATGATCTTAGTAAACAGGCCGTGG

CATATCGACAAATGTCATTATTGTTACGCCGACCACCGGGCCGTGAGGCTTTCCCAGGCGATGTTTTCTA

TTTACATTCCCGTCTCTTAGAAAGAGCCGCTAAACGATCGGACCAGACAGGCGCAGGTAGCTTGACCGCC

TTACCCGTCATTGAAACACAAGCTGGAGACGTATCGGCCTACATTCCCACCAATGTGATCCCCATTACTG

ATGGACAAATCTGTTTGGAAACAGAGCTCTTTTATCGCGGAATTAGACCTGCTATTAACGTCGGCTCATC

TGTCAGTCGCGTCGGGTCTGCCGCTCAGTTGAAAGCTATGAAACAAGTCCGCGGTAGTTCAAAACTAGAA

TTGGCACAATATCGCGAAGTGGCCGCCCTTGCTCAATTTGGGTCAGACCTTGATGCTGCGACTCAGGCAT

TACCCAATAGAGGTGCAAGGCTTACAGAAGTACCGAAACAACCACAATATGCACCACTTCCAATTGAAAA

ACAAATTATAGTAATTTATGCAGCTGTCAAGGGATTCTGTGATCGAATGCCACTAGACAGAATTTCTCAA

TATGAGAGAGCCATTCAAAGTAGTATCAAAGAAGAATTACTACAATCCCTTTTAGAAAAAGATGGGTTAA

CTAACGAAAGAAAGATGGAACCAGACGAATTCTTAAAAGAAAGCGCTTTGCCTTA---------------

--------CATCCAACGCAAAGCGGCCTTTCATTCCCTTGTTTCGTCGTGGCACA-CCCCCCCACAAGCA

CCCCCCGGCTCAGGGGGGACCAGAAAACTAAAGGTGTTTTCCCCCCTTCGTCGGCCCTTGCCGCCTTCCT

TAACAAGCCCTCGAGCCTCCTTTGCGCTGCCTTCCTCATAGAAGCCGCCGGGTTGACCCCGAAGGCCGAA

TTCTATGGTAGAGAACGCTGTTATAATAATAAGGCCATGAGAGACCTTTTTAA------CAAAAGAAAGG

GCCTGCTGATAGAGCTGGGCGGGGAGGCGATACTAGTTATCGGGTCAGAGAGAGGCCTGGCCCGTAAGCT

GGCCCCCTTAAAAACCCATTACTTAATAAGGATTTGTTACGCGCGATATGCCGACGACTCACTACTGGGA

ATCGTGGGTGCCGTAGAGCTTCTCATAGAAATACAAAAACGTATCGCCCACTTCCTACAATCCGGCCTGA

ACCTTTGGGTAGGCTCTGCAGGATCAACAACAATAGCTGCACGGAGTACGGTAGAATTCCTCGGTACGGT

CATTCGGGAAGTCCCTCCGAGGACGACTCCCATACAATTCTTGCGAGAGCTGGAGAAGCGTCTACGGGTA

AAGCACCGTATCCATATAACTGCTTGCCACCTACGCTCCGCCATCCATTCCAAGTTTAGGAACCTAGGTA

ATAGTATCCCGATCAAACAGCTGACGAAGGGGATGAGCGGAACAGGGAGTCTACTGGACGCGGTTCAACT

AGCGGAGACTCTTGGAACAGCTGGAGTAAGAAGTCCCCAAGTGAGCGTATTATGGGGGACCGTCAAGCAC

ATCCGGCAAGGATCAAGGGGGATCTCGTTGTTGCATAGCTCAGGTCGGAGCAACGTGCCATCGGACGTTC

AACAGGCAGTCTCACGATCGGGCATGAGTGTCCGGAAGTTGTC---ATTGTATACTCCCGCGGGTCGGGA

GGCGGCGGGGGAAGGAGGGGGACACTGGGCGAGATCTATCAGCTGCGAATTCCCCATACAGATAGAGGCG

CCTATCAAAAAGATACTCCGAAGGCTTCGGGATCGAGGTATCATTAGCCGAAGAAGACCCTGGCCAATCC

ACGTGGCCTGCTTGACGAACGTCAGCGACGGAGACATCGTAAATTGGTCCGCGGGCATCGCGATAAGTCC

TCTGTCCTACTACAGGTGCCGCGACAACCTTTACCAAGTCCGAACGATTGTCGACCACCAGATCCGCTGG

TCTGCAATATTCACCCCAGCCCACAAGCACAAATCCTCGGCGCGGAATATAATCCCAAAGTACTCCAAAG

ACTCAAATATAGTAAATCAAGAAGGTGGTAAGACCCTTGCAGAGTTCCCCAACAGCATAGAGCTTGGGAA

GCTCGGACCCGGTCAAGATCC-GAACAACAAGGAGCACTCAACTACTA-----------------ATGGT

CAAACTACATAACTTTTGCTTTTTCATTACTTCCATGGTCGTGCCTCGTGGCACGGCAGCACCCGTACTA

TTGAAATGGTTCGTCAGTAGAGATGTTCCCACAGGTGCCCCTTCTTCCAATGGTACTATAATTCCTATTC

CTATCCCTTCATTCCCTCTTTTGGTCTATATACATTCCAGGAAATTCATACGCTCCATGGACGGAGCAAA

AAGTGGAGTCTTGGTCAGAGCAAGCCGCCCTATTCTAT------TACCAGACATAATTGGGAGAAGCTCA

TCCGAAACTAGAGCTAGAAACGCCTCATTTCGTTTCGTTCCCGTTCTTCATTTCCTTCTTCTCGAATCCA

A------GGGGGACTTCTCATATTTAGAATCTTTCTGCGGTGTGCTCCGTTTACTATTCTTTCGTACTTT

CTTCTCTTTACCACGCGATAGGTCAGCGAAGCGGGAGCGGACGCGGAGAAGGAAACGCCAACCACTTCGG

CCTAAC------GGGAATGAGCAACGACGAAATGACAAGATGAGGTGCCTGGGGCACCCCCATT------

TAGA---AAGAAGGGTCGAAGGTTTTGGGCCTGTAGCTTTCCCCGTCCCCCCTTCGTCGGGTGGTGCTTG

TGTGGGGGGTGTGCCACCAGAAATCGGGCTTGAAGCTCTCTCCTTACCAAGGAGCCGACAGCTGATGGCT

GTTGGTCACGACTACTACCAAAAAGCTCCAATGAAGATGAATATTTCACATGGAGGAGTGTGCATCTGTA

TGTTGGGTGTTCTTCTG--CGTACATAGTTGTTCCAGCTGAAATACTTGGAATAATTCTACCACTTCTAC

TAGGAGTAGCCTTTTTAGTGCTAGCTGAACGTAAAGTAATGGCTTTTGTGCAACGTCGAAAGGGTCCTGA

TGTAGTGGGATCGTTCGGATTGTTACAACCTCTAGCAGATGGTTCGAAATTGATTCTAAAAGAACCTATT

TCACCAAGTAGTGCTAATTTCTCCCTTTTTAGAATGGCTCCAGTGGCTACATTTATGTTAAGTCTGGTCG

CTCGGGCCGTTGTACCTTTTGATTATGGTATGGTATTGTCAGATCCGAACATAGGGCTACTTTATTTGTT

TGCCATATCTTCGCTAGGTGTTTATGGAATTATTACAGCGGGTCGGTCTAGTATTTATTATATACGCTTA

GCGAAAAGAATGTTTTTTGATACACCTAGGACATGGATTCTATATGAACCAATGGATCGTGACAAGTCGT

TACTACTAGCAATGACTTCCTCTTTCATTACTTCATCCTTTCCATATCCCTCTCCCTTGTTCTCAGTTAC

TCATCAAATGGCACTCAGTTCATATCTTTA--TGTCAGAATTTGCACCTATTTGTATCTATTTAGTGATC

AGTCCGCTAGTTTCTTTGATCCCACTCGGTGTTCCTTTTCCATTTGCTTCCAATAGTTCGACCTATCCAG

AAAAATTGTCGGCCCACGAATGTGGTTCCGATCCTTCCGGTGATGCCAGAAGTCGTTTCGATATACGATT

TTATCTTGTTTCCATTTTATTTATTATCCCTGATCCGGAAGTAACCTTTTCCTTTCCTTGGGCAGTACCT

CCCAACAAGATTGATCTGTTTGGATCTTGGTCCATGATGGCCTTTTTATTGATTTTGACGATTGGATCTC

TCTATGAATGGAAAAGGGGTGCTTCGGATCGGGAGTAA---------------------------CAGCC

CACTTGAGCAATTTGCCATTCTCCCATTGATTCCTATGAATATAGGAAACTTGTATTTCTCATTCACAAA

TCCATCTTTGTTTATGCTGCTAACTCTCTGTTTGGTCCTACTTCTGGTTCATTTTGTTACTAAAAACGGA

GGAGGAAACTCAGTACCAAATGCTTGGCAATCCTTGGTAGAGCTTATTCATGATTTCGTGCCGAACCCGG

TAAACGAACAAATAGGTGGTCTTTCCGGAAATGTTAAACAAAAGTTTTCCCCTCGCATCTCGGTCACTTT

GACTTTTTCGTTATTTCGTAATCCCCAGGGTATGATACCTTATAGCTTCACAGTTACAAGTCATTTTCTC

ATTACTTTGGGTCTCTCTTTTTCTATTTTTATTGGTATTACTATAGTGGGATTTCAAAGAAATGGGCTTC

ATTTTTTAAGCTTCTCATTACCCGCAGGAGTCCCACTGCCGTTAGCACCTTTTTTAGTACTCCTTGAGCT

AATCCCTCATTGTTTTCGCGCATTAAGCTCAGGAATACGTTTATTTGCTAATATGATGGCCGGTCATAGT

TCAGTAAAGATTTTAAGTGGGTCCGCTTGGACTATGCTATGTATGAATGATCTTTTCTATTTCATAGGAG

ATCCTGGTCCTTTATTTATAGTTCTTGCATTAACCGGTCTGGAATTAGGTGTAGCTATATCACAAGCTCA

TGTTTCTACGATCTCAATCTGTATTTACTTGAATGATGCTACAAATCTCCATCAAA--------------

----------------ATATGTGGGCACCTGATATCTATGAGGGTTCACCCACCCCGGTTACAGCATTCC

TTTCTATTGCGCCTAAAATCTCTATTTCTGCTAATATTTCACGTGTTTCTATTTATGGTTCCTATGGAGC

TACATTGCAACAAATCTTCTTTTTCTGCAGCATTGCTTCTATGATCTTAGGAGCACTGGCCGCCATGGCC

CAAACGAAAGTCAAAAGACCTCTAGCTCATAGTTCAATTGGACATGTAGGTTATATTCGTACTGGTTTCT

CATGTGGAACCATAGAAGGAATTCAATCACTACTAATTGGTATCTTTATTTATGCATCAATGACGATAGA

TGCATTCGCCATAGTTTCAGCATTACGGCAAACCCGTGTCAAATATATAGCGGATTTGGGCGCTCTAGCC

AAAACGAATCCTATTTCGGCTATTACCTTCTCCATTACTATGTTCTCATACGCAGGAATACCCCCGTTAG

CCGGCTTTCGTAGCAAATTCTATTTGTTCTTCGCCGCTTTGGGTTGTGGGGCTTACTTCCTAGCCCCAGT

GGGAGTAGTGACTAGCGTTATAGGTCGT-TGTTCGATAGCCCGACCGTAGTGATGTTAATTGTGGTTACA

TCCATAAGTAGCTTGGTCCATCTTTATTCCATTTCATATATGTCTGAGGATCCGCATAGCCCTAGATTTT

TGTTTTATTTATCCATTCCTACTTTTTTTATGCCAATGTTGGTGACTGGAGATAACTCTCTTCAATTATT

CCTGGGATGGGAGGGAGTAGGTCTTGCTTCATATTTGTTAATTCATTTCTGGTTTACACGACTTCAGGCA

GATAAAGCAGCTACAAAAGCTATGCCTGTCAATCGAGTAGGTGATTTTGGATTAGCTCCTGGGATTTCGG

GTCGTTTTACTCTCTTTCAAACAGTAGACTCTTCAACCATTTTTGCTCGTGCTAGTGCCCCCAGAAATTC

TTGGATTTTTCGCAATATGAGATTGAATGCCATAACTCTTATTTGTATTTTACTTCTTATTGGTGCTGTT

GGGAAATCTGCACAGATAGGATCGCATACTTGGTCACCCGATGCTATGGAGGGTCCCACTCCTGTATCCG

CTTTGATTCATGCAGCTACTATGGTAACAGCTGGCGTTTTCATGATAGCAAGGTGCTCCCCTTTATTTGA

ATACCCACCTACGGCTTTGATTGTTATTACTTTTGCAGGAGCTACGACGTCATTCCTTGCGGCAACCACT

GGAATATTACAGAACGATCTAAAGAGGGTCATAGCTTATTCAACTTGCAGTCAATTAGGCTATATGATCT

TTGCTTGCGGCATCTCTAACTATTCGGTTAGCGTCTTTCACTTAATGAATCACGCGTTTTTCAAAGCATT

ACTATTCCTGAGTGCAGGTTCGGTGATTCATGCCATGTCGGATGAGCAAGATATGCGGAAGATGGGGGGG

CTTGCCTCCTCGTTCCCTTTTACCTATGCCATGATGCTCATGGGCAGCTTATCTCTAATTGGATTTCCTT

TTCTAACTGGATTTTATTCCAAAGATGTGATCTTAGAGCTAGCTTACACTAAGTATACCATCAGTGGGAA

CTTTGCTTTCTGGTTGGGAAGTGTCTCTGTCCTTTTCACTTCTTATTACTCTTTTCGTTCACTTTTTCTA

ACATTTCTAGTACCAACTAATTCATTCGGGCGAGACATCTTACGATGTCATGATGCGCCCATTCCTATGG

CCATTCCTTTAATACTTCTGGCTCTCGGGAGTCTCTTTGTAGGATACTTGGCCAA-CTAACACAAAGAAG

ATACAGTTCACTCAACGATTGCCTTTGGGTTCCGAACTCCATATGGGGAAGGAGCGTTGTTGTTTGCGAG

GTCTCGATCATTTACATGGACCCACTTATCATTCCATTTGTGGTAATTTGATGATCTATAAACCGTCCCT

AACGAACGATCGGCTCATGTT------TGAGCATGATGAATCACTTCGTGCCGACCTGTTGCCAATAAAC

TTTCCGGCCTCATATGAGAATGGAAAACTGGAGCATTTTCTGCATCGGTGGATGAAGAATAGCGAACATA

ATAATTTCTGGTTGACCATGTTCCCAGAAAAAAGAGACTTTCGAGAAACGACGAGCACGACTGAAGTGGC

TATACATACAAATCTATTTACGGATCTATATGCTCCGATTGGAACTTCCAGTTCCAGAACAGGCGGCTGG

TATACCACCATAATGAAACTGCCTTTTCTTTTTTTTATTCGGATAGGATTTATGTTGGCTTCGTCGGGAG

GCTCGCGTAGTTTGTTACGTCAGCTCCAAAAGGATAAGTTGCGTTGGAATCGAGAAAGTTCCGTGG----

AGTTCATAATTGCATA-CCAATTTTTGGGCCAATTCCCCCTTCGTACTACCAAAAAATGAGATTCTTGCC

GAATCCGAGTTTGCTGCTCCAACCATTACCAAACTAATACCTATTCTGTTTAGTACTTCAGGTGCTTCTG

TTGCGTATAATGTAAATCCCGTAGCGGATCAATTCC---------------AACGAGCCTTTCAAACTAG

TACTTTTTGTAATCGACTCTATAGCTTCTTCAATAAACGCTGGTTCTTCGATCAAGTTTTTAATGACTTT

CTAGTCAGATCGTTCCTGCGTTTCGGATATGAAGTATCATTCGAAGCTTTAGACAAAGGTGCTATTGAGA

TATTGGGCCCTTATGGTATCTCGTACACATTCCGACGATTGGCCGAGCGAATAAGTCAACTTCAAAGTGG

ATTTGT-TTGTTCCATGATCTATGGGTCTACTGGAGCTACCCACTTCGATCAATTAGCCAAGATTTTGAC

CGGATACGAAATCACTGGTGCTCGATCTAGTGGTATTTTTATGGGGATTCTATCTATCGCTGTAGGATCC

CTATTCAAGATCACTGCAGTTCCTTTT-TGGTCCATGCACATCGCTCTCTCCAGGAGGTTGGCCGCCTAT

CCTAGATCTTCCCATTTCCAAGAAGATCCCGGGCTCGATCCGGTTTAGTATCAAGGTGATTCTGTTTCTG

TTCCTATATATATGGGTCCGTGCAGCATTTCCACGATATCGTTATGATCAATTAATGGGACTTGGCCGGA

AAGTGTTCTTGCCTCTATCATTAGCTCGGGTAGTCCCCGTTTCTGGTGTTTTAGTCACCTTTCAATGGCT

CCCTTA--TGCCGCAACTGGATAAATTCACTTATTTCACACAATTCTTCTGGTCATGCCTTTTTTTCTTT

ACTTTCTATATTCCCATATGCAATGATGGAGATGGAGTACTGGGGATCAGCAGAATTCTAAAACTACGGA

ACCAACTGGTTTCACGTCGGGGGAACAACATCCGGA------GCAACGACCCCAACAGTTTGGAAGATAT

CTTGAGAAAAGGTTTTAGCACCGGTGTATCCTATATGTACTCTAGTTTATTCGAAGTATCCCAATGGTGT

AAGGCCGTCGACTTATTGGGAAAAAGGAGGAAAATTGCTTTGATCTCTTGTTTCGGAGAAATAAGTGGCT

CACGAGGAATGGAAAGAAACATATTCTATTTGATCTCGAAGTCCTCATATAGCACTTC------------

---TTCCAATCCTGGATGGGGGATCACTTGTAAGAATGACATAATGCTAATCCATGTTCTACACGGCCAA

G----------------------

>Actinidia chinensis NC065997.1

-TGATACTTTCTGTTTTGTCAAGCCCTGCTTTGGTCTCTGGTTTGATGGTTGTACGTGCTAAAAATCCGG

TACATTCCGTTTCGTTTCCCATCCCAGTCTTTCGCGACACTTCAGGTTTACTTCTTTTGTTAGGTCTCGA

CTTCTTCGCTATGATCTTCCCAGTAGTTCATATAGGAGCTATAGCCGTTTCATTCCTATTCGTTGTTATG

ATGTTCCATATTCAAATTGCGGAGATTCACGAAGAAGTATTGCGCTATTTACCAGTGAGTGGTATTATTG

GACTGATCTTTTGGTGGGAAATGTTCTTCATTTTAGATAATGAAAGCATTCCATTACTACCAACCCAAAG

AAATACGACCTCTCTGAGATATACGGTTTATGCCGTAAAGGTACGAAGTTGGACTAATTTGGAAACATTG

GGCAATTTACTTTATACCTACTATTCCGTCTGGTTTTTGGTTCCTAGTCTTATTTTATTAGTAGCCATGA

TTGGGGCTATAGTACTGACTATGCATAGGACTACTAA------GGTGAAAAGACAGGATGTATTCCGACG

AAATGCTATTGATTCTAGGAGGACTATAATGAGGAGGACGACAGACC----TCAATATATGAATTCCTTC

ATTATTCGTTATTTCCGGGTCTTTTAGTTGCATTCACTTACAACAAGAAAGAACCACCTGTGTTTGGTGC

AGCACCTGCATTTTGGTGTATTCTTCTTTCTTTCCTTGGTCTTTCGTTCCGTCATATTCCTAATAACTTA

TCCAATTACAACGTATTAACTGCTAATGCACCTTTCTTTTATCAAATCTCAGGGACATGGTCTAATCATG

AGGGTAGTATTTTATCATGGTGTCGGATCCTAAGTTTTTATGGATTTCTTCTTTGTTACCGGGGTCGACC

CCAAAGCCATAATGTCTCAAAACGAGTAGGCCATAGAGAAAGGCTTTTATATTCCTTTGTCTTGAACTTC

GTGAAGAACTCCATTCTATCTCTCCCTCGTTACGAACAAAAAAGTGGGGCT------GCGCCCCAGTTGT

ACACTCCCTTCGTTCTACGAA---CCCTTGTTGATTCTGAACTTCGTTCGCGAAGGAACCGGACTTTTGA

CGGGCCAGCTCTTTTTTATGCGCCGCTTTACCCTGAAAGGAAAATGAGCTTTGCTCCTCTGGGCGCTAGG

CGCTCCCGTGGTTCGCGAGAAGGAAAAAGGACTCATCCTTTGTTGCATCTGGCACTAGATAATAAAGAGA

GAGCTTCGTCTATCGATGAACAGCGGATTGACAGAGCTCTTGGCATTGCTTTGTTTTTCTCTCCTTTCCT

ATCAGCGAGTTCCGATCCTGTTGTTCGAAATTTATTCGTTCGTACCGAACCGCTTGCAGAATCAAATCCT

GTTCCACAAGATCCTATATCAGCTATACATCCTCCTTGCATTTATGCCGGAGACGTCGCCAGTGCTATGG

GCTTTGCCTTATGTAGATCAAAAATGATGAATGGGATTGTGGCACTCTACTCGCCGCCAATGCGGAAGGA

TGCCGCCGAAAAGAATGGAACGCTGTTTCGCTCTGCTGGATGCGTCGGATCCTGTATAACAAGCGAGCTC

TTTACCCTCAAATTCAAACATGTGGGCGCTAAATGCTATCCAGCTCTATTCTTACGTAGCAATAGAAGCC

CGCTTATGATGCTGCTTCGGCGGCGCTTTTTCGCCTTCTCTTCGCTCTGGACAGGAGCGCTAGTGGACGC

GGGGAGGGAGCA------GGCGAAGCCTGTCG------TTCGTAATGGAAAGAAAGATACCACTACAAAG

CCTCTTTGTTGGACCGCCGGCGCGAACACAGTGGTCTCTGACCAGGACCAGGAACCAATTCGAATTTGGA

TCTTGACATGTCGGTGGTTTTTAACCGTAGGCATCTTGCCAGGAAGTTGGTGGGCTCATCATGAATTAGG

TCGGGGTGGCTGGTGGTTTCGGGATCCCGTAGAAAATGTTTCTTTTATGCCTCGGGTATTAGCCACAGCT

CGTATTCATTCAGTAATTATACCCCTTCTTCATTCTTGGACCTCGCTTCTTAATATTGTGACTCTTCCAT

GCTGTGTCTCAGGAACCTCTTCAATACGGTCCGGATTGCTAGCTCCCGTTCATAGTTTTGCTACAGATGA

TACACGAGGAAGATTTTTATGGCGGTTCTTCCTTCTAATGACCGGCATATCTATGATTCTTTTCTCCCAG

ATGAAGCAGCAGGCATCGGTCCGTATAACCCATAAAAAAGAGATGGTTGTGACGCGAAGTACTCTTGTGT

ACCTACGT---TGATTGTTCTAGAATGGCTATTCCTCACAATTGCTCCTTGTGATGCAGCAGAACCATGG

CAATTAGGATCTCAAGACGCAGCAACACCTACGATGCAAGGAATAATAGACTTACATCACGATATCTTTT

TCTTCCTCATTCTTATTTTTGTTTTCGTATCACGGATCTTGGTTCGTGCTTTATGGCATTTCCACTATAA

AAAAAATCCAATCCCGCAAAGGATTGTTCATGGAACTACTATCGAGATTCTTCGGACCATATTTCCTAGT

ATCATCCCGATGTTCATTGCTATACCATCATTTGCTCTGTTATACTCAATGGACGAGGTAGTAGTAGATC

CAGCCATTACTATCAAAGCTATTGGACATCAATGGTATCGGACTTATGAGTATTCGGACTATAACAGTTC

CGATGAACAGTCACTCACTTTTGACAGTTATACGATTCCAGAAGATGATCCAGAATTGGGTCAATCACGT

TTATTAGAAGTGGACAATAGAGTGGTTGTACCAGCCAAAATTGATCTACGTATTATTGTAACACCTGCTG

ATGTACCTCATAGTTGGGCTGTACCTTCCTCAGGTGTCAAATGTGATGCTGTACCTGGTCGTTTAAATCA

GACCTCTATTTCGGTACAACGAGAAGGAGTTTACTATGGTCAGTGCAGTGAGATTTGTGGAACTAATCAT

GCCT------------------------------------------------------------------

-----------------------ATGAGACGACTCTTTCTTGAACTATTTCATAAACAGATCTTCCCCGT

AACACAAATCACGAGTTTTTCTCCATTCCTCTCATATATCGTCGTAACGCCCTTAATGCTAGGTTTTTCA

AAAGACTTTTCATGTCATTCCCATTTAGGTCCGATTCGGATCCATCCGTTGTTTCCTTTTTCTTCCGCAC

CTTTTCCTCGAAATGATAAAGAAGATGGTACACTTGAATTGTATTATTTAAGTGCTTATTGCTTGCCAAA

GATCCTACTTCTACAATTGGTAGGTCACCGGGTTATTCAAATAAGTCGTGTTTTCCGTGGTTTTCACATG

TTACAACTTCCGTACCAATTCGGTCGATCCGGAATAGATCGGTTAAACATTCTATTAGGGAGCCTGGTCT

TGACTCTTCTGTGTGGTATTCATTCTCGTTCGGCTCTTAGAATCACATCCAGCAGTGGTTGGAACAGCTC

GCAAAATCCAACCACTTCACCTACTTCATTGCCCCCAACCGTTTCTCGTACCTCTATTGAAACAGAATGG

TTTCATGTTCTTTCATCGATTGGTTATTCCTCTCCGTTCGTATCTCTTTTTCCAATTTCGGTCTCGATTA

GTTCACAAGAT---ATGTCCGTTTCGTTATTACAACCTTC-----TTTTTTGATGTCAAAGACCAGAAGC

TACGCGCAAATTCTCATTGGATTTCGGTTGTTCTTAACAGCGATGGCTATTCATTTAAGTCTTCGGGTAG

CACCACTAGATCTTCAACAAGGTGGAAATTCTCGTATTCCGTATGTACATGTTCCTGCGGCTCGGATGAG

TATTCTTGTTTATATCGCTACGGCTATAAACACTTTCTTGTTCCTATTAACAAAACATCCCCTTTTTCTT

CGCTCTTCCGGAACCGGTACAGAAATGGGTGCTTTTTCTACGTTGTTTACCTTAGTTACTGGGGGGTTTC

GGGGAAGACCTATGTGGGGCACCTTTCGGGTGTGGGATGCTCGTTTAACCTCTGTATTCATCTCGTTCCT

TATTTACCTGGGTGCACTGCGTTTTCAAAAGCTTCCTGTCGAACCGGCTCCTATTTCAATCCGTGCTGGA

CCGATCGATATACCAATAATAAAGTCTTCAGTCAACTGGTGGAATACATCGCATCAACCTGGGAGCATTA

GCCGATCTGGTACATCAATACATGTTCCTATGCCCATTCCAATCTTGTCTAACTTTGCAAACTCCCCCTT

CTCAACCCGTATCTTCTTTGTTCTGGAAACACGTCTTCCTATTCCATCTTTTCTCGAATCCCCTTTAACG

AATAAAATAGAAGCTCGAGAAGGAATAC-TGCAGGCTAGAAAGATGCTATTTGCTGCTATTCTATCTATT

TGTGCATCAAGTTCGAAGAAGATCTCAATCTATAATGAAGAAATGATAGTAGCTCGTTGTTTTATAGGCT

TTATCATATTCAGTCGGAAGAGTTTAGGTAAGACTTTCAAAGTGACTCTCGACGGGAGAATCCAGGCTAT

TCAGGAAGAATCGCAGCAATTCCCCAATCCTAACGAAGTAGTTCCTCCGGAATCCAATGAACAACAACGA

TTACTTAGGATCAGCTTGCGAATTTGTGGCACCGTAGTAGAATCATTACCAATGGCACGCTGTGCGCCTA

AGTGCGAAAAGACAGTGCAAGCTTTGTTATGCCGAAACCTAAATGTTAAGTCAGCAACACTTCCAAATGC

CACTTCTTCCCGTCGCATCTGTCTTCAGGACGATCTAGTCACAGGTTTTCACTTCTCAGTGAGTGAAAGA

TTTGTCCCCGGGTGTACGTTGAAAGCTTCTATAGTAGAACTCATTCGAGAGGGCTTGGCGGTCTTAAGAA

TGGTTCGGGTGGGGGGTTTCTCT-ATGAAAGAGGCGATCAGAATGGTACTCGAATCCATTTACGATCCCG

AGTTTCCAGACACATCGCACTTCCGCTCGGGTCGAGGCTGCCACTCGGCCCTAAGACGGATCAAAGAAGA

GTGGGGAACCTCTCGCTGGTTTTTGGAATTCGACATCTTGAAGTGTTTTCACACCATCGACCGACATCGA

CTCATCTCAATCTTTAAGGAAGAGATCGACGATCCCAAGTTCTTTTACTCCATTCAGAAAGTGTTTTCCG

CCGGACGACTCGTAGGAGGTGAGAAGGGCCCTTACTCCGTTCCACACAGTGTACTACTATCGGCCCTACC

AGGCAACATCTACCTACACAAGCTCGATCAGGAGATAGGTAGGATCCGACAGAAGTACGAAATTCCGATT

GTTCAGAGAATCAGATCGGTTCTATTAAGGACAGGTCGTATTGATGACCAAGAAAACTCTGGAGAAGAAG

C--TGGAAATCTCTCCCAGAGCTGCGGAACTAACGACTCTATTAGAAAGTAGAATTACCAACTTTTACAC

TCATTTTCAAGTGGATGAGATCGGTCGAGTGGTCTCAGTTGGAGATGGGATTGCACGTGTTTATGGATTG

AACGAGATTCAAGCTGGGGAAATGGTGGAATTTGCCAGCGGTGTGAAAGGAATAGCGTTGAATCTTGAGA

ATGAGAATGTAGGGATTGTTGTCTTTGGTAGTGATACATATATTAAAGAAGGCGATCTTGTCAAGCGCAC

TGGATCTATTGTGGATGTTCCTGCGGGAAAGGCTATGCTAGGGCGTGTGGTCGACGGGTTGGGAGTACCT

ATTGATGGAAAAGGGTCTCTAAGCGATCACGAGCGAAGACGTGTCGAAGTGAAAGCCCCTGGGATTATTG

AACGTAAATCTGTGCACGAGCCTATGCAAACAGGGTTAAAAGCGGTAGATAGCCTGGTTCCTATAGGCCG

TGGTCAACGAGAACTTATAATCGGGGACCGACAAACTGGAAAAACAGCTATAGCTATCGATACCATATTA

AACCAAAAGCAAATGAACTCAAGGAGCACCTCTGAGAGTGAGACATTGTATTGTGTCTATGTAGCGATTG

GACAGAAACGCTCAACTGTGGCACAATTAGTTCAAATTCTTTCAGAAGCGAATGCTTTGGAATATTCCAT

TCTTGTAGCAGCCACCGCTTCGGATCCTGCTCCTCTGCAATTTCTGGCCCCATATTCTGGGTGTGCCATG

GGGGAATATTTCCGCGATAATGGAATGCACGCATTAATAATATATGATGATCTTAGTAAACAGGCGGTAG

CATATCGACAAATGTCATTATTGTTACGCCGACCACCAGGCCGTGAGGCTTTCCCTGGGGATGTTTTCTA

TTTACATTCCCGTCTCTTAGAAAGAGCCGCTAAACGATCGGACCAGACAGGCGCAGGTAGCTTGACCGCC

TTACCCGTCATTGAAACACAAGCTGGAGACGTATCGGCCTATATTCCCACCAATGTGATCCCCATTACTG

ATGGACAAATCTGTTTGGAAACAGAGCTCTTTTATCGCGGAATTAGACCAGCTATTAACGTCGGCTTATC

TGTCAGTCGCGTCGGGTCTGCCGCTCAGTTGAAAGCTATGAAACAAGTCTGCGGTAGTTCAAAACTTGAA

TTGGCACAATATCGCGAAGTGGCCGCCCTTGCTCAATTTGGGTCAGACCTTGATGCTGCGACTCAGGCAT

TACTCAATAGAGGTGCAAGGCTTACCGAAGTATCGAAACAACCACAATATGCACCACTTCCAATTGAAAA

AGAAATTATAGTCATTTATGCAGCTGTCAATGGATTCTGTGATCGAATGCCATTAGACAAAATTTCTCAA

TATGAGAGAGCCATTCCAAGTAGTGTAAAACCAGAATTACTACAATCTCTTTTAGAAAAAGGTGGGTTAA

CTAACGAAAGAAAGATGGAACCAGATGCATTCTTAAGAGAAAGTGCTTTGCCTTACCTATGATGCAAGAA

ATAAT--------------AAGCCGCCTTTCATTCCCTTGTTTCGTCGTGGCACACCCCCCCCACAAGCA

CCCTTAGGAGCCGGGGGGACCAGAAAACGCCTTTCGTTTTCCCCCCTTCGTCGGCCCTTGCCGCCTTCCT

TAACAAGCCCTCGAGCCTCCTTTGCGCCGCCTTCCTAATAGAAGCCGCCGGGTTGACCCCGAAGGCCTCA

TTCTATAGTAGAGAAGGCTGTAATAATAATTGGGCCATGAGAGACTTTATTAAGTATTGCAAAAGAAAGG

GCCTGCTGATAGAGCTGGGCGGGGAGGCGATACTAGTTATCAGGTCAGAGAGACGCCTGGCCCGTAAGCT

GGCCCCCTTAAAAAGCCATTACTTAATAAGGATTTGTTACGCGCGATATGCCGACGACTCACTACTGGGA

ATCGTGGGTGCCGTAGAGCTTCTCATAGAAATACAAAAACGTATCGCCCACTTCCTACAATCCGGCCTGA

ACCTTTGGGTAAGCTCTGCAGGATCAACAACAATAACTGCACTACGTACGGTAGAATTCCTCGGTACGAT

CATTCGGGAAGTCCCTCCGAGGACGACTCCCATACAATTCTTGCGAGAGCTGGAGAAGCGTCTACGGGTA

AAGCACCGTATCCATATAACTGCTTGCCACCTACGCTCCGCCATCCATTCCAAGTTTAGGAACCTAGGTA

ATAGTATCCCGATCAAACAGCTGACGAAGGGGATGAGCGGAACAGGGAGTCTACTGGACGCGGTTCAACT

AGCGGAGACTCTTGGAACAGCTGGAGTAATCAGTCCCCAAGTGAGCGTATTATGGGGGACCGTCAAGCAC

ATCCGGCAAGGATCAAGGGGGATCTCGTTGTTGCATAGCTCAGGTCGGAGCAAGGTGCCATCGGACGTTC

AACAGGCAGTCTCACGATCGGGCATGAGTGCCCGGAAGTTGTC---ATTGTATACTCCCGCGGGTCGGAA

GGCGGCGGGGGAAGGAGGGGGACACTGGGCGAGATCTATCAGCAGCGAATTCCCCATACAAATAGAGGCG

CCTATCAAAAAGATACTCCGAAGGCTTCGGGATCGAGGTCTCATTAGCCGAAGAAGACCCTGGCCAATCC

ACGTGGCCTGCTTGACGAACGTCAGCGACGGAGACATCGTAAATTGGTCCGCGGACATTGCGATAAGTCC

TCTGTCCTACTACAGGTGCCGCGACAACCTTTACCAAGTCCGAACGATTGTCGACCACCAGATCCGCTGG

TCTGCAATATTCACCCCGGCCCACAAGCACAAATCCTCGGCGCGGAATATAATCCAAAAGTACTCCAAAG

ACTCATCTATAGTAAATCAAGAAGGTGGTAAGACCCTTGCAGAGTTCCCCAACAGCATAGAGCTTGGGAA

GCTCGGACCCGGTCAAGATCC-GAACAACAAGGAGCACTCAACTACT------------------ATGGT

CCAACTACATAACTTTTTCTTTTTCATTACTTCCATGGTCGTGCCTCGTGGCACGGCAGCACCCGTACTA

TTGAAATGGTTCGTCAGTAGAGATGTTCCCACAGGTGCCCCTTCTTCCAATGGTACTATAATTCCTATTC

CTATCCCTTCATTCCCTCTTTTGGTCTATCTACATTCCAGGAAATTCATACGCCCCACGGACGGAGCAAA

AAGTGGAGTCTTGGTCAGAGCAAGCCGCCCTATTCTAT------TACCAGACATAATTGGGAGAAGCTCA

TCCGAAACTAGAGAAAGAAACGCCTCATTTCGGTTTGTTCCTGTTCTTAATTTCCTTCTTCTTCAATCCA

A------GGGGGACTTCTCATATTTAGAATCTTTCTGCGGTGTGTTCCGTTTACTATTATTTCGTACTTT

CTTCTTTTTACCACGCGATAGGTCAGCGAAGCGTGAGCGGGCGCGGAGAAGGAAAGGCCAAACACTTCGG

CCTAAC------GGGAATGAGCAACGACGAAATGACAAGATGAGGTGCCCCGGGCATCCCCATT------

TAGA---AAGAAGGATCGATGGTTTTGGGCCTGTAGCTTTTCCCGTCCCCCCTTCGTCGGGTGGTCCTTG

TGTGGGGGGTGCGCCACCTTCAATCGGGCTTGAAGCTCTCGCCTTACCAACGAGCCGACAGCTGATGGCT

GTTGGTCACGACTACTACCAAAAAGCTCCAATAAAGATTCATATTTCACATGGAGGAGTATGCATCTGTA

TGTTGGGTGTTCTTCTG----TACATAGCTGTTCCAGCGGAAATACTTGGAATAATTCTACCACTTCTAC

TAGGAGTAGCCTTTTTAGTGCTAGCTGAACGTAAAGTAATGGCTTTTGTGCAACGTCGAAAGGGTCCTGA

TGTAGTTGGATCGTTTGGATTGTTACAACCTCTAGCAGATGGTTTGAAATTGATTCTAAAAGAACCTATT

TCACCAAGTAGTGCAAATTTCTCCCTTTTTAGAATGGCTCCAGTGGCTACATTTATGTTAAGTCTGGTCG

CTCGGGCCGTTGTACCTTTTGATTATGGTATGGTATTGTCAGATCCGAACATAGGGCTACTTTATTTGTT

TGCCATATCTTCGCTAGGTGTTTATGGAATTATTATAGCAGGTCGGTCTAGTA-TTATTATATACGCTTA

GTGAAAAGAATGTTTTTTGATACACCTAGGACATGGATTCTATATGAACCAATGGATCGTGACAAGTCGT

TACTACTAGCAATGACTTCCTCTTTCATTACTTCATTCTTTCCATATCCCTCTCCTTTGTTCTCAGTTAC

TCATCAAATGGCACTCAGTTCATATCTTTAA-TGTCAGAATTTGCACCTATTTGTATCTATTTAGTGATC

AGTCCGCTAGTTTCTTTGATCCTACTCGGTCTTCCTTTTCCATTTTCTTCCAATAGTTCGACCTATCCAG

AGAAATTGTCGGCCTACGAATGTGGTTTCGATCCTTCCGGTGATGCCAGAAGTCGCTTTGATATACGATT

TTATCTTGTTTCAATTTTATTTATTATCCCTGACCCGGAAGTAACCTTTTCTTTTCCTTGGGCAGTACCT

CCCAACAAGATTGATCCGTTTGGATCTTGGTCCATGATGGCCTTTTTATTGATTTTGACGATTGGATCTC

TCTATGAATGGAAAAGGGGTGCTTCGGATCGGGAATAA--AAGTGTTTCTTACGATTACGCCCAACAGCC

CACTTGAGCAATTTGCCATTCTCCCATTGATTCCTATGAATATAGGAAACTTGTATTTCTCATTCACAAA

TCCATCTTTGTTTATGCTGCTAACTCTCAGTTTGGTCCTACTTCTGTTTCATTTTGTTACTAAAAACGGA

GGAGGAAACTCAGTACCAAATGCTTTGCAATCCTTGGTAGAGCTTATTCATGATTTCGTGCCGAACCCGG

TAAACGAACAAATAGGTGGTCTTTCCGGAAATGTTAAACAAAAGTTTTCCCCTCGCATCTCGGTCACTTT

TACTTTTTTGTTATTTCGTAATCCCCAGGGTATGATACCTTATAGCTTCACAGTTACAAGTCATTTTCTC

ATTACTTTGGGTCTCTCATTTTCTCTTTTTATTGGCATTACTATAGTGGGATTTCAAAGAAATGGGCTTC

ATTTTTTAAGCTTCTCATTACCCGCAGGAGTCCCACTGCCGTTAGCACCTTTTTTAGTACTCCTTGAGCT

AATCCCTCATTGTTTTCGCGCATTAAGCTCAGGAATACGTTTATTTGCTAATATGATGGCCGGTCATAGT

TCAGTAAAGATTTTAAGTGGGTCCGCTTGGACTATGCTATGTATGAATGATCTTTTATATTTCATAGGAG

ATCTTGGTCCTTTATTTATAGTTCTTGCATTAACCGGTCCGGAATTAGGTGTAGCTATATCACAAGCTCA

TGTTTCTACGATCTCAATCTGTATTTACTTGAATGATGCTACAAATCTCCATCAAAG-------------

-----------------TATGTGGGCACCTGATATCTATGAGGGTTCACCCACCCCGGTTACAGCATTCC

TTTCTATTGCGCCTAAAATCTCTATTTCTGCTAATATTTCACGTCTTTCTATTTATGGTTCCTATGGAGC

TACATTGCAACAAATCTTCTTTTTCTGCAGCATTGCTTCTATGATCTTAGGAGCACTGGCCGCCATGGCC

CAAACGAAAGTCAAAAGACTTCTAGCTTATAGTTCAATTGGACATGTAGGTTATATTCGTACTGGTTTCT

CATGTGGAACCATAGAAGGAATTCAATCACTACTAATTGGTATCTTTATTTATGCATCAATGACGATAGA

TGCATTCGCCATAGTTTCAGCATTACGGCAAACCCGTGTCAAATATATAGCGGATTTGGGCGCTCTAGCC

AAAACGAATCCTATTTCGGCGATTACCTTCTCCATTACTATGTTCTCATACATAGGAATACCCCCGTTAG

CCGGCTTTTGTAGCAAATTCTATTTGTTCTTCGCCGCTTTGGGTTGTGGGGCTTACTTACTAGCCATAGT

GGGAGTATTGACTAGCGTTATAGGTCGTTTGTTCGATAGCCCGACCGTAGTGATGTTAATTGTGGTTACA

TCCATAAGTAGCTTGGTCCATCTTTATTCCATTTCATATATGTCTGAGGATCCGCATAGCCCTCGATTTA

TGTGTTATTTATCCATTTCTACTTTTTTTATGCCAATGTTGGTGACTGGAGATAACTCTCTTCAATTATT

CCTGGGATGGGAGGGAGTAGGTCTTGCTTCATATTTGTTAATCAATTTCTGGTTTACACGACTTCAGGCA

GATAAAGCAGCTATAAAAGCTATGCCTGTCAATCGAGTAGGTGATTTTGGATTAGCTCCTGGGATTTCGG

GTTGTTTTACTCTCTTTCAAACAGTAGACTTTTCAACCATTTTTGCTTGTGCGTCTGCCCCCAGAAATTC

TTGGATTTCTCGCAATATGAGATTGAATGCCATAACTCTGATTTGTATTTTACTTCTTATTGGTGCTGTT

GGGAAATCTGCACAGATAGGATCGCATACTTGGTCACCCGATGCTATGGAGGGTCCCACTCCAGTATCCG

CTTTGATTCATGCAGCTACTATGGTAACAGCTGGCGTTTTCATGATAGCAAGGTGCTCCCCTTTATTTGA

ATACCCACCTACGGCTTTGATTGTTATTACTTTTGCAGGAGCTACGACGTCATTCCTTGCGGCAACCACT

GGAATATTACAGAACGATCTAAAGAGGGTCATAGCTTATTCAACTTGCAGTCAATTAGGCTATATGATCT

TTGCTTGCGGCATCTCTAACTATTCGGTTAGCGTCTTTCACTTAATGAATCACGCGTTTTTCAAAGCATT

ACTATTCCTGAGTGCAGGTTCGGTGATTCATGCCATGTCGGATGAGCAAGATATGCGGAAGAT---GGGG

TTCCAAACCTCGTTCCCTTTTACCTATGCCATGATGCTCATGGGCAGCTTATCTCTAATTGGATTTCCTT

TTCTAACTGGATTTTATTCCAAAGATGTGATCTTAGAGCTCGCTTACACTAAGTATACCATCAGTGGGAA

CTTTGCTTTCTGGTTGGGAAGTGTCTCTGTCCTTTTCACTTCTTATTACTCTTTTCGTTCACTTTTTCTA

ACATTTCTAGTACCAACTAATTCATTCGGGCGAGACATCCTACGATGTCATGATGCGCCCATTCCTATGG

CCATTCCTTTAATACTTCTGGCTCTCGGGAGTCTCTTTGTAGGATACTTGGCCAA-CTAACACAAAGAAG

ATAGAGTTCACTCAACGATTGCCTTTGGGTTCCGAACTCTATATGGAGAAGGAGCGTTGTAGTTTGCGAG

GTCTCGATCATTTACATGGACCCACTTTTCATTCTATTTGTGGGAATTTTATGATCTATAAACCGTCCTT

AACGAACGATCGGCTCATGCTCAAAGATGAGCATGATGAATCACTTCGTGCCGACCTGTTGCCAATAAAC

TTTCCGGCCTCATATGAGAATGGAAAACTGGAGCATTTTCTGCATCGGTGGATGAAGAATCTCGAACATA

AGAATTTCTGGTTGACCATGTTCCTAGAAAACAGAAACTTTCGAGAAACGACGAGTACGACTGAAGTGGC

TATACATACAAATCCATTTACGGATCTATATGCTTCGATTGGAACTTCCAGTTCCAGAACATGCGGCTGG

TATACCACCATAATGAAACTGCCTTTTATTTTTTTTATTTGGATAGGATTTATGTTGGCTTCGTTGGGAG

GCTCGCGTAGTTTGTTACGTCAGCTCCAAAAGGATAAGTTGCGTTGGAATCGAGAAAGTTCCGTGG----

AGTTAATAATTGCATA-CCAATTTTTGGGCCAATTCCCCCTTCGTACTACCAAAAAATGAGATTCTTGCC

GAATCCGAGTTTGCTGCTCCAACCATTACCAAACTAATACCTATTCCGTTTAGTACTTCAGGTGCTTCTG

TAGCGTATAATGTAAATCCCGTAGCGGATCAATTCC---------------AACGAGCCTTTCAAACTAG

TACTTTTTGTAATCGACTCTATAGCTTCTTCAATAAACGCTGGTTCTTCGATCAAGTTTTGAATGACTTT

CTAGTCAGATCGTTCCTGCGTTTCGGATATGAAGTCTCATTCGAAGCTTTAGACAAAGGTGCTATTGAGA

TATTGGGCCCTTATGGTATCTCGTACACATTCCGACGATTGGTCGAGCGAATAAGTCAACTTCAAAGTGG

ATTTGT--TGTTCCATGATCTATGGGTCTACTGGAGCTACCCACTTCGATCAATTAGCCAAGATTTTGAC

CGGATACGAAATCACTGGTGCTCGATCTAGTGGTATTTTTATGGGGATTCTATCTATCGCTGTAGGATCC

CTATTCAAGATCACTGCAGTTCCTTTTCTGGTCCATGCACATCGCTCTCTCCAGGAGGTTGGCCGCCTAT

CCTAGATCTTCCCATTTCCAAGAAGATCCCGGGCTCGATCCGGTTTAGTATCAAGGTGATTCTTTTTCTC

TTTCTATATATATGGGTCCGTGCAGCATTTCCACGATATCGTTATGATCAATTAATGGGACTTGGCCGGA

AAGTGTTCTTGCCTCTATCATTAGCTCGGGTAGTCGCCGTTTCTGGTGTTTTAGTCACCTTTCAATGGCT

CCCTTA--TGCCTCAACTGGATAAATTCACTTATTTCACACAATTCTTCTGGTCATGCCTTTTCCTCTTT

ACTTTCTATATTCCCATATGCAATGATGGAGATGGAGTACTTGGGATCAGCAGAATTCTCAAACTACGGA

ACCAACTGGTTTCACACCGGGAGAACAACATCCGAA------GCAACGACCCCAAGAGTTTTGAAGATAT

CTTGAGAAAAGGTTTTAGCACCGGTGTATCCTATATGTACTCCAGTTTATTCGAAGTATCCCAATGGTGT

AACGCCGTCGACTTATTGGGAAAAAGGAGGAAAAGCACTTTTCTCTCTTGTTTCGGAGAAATAAGTGGCT

CACGAGGAATGGAAAGAAACATATTCTATTTGATCTCGAAGTCCTCATATAGCACTTC------------

---TTCCAATCCTGAATGGGGGATCACTTGTAGGAATGACATAATGCTCATCCATGTTCCACACGGCCAA

GGAAGCATCGTTTTTT-------

>Actinidia eriantha MZ959063.1

ATGATACTTTCTGTTTTGTCAAGCCCTGCTTTGGTCTCTGGTTTGATGGTTGTACGTGCTAAAAATCCGG

TACATTCCGTTTCGTTTCCCATCCCAGTCTTTCGCGACACTTCAGGTTTACTTCTTTTGTTAGGTCTCGA

CTTCTTCGCTATGATCTTCCCAGTAGTTCATATAGGAGCTATAGCCGTTTCATTCCTATTCGTTGTTATG

ATGTTCCATATTCAAATTGCGGAGATTCACGAAGAAGTATTGCGCTATTTACCAGTGAGTGGTATTATTG

GACTGATCTTTTGGTGGGAAATGTTCTTCATTTTAGATAATGAAAGCATTCCATTACTACCAACCCAAAG

AAATACGACCTCTCTGAGATATACGGTTTATGCCGTAAAGGTACGAAGTTGGACTAATTTGGAAACATTG

GGCAATTTACTTTATACCTACTATTCCGTCTGGTTTTTGGTTCCTAGTCTTATTTTATTAGTAGCCATGA

TTGGGGCTATAGTACTGACTATGCATAGGACTACTAA------GGTGAAAAGACAGGATGTATTCCGACG

AAATGCTATTGATTCTAGGAGGACTATAATGAGGAGGACGACAGAC----------------------TC

ATTATTCGTTATTTCCGGGTCTTTTAGTTGCATTCACTTACAACAAGAAAGAACCACCTGTGTTTGGTGC

AGCACCTGCATTTTGGTGTATTCTTCTTTCTTTCCTTGGTCTTTCGTTCCGTCATATTCCTAATAACTTA

TCCAATTACAACGTATTAACTGCTAATGCACCTTTCTTTTATCAAATCTCAGGGACATGGTCTAATCATG

AGGGTAGTATTTTATCATGGTGTCGGATCCTAAGTTTTTATGGATTTCTTCTTTGTTACCGGGGTCGACC

CCAAAGCCATAATGTCTCAAAACGAGTAGGCCATAGAGAAAGGCTTTTATATTCCTTTGTCTTGAACTTC

GTGAAGAACTCCATTCTATCTCTCCCTCGTTACGAACAAAAAAGTGGGGCT------GCGCCCCAGTTGT

ACACTCCCTTCGTTCTACGAA---CCCTTGTTGATTCTGAACTTCGTTCGCGAAGGAACCGGACTTTTGA

CGGGCCAGCTCTTTTTTATGCGCCGCTTTACCCTGAAAGGAAAATGAGCTTTGCTCCTCTGGGCGCTAGG

CGCTCCCGTGGTTCGCGAGAAGGAAAAAGGACTCATCCTTTGTTGCATCTGGCACTAGATAATAAAGAGA

GAGCTTCGTCTATCGATGAACAGCGGATTGACAGAGCTCTTGGCATTGCTTTGTTTTTCTCTCCTTTCCT

ATCAGCGAGTTCCGATCCTGTTGTTCGAAATTTATTCGTTCGTACCGAACCGCTTGCAGAATCAAATCCT

GTTCCACAAGATCCTATATCAGCTATACATCCTCCTTGCATTTATGCCGGAGACGTCGCCAGTGCTATGG

GCTTTGGCTTATGTAGATCAAAAATGATGAATGGGATTGTGGCACTCTACTCGCCGCCAATGCGGAAGGA

TGCCGCCGAAAAGAATGGAACGCTGTTTCGCTCTGCTGGATGCGTCGGATCCTGTATAACAAGCGAGCTC

TTTACCCTCAAATTCAAACATGTGGGCGCTAAATGCTATCCAGCTCTATTCTTACGTAGCAATAGAAGCC

CGCTTATGATGCTGCTTCGGCGGCGCTTTTTCGCCTTCTCTTCGCTCTGGACAGGAGCGCTAGTGGACGC

GGGGAGGGAGCA------GGCGAAGCCTGTCG------TTCGTAATGGAAAGAAAGATACCACTACAAAG

CCTCTTTGTTGGACCGCCGGCGCGAACACAGTGGTCTCTGACCAGGACCAGGAACCAATTCGAATTTGGA

TCTTGACATGTCGGTGGTTTTTAACCGTAGGCATCTTGCCAGGAAGTTGGTGGGCTCATCATGAATTAGG

TCGGGGTGGCTGGTGGTTTCGGGATCCCGTAGAAAATGTTTCTTTTATGCCTCGGGTATTAGCCACAGC-

--TATTCATTCAGTAATTATACCCCTTCTTCATTCTTGGACCTCGCTTCTTAATATTGTGACTCTTCCAT

GCTGTGTCTCAGGAACCTCTTCAATACGGTCCGGATTGCTAGCTCCCGTTCATAGTTTTGCTACAGATGA

TACACGAGGAAGATTTTTATGGCGGTTCTTCCTTCTAATGACCGGCATATCTATGATTCTTTTCTCCCAG

ATGAAGCAGCAGGCATCGGTCCGTATAACCCATAAAAAAGAGATGGTTGTGACGCGAAGTACTCTTGTGT

ACCTACG----TGATTGTTCTAGAATGGCTATTCCTCACAATTGCTCCTTGTGATGCAGCAGAACCATGG

CAATTAGGATCTCAAGACGCAGCAACACCTACGATGCAAGGAATAATAGACTTACATCACGATATCTTTT

TCTTCCTAATTCTTATTTTTGTTTTCGTATCACGGATCTTGGTTCGTGCTTTATGGCATTTCCACTATAA

AAGAAATCCAATCCCGCAAAGGATTGTTCATGGAACTACTATCGAGATTCTTCGGACCATATTTCCTAGT

ATCATCCCGATGTTCATTGCTATACCATCATTTGCTCTGTTATACTCAATGGACGAGGTAGTAGTAGATC

CAGCCATTACTATCAAAGCTATTGGACATCAATGGTATCGGACTTATGAGTATTCGGACTATAACAGTTC

CGATGAACAGTCACTCACTTTTGACAGTTATACGATTCCAGAAGATGATCCAGAATTGGGTCAATCACGT

TTATTAGAAGTGGACAATAGAGTGGTTGTACCAGCCAAAATTGATCTACGTATTATTGTAACACCTGCTG

ATGTACCTCATAGTTGGGCTGTACCTTCCTCAGGTGTCAAATGTGATGCTGTACCTGGTCGTTTAAATCA

GACCTCTATTTCGGTACAACGAGAAGGAGTTTACTATGGTCAGTGCAGTGAGATTTGTGGAACTAATCAT

GCCT------------------------------------------------------------------

-----------------------ATGAGACGACTCTTTCTTGAACTATTTCATAAACAGATCTTCCCCGT

AACACAAATCACGAGTTTTTCTCCATTCCTCTCATATATCGTCGTAACGCCCTTAATGCTAGGTTTTTCA

AAAGACTTTTCATGTCATTCCCATTTAGGTCCGATTCGGATCCATCCGTTGTTTCCTTTTTCTTCCGCAC

CTTTTCCTCGAAATGAGAAAGAAGATGGTACACTTGAATTGTATTATTTAAGTGCTTATTGCTTGCCAAA

GATCCTACTTCTACAATTGGTAGGTCACCGGGTTATTCAAATAAGTCGTGTTTTCCGTGGTTTTCACATG

TTACAACTTCCGTACCAATTCGGTCGATCCGGAATAGATCGGTTAAACATTCTATTAGGGAGCCTGGTCT

TGACTCTTCTGTGTGGTATTCATTCTCGTTCGGCTCTTAGAATCACATCCAGCAGTGGTTGGAACAGCTC

GCAAAATCCAACCACTTCACCTACTTCATTGCCCCCAACCGTTTCTCGTACCTCTATTGAAACAGAATGG

TTTCATGTTCTTTCATCGATTGGTTATTCCTCTCCGTTCGTATCTCTTTTTCCAATTTCGGTCTCGATTA

GTTCACAAGAT---ATGTCCGTTTCGTTATTACAACCTTC-----TTTTTTGATGTCAAAGACCAGAAGC

TACGCGCAAATTCTCATTGGATTTCGGTTGTTCTTAACAGCGATGGCTATTCATTTAAGTCTTCGGGTAG

CACCACTAGATCTTCAACAAGGTGGAAATTCTCGTATTCCGTATGTACATGTTCCTGCGGCTCGGATGAG

TATTCTTGTTTATATCGCTACGGCTATAAACACTTTCTTGTTCCTATTAACAAAACATCCCCTTTTTCTT

CGCTCTTCCGGAACCGGTACAGAAATGGGTGCTTTTTCTACGTTGTTTACCTTAGTTACTGGGGGGTTTC

GGGGAAGACCTATGTGGGGCACCTTTCGGGTGTGGGATGCTCGTTTAACCTCTGTATTCATCTCGTTCCT

TATTTACCTGGGTGCACTGCGTTTTCAAAAGCTTCCTGTCGAACCGGCTCCTATTTCAATCCGTGCTGGA

CCGATCGATATACCAATAATAAAGTCTTCAGTCAACTGGTGGAATACATCGCATCAACCTGGGAGCATTA

GCCGATCTGGTACATCAATACATGTTCCTATGCCCATTCCAATCTTGTCTAACTTTGCAAACTCCCCCTT

CTCAACCCGTATCTTCTTTGTTCTGGAAACACGTCTTCCTATTCCATCTTTTCTCGAATCCCCTTTAACG

AATAAAATAGAAGCTCGAGAAGGAATAC-TGCAGGCTAGAAAGATGCTATTTGCTGCTATTCTATCTATT

TGTGCATCAAGTTCGAAGAAGATCTCAATCTATAATGAAGAAATGATAGTAGCTCGTTGTTTTATAGGCT

TTATCATATTCAGTCGGAAGAGTTTAGGTAAGACTTTCAAAGTGACTCTCGACGGGAGAATCCAGGCTAT

TCAGGAAGAATCGCAGCAATTCCCCAATCCTAACGAAGTAGTTCCTCCGGAATCTAATGAACAACAACGA

TTACTTAGGATCAGCTTGCGAATTTGTGGCACCGTAGTAGAATCATTACCAATGGCACGCTGTGCGCCTA

AGTGCGAAAAGACAGTGCAAGCTTTGTTATGCCGAAACCTAAATGTTAAGTCAGCAACACTTCCAAATGC

CACTTCTTCCCGTCGCATCTGTCTTCAGGACGATCTAGTCACAGGTTTTCACTTCTCAGTGAGTGAAAGA

TTTGTCCCCGGGTGTACGTTGAAAGCTTCTATAGTAGAACTCATTCGAGAGGGCTTGGCGGTCTTAAGAA

TGGTTCGGGTGGGGGGTTTCTCT--TGAAAGAGGCGATCAGAATGGTACTCGAATCCATTTACGATCCCG

AGTTTCCAGACACATCGCACTTCCGCTCGGGTCGAGGCTGCCACTCGGCCCTAAGACGGATCAAAGAAGA

GTGGGGAACCTCTCGCTGGTTTTTGGAATTCGACATCTTGAAGTGTTTTCACACCATCGACCGACATCGA

CTCATCTCAATCTTTAAGGAAGAGATCGACGATCCCAAGTTCTTTTACTCCATTCAGAAAGTCTTTTCCG

CCGGACGACTCGTAGGAGGTGAGAAGGGCCCTTACTCCGTTCCACACAGTGTACTACTATCGGCCCTACC

AGGCAACATCTACCTACACAAGCTCGATCAGGAGATAGGTAGGATCCGACAGAAGTACGAAATTCCGATT

GTTCAGAGAATCAGATCGGTTCTATTAAGGACAGGTCGTATTGATGACCAAGAAAACTCTGGAGAAGAAG

CAATGGAAATCTCTCCCAGAGCTGCGGAACTAACGACTCTATTAGAAAGTAGAATTACCAACTTTTACAC

TCATTTTCAAGTGGATGAGATCGGTCGAGTGGTCTCAGTTGGAGATGGGATTGCACGTGTTTATGGATTG

AACGAGATTCAAGCTGGGGAAATGGTGGAATTTGCCAGCGGTGTGAAAGGAATAGCGTTGAATCTTGAGA

ATGAGAATGTAGGGATTGTTGTCTTTGGTAGTGATACATATATTAAAGAAGGCGATCTTGTCAAGCGCAC

TGGATCTATTGTGGATGTTCCTGCGGGAAAGGCTATGCTAGGGCGTGTGGTCGACGGGTTGGGAGTACCT

ATTGATGGAAGAGGGTCTCTAAGCGATCACGAGCGAAGACGTGTCGAAGTGAAAGCCCCTGGGATTATTG

AACGTAAATCTGTGCACGAGCCTATGCAAACAGGGTTAAAAGCGGTAGATAGCCTGGTTCCTATAGGCCG

TGGTCAACGAGAACTTATAATCGGGGACCGACAAACTGGAAAAACAGCTATAGCTATCGATACCATATTA

AACCAAAAGCAAATGAACTCAAGGAGCACCTCTGAGAGTGAGACATTGTATTGTGTCTATGTAGCGATTG

GACAGAAACGCTCAACTGTGGCACAATTAGTTCAAATTCTTTCAGAAGCGAATGCTTTGGAATATTCCAT

TCTTGTAGCAGCCACCGCTTCGGATCCTGCTCCTCTGCAATTTCTGGCCCCATATTCTGGGTGTGCCATG

GGGGAATATTTCCGCGATAATGGAATGCACGCATTAATAATATATGATGATCTTAGTAAACAGGCGGTAG

CATATCGACAAATGTCATTATTGTTACGCCGACCACCAGGCCGTGAGGCTTTCCCTGGGGATGTTTTCTA

TTTACATTCCCGTCTCTTAGAAAGAGCCGCTAAACGATCGGACCAGACAGGCGCAGGTAGCTTGACCGCC

TTACCCGTCATTGAAACACAAGCTGGAGACGTATCGGCCTATATTCCCACCAATGTGATCCCCATTACTG

ATGGACAAATCTGTTTGGAAACAGAGCTCTTTTATCGCGGAATTAGACCAGCTATTAACGTCGGCTTATC

TGTCAGTCGCGTCGGGTCTGCCGCTCAGTTGAAAGCTATGAAACAAGTCTGCGGTAGTTCAAAACTTGAA

TTGGCACAATATCGCGAAGTGGCCGCCCTTGCTCAATTTGGGTCAGACCTTGATGCTGCGACTCAGGCAT

TACTCAATAGAGGTGCAAGGCTTACCGAAGTATCGAAACAACCACAATATGCACCACTTCCAATTGAAAA

AGAAATTCTAGTCATTTATGCAGCTGTCAATGGATTCTGTGATCGAATGCCATTAGACAAAATTTCTCAA

TATGAGAGAGCCATTCCAAGTAGTGTAAAACCAGAATTACTACAATCTCTTTTAGAAAAAGGTGGGTTAA

CTAACGAAAGAAAGATGGAACCAGATGCATTCTTAAGAGAAAGTGCTTTGCCTTACCTATGATGCAAGAA

ATAATGG-------------AGCCGCCTTTCATTCCCTTGTTTCGTCGTGGCACACCCCCCCCACAAGCA

CCCTTAGGAGCCGGGGGGACCAGAAAACGCCTTTCGTTTTCCCCCCTTCGTCGGCCCTTGCCGCCTTCCT

TAACAAGCCCTCGAGCCTCCTTTGCGCCGCCTTCCTAATAGAAGCCGCCGGGTTGACCCCGAAGGCCTCA

TTCTATAGTAGAGAAGGCTGTAATAATAATTGGGCCATGAGAGACTTTATTAAGTATTGCAAAAGAAAGG

GCCTGCTGATAGAGCTGGGCGGGGAGGCGATACTAGTTATCAGGTCAGAGAGACGCCTGGCCCGTAAGCT

GGCCCCCTTAAAAAGCCATTACTTAATAAGGATTTGTTACGCGCGATATGCCGACGACTCACTACTGGGA

ATCGTGGGTGCCGTAGAGCTTCTCATAGAAATACAAAAACGTATCGCCCACTTCCTACAATCCGGCCTGA

ACCTTTGGGTAAGCTCTGCAGGATCAACAACAATAACTGCACTACGTACGGTAGAATTCCTCGGTACGAT

CATTCGGGAAGTCCCTCCGAGGACGACTCCCATACAATTCTTGCGAGAGCTGGAGAAGCGTCTACGGGTA

AAGCACCGTATCCATATAACTGCTTGCCACCTACGCTCCGCCATCCATTCCAAGTTTAGGAACCTAGGTA

ATAGTATCCCGATCAAACAGCTGACGAAGGGGATGAGCGGAACAGGGAGTCTACTGGACGCGGTTCAACT

AGCGGAGACTCTTGGAACAGCTGGAGTAATCAGTCCCCAAGTGAGCGTATTATGGGGGACCGTCAAGCAC

ATCCGGCAAGGATCAAGGGGGATCTCGTTGTTGCATAGCTCAGGTCGGAGCAAGGTGCCATCGGACGTTC

AACAGGCAGTCTCACGATCGGGCATGAGTGCCCGGAAGTTGTC---ATTGTATACTCCCGCGGGTCGGAA

GGCGGCGGGGGAAGGAGGGGGACACTGGGCGAGATCTATCAGCAGCGAATTCCCCATACAAATAGAGGCG

CCTATCAAAAAGATACTCCGAAGGCTTCGGGATCGAGGTCTCATTAGCCGAAGAAGACCCTGGCCAATCC

ACGTGGCCTGCTTGACGAACGTCAGCGACGGAGACATCGTAAATTGGTCCGCGGACATTGCGATAAGTCC

TCTGTCCTACTACAGGTGCCGCGACAACCTTTACCAAGTCCGAACGATTGTCGACCACCAGATCCGCTGG

TCTGCAATATTCACCCCGGCCCACAAGCACAAATCCTCGGCGCGGAATATAATCCAAAAGTACTCCAAAG

ACTCATCTATAGTAAATCAAGAAGGTGGTAAGACCCTTGCAGAGTTCCCCAACAGCATAGAGCTTGGGAA

GCTCGGACCCGGTCAAGATCC-GAACAACAAGGAGCACTCAACTACTA-----------------ATGGT

CCAACTACATAACTTTTTCTTTTTCATTACTTCCATGGTCGTGCCTCGTGGCACGGCAGCACCCGTACTA

TTGAAATGGTTCGTCAGTAGAGATGTTCCCACAGGTGCCCCTTCTTCCAATGGTACTATAATTCCTATTC

CTATCCCTTCATTCCCTCTTTTGGTCTATCTACATTCCAGGAAATTCATACGCCCCACGGACGGAGCAAA

AAGTGGAGTCTTGGTCAGAGCAAGCCGCCCTATTCTAT------TACCAGACATAATTGGGAGAAGCTCA

TCCGAAACTAGAGAAAGAAACGCCTCATTTCGGTTTGTTCCTGTTCTTAATTTCCTTCTTCTTCAATCCA

A------GGGGGACTTCTCATATTTAGAATCTTTCTGCGGTGTGTTCCGTTTACTATTATTTCGTACTTT

CTTCTTTTTACCACGCGATAGGTCAGCGAAGCGTGAGCGGGCGCGGAGAAGGAAAGGCCAAACACTTCGG

CCTAAC------GGGAATGAGCAACGACGAAATGACAAGATGAGGTGCCCCGGGCATCCCCATT------

TAGA---AAGAAGGATCGATGGTTTTGGGCCTGTAGCTTTTCCCGTCCCCCCTTCGTCGGGTGGTCCTTG

TGTGGGGGGTGCGCCACCTTCAATCGGGCTTGAAGCTCTCGCCTTACCAACGAGCCGACAGCTGATGGCT

GTTGGTCACGACTACTACCAAAAAGCTCCAATAAAGATTCATATTTCACATGGAGGAGTATGCATCTGTA

TGTTGGGTGTTCTTCTG----TACATAGCTGTTCCAGCGGAAATACTTGGAATAATTCTACCACTTCTAC

TAGGAGTAGCCTTTTTAGTGCTAGCTGAACGTAAAGTAATGGCTTTTGTGCAACGTCGAAAGGGTCCTGA

TGTAGTTGGATCGTTTGGATTGTTACAACCTCTAGCAGATGGTTTGAAATTGATTCTAAAAGAACCTATT

TCACCAAGTAGTGCAAATTTCTCCCTTTTTAGAATGGCTCCAGTGGCTACATTTATGTTAAGTCTGGTCG

CTCGGGCCGTTGTACCTTTTGATTATGGTATGGTATTGTCAGATCCGAACATAGGGCTACTTTATTTGTT

TGCCATATCTTCGCTAGGTGTTTATGGAATTATTATAGCAGGTCGGTCTAGTATTTATTATATACGCTTA

GTGAAAAGAATGTTTTTTGATACACCTAGGACATGGATTCTATATGAACCAATGGATCGTGACAAGTCGT

TACTACTAGCAATGACTTCCTCTTTCATTACTTCATTCTTTCCATATCCT------TTGTTCTCAGTTAC

TCATCAAATGGCACTCAGTTCATATCTTTA-ATGTCAGAATTTGCACCTATTTGTATCTATTTAGTGATC

AGTCCGCTAGTTTCTTTGATCCTACTCGGTCTTCCTTTTCCATTTTCTTCCAATAGTTCGACCTATCCAG

AGAAATTGTCGGCCTACGAATGTGGTTTCGATCCTTCCGGTGATGCCAGAAGTCGCTTTGATATACGATT

TTATCTTGTTTCAATTTTATTTATTATCCCTGACCCGGAAGTAACCTTTTCTTTTCCTTGGGCAGTACCT

CCCAACAAGATTGATCCGTTTGGATCTTGGTCCATGATGGCCTTTTTATTGATTTTGACGATTGGATCTC

TCTATGAATGGAAAAGGGGTGCTTCGGATCGGGAATA---AAGTGTTTCTTACGATTACGCCCAACAGCC

CACTTGAGCAATTTGCCATTCTCCCATTGATTCCTATGAATATAGGAAACTTGTATTTCTCATTCACAAA

TCCATCTTTGTTTATGCTGCTAACTCTCAGTTTGGTCCTACTTCTGTTTCATTTTGTTACTAAAAACGGA

GGAGGAAACTCAGTACCAAATGCTTGGCAATCCTTGGTAGAGCTTATTCATGATTTCGTGCCGAACCCGG

TAAACGAACAAATAGGTGGTCTTTCCGGAAATGTTAAACAAAAGTTTTCCCCTCGCATCTCGGTCACTTT

TACTTTTTTGTTATTTCGTAATCCCCAGGGTATGATACCTTATAGCTTCACAGTTACAAGTCATTTTCTC

ATTACTTTGGGTCTCTCATTTTCTCTTTTTATTGGCATTACTATAGTGGGATTTCAAAGAAATGGGCTTC

ATTTTTTAAGCTTCTCATTACCCGCAGGAGTCCCACTGCCGTTAGCACCTTTTTTAGTACTCCTTGAGCT

AATCCCTCATTGTTTTCGCGCATTAAGCTCAGGAATACGTTTATTTGCTAATATGATGGCCGGTCATAGT

TCAGTAAAGATTTTAAGTGGGTCCGCTTGGACTATGCTATGTATGAATGATCTTTTATATTTCATAGGAG

ATCTTGGTCCTTTATTTATAGTTCTTGCATTAACCGGTCCGGAATTAGGTGTAGCTATATCACAAGCTCA

TGTTTCTACGATCTCAATCTGTATTTACTTGAATGATGCTACAAATCTCCATCAAAG-------------

----------------ATATGTGGGCACCTGATATCTATGAGGGTTCACCCACCCCGGTTACAGCATTCC

TTTCTATTGCGCCTAAAATCTCTATTTCTGCTAATATTTCACGTCTTTCTATTTATGGTTCCTATGGAGC

TACATTGCAACAAATCTTCTTTTTCTGCAGCATTGCTTCTATGATCTTAGGAGCACTGGCCGCCATGGCC

CAAACGAAAGTCAAAAGACTTCTAGCTTATAGTTCAATTGGACATGTAGGTTATATTCGTACTGGTTTCT

CATGTGGAACCATAGAAGGAATTCAATCACTACTAATTGGTATCTTTATTTATGCATCAATGACGATAGA

TGCATTCGCCATAGTTTCAGCATTACGGCAAACCCGTGTCAAATATATAGCGGATTTGGGCGCTCTAGCC

AAAACGAATCCTATTTCGGCGATTACCTTCTCCATTACTATGTTCTCATACATAGGAATACCCCCGTTAG

CCGGCTTTTGTAGCAAATTCTATTTGTTCTTCGCCGCTTTGGGTTGTGGGGCTTACTTACTAGCCATAGT

GGGAGTATTGACTAGCGTTATAGGTCGT--GTTCGATAGCCCGACCGTAGTGATGTTAATTGTGGTTACA

TCCATAAGTAGCTTGGTCCATCTTTATTCCATTTCATATATGTCTGAGGATCCGCATAGCCCTCGATTTA

TGTGTTATTTATCCATTTCTACTTTTTTTATGCCAATGTTGGTGACTGGAGATAACTCTCTTCAATTATT

CCTGGGATGGGAGGGAGTAGGTCTTGCTTCATATTTGTTAATCAATTTCTGGTTTACACGACTTCAGGCA

GATAAAGCAGCTATAAAAGCTATGCCTGTCAATCGAGTAGGTGATTTTGGATTAGCTCCTGGGATTTCGG

GTTGTTTTACTCTCTTTCAAACAGTAGACTTTTCAACCATTTTTGCTTGTGCGTCTGCCCCCAGAAATTC

TTGGATTTCTCGCAATATGAGATTGAATGCCATAACTCTGATTTGTATTTTACTTCTTATTGGTGCTGTT

GGGAAATCTGCACAGATAGGATCGCATACTTGGTCACCCGATGCTATGGAGGGTCCCACTCCAGTATCCG

CTTTGATTCATGCAGCTACTATGGTAACAGCTGGCGTTTTCATGATAGCAAGGTGCTCCCCTTTATTTGA

ATACCCACCTACGGCTTTGATTGTTATTACTTTTGCAGGAGCTACGACGTCATTCCTTGCGGCAACCACT

GGAATATTACAGAACGATCTAAAGAGGGTCATAGCTTATTCAACTTGCAGTCAATTAGGCTATATGATCT

TTGCTTGCGGCATCTCTAACTATTCGGTTAGCGTCTTTCACTTAATGAATCACGCGTTTTTCAAAGCATT

ACTATTCCTGAGTGCAGGTTCGGTGATTCATGCCATGTCGGATGAGCAAGATATGCGGAAGAT---GGGG

TTCCAAACCTCGTTCCCTTTTACCTATGCCATGATGCTCATGGGCAGCTTATCTCTAATTGGATTTCCTT

TTCTAACTGGATTTTATTCCAAAGATGTGATCTTAGAGCTCGCTTACACTAAGTATACCATCAGTGGGAA

CTTTGCTTTCTGGTTGGGAAGTGTCTCTGTCCTTTTCACTTCTTATTACTCTTTTCGTTCACTTTTTCTA

ACATTTCTAGTACCAACTAATTCATTCGGGCGAGACATCCTACGATGTCATGATGCGCCCATTCCTATGG

CCATTCCTTTAATACTTCTGGCTCTCGGGAGTCTCTTTGTAGGATACTTGGCCAAACTAACACAAAGAAG

ATAGAGTTCACTCAACGATTGCCTTTGGGTTCCGAACTCTATATGGAGAAGGAGCGTTGTAGTTTGCGAG

GTCTCGATCATTTACATGGACCCACTTTTCATTCTATTTGTGGGAATTTTATGATCTATAAACCGTCCTT

AACGAACGATCGGCTCATGCTCAAAGATGAGCATGATGAATCACTTCGTGCCGACCTGTTGCCAATAAAC

TTTCCGGCCTCATATGAGAATGGAAAACTGGAGCATTTTCTGCATCGGTGGATGAAGAATCTCGAACATA

AGAATTTCTGGTTGACCATGTTCCTAGAAAACAGAAACTTTCGAGAAACGACGAGTACGACTGAAGTGGC

TATACATACAAATCCATTTACGGATCTATATGCTTCGATTGGAACTTCCAGTTCCAGAACATGCGGCTGG

TATACCACCATAATGAAACTGCCTTTTATTTTTTTTATTTGGATAGGATTTATGTTGGCTTCGTTGGGAG

GCTCGCGTAGTTTGTTACGTCAGCTCCAAAAGGATAAGTTGCGTTGGAATCGAGAAAGTTCCGTGG----

AGTTAATAATTGCATA--CAATTTTTGGGCCAATTCCCCCTTCGTACTACCAAAAAATGAGATTCTTGCC

GAATCCGAGTTTGCTGCTCCAACCATTACCAAACTAATACCTATTCCGTTTAGTACTTCAGGTGCTTCTG

TAGCGTATAATGTAAATCCCGTAGCGGATCAATTCC---------------AACGAGCCTTTCAAACTAG

TACTTTTTGTAATCGACTCTATAGCTTCTTCAATAAACGCTGGTTCTTCGATCAAGTTTTGAATGACTTT

CTAGTCAGATCGTTCCTGCGTTTCGGATATGAAGTCTCATTCGAAGCTTTAGACAAAGGTGCTATTGAGA

TATTGGGCCCTTATGGTATCTCGTACACATTCCGACGATTGGTCGAGCGAATAAGTCAACTTCAAAGTGG

ATTTGTTTTGTTCCATGATCTATGGGTCTACTGGAGCTACCCACTTCGATCAATTAGCCAAGATTTTGAC

CGGATACGAAATCACTGGTGCTCGATCTAGTGGTATTTTTATGGGGATTCTATCTATCGCTGTAGGATCC

CTATTCAAGATCACTGCAGTTCCTTTT--GGTCCATGCACATCGCTCTCTCCAGGAGGTTGGCCGCCTAT

CCTAGATCTTCCCATTTCCAAGAAGATCCCGGGCTCGATCCGGTTTAGTATCAAGGTGATTCTTTTTCTC

TTTCTATATATATGGGTCCGTGCAGCATTTCCACGATATCGTTATGATCAATTAATGGGACTTGGCCGGA

AAGTGTTCTTGCCTCTATCATTAGCTCGGGTAGTCGCCGTTTCTGGTGTTTTAGTCACCTTTCAATGGCT

CCCTTAA-TGCCTCAACTGGATAAATTCACTTATTTCACACAATTCTTCTGGTCATGCCTTTTCCTCTTT

ACTTTCTATATTCCCATATGCAATGATGGAGATGGAGTACTTGGGATCAGCAGAATTCTCAAACTACGGA

ACCAACTGGTTTCACACCGGGAGAACAACATCCGAA------GCAACGACCCCAAGAGTTTTGAAGATAT

CTTGAGAAAAGGTTTTAGCACCGGTGTATCCTATATGTACTCCAGTTTATTCGAAGTATCCCAATGGTGT

AACGCCGTCGACTTATTGGGAAAAAGGAGGAAAAGCACTTTTCTCTCTTGTTTCGGAGAAATAAGTGGCT

CACGAGGAATGGAAAGAAACATATTCTATTTGATCTCGAAGTCCTCATATAGCACTTC------------

---TTCCAATCCTGAATGGGGGATCACTTGTAGGAATGACATAATGCTCATCCATGTTCCACACGGCCAA

GGAAGCATCGTTTTTT-------

>Bougainvillea spectabilis NC056281.1

-TGATACTTTCTGTTTTGTCGAGCCCGGCTTTGGTCTCTGGTTTGATGGTTGTACGTGCTAAAAATCCGG

TACATTCCGTTTCGTTTCCCATCCCAGTCTTTCGCAACACTTCAGGTTTACTTCTTTTGTTAGGTCTCGA

CTTTTCCGCTATGATCTTCCCAGTAGTTTATATAGGAGCTATAGCCGTTTCATTCCTATTCGTTGTTATG

ATGTTCCATATTCAAATAGCGGAGATTCACGAAGAAGTATTGCGCTATTTACCAGTGAGTGGTATTATTG

GACTGATCTTTTGGTGGGAAATGTTCTTCATTTTAGATAATGAAACCATTCCATTACTACCAACCCAAAG

AAATACGACCTCTCTGAGATATACGGTTTATGCCGGAAAGGTACGAAGTTGGACTAATTTGGAAACATTG

GGCAATTTACTTTATACTTACTATTTTGTCTGGTTTTTGGTTTCTAGTCTTATTTTATTAGTAGCCATGA

TTGGGGCTATAGTACTGACTATGCATAGGACTACTAA------GGTGAAAAGACAGGATGTATTCCGACG

AAATGCTATTGATTCTAGAAGGACTATAATGAGGAGGACGACAGACC--TGTCAATATATGAATTATTTC

ATTATTCGTTATTTCCGGGTCTTTTCATTGCATTCACTTACAACAAAAAACAACCACCAGCGTTTGGTGC

AGCACTTGCATTTTGGTGTATTCTTCTTTCTTTCCTTGGTCTTTTGTTCTGTCATATTCCTAATAACTTA

TCCAATTACAACGTATTAACCGCTAATGCACCTTTCTTTTATCAAATCTCAGGGACATGGTCTAATCATG

AAGGTAGTATTTTATTATGGTGTCGGATCCCAAGTTTTTATGGATTCCTTCTTTGTTACCGGGGTCGATC

CAAAAGCCATAATGTCTCAAAACGAGGAGGCCATAGAGAAAGTCTTATTTTTTCCTTTGTCTTAAACTTC

GTGAAGAACTCCATTCTATCTCTTCCTCGTTACGAACAAAAAAGTAGAGTTCTTCACGAACCCCAGTTGT

ACACTCTCTTCGTTCTACGAA---CTCTTGTTGATTCTGAACTTTGTTCGCGAAGGAACCGGAC------

------------TTTTTACGCGCCGCTTTACCCTGAAAGGAAAATGAGCTTTGCTCTTCTGGGCGCTAGG

CGCTCTCGTGGTTCGCGAGAAGGAAAAAGGACTCATCCTTTGTTGCATCTGGCACGAGATGATAAAGAGA

GAGCTTCGTCTATCGATGAACAGCGGATTGACGGAGCTCTTGGCATTGCTTTGTTTTTCTTTCCTTTCCT

ATCAGCGAGTTCCGATCCTTTTGTTCGAAATTTCTTCGTTCGTACCGAACCGCTTGCCGAATCAAATCCT

GTTCCACAAGATCCTATATCAGCTATACATCCTCCTTGCATTTATGCCGGAGACGTCGCCAGTGCTATGG

GCTTTGGCTTATGTAGATCAAAAATGATGAATGGGATTGTGGCACTCCACTCGCCGCCAATGCGGAAGGA

TGTCGCCGAAAAGAATGGAACGCTGCTTTGCTCTGCTGGATGCGTCGGATCCCGTATAACAAGTGAGCTC

TTTACCCTTAAATTCAAACATGTGGGCGCCAAATGCTATCCTGCTCTATTGTTGCGTAGCAATAGAAGCC

TGCTC---ATGCTGCTTCGGCGGCGCTTTTTCGCCTTCTCTTCGCTCTGGACAAGAGCGCTAGCGGACAC

GGGGAGGGAGCG------GGCGAAGCGTTTCT------TTCGTAATGGAAAGAAAGATACCACTACTTCG

CCTCTTTGTTGGACCGCCGGCGCGAACACAGTGGTCTCTGACCAGGACCAGGAACCAATTCGAATTTGGA

TCTTGATATGTCGGTGGTTTTTAACCGTAGGCATCTTGCCAGGAAGTTGGTGGGCTCATCATGAATTAGG

TCGGGGTGGCTGGTGGTTTCGGGATCCCGTAGAAAATGCTTCTTTTATGCCTCGGGTATTAGCCACAGCT

CGTATTCATTCAGTAATTTTACCCCTTCTTCATTCTTGGACTTTGCTTCTGAATATTGTGACTTTTCTAT

GCTGTGTCTTAGGAACCTTTTCAATACGGTCCGGATTGCTAGCTCCCGTTCATAGTTTTGCTACAGATGA

TACACGAGGAATCTTTTTATGGCGGTTCTTCCTTCTAATGACCGGCATATCTATGATTCTTTTCTCTCAG

ATGAAGCAGCAGGCATCGGTCCGTAGAACCTATAAAAAAGAGATGGTTGTAGCGCGAAGTACTCTTGTGC

ACTTACGT--ATGATTGTTCGAGAATGGCTATTCTTCCCAATTGCTCCTTGTGATGCAGCGGAACCATGG

CAATTAGGATTTCAAGACGCAGCAACACCTATGATGCAAGGAATAATAGACTTACATCATGATATCTTTT

TCTTCCTCATTCTTATTTTGGTTTTCGTATCATGGATCTTGGTTCGCGCTTTATGGCATTTCCACTATAA

AAAAAATCCAATCCCGCAAAGGATTGTTCATGGAACTACTATCGAGATTATTCGGACCATATTTCCAAGT

ATCATCCTGATGTTCATTGCTATACCATCATTTGCTCTGTTATACTCAATGGACGAGGTAGTAGTAGATC

CAGCCATTACTATCAAAGCTATTGGACATCAATGGTATCGGAG---------------------------

----------------------------------------------------------------------

----------------------------------------------------------------------

----------------------------------------------------------------------

----------------------------------------------------------------------

----------------------------------------------------------------------

-----------------------ATGAGACGACTCTTTTTTGAACTATATCATAAACAGATCTTCTTCTC

CACACCAATCACGAGTTTTTCTCCATTCCTCTCGTATATTGTCGTAACGCCCTTAATGCTAGGTTTTGAA

AAAGACTTTTCATGTCATTTCCATTTAGGTCCGATTCGGATCCCTCCGTTGTTTCCTTTTCCTCCCGCAC

CTTTTCTTCGAAATGAGAAAGAAGATGGTACACTCGAATTGTATTATTTAAGTGCTTATTGCTTGCCAAA

GATCCTACTTCTACAATTGGTAGGTCACCGGGTTATTCAAATAAGTCGTGTTTTCTGTAGTTTTCCCATG

TTACAACTTCTGTACCAATTCGGTCAATCCGGAATGGATCGGTTAAACATTCTATTAGGGAGCCCGGTCT

TGACTCTTCTGTGTGGTATTCATTCTTGTTTGGCTCTTGGAATCACATCCAGCAGTGGTTGGAACAGCTC

GCAAAATTTAACCACTTCACCTACTTCATTGCCCTCAACCGTTTCTCGTACCTCTATTGAAACAGAATGG

TTTCATGTTCTTTCATCGATTGGTTATTTTTCTTCGTTCGTATCTCTTTTTCCAATTTCGGTCTCGATTA

GTTCACAAGATTG--TGTCCGTTTTGTTATTACAACCTTC-----TTTTTTGATGTCAAAGACCAGAAGC

TACGCGCAAATTCTCATGGGATCTTGGTTGTTCTTAACAGCGATGGCTATTCATTTAAGTCTTTGGGTAG

CACCACTAGATCTTCAACAAGGTGGAAATTCTCGTATTCTCTATGTACATGTTCCTGTGGCTCGGATGAG

TATTCTTGTTTATATCGTTACGGCTATAAACACTTTCTTGTTCCTATTAACAAAACATCCTCTTTTTCTT

CGCTCTTCCGGAACCGGTACAGAAATGGGTGCTTTTTCTACGTTGTTTACTTTAGTAACTGGGGGGTTTC

GGGGAAGACCCATGTGGGGCACCTTTTGGGTGTGGGATGCTCGTTTAACTTCTGTATTCATCTCGTTCCT

TATTTACCTGGGTGCACTGTGTTTTCAAAAGCTTCCTGTCGAACCGGCTCCTATTTCAATCCGTGCTGGA

CCGATCGATATACCAATAATCAAGTCTCCAGTCAACTGGTGGAATACATCGCATCAACCTGGGAGCATTA

GCCGATCTGGTACATCAATACATGTTCCTATGCCCATTCCAATCTTGTCTAACTTTGCTAACTTCCTCTT

CTCAACCCGTATCTTGTTTGTTCTGGAAACACGTCTTCCTATTCCATCTTTTCTCGAATCTCCTTTAACG

GAAGAAATAGAAGCTCGAGAAGGAA----------------AGATGCTATTTGCTGCTATTCTATCTATT

TTTGCATTAAGTTCGAAGAAGATCTCAATCTATAATGAAGAAATGATAGTAGCTCTATGTTTTATAGGCT

TTATCATATTCAGTCGGAAGAGTTTAGGTAAGACTTTCAAAGTGACTCTCGACGAGAGAATCCAGGCTAT

TCAGGAAGAATCGCAGCAATTCCCCAATCCTAACGAAGTAGTTCCTCCGGAATACAATGAACAACAACGG

TTACTTAGGATCAGCTTGCGAATTTGTGGAACCGTAGTAGAATCATTACCAATGGCACGCTGTGCGCCTA

AGTGCGAAAAGGCAGTGCAAGCTTTGTTATGTCGAAACCTAAATGTTAAGTCAGCAACACTTCCAAATGC

CACTTCTTCCCGTCGCACCCGTCTTCAGGACGATCTAGTCACAGGTTTTCACTTCTCAGTGAGTGAAAGA

TTTGTCCCCGGGTCTACGTTGAAAGCTTCTATAGTAGAACTCATTCGAGAAGGCTTGGCGGTCTTAAGAA

TGGTTCGGGTAGGAGGTTCTCTT--TGAAAGAGGCGATCAGAATGGTACCCGAATCCATTTACGATCCCG

AGTTTCCAGACACATCGCACTTCCGCTCGGGTCGAGGCTGCCACTCGGCCCTAAGACGGATCAAAGAAGA

GTGGGGAACCTCTCGCTGGTTTTTGGAATTCGACATCAGGAAGTGTTTTCACACCATCGACCGACATCGA

CTCATCTCAATCTTTAAGGAAGAGATCGACGATCCCAAGTTCTTTTACTCCATTCAGAAAGTCTTTTCTG

CCGGACGACTCGTAGGAGGTGAGAAGGGCCCTTACTCCGTCCCACACAGTGTACTACTATCGGCCCTACC

AGGCAACATCTACTTACACAAGCTCGATCAGGAGATAGGGAGGATCCGACAGAAGTACGAAATTCCGATT

GTTCAGAGAATCAGATCGGTTCTATTAAAGACAAGTCGTATTGATGACCAAGAAAACTCTGGAGAAGAAG

---TGGAATTCTCTCCCAGAGCTGCGGAACTAACGACTCTATTAGAAAGTAGAATTACCAACTTTTACAC

GAATTTTCAAGTGGATGAGATCGGTCGAGTGGTCTCAGTTGGAGATGGGATTGCACGTGTTTATGGATTG

AACGAGATTCAAGCTGGGGAAATGGTGGAATTTGCCAGCGGTGTGAAAGGAATAGCCTTAAATCTTGAGA

ATGAGAATGTAGGGATTGTTGTCTTTGGTAGTGATACCGCTATTAAAGAAGGAGATCTTGTCAAGCGCAC

TGGATCTATTGTGGATGTTCCTGCGGGAAAGGCTATGCTAGGGCGTGTGGTCGACGCGTTGGGAGTACCT

ATTGATGGAAGAGGGGCTCTAAGCGATCACGAGCGTCGACGTGTCGAAGTGAAAGCCCCTGGGATTATTG

AACGTAAATCTGTGCACGAGCCTATGCAAACAGGGTTAAAGGCGGTAGATAGCCTGGTTCCTATAGGCCG

TGGTCAACGAGAACTTATAATCGGGGACCGACAAACGGGAAAAACAGCTATTGCTATCGATACCATATTA

AACCAAAAGCAACTGAACTCAAGGGCCACCTCTGAGAGTGAGACATTGTATTGTGTCTATGTAGCGATTG

GACAGAAACGTTCAACTGTGGCACAATTAGTTCAAATTCTTTCAGAAGCGAATGCTTTGGAATATTCCAT

TCTTGTAGCAGCCACCGCTTCGGATCCTGCTCCTCTTCAATTTCTGGCCCCATATTCTGGGTGTGCTATG

GGAGAATATTTCCGCGATAATGGAATGCACGCATTAATAATCTATGATGATCTTAGTAAACAGGCGGTGG

CATATCGACAAATGTCATTATTGTTACGCCGACCACCAGGCCGTGAGGCTTTCCCAGGCGACGTTTTCTA

TTTGCATTCTCGTCTCTTAGAAAGAGCCGCTAAACGATCGGACCAGACAGGTGCAGGTAGCTTGACCGCC

TTACCCGTCATTGAAACACAAGCTGGAGACGTATCGGCCTATATTCCCACCAATGTGATCTCCATTACTG

ATGGACAAATCTGTTCGGAAACAGAGCTCTTTTATCGCGGAATTAGACCTGCTATTAACGTCGGCTTATC

TGTCAGTCGCGTCGGGTCTGCCGCTCAGTTGAAAGCTATGAAACAAGTCTGCGGTAGTCCAAAACTTGAA

TTGGCACAATATCGCGAAGTGGCCGCCTTTGCTCAATTTGGGTCAGACCTTGATGCTGCGACTCAGGCAT

TACTCAATAGAGGTGCAAGGCTTACAGAAGTACCGAAACAACCACAATATGCACCACTTCCAATTGAAAA

ACAAATTCTAGTCATTTACGCAGCTGTCAATGGATTCTGTGATCGAATGCCACTAGATAAAATTTCTCAA

TATGAGAGAACCATTCCAAATAGTGTAAAACCAGAATTATTACAATCCCT------AAAGGGGGGCTTAA

CTAACGAAATAAAGATGGAACTAGATGAATTCTTAAAAGAATGCGCTTTGACTTACC-------------

--------CATCCAACGCAAAGCGGCCTTTCATTCCCTTGTTTCGTCGTGGCACACCCTCCCCACAAGCA

CCCCCCGGCTCAGGGGGGACCAGAAAAGGCCTTTCGTTTTCCCCCCTTCGTCGGCCCTTGCCACCTTCCT

TAACAAGCCCTCGAGCCTCCTTTTCGCTGCCTTCCTCATAGAAGCCGCCGGGTTGACCCCGAAGGCCGAA

TTCTATGGTAGAGAACGCTGTAATAATAATTGGGCCATGAGAGACCTTTTTAAGTATTGCAAAAGAAAGG

GCCTGCTGATAGAGCTGGGCGAGGCAGCGATACTAGTTATCAGGTCAGAGAAAGGCCTGGCCCGTAAGCT

GGCCCCCTTAAAAACCCATTACTTAATAAGGATTTGTTACGCGCGATATGCCGACGACTTACTATTGGGA

ATCGTGGGTGCCGTAGAGCTTCTCATAGAAATACAAAAACGTATCGCCCACTTCCTACAATCCGGCCTGA

ACCTTTTGGTAGGCTCTGCGGGATCAACAACAATAGCTGCACGGAGTACGGTAGAATTCCTCGGTACGGT

CATTCGGGAAGTCCCTCCGAAGACGACTCCCATACAATTCTTGCGAGAGCTGGAGAAGCGTCTACGGGTA

AAGCACCGTATCCATATAACTGCTTGCCACTTACGCTCTGCCATTCATTCCAAGTTTAGGAACCTAGGTA

ATAGTATCCCGGTCAAACAGCTGACGAAGGGGATGAGCAAAACAGGGAGTCTACTGGACGCGGTTCAACT

AGCGGAAACTCTTGGAACAGCTAGAGTAAGAAGTCCCCAAGTGAGCGTATTATGGGAGACCGTCAAGCAC

ATCCGGCAAGGATCAAGGGAGATCTCGTTGTTGCATAGCTCAGGTCAGAGCAAGGTGCCATCGGACGTTC

AACAGGCAGTCTCGCGATCGGGCATGAGTGTCCGGAAGTTGTC---ATTGTATACTCCCGCGGGTCGGAA

GGCGGCGGGGGAAGGAGGGGGACACTGGGCGAGATCTATCAGCAGCGAATTCCCCATACAGATAGAAGCG

CCTATCAAAAAGATACTCCGAAGGCTTCGAGATCGAGGTCTCATTAGCCGAAGAAGACCCTGGCCAATCC

ACGTGGCCTGCTTGACGAACGTCAGCGACGGAGACATCGTAAATTGGTCCGCGGGCATCGCGATAAGTCC

TCTGTCCTACTACAGGTGCCGCGACAACCTTTACCAAGTCCGAACGATTGTCGACCACCAGATCCGCTGG

TCTGCAATATTCACCCCAGCCCACAAGCACAAATCCTCAGCGCGGAATATAATCCCAAAGTACTCAAAAG

ACTCAAATATAGTAAATCAAGAAGGTGGTAAGACCCTAGCAGAGTTCCCCAACAGCATAGAGCTTGGGAA

GCTCGGACCCGGTCAAGATCC-GAACAACAAAGAGCACTCAACTACTA------------------TGGT

CCAACTACATAACTTTTTCTTTTTCATTACTTCCATGGTCGTGCCTTGTGGCACGGCAGCACCCGTACTA

TTGAAATGGTTCGTCAGTAGAGATGTTCCCACAGGTGCCCCTTTTTCCAATGGTACTCTAATTCCTATTC

CTATCCCTTCATTCCTTCTTTTGGTCTATCTACATTCCAGGAAATTCATACGCTCCATGGACGGAGTCAA

AAGTGGAGTCTTGGTCAGAGCAAGCTGCCCTATTTTAT------TACCAGACATAATTGGGAGAAGCTCA

TCCGAAACTAGAGCTAGAAACGCCTTATTTCGTTTCGTTCCCATTCTTCATTTTCTTCTTCTCGAATCCA

A------GGGGGACTTCCCATATTTAGAATCTTTTTGCGGTGTGCTCCGTTTACTATTCTTTCGTACTTT

CTTCTCTTTACCACGCGATAGGTCAGCGAAGCGTGAGCGGGCGCGGAGAAGAAAACGCCAAACACTTCGG

CCTAAC------GGGAATGAGCAACGACGAAATGACAAGATAAAGTGCCC---------CCATT------

TAGA---AAGAAGGGTCGAAGGGTTTGGGCCTGTAGCTTTCCCCGTCCCCCCTTCGTCGGGTGGTGCTTG

TGTGGGGGGTGTGCTACCTGAAATCGGGCTTGAAGCTCCCGCCTTACCAACGAGCCGACAGCTGATGGCT

GTTGGTCACGACTACTACCAAAAAGTGAACATGAAGATGAATATTTCACATGGAGGAGTGTGCATCTTTA

TGTTGGGTGTTCTTCTGTACGTACATAGCTGTTCCAGCTGAAATACTTGGAATAATTCTACCACTTCTAC

TAGGAGTAGCCTTTTTAGTGCTAGCTGAACGTAAAGTAATGGCTTTTGTGCAACGTCGAAAGGGTCCTGA

TGTAGTGGGATCGTTCGGATTGTTACAACCTCTAGCAGATGGTTCGAAATTGATTCTAAAAGAACCTATT

TCACCAAGTAGTGCTAATTTCTCCCTTTTTAGAATGGCTCCAGTCACTACATTTATGCTAAGTCTGGTTG

CTCGGGCCGTTGTACCTTTTGATTATGGTATGGTATTGTCAGATCCGAACATAGGGCTACTTTATTTGTT

TGCCATATCTTCGCTAGGTGTTTATGGAATTATTATAGCAGGTTGGTCTAGT-TTTATTATATACGTTTA

GTGAAAAGAATGTTTTTTGATACACCTAGGACATGGATTCTATATGAACCAATGGATCGTGACAAGTCGT

TACTACTAGCAATGACTTCCTCTTTCATTACTTCATCCTTTCCATATCCTTCTCCCTTGTTCTCAGTTAC

TCATCAAATGGCACTCAGTTTATATCTTTA-ATGTCAGAATTTGCGCCTATTTGTATCTATTTAGTGATC

AGTCTACTAGTTTCTTTGATCTTACTCGGTGTTCCTTTTCCATTTTCTTCTAATAGTTCGACTTATCCAG

AAAAATTGTCGGCCTACGAATGTGGTTTCGATCCTTTCGGTGATGCCAGAAGTCGTTTCGATATACGATT

TTATCTTGTTTCAATTTTATTTATTATCCTTGATCTGGAAGTCACCTTTTTCTTTCCTTGGGCAGTACCT

CTCAACAAGATTGATCCGTTTGGATTTTGGTCTATGATGGCCTTTTTATTGATTTTAACGATTGGATTTC

TCTATGAATGGAAAAGGGGTGCTTTGGATCGGGAGTA-AAAAGTGTTTATTACGATTACGCCCAACAGCC

CACTTGAGCAATTTGCCATTCTCCCATTGATTCCTATGAAAATAGGAAACTTGTATTTCTCATTCACAAA

TCCATCTTTGTTTATGCTGCTAACTCTCAGTTTGGTCCTACTTCTGCTTCATTTTGTTACTAAAAACGGA

GGAGGAAACTCAGTACCAAATGTTTGGCAATCCTTGGTAGAGCTTATTTATGATTTCGTGCTGAACCTGG

TAAACGAACAAATAGGTGGTCTTTCCGGAAATGTGAAACAAAAGTTTTTCCCTTGCATCTTGGTCACTTT

TACTTTTTTGTTATTTCGTAATCTCCAGGGTATGATACCCTATAGCTTTACAGTTACAAGTCATTTTCTC

ATTACTTTGGGTCTTTCATTTTCAATTTTTATTGGCATTACTATAGTGGGATTTCAAAGAAATGGGCTTC

ATTTTTTAAGCTTCTTATTACCTGCAGGAGTCCCACTGCCGTTAGCACCTTTTTTAGTACTCCTTGAGCT

AATCCCTCATTGTTTTCGCGCATTAAGCTCAGGAATACGTTTATTTGCTAATATGATGGCCGGTCATAGT

TCAGTAAAGATTTTAAGTGGGTTCGCTTGGACTATGCTATGTATGAATGATCTTTTATATTTCATAGGAG

ATCTTGGTCCTTTATTTATAGTTCTTGCATTAACCGGTCTTGAATTAGGTGTAGCTATATTACAAGCTCA

TGTTTTTACGATCTTAATCTGTATTTACTTGAATGATGCTACAAATCTCCATCAA---------------

----------------ATATGTGGGCACCTGATATCTATGAGGGTTCACCCACCCCGGTTACAGCATTCT

TTTCTATTGCGCCTAAAATTTCCATTTCTGCGAATATTTTACGTGTTTTTATTTATGGTTCCTATGGAGC

TACATTGCAACAAATCTTCTTTTTCTGCAGCATTGCTTCTATGATCTTAGGAGCACTGGCCGCCATGGCC

CAAACGAAAGTAAAAAGACTTCTAGCTCATAGTTCAATTGGACATGTAGGTTATATTCGTACTGGTTTCT

CATGTGGAACCATAGAAGGAATTCAATCACTACTAATTGGTCTCTTTATTTATGCATCAATGACGATAGA

TGCATTCGCTATAGTTTCAGCATTACGGCAAACCCGTGTCAAATATATAGCGGATTTGGGCGCTCTAGCC

AAAACGAATCCTATTTCGGCTATTACCTTCTCTATTACTATGTTCTCATACGCAGGAATACCCCCGTTAG

CCGGCTTTTGTAGTAAATTCTATTTGTTCTTCGCCGCTTTGGGTTGTGGGGCTTACTTCCTAGCCCCAGT

GGGAGTAGTGACTAGCGTTATAGGTTGT--GTTCGATAGCCCGACCGTAGTGATGTTAATTGTGGTTACA

TTCATAAGTAGCTTGGTCCATCTTTATTCTATTTCATATATGTCCGAGGATCCGCATAGCCCTCGATTTA

TGTGTTATTTATCCATTCTTACTTTTTTTATGCCAATGTTGGTGACTGGAGATAACTCTCTTCAATTATT

CTTGGGATGGGAGGGAGTAGGTCTTGCTTCATATTTGTTAATTCATTTTTGGTTTACACGACTTCAGGCA

GATAAAGCAGCTATAAAAGCTATGCTTGTCAATCGAGTAGGTGATTTTGGATTAGCTCTTGGGATTTCGG

GTCGTTTTACTCTCTTTCAAACAGTAGACTTTTCTACCATTTTTGCTTGTGCTAGTGCCCCTAGAAATTC

TTGGATTTTTTGCAATATGAGATTGAATGCCATAACTCTTATTTGTATTTTACTTTTTATTGGTGCTGTT

GGAAAATCTGCACAGATAGGATCGCATACTTGGTCACCCGATGCTATGGAGGGTCCCACTCCAGTATCCG

CTTTGATTCATGCAGCTACTATGGTAACAGCTGGCGTTTTCATGATAGCAAGGTGTTCCCCTTTATTTGA

ATACCCACCTACGGCTTTAATTGTTATTACTTTTGCAGGAGCTATGACGTCATTCCTTGCGGCAACCACT

GGAATATTACAGAACGATCTAAAGAGGGTCATAGCTTATTCAACTTGCAGTCAATTAGGCTATATGATCT

TTGCTTGCGGCATTTCTAACTATTCGGTTAGCGTCTTTCATTTAATGAATCACGCCTTTTTCAAAGCATT

ACTATTCCTGAGTGCAGGTTCGGTGATTCATGCCATGTCGGATGAGCAAGATATGCGGAAGATGGGGGGG

CTCGCCTCCTCGTTCCCTTTTACCTATGCCATGATGCTCATGGGCAGCTTATCTCTAATTGGATTTCCTT

TTCTAACTGGATTTTATTCCAAAGATGTGATCTTAGAGCTCGCTTACACTAAGTATACCATCAGTGGGAA

CTTTGCTTTCTGGTTGGGAAGTGTCTCTGTCCTTTTCACTTCTTATTACTCCTTTCGTTCACTTTTTCTA

ACATTTCTAGTACCAACTAATTCATTCGGGCGAGACATCTTACGATGTCATGATGCGCCCATTCCTATGG

CCATTCCTTTAATACTTCTGGCTTTCGGGAGTCTCTTTGTAGGATACTTGGCCAAA-TAACACAAAGAAG

ATACAGTTCACTCAACGATTGCCTTTGGGTTCCGAACTCCATATGGGGAAGGAACGTTGTTGTTTGCGGG

GTCTCGATCATTTACATGGACCCACTTTTCATTCCATTTGTGGGAATTTGATGATCTATAAACCGTCCTT

AACGAACGATCGGCTCATCTT------TGAGCATGATGAATCACTTCGTGCCGACCTGTTGTCAATAAAC

TTTTTGGCCTCATATGAGAATGGAAAACTGGAGCATTTTCTTCATCGGTGGATGAAGAATCGCGAACATA

ATAATTTCTGGTTAAGCATGTTCCCAGAAAAAAGATACTTTCGAGAAACAACGAGCACGACTGAAGTGGC

TATACATACAAATCCATTTACGGATCTATATGCTTCGATTGGAACTGGAAGTTCAAGAACAGGCGGCTGG

TATACTACCATAATGAAACTGCCTTTTATTTTTTTTATTCGGATAGGATTTCTGTTGGCTTCGTTGGGAG

GCTCGCGTAGTTTGTTACGTCAGCTCCAAAAGGAAAAATTGCGTTGGAATCGAGAAAGTTACGTAA----

AGTTCATAATTGTATAA-CAATTTTTGGGCCAATTCCCTCTTCGTACTACCAAAAAATGAGATTCTTGCC

GAATCCGAGTTTGCTGCTCCAACCATTACCAAACTAATACCTATTCTGTTTAGTACTTCAGGTGCTTCTG

TTGCGTATAATGTAAATCCCGTAGCGGATCAATTCC---------------AACGAGCCTTTCAAACTAG

TACTTTTTGTAATCGACTCTATAGCTTCTTCAATAAACGCTGGTTCTTCGATCAAGTTTTGAATGACTTT

CTAGTCAGATCGTTCTTGCGTTTCGGATATGAAGTCTCATTCGAAGCTTTAGACAAAGGTGCTATTGAGA

TATTGGGCCCCTATGGTATCTCGTACACATTCCGACGATTGGCCGAGCGAATAAGTCAACTTCAAAGTGG

ATTTGTTTTGTTCCATGATCTATGGGTCTACTGGAGCTACCCATTTCGATCAATTAGCCAAGATTTTGAC

CGGATACGAAATCACTGGTGTTCAATCTAGTGGTATTTTTATGGGGATTCTTTTTATCGCTGTAGGATCC

CTATTCAAGATCACTGCAGTTCCTTTT--GGTCTATGCACATTGCTTTCTCCAGGAGGTTGGCCGCCTAT

CCTAGATCTTCCCATTTCCAAGAGGATCCCGGGCTCAATCTGGTTTAGTATCAAGGTGATTCTCTTTCTC

TTTCTATATATATGGGTCCGTGCAGCATTTCCACGATATCGTTATGATCAATTAATGGGACTTGGCCGGA

AAGTGTTCTTGCCTCTATCATTAGCTCGGGTAGTCGCCGTTTCTGGTGTTTTAGTCACCTTTCAATGGCT

CCCTTAAATGCCTCAACTGGATAAATTTACTTATTTCACACAATTCTTCTGGTCATGCCTTTTCTTCTTT

ACTTTCTATATTTTAATATGCAATGATAGAGATGGAGTACTGGGGATCAGCAGAATTCTAAAACTACGAA

ACCAACTGCTTTCACACCGGGGGAACAACATCCAAA------GCAAGGAACCCAACAGTTTGGAAGATAT

CTTGAGAAAAGGTTTTCACACAGGTGTATCCTATATGTACTCTAGTTTATTCGAAGTATCCCAATGGTGT

AAGTCCGTCGACTTATTGGGAAAAAGGAAGAAAATCACTTTGATCTCTTCTTTCGGAGAAATAAGTGGCT

CGCGAGGAATGGAAAGAAACATATTTTATTTGATCTCGAAGTCTTCATATAGCACTTT------------

---TTCCAATCATGGATGGGGGATCACTTGTAAGAATGACATAATGCTAATCCATGTTCTACACGGCCAA

GGA--------------------

>Tetragonia tetragonoides MW971440.1

ATGATACTTTCTGTTTTGTCGAGCCCGGCTTTGGTCTCTGGTTTGATGGTTGTACGTGCTAAAAATCCGG

TACATTCCGTTTCGTTTCCCATCCCAGTCTTTCGCAACACTTCAGGTTTACTTCTTTTGTTAGGTCTCGA

CTTTTTCGCTATGATCTTCCCAGTAGTTTATATAGGAGCTATAGCCGTTTCATTCCTATTCGTTGTTATG

ATGTTCCATATTCAAATAGCGGAGATTCACGAAGAAGTATTGCGCTATTTACCAGTGAGTGGTATTATTG

GACTGATCTTTTGGTGGGAAATGTTCTTCATTTTAGATAATGAAACCATTCCATTACTACCAACCCAAAG

AAATACGACCTCTCTGAGATATACGGTTTATGCCGGAAAGGTACGAAGTTGGACTAATTTGGAAACATTG

GGCAATTTACTTTATACTTACTATTTTGTCTGGTTTTTGGTTTCTAGTCTTATTTTATTAGTAGCCATGA

TTGGGGCTATAGTACTGACTATGCATAGGACTACTAA------GGTGAAAAGACAGGATGTATTCCGACG

AAATGCTATTGATTTTAGAAGGACTATAATGAGGAGGACGACAGAC--ATGTCAATATATGAATTGTTTC

ATTATTCGTTATTTCCGGGGCTTTTCATTGCATTCACTTACAACAAAAAACAACCACCAGCGTTTGGTGC

AGCACCTGCATTTTGGTGTATTCTTCTTTCTTTCCTTGGTCTTTTGTTCTGTCATATTCCTAATAACTTA

TCCAATTACAACGTATTAACCGCTAATGCACCTTTCTTTTATCAAATCTCAGGGACATGGTCTAATCATG

AAGGTAGTATTTTATTATGGTGTCGGATCCCAAGTTTTTATGGATTCCTTCTTTGTTACCGGGGTCGATC

CCAAAGTCATAATGTCTCAAAACGAGGAGGCCATAGAGAAAGTCTTCTTTTTTCCTTTGTCTTAAACTTC

GTGAAGAACTCCATTCTATCTCTTCCTCGTTACGAACAAAAAAGTAGAGTTCTTCACGAACCCCAGTTGT

ACACTCTCTTCGTTCTACGAA---CTCTTGTTGATTCTGAACTTTGTTCGCGAAGGAACCGGACTTTTGA

CGGGCCAGCTCTTTTTTACGCGCCGCTTTACCCTGAAAGGAAAATGAGCTTTGCTCTTCTGGGCGCTAGG

CGCTCTCGTGGTTCGCGAGAAGGAAAAAGGACTCATCCTTTGTTGCATCTGGCACGAGATGATAAAGAGA

GAGCTTCGTCTATCGATGAACAGCGGATTGACGGAGCTCTTGGCATTGCTTTGTTTTTCTTTCCTTTCCT

ATCAGCGAGTTCCGATCCTTTTGTTCGAAATTTCTTCGTTCGTACCGAACCGCTTGCAGAATCAAATCCT

GTTCCACAAGATCCTATATTAGCTATACATCCTCCTTGCATTTATGCCGGAGACGTCGCCAGTGCTATGG

GCTTTGGCTTATGTAGATCAAAAATGATGAATGGGATTGTGGCACTCCACTCGCCACCAATGCGGAAGGA

TGTCGCCGAAAAGAATGGAACGCTGCTTTGCTCTGCTGGATGCGTCGGATCCCGTATAACAAGCGAGCTC

TTTACCCTTAAATTCAAACATGTGGGCGCCAAATGCTATCCTGCTCTATTGTTGCGTAGCAATAGAAGCC

TGCTC---ATGCTGCTTCGGCGGCGCTTTTTCGCCTTCTCTTCGCTCTGGACAAGAGCGCTAGCGGACAC

GGGGAGGGAGCG------GGCGAAGCCTTTCT------TTCGTAATGGAAAGAAAGATACCACTACTTCG

CCTCTTTGTTGGACCGCCGGCGCGAACACAGTGGTCTCTGACCAGGACCAGGAACCAATTCGAATTTGGA

TCTTGATATGTCGGTGCTTTTTAACCGTAGGCATCTTGCCAGGAAGTTGGTGGGCTCATCATGAATTAGG

TCGGGGTGGCTGGTGGTTTCGGGATCCCGTAGAAAATGCTTCTTTTATGCCTCGGGTATTAGCCACAGCT

CGTATTCATTCAGTAATTTTACCCCTTCTTCATTCTTGGACTTTGCTTCTTAATATTGTGACTTTTCTAT

GCTGTGTCTTAGGAACCTTTTCAATACGGTCCGGATTGCTAGCTTCCGTTCATAGTTTTGCTACAGATGA

TACACGAGGAATCTTTTTATGGCGGTTCTTCCTTCTAATGACCGGCATATCTATGATTCTTTTCTCTCAG

ATGAAGCAGCAGGCATCGGTCCGTAGAACCTATAAAAAAGAGATGGTTGTAGCGCGAAGTACTCTTGTGC

ACTTACG---ATGATTGTTCGAGAATGGCTATTCTTCCCAATTGCTCCTTGTGATGCAGCGGAACCATGG

CAATTAGGATTTCAAGACGCAGCAACACCTATGATGCAAGGAATAATAGACTTACATCATGATATCTTTT

TCTTCCTCATTCTGATTTTGGTTTTCGTATCATGGATCTTGGTTCGCGCTTTATGGCATTTCCACTATAA

AAAAAATCCAATCCCGCAAAGGATTGTTCATGGAACTACTATCGAGATTATTCGGACCATCTTTCCTAGT

ATCATCCTGATGTTCATTGCTATACCATCATTTGCTCTGTTATACTCAATGGACGAGGTAGTAGTAGATC

CAGCCATTACTATCAAAGCTATTGGACATCAATGGTATCGGAG---------------------------

----------------------------------------------------------------------

----------------------------------------------------------------------

----------------------------------------------------------------------

----------------------------------------------------------------------

----------------------------------------------------------------------

------------------------TGAGACGACTCTTTTTTGAACTATATCATAAACAGATCTTCTTCTC

CACACCAATCACGAGTTTTTCTCCATTCCTCTCGTATATTGTCGTAACGCCCTTAATGCTAGGTTTTGAA

AAAGACTTTTCATGTCATTTCCATTTAGGTCCGATTCGGATCCCTCCGTTGTTTCCTTTTCCTCCCGCAC

CTTTTCTTCGAAATGAGAAAGAAGATGGTACACTCGAATTGTATTATTTAAGTGCTTATTGCTTGCCAAA

GATCCTACTTCTACAATTGGTAGGTCACCGGGTTATTCAAATAAGTCGTGTTTTCTGTAGTTTTCCCATG

TTACAACTTCTGTACCAATTCGGTCAATCCGGAATGGATCGGTTAAACATTCTATTAGGGAGCCTGGTCT

TGACTCTTCTGTGTGGTATTCATTCTCGTTTGGCTCTTGGAATCACATCCAGCAGTGGTTGGAACAGCTC

GCAAAATTTAACCACTTCACCTACTTCATTGCCCTCAACCGTTTCTCGTACCTCTATTGAAACAGAATGG

TTTCATGTTCTTTCATCGATTGGTTATTTTTCTTCGTTCGTATCTCTTTTTCCAATTTCGGTCTCGATTA

GTTCACAAGATTGAATGTCCGTTTTGTTATTACAACCTTC-----TTTTTTGATGTCAAAGACCAGAAGC

TACGCGCAAATTCTCATTGGATCTTGGTTGTTCTTAACAGCGATGGCTATTCATTTAAGTCTTTGGGTAG

CACCACTAGATCTTCAACAAGGTGGAAATTCTCGTATTCTCTATGTACATGTTCCTGTGGCTCGGATGAG

TATTCTTGTTTATATTGTTACGGCTATAAACACTTTCTTGTTCCTATTAACAAAACATCCTCTTTTTCTT

CGCTCTTCCGGAACCGGTACAGAAATGGGTGCTCTTTCTACGTTGTTTACCTTAGTTACTGGGGGGTTTC

GGGGAAGACCCATGTGGGGCACCTTTTGGGTGTGGGATGCTCGTTTAACTTCTGTATTCATCTCGTTCCT

TATTTACCTGGGTGCACTGTGTTTTCAAAAGCTTCCTGTCGAACCGGCTCCTATTTCAATCCGTGCTGGA

CCGATCGATATACCAATAATCAAGTCTCCAGTCAACTGGTGGAATACATCGCATCAACCTGGGAGCATTA

GCCGATCTGGTACATCAATACATGTTCCTATGCCCATTCCAATCTTGTCTAACTTTGCTAACTTCCTCTT

CTCAACCCGTATCTTGTTTGTTCTGGAAACACGTCTTCCTATTCCATCTTTTCTCGAATCTCCTTTAACG

GAAGAAATAGAAGCTCGAGAAGGAATA-------------AAGATTCTCTTTGCTGCTATTCTCTCTATT

TGTGCATTAAGTTCGAAGAAGATCTCAATCTATAATGAAGAAATGATAGTAGCTCGTTGTTTTATAGGCT

TTATCATATTCAGTCGGAAGAGTTTAGGTAATACTTTCAAAGTGACTCTCGACGAGAGAATCCAGGCTAT

TCAGGAAGAATCGCAGCAATTCCCCAATCCTAACGAAGTAGTTCCTCCGGAATCCAATGAACAACAACGA

TTACTTAGGATCAGCTTGCGAATTTGTGGAGCCGTAGTAGAATCATTACCAATGGCACGCTGTGCGCCTA

AGTGCGAAAAGACAGTGCAAGCTTTGTTATGTCGAAACCTAAATGTTAAGTCAGCAACACTTCCAAATGC

CACTTCTTCCCGTCGCACCCGTCTTCAGGACGATCTAGTCACAGGTTTTCACTTCTCAGTGAGTGAAAGA

TTTGTCCCCGGGTCTACGTTGAAAGCTTCTATAGTAGAACTCATTCGAGAAGGCTTGGCGGTCTTAAGAA

TGGTTCGGGTGGGAGGTTCTCT---TGAAAGAGGCGATCAGAATGGTACCCGAATCCATTTACGATCCCG

AGTTTCCAGACACATCGCACTTCCGCTCGGGTCGAGGCTGCCACTCGGCCCTAAGACGGATCAAAGAAGA

GTGGGGAACCTCTCGCTGGTTTTTGGAATTCGACATCAGGAAGTGTTTTCACACCATCGACCGACATCGA

CTCATCCCAATCTTTAAGGAAGAGATCGACGATCCCAAGTTCTTTTACTCCATTCAGAAAGTCTTTTCTG

CCGGACGACTCGTAGCAGGTGAGAAGGGCCCTTACTCCGTCCCACACAGTGTACTACTATCGGCCCTACC

AGGCAACATCTACTTACACAAGCTCGATCAGGAGATAGGGAGGATCCGACAGAAGTACGAAATTCCGATT

GTTCAGAGAATCAGATCGGTTCTATTAAAGACAAGTCGTATTGATGACCAA-------------------

--ATGGAATTCTCTCCCAGAGCTGCGGAACTAACGACTCTATTAGAAAGTAGAATTACCAACTTTTACAC

GAATTTTCAAGTGGATGAGATCGGTCGAGTGGTCTCAGTTGGAGATGGGATTGCACGTGTTTATGGATTG

AACGAGATTCAAGCTGGGGAAATGGTGGAATTTGCCAGCGGTGTGAAAGGAATAGCCTTAAATCTTGAGA

ATGAGAATGTAGGGATTGTTGTCTTTGGTAGTGATACCGCTATTAAAGAAGGAGATCTTGTCAAGCGCAC

TGGATCTATTGTGGATGTTCCTGCGGGAAAGGCTATGCTAGGGCGTGTGGTCGACGCGTTGGGAGTACCT

ATTGATGGAAGAGGGGCTCTAAGCGATCACGAGCGTCGACGTGTCGAAGTGAAAGCTCCTGGGATTATTG

AACGTAAATCTGTGCACGAGCCTATGCAAACAGGGTTAAAGGCGGTAGATAGCCTGGTTCCTATAGGCCG

TGGTCAACGAGAACTTATAATCGGGGACCGACAAACGGGAAAAACAGCTATTGCTATCGATACCATATTA

AACCAAAAGCAACTGAACTCAAGGGCCACCTCTGAGAGTGAGACATTGTATTGTGTCTATGTAGCGGTTG

GACAAAAACGTTCAACTGTGGCACAATTAGTTCAAATTCTTTCAGAAGCGAATGCTTTGGAATATTCCAT

TCTTGTAGCAGCCACCGCTTCGGATCCTGCTCCTCTTCAATTTCTGGCCCCATATTCTGGGTGTGCTATG

GGAGAATATTTCCGCGATAATGGAATGCACGCATTAATAATCTATGATGATCTTAGTAAACAGGCGGTGG

CATATCGACAAATGTCATTATTGTTACGCCGACCACCAGGCCGTGAGGCTTTCCCAGGCGACGTTTTCTA

TTTACATTCTCGTCTCTTAGAAAGAGCCGCTAAACGATCGGACCAGACAGGTGCAGGTAGCTTGACCGCC

TTACCCGTCATTGAAACACAAGCTGGAGACGTATCGGCCTATATTCCCACCAATGTGATCTCCATTACTG

ATGGACAAATCTGTTCGGAAACAGAGCTCTTTTATCGCGGAATTAGACCTGCTATTAACGTCGGCTTATC

TGTCAGTCGCGTCGGGTCTGCCGCTCAGTTGAAAGCTATGAAACAAGTCTGCGGTAGTCCAAAACTGGAA

TTGGCACAATATCGCGAAGTGGCCGCCTTTGCTCAATTTGGGTCAGACCTTGATGCTGCGACTCAGGCAT

TACTCAATAGAGGTGCAAGGCTTACAGAAGTACCGAAACAACCACAATATGCACCACTTCCAATTGAAAA

ACAAATTCTAGTCATTTACGCAGCTGTCAATGGATTCTGTGATCGAATGCCACTAGATAAAATTTCTCAA

TATGAGAGAACCATTCCAAATAGTGTAAAACCTGAATTATTACAATCCCT------AAAGGGCGGCTTAG

CTAACGAAAAAAAGATGGAACTAGATGCATTCTTAAAAGAATGCGCTTTGACTTAC--------------

--------CATCCAACGCAAAGCGGCCTTTCATTCCCTTGTTTCGTCGTGGCACACCCTCCCCACAAGCA

CCCCCCGACGAAGGGGGGACCAGAAAAGGCCTTTCGTTTTCCCCCCTTCGTCGGCCCTTGCCACCTTCCT

TAACAAGCCCTCGAGCCTCCTTTTCGCTGCCTTCCTCATAGAAGCCGCCGGGTTGACCCCGAAGGCCGAA

TTCTATGGTAGAGAACGCTGTAATAATAATTGGGCCATGAGAGACCTTTTTAAGTATTGCAAAAGAAAGG

GCCTGCTGATAGAGCTGGGCGAGGCAGCGATACTAGTTATCAGGTCAGAGAAAGGCCTGGCCCGTAAGCT

GGCCCCCTTAAAAACCCATTACTTAATAAGGATTTGTTACGCGCGATATGCCGACGACTTACTATTGGGA

ATCGTGGGTGCCGTAGAGCTTCTCATAGAAATACAAAAACGTATCGCCCACTTCCTACAATCCGGCCTGA

ACCTTTGGGTAGGCTCTGCGGGATCAACAACAATAGCTGCACGGAGTACGGTAGAATTCCTCGGTACGGT

CATTCGGGAAGTCCCTCCGAAGAGGACTCCCATACAATTCTTGCGAGAGCTGGAGAAGCGTCTACGGGTA

AAGCACCGTATCCATATAACTGCTTGCCACTTACGCTCTGCCATTCATTCCAAGTTTAGGAACCTAGGTA

ATAGTATCCCGGTCAAACAGCTGACGAAGGGGATGAGCAAAACAGGGAGTCTACTGGACGCGGTTCAACT

AGCGGAAAGTCTTTCCACAGCTAGAGTAAGAAGTCCCCAAGTGAGCGTATTATGGGAGACCGTCAAGCAC

ATCCGGCAAGGATCAAGGGAGATCTCGTTGTTGCATAGCTCAGGTCAGAGCAAGGTGCCATCGGACGTTC

AACAGGCAGTCTCGCGATCGGGCATGAGTGTCCGGAAGTTGTC---ATTGTATACTCTCGCGGGTCGGAA

GGCGGCGGGGGAAGGAGGGGGACACTGGGCGAGATCTATCAGCAGCGAATTCCCCATACAGATAGAAGCG

CCTATCAAAAAGATACTCCGAAGGCTTCGAGATCGAGGTATCATTAGCCGAAGAAGACCCTGGCCAATCC

ACGTGGCCTGCTTGACGAACGTCAGCGACGGAGACATCGTAAATTGGTCCGCGGGCATCGCGATAAGTCC

TCTGTCCTACTACAGGTGCCGCGACAACCTTTACCAAGTCCGAACGATTGTCGACCACCAGATCCGCTGG

TCTGCAATATTCACCCCAGCCCACAAGCACAAATCCTCAGCGCGGAATATAATCCCAAAGTACTCCAAAG

ACTCAAATATACTAAATCAAGAAGGTGGTAAGACCCTAGCAGAGTTCCCCAACAGCATAGAGCTTGGGGA

GCTCGGACCCGGTCAAGATCC-GAACAACAAAGAGCACTCAACTACTA------------------TGGT

CCAACTACATAACTTTTTCTTTTTCATTACTTCCATGGTCGTGCCTTGTGGCACGGCAGCACCCGTACTA

TTGAAATGGTTCGTCAGTAGAGATGTTCCCACAGGTGCCCCTTTTTCCAATGGTACTATAATTCCTATTC

CTATCCCTTCATTCCTTCTTTTGGTCTATCTACATTCCAGGAAATTCATACGCTCCATGGACGGAGTCAA

AAGTGGAGTCTTGGTCAGAGCAAGCTGCCCTATTTTAT------TACCAGACATAATTGGGAGAAGCTCA

TCCGAAACTAGAGCTAGAAACGCCTTATTTCGTTTCGTTCCCATTCTTCATTTTCTTCTTCTCGAATCCA

A------GGGGGACTTCCCATATTTAGAATCTTTTTGCGGTGTGCTCCGTTTACTATTCTTTCGTACTTT

CTTCTCTTTCCCACGCGATAGGTCAGCGAAGCGTGAGCGGGCGCGGAGAAGAAAACGCCAAACACTTCGG

CCTAAC------GGGAATGAGCAACGACGAAATGACAAGATAAAGTGCCC--------------------

----------------------------------------------------------------------

----------------------------------------------------------------------

----------------------------------------------------------------------

-------------------CGTACATAGCTGTTCCAGCTGAAATACTTGGAATAATTCTACCACTTCTAC

TAGGAGTAGCCTTTTTAGTGCTAGCTGAACGTAAAGTAATGGCTTTTGTGCAACGTCGAAAGGGTCCTGA

TGTAGTGGGATCGTTCGGATTGTTACAACCTCTAGCAGATGGTTCGAAATTGATTCTAAAAGAACCTATT

TCACCAAGTAGTGCTAATTTCTCCCTTTTTAGAATGGCTCCAGTCACTACATTTATGCTAAGTCTGGTTG

CTCGGGCCGTTGTACCTTTTGATTATGGTATGGTATTGTCAGATCCGAACATAGGGCTACTTTATTTGTT

TGCCATATCTTCGCTAGGTGTTTATGGAATTATTATAGCAGGTTGGTCTAGTA-TTATTATATACGTTTA

GTGAAAAGAATGTTTTTTGATACACCTAGGACATGGATTCTATATGAACCAATGGATCGTGACAAGTCGT

TACTACTAGCAATGACTTCCTCTTTCATTACTTCATCCTTTCCATATCCTTCTCCCTTGTTCTCAGTTAC

TCATCAAATGGCACTCAGTTTATATCTTTAA-TGTCAGAATTTGCGCCTATTTGTATCTATTTAGTGATC

AGTCTGCTAGTTTCTTTGATCCCACTTGGTGTTCCTTTTCCATTTTCTTCTAATAGTTCGACTTATCCAG

AAAAATTGTCGGCCTACGAATGTGGTTTCGATCCTTTCGGTGATGCCAGAAGTCGTTTTGATATACGATT

TTATCTTGTTTCAATTTTATTTATTATCCTTGATCCGGAAGTCACCTTTTTCTTTCCTTGGGCAGTACCT

CTCAACAAGATTGATCCGTTTGGATCTTGGTCCATGATGGCCTTTTTATTGATTTTAACGATAGGATTTC

TCTATGAATGGAAAAGGGGTGCTTCGGATCGGGAGTAAAAAAGTGTTTATTACGATTACGCCCAACAGCC

CACTTGAGCAATTTGCCATTCTCCCATTGATTCCTATGAAAATAGGAAACTTGTATTTCTCATTCACAAA

TCCATCTTTGTTTATGCTGCTAACTCTCAGTTTGGTCCTACTTCTGCTTCATTTTGTTACTAAAAACGGA

GGAGGAAACTCAGTACCAAATGTTTGGCAATCCTTGGTAGAGCTTATTTATGATTTCGTGCTGAACCTGG

TAAACGAACAAATAGGTGGTCTTTCCGGAAATGTGAAACAAAAGTTTTTCCCTTGCATCTTGGTCACTTT

TACTTTTTTGTTATTTCGTAATCTTCAGGGTATGATACCCTATAGCTTTACAGTTACAAGTCATTTTCTC

ATTACTTTGGGTCTTTCATTTTCCATTTTTATTGGCATTACTATAGTGGGATTTCAAAGAAATGGGCTTC

ATTTTTTAAGCTTCTTATTACCTGCAGGAGTCCCACTGCCGTTAGCACCTTTTTTAGTACTCCTTGAGCT

AATCTCTCATTGTTTTCGCGCATTAAGCTCAGGAATACGTTTATTTGCTAATATGATGGCCGGTCATAGT

TCAGTAAAGATTTTAAGTGGGTTCGCTTGGACTATGCTATGTATGAATGATCTTTTATATTTCATAGGAG

ATCTTGGTCCTTTATTTATAGTTCTTGCATTAACCGGTCTTGAATTAGGTGTAGCTATATTACAAGCTCA

TGTTTTTACGATCTTAATCTGTATTTACTTGAATGATGCTACAAATCTCCATCAA---------------

-----------------TATGTGGGCACCTGATATCTATGAGGGTTCACCCACCCCGGTTACAGCATTCT

TTTCTATTGCGCCTAAAATTTCTATTTCTGCTAATATTTTACGTGTTTTTATTTATGGTTCCTATGGAGC

TACATTGCAACAAATCTTCTTTTTCTGCAGCATTGCTTCTATGATCTTAGGAGCACTGGCCGCCATGGCC

CAAACGAAAGTAAAAAGACTTCTAGCTCATAGTTCAATTGGACATGTAGGTTATATTCGTACTGGTTTCT

CATGTGGAACCATAGAAGGAATTCAATCACTACTAATTGGTCTCTTTATTTATGCATCAATGACGATAGA

TGCATTCGCTATAGTTTCAGCATTACGGCAAACCCGTGTCAAATATATAGCGGATTTGGGCGCTCTAGCC

AAAACGAATCCTATTTCGGCTATTACCTTCTCTATTACTATGTTCTCATACGCAGGAATACCCCCGTTAG

CCGGCTTTTGTAGTAAATTCTATTTGTTCTTCGCCGCTTTGGGTTGTGGGGCTTACTTCCTAGCCCCAGT

GGGAGTAGTGACTAGCGTTATAGGTTGTTTGTTCGATAGCCCGACCGTAGTGATGTTAATTGTGGTTACA

TTCATAAGTAGCTTGGTCCATCTTTATTCTATTTCATATATGTCTGAGGATCCGCATAGCCCTCGATTTA

TGTGTTATTTATCCATTCTTACTTTTTTTATGCCAATGTTGGTGACTGGAGATAACTCTCTTCAATTATT

CTTGGGATGGGAGGGAGTAGGTCTTGCTTCATATTTGTTAATTCATTTTTGGTTTACACGACTTCAGGCA

GATAAAGCAGCTATAAAAGCTATGCTTGTCAATCGAGTAGGTGATTTTGGATTAGCTCTTGGGATTTCGG

GTCGTTTTACTCTCTTTCAAACAGTAGACTTTTCTACCATTTTTGCTTGTGCTGGTGCCCCTAGAAATTC

TTGGATTTCTTGCAATATGAGATTGAATGCCATAACTCTTATTTGTATTTTACTTTTTATTGGTGCTGTT

GGAAAATCTGCACAGATAGGATCGCATACTTGGTCACCCGATGCTATGGAGGGTCCCACTCCAGTATCTG

CTTTGATTCATGCAGCTACTATGGTAACAGCTGGCGTTTTCATGATAGCAAGGTGTTCCCCTTTATTTGA

ATACCCACCTACGGCTTTAATTGTTATTACTTTTGCAGGAGCTATGACGTCATTCCTTGCGGCAACCACT

GGAATATTACAGAACGATCTAAAGAGGGTCATAGCTTATTCAACTTGCAGTCAATTAGGCTATATGATCT

TTGCTTGCGGCATTTCTAACTATTCGGTTAGCGTCTTTCATTTAATGAATCACGCCTTTTTCAAAGCATT

ACTATTCCTGAGTGCAGGTTCGGTGATTCATGCCATGTCGGATGAGCAAGATATGCGGAAGATGGGGGGG

CTCGCCTCCTCGTTCCCTTTTACCTATGCCATGATGCTCATGGGCAGCTTATCTCTAATTGGATTTCCTT

TTCTAACTGGATTTTATTCCAAAGATGTGATCTTAGAGCTCGCTTACACTAAGTATACCATCAGTGGGAA

CTTTGCTTTCTGGTTGGGAAGTGTCTCTGTCCTTTTCACTTCTTATTACTCCTTTCGTTCACTTTTTCTA

ACATTTCTAGTACCAACTAATTCATTCGGGCGAGACATCTTACGATGTCATGATGCGCCCATTCCTATGG

CCATTCCTTTAATACTTCTGGCTTTCGGGAGTCTCTTTGTAGGATACTTGGCCAA--TAACACAAAGAAG

ATACAGTTCACTCAACGATTGCCTTTGGGTTCCGAACTCCATATGGGGAAGGAACGTTGTTGTTTGCGGG

GTCTCGATCATTTACATGGACCCACTTTTCATTCCATTTGTGGGAATTTGATGATCTATAAACCGTCCTT

AACGAACGATCGGCTCATCTT------TGAGCATGATGAATCACTTCGTGCCGACCTGCTGTCAATAAAC

TTTTTGGCCTCATATGAGAATGGAAAACTGGAGCATTTTCTTCATCGGTGGATGAAGAATCGCGAACATA

AAAATTTCTGGTTAAGCATGTTCCCAGAAAAAAGATACTTTCGAGAAACAACGAGCACGACTGAAGTGGC

TATACATACAAATCCATTTACGGATCTATATGCTTCGATTGGAACTGGAAGTTCAAGAACAGGCGGCTGG

TATACTACCATAATGAAACTGCCTTTTCTTTTTTTTATTCGGATAGGATTTCTGTTGGCTTCGTTGGGAG

GCTCGCGTAGTTTGTTACGTCAGCTCCAAAAGGAAAAATTGCGTTGGAATCGAGAAAGTTACGTAA----

AGTTCATAATTGTATAACCAATTTTTGGGCCAATTCCCTCTTCGTACTACCAAAAAATGAGATTCTTGCC

GAATCCGAGTTTGCTGCTCCAACCATTACCAAACTAATACCTATTCTGTTTAGTACTTCAGGTGCTTCTG

TTGCGTATAATGTAAATCCCGTAGCGGATCAATTCC---------------AACGAGCCTTTCAAACTAG

TACTTTTTGTAATCGACTCTATAGCTTCTTCAATAAACGCTGGTTCTTCGATCAAGTTTTGAATGACTTT

CTAGTCAGATCGTTCTTGCGTTTCGGATATGAAGTCTCATTCGAAGCTTTAGACAAAGGTGCTATTGAGA

TATTGGGCCCCTATGGTATCTCGTACACATTCCGACGATTGGCCGAGCGAATAAGTCAACTTCAAAGTGG

ATTTGT--TGTTCCATGATCTATGGGTCTACTGGAGCTACCCATTTCGATCAATTAGCCAAGATTTTGAC

CGGATACGAAATCACTGGTGTTCGATCTAGTGGTATTTTTATGGGGATTCTTTTTATCGCTGTAGGATCC

CTATTCAAGATCACTGCAGTTCCTTTTCTGGTCTATGCACATCGCTTTTTCCAGGAGGTTGGCCGCCTAT

CCTAGATCTTCCCATTTCCAAGAGGATCCCGGGCTCAATCTGGTTTAGTATCAAGGTGATTCTCTTTCTC

TTTCTATATATATGGGTCCGTGCAGCATTTCCACGATATCGTTATGATCAATTAATGGGACTTGGCCGGA

AAGTGTTCTTGCCTCTATCATTAGCTCGGGTAGTCGCCGTTTCTGGTGTTTTAGTCACCTTTCAATGGCT

CCCTTA-ATGCCTCAACTGGATAAATTTACTTATTTCACACAATTCTTCTGGTCATGCCTTTTCTTCTTT

ACTTTCTATATTTTAATATGCAATGATAGAGATGGAGTACTTGGGATCAGCAGAATTCTAAAACTACGAA

ACCAACTGCTTTCACACCGGGGGAACAACATCCAAA------GCAAGGACCCCAACAGTTTGGAAGATAT

CTTGAGAAAAGGTTTTAACACAGGTGTATCCTATATGTACTCTAGTTTATTCGAAGTATCCCAATGGTGT

AAGTCCGTCGACTTATTGGGAAAAAGGAAGAAAATCACTTTGATCTCTTGTTTCGGAGAAATAAGTGGCT

CACGCGGAATGGAAAGAAACATATTCTATTTGATCTCGAAGTCCTCATATAGCACTTT------------

---TTCCAATCATGGATGGGGGATCACTTGTAAGAATGACATAATGCTAATCCATGTTCTACACGGCCAA

GGA--------------------

>Tribulus terrestris MK431825.1

-TGATACTTTCTGTTTTGTCGAGCCCTGCTTTGGTCTCTGGTTTGATGGTTGCACGTGCTAAAAATCCGG

TACATTCCGTTTTGTTTCCCATCCTAGTCTTTCGCAACACTTCAGGTTTACTTCTTTTGTTAGGTCTCGA

CTTCTCCGCTATGATCTTCCCAGTAGTTTATATAGGAGCTATAGCCGTTTCATTCCTATTCGTTGTTATG

ATGTTCCATATTCAAATAGCGGAGATTCACGAAGAAGTATTGCGCTATTTACCAGTGAGTGGTATTATTG

GACTGATCTTTTGGTGGGAAATGTTCTTCATTTTAGATAATGAAACCATTCCATTACTACCAACCCAAAT

AAATACGACCTCTCTGAGATATACGGTTTATGCCGGAAAGGTACGAAGTTGGACAAATTTGGAAACATTG

GGCAATTTACTTTATACCTACTATTTCGTCTGGTTTTTGGTTCCTAGTCTGATTTTATTAGTAGCCATGA

TTGGGGCTATAGTACTTACTATGCATAGGACTACGAA------GGTGAAAAGACAGGATGTATTCCGACG

AAATGCTCTTGATTTTAGGAGGACTATAATGAGGAGGACGACAGACC-ATGTCAATATATGAATTTTTTC

ATTATTCGTTATTTCCGGGTCTTTTCGTTGCATTCACTTACAACAAGAAACAACCACCAGCGTTTGGTGC

TTCACCTGCATTTTGGTGCATTCTTCTTTCTTTCCTTGGTCTTTCGTTCCGTCATATTCCTAATAACTTA

TCCAATTACAACGTATTAACCGCTAATGCACCTTTCTTTTATCAAATCTCAGGGACATGGTCTAATCATG

AGGGTAGTATTTTATCATGGTGTCGGATCTCAAGTTTTTATGGATTCCTTCTTTGTTACCGGGGTCGACC

CCAAAGCCATAATGTCTCAAAACGAGGAGGCCATAGAGAAACTCTTTTTTATTCCTTTGTCTCGAACTTC

GTGAAGAACTCCATTCTATCTCTCCCTCGTTACG------AAAGTGGGGCT------GCGCCCCAGTTGT

ACACCCCCTTCGTTCTACGAA---CCCTTGTTGATTCTGAACTTCGTTCGCGAAGGAACCGGACTTTGGA

CGGGCCAGCCCTTTTTTACGCGCCGCTTTACCCTGAAAGGAAAATTCGCTTTGCTCCTCTGGGCGCTAGG

CGCTCCCGTGGTTCGCGAGAAGGAAAAAGGACTCATCCTTTGTTGCATCTGGCACGAGATGATAAAGAGA

GAGCTTCGTCTATCGATGAACAGCGGATTGACGGAGCTCTTGGCATTGCTTTGTTTTTCTCTCCTTTCCT

ATCAGCGAGTTCCGATCCTTTTGTTCGAAATTTCTTCATTCGTACCGAACCGCTTGCAGAATCAAATCCT

GTTCCACAAGATCCTATATCAGCTATACATCCTCCTTGCATTTATGCCGGAGACGTCGCCAGTGCTATGG

GCTTTGGCTTATGTAGATCAAAAATGATGAATGGGATTGTGGCACTCCACTCGCCGCCAATGCGGAAGGA

TGCCGCCGAAAAGAATGGAACGCTGCTTCGCTCTGCTGGATGCGTCGGATCCCGTATAACAAGCGAGCTT

TTTACCCTCAAATTCAAACATGTGGGCGCAAAATGCTATCCTGCTCTATTGTTGCGTAGCAATAGAAGCC

TGCTC---ATGCTGTTTCGGCGGCGCTTTTTCGCCTTCTCTTCGCTCTGGACAGGAGCGCTAGTGGACAC

GGGGAGGGAGCA------GGCGAAGCGTGTCG------TTCGTAATGGAAAGAAAGAGACCACTACGAAG

CCTCTTTGTTGGACCGCCGGCGCGAACACAGTGGTCTCTGACCAGGACCAGGAACCAATTCGAATTTGGA

TCTTGACATGTCGGTGGTTTTTAACCGTAGGCATCTTGCCAGGAAGTTGGTGGGCTCATCATGAATTAGG

TCGGGGTGGCTGGTGGTTTCGGGATCCCGTAGAAAATGCTTCTTTTATGCCTCGGGTATTAGCCACAGCT

TGTATTCATTCAGTAATTCTACCCCTTCTTCATTCTTGGACCTCGTTTCTTAATATTGTTACTTTTCCAT

GCTGTGTCTCAGGAACCTTTTCAATACGGTCCGGATTGCTAGCTCCCGTTCATAGTTTCGCTACAGATGA

TACACGAGGAATCTTTTTATGGCGGTTCTTCCTTCTAATGACCGGCATATCTATGATTCTTTTCTCCCAG

ATTAAGCAGCAGGCATCGGTCCGTAAAACCTATAAAAAAGAGATGGTTGTGGCGCGAAGTACTCTTGTGC

ATCTACG-----------------ATGGCTATTCATCACGATTTCTCCTTGTGATGCAGCGGAACCATGG

CAATTAGGATCTCAAGACGCAGCAACGCCTATGATGCAAGGAATAATGGACTTACATCACGATATCTTTT

TCTTCCTCATTCTGATTTTGGTTTTCGTTTCATGGATCTTGGTTCGCGCTTTATGGCATTTCCACTATCA

AAAAAATCCAATCCCGCAAAGGATTGTTCATGGAACTACTATCGAGATTCTTCGGACCATATTTCCTAGT

ATCATCCCGATGTTCATTGCTATACCATCATTTGCTCTTTTATACTCAATGGACGAGGTAGTAGTAGATC

CAGCCATTACTATCAAAGCTATTGGACATCAATGGTATCGGACTTACGAGTATTCGGACTATAACAGTTC

CGATGAACAGTCACTCACTTTTGACAGTTATACGATTCCAGAAGAAGATCTAGAACTGGGTCAATCACGT

TTATTAGAAGTGGACAATAGAGTGGTTGTACCGGCAAAAACTCATCTACGTATTATTGTCACATCTGCTG

ATGTACCTCATAGTTGGGCTGTACCTTCTTCAGGTGTCAAATGTGATGCTGTACCTGGTCGTTTAAATCA

GATCTCTATTTCGGTACAACGAGAAGGAGTTTACTATGGTCAGTGCAGTGAGATTTGTGGAACTAATCAT

GCCTTTACGCG-----------------------------------------------------------

-----------------------ATGAGACGACTCTTTCTTGAACTATATCATAAACAGATCTTTCCCTC

CACACCAATCACGAGTTTTTCTTCATTCCTCTCGTATATCGTCGTCACGCCCTTAATGCTAGGTTTTGAA

AAAGACTTTTCATGTCATTCCCATTTAGGTCCGATTCGGATCCCTCCGTTGTTTCCTTTTCCTCCCGCAC

CTTTTCCTCGAAATGAGAAAGAAGATGGTACACTCGAATTGTATTATTTAAGTGCTTATTGCTTGCCAAA

GATCCTACTTCTACAATTGGTAGGTCACTGGGTTATTCAAATAAGTCGTGTTTTCTGTGGTTTTCCCATG

TTACAACTTCCGTACCAATTCGGTCGATCCGGAATGGATCGGTTAAACATTCCATTAGGGAGCCTGGTCT

TGACTCTTCTGTGTGGTATTCATTCTTGTTCGGCTCTTGGAATCACATCCAGCAGTGGTTGGAACAGCTC

GCAAAATCCAACCACTTCACCTACTTCATTGCCCCCAACCGTTTTTCGTACCTCTATTGAAACAGAATGG

TTTCATGTTCCTTCATCGATTGGTTATTCCTCTCCGTTTGTATCTCTTTTTCCAATTTCGGTCTCGATGA

GTTTACAAGATTG--TGTCCGTTTCGTTATTACAACCTTT-----TTTTTTGATGGCAAAGACCAGAAGC

TACGCGCAAATTCTCATTGGATCTCGGTTTTTCTTAACAGCGATGGCTATTCATTTAAGTCTTCGGGTAG

CACCACTAGATCTTCAACAAGGTGGAAATTCTCGTATTCTGTATGTACATGTTCCTGCGGCTCGGATGAG

TATTCTTGTTTATATCGCTACGGCTATAAACACTTTCTTTTTCCTATTAACAAAACATCCCCTTTTTCTT

CGCTCTTCCGGAACCGGTACAGAAATGGGTGCTTTTTTTACGTTTTTTACCTTAGTTACTGGGGGGTTTC

GGGGAAGACCTATGTGGGGCACCTTTTGGGTGTGGGATGCTCGTTTAACCTCTGTATTCATCTCGTTTCT

TATTTACCTGGGTGCACTGTGTTTTCAAAAGCTTCCTGTCGAACCGGCTTCTATTTCAATTCGTGCTGGA

CCGATCGATATACCAATAATCAAGTCTTCAGTCAACTGGTGGAATACATCGCATCAACCTGGGAGCATTA

GCCGATCTGGTACATCAATACATGTTCCTATGCCCATTCCAATCTTGTCTAACTTTGCTAACTCCCCCTT

CTCAACCCGTATCTTGTTTGTTTTGGAAACACGTCTTCCTATTCCATCTTTTCCCGAATCTCCTTTAACG

GAAGAAATAGAAGCTCGAGAAGGAATACC-GCAGGCTAGAACGATGCTATTTGCTGCTATTCTATCTATT

TGTGCATTAAGTTCGAAGAAGATCTCAATCTATAATGAAGAAATGATAGTAGCTCGTTGTTTTATAGGCT

TTATCATATTAAGTCGGAAGAGTTTAGGTAATACTATCAAAGTGACTCTCGACGGGAGAATCCAGGCTAT

TCAGGAAGAATCGCAGCAATTCCCCAATCCTAACGAAGTAGTTCCTCCGGAATCCAATGAACAACAAAAA

TTACTTAGGATCAGCTTGCGAATTTGTGGCGCCGTAGTAGAATCATTACCAATGGCACGCTGTGCGCCTA

AGTGCGAAAAGACAGTGCAAGCTTTGTTATGCCGAAACCTAAATGTGAAGTCAGCAACACTTCCAAATGC

CACTTCTTCCCGTCGCATCCGTCTTCAGGACGATCTAGTCACAGGTTTTCACTTCTCAGTGAGTGAAAGA

TTTTTTCCCGGGTGTACGTTGAAAGCTTCTATAGTAGAACTCATTCGAGAGGGCTTGGTGGTCTTAAAAA

TGGTTCGGGTGGGGGGTTCTCTT-ATGAAAGAGGCCATCAGAATGGTACTCGAATCCATTTACGATCCTG

AGTTTCCAGACACATCGCACTTCCGCTCGGGTCGAGGCTGCCACTCGGCCCTAAGACGGATCAAAGAAGA

GTGGGGAACCTCTCGCTGGTTTTTGGAATTCGACATCAGGAAGTGTTTTCACACCATCGACCGACATCGA

CTCATCCCAATCTTTAAGGAAGAGATCGACGATCCCAAGTTCTTTTACTCCATTCAGAAAGTCTTTTCCG

CCGGACGACTCGTAGGAGGTGAGAAGAGCCCTGACTCCGTCCCACACAGTGTACTATTATCGGCCCTACC

AGGCAACATCTACCTACACAAGCTCGATCAGGAGATAGGGAGGATCCGACAGAAGTACGAAATTCCGATT

GTTCAGAGAATCAGATCGGTTCTATTAAGGACAGGTCGTCTTTATGACCAAGAAAACTCTGGAGAAGAAG

C-ATGGAATTCACCACCAGAGCTGCGGAACTAACGACTCTATTAGAAAGTCGAATTACCAACTTTTACAC

GAATTTTCAAGTGGATGAGATCGGTCGAGTGATTTCAGTTGGAGATGGGATTGCGCGTGTTCATGGATTG

AACGAGATTCAAGCTGGGGAGATGGTTGAATTTGCCAGCGGTGTGAAAGGAATAGCGTTAAATCTTGAGA

ATGAGAATGTAGGGATTGTTGTCTTTGGTAGTGATACCGCTATTAAAGAAGGAGATCTTGTCAAGCGCAC

TGGATCTATTGTGGATGTTCCCGCGGGAAAGGCTATGCTAGGGCGTGTGGTCGACGCGTTAGGAGTACCT

ATTGATGGAAGAGGGGCTCTCAGCGATCACGAGCGAAGACGCGTCGAAGTGAAAGCCCCTGGGATTATTG

AACGTAAATCTGTGCACGAGCCTATGCAAACAGGGTTAAAAGCGGTAGATAGCCTGGTTCCTATAGGCCG

TGGTCAACGAGAACTTATAATCGGGGACCGACAAACTGGAAAAACAGCTATTGCTATCGATACCATATTA

AACCAAAAGCAACTGAACTCAAGGGCCACCTCTGAGAGTGAGAAATTGTATTGTATCTATGTAGCGATTG

GACAGAAACGCTCAACTGTGGCACAATTAGTTCAAATTCTTTCAGAAGCGAATGCTTTGGAATATTCCAT

TCTTGTAGCAGCCACCGCTTCGGATCCTGCTCCTCTGCAATTTCTGGCCCCATATTCTGGGTGTGCCATG

GGGGAATATTTCCGCGATAATGGAATGCACGCATTAATCATCTATGATGATCTTAGTAAACAGGCGGTGG

CATATCGACAAATGTCATTATTGTTACGCCGACCACCAGGCCGTGAGGCTTTCCCGGGTGATGTTTTCTA

TTTACATTCCCGTCTCTTAGAAAGAGCCGCTAAACGATCGGACCAGACAGGTGCAGGTAGCTTGACCGCC

TTACCCGTCATTGAAACACAAGCTGGAGACGTATCGGCCTATATTCCCACCAATGTGATCCCCATTACTG

ATGGACAAATCTGTTTGGAAACAGAGCTCTTTTATCGCGGAATTAGACCTGCTATTAACGTCGGCTTATC

TGTCAGTCGCGTCGGGTCTGCCGCTCAGTTGAAAGCTATGAAACAAGTCTGCGGTAGTTCAAAACTGGAA

TTGGCACAATATCGCGAAGTGGCCGCCCTTGCTCAATTTGGGTCAGACCTTGATGCTGCGACTCAGGCAT

TACTCAATAGAGGTGCAAGGCTGACAGAAGTACCGAAACAACCACAATATGCACCACTTCCAATTGAAAA

ACAAATTCTAGTCATTTATGCAGCTGTCAATGGATTCTGTGATCGAATGCCACTAGACAAAATTTCTCAA

TATGAGAGAGCCATTCCAAGTAGTATCAAACCAGAATTACTACAATCCCTTGTAGAGAAAGGTGGCTTAA

CTAACGAAAGAAAGATAGAACCAGATTCATTCTTAAAAGAAAGCGCCTTGGAATACCAAATAGC------

-------GCATCCAACGCAAAGCGGCCTTTCATTCCCTTGTTTCGTCGTGGCACACCCCCCCCACAAGCA

CCCCCCGGCTCTGGGGGGACCAGAAAACGCCTTTCGTTTTCCCCCCTTCGTCGGCCCTTGCCGCCTTCCT

TAACAAGCCCTCGAGCCTCCTTTGCGCCGCCTTCCTCATAGAAGCCGCCGGATTGACCCCGAAGGCCGAA

TTCTATGGTAGAGAACGCTGTAATAATAATTGGGCCATGAGAGACCTTATTCAGTATTGCAAAAGAAAGG

GCCTGCTGATAGAGCTGGGCGGGGAGGCGCTACTAGTTATCAGGTCAGAGAGACGCCTGGCCCGTAAGCT

GGCCCCCTTCAAAACCCATTACTTAATAAGGATTTGTTACACGCGATATGCCGACGACTTACTACTGGGA

ATCGTGGGTGCTGTAGAGCTTCTCATAGAAATCCAAAAAGGTATCGCCCACTTCCTAAAATCCGGCCTTC

ACCTTTGGGTAGGATCCGCAGGATCAACAACAATAGCTGCACGGAGTACGGTAGAATTCCTCGGTACGGT

CATTCGGGAAGTCCCTCCGAGGACGACTCCCATACAATTCTTGCGAGAGCTGGAGAAGCGTCTACGGGTA

AAGCACCGTATCCATATAACTGCTTGCCACCTACGCTCCGCCATCCATTCAAAGTTGAGCAACCTAGGTA

ATAGTATCCCGATCAAACAGCTGACGAAGGGGATGAGCGAAAAAGGGAGTCTACTGGACGGGGTTCAACT

AGCGGAGACTCTTGGAACAGCTGGAGTAATAATTCCCCAAGTGAGCGTCTTATGGGGGACCGTCAAGCAC

ATCCGGCAAGGATCAAGGGGGATCTCGTTGTTGCATAACTCAGGTCGGAGCAACGCGCCATCGGACGTTC

AACAGGCAGTCTCACGATCGGGCATGAGTGTCCGGAAGTTGTC---ATTGTATACTCCCGCGGGTCGGAA

GGCGGCGGGGGAAGGA---GGACACTGGGCGGGATCTATCAGCAGCGAATTCTCCATACAGATGGAGGCG

CCTATCAAAAAGATACTCCGAAGGCTTCGGGATCGAGGTATAATTAGCCGACGAAGACCCTGGCCAATCC

ACGTGGCCTGCTTGACGAACGTCAGCGACGGAGACATCGTAAATTGGTCCGCGGGCATCGCGATAAGTCC

TCTGTCCTACTACAGGTGCCGCGACAACCTTTACCAAGTCCGAACGATTGTCGACCACCAGATCCGCTGG

TCTGCAATATTCACCCTAGCCCACAAGCACAAATCCTCGGCGCGGAATATAATCCCAAAGTACTCCAAAG

ACTCACATATAGTCAATCAAGAAGGTGGTCAGACCCTTGCAGAGTTTCCCAACAGCATAGAGCTTGGGAA

GCTCGGACCCGGTCAAGATCCAAAAAAAAAAGGAGCACTCAACTACT-------------------TGGT

CCAACTACATAACTTTTTCTTTTTCATTACTTCCATGGTCGTGCCTCGTGGCACGGCAGCACCCGTACTA

TTGAAATGGTTCGTCAGTAGAGATGTTCCCACAGGTGCCCCTTCTTCCAATGGTACTATAATTCCTATTC

CTATCTCTTCATTCCCTTTTTTGGTCTATCTACATTCCAGGAAATTCATACGCTCCATGGACAGAGCAAA

AAGTGGAGTCTTGGTCAGAGCAAGCCGCCCTCTTCTAT------TACCAGACATAATTGGGAGAAGCTCA

TCCGAAACTAGAGCTAGAAACGCTTCATTTCGTTTCGTTCCCGTTCTTCATTTCCTTCTTCTCGAATCCA

A------GGGGGACTTCTCATATTTAGAATCTTTCTGCGGTGTGCTCTGTTTACTATTCTTTCGTACTCT

CTTCTCTTTACCACGCGATAGGTCAGCGAAGCGTGAGCGGGCGCGGAGAAGGAAAGGCCAACAACTTCGG

AGAAAG------GAGAATGAGAAACGACGAAATGACAAGATGAGGTGCTCCGGGCACCCCCATT------

TCGA---AAGAAGGGTCGAAGGTTTTTGGCCTTTAGCTTTCCCCGTCCCCCCTTCGTCGGGTGGTGCTTG

TGTGGGGGGTGTGCCACCAGAAATCGGGCTTGAAGCTCTCGCCTTACCAATGAGCCGACAGCTGATAGCT

GTTGGTCACGACTACTACCAAAAAGCTCCAATGAAGATGAATATTTCACATGGAGGAGTGTGCATCTGTA

TGTTGGGTGTTCTTCTGT-CGTACATAGCTGTTCCAGCTGAAATACTTGGAATAATTCTACCACTTCTAC

TAGGAGTAGCCTTTTTAGTGCTAGCTGAACGTAAAGTAATGGCTTTTGTGCAACGTCGAAAGGGTCCTGA

TGTAGTGGGATCGTTCGGATTGTTACAACCTCTAGCAGATGGTTCGAAATTGATTCTAAAAGAACCTATT

TCACCAAGTAGTGCTAATTTCTCCCTTTTTAGAATGGCTCCAGTGGTTACATTTATGTTAAGTCTGGTCG

CTCGGGCCGTTGTACCTTTTGATTATGGTATGGTATTGTCAGATCTGAACATAGGGCTACTTTATTTGTT

TGCCATATCTTCGCTAGGTGTTTATGGAATTATTATAGCAGGTCGGTCTAGTATTTATTATATACGCTTA

GCGAAAAGAATGTTTTTTGATACACCTAGGACATGGATTCTATATGAACCAATGGATCGTGACAAGTCGT

TACTACTAGCAATGACTTCCTCTTTCATTACTTCATCCTTTCCATATCCCTCTCCCTTGTTCTCAGTTAC

TCATCAAATGGCACTCAGTTCATATCTTTA-ATGTCAGAATTTGCACCTATTGTTATCTATTTAGTGATC

AGTTCGCTAGTTTCTTTGATCCCACTCGGTGTTCCTTTTCTATTTTCTTCCAATAGTTCGACCTATCCAG

AAAAATTGTCGGCCTACGAATGTGGTTTCGATCCTTCCGGTGATGCCAGAAGTCGTTTTGATATACGATT

TTATCTTGTTTCAATTTTATTTATTATCCCTGATCCGGAAGTCACCTTTTTCTTTCCTTGGGCAGTACCT

CCCAACAAGATTGATCTGTTTGGATCTTGGTCCATGATGGCCTTTTTATTGATTTTGACGATAGGATCTC

TCTATGAATGGAAAAGGGGTGCTTCGGATCGGGAGTA-------------------------------CC

CACTTGATCAATTTTCCATTCTCCCATTGATTCCTATGAATATAGGAAACTTGTATTTCTCATTCACAAA

TCCATCTTTGTTTATGCTGCTAACTCTCAGTTTTGTCCTACTTCTGGTTCATTTTGTTACGAAAAAGGGA

GGAGGAAACTTAGTACCAAATGCTTGGCAATCCTTGGTAGAGCTTATTCATGATTTCGTGCTGAACCCGG

TAAACGAACAAATAGGTGGTCTTTCCGGAAATGTGAAACAAAAGTTTTTCCCTTGCATCTCGGTCACTTT

TACTTTTTCGTTATTTCGTAATCCCCAGGGTATGATACCTTATAGCTTCACAGTTACAAGTCATTTTCTC

ATTACTTTGGGTCTCTCATTTTCGATTTTTATTGGCATTACTATAGTGGGATTTCAAAAAAATGGGCTTC

ATTTTTTAAGCTTCTCATTACCCGCGGGAGTCCCACTGCCGTTAGCACCTTTTTTAGTACTCCTTGAGCT

AATCCCTCATTGTTTTCGCGCATTAAGCTCAGGAATACGTTTATTTGCTAATATGATGGCCGGTCATAGT

TCAGTAAAGATTTTAAG-----------------------------------------------------

----------------------------------------------------------------------

----------------------------------------------------------------------

----------------ATATGTGGGCACCTGATATCTATGAGGGTTCACCCACCCCGGTGACAGCATTCC

TTTCTATTGCGCCTAAAATATCTATTTCTGCTAATATTTCACGTGTTTCTATTTATGGTTCCTATGGAGC

TACATTGCAACAAATCTTCTTTTTCTGCAGCATTGCTTCTATGATCTTAGGAGCACTGGCCGCCATGGCC

CAAACGAAAGTAAAAAGACCTCTAGCTCATAGTTCAATTGGACATGTAGGTTATATTCGTACTGGTTTCT

CATGTGGAACCATAGAAGGAATTCAATCACTACTCATTGGTATCTTTATTTATGCATTAATGACGATAGA

TGCATTCGCCATAGTTTCAGCATTACGGCAAACCCGTGTCAAATATATAGCGGATTTGGGCGCTCTAGCC

AAAACGAATCCTATTTCGGCTATTACCTTCTCCATTACTATGTTCTCATACGCAGGAATACCCCCGTTAG

CCGGCTTTTGTAGCAAATTCTATTTGTTTTTCGCCGCTTTGGGTTGTGGGGCTTACTTCCTAGCCCCAGT

GGGAGTAGTGACTAGCGTTATAGGTCGT--GTTCGATAGCCCGACCGTAGTGATGTTAATTGTGGTTACA

TCCATAAGTAGCTTGGTCCATCTTTATTCCATTTCATATATGTCTGAGGATCCGCATAGCCCTCGATTTA

TGTGTTATTTATCCATTCTTACTTTTTTTATGCCAATGTTGGTGACTGGAGATAACTCTCTTCAATTATT

CCTGGGATGGGAGGGAGTAGGTCTTGCTTCATATTTGTTAATTCATTTCTGGTTTACACGACTTCAGGCA

GATAAAGCAGCTACAAAAGCTATGCCTGTCAATCGAGTAGGTGATTTTGGATTAGCTCCTGGGATTTCGG

GTCGTTTTACTCTCTTTCAAACAGTAGACTTTTCCACCATTTTTGCTCGTGCTAGTGCCCCCAGAAATTC

TTGGATTTCTTGCAATATGAGATTGAATGCCATCACTCTGATTTGTATTTTACTTCTTATTGGTGCTGTT

GGGAAATCTGCACAGATAGGATCGCATACTTGGTCACCCGATGCTATGGAGGGTCCCACTCCAGTATCCG

CTTCGATTCATGCAGCTACTATGGTAACAGCTGGCGTTTTCATGATAGCAAGGTGCTCCCCTTTATTTGA

ATACCCACCTACGGCTTTGATTGTGATTACTTCTGCAGGAGCTATGACGTCATTCCTTGCGGCAACCACT

GGAATATTACAGAACGATCTCAAGAGGGTCATAGCTTATTCAACTTGCAGTCAATTAGGCTATATGATCT

TTGCTTGCGGCATCTCTAACTATTCGGTTAGCGTCTTTCACTTAATGAATCACGCGTTTTTCAAAGCATT

ACTCTTCCTGAGTGCGGGTTCGGTGATTCATGCCATGTCGGATGAGCAAGATATGCGGAAGATGGGGGGG

CTTGCCTCCTCGTTCCCTTTGACCTATGCCATGATGCTCATAGGCAGCTTATCTCTAATTGGATTTCCTT

TTCTAACTGGATTTTATTCCAAGGATGTGATCTTAGAGCTCGCTTACACTAAGTATACCATCAGTGGGAA

CTTTGCTTTCTGGTTGGGAAGTGTCTCTGTCCTGTTCACTTCTTATTACTCTTTTCGTTCACTTTTTCTA

ACATTTCTAGTACCAACTAATTCATTCGGGCGAGACATCTTACGATGTCATGATGCGCCCATTCCTATGG

CCATTCCTTCAATACTTCTGGCTCTCGGGAGTCTCTTTGTAGGATACTTGGCCAAA-TAACACAAAGAAG

AAACAGTTCACTCAACGATTGCCTTTGGGTTCCGAACTCCATATGGGGAAGGAGCGTTGTTGTTTGCGAG

GTCTCGATCATTTACATGGACCCACTTCTCATTCTATTTGTGGTAATTTGATGATCTATAAACCGTCCCT

AACGAACGATCGGCTCATGTT------TGAGCATGATGAATCACTTCGTGCCGACCTGTTGCCAATCAAC

TTTCCGGCCTCATATGAGAATGGAAAACTGGAGCATTTTCTGCATCGGTGGATGAAGAATCGCGAACATA

ATAATTTCTGGTTGACCATGTTCCCAGAAAAAAGATACTTTCGAGAAACGACAAGCACGACTGAAGTGGC

TATACATACAAATCTATTTACGGATCTATATGCTTCGATTGGAACTGGAAGTTCCAGAACAGGCGGCTGG

TATACCACCATAATGAAACTGCCTTTTATTTTTTTTATTCGGATAGGATTTATGTTGGCTTCGTTGGGAG

GCTCGCGTAGTTTGTTACGTCAGCTCCAAAAGGATAAGTTGCGTTGGAATTGAAAAAGTTCCGTGG----

AGTTCATAATTGCATAA-CAATTTTTGGGCTAATTCCCCCTTCGTACTACCAAAAAATGAGATTCTTGCC

GAATCCGAGTTTGCTGCTCCAACCATTACCAAACTAATACCTATTCTGTTTAGTACTTCAGGTGCTTCTG

TTGCGTATAATGTAAATCCCGTAGCGGATCAATTCC---------------AACGAGCCTTTCAAACTAG

TCTTTTTTGTAATCGACTCTATAGCTTCTTCAATAAACGCTGGTTCTTCGATCAAGTTTTGAATGACTTT

CTAGTCAGATCGTTCCTGCGTTTCGGATATGAAGTCTCATTCGAAGCTTTAGACAAAGGTGCTATTGAGA

TATTGGGCCCTTATGGTATCTCGTACACATTCCGACGATTGGCCGAGCGAATAAGTCAACTTCAAAGTGG

ATTTGTTTTGTTCCATGATCTATGGGTCTACTGGAGCTACCCACTTCGATCAATTAGCCAAGATTTTGAC

CGGATACGAAATCACTGGTGCTCGATCTAGTGGTATTTTTATGGGGATTCTCTTTATCGCTGTAGGATTC

CTATTCAAGATCACTGCAGTTCCTTTT--GGTCCATGCACATCGCTCTCTCCAGGAGGTTGGCCGCCTAT

CCTAGATCTTCCCATTTTCAATAAGATCCCGGGCTCGATCCGGTTTAGTATCAAGGTGATTCTTTTTCTG

TTTCTATATATATGGGTCCGTGCAGCATTTCCACGATATCGTTATGATCAATTAATGGGACTTGGCCGGA

AAGTGTTCTTGCCTCTATCATTAGCTCGGGTAGTCCCCGTTTCTGGTGTTTCAGTCACCTTTCAATGGCT

CCCTTAAATGCCTCAACTGGATAAATTTACTTATTTCACACAATTCTTCTGGTCATGTCTTTTCCTCTTT

ACTTTCTATATTCCCATATGCAATAATGGAGATGGAGTAATTGGGATCAGCAGAATTCTCAAACTACGGA

ACCAACTGGTTTCACACCGGGGGAAAAACATCCGAA------GCAACGACCCCAAAAGTTTTGAAGATAT

CTTGAGAAAAGGTTTTAGCACCGGTGTATCCTATATGTACTCTAGTTTATTCGAAGTATCCAAATGGTGT

AACGCCGTCGACTTATTGGGAAAAAAGAGGAAAATCACTTTGATCTCTTGTTTCGGAGAAATCAGTGGTT

CACGAGGAATGGAAAAAAACATATTATATTTGATCTCGAAGTCCTCATATAGCACTTC------------

---TTCCAATCCTGGATGGGGGAAAACTTGTAGGAATGACATCATGCTCATCCATGTTCCACACGGCCAA

GGAAACATCGTTT----------

>Diospyros oleifera NC065039.1

-TGATACTTTCTGTTTTGTCAAGCCCTGCTTTGGTCTCTGGTTTGATGGTTGTACGTGCTAAAAATCCGG

TACATTCTGTTTCGTTTCCCATCCTAGTCTTTCGCAACACTTCAGGTTTACTTCTTTTGTTAGGTCTCGA

CTTTTTCGCTATGATCTTCCCAGTAGTTCATATAGGAGCGATAGCCGTTTCATTCCTATTCGTTGTTATG

ATGTTCCATATTCAAATAGCGGAGATTCACGAAGAAGTATTGCGCTATTTACCAGTGAGTGGTATTATTG

GACTGATCTTTTGGTGGGAAATTTTCTTCATTTTAGATAATGAAAGCATTCCATTACTACCAACCCAAAG

AAATACGACCTCTCTGAGATATACGGTTTATGCCAGAAAGGTACGAAGTTGGACTAATTTGGAAACATTG

GGCAATTTACTTTATACCTACTATTTCGTCTGGTTTTTGGTTCCTAGTCTTATTTTATTAGTAGCCATGA

TTGGGGCTATAGTACTGACTATGCATAGGACTACGAA------GGTGAAAAGACAGGATGTATTCCGACG

AAATGCTATTGATTCTAGGAGGACTATAATGAGGAGGACGACAGACC-ATGTCAATATATGAATTGTTTC

ATTATTCGTTATTTCCGGGTCTTTTCGTTGCATTCACTTACAACAAGAAACAACCACCAGTGTTTGGTGC

AGCACCTGCATTTTGGTGCATTCTTCTTTCTTTCCTTGGTCTTTCGTTCCGTCATATTCCTAATAACTTA

TCCAATTACAACGTATTAACTGCTAATGCACCTTTCTTTTATCAAATCTCAGGGACATGGTCTAATCATG

AGGGTAGTATTTTATCATGGTGTCGGATCCTAAGTTTTTATGGATTTCTTCTTTGTTACCGGGGTCGACC

CCAAAGCCATAATGTCTCAAAACGAGGAGGCCATAGAGAAACTCTTTTTTATTCCTTTGTCTTGAACTTC

GTGAAGAACTCCATTCTATCTCTCCCTCGTTACGAACAAAAAAGTGGGGCT------GCGCCCCAGTTGT

ACACTCCCTTCGTTCTACGAA---CCCTTGTTGATTCTGAACTTCGTTCGCGAAGGAACCGGACTTTTGA

CGGGCCAGCCCTTTTTTATGCGCCGCTTTACCCTGAAAGGAAAATGAGCTTTGCTCCTCTGGGCGCTAGG

CGCTCCCGTGGTTCGCGAGAAGGAAAAAGGGCTCATCCTTTGTTGCATCTGGCACGAGATGATAAAGAGA

GAGCTTTGTCTATCGATGAACAGCGGATTGACGGAGCTCTTGGCATTGCTTTGTTTTTCTCTCCTTTCCT

ATCAGCGAGTTCCGATCCTTTTGTTCGAAATTTCTTCGTTCGTACCGAACCGCTTGCAGAATCAAATCCT

GTTCCACAAGATCCTATATCAGCTATACATCCTCCTTGCATTTATGCCGGAGACGTCGCCAGTGCTATGG

GCTTTGGCTTATGTAGATCAAAAATGATGAATGGGATTGTGGCACTCCACTCGCCGCCAATGCGGAAGGA

TGCTGCCGAAAAGAATGGAACGCTGTTTCGCTCTGCTGGATGCGTCGGATCCCGTATAACAAGCGAGCTC

TTTACTCTCAAATTCAAACATGTGAGCGCAAAATGCTATCCTGCTCTATTCTTACGTAGCAATAGAAGCC

TGCTT---ATGCTGCTTCGGCGGCGCTTTTTCGCCTTCTCTTCGCTCTGGACAGGAGCGCTAGTGGACGC

GGGGAGGGAGCA------GGCGAAGCCTGTCG------TTCGTAATGGAAAGAAAGATACCACTACTTCG

CCTCTTTGTTGGACCGCCGGCGCGAACACAGTGGCCTCTGACCAGGACCAGGAACCAATTCGAATTTGGA

TCTTGACATGTCGGTGGTTTTTAACCGTAGGCATCTTGCCAGGAAGTTGGTGGGCTCATCATGAATTAGG

TCGGGGTGGCTGGTGGTTTCGGGATCCCGTAGAAAATGCTTCTTTTATGCCTCGGGTATTAGCCACAGCT

CGTATTCATTCAGTAATTCTACCCCTTCTTCATTCTTGGACCTCGCCTCTTAATATTGTGACTCTTCCAT

GCTGTGTCTCAGGAACCTCTTCAATACGGTCCGGATTGCTAGCTCCCGTTCATAGTTTTGCTACAGATGA

TACACGAGGAATCTTTTTATGGCAGTTCTTCCTTCTAATGACCGGCATATCTATGATTCTTTTCTCCCAG

ATGAAGCAGCAGGCATCGGTCCGTAGAACCTATAAAAAAGAGATGGTTGTGGCGCGAAGTACTCTTGTGC

ACCTACG-----------------ATGGCTATTCCTCACAATTGCTCCTTGTGATGCAGCAGAACCATGG

CAATTAGGATCTCAAGACGCAGCAACACCTATGATGCAAGGAATAATAGACTTACATCACGATATCTTTT

TCTTCCTCATTCTGATTTTGGTTTTCGTATCACGGATCTTGGTTCGCGCTTTATGGCATTTCCACTATAA

AAAAAATCCAATCCCGCAAAGGATTGTTCATGGAACTACTATCGAGATTCTTCGGACCATATTTCCTAGT

ATCATCCCGATGTTCATTGCTATACCATCATTTGCTCTGTTATACTCAATGGACGAGGTAGTAGTAGATC

CAGCCATTACTATCAAAGCTATTGGACATCAATGGTATCGGAG---------------------------

----------------------------------------------------------------------

----------------------------------------------------------------------

----------------------------------------------------------------------

----------------------------------------------------------------------

----------------------------------------------------------------------

-----------------------ATGAGACGACTCTTTCTTGAACTATATCATAAACAGATCTTCCCCTC

CACACCAATCACGAGTTTTTCTCCATTCCTCTCGTATATCGTCGTAACCCCCTTAATGCTAGGTTTTGAA

AAAGACTTTTCATGTCATTCCCATTTAGGTCCGATTCGGATCCCTCCGTTGTTTCCTTTTCCTTCCGCAC

CCTTTCCTCGAAATGAGAAAGAAGATGGTACACTTGAATTGTATTATTTAAGTGCTTATTGCTTGCCAAA

GATCCTACTTCTACAATTGGTAGGTCACCGGGTTATTCAAATAAGTCGTGTTTTCCGTGGTTTTCCCATG

TTACAACTTCCGTACCAATTCGGTCGATCCGGAATGGATCGGTTAAACATTCTATTAGGGAGCCTGGTCT

TGACTCTTCTGTGTGGTATTCATTCTCGTTCGGCTCTTGGAATTACATCCAGCAGTGGTTGGAACAGCTC

GCAAAATCCAACCACTTCACCTACTTCATTGCCCCCAACCGTTTCTCGTACCTCTATTGAAACAGAATGG

TTTCATGTTCTTTCATCGATTGGTTATTCCTCTCCGTTCGTATCTCTTTTTCCAATTTCGGTCTCGATTA

GTTCACAAGATTG-ATGTCCGTTTCGTTATTACAACCTTC-----TTTTTTGATGTCAAAGACCAGAAGC

TACGCGCAAATTCTCATTGGATTTCGGTTGTTCTTAACAGCGATGGCTATTCATTTAAGTCTTCGGGTAG

CACCACTAGATCTTCAACAAGGTGGAAATTCTCGTATTCCGTATGTACATGTTCCTGCGGCTCGGATGAG

TATTCTTGTTTATATCGCTACGGCTATAAACACTTTCTTGTTCCTATTAACAAAACATCCCCTTTTTCTT

CGCTCTTCCGGAACCGGTATAGAAATGGGTGCTTTTTCTACGTTGTTTACCTTAGTTACTGGGGGGTTTT

GGGGAAGACCTATGTGGGGCACCTTTTGGGTGTGGGATGCTCGTTTAACCTCTGTATTCATCTCGTTCCT

TATTTACCTGGGTGCACTGTGTTTTCAAAAGCTTCCTGTCGAACCGGCTCCTATTTCAATCCGTGCTGGA

CCGATCGATATACCAATAATCAAGTCTTCAGTCAACTGGTGGAATACATCGCATCAACCTGGGAGCATTA

GCCGATCTGGTACATCCATACATGTTCCTATGCCCATTCCAATCTTGTCTAACTTTGCTAACTCCCCCTT

CTCAACCCGTATCTTGTTCGTTCTGGAAACACGTCTTCCTATTCCATCTTTTCTCGAATCTCCTTTAACG

GAAGAAATAGAAGCTCGAGAAGGAATAC--GCAGGCTAGAAAGATGCTATTTGCTGCTATTCTATCTATT

TGTGCATCAAGTTCGAAGAAGATCTCAATCTATAATGAAGAAATGATAGTAGCTCGTTGTTTTATAGGCT

TTATCATATTCAGTCGGAAGAGTTTCAGTAATACTTTCAAAGAGATTCTCGACGGGAGAATCCAGGCTAT

TCAGGAAGAATTGCAGCAATTCCCCAATCCTAACGAAGTAGTTCCTCCGGAATCCAATGAACAACAACGA

TTACTTAGGATCAGCTTGCGAATTTGTGGCACCGTAGTAGAATCATTACCAATGGCACGCTGTGCGCCTA

AGTGCGAAAAGACAGTGCAAGCTTTGTTATGCCGAAACCTAAATGTTAAGTCAGCAACACTTCTAAATGC

CACTTCTTCCCGTCGCATCCGTCTTCAGGACGATCTAGTCACAGGTTTTCACTTCTCAGTGAGCGAAAGA

TTTGTCCCCGGGTGTACGTTGAAAGCTTCTATAGTAGAACTCATTCGAGAGGGCTTGGCGGTCTTAAGAA

TGGTTCGGGTGGGGGATTTCTCTT-TGAAAGAGGCGATCAGAATGGTACTCGAATCCATTTACGATCCCG

AGTTTCCAGACACATCGCACTTCCGCTCGGGTCGAGGCTGCCACTCGGCCCTAAGACGGATCAAAGAAGA

GTGGGGAACCTCTCGCTGGTTTTTGGAATTCGACATCAGGAAGTGTTTTCACACCATCGACCGACATCGA

CTCATCTCAATCTTTAAGGAGGAGATCGACGATCCCAAGTTCTTTTACTCCATTCAGAAAGTCTTTTCCG

CCGGACGACTCGTAGGAGGTGAGAAGGGGCCTTACTCCGTTCCACACAGTGTACTACTATCGGCCCTACC

AGGCAACATCTACCTACACAAGCTCGATCAGGAGATAGGGAGGATCCGACAGAAGTACGAAATTCCGATT

GTTCAGAGAATCAGATCGGTTCTATTAAGGACAGGTCGTATTGATGACCAAGAAAACTCTGGAGAAGAAG

CAATGGAAATCTCTCCCAGAGCTGCGGAACTAACGACTCTATTAGAAAGTCGAATTACCAACTTTTACAC

GAATTTTCAAGTGGATGAGATCGGTCGAGTGGTCTCAGTTGGAGATGGGATTGCACGTGTTTATGGATTG

AACGAGATTCAAGCCGGGGAAATGGTTGAATTTGCCAGCGGTGTGAAAGGAATAGCGTTGAATCTTGAGA

ATGAGAATGTAGGGATTGTTGTCTTTGGTAGTGATACCGCTATTAAAGAAGGAGATCTTGTCAAGCGCAC

TGGATCTATTGTGGATGTTCCTGCGGGAAAGGCTATGCTAGGGCGTGTGGTCGACGCGTTGGGAGTACCT

ATTGATGGAAGAGGGTCTCTAAGCGATCACGAGCGAAGACGTGTCGAAGTGAAAGCCCCTGGGATTATTG

AACGTAAATCTGTGCACGAGCCCATGCAAACAGGGTTAAAAGCGGTAGATAGCCTGGTTCCTATAGGCCG

TGGTCAACGAGAACTTATAATCGGGGATCGACAAACTGGAAAAACTGCTATTGCTATCGATACCATATTA

AACCAAAAGCAAATGAACTCAAGGAGCACCTCTGAGAGTGAGACATTGTATTGTGTCTATGTAGCGATTG

GACAGAAACGTTCAACTGTGGCACAATTAGTTCAAATTCTTTCAGAAGGGAATGCTTTGGAATATTCCAT

TCTTGTAGCAGCCACCGCTTCGGATCCTGCTCCTCTGCAATTTCTGGCCCCATATTCTGGGTGTGCCATG

GGGGAATATTTCCGCGATAATGGAATGCATGCATTAATAATATATGATGATCTTAGTAAACAGGCGGTGG

CATATCGACAAATGTCATTATTGTTACGCCGACCACCAGGCCGTGAGGCTTTCCCAGGGGATGTTTTCTA

TTTACATTCCCGTCTCTTAGAAAGAGCCGCTAAACGATCGGACCAGACAGGCGCAGGTAGCTTGACCGCC

TTACCCGTCATTGAAACACAAGCTGGAGACGTATCGGCCTATATTCCCACCAATGTGATCTCCATTACTG

ATGGACAAATCTGTTTGGAAACAGAGCTCTTTTATCGCGGAATTAGACCTGCTATTAACGTCGGCTTATC

TGTCAGTCGCGTCGGGTCTGCCGCTCAGTTGAAAACTATGAAACAAGTCTGCGGTAGTTTAAAACTGGAA

TTGGCACAATATCGCGAAGTGGCCGCCCTTGCTCAATTTGGGTCAGACCTTGATGCTGCGACTCAGGCAT

TACTCAATAGAGGTGCAAGGCTTACAGAAGTACCGAAACAACCACAATATGCACCACTTCCAATTGAAAA

ACAAATTATAGTCATTTATGCAGCTGTCAATGGATTCTGTGATCGAATGCCATTAGACAAAATTTCTCAA

TATGAGAGAGCCATTCCAAGTAGTGTAAAACCAGAATTACTACAATCTCTTTTAGAAAAAGGTGGGTTAA

CTAACGAAAGAAAGATGGAACCAGATGCATTCTTAAGAGAAAGTGCTTTGCCTTACCTATGATGCAAGAA

ATAATGG-CATCCAACGCAAAGCGGCCTTTCATTCCCTTGTTTCGTCGTGGCACACCCCCCCCACAAGCA

CCCCCCGGCTCAGGGGGGACCAGAAAAAGCCTTTCGTTTTCCCCCCTTCGTCGGCCCTTGCCGCCTTCCT

TAACAAGCCCTCGAGCCTCCTTTGCGCCGCCTTCCTCATAGAAGCCGCCGGGTTGACCCCGAAGTCCGAA

TTCTATGGTAGAGAAGGCTTTAATAATAATTGGGCCATGAGAGACTTTATGTTGTATTGCAAAAGAAAGG

GCCTGCTGATAGAGCTGGGCGGAGAGGCGATACTAGTTATCAGGTCAGAGAGACGCCTGGCCCGTAAGCT

GGCCCCCTTAAAAACCCATTACAACATAAGGATTTGTTACGCGCGATATGCCGACGACTTACTACTGGGA

ATCGTGGGTGCCGTAGAGCTTCTCATAGAAATACAAAAACGTATCGCCCACTTCCTACAATCCGGCCTGA

ACCTTTGGGTAAGCTCTGCAGGATCAACAACAATAACTGCACGGAGTACGGTAGAATTCCTCGGTACGAT

CATTCGGGAAGTCCCTCCGAGGACGACTCCCATACAATTCTTGCGAGAGCTGGAGAAGCGTCTACGGGTA

AAGCACCGTATCCATATAACTGCTTGCCACCTACGCTCCGCCATCCATTCCAAGTTTAGGAACCTAGGGT

TTAGTATCCCGATCAAACAGCTGACGAAGGGGATGAGCGGAACAGGGAGTCTACTGGACGCGGTTCAACT

AGCGGAGACTCTTGGAACAGCTGGAGTAAGAAGTCCCCAAGTGAGCGTATTATGGGGGACCGTCAAGCAC

ATCCGGCAAGGATCAAGGGGGATCGAGTTCTTGCATAGCTCAGGTCGGAGCAAGGTGCCATCGGACGTTC

AACAGGCAGTCTCACGATCGGGCACTCATGCCCGGAAGTTGTC---ATTGTATACTCCCGCGGGTCGGAA

GGCGGCGGGGGAAGGAGGGGGACACTGGGCGAGATCTATCAGCAGCGAATTCCCCATACAAATAGAGGCG

CCTATCAAAAAGATACTCCGAAGGCTTCGGGATCGAGGTATCATTAGCCGAAGAAGACCCTGGCCAATCC

ACGTGGCCTGCTTGACGAACGTCAGCGACGGAGACATCGTAAATTGGTCCGCGGGCATTGCGATAAGTCC

TCTGTCCTACTACAGGTGCCGCGACAACCTTTACCAAGTCCGAACGATTGTCGACCACCAGATCCGCTGG

TCTGCAATATTCACCCCGGCCCACAAGCACAAATCCTCGGCGCGGAATATAATCCCAAAGTACTCCAAAG

ACTCAAATATAGTAAATAAAGAAGGTGGTAAGACCCTTGCAGAGTTCCCCAACAGCATAGAGCTTGGGAA

GCTCGGACCCGGTCAAGATCC-GAACAACAAGGAGCACTCAACTACTA------------------TGGT

CCAACTACATAACTTTTTCTTTTTCATTACTTCCATGGTCGTGCCTCGTGGCACGGCAGCACCCGTACTA

TTGAAATGGTTCGTCAGTAGAGATGTTCCCACAGGTGCCCCTTCTTCCAATGGTACTATAATTCCTATTC

CTATCCCTTCATTCCCTCTTTTGGTCTATCTACATTCCAGGAAATTCATACGCTCCACGGACGGAGCAAA

AAGTGGAGTCTTGGTCAGAGCAAGCCGCCCTATTCTAT------TACCAGACATAATTGGGAGAAGCTCA

TCCGAAACTAGAGCTAGAAACGCCTCATTTCGTTTTGTTCCTGTTCTTCATTTCCTTCTTCTTCAATCCA

A------GGGGGACTTCTCATATTTAGAATCTTTTTGCGGTGTGCTCCGTTTACTATTCTTTCGTACTTT

CTTCTTTTTACCACGCGATAGGTCAGCGAAGCGTGAGCGGGCGCGGAGAAGGAAAGGCCAAACACTTCGG

CCTAAC------GGGAATGAGCAACGACGAAATGACAAGATGAGGTGCCCCGGGCATCCCCATTTAGAAA

TAGA---AAGAAGGATCGAAGGTTTTGGGCCTGTCGCCTTCCCCGTCCCCCCTTCGTCGGGTGGTCCTTG

TGTGGGGGGTGCGCCACCAGAAATCGGGCTTGAAGCTCTCGCCTTACCAACGAGCCGACAGCTGATGGCT

GTTGGTCACGACTACTACCAAAAAGCTCCAATGAAGATGAATATTTCACATGGAGGAGTGTGCATCTGTA

TGTTGGGTGTTCTTCTGT---TACATAGCTGTTCCAGCTGAAATACTTGGAATAATTCTACCACTTCTAC

TAGGAGTAGCCTTTTTAGTGCTAGCTGAACGTAAAGTAATGGCTTTTGTGCAACGTCGAAAGGGTCCTGA

TGTAGTGGGATCGTTTGGATTGTTACAACCTATAGCAGATGGTTTGAAATTGATTCTAAAAGAACCTATT

TCACCAAGTAGTGCTAATTTCTCCCTTTTTAGAATGGCTCCAGTGGCTACATTTATGTTAAGTCTGGTCG

CTCGGGCCGTTGTACCTTTTGATTATGGTATGGTATTGTCAGATCCGAACATAGGGCTACTTTATTTGTT

TGCCATATCTTCGCTAGGTGTTTATGGAATTATTATAGCAGGTTGGTCTAGTATTTATTATATACGCTTA

GCGAAAAGAATGTTTTTTGATACACCTAGGACATGGATTCTATATGAACCAATGGATCGTGACAAGTCGT

TACTACTAGCAATGACTTCCTCTTTCATTACTTCATTCTTTCCATATCCCTCTCCTTTGTTCTCAGTTAC

TCATCAAATGGCACTCAGTTCATATCTTTA-ATGTCAGAATTTGCACCTATTTGTATCTATTTAGTGATC

AGTCCGCTAGTTTCTTTGATCCCACTTGGTGTTCCTTTTCCATTTGCTTCCAATAGTTCGACCTATCCAG

AAAAATTGTCGGCCTACGAATGTGGTTTCGATCCTTCCGGTGATGCCAGAAGTCGTTTTGATATACGATT

TTATCTTGTTTCAATTTTATTTATTATCCCTGATCCGGAAGTAACCTTTTCCTTTCCTTGGGCAGTACCT

CTCAACAAGATTGATCCGTTTGGATCTTGGTCCATGATGGCCTTTTTATTGATTTTGACGATTGGATCTC

TCTATGAATGGAAAAGGGGTGCTTCGGATCGGGAATA--AAAGTGTTTCTTACGATTACGCCCAACAGCC

CACTTGAGCAATTTGCCATTCTTCCATTGATTCCTATTCATATAGGAAACTTGTATTTCTCATTCACAAA

TCCATCTTTGTTTATGCTGCTAACTCTCAGTTTGGTCCTACTTCTGGTTCATTTTGTTACTAAAAAGGGA

GGAGGAAACTCAGTACCAAATGCTTGGCAATCCTTGGTAGAGCTTATTTATGATTTCGTGCCGAACCCGG

TAAACGAACAAATAGGTGGTCTTTCCGGAAATGTTAAACAAAAGTTTTTCCCTCGCATCTCGGTCACTTT

TACTTTTTCGTTATTTCGTAATCCCCAGGGTATGATACCTTATAGCTTCACAGTTACAAGTCATTTTCTC

ATTACTTTGGGTCTCTCATTTTCTCTTTTTATTGGCATTACTATAGTGGGATTTCAAAAAAATGGGCTTC

ATTTTTTAAGCTTCTCATTACCCGCAGGAGTCCCACTGCCGTTAGCACCTTTTTTAGTACTCCTTGAGCT

AATCCCTCATTGTTTTCGCGCATTAAGCTCAGGAATACGTTTATTTGCTAATATGATGGCCGGTCATAGT

TCAGTAAAGATTTTAAGTGGGTTCGCTTGGACTATGCTATGTATGAATGATCTTTTATATTTCATAGGAG

ATCTTGGTCCTTTATTTATAGTTCTAGCATTAACCGGTCTGGAATTAGGTGTAGCTATATCACAAGCTCA

TGTTTCTACGATCTCAATCTGTATTTACTTGAATGATGCTACAAATCTCCATCAAAG-------------

----------------ATATGTGGGCACCTGATATCTATGAGGGTTCACCCACCCCGGTTACAGCATTCC

TTTCTATTGCGCCTAAAATTTCTATTTCTGCTAATATTTCACGTGTTTCTATTTATGGTTCCTATGGAGC

TACATTGCAACAAATCTTCTTTTTCTGCAGCATTGCTTCTATGATCTTAGGATCACTGGCCGCCATGGCC

CAAACGAAAGTAAAAAGACTTCTAGCTCATAGTTCAATTGGACATGTAGGTTATATTCGTACTGGTTTCT

CATGTGGAACCATAGAAGGAATTCAATCACTACTAATTGGTATCTTTATTTATGCATCAATGACGATAGA

TGCATTCGCCATAGTTTCAGCATTACGGCAAACCCGTGTCAAATATATAGCGGATTTGGGCGCTCTAGCC

AAAACGAATCCTATTTCGGCTATTACCTTCTCCATTACTATGTTCTCATACGCAGGAATACCCCCGTTAG

CCGGCTTTTGTAGCAAATTCTATTTGTTCTTCGCCGCTTTGGGTTGTGGGGCTTACTTCCTAGCCCTAGT

GGGAGTAGTGACTAGCGTTATAGGTCGT-TGTTCGATAGCCCGACCGTAGTGATGTTAATTGTGGTTACA

TCCATAAGTAGCTTGGTCCATCTTTATTCCATTTCATATATGTCTGAGGATCCGCATAGCCCTCGATTTA

TGTGTTATTTATCCATTCCTACTTTTTTTATGTCAATGTTGGTGACTGGAGATAACTCTCTTCAATTATT

CCTGGGATGGGAGGGAGTAGGCCTTGCTTCATATTTGTTAATTCATTTCTGGTTTACACGACTTCAGGCA

GATAAAGCAGCTATAAAAGCTATGCCTGTCAATCGAGTAGGTGATTTTGGATTAGCTCTTGGGATTTCGG

GTTGTTTTACTCTCTTTCAAACAGTAGACTTTTCAACCATTTTTGCTTGTGCTAGTGCCCCCAGAAATTC

TTGGATTTCTCGCAATATGAGATTGAATGCCATAACTCTTATTTGTATTTTACTTCTTATTGGTGCTGTT

GGAAAATCTGCACAGATAGGATCGCATACTTGGTCACCCGATGCTATGGAGGGTCCCACTCCAGTATCCG

CTTTGATTCATGCAGCTACTATGGTAACAGCTGGCGTTTTCATGATAGCAAGGTGCTCCCCTTTATTTGA

ATACCCACCTACGGCTTTGATTGTTATTACTTTTGCAGGAGCTACGACGTCATTCCTTGCGGCAACCACT

GGAATATTACAGAACGATCTAAAGAGGGTCATAGCTTATTCAACTTGCAGTCAATTAGGCTATATGATCT

TTGCTTGCGGCATCTCTAACTATTCGGTTAGCGTCTTTCACTTAATGAATCACGCGTTTTTCAAAGCATT

ACTATTCCTGAGTGCAGGTTCGGTGATTCATGCCATGTCGGATGAGCAAGATATGCGGAAGATGGGGGGG

CTTGCCTCCTCGTTCCCTTTTACCTATGCCATGATGCTCATGGGCAGCTTATCTCTAATTGGATTTCCTT

TTCTAACTGGATTTTATTCCAAAGATGTGATCTTAGAGCTCGCTTACACTAAGTATACCATCAGTGGGAA

CTTTGCTTTCTGGTTGGGAAGTGTCTCTGTCCTTTTCACTTCTTATTACTCTTTTCGTTCACTTTTTCTA

ACATTTCTAGTACCAACTAATTCATTCGGGCGAGACATCTTACGATGTCATGATGCGCCCATTCCTATGG

CCATTCCTTTAATACTTCTGGCTCTCGGGAGTCTCTTTGTAGGATACTTGGCCAA--TAACACAAAGAAG

ATACAGTTCACTCAACGATTGCCTTTGGGTTCCGAACTCCATATGGGGAAGGAGCGTTGTTGTTTGCGAG

GTCTCGATCATTTACATGGACCCACTTTTCATTCCATTTGTGGGAATTTGATGATCTATAAACCGTCCTT

AACGAACGATCGGCTCATGTT------TGAGCATGATGAATCACTTCGTGCCGACCTGTTGCCAATAAAC

TTTCCGGCCTCATATGAGAATGGAAAACTGGAGCATTTTCTGCATCGGTGGATGAAGAATCGCGAACATA

ATAATTTATGGTTGGCCATGTTCCCAGAAAAAAGATACTTTCGAGAAACGACGAGTACGACTGAAGTGGC

TATACATACAAATCCATTTACGGATCTATATGCTTCGATTGGAACTGGAAGTTCCAGAACAGGTGGCTGG

TATACCACCATAATGAAACTGCCTTTTCTTTTTTTTATTTGGATAGGATTTATGTTGGCTTCGTTGGGAG

GCTCGCGTAGTTTGTTACGTCAGCTCCAAAAGGATAAGTTGCGTTGGAATCGAGAAAGTTCCGTGG----

AGTTCATAATTGCATAACCAATTTTTGGGCCAATTCCCCCTTCGTACTACCAAAAAATGAGATTCTTGCC

GAATCCGAGTTTGCTGCTCCAACCATTACCAAACTAATACCTATTCCGTTTAGTACTTCAGGTGCTTCTG

TTGCGTATAATGTAAATCCCGTAGCGGATCAATTCC---------------AACGAGCCTTTCAAACTAG

TACTTTTTGGAATCGACTCTATAGCTTCTTCAATAAACGCTGGTTCTTCGATCAAGTTTTGAATGACTTT

CTAGTCAGATCGTTCCTGCGTTTCGGATATGAAGTCTCATTCGAAGCTTTAGACAAAGGTGCTATTGAGA

TATTGGGCCCTTATGGTATCTCGTACACATTCCGACGATTGGCCGAGCGAATAAGTCAACTTCAAAGTGG

ATTTGT-TTGTTCCATGATCTATGGGTCTACTGGAGCTACCCACTTCGATCAATTAGCCAAGATTTTGAC

CGGATACGAAATCACTGGTGCTCGATCTAGTGGTATTTTTATGGGGATTCTATCTATCGCTGTAGGATCC

CTATTCAAGATCACTGCAGTTCCTTTT--GGTCCATGCACATCGCTCTCTCCAGGAGGTTGGCCGCCTAT

CCTAGATCTTCCCATTTCCAAGAAGATCCCGGGCTCGATCCGGTTTAGTATCAAGGTGATTCTTTTTCTG

TTCCTATATATATGGGTCCGTGCAGCATTTCCACGATATCGTTATGATCAATTAATGGGACTTGGCCGGA

AAGTGTTCTTGCCTCTATCATTAGCTCGGGTAGTCGCCGTTTCTGGTGTTTTAGTCACCTTTCAATGGCT

CCCTTAAATGCCTCAACTGGATAAATTCACTTATTTCACACAATTCTTCTGGTCATGCCTTTTCCTCTTT

ACTTTCTATATTCCCATATGCAATGATGGAGATGGAGTACTTGGGATCAGCAGAATTCTAAAACTACGGA

ACCAACTGGTTTCACACCGGGAGAACAACATCCGGA------GCAACGACCCCAAGAGTTTGGAAGATAT

CTTGAAAAAAGGTTTTAGCACCGGTGTATCCTATATGTACTCCAGTTTATTCGAAGTATCCCAATGGTGT

AACGCCGTCGACTTATTGGGAAAAAGGAGGAAAATGACTTTGATCTCTTGTTTCGGAGAAATAAGGGGCT

CACGAGGAATGGAAAGAAACATATTCTATTTGATCTCGAAGTCCTCATATAGCACTTC------------

---TTCCAATCCTGAATGGGGGATCACTTGTAGGAATGACATAATGCTAATCCATGTTCCACACGGCCAA

GGAAGCATCGTTTTTTAATCTCT

>Mirabilis jalapa NC056991.1

-TGATACTTTCTGTTTTGTCGAGCCCGGCTTTGGTCTCTGGTTTGATGGTTGTACGTGCTAAAAATCCGG

TACATTCCGTTTCGTTTCCCATCCCAGTCTTTCGCAACACTTCAGGTTTACTTCTTTTGTTAGGTCTCGA

CTTTTCCGCTATGATCTTCCCAGTAGTTTATATAGGAGCTATAGCCGTTTCATTCCTATTCGTTGTTATG

ATGTTCCATATTCAAATAGCGGAGATTCACGAAGAAGTATTGCGCTATTTACCAGTGAGTGGTATTATTG

GACTGATCTTTTGGTGGGAAATGTTCTTCATTTTAGATAATGAAACCATTCCATTACTACCAACCCAAAG

AAATACGACCTCTCTGAGATATACGGTTTATGCCGGAAAGGTACGAAGTTGGACGAATTTGGAAACATTG

GGCAATTTACTTTATACTTACTATTTTGTCTGGTTTTTGGTTTCTAGTCTTATTTTATTAGTAGCCATGA

TTGGGGCTATAGTACTGACTATGCATAGGACTACTAA------GGTGAAAAGACAGGATGTATTCCGACG

AAATGCTATTGATTCTAGAAGGACTATAATGAGGAGGACGACAGACC-ATGTCAATATATGAATTATTTC

ATTATTCGTTATTTCCGGGTCTTTTCATTGCATTCACTTACAACAAAAAACAACCACCAGCGTTTGGTGC

AGCACTTGCATTTTGGTGTATTCTTCTTTCTTTCCTTGGTCTTTTGTTCTGTCATATTCCTAATAACTTA

TCCAATTACAACGTATTAACCGCTAATGCACCTTTCTTTTATCAAATCTCAGGGACATGGTCTAATCATG

AAGGTAGTATTTTATTATGGTGTCGGATCCCAAGTTTTTATGGATTCCTTCTTTGTTACCGGGGTCGATC

CAAAAGCCATAATGTCTCAAAACGAGGAGGCCATAGAGAAAGTCTTATTTTTTCCTTTGTCTTAAACTTC

GTGAAGAACTCCATTCTATCTCTTCCTCGTTACGAACAAAAAAGTAGAGTTCTTCACGAACCCCAGTTGT

ACACTCTCTTCGTTCTACGAA---CTCTTGTTGATTCTGAACTTTGTTCGCTAAGGAACCGGAC------

------------TTTTTACGCGCCGCTTTACCCTGAAAGGAAAATGAGCTTTGCTCTTCTGGGCGCTAGG

CGCTCTCGTGGTTCGCGAGAAGGAAAAAGGACTCATCCTTTGTTGCATCTGGCACGAGATGATAAAGAGA

GAGCTTCGTCTATCGATGAACAGCGGATTGACGGAGCTCTTGGCATTGCTTTGTTTTTCTTTCCTTTCCT

ATCAGCGAGTTCCGATCCTTTTGTTCGAAATTTCTTCGTTCGTACCGAACCGCTTGCCGAATCAAATCCT

GTTCCACAAGATCCTATATCAGCTATACATCCTCCTTGCATTTATGCCGGAGACGTCGCCAGTGCTATGG

GCTTTGGCTTATGTAGATCAAAAATGATGAATGGGATTGTGGCACTCCACTCGCCGCCAATGCGGAAGGA

TGTCGCCGAAAAGAATGGAACGCTGCTTTGCTCTGCTGGATGCGTCGGATCCCGTATAACAAGTGAGCTC

TTTACCCTTAAATTCAAACATGTGGGCGCCAAATGCTATCCTGCTCTATTGTTGCGTAGCAATAGAAGCC

TGCTC---ATGCTGCTTCGGCGGCGCTTTTTCGCCTTCTCTTCGCTCTGGACAAGAGCGCTAGCGGACAC

GGGGAGGGAGCG------GGCGAAGCGTTTCT------TTCGTAATGGAAAGAAAGATACCACTACTTCG

CCTCTTTGTTGGACCGCCGGCGCGAACACAGTGGTCTCTGACCAGGACCAGGAACCAATTCGAATTTGGA

TCTTGATATGTCGGTGGTTTTTAACCGTAGGCATCTTGCCAGGAAGTTGGTGGGCTCATCATGAATTAGG

TCGGGGTGGCTGGTGGTTTCGGGATCCCGTAGAAAATGCTTCTTTTATGCCTCGGGTATTAGCCACAGCT

CGTATTCATTCAGTAATTTTACCCCTTCTTCATTCTTGGACTTTGCTTCTTAATATTGTGACTTTTCTAT

GCTGTGTGTTAGGAACCTTTTCAATACGGTCCGGATTGCTAGCTCCCGTTCATAGTTTTGCTACAGATGA

TACACGAGGAATCTTTTTATGGCGGTTCTTCCTTCTAATGACCGGCATATCTATGATTCTTTTCTCTCAG

ATGGAGCAGCAGGCATCGGTCCGTAGAACCTATAAAAAAGAGATGGTTGTAGCGCGAAGTACTCTTGTGC

ACTTACG---ATGATTGTTCGAGAATGGCTATTCTTCCCAATTGCTCCTTGTGATGCAGCGGAACCATGG

CAATTAGGATTTCAAGACGCAGCAACACCTATGATGCAAGGAATAATAGACTTACATCATGATATCTTTT

TCTTCCTCATTCTGATTTTGGTTTTCGTATCATGGATCTTGGTTCGCGCTTTATGGCATTTCCACTATCA

AAAAAATCCAATCCCGCAAAGGATTGTTCATGGAACTACTATCGAGATTATTCGGACCATATTTCCAAGT

ATCATCCTGATGTTCATTGCTATACCATCATTTGCTCTGTTATACTCAATGGACGAGGTAGTAGTAGATC

CAGCCATTACTATCAAAGCTATTGGACATCAATGGTATCGGAG---------------------------

----------------------------------------------------------------------

----------------------------------------------------------------------

----------------------------------------------------------------------

----------------------------------------------------------------------

----------------------------------------------------------------------

-----------------------ATGAGACGACTCTTTTTTGAACTATATCATAAACAGATCTTCTTCTC

CACACCAATCACGAGTTTTTCTCCATTCCTCTCGTATATTGTCGTAACGCCCTTAATGCTAGGTTTTGAA

AAAGACTTTTCATGTCATTTCCATTTAGGTCCGATTCGGATCCCTCCGTTGTTTCCTTTTCCTCCCGCAC

CTTTTCTTCGAAATGAGAAAGAAGATGGTACACTCGAATTGTATTATTTAAGTGCTTATTGCTTGCCAAA

GATCCTACTTCTACAATTGGTAGGTCACCGGGTTATTCAAATAAGTCGTGTTTTCTGTAGTTTTCCCATG

TTACAACTTCTTTACCAATTCGGTCAATCTGGAATGGATCGGTTAAACATTCTATTAGGGAGCCCGGTCT

TGACTCTTCTGTGTGGTATTCATTCTTGTTTGGCTCTTGGAATCACATCCAGCAGTGGTTGGAACAGCTC

GCAAAATTTAACCACTTCACCTACTTCATTGCCCTCAACCGTTTCTCGTACCTCTATTGAAACAGAATGG

TTTCATGTTCTTTCATCGATTGGTTATTTTTCTTCGTTCGTATCTCTTTTTCCAATTTCGGTCTCGATTA

GTTCACAAGATTG-ATGTCCGTTTTGTTATTACAACCTTC-----TTTTGTGATGTCAAAGACCAGAAGC

TACGCGCAAATTCTCATGGGATCTTGGTTGTTCTTAACAGCGATGGCTATTCATTGTTGTCTTTGGGTAG

CGCCACTAGATCTTCAACAAGGTGGAAATTCTCGTATTCTCTATGTACATGTTCCTGTGGCTCGGATGAG

TATTCTTGTTTATATCGTTACGGCTATAAACACTTTCTTGTTCCTATTAACAAAACATCCTCTTTTTCTT

CGCTCTTCCGGAACCGGTACAGAAATGGGTGCTTTTTCTACGTTGTTTACCTTAGTAACTGGGGGGTTTC

GGGGAAGACCCATGTGGGGCACCTTTTGGGTGTGGGATGCTCGTTTAACTTCTGTATTCATCTCGTTCCT

TATTTACCTGGGTGCACTGTGTTTTCAAAAGCTTCCTGTCGAACCGGCTCCTATTTCAATCCGTGCTGGA

CCGATCGATATACCAATAATCAAGTCTTCTGTCAACTGGTGGAATACATCGCATCAACCTGGGAGCATTA

GCCGATCTGGTACATCAATACATGTTCCTATGCCCATTCCAATCTTGTCTAACTTTGCTAACTTCCTCTT

CTCAACCCGTATCTTGTTTGTTCTGGAAATACGTCTTCCTATTCCATCTTTTCTCGAATCTCCTTTAACG

GAAGAAATCGAAGCTCGAGAAGGAATA-------------AAGATGCTATTTGCTGCTATTCTATCTATT

TTTGCATTAAGTTCGAAGAAGATCTCAATCTATAATGAAGAAATGATAGTAGCTCTATGTTTTATAGGCT

TTATCATATTCAGTCGGAAGAGTTTAGGTGAGACTTTCAAAGTGACTCTCGATGAGAGAATCCAGGCTAT

TCAGGAAGAATCGCAGCAATTCCCCAATCCTAACGAAGTAGTTCCTCCGGAATCCAATGAACAACAACGA

TTACTTAGGATCAGCTTGCGAATTTGTGGAACCGTTTTAGAATCATTACCAATGGCACGCTGTGCGCCTA

AGTGCGAAAAGACAGTGCAAGCTTTGTTATGTCGAAACCTAAATGTTAAGTCAGCAACACTTCCAAATGC

CATTTCTTCCCGTCGCACCCGTCTTCAGGACGATCTAGTCACAGGTTTTCACTTCTCAGTGAGTGAAAGA

TTTGTCCCCGGGTCTACGTTGAAAGCTTCTATAGTCGAACTCATTCGAGAAGGCTTGGCGGTCTTAAGAA

TGGTTCGGGTAGGAGATTCTCT--ATGAAAGAGGCGATCCGAATGGTACCCGAATCCATTTACGATCCCG

AGTTTCCAGACACATCGCACTTCCGCCCGGGTCGAGGCTGCCACTCGGCCCTAAGACGGATCAAAGAAGA

GTGGGGAACCTCTCGCTGGTTTTTGGAATTCGACATCAGGAAGTGTTTTCACACCATCGACCGACATCGA

CTCATCTCAATCTTTAAGGAAGAGATCGACGATCCCAAGTTCTTTTACTCCATTCAGAAAGTATTTTCTG

CCGGACGACTCGTAGGAGGTGAGAAGGGCCCTTACTCCGTCCCACACAGTGTACTACTATCGGCCCTACC

AGGCAACATCTACTTACACAAGCTCGATCAGGAGATAGGGAGGATCCGACAGAAGTACGAAATTCCGATT

GTTCAGAGAATCAGATTGGTTCTATTAAAGACAAGTCGTATTGATGACCAAGAAAACTCTGGAGAAGAA-

--ATGGAATTCTCTCCCAGAGCTGCGGAACTAACGACTCTATTAGAAAGTAGAATTACCAACTTTTACAC

GAATTTTCAAGTGGATGAGATCGGTCGAGTGGTCTCAGTTGGAGATGGGATTGCACGTGTTTATGGATTG

AACGAGATTCAAGCTGGGGAAATGGTGGAATTTGCCAGCGGTGTGAAAGGAATAGCCTTAAATCTTGAGA

ATGAGAATGTAGGGATTGTTGTCTTTGGTAGTGATACCGCTATTAAAGAAGGAGATCTTGTCAAGCGCAC

TGGATCTATTGTGGATGTTCCTGCGGGAAAGGCTATGCTAGGGCGTGTGGTCGACGCGTTGGGAGTACCT

ATTGATGGAAGAGGGGCTCTAAGCGATCACGAGCGTCGACGTGTCGAAGTGAAAGCCCCTGGGATTATTG

AACGTAAATCTGTGCACGAGCCTATGCAAACAGGGTTAAAGGCGGTAGATAGCCTGGTTCCTATAGGCCG

TGGTCAACGAGAACTTATAATCGGGGACCGACAAACGGGAAAAACAGCTATTGCTATCGATACCATATTA

AACCAAAAGCAACTGAACTCAAGGGCCACCTCTGAGAGTGAGACATTGTATTGTGTCTATGTAGCGATTG

GACAGAAACGTTCAACTGTGGCACAATTAGTTCAAATTCTTTCAGAAGGGAATGCTTTGGAATATTCCAT

TCTTGTAGCAGCCACCGCTTCGGATCCTGCTCCTCTTCAATTTCTGGCCCCATATTCTGGGTGTGCTATG

GGAGAATATTTCCGCGATAATGGAATGCACGCATTAATAATCTATGATGATCTTAGTAAACAGGCGGTGG

CATATCGACAAATGTCATTATTGTTACGCCGACCACCAGGCCGTGAGGCTTTCCCAGGCGACGTTTTCTA

TTTACATTCTCGTCTCTTAGAAAGAGCCGCTAAACGATCGGACCAGACAGGTGCAGGTAGCTTGACCGCC

TTACCCGTCATTGAAACACAAGCTGGAGACGTATCGGCCTATATTCCCACCAATGTGATCTCCATTACAG

ATGGACAAATCTGTTTGGAAACAGAGCTCTTTTATCGCGGAATTAGACCTGCTATTAACGTCGGCTTATC

TGTCAGTCGCGTCGGGTCTGCCGCTCAGTTGAAAGCTATGAAACAAGTCTGCGGTAGTCCAAAACTTGAA

TTGGCACAATATCGCGAAGTGGCCGCCTTTGCTCAATTTGGGTCAGACCTTGATGCTGCGACTCAGGCAT

TACTCAATAGAGGTGCAAGGCTGACAGAAGTACCGAAACAACCACAATATGCACCACTTCCAATTGAAAA

ACAAATTCTAGTCATTTACGCAGCTGTCAATGGATTCTGTGATCGAATGCCACTAGATAAAATTTCTCAA

TATGAGAGAACCATTCCAAATAGTGTAAAACCAGAATTATTACAATCCCT------AAAGGGGGGCTTAA

CTAACGAAATAAAGATGGAACTAGATGAATTCTTAAAAGAATGCGCTTTGACTTAC--------------

-------GCATCCAACGCAAAGCGGCCTTTCATTCCCTTGTTTCGTCGTGGCACACCCTCCCCACAAGCA

CCCCCCGGCTCAGGGGGGACCAGAAAAGGCCTTTCGTTTTCCCCCCTTCGTCGGCCCTTGCCACCTTCCT

TAACAAGCCCTCGAGCCTCCTTTTCGCTGCCTTCCTCATAGAAGCCGCCGGGTTGACCCCGAAGGCCGAA

TTCTATGGTAGAGAACGCTGTAATAATAATTGGGCCATGAGAGACCTTTTTAAGTATTGCAAAAGAAAGG

GCCTGCTGATAGAGCTGGGCGAGGCAGCGATACTAGTTATCAGGTCAGAGAAAGGCCTGGCCCGTAAGCT

GGCCCCCTTAAAAACCCATTACTTAATAAGGATTTGTTACGCGCGATATGCCGACGACTTACTATTGGGA

ATCGTGGGTGCCGTAGAGCTTCTCATAGAAATACAAAAACGTATCGCCCACTTCCTACAATCCGGCCTGA

ACCTTTTGGTAGGCTCTGCGGGATCAACAACAATAGCTGCACGGAGTACGGTAGAATTCCTCGGTACGGT

CATTCGGGAAGTCCCTCCGAAGACGACTCCCATACAATTCTTGCGAGAGCTGGAGAAGCGTCTACGGGTA

AAGCACCGTATCCATATAACTGCTTGCCACTTACGCTCTGCCATTCATTCCAAGTTTAGGAACCTAGGTA

AGAGTATCCCGGTCAAACAGCTGACGAAGGGGATGAGCAAAACAGGGAGTCTACTGGACGCGGTTCAACT

AGCGGAAACTCTTGGAACAGCTAGAGTAAGAAGTCCCCAAGTGAGCGTATTATGGGAGACCGTCAAGCAC

ATCCGGCAAGGATCAAGGGAGATCTCGTTGTTGCATAGCTCAGGTCAGAGCAAGGTGCCATCGGACGTTC

AACAGGCAGTCTCGCGATCGGGCATGAGTGTCCGGAAGTTGTC---ATTGTATACTTCCGCGGGTCGGAA

GGCGGCGGGGGAAGGAGGGGGACACTGGGCGAGATCTATCAGCAGCGAATTCCCCATACAGATAGAAGCG

CCTATCAAAAAGATACTCCGAAGGCTTCGAGATCGAGGTCTCATTAGCCGAAGAAGACCCTGGCCAATCC

ACGTGGCCTGCTTGACGAACGTCAGCGACGGAGACATCGTAAATTGGTCCGCGGGCATCGCGATAAGTCC

TCTGTCCTACTACAGGTGCCGCGACAACCTTTACCAAGTCCGAACGATTGTCGACCACCAGATCCGCTGG

TCTGCAATATTCACCCCAGCCCACAAGCACAAATCCTCAGCGCGGAATATAATCCCAAAGTACTCAAAAG

ACTCAAATATAGTAAATCAAGAAGGTGGTAAGACCCTAGCAGAGTTCCCCAACAGCATAGAGCTTGGGAA

GCTCGGACCCGGTCAAGATCC-GAACAACAAAGAGCACTCAACTACT------------------ATGGT

CCAACTACATAACTTTTTCTTTTTCATTACTTCCATGGTCGTGCCTTGTGGCACGGCAGCACCCGTACTA

TTGAAATGGTTCGTCAGTAGAGATGTTCCCACAGGTGCCCCTTTTTCCAATGGTACTCTAATTCCTATTC

CTATCCCTTCATTCCTTCTTTTGGTCTATCTACATTCCAGGAAATTCATACGCTCCATGGACGGAGTCAA

AAGTGGAGTCTTGGTCAGAGCAAGCTGCCCTATTTTAT------TACCAGACATAATTGGGAGAAGCTCA

TCCGAAACTAGAGCTAGAAACGCCTTATTTCGTTTCGTTCCCATTCTTCATTTTCTTCTTCTCGAATCCA

A------GGGGGACTTCCCATATTTAGAATCTTTTTGCGGTGTGCTCCGTTTACTATTCTTTCGTACTTT

CTTCTCTTTACCACGCGATAGGTCAGCGAAGCGTGAGCGGGCGCGGAGAAGAAAACGGCAAA------GG

CCTAAC------GGGAATGAGCAACGACGAAATGACAAGATAAAGTG---------CCCCCATT------

TAGA---AAGAAGGGTCGAAGGGTTTGGGCCTGTAGCTTTCCCCGTCCCCCCTTCGTCGGGTGGTGCTTG

TGTGGGGGGTGTGCTACCTGAAATCGGGCTTGAAGCTCCCGCCTTACCAACGAGCCGACTGCTGATGGCT

GTTGGTCACGACTACTACCAAAAAGTGAACATGAAGATGAATATTTCACATGGAGGAGTGTGCATCTTTA

TGTTGGGTGTTCTTCTG--CGTACATAGCTGTTCCAGCTGAAATACTTGGAATAATTCTACCACTTCTAC

TAGGAGTAGCCTTTTTAGTGCTAGCTGAACGTAAAGTAATGGCTTTTGTGCAACGTCGAAAGGGTCCTGA

TGTAGTGGGATCGTTCGGATTGTTACAACCTCTAGCAGATGGTTCGAAATTGATTCTAAAAGAACCTATT

TCACCAAGTAGTGCTAATTTCTCCCTTTTTAGAATGGCTCCAGTCACTACATTTATGCTAAGTCTGGTTG

CTCGGGCCGTTGTACCTTTTGATTATGGTATGGTATTGTCAGATCCGAACATAGGGCTACTTTATTTGTT

TGCCATATCTTCGCTAGGTGTTTATGGAATTATTATAGCAGGTTGGTCTAGTA-TTATTATATACGTTTA

GTGAAAAGAATGTTTTTTGATACACCTAGGACATGGATTCTATATGAACCAATGGATCGTGACAAGTCGT

TACTACTAGCAATGACTTCCTCTTTCATTACTTCATCCTTTCCATATCCTTCTCCCTTGTTCTCAGTTAC

TCATCAAATGGCACTCAGTTTATATCTTTAAATGTCAGAATTTGCGCCGATTTGTATCTATTTAGTGATC

AGTCTGCTAGTTTCTTTGATCTTACTCGGAGTTCCTTTTCCATTTTCTTCTAATAGTTCGACTTATCCAG

AAAAATTGTCGGCCTACGAATGTGGTTTCGATCCTTTCGGTGATGCCAGAAGTCGTTTCGATATACGATT

TTATCTTGTTTCAATTTTATTTATTATCCTTGATCTGGAAGTCACCTTTTTCTTTCCTTGGGCAGTACCT

CTCAACAAGATTGATCCGTTTGGATTTTGGTCTATGATGGCCTTTTTATTGATTTTAACGATTGGATTTC

TCTATGAATGGAAAAGGGGTGCTTTGGATTGGGAGTA--AAAGTGTTTATTACGATTACGCCCAACAGCC

CACTTGAGCAATTTTCCATTCTCCCATTGATTCCTATGAAAATAGGAAACTTGTATTTCTCATTCACAAA

TCCATCTTTGTTTATGCTGCTAACTCTCAGTTTGGTCCTACTTCTGCTTCATTTTGTTACTAAAAACGGA

GGAGGAAACTCAGTACCAAATGTTTGGCAATCCTTGGTAGAGCTTATTTATGATTTCGTGCTGAACCTGG

TAAACGAACAAATAGGTGGTCTTTCCGGAAATGTTAAACAAAAGTTTTTCCCTTGCATCTTGGTCACTTT

TACTTTTTTGTTATTTCGTAATCTCCAGGGTATGATACCCTATAGCTTTACAGTTACAAGTCATTTTCTC

ATTACTTTGGGTCTTTCATTTTCCATTTTTATTGGCATTACTATAGTGGGATTTCAAAGAAATGGGCTTC

ATTTTTTAAGCTTCTTATTACCTGCAGGAGTCCCACTGCCGTTAGCACCTTTTTTAGTACTCCTTGAGCT

AATCCCTCATTGTTTTCGCGCATTAAGCTCAGGAATACGTTTATTTGCGAATATGATGGCCGGTCATAGT

TCAGTAAAGATTTTAAGTGGGTTCGCTTGGACTATGCTATGTATGAATGATCTTTTATATTTCATAGGAG

ATCTTGGTCCTTTATTTATAGTTCTTGCATTAACCGGTCTTGAATTAGGTGTAGCTATATTACAAGCTCA

TGTTTTTACGATCTTAATCTGTATTTACTTGAATGATGCTACAAATCTCCATCAAA--------------

-----------------TATGTGGGCACCTGATATCTATGAGGGTTCACCCACCCCGGTTACAGCATTCT

TTTCTATTGCGCCTAAAATTTCCATTTCTGCAAATATTTTACGTGTTTTTATTTATGGTTCCTATGGAGC

TACATTGCAACAAATCTTCTTTTTCTGCAGCATTGCTTCTATGATCTTAGGAGCACTGGCCGCCATGGCC

CAAACGAAAGTCAAAAGACTTCTAGCTCATAGTTCAATTGGACATGTAGGTTATATTCGTACTGGTTTCT

CATGTGGAACCATAGAAGGAATTCAATCACTACTAATTGGTCTCTTTATTTATGCATCAATGACGATAGA

TGCATTCGCTATAGTTTCAGCATTACGGCAAACCCGTGTCAAATATATAGCGGATTTGGGCGCTCTAGCC

AAAACGAATCCTATTTCGGCTATTACCTTCTCTATTACTATGTTCTCATACGCAGGAATACCCCCGTTAG

CCGGCTTTTGTAGTAAATTCTATTTGTTCTTCGCCGCTTTGGGTTGTGGGGCTTACTTCCTAGCCCCAGT

GGGAGTAGTGACTAGCGTTATAGGTTGTTTGTTCGATAGCCCGACCGTAGTGATGTTAATTGTGGTTACA

TTCATAAGTAGCTTGGTCCATCTTTATTCTATTTCATATATGTCCGAGGATCCGCATAGCCCTCGATTTA

TGTGTTATTTATCCATTCTTACTTTTTTTATGCCAATGTTGGTGACTGGAGATAACTCTCTTCAATTATT

CTTGGGATGGGAGGGAGTAGGTCTTGCTTCATATTTGTTAATTCATTTTTGGTTTACACGACTTCAGGCA

GATAAAGCAGCTATAAAAGCTATGCTTGTCAATCGAGTAGGTGATTTTGGATTAGCTCTTGGGATTTCGG

GTCGTTTTACTCTCTTTCAAACAGTAGACTTTTCTACCATTTTTGCTTGTGCTAGTGCCCCTAGAAATTC

TTGGATTTTTTGCAATATGAGATTGAATGCCATAACTCTTATTTGTATTTTACTTTTTATTGGTGCTGTT

GGAAAATCTGCACAGATAGGATCGCATACTTGGTCACCCGATGCTATGGAGGGTCCCACTCCAGTATCTG

CTTTGATTCATGCAGCTACTATGGTAACAGCTGGCGTTTTCATGATAGCAAGGTGTTCCCCTTTATTTGA

ATACCCACCTACGGCTTTAATTGTTATTACTTTTGCAGGAGCTATGACGTCATTCCTTGCGGCAACCACT

GGAATATTACAGAACGATCTAAAGAGGGTCATAGCTTATTCAACTTGCAGTCAATTAGGCTATATGATCT

TTGCTTGCGGCATTTCTAACTATTCGGTTAGCGTCTTTCATTTAATGAATCACGCCTTTTTCAAAGCATT

ACTATTCCTGAGTGCAGGTTCGGTGATTCATGCCATGTCGGATGAGCAAGATATGCGGAAGATGGGGGGG

CTCGCCTCCTCGTTCCCTTTTACCTATGCCATGATGCTCATGGGCAGCTTATCTCTAATTGGATTTCCTT

TTCTAACTGGATTTTATTCCAAAGATGTGATCTTAGAGCTCGCTTACACTAAGTATACCATCAGTGGGAA

CTTTGCTTTCTGGTTGGGAAGTGTCTCTGTCCTTTTCACTTCTTATTACTCCTTTCGTTCACTTTTTCTA

ACATTTCTAGTACCAACTAATTCATTCGGGCGAGACATCTTACGATGTCATGATGCGCCCATTCCTATGG

CCATTCCTTTAATACTTCTGGCTTTCGGGAGTCTCTTTGTAGGATACTTGGCCAA-CTAACACAAAGAAG

ATACAGTTCACTCAACGATTGCCTTTGGGTTCCGAACTCCATATGGGGAAGGAACGTTGTTGTTTGCGGG

GTCTCGATCATTTACATGGACCCACTTTTCATTCCATTTGTGGGAATTTGATGATCTATAAACCGTCCTT

AACGAACGATCGGCTCATCTT------TGAGCATGATGAATCACTTCGTGCCGACCTGTTGTCAATAAAC

TTTTTGGCCTCATATGAGAATGGAAAACTGGAGCATTTTCTTCATCGGTGGATGAAGAATCGCGAACATA

ATAATTTCTGGTTAAGCATGTTCCCAGAAAAAAGATACTTTCGAGAAACAACGAGCACGACTGAAGTGGC

TATACATACAAATCCATTTACGGATCTATATGCTTCGATTGGAACTGGAAGTTCAAGAACAGGCGGCTGG

TATACTACCATAATGAAACTGCCTTTTCTTTTTTTTATTCGGATAGGATTTCTGTTGGCTTCGTTGGGAG

GCTCGCGTAGTTTGTTACGTCAGCTCCAAAAGGAAAAATTGCGTTGGAATCGAGAAAGTTACGTAA----

AGTTCATAATTGTATA--CCATTTTTGGGCCAATTCCCTCTTCGTACTACCAAAAAATGAGATTCTTGCC

GAATCCGAGTTTGCTGCTCCAACCATTACCAAACTAATACCTATTCTGTTTAGTACTTCAGGTGCTTCTG

TTGCGTATAATGTAAATCCCGTAGCGGATCAATTCC---------------AACGAGCCTTTCAAACTAG

TACTTTTTGTAATCGACTCTATAGCTTCTTCAATAAACGCTGGTTCTTCGATCAAGTTTTGAATGACTTT

CTAGTCAGATCGTTCTTGCGTTTCGGATATGAAGTCTCATTCGAAGCTTTAGACAAAGGTGCTATTGAGA

TATTGGGCCCCTATGGTATCTCCTACACATTCCGACGATTGGCCGAGCGAATAAGTCAACTTCAAAGTGG

ATTTGTT-TGTTCCATGATCTATGGGTCTACTGGAGCTACCCATTTCGATCAATTAGCCAAGATTTTGAC

CGGATACGAAATCACTGGTGTTCAATCTAGTGGTATTTTTATGGGGATTCTTTTTATCGCTGTAGGATCC

CTATTCAAGATCACTGCAGTTCCTTTTCTGGTCTATGCACATTGCTTTCTCCAGGAGGTTGGCCGCCTAT

CCTAGATCTTCCCATTTCCAAGAGGATCCCGGGCTCAATCTGGTTTAGTATCAAGGTGATTCTCTTTCTC

TTTCTATATATATGGGTCCGTGCAGCATTTCCACGATATCGTTATGATCAATTAATGGGACTTGGCCGGA

AAGTGTTCTTGCCTCTATCATTAGCTCGGGTAGTCGCCGTTTCTGGTGTTTTAGTCACCTTTCAATGGCT

CCCTTA-ATGCCTCAACTGGATAAATTTACTTATTTCACACAATTCTTCTGGTCATGCCTTTTCTTCTTT

ACTTTCTATATTTTAATATGCAATGATAGAGATGGAGTACTTGGGATCAGCAGAATTCTAAAACTACGAA

ACCAACTGCTTTCACACCGGGGGAACAACATCCAAA------GCAAGGACCCCAACAGTTTGGAAGATAT

CTTGAGAAAAGGTTTTCACACAGGTGTATCCTATATGTACTCTAGTTTATTCGAAGTATCCCAATGGTGT

AAGTCCGTCGACTTATTGGGAAAAAGGAAGAAAATCACTTTGATCTCTTCTTTCGGAGAAATAAGTGGCT

CGCGAGGAATGGAAAGAAACATATTTTATTTGATCTCGAAGTCTTCATATAGCACTTT------------

---TTCCAATCATGGATGGGGGATCACTTGTAAGAATGACATAATGCTAATCCATGTTCTACACGGCCAA

GGA--------------------

>Mirabilis himalaica NC048974.1

-TGATACTTTCTGTTTTGTCGAGCCCGGCTTTGGTCTCTGGTTTGATGGTTGTACGTGCTAAAAATCCGG

TACATTCCGTTTCGTTTCCCATCCCAGTCTTTCGCAACACTTCAGGTTTACTTCTTTTGTTAGGTCTCGA

CTTTTCCGCTATGATCTTCCCAGTAGTTTATATAGGAGCTATAGCCGTTTCATTCCTATTCGTTGTTATG

ATGTTCCATATTCAAATAGCGGAGATTCACGAAGAAGTATTGCGCTATTTACCAGTGAGTGGTATTATTG

GACTGATCTTTTGGTGGGAAATGTTCTTCATTTTAGATAATGAAACCATTCCATTACTACCAACCCAAAG

AAATACGACCTCTCTGAGATATACGGTTTATGCCGGAAAGGTACGAAGTTGGACGAATTTGGAAACATTG

GGCAATTTACTTTATACTTACTATTTTGTCTGGTTTTTGGTTTCTAGTCTTATTTTATTAGTAGCCATGA

TTGGGGCTATAGTACTGACTATGCATAGGACTACTAA------GGTGAAAAGACAGGATGTATTCCGACG

AAATGCTATTGATTCTAGAAGGACTATAATGAGGAGGACGACAGACC-ATGTCAATATATGAATTATTTC

ATTATTCGTTATTTCCGGGTCTTTTCATTGCATTCACTTACAACAAAAAACAACCACCAGCGTTTGGTGC

AGCACTTGCATTTTGGTGTATTCTTCTTTCTTTCCTTGGTCTTTTGTTCTGTCATATTCCTAATAACTTA

TCCAATTACAACGTATTAACCGCTAATGCACCTTTCTTTTATCAAATCTCAGGGACATGGTCTAATCATG

AAGGTAGTATTTTATTATGGTGTCGGATCCCAAGTTTTTATGGATTCCTTCTTTGTTACCGGGGTCGATC

CAAAAGCCATAATGTCTCAAAACGAGGAGGCCATAGAGAAAGTCTTATTTTTTCCTTTGTCTTAAACTTC

GTGAAGAACTCCATTCTATCTCTTCCTCGTTACGAACAAAAAAGTAGAGTTCTTCACGAACCCCAGTTGT

ACACTCTCTTCGTTCTACGAA---CTCTTGTTGATTCTGAACTTTGTTCGCTAAGGAACCGGAC------

------------TTTTTACGCGCCGCTTTACCCTGAAAGGAAAATGAGCTTTGCTCTTCTGGGCGCTAGG

CGCTCTCGTGGTTCGCGAGAAGGAAAAAGGACTCATCCTTTGTTGCATCTGGCACGAGATGATAAAGAGA

GAGCTTCGTCTATCGATGAACAGCGGATTGACGGAGCTCTTGGCATTGCTTTGTTTTTCTTTCCTTTCCT

ATCAGCGAGTTCCGATCCTTTTGTTCGAAATTTCTTCGTTCGTACCGAACCGCTTGCCGAATCAAATCCT

GTTCCACAAGATCCTATATCAGCTATACATCCTCCTTGCATTTATGCCGGAGACGTCGCCAGTGCTATGG

GCTTTGGCTTATGTAGATCAAAAATGATGAATGGGATTGTGGCACTCCACTCGCCGCCAATGCGGAAGGA

TGTCGCCGAAAAGAATGGAACGCTGCTTTGCTCTGCTGGATGCGTCGGATCCCGTATAACAAGTGAGCTC

TTTACCCTTAAATTCAAACATGTGGGCGCCAAATGCTATCCTGCTCTATTGTTGCGTAGCAATAGAAGCC

TGCTC---ATGCTGCTTCGGCGGCGCTTTTTCGCCTTCTCTTCGCTCTGGACAAGAGCGCTAGCGGACAC

GGGGAGGGAGCG------GGCGAAGCGTTTCT------TTCGTAATGGAAAGAAAGATACCACTACTTCG

CCTCTTTGTTGGACCGCCGGCGCGAACACAGTGGTCTCTGACCAGGACCAGGAACCAATTCGAATTTGGA

TCTTGATATGTCGGTGGTTTTTAACCGTAGGCATCTTGCCAGGAAGTTGGTGGGCTCATCATGAATTAGG

TCGGGGTGGCTGGTGGTTTCGGGATCCCGTAGAAAATGCTTCTTTTATGCCTCGGGTATTAGCCACAGCT

CGTATTCATTCAGTAATTTTACCCCTTCTTCATTCTTGGACTTTGCTTCTTAATATTGTGACTTTTCTAT

GCTGTGTGTTAGGAACCTTTTCAATACGGTCCGGATTGCTAGCTCCCGTTCATAGTTTTGCTACAGATGA

TACACGAGGAATCTTTTTATGGCGGTTCTTCCTTCTAATGACCGGCATATCTATGATTCTTTTCTCTCAG

ATGAAGCAGCAGGCATCGGTCCGTAGAACCTATAAAAAAGAGATGGTTGTAGCGCGAAGTACTCTTGTGC

ACTTACG---ATGATTGTTCGAGAATGGCTATTCTTCCCAATTGCTCCTTGTGATGCAGCGGAACCATGG

CAATTAGGATTTCAAGACGCAGCAACACCTATGATGCAAGGAATAATAGACTTACATCATGATATCTTTT

TCTTCCTCATTCTGATTTTGGTTTTCGTATCATGGATCTTGGTTCGCGCTTTATGGCATTTCCACTATCA

AAAAAATCCAATCCCGCAAAGGATTGTTCATGGAACTACTATCGAGATTATTCGGACCATATTTCCAAGT

ATCATCCTGATGTTCATTGCTATACCATCATTTGCTCTGTTATACTCAATGGACGAGGTAGTAGTAGATC

CAGCCATTACTATCAAAGCTATTGGACATCAATGGTATCGGAG---------------------------

----------------------------------------------------------------------

----------------------------------------------------------------------

----------------------------------------------------------------------

----------------------------------------------------------------------

----------------------------------------------------------------------

-----------------------ATGAGACGACTCTTTTTTGAACTATATCATAAACAGATCTTCTTCTC

CACACCAATCACGAGTTTTTCTCCATTCCTCTCGTATATTGTCGTAACGCCCTTAATGCTAGGTTTTGAA

AAAGACTTTTCATGTCATTTCCATTTAGGTCCGATTCGGATCCCTCCGTTGTTTCCTTTTCCTCCCGCAC

CTTTTCTTCGAAATGAGAAAGAAGATGGTACACTCGAATTGTATTATTTAAGTGCTTATTGCTTGCCAAA

GATCCTACTTCTACAATTGGTAGGTCACCGGGTTATTCAAATAAGTCGTGTTTTCTGTAGTTTTCCCATG

TTACAACTTCTTTACCAATTCGGTCAATCTGGAATGGATCGGTTAAACATTCTATTAGGGAGCCCGGTCT

TGACTCTTCTGTGTGGTATTCATTCTTGTTTGGCTCTTGGAATCACATCCAGCAGTGGTTGGAACAGCTC

GCAAAATTTAACCACTTCACCTACTTCATTGCCCTCAACCGTTTCTCGTACCTCTATTGAAACAGAATGG

TTTCATGTTCTTTCATCGATTGGTTATTTTTCTTCGTTCGTATCTCTTTTTCCAATTTCGGTCTCGATTA

GTTCACAAGATTG--TGTCCGTTTTGTTATTACAACCTTC-----TTTTGTGATGTCAAAGACCAGAAGC

TACGCGCAAATTCTCATGGGATCTTGGTTGTTCTTAACAGCGATGGCTATTCATTGTTGTCTTTGGGTAG

CGCCACTAGATCTTCAACAAGGTGGAAATTCTCGTATTCTCTATGTACATGTTCCTGTGGCTCGGATGAG

TATTCTTGTTTATATCGTTACGGCTATAAACACTTTCTTGTTCCTATTAACAAAACATCCTCTTTTTCTT

CGCTCTTCCGGAACCGGTACAGAAATGGGTGCTTTTTCTACGTTGTTTACCTTAGTAACTGGGGGGTTTC

GGGGAAGACCCATGTGGGGCACCTTTTGGGTGTGGGATGCTCGTTTAACTTCTGTATTCATCTCGTTCCT

TATTTACCTGGGTGCACTGTGTTTTCAAAAGCTTCCTGTCGAACCGGCTCCTATTTCAATCCGTGCTGGA

CCGATCGATATACCAATAATCAAGTCTTCTGTCAACTGGTGGAATACATCGCATCAACCTGGGAGCATTA

GCCGATCTGGTACATCAATACATGTTCCTATGCCCATTCCAATCTTGTCTAACTTTGCTAACTTCCTCTT

CTCAACCCGTATCTTGTTTGTTCTGGAAATACGTCTTCCTATTCCATCTTTTCTCGAATCTCCTTTAACG

GAAGAAATCGAAGCTCGAGAAGGAA----------------AGATGCTATTTGCTGCTATTCTATCTATT

TTTGCATTAAGTTCGAAGAAGATCTCAATCTATAATGAAGAAATGATAGTAGCTCTATGTTTTATAGGCT

TTATCATATTCAGTCGGAAGAGTTTAGGTGAGACTTTCAAAGTGACTCTCGATGAGAGAATCCAGGCTAT

TCAGGAAGAATCGCAGCAATTCCCCAATCCTAACGAAGTAGTTCCTCCGGAATCCAATGAACAACAACGA

TTACTTAGGATCAGCTTGCGAATTTGTGGAACCGTTTTAGAATCATTACCAATGGCACGCTGTGCGCCTA

AGTGCGAAAAGACAGTGCAAGCTTTGTTATGTCGAAACCTAAATGTTAAGTCAGCAACACTTCCAAATGC

CATTTCTTCCCGTCGCACCCGTCTTCAGGACGATCTAGTCACAGGTTTTCACTTCTCAGTGAGTGAAAGA

TTTGTCCCCGGGTCTACGTTGAAAGCTTCTATAGTCGAACTCATTCGAGAAGGCTTGGCGGTCTTAAGAA

TGGTTCGGGTAGGAGATTCTCTT-ATGAAAGAGGCGATCCGAATGGTACCCGAATCCATTTACGATCCCG

AGTTTCCAGACACATCGCACTTCCGCCCGGGTCGAGGCTGCCACTCGGCCCTAAGACGGATCAAAGAAGA

GTGGGGAACCTCTCGCTGGTTTTTGGAATTCGACATCAGGAAGTGTTTTCACACCATCGACCGACATCGA

CTCATCTCAATCTTTAAGGAAGAGATCGACGATCCCAAGTTCTTTTACTCCATTCAGAAAGTATTTTCTG

CCGGACGACTCGTAGGAGGTGAGAAGGGCCCTTACTCCGTCCCACACAGTGTACTACTATCGGCCCTACC

AGGCAACATCTACTTACACAAGCTCGATCAGGAGATAGGGAGGATCCGACAGAAGTACGAAATTCCGATT

GTTCAGAGAATCAGATTGGTTCTATTAAAGACAAGTCGTATTGATGACCAAGAAAACTCTGGAGAAGAA-

---TGGAATTCTCTCCCAGAGCTGCGGAACTAACGACTCTATTAGAAAGTAGAATTACCAACTTTTACAC

GAATTTTCAAGTGGATGAGATCGGTCGAGTGGTCTCAGTTGGAGATGGGATTGCACGTGTTTATGGATTG

AACGAGATTCAAGCTGGGGAAATGGTGGAATTTGCCAGCGGTGTGAAAGGAATAGCCTTAAATCTTGAGA

ATGAGAATGTAGGGATTGTTGTCTTTGGTAGTGATACCGCTATTAAAGAAGGAGATCTTGTCAAGCGCAC

TGGATCTATTGTGGATGTTCCTGCGGGAAAGGCTATGCTAGGGCGTGTGGTCGACGCGTTGGGAGTACCT

ATTGATGGAAGAGGGGCTCTAAGCGATCACGAGCGTCGACGTGTCGAAGTGAAAGCCCCTGGGATTATTG

AACGTAAATCTGTGCACGAGCCTATGCAAACAGGGTTAAAGGCGGTAGATAGCCTGGTTCCTATAGGCCG

TGGTCAACGAGAACTTATAATCGGGGACCGACAAACGGGAAAAACAGCTATTGCTATCGATACCATATTA

AACCAAAAGCAACTGAACTCAAGGGCCACCTCTGAGAGTGAGACATTGTATTGTGTCTATGTAGCGATTG

GACAGAAACGTTCAACTGTGGCACAATTAGTTCAAATTCTTTCAGAAGGGAATGCTTTGGAATATTCCAT

TCTTGTAGCAGCCACCGCTTCGGATCCTGCTCCTCTTCAATTTCTGGCCCCATATTCTGGGTGTGCTATG

GGAGAATATTTCCGCGATAATGGAATGCACGCATTAATAATCTATGATGATCTTAGTAAACAGGCGGTGG

CATATCGACAAATGTCATTATTGTTACGCCGACCACCAGGCCGTGAGGCTTTCCCAGGCGACGTTTTCTA

TTTACATTCTCGTCTCTTAGAAAGAGCCGCTAAACGATCGGACCAGACAGGTGCAGGTAGCTTGACCGCC

TTACCCGTCATTGAAACACAAGCTGGAGACGTATCGGCCTATATTCCCACCAATGTGATCTCCATTACAG

ATGGACAAATCTGTTTGGAAACAGAGCTCTTTTATCGCGGAATTAGACCTGCTATTAACGTCGGCTTATC

TGTCAGTCGCGTCGGGTCTGCCGCTCAGTTGAAAGCTATGAAACAAGTCTGCGGTAGTCCAAAACTTGAA

TTGGCACAATATCGCGAAGTGGCCGCCTTTGCTCAATTTGGGTCAGACCTTGATGCTGCGACTCAGGCAT

TACTCAATAGAGGTGCAAGGCTGACAGAAGTACCGAAACAACCACAATATGCACCACTTCCAATTGAAAA

ACAAATTCTAGTCATTTACGCAGCTGTCAATGGATTCTGTGATCGAATGCCACTAGATAAAATTTCTCAA

TATGAGAGAACCATTCCAAATAGTGTAAAACCAGAATTATTACAATCCCT------AAAGGGGGGCTTAA

CTAACGAAATAAAGATGGAACTAGATGAATTCTTAAAAGAATGCGCTTTGACTTACC-------------

-------GCATCCAACGCAAAGCGGCCTTTCATTCCCTTGTTTCGTCGTGGCACACCCTCCCCACAAGCA

CCCCCCGGCTCAGGGGGGACCAGAAAAGGCCTTTCGTTTTCCCCCCTTCGTCGGCCCTTGCCACCTTCCT

TAACAAGCCCTCGAGCCTCCTTTTCGCTGCCTTCCTCATAGAAGCCGCCGGGTTGACCCCGAAGGCCGAA

TTCTATGGTAGAGAACGCTGTAATAATAATTGGGCCATGAGAGACCTTTTTAAGTATTGCAAAAGAAAGG

GCCTGCTGATAGAGCTGGGCGAGGCAGCGATACTAGTTATCAGGTCAGAGAAAGGCCTGGCCCGTAAGCT

GGCCCCCTTAAAAACCCATTACTTAATAAGGATTTGTTACGCGCGATATGCCGACGACTTACTATTGGGA

ATCGTGGGTGCCGTAGAGCTTCTCATAGAAATACAAAAACGTATCGCCCACTTCCTACAATCCGGCCTGA

ACCTTTTGGTAGGCTCTGCGGGATCAACAACAATAGCTGCACGGAGTACGGTAGAATTCCTCGGTACGGT

CATTCGGGAAGTCCCTCCGAAGACGACTCCCATACAATTCTTGCGAGAGCTGGAGAAGCGTCTACGGGTA

AAGCACCGTATCCATATAACTGCTTGCCACTTACGCTCTGCCATTCATTCCAAGTTTAGGAACCTAGGTA

AGAGTATCCCGGTCAAACAGCTGACGAAGGGGATGAGCAAAACAGGGAGTCTACTGGACGCGGTTCAACT

AGCGGAAACTCTTGGAACAGCTAGAGTAAGAAGTCCCCAAGTGAGCGTATTATGGGAGACCGTCAAGCAC

ATCCGGCAAGGATCAAGGGAGATCTCGTTGTTGCATAGCTCAGGTCAGAGCAAGGTGCCATCGGACGTTC

AACAGGCAGTCTCGCGATCGGGCATGAGTGTCCGGAAGTTGTC---ATTGTATACTTCCGCGGGTCGGAA

GGCGGCGGGGGAAGGAGGGGGACACTGGGCGAGATCTATCAGCAGCGAATTCCCCATACAGATAGAAGCG

CCTATCAAAAAGATACTCCGAAGGCTTCGAGATCGAGGTCTCATTAGCCGAAGAAGACCCTGGCCAATCC

ACGTGGCCTGCTTGACGAACGTCAGCGACGGAGACATCGTAAATTGGTCCGCGGGCATCGCGATAAGTCC

TCTGTCCTACTACAGGTGCCGCGACAACCTTTACCAAGTCCGAACGATTGTCGACCACCAGATCCGCTGG

TCTGCAATATTCACCCCAGCCCACAAGCACAAATCCTCAGCGCGGAATATAATCCCAAAGTACTCAAAAG

ACTCAAATATAGTAAATCAAGAAGGTGGTAAGACCCTAGCAGAGTTCCCCAACAGCATAGAGCTTGGGAA

GCTCGGACCCGGTCAAGATCC-GAACAACAAAGAGCACTCAACTACT------------------ATGGT

CCAACTACATAACTTTTTCTTTTTCATTACTTCCATGGTCGTGCCTTGTGGCACGGCAGCACCCGTACTA

TTGAAATGGTTCGTCAGTAGAGATGTTCCCACAGGTGCCCCTTTTTCCAATGGTACTCTAATTCCTATTC

CTATCCCTTCATTCCTTCTTTTGGTCTATCTACATTCCAGGAAATTCATACGCTCCATGGACGGAGTCAA

AAGTGGAGTCTTGGTCAGAGCAAGCTGCCCTATTTTAT------TACCAGACATAATTGGGAGAAGCTCA

TCCGAAACTAGAGCTAGAAACGCCTTATTTCGTTTCGTTCCCATTCTTCATTTTCTTCTTCTCGAATCCA

A------GGGGGACTTCCCATATTTAGAATCTTTTTGCGGTGTGCTCCGTTTACTATTCTTTCGTACTTT

CTTCTCTTTACCACGCGATAGGTCAGCGAAGCGTGAGCGGGCGCGGAGAAGAAAACGGCAAA------GG

CCTAAC------GGGAATGAGCAACGACGAAATGACAAGATAAAGTG---------CCCCCATT------

TAGA---AAGAAGGGTCGAAGGGTTTGGGCCTGTAGCTTTCCCCGTCCCCCCTTCGTCGGGTGGTGCTTG

TGTGGGGGGTGTGCTACCTGAAATCGGGCTTGAAGCTCCCGCCTTACCAACGAGCCGACTGCTGATGGCT

GTTGGTCACGACTACTACCAAAAAGTGAACATGAAGATGAATATTTCACATGGAGGAGTGTGCATCTTTA

TGTTGGGTGTTCTTCTG-ACGTACATAGCTGTTCCAGCTGAAATACTTGGAATAATTCTACCACTTCTAC

TAGGAGTAGCCTTTTTAGTGCTAGCTGAACGTAAAGTAATGGCTTTTGTGCAACGTCGAAAGGGTCCTGA

TGTAGTGGGATCGTTCGGATTGTTACAACCTCTAGCAGATGGTTCGAAATTGATTCTAAAAGAACCTATT

TCACCAAGTAGTGCTAATTTCTCCCTTTTTAGAATGGCTCCAGTCACTACATTTATGCTAAGTCTGGTTG

CTCGGGCCGTTGTACCTTTTGATTATGGTATGGTATTGTCAGATCCGAACATAGGGCTACTTTATTTGTT

TGCCATATCTTCGCTAGGTGTTTATGGAATTATTATAGCAGGTTGGTCTAGT--TTATTATATACGTTTA

GTGAAAAGAATGTTTTTTGATACACCTAGGACATGGATTCTATATGAACCAATGGATCGTGACAAGTCGT

TACTACTAGCAATGACTTCCTCTTTCATTACTTCATCCTTTCCATATCCTTCTCCCTTGTTCTCAGTTAC

TCATCAAATGGCACTCAGTTTATATCTTTAA-TGTCAGAATTTGCGCCGATTTGTATCTATTTAGTGATC

AGTCTGCTAGTTTCTTTGATCTTACTCGGAGTTCCTTTTCCATTTTCTTCTAATAGTTCGACTTATCCAG

AAAAATTGTCGGCCTACGAATGTGGTTTCGATCCTTTCGGTGATGCCAGAAGTCGTTTCGATATACGATT

TTATCTTGTTTCAATTTTATTTATTATCCTTGATCTGGAAGTCACCTTTTTCTTTCCTTGGGCAGTACCT

CTCAACAAGATTGATCCGTTTGGATTTTGGTCTATGATGGCCTTTTTATTGATTTTAACGATTGGATTTC

TCTATGAATGGAAAAGGGGTGCTTTGGATTGGGAGTAA-AAAGTGTTTATTACGATTACGCCCAACAGCC

CACTTGAGCAATTTTCCATTCTCCCATTGATTCCTATGAAAATAGGAAACTTGTATTTCTCATTCACAAA

TCCATCTTTGTTTATGCTGCTAACTCTCAGTTTGGTCCTACTTCTGCTTCATTTTGTTACTAAAAACGGA

GGAGGAAACTCAGTACCAAATGTTTGGCAATCCTTGGTAGAGCTTATTTATGATTTCGTGCTGAACCTGG

TAAACGAACAAATAGGTGGTCTTTCCGGAAATGTTAAACAAAAGTTTTTCCCTTGCATCTTGGTCACTTT

TACTTTTTTGTTATTTCGTAATCTCCAGGGTATGATACCCTATAGCTTTACAGTTACAAGTCATTTTCTC

ATTACTTTGGGTCTTTCATTTTCCATTTTTATTGGCATTACTATAGTGGCATTTCAAAGAAATGGGCTTC

ATTTTTTAAGCTTCTTCTTACCTGCAGGAGTCCCACTGCCGTTAGCACCTTTTTTAGTACTCCTTGAGCT

AATCCCTCATTGTTTTCGCGCATTAAGCTCAGGAATACGTTTATTTGCTAATATGATGGCCGGTCATAGT

TCAGTAAAGATTTTAAGTGGGTTCGCTTGGACTATGCTATGTATGAATGATCTTTTCTATTTCATAGGAG

ATCTTGGTCCTTTATTTATAGTTCTTGCATTAACCGGTCTTGAATTAGGTGTAGCTATATTACAAGCTCA

TGTTTTTACGATCTTAATCTGTATTTACTTGAATGATGCTACAAATCTCCATCAAA--------------

-----------------TATGTGGGCACCTGATATCTATGAGGGTTCACCCACCCCGGTTACAGCATTCT

TTTCTATTGCGCCTAAAATTTCCATTTCTGCAAATATTTTACGTGTTTTTATTTATGGTTCCTATGGAGC

TACATTGCAACAAATCTTCTTTTTCTGCAGCATTGCTTCTATGATCTTAGGAGCACTGGCCGCCATGGCC

CAAACGAAAGTCAAAAGACTTCTAGCTCATAGTTCAATTGGACATGTAGGTTATATTCGTACTGGTTTCT

CATGTGGAACCATAGAAGGAATTCAATCACTACTAATTGGTCTCTTTATTTATGCATCAATGACGATAGA

TGCATTCGCTATAGTTTCAGCATTACGGCAAACCCGTGTCAAATATATAGCGGATTTGGGCGCTCTAGCC

AAAACGAATCCTATTTCGGCTATTACCTTCTCTATTACTATGTTCTCATACGCAGGAATACCCCCGTTAG

CCGGCTTTTGTAGTAAATTCTATTTGTTCTTCGCCGCTTTGGGTTGTGGGGCTTACTTCCTAGCCCCAGT

GGGAGTAGTGACTAGCGTTATAGGTTGTTTGTTCGATAGCCCGACCGTAGTGATGTTAATTGTGGTTACA

TTCATAAGTAGCTTGGTCCATCTTTATTCTATTTCATATATGTCCGAGGATCCGCATAGCCCTCGATTTA

TGTGTTATTTATCCATTCTTACTTTTTTTATGCCAATGTTGGTGACTGGAGATAACTCTCTTCAATTATT

CTTGGGATGGGAGGGAGTAGGTCTTGCTTCATATTTGTTAATTCATTTTTGGTTTACACGACTTCAGGCA

GATAAAGCAGCTATAAAAGCTATGCTTGTCAATCGAGTAGGTGATTTTGGATTAGCTCTTGGGATTTCGG

GTCGTTTTACTCTCTTTCAAACAGTAGACTTTTCTACCATTTTTGCTTGTGCTAGTGCCCCTAGAAATTC

TTGGATTTTTTGCAATATGAGATTGAATGCCATAACTCTTATTTGTATTTTACTTTTTATTGGTGCTGTT

GGAAAATCTGCACAGATAGGATCGCATACTTGGTCACCCGATGCTATGGAGGGTCCCACTCCAGTATCTG

CTTTGATTCATGCAGCTACTATGGTAACAGCTGGCGTTTTCATGATAGCAAGGTGTTCCCCTTTATTTGA

ATACCCACCTACGGCTTTAATTGTTATTACTTTTGCAGGAGCTATGACGTCATTCCTTGCGGCAACCACT

GGAATATTACAGAACGATCTAAAGAGGGTCATAGCTTATTCAACTTGCAGTCAATTAGGCTATATGATCT

TTGCTTGCGGCATTTCTAACTATTCGGTTAGCGTCTTTCATTTAATGAATCACGCCTTTTTCAAAGCATT

ACTATTCCTGAGTGCAGGTTCGGTGATTCATGCCATGTCGGATGAGCAAGATATGCGGAAGATGGGGGGG

CTCGCCTCCTCGTTCCCTTTTACCTATGCCATGATGCTCATGGGCAGCTTATCTCTAATTGGATTTCCTT

TTCTAACTGGATTTTATTCCAAAGATGTGATCTTAGAGCTCGCTTACACTAAGTATACCATCAGTGGGAA

CTTTGCTTTCTGGTTGGGAAGTGTCTCTGTCCTTTTCACTTCTTATTACTCCTTTCGTTCACTTTTTCTA

ACATTTCTAGTACCAACTAATTCATTCGGGCGAGACATCTTACGATGTCATGATGCGCCCATTCCTATGG

CCATTCCTTTAATACTTCTGGCTTTCGGGAGTCTCTTTGTAGGATACTTGGCCAA-CTAACACAAAGAAG

ATACAGTTCACTCAACGATTGCCTTTGGGTTCCGAACTCCATATGGGGAAGGAACGTTGTTGTTTGCGGG

GTCTCGATCATTTACATGGACCCACTTTTCATTCCATTTGTGGGAATTTGATGATCTATAAACCGTCCTT

AACGAACGATCGGCTCATCTT------TGAGCATGATGAATCACTTCGTGCCGACCTGTTGTCAATAAAC

TTTTTGGCCTCATATGAGAATGGAAAACTGGAGCATTTTCTTCATCGGTGGATGAAGAATCGCGAACATA

ATAATTTCTGGTTAAGCATGTTCCCAGAAAAAAGATACTTTCGAGAAACAACGAGCACGACTGAAGTGGC

TATACATACAAATCCATTTACGGATCTATATGCTTCGATTGGAACTGGAAGTTCAAGAACAGGCGGCTGG

TATACTACCATAATGAAACTGCCTTTTATTTTTTTTATTCGGATAGGATTTCTGTTGGCTTCGTTGGGAG

GCTCGCGTAGTTTGTTACGTCAGCTCCAAAAGGAAAAATTGCGTTGGAATCGAGAAAGTTACGTAA----

AGTTCATAATTGTATA-CCAATTTTTGGGCCAATTCCCTCTTCGTACTACCAAAAAATGAGATTCTTGCC

GAATCCGAGTTTGCTGCTCCAACCATTACCAAACTAATACCTATTCTGTTTAGTACTTCAGGTGCTTCTG

TTGCGTATAATGTAAATCCCGTAGCGGATCAATTCC---------------AACGAGCCTTTCAAACTAG

TACTTTTTGTAATCGACTCTATAGCTTCTTCAATAAACGCTGGTTCTTCGATCAAGTTTTGAATGACTTT

CTAGTCAGATCGTTCTTGCGTTTCGGATATGAAGTCTCATTCGAAGCTTTAGACAAAGGTGCTATTGAGA

TATTGGGCCCCTATGGTATCTCCTACACATTCCGACGATTGGCCGAGCGAATAAGTCAACTTCAAAGTGG

ATTTGT--TGTTCCATGATCTATGGGTCTACTGGAGCTACCCATTTCGATCAATTAGCCAAGATTTTGAC

CGGATACGAAATCACTGGTGTTCAATCTAGTGGTATTTTTATGGGGATTCTTTTTATCGCTGTAGGATCC

CTATTCAAGATCACTGCAGTTCCTTTTC-GGTCTATGCACATTGCTTTCTCCAGGAGGTTGGCCGCCTAT

CCTAGATCTTCCCATTTCCAAGAGGATCCCGGGCTCAATCTGGTTTAGTATCAAGGTGATTCTCTTTCTC

TTTCTATATATATGGGTCCGTGCAGCATTTCCACGATATCGTTATGATCAATTAATGGGACTTGGCCGGA

AAGTGTTCTTGCCT----------CTCGGGTAGTCGCCGTTTCTGGTGTTTTAGTCACCTTTCAATGGCT

CCCTTAAATGCCTCAACTGGATAAATTTACTTATTTCACACAATTCTTCTGGTCATGCCTTTTCTTCTTT

ACTTTCTATATTTTAATATGCAATGATAGAGATGGAGTACTTGGGATCAGCAGAATTCTAAAACTACGAA

ACCAACTGCTTTCACACCGGGGGAACAACATCCAAA------GCAAGGACCCCAACAGTTTGGAAGATAT

CTTGAGAAAAGGTTTTCACACAGGTGTATCCTATATGTACTCTAGTTTATTCGAAGTATCCCAATGGTGT

AAGTCCGTCGACTTATTGGGAAAAAGGAAGAAAATCACTTTGATCTCTTCTTTCGGAGAAATAAGTGGCT

CGCGAGGAATGGAAAGAAACATATTTTATTTGATCTCGAAGTCTTCATATAGCACTTT------------

---TTCCAATCATGGATGGGGGATCACTTGTAAGAATGACATAATGCTAATCCATGTTCTACACGGCCAA

GGA--------------------

>Sesuvium portulacastrum MN683736.1

-TGATACTTTCTGTTTTGTCGAGCCCGGCTTTGGTCTCTGGTTTGATGGTTGTACGTGCTAAAAATCCGG

TACATTCCGTTTCGTTTCCCATCCCAGTCTTTCGCAACACTTCAGGTTTACTTCTTTTGTTAGGTCTCGA

CTTTTCCGCTATGATCTTCCCAGTAGTTTATATAGGAGCTATAGCCGTTTCATTCCTATTCGTTGTTATG

ATGTTCCATATTCAAATAGCGGAGATTCACGAAGAAGTATTGCGCTATTTACCAGTGAGTGGTATTATTG

GACTGATCTTTTGGTGGGAAATGTTCTTCATTTTAGATAATGAAACCATTCCATTACTACCAACCCAAAG

AAATACGACCTCTCTGAGATATACGGTTTATGCCGGAAAGGTACGAAGTTGGACTAATTTGGAAACATTG

GGCAATTTACTTTATACTTACTATTTTGTCTGGTTTTTGGTTTCGAGTCTTATTTTATTAGTAGCCATGA

TTGGGGCTATAGTACTGACTATGCATAGGACTACTAA------GGTGAAAAGACAGGATGTATTCCGACG

AAATGCTATTGATTCTAGAAGGACTATAATGAGGAGGACGACAGACC-ATGTCAATATATGAATTGTTTC

ATTATTCGTTATTTCCGGGTCTTTTCATTGCATTCACTTACAACAAAAAACAACCACCAGCGTTTGGTGC

AGCACCTGCATTTTGGTGTATTCTTCTTTCTTTCCTTGGTCTTTTGTTCTGTCATATTCCTAATAACTTA

TCCAATTACAACGTATTAACCGCTAATGCACCTTTCTTTTATCAAATCTCAGGGACATGGTCTAATCATG

AAGGTAGTATTTTATTATGGTGTCGGATCCCAAGTTTTTATGGATTCTTTCTTTGTTACCGGGGTCGATC

CCAAAGCCATAATGTCTCAAAACGAGGAGGCCATAGAGAAAGTCTTATTTTTTCCTTTGTCTTAAACTTC

GTGAAGAACTCCATTCTATCTCTTCCTCGTTACGAACAAAAAAGTAGAGTTCTTCACAAACCCCAGTTGT

ACACTCTCTTCGTTCTACGAA---CTCTTGTTGATTCTGAACTTTGTTCGCGAAGGAACCGGACTTTTGA

CGGGCCAGCTCTTTTTTACGCGCCGCTTTACCCTGAAAGGAAAATGAGCTTTGCTCTTCTGGGCGCTAGG

CGCTCTCGTGGTTCGCGAGAAGGAAAAAGGACTCATCCTTTGTTGCATCTGGCACGAGATGATAAAGAGA

GAGCTTCGTCTATCGATGAACAGCGGATTGACGGAGCTCTTGGCATTGCTTTGTTTTTCTTTCCTTTCCT

ATCAGCGAGTTCCGATCCTTTTGTTCGAAATTTCTTCGTTTGTACCGAACCGCTTGCAGAATCAAATCCT

GTTCCACAAGATCCTATATCAGCTATACATCCTCCTTGCATTTATGCCGGAGACGTCGCCAGTGCTATGG

GCTTTGGCTTATGTAGATCAAAAATGATGAATGGGATTGTGGCACTCCACTCGCCGCCAATGCGGAAGGA

TGTCGCCGAAAAGAATGGAACGCTGCTTTGCTCTGCTGGATGCGTCGGATCCCGTATAACAAGCGAGCTC

TTTACCCTTAAATTCAAACATGTGGGCGCCAAATGCTATCCTGCTTTATTGTTGCGTAGCAATAGAAGCC

TGCTC---ATGCTGCTTCGGCGGCGCTTTTTCGCCTTCTCTTCGCTCTGGATAAGAGCGCTAGCGGACAC

GGGGAGGGAGCG------GGCGAAGCGTTTCT------TTCGTAATGGAAAGAAAGATACCACTACTTCG

CCTCTTTGTTGGACCGCCGGCGCGAACACAGTGGTCTCTGACCAGGACCAGGAACCAATTCGAATTTGGA

TCTTGATATGTCGGTGCTTTTTAACCGTAGGCATCTTGCCAGGAAGTTGGTGGGCTCATCATGAATTAGG

TCGGGGTGGCTGGTGGTTTCGGGATCCCGTAGAAAATGCTTCTTTTATGCCTCGGGTATTAGCCACAGCT

CGTATTCATTCAGTAATTTTACCCCTTCTTCATTCTTGGACTTTGCTTCTTAATATTGTGACTTTTCTAT

GCTGTGTCTTAGGAACCTTTTCAATACGGTCCGGATTGCTAGCTCCCGTTCATAGTTTTGCTACAGATGA

TACACGAGGAATCTTTTTATGGCGGTTCTTCCTTCTAATGACCGGTATCTCTATGATTCTTTTCTCTCAG

ATGAAGCAGCAGGCATCGGTCCGTAGAACCTATAAAAAAGAGATGGTTGTAGCGCGAAGTACTCTTGTGC

ACTTACG----TGATTGTTCGAAAATGGCTATTCTTCCCAATTTCTCCTTGTGATGCAGCGGAACCATGG

CAATTAGGATTTCAAGACGCAGCAACACCTATGATGCAAGGAATAATAGACTTACATCATGATATCTTTT

TCTTCCTCATTCTTATTTTGGTTTTCGTATCATGGATCTTGGTTCGCGCTTTATGGCATTTCCACTATAA

AAAAAATCCAATCCCGCAAAGGATTGTTCATGGAACTACTATCGAGATTATTCGGACCATATTTCCTAGT

ATCATCCTGATGTTCATTGCTATACCATCATTTGCTCTGTTATACTCAATGGACGAGGTAGTAGTAGATC

CAGCCATTACTATCAAAGCTATTGGACATCAATGGTATTGGACTTATGAGTATTCGGACTATAACAGTTC

CGATGAACAGTCACTCACTTTTGACAGTTATATGATTCCAGAAGATGATCTAGAATTGGGTCAATCACGT

TTATTAGAAGTGGACAATAGAGTGGTTGTACCAGCAAAAAGTAATATACGTATTATTGTAACATCTGCTG

ATGTACTTCATAGTTGGGCTGTACCTTCCTCAGGTGTCAAATGTGATGCTGTACCTGGTCGTTTAAATCA

GACCTCTATTTTGGTACAACGAGAAGGAGTTTACTATGGTCAGTGCAGTGAAATTCGTGGAACTAATCAT

GCTTTTATGCGT----------------------------------------------------------

------------------------TGAGACGACTCTTTTTTGAACTATATCATAAACAGATCTTCTTCTC

CACACCAATCACGAGTTTTTCTCTATTCCTCTCGTATATTGTCGTAACGCCCTTAATGCTAGGTTTTGAA

AAAGACTTTTCATGTCATTTCCATTTAGGTCCGATTCGGATCCCTCCGTTGTTTCCTTTTCCTCCCGCAC

CTTTTCTTCGAAATGAGAAAGAAGATGGTACACTCGAATTGTATTATTTAAGTGCTTATTGCTTGCCAAA

GATCCTACTTCTACAATTGGTAGGTCACCGGGTTATTCAAATAAGTCGTGTTTTCTGTAGTTTTCCCATG

TTACAACTTCTGTACCAATTCGGTCAATCCGGAATGGATCGGTTAAACATTCTATTAGGGAGCCTGGTCT

TGACTCTTCTGTGTGGTATTCATTCTTGTTTGGCTCTTGGAATCACATCCAGCAGTGGTTGGAACAGCTC

GCAAAATTTAACCACTTCACCTACTTCATTGCCCTCAACCGTTTCTCGTACCTCTATTGAAACAGAATGG

TTTCATGTTCTTTCATCGATTGGTTATTTTTCTTCGTTCGTCTCTCTTTTTCCAATTTCGGTCTCGATTA

GTTCACAAGATTGAATGTCCGTTTTGTTATTACAACCTTC-----TTTTTTGATGTCAAAGACCAGAAGC

TACGCGCAAATTCTCATTGGATCTTGGTTGTTCTTAACAGCGATGGCTATTCATTTAAGTCTTTGGGTAG

CACCACTAGATCTTCAACAAGGTGGAAATTCTCGTATTCTCTATGTACATGTTCCTGTGGCTCGGATGAG

TATTCTTGTTTATATCGTTACGGCTATAAACACTTTCTTGTTCCTATTAACAAAACATCCTCTTTTTCTT

CGCTCTTCCGGAACCGGTACAGAAATGGGTGCTTTTTCTACGTTGTTTACCTTAGTTACTGGGGGGTTTC

GGGGAAGACCCATGTGGGGCACCTTTTGGGTGTGGGATGCTCGTTTAACTTCTGTATTCATCTCGTTCCT

TATTTACCTGGGTGCACTGTGTTTTCAAAAGCTTCCTGTCGAACCGGCTCCTATTTCAATCCGTGCTGGA

CCGATCGATATACCAATAATCAAGTCTCCTGTCAACTGGTGGAATACATCGCATCAACCTGGGAGCATTA

GCCGATCTGGTACATCAATACATGTTCCTATGCTCATTCCAATCTTGTCTAACTTTGCTAACTTCCTCTT

CTCAACCCGTATCTTGTTTGTTCTGGAAACACGTCTTCCTATTCCATCTTTTCTCGAATCTCCTTTAACG

GAAGAAATAGAAGCTCGAGAAGGAATA-------------AAGATCCTATTTGCTGCTATTCTATCGATT

TGTGCATTAAGTTCGAAGAAGATCTCAATCTATAATGAAGAAATGATAGTAGCTCTTTGTTTTATAGGCT

TTATCATATTAAGTCGGAAGAGTTTAGGTAAGACTTTCAAAGTGACTCTCGACGAGAGAATCCAGGCTAT

TCAGGAAGAATCGCAGCAATTCCCCAATCCTAACGAAGTAGTTCCTCCGGAATCCAATGAACAACAACGA

TTACTTAGGATCAGCTTGCGAATTTGTGGAACCGTAGTCGAATCATTACCAATGGCACGCTGTGCGCCTA

AGTGCGAAAAGACAGTGCAAGCTTTGTTATGTCGAAACCTAAATGTTAAGTCAGCAACTCTTCCAAATGC

CACTTCTTCCCGTCGCACCCGTCTTCAGGACGATCTAGTCACAGGGTTTAACTTCTCAGTGAGTGAAAGA

TTTGTCCCCGGGTCTACGTTGAAAGCTTCTATAGTAGAACTCATTCGAGAAGGCTTGGCGGTCTTAAGAA

TGGTTCGGGTAG-------------TGAAAGAGGCGATCAGAATGGTACCCGAATCCATTTACGATCCCG

AGTTTCCAGACACATCGCACTTCCGCTCGGGTCGAGGCTGCCACTCGGCCCTAAGACGGATCAAAGAAGA

GTGGGGAACCTCTCGCTGGTTTTTGGAATTCGACATCAGGAAGTGTTTTCACACCATCGACCGACATCGA

CTCATCCCAATCTTTAAGGAAGAGATCGACGATCCCAAGTTCTTTTACTTCATTCAGAAAGTCTTTTCTG

CCGGACGACTCGTAGGAGGTGAAAAGGGCCCTTACTCCGTCCCACACAGTGTACTACTATCGGCCCTACC

AGGCAACATCTACTTACACAAGCTCGATCAGGAGATAGGGAGGATCCGACAGAAGTACGAAATTCCGATT

GTTCAGAGAATCAGATCGGTTCTATTAAAGACAAGTCGTATTGATGACCAAGAAAACTCTGGAGAAGAAG

---TGGAATTCTCCCCCAGAGCTGCGGAACTAACGACTCTATTAGAAAGTAGAATTACCAACTTTTACAC

GAATTTTCAAGTGGATGAGATCGGTCGAGTGGTCTCAGTTGGAGATGGGATTGCACGTGTTTATGGATTG

AACGAGATTCAAGCTGGGGAAATGGTGGAATTTGCCAGCGGTGTGAAAGGAATAGCCTTAAATCTTGAGA

ATGAGAATGTAGGGATTGTTGTCTTTGGTAGTGATACCGCTATTAAAGAAGGAGATCTTGTCAAGCGCAC

TGGATCTATTGTGGATGTTCCTGCGGGAAAGGCTATGCTAGGGCGTGTGGTCGACGCGTTGGGAGTACCT

ATTGATGGAAGAGGGGCTCTAAGCGATCACGAGCGTCGACGTGTCGAAGTGAAAGCCCCTGGGATTATTG

AACGTAAATCTGTGCACGAGCCTATGCAAACAGGGTTAAAGGCGGTAGATAGCCTGGTTCCTATAGGCCG

TGGTCAACGAGAACTTATAATCGGGGACCGACAAACGGGAAAAACCGCTATTGCTATCGATACCATATTA

AATCAAAAGCAACTGAACTCAAGGGCCACCTCTGAGAGTGAGACATTGTATTGTGTCTATGTAGCGATTG

GACAGAAACGTTCAACTGTGGCACAATTAGTTCAAATTCTTTCAGAAGGGAATGCTTTGGAATATTCTAT

TCTTGTAGCAGCCACCGCTTCGGATCCTGCTCCTCTTCAATTTCTGGCCCCATATTCTGGGTGTGCTATG

GGAGAATATTTCCGCGATAATGGAATGCACGCATTAATAATCTATGATGATCTTAGTAAACAGGCGGTGG

CATATCGACAAATGTCATTATTGTTACGCCGACCACCAGGCCGTGAGGCTTTCCCAGGCGACGTTTTCTA

TTTACATTCTCGTCTCTTAGAAAGAGCCGCTAAACGATCGGACCAGACAGGTGCAGGTAGCTTGACCGCC

TTACCCGTCATTGAAACACAAGCTGGAGACGTATCGGCCTATATTCCCACCAATGTGATCTCCATTACTG

ATGGACAAATCTGTTCGGAAACAGAGCTCTTTTATCGCGGAATTAGACCTGCTATTAACGTCGGCTTATC

TGTCAGTCGCGTCGGGTCTGCCGCTCAGTTGAAAGCTATGAAACAAGTCTGCGGTAGTCCAAAACTGGAA

TTGGCACAATATCGCGAAGTGGCCGCCTTTGCTCAATTTGGGTCAGACCTTGATGCTGCGACTCAGGCAT

TACTCAATAGAGGTGCAAGGCTTACAGAAGTACCGAAACAACCACAATATGCACCACTTCCAATTGAAAA

ACAAATTCTAGTCATTTACGCAGCTGTCAATGGATTCTGTGATCGAATGCCACTAGATAAAATTTCTCAA

TATGAGAGAACCATTCCAAATAGTGTAAAACCAGAATTATTACAATCCCT------AAAGGGCGGCTTAA

CTAACGAAAAAAAGATGGAACTAGATGCATTCTTAAAAGAATGCGCTTTGACTTACC-------------

--------CATCCAACGCAAAGCGGCCTTTCATTCCCTTGTTTCGTCGTGGCACACCCTCCCCACAAGCA

CCCCCCGGCTCAGGGGGGACCAGAAAAGGCCTTTCGTTTTCCCCCCTTCGTCGGCCCTTGCCACCTTCCT

TAACAAGCCCTCGAGCCTCCTTTTCGCTGCCTTCCTCATAGAAGCCGCCGGGTTGACCCCGAAGGCCGAA

TTCTATGGTAGAGAACGCTGTAATAATAATTGGGCCATGAGAGACCTTTTTAAGTATTGCAAAAGAAAGG

GCCTGCTGATAGAGCTGGGCGAGGCAGCGATACTAGTTATCAGGTCAGAGAAAGGCCTGGCCCGTAAGCT

GGCCCCCTTAAAAACCCATTACTTAATAAGGATTTGTTACGCGCGATATGCCGACGACTTACTATTGGGA

ATCGTGGGTGCCGTAGAGCTTCTCATAGAAATACAAAAACGTATCGCCCACTTCCTACAATCCGGCCTGA

ACCTTTGGGTAGGCTCTGCGGGATCAACAACAATAGCTGCACGGAGTACGGTAGAATTCCTCGGTACGGT

CATTCGGGAAGTCCCTCCGAAGAGGACTCCCATACAATTCTTGCGAGAGCTGGAGAAGCGTCTACGGGTA

AAGCACCGTATCCATATAACTGCTTGCCACTTACGCTCTGCCATTCATTCCAAGTTTAGGAACCTAGGTA

ATAGTATCCCGGTCAAACAGCTGACGAAGGGGATGAGCAAAACAGGGAGTCTACTGGACGCGGTTCAACT

AGCGGAAAGTCTTTCCACAGCTAGAGTAAGAAGTCCCCAAGTGAGCGTATTATGGGAGACCGTCAAGCAC

ATCCGGCAAGGATCAAGGGAGATCTCGTTGTTGCATAGCTCAGGTCAGAGCAAGGTGCCATCGGACGTTC

AACAGGCAGTCTCGCGATCGGGCATGAGTGTCCGGAAGTTGTC---ATTGTATACTCTCGCGGGTCGGAA

GGCGGCGGGGGAAGGAGGGGGACACTGGGCGAGATCTATCAGCAGCGAATTCCCCATACAGATAGAAGCG

CCTATCAAAAAGATACTCCGAAGGCTTCGAGATCGAGGTATCATTAGCCGAAGAAGACCCTGGCCAATCC

ACGTGGCCTGCTTGACGAACGTCAGCGACGGAGACATCGTAAATTGGTCCGCGGGCATCGCGATAAGTCC

TCTGTCCTACTACAGGTGCCGCGACAACCTTTACCAAGTCCGAACGATTGTCGACCACCAGATCCGCTGG

TCTGCAATATTCACCCCAGCCCACAAGCACAAATCCTCAGCGCGGAATATAATCCCAAAGTACTCCAAAG

ACTCAAATATAGTAAATCAAGAAGGTGGTAAGACCCTAGCAGAGTTCCCCAACAGCATAGAGCTTGGGAA

GCTCGGACCCGGTCAAGATCC-GAACAACAAAGAGCACTCAACTACTA-----------------ATGGT

CCAACTACATAACTTTTTCTTTTTCATTACTTCCATGGTCGTGCCTTGTGGCACGGCAGCACCCGTACTA

TTGAAATGGTTCGTCAGTAGAGATGTTCCCACAGGTGCCCCTTTTTCCAATGGTACTATAATTCCTATTC

CTATCCCTTCATTCCTTCTTTTGGTCTATCTACATTCCAGGAAATTCATACGCTCCATGGACGGAGTAAA

AAGTGGAGTCTTGGTCAGAGCAAGCTGCCCTATTTTAT------TACCAGACATAATTGGGAGAAGCTCA

TCCGAAACTAGAGCTAGAAACGCCTTATTTCGTTTCGTTCCCATTCTTCATTTTCTTCTTCTCGAATCCA

A------GGGGGACTTCCCATATTTAGAATCTTTTTGCGGTGTGCTCCGTTTACTATTCTTTCGTACTTT

CTTCTTTTTACCACGCGATAGGTCAGCGAAGCGTGAGCGGGCGCGGAGAAGAAAACGCCAAACACTTCGG

CCTAAC------GGGAATGAGCAACGACGAAATGACAAGAGAAAGTGCCCCGGGCGCCCCCATT------

TAGA---AAGAAGGGTCGAAGGGTTTGGGCCTGTAGCTTTCCCTGTCCCCCCTTCGTCGGGTGGTGCTTG

TGTGGGGGGTGTGCTACCTGAAATCGGGCTTGAAGCTCCCGCCTTACCAACGAGCCGACAGCTGATGGCT

GTTGGTCACGACTACCACCAAAAAGTGAACATGAAGATGCATATTTCACATGGAGGAGTGTGCATCTTTA

TGTTGGGTGTTCTTCTG-ACGTACATAGCTGTTCCAGCTGAAATACTTGGAATAATTCTACCACTTCTAC

TAGGAGTAGCCTTTTTAGTGCTAGCTGAACGTAAAGTAATGGCTTTTGTGCAACGTCGAAAGGGTCCTGA

TGTAGTGGGATCGTTCGGATTGTTACAACCTCTAGCAGATGGTTCGAAATTGATTCTAAAAGAACCTATT

TCACCAAGTAGTGCTAATTTCTCCCTTTTTAGAATGGCTCCAGTCACTACATTTATGCTAAGTCTGGTTG

CTCGGGCCGTTGTACCTTTTGATTATGGTATGGTATTGTCAGATCCGAACATAGGGCTACTTTATTTGTT

TGCCATATCTTCGCTAGGTGTTTATGGAATTATTATAGCAGGTTGGTCTAGT--TTATTATATACGTTTA

GTGAAAAGAATGTTTTTTGATACACCTAGGACATGGATTCTATATGAACCAATGGATCGTGACAAGTCGT

TACTACTAGCAATGACTTCCTCTTTCATTACTTCATCCTTTCCATATCCTTCTCCCTTGTTCTCAGTTAC

TCATCAAATGGCACTCAGTTTATATCTTTAAATGTCAGAATTTGCGCCTATTTGTATCTATTTAGTGATC

AGTCTGCTAGTTTCTTTGATCTCACTCGGTGTTCCTTTTCCATTTTCTTCTAATAGTTCGACTTATCCAG

AAAAATTGTCGGCCTACGAATGTGGTTTCGATCCTTTCGGTGATGCCAGAAGTCGTTTCGATATACGATT

TTATCTTGTTTCAATTTTATTTATTATCCTTGATCCGGAAGTCACCTTTTTCTTTCCTTGGGCAGTACCT

CTCAACAAGATTGATCCGTTTGGATCTTGGTCCATGATGGCCTTTTTATTGATTTTAACGATAGGATTTC

TCTATGAATGGAAAAGGGGTGCTTCGGATCGGGAGTA------------------------CCAACAGCC

CACTTGAGCAATTTGCCATTCTCCCATTGATTCCTATGAAAATAGGAAACTTGTATTTCTCATTCACAAA

TCCCTCTTTGTTTATGCTGCTAACTCTCAGTTTGGTCCTACTTCTGCTTCATTTTGTTACTAAAAACGGA

GGAGGAAACTCAGTACCAAATGTTTGGCAATCCTTGGTAGAGCTTATTTATGATTTCGTGCTGAACCTGG

TAAACGAACAAATAGGTGGTCGTTCCGGAAATGTGAAACAAAAGTTTTTCCCTTGCATCTTGGTCACTTT

TACTTTTTTGTTATTTCGTAATCTCCAGGGTATGATACCCTATAGCTTTACAGTTACAAGTCATTTTCTC

ATTACTTTGGGTCTTTCATTTTCCATTTTTATTGGCATTACTATAGTGGGATTTCAAAGAAATGGGCTTC

ATTTTTTAAGCTTCTTATTACCTGCAGGAGTCCCACTGCCGTTAGCACCTTTTTTAGTACTCCTTGAGCT

AATCCCTCATTGTTTTCGCGCATTAAGCTCAGGAATACGTTTATTTGCTAATATGATGGCCGGTCATAGT

TCAGTAAAGATTTTAAGTGGGTTCGCTTGGACTATGCTATGTATGAATGATCTTTTATATTTCATAGGAG

ATCTTGGTCCTTTATTTATAGTTCTTGCATTAACCGGTCTTGAATTAGGTGTAGCTATATTACAAGCTCA

TGTTTTTACGATCTTAATCTGTATTTACTTGAATGATGCTACAAATCTCCATCAAA--------------

-----------------TATGTGGGCACCTGATATCTATGAGGGTTCACCCACCCCGGTTACAGCATTCT

TTTCTATTGCGCCTAAAATTTCGATTTCTGCTAATATTTTACGTGTTTTTATTTATGGTTCCTATGGAGC

TACATTGCAACAAATCTTCTTTTTCTGCAGCATTGCTTCTATGATCTTAGGAGCACTGGCCGCCATGGCC

CAAACGAAAGTAAAAAGACTTCTAGCTCATAGTTCAATTGGACATGTAGGTTATATTCGTACTGGTTTCT

CATGTGGAACCATAGAAGGAATTCAATCACTACTAATTGGTCTCTTTATTTATGCATTAATGACGATAGA

TGCATTCGCTATAGTTTCAGCATTACGGCAAACCCGTGTCAAATATATAGCGGATTTGGGCGCTCTAGCC

AAAACGAATCCTATTTCGGCTATTACCTTCTCTATTACTATGTTCTCATACGCAGGAATACCCCCGTTAG

CCGGCTTTTGTAGTAAATTCTATTTGTTCTTCGCCGCTTTGGGTTGTGGGGCTTACTTCCTAGCCCCAGT

GGGAGTAGTGACTAGCGTTATAGGTTGTTTGTTCGATAGCCCGACCGTAGTGATGTTAATTGTGGTTACA

TTCATAAGTAGCTTGGTCCATCTTTATTCTATTTCATATATGTCTGAGGATCCGCATAGCCCGCGATTTA

TGTGTTATTTATCCATTCTTACTTTTTTTATGCCAATGTTGGTGACTGGAGATAACTCTCTTCAATTATT

CTTGGGATGGGAGGGAGTAGGTCTTGCTTCATATTTGTTAATTCATTTTTGGTTTACACGACTTCAGGCA

GATAAAGCAGCTATAAAAGCTATGCTTGTCAATCGAGTAGGTGATTTTGGATTAGCTCTTGGGATTTCGG

GTCGTTTTACTCTCTTTCAAACAGTAGACTTTTCTACCATTTTTGCTTGTGCTAGTGCCCCTAGAAATTC

TTGGATTTCTTGCAATATGAGATTGAATGCCATAACTCTTATTTGTATTTTACTTTTTATTGGTGCTGTT

GGAAAATCTGCACAGATAGGATCGCATACTTGGTCACCCGATGCTATGGAGGGTCCCACTCCAGTATCTG

CTTTGATTCATGCAGCTACTATGGTAACAGCTGGCGTTTTCATGATAGCAAGGTGTTCCCCTTTATTTGA

ATACCCACCTACGGCTTTAATTGTTATTACTTTTGCAGGAGCTATGACGTCATTCCTTGCGGCAACCACT

GGAATATTACAGAACGATCTAAAGAGGGTCATAGCTTATTCAACTTGCAGTCAATTAGGCTATATGATCT

TTGCTTGCGGCATTTCTAACTATTCGGTTAGCGTCTTTCATTTAATGAATCACGCCTTTTTCAAAGCATT

ACTATTCCTGAGTGCAGGTTCGGTGATTCATGCCATGTCGGATGAGCAAGATATGCGGAAGATGGGGGGG

CTCGCCTCCTCGTTCCCTTTTACCTATGCCATGATGCTCATGGGCAGCTTATCTCTAATTGGATTTCCTT

TTCTAACTGGATTTTATTCCAAAGATGTGATCTTAGAGCTCGCTTACACTAAGTATACCATCAGTGGGAA

CTTTGCTTTCTGGTTGGGAAGTGTCTCTGTCCTTTTCACTTCTTATTACTCCTTTCGTTCACTTTTTCTA

ACATTTCTAGTACCAACTAATTCATTCGGGCGAGACATCTTACGATGTCATGATGCGCCCATTCCTATGG

CCATTCCTTTAATACTTCTGGCTTTCGGGAGTCTCTTTGTAGGATACTTGGCCAA-CTAACACAAAGAAG

ATACAGTTCACTCAACGATTGCCTTTGGGTTCCGAACTCCATATGGGGAAGGAACGTTGTTGTTTGCGGG

GTCTCGATCATTTACATGGACCCACTTTTCATTCCATTTGTGGGAATTTGATGATCTATAAACCGTCCTT

AACGAACGATCGGCTCATCTT------TGAGCATGATGAATCACTTCGTGCCGACCTGTTGTCAATCAAC

TTTTTGGCCTCATATGAGAATGGAAAACTGGAGCATTTTCTTCATCGGTGGATGAAGAATCGCGAACATA

AGCATTTTTTGTTAAGCATGTTCCCAGAAAAAAGATACTTTCGAGAAACAACGAGCACGACTGAAGTGGC

TATACATACAAATCCATTTACGGATCTATATGCTTCGATTGGAACTGGAAGTTCAAGAACAGGCGGCTGG

TATACTACCATAATGAAACTGCCTTTTATTTTTTTTATTCGGATAGGATTTCTGTTGGCTTCGTTGGGAG

GCTCGCGTAGTTTGTTACGTCAGCTCCAAAAGGAAAAATTGCGTTGGAATTGAGAAAGTTACATAA----

AGTTCATAATTGTATA--CAATTTTTGGGCCAATTCCCTCTTCGTACTACCAAAAAATGAGATTCTTGCC

GAATCCGAGTTTGCTGCTCCAACCATTACCAAACTAATACCTATTCTGTTTAGTACTTCAGGTGCTTCTG

TTGCGTATAATGTAAATCCCGTAGCGGATCAATTCC---------------AACGAGCCTTTCAAACTAG

TCCTTTTTGTAATCGACTCTATAGCTTCTTCAATAAACGCTGGTTCTTCGATCAAGTTTTGAATGACTTT

CTAGTCAGATCGTTCTTGCGTTTCGGATATGAAGTCTCATTCGAAGCTTTAGACAAAGGTGCTATTGAGA

TATTGGGCCCCTATGGTATCTCGTACACATTCCGACGATTGGCCGAGCGAATAAGTCAACTTCAAAGTGG

ATTTGTT-TGTTCCATGATCTATGGGTCTACTGGAGCTACCCATTTCGATCAATTAGCCAAGATTTTGAC

CGGATACGAAATCACTGGTGTTCGATCTAGTGGTATTTTTATGGGGATTCTTTTTATCGCTGTAGGATCC

CTATTCAAGATCACTGCAGTTCCTTTTC-GGTCTATGCACATCGCTTTCTCCAGGAGGTTGGCCGCCTAT

CCTAGATCTTCCCATTTCCAAGAGGATCCCGGGCTCAATCTGGTTTAGTATCAAGGTGATTCTCTTTCTC

TTTCTATATATATGGGTCCGTGCAGCATTTCCACGATATCGTTATGATCAATTAATGGGACTTGGCCGGA

AAGTGTTCTTGCCTCTATCATTAGCTCGGGTAGTCGCCGTTTCTGGTGTTTTAGTCACCTTTCAATGGCT

CCCTTAAATGCCTCAACTGGATAAATTTACTTATTTCACACAATTCTTCTGGTCATGCCTTTTCTTCTTT

ACTTTCTATATTCTAATATGCAATGATAAAGATGGAGTACTTGGGATCAGCAGAATTCTAAAACTACGAA

ACCAACTGCTTTCACACCGGGGGAACAAGATCCAAA------GCAAGGACCCCAACAGTTTGGAAGATAT

CTTGAGAAAAGGTTTTCACACAGGTGTATCCTATATGTACTCTAGTTTATTCGAAGTATCCCAATGGTGT

AAGTCCGTCGACTTATTGGGAAAAAGGAAGAAAATCACTTTGATCTCTTGTTTCGGAGAAATAAGTGGCT

CACGAGGAATGGAAAGAAACATATTCTATTTGATCTCGAAGTCCTCATATAGCACTTT------------

---TTCCAATCATGGATGGGGGATCACTTGTAAGAATGACATAATGCTAATCCATGTTCTACACGGCCAA

GGA--------------------

>Zygophyllum fabago MK431827.1

-TGATACTTTCTGTTTTGTCGAGCCCTGCTTTGGTCTCTGGTTTGATGGTTGCACGTGCTAAAAATCCGG

TACATTCCGTTTTGTTTCCCATCCTAGTCTTTCGCAACACTTCAGGTTTACTTCTTTTGTTAGGTCTCGA

CTTCTCCGCTATGATCTTCCCAGTCGTTCATATAGGAGCCATAGCCGTGTCATTCCTCTTCGTTGTTATG

ATGTTCCATATTCAAATAGCGGAGATTCACGAAGAAGTCTTGCGCTATTTACCAGTGAGTGGTCTTATTG

GACTGATCTTTTGGTGGGAAATGTTCTTCATTTTAGATAATGAAACCATTCCATTACTACCAACCCAAAG

AAAGACAACCTCTCTGAGATATACGGTTTATGCCGGAAAGGTACGAAGTTGGACTAATTTGGAAACATTG

GGCAATTTACTTTATACCTACTATTTCGTCTGGTTTTTGGTTCCTAGTCTGATTTTATTAGTAGCCATGA

TTGGGGCTATAGTACTGACTATGCATAGGACTACGAA------GGTGAAAAGACAGGATGTATTCCGACG

AAATGCTCTCGATTTTAGGAGGACTATCATGAGGAGGACGACAGACC-ATGTCAATATATGAATTTTTTC

ATTATTCGTTATTTCCGGGTCTTTTCGTTGCATTCACTTACAACAAGAAAAAACCACCAGCGTTTGGTGC

AGCACCTGCATTTTGGTGCATTCTTCTTTCTTTCCTTGGTCTTTCGTTCCGTCATCTTCCTAATAACTTA

TCCAATTACAACGTATTAACCGCTAATGCACCTTTCTTTTATAAAATCTCAGGGACATGGTCGAATCATG

AGGGTAGTATTTTATCATGGTGCCGGATCTCAAGTTTTTATGGATTCCTTCTTTGTTACCGGGGTCGACC

CCAAAGCCATAATGTCTCAAAACGAGGAGGCCATAGAGACACTCTTTTTGATTCCTTTGTCTCGAACTTC

GTGAAGAACTCCATTCTATCTCTCCCTCGTTACGAACAAAAAAGTGGTGTA------GATACCCAGTTGT

ACACCCCCTTCGTTCTACGAA---CCCTTGTTGATTCTTCACTTTGTTCGCGAAGGAACCGGACTTTTGA

CGGGCCAGCCC---------CGCCGCTTGACCCTGAAAGGAAAATGAGCTTTGCTCCTCTGGGCGCTAGG

------CGTGGTTCGCGAGAAGGAAAAAGGACTCATCCTTTGTTGCATCTGGCACGAGATGATCAAGAGA

GAGCTTCGTCTATCGATGAACAGCGGATTGACGGAGCTCTTGGCATTGCTTTGTTTTTCTCTCCTTTCCT

ATCAGCGAGTTCCGATCCTTTTGTTCGAAATTTATTCGTTCGTACCGAACCGCTTGCAGAATCAAATCCT

GTTCCACAAGATCCTATATCAGCTATACATCCTCCTTGCATTTATGCCGGAGACGTCGCCAGTGCTATGG

GCTTTGGCTTATGTAGATCAAAAATGATGAATGGGATTGTGGCACTCCACTCGCCGCCAATGCGGAAGGA

TGCCGCCGAAAAGAATGGAACGCTACTTCGCTCTGCTGGATGCGTCGGATCCCGTATAACAAGCGAGCTT

TTTACCCTCAAATTCAAACATGTGGGCGCAAAATGCTATCCTGCTCTATTTTTGTGT---------AGCC

TGCTC---ATGCTGCTTCGGCGGTGCTTTTTCGCCTTCTCTTCGCTCTGGACAGGAGCGCTAGTGGACAC

GGGGAGGGAGCA------GGCGAAGCGTGTCG------TTCGTAATGGAAAGAAAGATACCACTACTTCG

CCTCTTTGTTGGACCGCCGGCGCGAACACAGTGGTCTCTGACCAGGACCAGGAACAAATTCGAATTTGGA

TCTTGACATGTCGGTGGTTTTTAACCGTAGGCATCTTGCCAGGAAGTTGGTGGGCTCATCATGAATTAGG

TCGGGGTGGCTGGTGGTTTCGGGATCCCGTAGAAAATGCTTCTTTTATGCCTCGGGTATTAGCCACAGCT

CGTCTTCATTCAGTAATTCTACCCCTTCTTCATTCTTGGATCTCGCTTCTAAATATTGTGACTTTTCCAT

GCTGTGTCTCAGGAACCTTTTCAATACGGTCCGGATTGCTAGCTCCCGTTCATAGTTTTGCTACAGATGA

TACACGAGGAATCTTTTTATGGCGGTTCTTCCTTCTAATGACCGGCATATCTATGATTCTTTTCTCCCAG

ATTCAGCAGCAGGCATCGGTCCGTAGAACCTATAAAAAAGAGATGGTTGTGGCGCGAAGTACTCTTGTGC

ACCTACG--------------------GCTATTCATCCAAATTTCTCCTTGTGATGCAGCGGAACCATGG

CAATTAGGATCTCAAGACGCAGCAACACCTATGATGCAAGGAATCATGGACTTACATCACGATATCTTTT

TCTTCCTCATTCTTATTTTGGTTTTCGTATCACGGATCTTGGTTCGCGCTTTATGGCACTTCCACTATCA

AAAAAATCCAATCCCGCAAAGGATTGTTCATGGAACTACTATCGAGATTATTCGGACCATCTTTCCTAGT

ATCATCCCGATGTTCATTGCTATACCATCATTTGCTCTTTTATACTCAATGGACGAGGTAGTAGTAGATC

CAGCGATTACTATCAAAGCTATTGGACATCAATGGTATCGGACTTACGAGTATTCGGACTATAACAGTTC

TGATGAACAGTCACTAACTTTTGACAGTTATACGATTCCAGAAGAAGATCTAGAACTGGGTCAATCACGT

TTATTAGAAGTGGACAATAGAGTGGTTGTACCAGCAAAAACGCATCTACGTCTTCTTGTAACATCTGCTG

ATGTACCTCATAGTTGGGCTGTACCTTCTTCAGGTGTCAAATGTGATGCTGTACCTGGTCGTTTAAATCA

GATCTCTATTTCGGTACAACGAGAAGGAGTTTACTATGGTCAGTGCAGTGAGATTTGTGGAACTAATCAT

GCCTTTACGCCTATCGTCGTAGAAGCTGTTTCTAGGAAAGATTATGGTTCTCGGGTATCAAATCAATTAA

TCCCCCAAACCGGGGAAGCTTA-ATGAGACGACTCTTTCTTGAACTATATCATAAACAGATCTTTCCCTC

CACACCAATCACGAGTTTTTCTCCATTCCTCTCGTATATCGTCGTCACGCCCTTAATGCTAGGTTTTGAA

AAAGACTTTTCATGTCATTCCCATTTAGGTCCGATTCGGATCCCTCCGTTGTTTCCTTTTCCTCCCGCAC

CTTTTCCTCGAAATGAGAAAGAAGATGGTACACTCGAATTGTATTCTTTAAGTGCTTATTGCTTGCCAAA

GATCCTACTTCTACAATTGGTAGGTCACTGGGTTATTCAAATCAGTCGTGTTTTCTGTGGGTTTCCCATG

TTACAACTTCCGTACCAATTCGGTCGATCCGGAATGGATCGGTTAAACATTCCATTAGGGAGCCTGGTCT

TGACTCTTCTGTGTGGTATTCATTCTTGTTCGGCTCTTGGAATCACATCCAGCAGTGGTTGGAACAGCTC

GCAAAATCCAACCACTTCACCTACTTCATTGCCCCCAACCGTTTCTCGTACCTCTATTGAAACAGAATGG

TTTCATGTTCCTTCATCGATTGGTTATTCCTCTCCGTTTGTATCTCTTTTTCCAATTTCGGTCTCGATGA

GTTTACAAGATTG--TGTCCCTTTCGTTATTACAACCTTT-----TTTTTTGATGGCAAAGACCAGAAGC

TACGCGCAAATTCTCATTGGATCTCGGTTTTTCTTAACAGCGATGGCTATTCATTTAAGTCTTCGGGTAG

CACCACTAGATCTTCAACAAGGTGGAAATTCTCGTATTATCTATGTACATGTTCCTGCGGCTCGGATGAG

TATTCTTGTTTATATCGCCACGGCTATAAACACTTTCGTTTTCCTATTAACAAAACATCCCCTTTTTCTT

CGATCTTCCGGAACCGGTACAGAAATGGGTGCTTTTTTTACGTTTTTTACCTTAGTTACTGGGGGGTTTC

GGGGAAGACCTATGTGGGGCACCTTTTGGGTGTGGGATGCTCGTTTAACCTCTGTATTCATCTCGTTTCT

TATTTACCTGGGTGCACTGTGTTTTCAAAAGCTTCCTGTCGAACCGGCTTCTATTTCAATTTGTGCTGGA

CCGATCGATATACCAATAATAAAGTCTTCAGTCAACTGGTGGAATACATCGCATCAACCTGGGAGCATTA

GCCGATCTGGTACATCAATACATGTTCCTATGCCCATTCCAATCTTGTCTAACTTTGCTAACTCCCCCTT

CTCAACGGTTATCTTGTTTGTTTTGGAAACACGTCTTCCTATTCCATCTTTTCTCGAATCTCCTTTAACG

GAAGAAATAGAAGCTCGAGAAGGAA----TGCAGGCTAGAAAGATGCTATTTGCTGCTATCCCATCTATT

TGTGCATTAAGTTCGAAGAAAATCTCAATCTATAATGAAGAAATGATAGTAGCTCGTTGTTTTATAGGCT

TTATTATATTAAGTCGGAAGAGTTTCGGTCAGACTTTCAAAGTGACTCTCGACGGGAGAATCAAGGCTAT

TCAGGAAGAATCGCAGCAATTCCCCAATCCTAACGAAGTAGTTCCTCCGGAATCCAATGAACAACAAAAA

TTACTTAGGATCAGCTTGCGAATTTGTGGCGCCGTAGTAGAATCATTACCAATGGCACGCTGTGCGCCTA

AGTGCGAAAAGACAGTGCAAGCTTTGTTATGCCGAAACCTAAATGTTAAGTCAGCAACACTTCCAAATGC

CACTTCTTCCCGTCGCATCCGTCTTCAGGACGATCTAGTCAAAGGTTTTCACTTCTCAGTGAGTGAAAGA

TTTTTCCCCGGGTGTACTAAGAAAGCTTCTATAGTAGAACTCATTCGAGAGGGCTTGGTGGTCTTAAGAA

GGGTTCGGGTGGGGGGTTCTAT---TGAAAGAGGCGATCAGAATGGTACTCGAATCCATTTACGATCCCG

AGTTTCCAGACACATCGCACTTCCGCTCGGGTCGAGGCTGCCACTCGGCCCTCAGACGGATCAAAGAAGA

GTGGGGAACCTCTCGCTGGTTTTTGGAATTCGACATCAGGAAGTGTTTTCACACCATCGACCGACATCGA

CTCATCCCAATCTTGAAGGAAGAGATCGACGATCCCAAGTTCTTTTACTCCATTCAGAAAGTCTTTTCCG

CCGGACGACTCGTAGGAGGTGAGAAGAGCCCTGACTCCGTCCCACACAGTGTACTATTATCGGCCCTACC

AGGCAACATCTACCTACACAAGCTCGATCAGGAGATAGGGAGGATCCGACAGAAGTACGAAATTCCGATT

TTTCAGAGAATCAGATCGGTTCTATTAAGGACAGGTCGTCTTTATGACCAAGAACACTCTGGAGAAGAAG

CAATGGAATTCTCTACCAGAGCTGCGGAATTAACAACTCTATTAGAAAGTCGAATTACCAACTTTTACAC

GAATGTGAAAGTGGATGAGATCGGTCGAGTGATCTCAGTTGGAGATGGTATTGCGCGTGTTTATGGATTG

AAGGAGATTCAAGCTGGGGAAATGGTTGAATTTGCCAGCGGTGTGAAAGGAATAGCCTTCAATCTTGAGA

ATGAGAATGTAGGGATTGTTGTCTTTGGTAGTGATACCGCTATTAAAGAAGGAGATCTTGTCAAGCGCAC

TGGATCTATTGTTGATGTTCCCGCGGGAAAGGCTATGCTAGGGCGTGTGGTCGACGCGTTGGGAGTACCT

ATTGATGGAAGAGGGGCTCTAAGCGATCACGAGCGAAGACGTGTCGAAGTGAAAGCCCCTGGGATTATTG

AACGTCAATCTGTGCACGAACCTATGCAAACAGGGTTAAAAGCGGTAGATAGCCTGGTTCCTATAGGCCG

TGGTCAACGAGAACTTATAATCGGGGACCGACAAACTGGAAAAACAGCTATTGCTATCGATACCATATTA

AACCAAAAGCAACTGAACTCAAGGGCCACCTCTGAGAGTGAGAAATTGTATTGTGTTTATGTAGCGATTG

GACAGAAACGCTCAACTGTTGCACAATTAGTTCAAATTCTTTCAGAAGCTAATGCTTTGGAATATTCCAT

TCTTGTAGCAGCCACCGCTTCGGATCCTGCTCCTCTGCAATTTATGGCCCCATATTCTGGGTGTGCCATG

GGGGAATATTTCCGCGACAATGGAATGCACGCATTAATAATCTATGATGATCTTAGTAAACAGGCGGTGG

CATATCGACAAATGTCATTATTGTTACGCCGACCACCAGGCCGTGAGGCTTTCCCAGGGGATGTTTTCTA

TTTACATTCCCGTCTCTTAGAGAGAGCCGCTAAACGATCGGACCAGACAGGTGCAGGTAGCTTGACCGCC

TTACCCGTTATTGAAACACAAGCTGGAGACGTATCGGCCTATATTCCCACCAATGTGATCCCCATTACTG

ATGGACAAATCTGTTTGGAAACAGAGCTCTTTTATCGCGGAATTAGACCTGCTATTAACGTCGGCTTATC

TGTCAGTCGCGTCGGGTCTGCCGCTCAGTTGAAAGCTATGAAACAAGTCTGCGGTAGTTCAAAACTAGAA

TTGGCACAATATCGCGAAGTGGCCGCCCTTGCTCAATTTGGCTCAGACCTTGATGCTGCGACTCAGGCAT

TACTCAATAGAGGTGCAAGGCTGACAGAAGTACCGAAACAACCACAATATGCACCACTTCCAATTGAAAA

ACAAATTCTAGTCATTTATGCAGCTGTCAATGGATTCTGTGATCGAATGCCACTAGACAAAATTTCTCAA

TATGAGAGAGCCATCCCAAGTAGTATCAACCCAGAATTACTACAAGCACTTGTAGAGAAAGGTGGGTTAA

CTAAAGAAAGAAAGATGGAACCAGATGCATTATTAAACGAAAGCGCTTTGCCTTACCTAAGA--------

--------CATCCAACGCAAAGCGGCCTTTCATTCCCTTCTTTCGTCGTGGCACACCCCCCCCACAAGCA

CCCCCCGGCTCAGGGGGGACCAGAAAACGCCTGTCGTTTTCCCCCCTTCGTCGGCCCTTGCCGCCTTCCT

TAACAAGCCCTCGAGCCTCCTTTGCGCCGCCTTCCTCATAGAAGCCGCCGGATTGACCCCGAAGGCC---

------GGTAGAGAACGCTGTCATCATAATTGGGCCATGAGAGACCTTCTTCAGTCTTGCAAAAGAAAGG

GCCTGCTGATAGAGCTGGGCGGGGAGGCGATACTAGTGATCAGGTCAGAGAGACGCCTGGCCCGTCAGCT

GGCCCCCTTAAAAACCCATTACTTCATCAGGATTTGTTACACGCGATATGCCGACGACTTACTACTGGGA

ATCGTGGGTGCTGTAGAGCTTCTCATAGAAATACAAAAAGGTATCGCCCACTTCCTCAAATCCGGCCTTC

ACCTTTGGGTAGGATCCGCAGGATCAACAACAATAGCTGCACGGAGTACGGTAGAATTCCTCGGTACGGT

CATTCGGGAAGTCCCTCCGAGGACGACTCCCATCCAATTCTTGCGAGAGCTGGAGAAGCGTCTACGGGTA

AAGCACCGTATCCATATAACTGCTTGCCACCTACGCTCCGCCATCCATTCCAAGTGGAGCAACCTAGGTA

ATAGTATCCCGATCAAACAGCTGACGAAGGGGATGAGCGACAAAGGGAGTCTACTGGACGGGGTTAAACT

AGCGGAGACTCTTGGAACAGCTGGAGTCAGAATTCCCCAAGTGAGCGTCTTATGGGGGACCGTCAAGCAC

ATCCGGCAAGGATCCAGGGGGATCTCGTTGTTGCATAACTCAGGTCGGAGCAACGCACCATCGGACGTTC

AACAGGCAGTCTCACGATCGGGCATGAGTGTCCGGAAGTTGTC---ATTTGATACTCCCGCGGGTCGGAA

GGCGGCGGGGGAAGGA---GGACACTGGGCGGGATCTATCAGCAGCGAATTCTCCATACAAATAGAGGCG

CCTATCAAAAAGATAGTCACTCGGCTTCGGGATCGAGGTCTAATTAGCCGACGAAGACCCTGGCCAATCC

ACGTGGCCTGCTTGACGAACGTCAGCGACGTAGACATCGTCAATTGGTCCGCGGGCATCGCGATCAGTCC

TCTGTCCTACTACAGGTGCCGCGACAACCTTGACCAAGTCCGAACGATTGTCGACCACCAGATCCGCTGG

TCTGCAATATTCACCCTAGCCCACAAGCACAAATCCTCGGCGCGGAATATAATCCCAAAGTACTCCAAAG

ACTCACATATAGTCAATCAAGAAGGTGGTCAGACCCTTGCAGAGTTTCCCAACAGCATAGAACTTGGGAA

GCTCGGACCCGGTCAAGAT---AAAAAAAAAGGAGCACTC--------------------------TGGT

CCAACTACAGAACTTTGTCTTTTTAATTACTTCCATGGTCGTGCCTTGTGGCACGGCAGCACCCGTACTA

TTGAAATGGTTCGTCAGTAGAGATGTTCCCACAGGTGCCCCTTCTTCCAATGGTACTATAATTCCTATTC

CTATTCCTTCATTCCCTTTTTTAGTCTATCTACATTCCAGGAAATTCATACGCTCCATAGACAGAGCAAA

AAGTGGAGTCTTGGTCAGAGCAAGCCGCCCTCTTATAT------TACCAGACATAATTGGGAGAAGCTCA

TCCGAAACTAGAGCTAGAAACGCTTCATTTCGTTTCGTTCCCGTTCTTCATTTCCTTCTTCTCGAATCCA

A------GGGGGACTTCTCATATTTAGAATCTTTCTGCGGTGTGCTCCGTTTACTATTCTTTCGTACTCT

CTTCTCTTTACCACGCGATAGGTCAGCGAAGCGTGA------GCGGAGAAGGAAAGGCCAACAACTTCGG

CCTAAG------GAGAATGAGCAACGACGAAATGACAAGATGAGGTGCTCCGGGCACCCCCATT------

TAGAAAGAAGAAGGGTCGAAGGTTTTTGGCCTTTAGCTTTCCCCGTCCCCCCTTCGTCGGGTGGTGCTTG

TGTGGGGGGTGTGCCACCAGAAATCGGGCTTGAAGCTCTCGCCTTACCAACGAGCCAACAGCTGATAGCT

TTTGGTCACGACTACTACCAAAAAGCTCCAATGAAGATGAAGATTTCACATGGAGGAGTGTGCATCTGTA

TGTTGGGTGTTCTTCTGT-CGTACATAGCTGTTCCAGCTGAAATACTTGGAATAATTCTACCACTTCTCC

TAGGAGTAGCCTTTTTAGTGCTAGCTGAACGTAAAGTCATGGCTTTTGTGCAACGTCGAAAGGGTCCTGA

TGTAGTGGGATCGTTCGGATTGTTACAACCTCTAGCAGATGGGTCGAAATTGATTCTAAAAGAACCTATT

TCACCAAGTAGTGCTAATTTCTCCCTGTTTAGAATGGCTCCAGTGGTGACATTTATGTTAAGTCTGGTCG

CTCGGGCCGTTGTACCTTTTGATTATGGTATGGTATTGTCAGATCCGAACATAGGGCTACTTTATTTGTT

TGCCATATCTTCGCTAGGTGTTTATGGAATTATTATAGCAGGTCGGTCTAGTATTTATTATATACGCTTA

GCGAAAAGAATGTTTTTTGATACACCTAGGACATGGATTCTATATGAACCAATGGATCGTGACAAGTCGT

TACTACTAGCAATGACTTCCTCTTTCATTACTTCATCCTTTCCATATCCCTCTCCCTTGTTCTCAGTTAC

TCATCAAATGGCACTCAGTTCATATCTTTA--TGTCAGAATTTTCACCTATTTTGATCTATTTAGTGATC

AGTCCGCTAGTTTCTTTGATCCCACTCGGTGTTCCTTTTCCATTTTCTTCCAATAGTTCGACCTATCCAG

AAAAATTGTCGGCCTACGAATGTGGTTTCGATCCTTCCGGTGATGCCAGAAGTCGTTTTGATATACGATT

TTATCTTGTTTCAATTTTATTTATTATCCCTGATCCGGAAGTCACCTTTTTCTTTCCTTGGGCAGTACCT

CCCAACAAGATTGATCTGTTTGGATCTTGGTCCATGATGGCCTTTTTATTGATTTTAACAATTGGGTCTC

TCTATGAATGGAAAAGGGGAGCTCCAGATCGGGAGTAA--AAGTGTTTCTTACGATTACGCCCAACAGCC

CCCTTGAGCAATTTGCCATTATCCCATTGATTCCTATGAATATAGGAAACTTGTCTTTCTCATTCACAAA

TCCATCTTTGTTTATGCTGCTCACTCTCAGTTTTGTCCTACTTCTGGTTCATTTTGTGACTAAAAAGGGA

GGAGGAAACTTAGTACCAAATGCTTGGCAATCCTTGGTAGAGCTTATTCATGATTTCGTGCTGAACCCGG

TCAACGAACAAATAGGTGGTCTTTCCGGAAATGTGAAACAAAAGTTTTTCCCTCGCATCTCGGTCACTTT

TACTTTTTCGTTATTTCGTAATCCCCAGGGTATGATACCTTATAGCTTCACAGTGACAAGTCATTTTCTC

ATTACTTTGGGTCTCTCATTTTCTATTTTTATTGGCATTACTATAGTGGGATTTCAACAAAATGGGCTTC

ATTTTTTAAGCTTCTCATTACCCGCAGGAGTCCCACTGCCGTTAGCACCTTTTTTAGTACTCCTTGAGCT

AATCCCTCATTGTTTTCGCGCATTAAGCTCAGGAATACGTTTATTTGCTAATATGATGGCCGGTCATAGT

TCAGTCAAGATTTTAAGTGGGTCCGCTTGGACTATGCTATGTATGAATGATCTTTTCTATTTCATAGGAG

ATCTTGGCCCCTTATTTATAGTTCTTGCATTAACCGGTCCGGAATTAGGTGTAGCTATATCACAAGCTCA

TGTTTCTACGATCTCAATCTGTATTTACTTGAATGATGCTACAAATCTCCATCAAAG-------------

----------------ATATGTGGGCACCTGATATCTATGAGGGTTCACCCACCCCGGTGACAGCATTCC

TTTCGATTGCGCCTAAAATCTCTATTTCTGCTAATATTTCACGTGTTTCTATTTATGGTTCCTATGGAGC

TACATTGCAACAAATCTTCTTTTTCTGCAGCATTGCTTCTATGATCTTAGGAGCACTGGCCGCCATGGCC

CAAACGAAAGTCAAAAGACCTCTAGCTCATAGTTCAATTGGACATGTAGGTTATATTCGTACTGGTTTCT

CATGTGGAACCATAGAAGGAATTCAATCACTACTCATTGGTATCTTTATTTATGCATTAATGACGATAGA

TGCATTCGCCATAGTTTCAGCATTACGGCAAACCCGTGTCAAATATATAGCGGATTTGGGCGCTCTAGCC

AAAACGAATCCTATTTCGGCTATTACCTTCTCAATTACTATGTTCTCATACGCAGGAATACCCCCGTTAG

CCGGCTTTTGTAGCAAATTCTATTTGTTTTTCGCCGCTTTGGGTTGTGGGGCTTACTTCCTAGCCCCAGT

GGGAGTAGTGACTAGCGTTATAGGTCGT--GTTCGATAGCCCGACCGTAGTGATGTTAATTGTGGTTACA

TCCATAAGTAGCTTGGTCCATCTTTATTCAATTTCATATATGTCTGAGGATCCGCATAGCCCTCGATTTA

TGTGTTATTTATCCATTCTTACTTTTTTTATGCCAATGTTGGTGACTGGAGATAACTCTCTTCAATTATT

CCTGGGATGGGAGGGAGTAGGTCTTGCTTCATATTTGTTAATTCATTTCTGGTTTACACGACTTCAGGCA

GATAAAGCAGCTACAAAAGCTATGCCTGTCAATCGAGTAGGTGATTTTGGATTAGCTCCTGGGATTTCGG

GTCGTTTTACTCTCTTTCAAACAGTAGACTTTTCAACCATTTTTGCTCGTGCTAGTGCCCCCAGAAATTC

TTGGATTTCTTGCAATATGAGATTGAATGCCATCACTCTGATTTGTATTTTACTTCTTATTGGTGCTGTT

GGGAAATCTGCACAGATAGGATCGCATACTTGGTCACCCGATGCTATGGAGGGTCCCACTCCAGTATCCG

CTTCGATTCATGCAGCTACTATGGTCACAGCTGGCGTTTTCATGATAGCAAGGTGCTCCCCTTTATTTGA

ATACCCACCTACGGCTTTGATTGTGATTACTTCTGCAGGAGCTATGACGTCATTCCTTGCGGCAACCACT

GGAATCTTACAGAACGATCTCAAGAGGGTCATAGCTTATTCAACTTGCAGTCAATTAGGCTATATGATCT

TTGCTTGCGGCATCTCTAACTATTCGGTTAGCGTCTTTCACTTAATGAATCACGCGTTTTTCAAAGCATT

ACTCTTCCTGAGTGCGGGTTCGGTGATTCATGCCATGTCGGATGAGCAAGATATGCGGAAGATGGGGGGG

CTTGCCTCCTCGTTCCCTTTGACCTATGCCATGATGCTCATAGGCAGCTTATCTCTAATTGGATTTCCTT

TTCTAACTGGATTTTATTCCAAGGATGTGATCTTAGAGCTCGCTTACACTAAGTATACCATCAGTGGGAA

CTTTGCTTTCTGGTTGGGAAGTGTCTCTGTCCTTTTCACTTCTTTTTACTCTTTTCGTTCACTTTTTCTA

ACATTTATAGTACCAACTAATTCATTCGGGCGAGACACCTTACGATGTCATGATGCGCCCATTCCTATGG

CCATTCCTTCAATACTTCTGGCTCTCGGGAGTCTCTTTGTAGGATACTTGGCCAAA-TAACACAAAGAAG

ATACAGTTCACTCAACGATTGCCTTTGGGTTCCGAACTCCATATGGGGAAGGAGCGTTGTTGTTTGCGAG

GTCTCGATCATTTACATGGACCCACTTCTCATTCTATTTGTGGTAATTTGATTATCTATAAACCGTCCCT

CACGAACGATCGGCTCATGTT------TGAGCATGATGAATCACTTCGTGCCGACCTGTTGCCAATAAAC

TGTCCGGCCTCATATGAGAATGGAAAACTGGAGCATTTTCTGCATCGGTGGATGAAGAATCGCGAACATC

AGAATTTCTGGTTGACCATGTTCCCAGAAAAAAGATACTTTCGAGAAACGACAAGCACGACTGAAGTGGC

TATACATACAAATCTATTTACGGATCTATATGCTTCGATTGGAACTGGAAGTTCCAGAACAGGCGGCTGG

TATACCACCATAATGAAACTGCCTTTTATTTTTTTGATTCGGATAGGATTGATGTTGGCTTCGTCGGGAG

GCTCGCGTAGTTTGTTACGTCAGCTCCAAAAGGATAAGTTGCGTTGGAATTGAGAAAGTTCCGTGG----

AGTTCATAATTGCATAA-CAATTTTTGGGCTAATTCCCCATTCGTACTACCAAAAAATGAGATTCTTGCC

GAATCCGAGTTTGCTGCTCCAACCATTACCAAACTCATACCTATTCTTTTTAGTACTTCAGGTGCTTATG

TTGCGTATAATGTCAATCCCGTAGCGGATAAATTCC---------------AACGAGCCTTTCAAACTAG

TCTTTTTTGTAATCGACTCTATAGCTTCTTCAATAAACGCTGGTTCTTCGATCAAGTTTTGAATGACTTT

CTAGTCAGATCGTTCCTGCGTTTCGGATATGAAGTCTCATTCGAAGCTTTAGACAAAGGTGCTATTGAGA

TCTTGGGCCCTTATGGTATCTCGTACACATTCCGACGATTGGCCGAGCGAATCAGTCAACTTCAAAGTGG

ATTTGTTTTGTTCCATGATCTATGGGTCTACTGGAGCTACCCACTTCGATCAATTAGCCAAGATTTTGAC

CGGATACGAAATCACTGGTGCTCGATCTAGTGGTCTTTTTATGGGGATTCTCTTTATCGCTGTAGGATTC

CTATTCAAGATAACTGCAGTTCCTTTT--GGTCCATGCACATCGCTCTCTCCAGGAGGTTGGCCGCCTAT

CCTAGATCTTCCCATTTTAAAGAAGATCCCGGGCTCGATCTGGTTTAGTATCAAGGTGATTCTTTTTCTG

TTTCTATATATATGGGTCCGTGCAGCATTTCCACGATATCGTTATGATCAATTAATGGGACTTGGCCGGA

AAGTGTTCTTGCCTCTATCATTAGCTCGGGTAGTCCCCGTTTCTGGTGTTTCAGTCACCTTTCAATGGCT

CCCTTAAATGCCTCAACTGGATCAATTTACTTATTTCACACAATTCTTCTGGTCATGTCTTTTCCTCTTT

ACTTTCTATATTCCCATATGCAATAATGGAGATGGAGTCATTGGGATCAGCAGAATTCTGAAACTACGGA

ACCAACTGGTTTCACACCGGGGGAAAAACATCCGAA------GCAAGGACCCCAACAGTTTGGAAGATAT

CTTGAGAAAAGGTTTGAGCACCGGTGTATCCTATATGTACTCTAGTTTATTCGAAGTATCAAAATGGTGT

CACGCCGTCGACTTATTGGGAAAAAAGAAGACAATCACTTTGATCTCTTGTTTCGGAGAAATCAGTGGTT

CACGAGGAATGGAAAAAAACATCTTCTATTTGATCTCGAAGTCCTCATATAGCACTTA------------

---TTCCAATCCTGGATGGGGGATCACTTGTAGGAATGACATCATGCTCATCCATGTTCCACACGGCCAA

GGAAGCATCGTTT----------

>Agrostemma githago NC057604.1

ATGATACTTTCTGTTTTGTCGAGCCCAGCTTTGGTCTCTGGTTTGATGGTTGTACGTGCTAAAAATCCGG

TACATTCCGTTTTGTTTCCCATCCCAGTCTTTCGCAACACTTCAGGTTTACTTCTTTTGTTAGGTCTCGA

CTTTTCCGCTATGATCTTCTTAGTAGTTTATATAGGAGCTATAGCCGTTTCATTCCTATTCGTTGTTATG

ATGTTCCATATTCAAATAGCGGAGATTCACGAAGAAGTCTTGCGCTATTTACCAGTGAGTGGTATTATTG

GACTGATCTTTTGGTGGGAAATGTTCTTCATTTTAGATAATGAAACCATTCCATTACTACCAACCCAAAG

AAATACGACCTCTCTGAGATATACGGTTTATGCCGGAAAGGTACGAAGTTGGACTAATTTGGAAACATTG

GGCAATTTACTTTATACTTACTATTTTGTCTGGTTTTTGGTTTCTAGTCTTATTTTATTAGTAGCCATGA

TTGGGGCTATAGTACTGACTATGCATAGGACTACTAA------GGTGAAAAGACAGGATGTATTCCGACG

AAATGCTATTGATTCTAGAAGGACTATAATGAGGAGGACGACAGAC---TGTCAATATATGAATTGTTTC

ATTATTCGTTATTTCCGGGTCTTTTCATTGCATTCACTTACAACAAGAAACAACCACCAGCGTTTGGTGC

AGCACCCGCATTTTGGTGTATTCTTTTTTCTTTCTTTGGTCTTTTGTTCTGTCATATTTCTAATAACTTA

TCCAATTACAACGTATTAACCGCTAATGCACCTTTCTTTTATCAAATCTCAGGGACATGGTCTAATCATG

AAGGTAGTATTTTATTATGGTGTCGGATCCCAAGTTTTTATGGATTCCTTCTTTGTTACCGGGGTCGATC

CCAAAGCCATAATGTCTCAAAACGAGGAGGCCATAGAGAAAGTCTTCTTTTTTCCTTTGTCTTAAACTTC

GTGAAGAACTCCATTCTATCTCTTCCTCGTTACGAACAAAAAAGTAGAGTTCTTCACGAACCCCAGTTGT

ACACTCTCTTCGTTCTACGAA---CTCTTGTTGATTCTGAACTTTGTTCGCGAAGGAAGCGGACTTTTGA

CGGGCCAGCTCTTTTTTACGCGCCGCTTTACCCTGAAAGGAAAATGAGCTTTGCTCTTCTGGCCGCGAGG

CGCTCTCGTGGTTCGCGAGAAGGAAAAAGGACTCATCCTTTGTTGCATCTGGCACGAGATGATAAAGAGA

GAGCTTCGTCTATCGATGAACAGCGGATTGACGGAGCTCTTGGCATTGCTTTGTTTTTCTTTCCTTTCTT

ATCAGCGAGTTCCGATCCTTTTGTTCGAAATTTCTTTGTTCGTACCGAACCGCTTGCAGAATCAAATCCT

GTTCCACAAGATCCTATATCAGCAATACACCCCCCTTGTATTTATGCCGGAGACGTCGCCAGTGCTATGG

GCTTTGGCTTATGTAGATCAAAAATGATGAATGGGATTGTGGCACTCTACTCGCCGCCAATGCGGAAGGA

TGCCGCCGAAAAGAATGGAACGCTACTTTGCTCTGCTGGATGCGTCGGATCCCGTATAACAAGCGAGCTC

TTTACCCTCAAATTCAAACATGTGGGCACCAAATGCTATCCTGCTTTATTGTTGCGTAGCAAAAGAAGCC

TGCTC---ATGCTGCTTCGGCGGCGCTTTTTCGCCTTCTCTTGGCTCTGGACAAGAGCGCTAGTGGACAC

GGGGGGGGGGCG------GGCGAAGCCTTTCT------TTCGTAATGGAAAGAAAGAGACCACTACTTTG

CCTCTTTGTTGGACCGCCGGCGCGAACACAGTGGTCTCTGACCAGGACCAGGAAGCTATTCGAATTTGGA

TCTTGACATGTCGGTGCTTTTTAACCGTAGGCATCTTGCCAGGAAGTTGGTGGGCTCATCATGAATTAGG

TCGGGGTGGCTGGTGGTTTCGGGATCCCGTAGAAAATGCTTCTTTTATGCCTCGGGTATTAGCCACAGCT

CGTATTCATTCAGTCATTTTACCCCTTCTTCATTCTTGGACTTTGCTTCTTAATATTGTGACTTTTCTAT

GCTGTGTCTTAGGAACCTTTTCAATACGGTCCGGATTGCTAGCTCCCGTTCATAGTTTTGCTACAGATGA

TACACGAGGAATCTTTTTATGGCGGTTTTTCCTTCTAATGACCGGCATATCTATGATTATTTTCTCTCAG

ATGAAGCAGCAGGCATCGGTCCGTAGAACCTATAAAAAAGAGATGGTTGTAGCGCGAAGTACTCTTGTGC

ACTTACGT---TGATTGTTCGAGAATGGCTA---TTCACAATTGCTCCTTGTGATGCAGCGGAACCGTGG

CAATTAGGATTTCAAGACGCAGCAACACCTATGATGCAAGGAATAATAGACTTACATCATGATATCTTTT

TCTTCCTTATTCTTATTTTGGTTTTCGTATCATGGATCTTGGTTCGCGCTTTATGGCATTTCCACTATAA

AAAAAATCCAATCCCGCAAAGGATTGTTCATGGAACTACTATCGAGATTATTCGGACCATCTTTCCTAGT

ATCATCCTGATGTTCATTGCTATACCATCATTTGCTCTCTTATACTCAATGGACGAGGTAGTAGTAGATC

CAGCCATTACTATCAAAGCTATTGGACATCAATGGTATTGGACTTATGAGTATTCGGACTATAACAGTTC

CGATGAACAGTCACTAACTTTTGACAGTTATATGATTCCAGAAGATGATTTAGAATTGGGTCAATTACGT

TTATTAGAAGTGGACAATAGAGTGGTTGTACCAGCCAAAACTCATATACGTATTATTGTAACATCTGCTG

ATGTACTTCATAGTTGGGCTGTACCTTCCTTAGGTGTAAAATGTGATGCTGTACCTGGTCGTTTAAATCA

GACCTCTATTTTGGTACAACGAGAAGGAGTTTACTATGGTCAGTGCAGTGAAATTTGTGGAACTAATCAT

GCTTTTATGCCTATCGTCGTAGAAGCTGTTTCTAGGAAAGATTATGGTTCTCGGGTATCTACTCAATTAA

TCCCCCAAACCGGAGAAGCTTAAATGAGACGACTCTTTTTTGAACTATATCATAAACAGATCTTCTTCTC

CACACCAATCACGACTTTTTCTCCATTCCTCTCGTATATTGTCGTAACGCCCTTAATGCTAGGTTTTGAA

AAAGACTTTTCATGTCATTTCCATTTAGGTCCGATTCGGATCCCTCCGTTGTTTCCTTTTCCTCCCGCAC

CTTTTCTTCGAAATGATAAAGAAGATGGTACACTCGAATTGTATTATTTAAGTGCTTATTGCTTGCCAAA

GATCCTACTTCTACAATTGGTAGGTCACCGGGTTATTCAAATAAGTCGTGTTTTCTGTAGTTTTCCCATG

TTAAAACTTTTGTACCAATTCGGCCAATCCGGAATGGATCGGTTAAACATTCTATTAGGGAGCCTGGTCT

TGACTCTTCTGTGTGGTATTCATTCTTGTTTGGCTCTTGGAATCACATCCAGCAGTGGTTGGAACAGCTC

GAAAAATTTAACCACTTCACCTACTTCATTGCCCTCAACCGTTTCTCGTACCTCTATTGAAACAGAATGG

TTTCATGTTCTTTCATCGATTGGTTATTTTTCTTCGTTCGTATCTCTTTTTCCAATTTCGGTCTCGATTA

GTTCACAAGATTG---------TTCTTTATTACAACCTTC-----TTTTTTGATGTCAAAGACCAAAAGC

TACGCGCAAATTCTCATTGGATCTTGGTTGTTCTTAACAGCGATGGCTATTCATTTAAGTCTTTGGGTAG

CACCACTAGATCTTCAACAAGGTGGAAATTCTCGTATTCTCTATGTACATGTTCCTGTGGCTCGGATGAG

TATTATTCTTTATATCGTTACGGCTATAAACACTTTCTTGTTCCTATTAACAAAACATCCTCTTTTTCTT

CGCTCTTCCGGAACCGGTACAGAAATGGGTGCTTTTTCTACGCTGTTAACTTTAGTAACTGGGGGGTTTC

GGGGAAGACCCATGTGGGGCACCTTTTGGGTGTGGGACGCTCGTTTAACTTCTGTATTCATCTCATTCCT

TATTTACTTGGGCGCACTGTGTTTTCAAAAGCTTTCTGTCGAACCGGCTCCTATTTCAATCCGTGCTGGA

CCGATCGATATACCAATAATAAAGTTTCCAGTCAACTGGTGGAATACATCGCATCAACCCGGGAGCATTA

GCCGATCTGGTACATCAATACATGTTCCTATGCCCATTCCAATCTTGTCTAACTTTGCTAACTTCCTCTT

CTCAACCCGTATCTTCTTTGTTCTGGAAACACGTCTTCCTATTCCATCTTTTCTCGAATCTCCTTTAACG

GAAGAAATAGAAGTTCGAGAAGGAA-----GCAGGCTAGAAAGATGCTATTTGCTGCTATTCTATCTATT

TGTGCATCAAGTTCGAAGAAGATCTCAATCTATAATGAAGAAATGATAGTAGCTCGTTGTTTTATAGGCT

TTATCATATTCAGTCGGAAGAGTTTAGGTAAGACTTTCAAAGTGACTCTCGACGAGAGAATCCAGGCTAT

TCAGGAAGAATCGCAGCAATTCCCCAATCCTAACGAAGTAGTTCCTCCGGAATCCAATGAACAACAACGA

TTACTTAGGATCAGCTTGCGAATTTGTGGCACCGTAGTAGAATCATTACCAATGGCACGCTGTGCGCCTA

AGTGCGAAAAGACAGTGCAAGCTTTGTTATGTCGAAACCTAAATGTTAAGTCAGCAACACTTCCAAATGC

CACTTCTTCCCGTCGCACCCGTCTTCAGGACGATCTAGTCACAGGGTTTCACTTCTCAGTGAGTGAAAGA

TTTGTCCCCGGGTCTACGTTGAAAGCTTCTATAGTTGAACTCATTCGAGAAGGCTTGGCGGTCTTAAGAA

TGGTTCGGGTAGGAGGTTCTCTT--TGAAAGAGGCGATCAGAATGGTACCCGAATCCATTTACGATCCCG

AGTTTCCAGACACATCGCACTTCCGCTCGGGTCGAGGCTGCCACTCGGCCCTAAGACGGATCAAAGAAGA

GTGGGGAACCTCTCGCTGGTTTTTGGAATTCGACATCAGGAAGTGTTTTCACACCATCGACCGACATCGA

CTCATACCAATCTTTAAGGAAGAGATCGACGATCCCAAGTTCTTTTACTCCATTCAGAAAGTCTTTTCCG

CCGGACGACTCGTAGGAGGTGAGAAGGGCCCTTACTCTGTCCCACACAGTGTACTACTATCGGCCCTACC

AGGCAACATCTACTTACACAAGCTCGATCAGGAGATAGGGAGGATCCGACAGAAGTACGAAATTCCGATT

GTTCAGAGAATCAGATCGGTTCTATTAAAGACAAGTCGTATTGATGACCAAGAAAACTCTGGAGAAGAAG

---TGGAATTCTCTCCCAGAGCTGCGGAACTAACAACTCTATTAGAAAGTAGAATTAGCAACTTTTATAC

GAATTTTCAAGTGGATGAGATCGGTCGAGTGGTCTCAGTTGGAGATGGGATTGCACGTGTTTATGGATTG

AACGAGATTCAAGCTGGAGAAATGGTGGAATTTGCCAGCGGTGTGAAAGGAATAGCCTTAAATCTTGAGA

ATGAAAATGTAGGGATTGTTGTCTTTGGTAGTGATACCGCTATTAAAGAGGGCGATCTTGTCAAGCGCAC

TGGATCTATTGTGGATGTTCCTGCGGGAAAAGCTATGCTAGGGCGTGTGGTCGACGCGTTGGGAGTACCT

ATTGATGGAAGAGGGGCTCTAAGCGATCACGAGCGTCGACGTGTCGAAGTGAAAGCCCCTGGGATTATTG

AACGTAAATCTGTGCACGAGCCTATGCAAACCGGCTTAAAGGCGGTAGATAGCCTGGTTCCTATAGGCCG

TGGTCAACGAGAACTTATAATCGGGGACCGACAAACGGGAAAAACAGCTATTGCTATCGATACCATATTA

AACCAAAAGCAACTGAACTCAAAGGCCACTTCTGAGAGTGAGACATTGTATTGTGTTTATGTAGCGATTG

GACAGAAACGTTCAACTGTGGCACAATTAGTTCAAATTCTTTCAGAAGCGAATGCTTTGGAATATTCCAT

TCTTGTAGCAGCCACTGCTTCGGATCCTGCTCCTCTTCAATTTCTGGCCCCATATTCTGGATGTGCTATG

GGAGAATATTTCCGCGATAATGGAATGCACGCATTAATAATCTATGATGATCTTAGTAAACAGGCGGTGG

CATATCGACAAATGTCATTATTGTTACGCCGACCACCAGGCCGTGAGGCTTTCCCAGGCGACGTTTTCTA

TTTACATTCTCGTCTCTTAGAAAGAGCCGCTAAACGATCGGACCAGACAGGTGCAGGTAGCTTGACCGCC

TTACCCGTCATTGAAACACAAGCTGGAGACGTATCAGCCTATATTCCCACCAATGTGATCTCCATTACTG

ATGGACAAATCTGTTTGGAAACAGAGCTCTTTTATCGCGGAATTAGACCTGCTATTAACGTCGGCTTATC

TGTCAGTCGCGTCGGGTCTGCCGCTCAGTTGAAAGCTATGAAACAAGTCTGCGGGAGTCCAAAACTGGAA

TTGGCACAATATCGCGAAGTGGCCGCCTTTGCTCAATTTGGCTCAGACCTTGATGCTGCGACTCAGGCAT

TACTCAATAGAGGTGCAAGGCTTACAGAAGTACCGAAACAACAACAATATGCACCACTTCCAATTGAAAA

ACAAATTATAGTCATTTACGCAGCTGTCAATGGATTCTGTGATCGAATGCCACTAGATAAAATTTCTCAA

TATGAGAGAACCATTCCAAATAGTGTAAACCCAGAATTCTTACAATCCCT------AAAGGGCGGCTTAA

CTAACGAAAAAAAGATGGAACTAGATTCATTCTTAAAAGAATGCGCTTTGAATTACC-------------

--------CATCCAACGCAAAGCGGCCTTTCATTCCCTTGTTTCGTCGTGGCACAACCCCCCCGCAAGCA

CCCCCCGGCTCAGGGGGGACCAGAAAAGGCCTTTCGTTTTGCCCCCTTCGTCGGCCCTTGCCGCCTTCCT

TAACAAGCCCTCGAGCCTCCTTTTCGCTGCCTTACTCATAGAAGCCGCCGGGTTGACCCCGAAGGCCGAA

TTCTATGGTAGAGAATGCTTTCATAATAATTGGGCCATGAGAGACCTTTTTAAGTATTGCAAAAGAAAGG

GCCTGCTGATAGAGCTGGGCGAGGCAGGGATACTCGTTCTCAGGTCAGATAAAGGCCTGGCCCGTAAGCT

GGCCCCCTTAAAAAGCCATTACTTAATAAGGATTTGTTACGCGCGATATGCCGACGACTTACTATTTGGA

ATCGTGGGTGCCGTAGAGCTTCTCATAGAAATACAAAAACGTATCGCCCACTTCCTACAATCCGGCCTGA

ACCTTTGGGTAGGCTCTGCGGGATCAACAACAATAGCTGCACGGAGTACGGTAGAATTCCTCGGTACGGT

CATTCGGGAAGTCCCTCCGAAGACGACTCCCATACAATTCTTGCGAGAGCTGGAGAAGCGTCTACGGGTA

AAGTACCGTATCCATATAACTGCTTGCCACTTACGCTCTGCCATTCATTCCAAGTTTAGGAACCTAGGTA

AGAGTATACCGATCAAACAGCTGACGAAGGGGATGAGCAAAACAAGGAGTCTACTGGACGCGGTTCAACT

AGCGGAAACTCTTGGAACAGCTAGAGTAAGAAGCCCCCAAGTGAGCTTATTATGGGAGACCGTCAAGCAC

ATCCGGCAAGGATCAAGGGAGATCTCGTTGTTGCATAGCTCAGGTCAGAGCAAGGTGCCACCGGACGTTC

AACAGGTAGTCTCGCGATCGGGCATGAGTGCCCGGAAGTTGTC---ATTGTATACTCTCGCGGGTCGGAA

GGCGGCGGGGGAAGGGGGGGGACACTGGGCGAGATCTATCAGCAGCGAATTCCCCATACAGATAGAAGCG

CCTATCAAAAAGATACTCCGAAGGCTTCGAGATCGAGGTCTCATTAGCCGAAGAAGACCCTGGCCAATCC

ACGTGGCCTGCTTGACGAACGTCAGCGACGGAGACATCGTAAATTGGTTCGCGGGCATCGCGATAAGTCC

TCTGTCCTACTACAGGTGCCGCGACAACCTTTACCAAGTCCGAACGATTGTCGACTACCAGATCCGCTGG

TCTGCAATCTTCACCCTAGCCCACAAGCACAAATCCTCAGCGCGGAATATAATCCCAAAGTACTCCAAAG

ACTTAAATATAGTAAATAAAGAAGGTGGCAAGACCCTTGCAGAGTTCCCCAACAGCAGAGAGCTTGGAAA

GCTCGGACCCGGGCAAGATCC-GAACAACAAAGAGCACTCAACTACTA------------------TGGT

CCAACTACATAACTTTTTCTTTTTCATTACTTCCATGGTCGTGCCTTGTGGCACGGCAGCACCCGTACTA

TTGAAATGGTTCGTCAGTAGAGATGTTCCCACAGGTGCCCCTTTTTCCAATGGTACTTTAATTCCTATTC

TTATCCCTTCATTCCTTCTTTTGGTTTATCTACATTCCAGGAAATTCATACGCTCCATGGACAGAGTCAA

AAGTGGAGTCTTGGTCAGAGCAAGCTGCCCTATTTTAT------TACCAGACATAATTGGGAGAAGCTCA

TCCGAAACTAGAGCTAGAAACGCCTTCTTTAGTTTCGTTCCCATTCTTCATTTTCTTCTTCTCGAATCCA

A------GGGGGACTTACCCTATTTAGAATCTTTTTGCGGTGTGCTCCGTTTACTATTCTTTCGTACTTT

CTTCTCTTTATCACGCGATAGGTCAGCGAAGCGTGAGCGGGCGCGGAGAAGAAAACGCCAAACACTTCGG

ACTAAC------GGGAATGAGCAACGACGAAATGACAAGATAAAGTGCCCCGGGCGCCCCCATT------

TAGA---AAGAAGGGTCGAAGGGTTTGGACCTGTAGCTTTCCCCGTGCCCCCTTCGTCGGGTGGTGCTTG

CATGGGGGGTGTGCTACCTGAAATCGGGCTTGAAGCTCCCGCCTTACCAACGAGCCGACAGCTGATGGCT

GTTGGTCACGACTACTACCAAAAAGTGAACATGAGGATGACTATTTCACATGGGGGAGTGTGCATCTTTA

TCTTGGGTGTTCTTCTGT-CGTACATAGCTGTTCCAGCTGAAATACTTGGAATAATTCTACCACTTCTAC

TAGGAGTAGCCTTTTTAGTGCTAGCTGAACGTAAAGTAATGGCCTTTGTGCAACGTCGAAAGGGTCCTGA

TGTAGTGGGATCGTTCGGATTGTTACAACCTCTAGCAGATGGTTCGAAATTGATTCTAAAAGAACCTATT

TCACCAAGTAGTGCTAATTTCTCCCTTTTTAGAATGGCTCCAGTCACTACATTTATGCTAAGTCTGGTTG

CTCGGGCCGTTGTACCTTTTGATTATGGTATGGTATTGTCAGATCCGAACATAGGGCTACTTTATTTGTT

TGCCATATCTTCGCTAGGTGTTTATGGAATTATTATAGCAGGTTGGTCTAGTATTTATTATATACGTTTA

GTGAAAAGAATGTTTTTTGATACACCTAGGACATGGATTCTATATGAACCAATGGATCGTAACAAGTCGT

TACTACTAGCAATGACTTCCTCTTTCATTACTTCATCCTTTCCATATCCTTCTCCCTTGTTCTCAGTTAC

TCATCAAATGGCACTCAGTTTATATCTTTA--TGTTAGAATTTGCGCCTATTTGTATCTATTTAGTGATC

AGTCTGCTAGTTTCTTTGATCCCACTCGGTGTTCCTTTTCCATTTTCTTCTAATACTTCGACTTATCCAG

AAAAATTGTCGGCCTACGAATGTGGTTTCGATCCTTTCGGTGATGCCAGAAGTCGTTTCGATATACGATT

TTATCTTGTTTCAATTTTATTTATTATCCTTGATCCGGAAGTAACCTTTTTCTTTCCTTGGGCAGTACCT

CTCAACAAGATTGATCCGTTTGGATTTTGGTCCATGATGGCCTTTTTATTGATTTTAACGATAGGATTTC

TCTATGAATGGAAAAGGGGTGCTTTGGATCGGGAGTAA---------------------GCCCAACAGCC

CACTTGACCAATTTGCCATTCTCCCATTGATTCCTATGAAACTAGGAAACTTGTATTTCTCATTCACAAA

TCCATCTTTGTTTATGCTGCTAACTCTCAGTTTGGTCCTACTTCTGCTTCATTTTGTTACTAAAAAGGGA

GGAGGAAACTCAGTACCAAATGTTTGGCAATCCTTGGTAGAGCTTATTTATGATTTCGTGCTGAACCTGG

TAAACGAACAAATAGGGGGTCTTTCCGGAAATGTTAAACAAAAGTTTTTCCCTTGCATCTTGGTCACTTT

TACTTTTTTGTTATTTCGTAATCTCCAAGGTATGATACCTTATAGCTTTACAGTTACAAGTCATTTTCTC

ATTACTTTGGGTCTTTCATTTTCCATTTTTATTGGCATTACTATAGTGGCATTTCAAAGAAATGGGCTTC

ATTTTTTAAGCTTCTCCTTACCTGCAGGAGTCCCACTGCCGTTAGCACCTTTTTTAGTACTCCTTGAGCT

AATCCCTCATTGTTTTCGCGCATTAAGCTTAGGAATACGGTTATTTGCTAATATGATGGCCGGTCATAGT

TCAGTAAAGATTTTAAGTGGGTTCGCTTGGACTATGCTATGTATGAATGATCTTTTATATTTCATAGGAG

ATCTTGGTCCTTTATTTATAGTTCTTGCATTAACCGGTCTTGAATTAGGTGTTGCTATATTACAAGCTCA

TGTTTTTACGATCTTAATCTGTATTTACTTGAATGATGCTACAAATCTCCATCAAA--------------

----------------ATATGTGGGCACCTGATATCTATGAGGGTTCACCCACCCCGGTTACAGCATTCT

TTTCTATTGCGCCTAAAATCTCTATTTCTGCTAATATTTTACGTGTTTTTATTTATGGTTCCTATGGAGC

TACATTGCAACAAATTTTCTTTTTCTGCAGCATTGCTTCTATGATCTTAGGAGCACTGGCTGCCATGGCC

CAAACAAAAGTCAAAAGACTTCTAGCTCATAGTTCAATTGGACATGTAGGTTATATTCGTACTGGTTTCT

CATGTGGAACCATAGAAGGAATTCAATCACTACTAATTGGTCTCTTTATTTATGCATCAATGACGATAGA

TGCATTCGCTATAGTTTCAGCATTACGGCAAACCCGTGTAAAATATATAGCGGATTTGGGCGCTCTAGCC

AAAACGAATCCTATTTCGGCTATTACCTTCTCTATTACTATGTTCTCATACGCAGGAATACCCCCGTTAG

CCGGCTTTTGTAGTAAATTCTATTTGTTCTTCGCCGCTTTGGGTTGTGGGGCTTACTTCCTAGCCCCAGT

GGGAGTAGTGACTAGCGTTATAGGTTGT--GTTCGATAGCCCGACCGTAGTGATGTTAATTGTGGTTACA

TTCATAAGTAGCTTGGTCCATCTTTATTCTATTTCATATATGTCTGAGGATCCGCATAGCCCTCGATTTA

TGTGTTATTTATCCATTCTTACTTTTTTTATGCCAATGTTGGTGACTGGAGATAACTCTCTTCAATTATT

CTTGGGATGGGAGGGAGTAGGTCTTGCTTCATATTTGTTAATTCATTTTTGGTTTACACGACTTCAGGCA

GATAAAGCAGCTATAAAAGCTATGCTTGTCAATCGAGTAGGTGATTTTGGATTAGCTCTTGGGATTTCGG

GTCGTTTTACTCTCTTTCAAACAGTAGACTTTTCTACCATTTTTGCTTGTGCTAGTGCCCCTAGAAATTC

TTGGATTTCTTGCAATATGAGATTGAATGCCATAACTCTTATTTGTATTTTACTTTTTATTGGTGCTGTT

GGAAAATCTGCACAGATAGGATCGCATACTTGGTCACCCGATGCTATGGAGGGTCCCACTCCAGTATCCG

CTTTGATTCATGCAGCTACTATGGTAACAGCTGGCGTTTTCATGATAGCAAGGTGTTCCCCTTTATTTGA

ATACCCACCTACGGCTTTAATTGTTATTACTTTTGCAGGAGCTATGACGTCATTCCTTGCGGCAACCACT

GGAATATTACAGAACGATCTAAAGAGGGTCATAGCTTATTCAACTTGCAGTCAATTAGGCTATATGATCT

TTGCTTGCGGCATTTCTAACTATTCGGTTAGCGTCTTTCATTTAATGAATCACGCCTTTTTCAAAGCATT

ACTATTCCTGAGTGCGGGTTCGGTGATTCATGCCATGTCGGATGAGCAAGATATGCGGAAGATGGGGGGG

CTCGCCTCCTCGTTCCCTTTTACCTATGCCATGATGCTCATGGGCAGCTTATCTCTAATTGGATTTCCGT

TTCTAACTGGATTTTATTCCAAAGATGTGATCTTAGAGCTCGCTTACACTAAGTATACCATCAGTGGGAA

CTTTGCTTTCTGGTTGGGAAGTGTCTCTGTCCTTTTCACTTCTTATTACTCTTTTCGTTCACTTTTTCTA

ACATTTCTAGTACCAACTAATTCATTCGGGCGAGACATCTTACGATGTCATGATGCGCCCATTCCTATGG

CCATTCCTTTAATACTTCTGGCTTTCGGGAGTCTCTTTGTAGGATACTTGGCCAAA-TAACACAAAGAAG

ATACAGTTCACTCAACGATTGCCTTTGGGTTCCGAACTCCATATGGGGAAGGAACGTTGTTGTTTGCGGG

GTCTCGATCATTTACATGGACCCACTTTTCATTCCATTTGTGGGAATTTGATGATCTATAAACCGTCCGT

AACGAACGATCGGCTCATCTT------TGAGCATGATGAATCACTTCGTGCCGACCTGTTGTCAATAAAC

TTTTTGGCCTCATATGAGAATGGAAAACTGGAGCATTTTCTGCATCGGTGGATGAAGAATCGCGAACAAA

AAAATGTCTGGTTAAGCATGTTCCCAGAAAAAAGATACTTTCGAGAAACAACGAGCACGACTGAAGTGGC

TATCCATACAAATCCATTTACGGATCTATATGCTTCGATTGGAACTGGAAGTTCAAGAACAGGCGGCTGG

TATACTACTATAATGAAACTGCCTTTTATTTTTTTTATTCGGATAGGATTTCTGTTGGCTTCGTTGGGGG

GCTCGCGTAGTTTGTTACGTCAACTCCAAAAGGAGAAATTGCGTTGGAATTGAGAAAGTTACGTAA----

AGTTCATAATTGTATAACCAATTTTTGGGCCAATTCCCTCTTCGTACTACCAAAAAATGAGATTCTTGCC

GAATCCGAGTTTGCTGCTCCAACCATTACCAAACTAATACCTATTCTGTTTAGTACTTCAGGTGCTTCTG

TTGCGTATAATGTAAATCCCGTAGCGGATCAATTCC---------------AACGAGCCTTTCAAACTAG

TACTTTTTGTAATCGACTCTATAGCTTCTTCAATAAACGCTGGTTCTTCGATCAAGTTTTTAATGACTTT

CTAGTCAGATCGTTCTTGCGTTTTGGATATGAAGTCTCATTCGAAGCTTTAGACAAAGGTGCTATTGAGA

TATTGGGCCCCTATGGTATCTCGTACACATTCCGACGATTGGCCGAGCGAATAAGTCAACTTCAAAGTGG

ATTTGT-TTGTTCCATGATCTATGGGTCTACTGGAGCTACCCATTTCGATCAATTAGCCAAGATTTTGAC

CGGATACGAAATCACTGGTGTTCAATCTAGTGGTATTTTTATGGGGATTCTTTTTATCGCTGTAGGATCC

CTATTCAAGATCACTGCAGTTCCTTTT--GGTCTATGCACATTGCTTTCTCCAGGAGGTTGGCCGCCTAT

CCTAGATCTTCCCATTTCCAAGAAGATCCCGGGCTCGATCTGGTTTAGTATCAAGGTGATTCTCTTTCTC

TTTCTATATATATGGGTCCGCGCAGCATTTCCACGATATCGTTATGATCAATTAATGGGACTTGGCCGGA

AAGTGTTCTTGCCTCTATCATTAGCTCGGGTAGTCTTAGTTTCTGGTGTTTTAGTCACCTTTCAATGGCT

CCCTTAAATGCCTCAACTGGATAAATTGACTTATTTCACACAATTCTTCTGGTCATGCCTTTTTTTCTTT

ACTTTCTATATTCTAATATGCAATGATAGAGATGGAGTACTTGGGATCAGCAGAATTCTAAAACTACGAA

ATCAACTGCTTTCACACCGGGGGAACGACATCCAAA------GCAAGGACCCCAACAGTTTGGAAGATAT

CTTGAGAAAGGGTTTTAACACAGGTGTATCCTATATGTACTCTAGTTTATTCGAAGTATCCCAATGGTGT

AAGGCCGTCGACTTATTTGGAAAAAGGAAGAAAATCACTTTGATCTCTTGTTTCGGAGAAATAATTGGCT

CAGGAGGAATAGAAGGAAACATATTCTATTTTATCTTGGAGTCTTCATATAGCACTTCGTTCTGGGAATT

CCTTTCAAGGACGGGATGGGGGATCACTGGTAAGAATGACATAACGCTAATCCATGCTCTAAACGGCCAA

GAA--------------------

>Nepenthes ventricosa NC039531.1

ATGATACTTTCAGTTTTGTCGAGCCCTGCTTTGGTCTCTGGTTTGATGGTTGTACGTGCTAAAAATCCGG

TACATTCCGTTTTGTTTCCCATCCCAGTCTTTCGCGACACTTCAGGTTTACTTCTTTTGTTAGGTCTCGA

CTTTTCCGCTATGATCTTCCCAGTAGTTCATATAGGAGCTATAGCCGTTTCATTCCTATTCGTTGTTATG

ATGTTCCATATTCAAATAGCGGAGATTCACGAAGAAGTATTGCGCTATTTACCAGTGAGTGGTATTATTG

GACTGATCTTTTGGTGGGAAATGTTCTTCATTTTAGATAATGAAACAATTCCATTACTACCAACCCAAAG

AAATACGACCTCTCTGAGATATACGGTTTATGCCGGAAAGGTACGAAGTTGGACTAATTTGGAAACATTG

GGCAATTTACTTTATACTTACTATTTTGTCTGGTTTTTCGTTTCTAGTCTTATTTTATTAGTAGCCATGA

TTGGGGCTATAGTACTTACTATGCATAGGACTACTAA------GGTGAAAAGACAGGATGTATTCCGACG

AAATGCTATTGATTTTAGGAGGACTATAATGAGGAGGACGACAGAC--ATGTCAATATATGAATTGTTTC

ATTATTCGTTATTTCCGGGTCTTTTCATTGCATTCACTTACAACAAGAAACAACCACCAGCGTTTGGTGC

AGCACCTTCATTTTTTTGCATTCTTCTTTCTTTCCTTGGTCTTTTGTTCTGTCATATTCCTAATAACTTA

TCCAATTACAACGTATTAACCGCTAATGCACCTTTCTTTTATCAAATCTCAGGGACATGGTCTAATCATG

AGGGTAGTATTTTATTATGGTGTCGGATCCCAAGTTTTTATGGATTCCTTCTTTGTTACCGGGGTCGATC

CCAAAGCCATAATGTCTCAAAACGAGGAGGCCATAGAGAAACTCTTTTTTATTCCTTTGTCTTGAACTTC

GTGAAGAACTCCATTCTATCTCTTCCTCGTTACGAAAAAAAAACTGGAGTTCTTCACGAACCCCAGTTGT

ACACTCTCTTCGTTCTACAAA---CTCTTGTTGATTCTGAACTTTGTTCGCGAAGGAACCGGACGTTTGA

CGGGCCAGCTCTTTTTTACGCGTCGCTTTACCCTGAAAGGAAAATTCGCTTTGCTCTTCTGGGCGCTAGG

CGCTGTCGTGGTTCGCGAGAAGGAAAAAGGACTCATCCTTTGTTGCATCTGGCACGAGATGATAAAGAGA

GAGCTTCGTCTATCGATGAACAGCGGATTGACGGAGCTCTTGGCATTGCTTTGTTTTTCTTTCCTTTCCT

ATCAGCGAGTTCCGATCCTTTTGTTCGAAATTTATTCGTTCGTACCGAACCGCTTGCAGAATCAAATCCT

GTTCCACAAGATCCTATATCAGCTATACATCCTCCTTGCATTTATGCCGGAGACGTCGCCAGTGCTATGG

GCTTTGGCTTATGTAGATCAATAATGATGAATGGGATTGTGGCACTCCACTCGCCGCCAATGCGGAAGGA

TGCCTCCGAAAAGAATGGAACGCTGCTTTGCTCTGCAGGATGCGTCGGATCCCGTATAACAAGCGAGCTC

TTTACCCTAAAATTCAAACATGTGGGCGCAAAATGCTATCCTGCTCTATTGTTGCGTAGCAATAGAAGCC

TGCTC---ATGCTGCTTCGGCGGCGCTTTTTCGCCTTCTCTTCGCTCTGGACAAGAGCGCTAGTGGACAC

GGGGAGGGAGCA------GGCGAAGCGTGTCG------TTCGTAATGGAAAGAAAGATAGCACTACTTCG

CCTCTTTGTTGGACCGCCGGCGCGAACACAGTGGTCTCTGACCAGGACCAGGAACCAATTCGAATTTGGA

TCTTGACATGTCGGTGGTTTTTAACCGTAGGCATCTTGCCAGGAAGTTGGTGGGCTCATCATGAATTAGG

TCGGGGTGGCTGGTGGTTTCGGGATCCCGTAGAAAATGCTTCTTTTATGCCTCGGGTATTAGCCACAGCT

CGTATTCATTCAGTAATTTTACCCCTTCTTAATTCTTGGACTTTCTTTCTTAATATTGTGACTTTTCTAT

GCTGTGTCTTAGGAACCTCTTTAATACGGTCCGGATTGCTAGCTCCCGTTCATAGTTTTGCTACAGATGA

TACACGAGGAATCTTTTTATGGCGGTTCTTCCTTCTAATGACCGGCATATCTATGATTCTTTTCTCTCAG

ATGAAGCAGCAGGCATCGGTCCGTAGAACCTATAAAAAAGAGATGGTTGTAGCGCGAAGTACTCTTGTGC

ACTTACG----TGATTGTTCTAGAATGGCTATTCTTCACAATTGCTCCTTGTGATGCAGCGGAACCATGG

CAATTAGGATCTCAAGACGCAGCAACACCTATGATGCAAGGAATAATAGACTTACATCATGATATCTTTT

TCTTCCTCATTCTGATTTTGGTTTTCGTATCATGGATCTTGGTTCGCGCTTTATGGCATTTCCACTATAA

AAAAAATCCAATCCCGCAAAGGATTGTTCATGGAACTACTCTCGAGATTCTTTGGACCATATTTCCTAGT

ATCATCCTGATGTTCATTGCTATACCATCATTTGCTCTGTTATACTCAATGGACGAGGTAGTAGTAGATC

CAGCCATTACTATCAAAGCTATTGGACATCAATGGTATCGGAGT--------------------------

----------------------------------------------------------------------

----------------------------------------------------------------------

----------------------------------------------------------------------

----------------------------------------------------------------------

----------------------------------------------------------------------

------------------------TGAGACGACTCTTTTTTGAACTATATCATAAACAGATCTTCTTCTC

CACACCAATCACGAGTTTTTCTCCATTCCTCTCGTATATTGTCGTAACGCCCTTAATGCTAGGTTTTGAA

AAACACTTTTCATGTCATTTCCATTTAGGTCCGATTCGGATCCCTCCGTTGTTTCCTTTTCCTCCCGCAC

CTTTTCCTCGAAATGAGAAAGAAGATGGTACACTCGAATTGTATTATTTAAGTGCTTATTGCTTGCCAAA

GATCCTACTTCTACAATTGGTAGGTCACCGGGTTATTCAAATAAGTCGTGTTTTCTGTAGTTTTCCCATG

TTACAACTTCTGTACCAATTCGGTCGATCCGGAATGGATCGGTTAAACATTCTATTAGGGAGCCTGGTCT

TGACTCTTCTGTGTGGTATTCATTCTCGTTCGGCTCTTGGAATCACATCCAGCAGTGGTTGGAACAGCTC

GCAAAATTCAACCACTTCACCTACTTCATTGCCCTCAACCGTTTCTCGTACCTCTATTGAAACAGAATGG

TTTCATGTTCTTTCATCGATTGGTTATTCTTCTCCGTTCGTATCTCTTTCTCCAATTTCGGTCTCGATTA

GTTCACAAGATTGA-TGTCCGTTTTGTTATTACAACCTTC-----TTTTTTGATGTCAAAGACCAGAAGC

TACGCGCAAATTCTCATTGGATCTTGGTTGTTCTTAACAGCGATGGCTATTCATTTAAGTCTTTGGGTAG

CACCACTAGATCTTCAACAAGGTGGAAATTCTCGTATTCTCTATGTACATGTTCCTGCGGCTCGGATGAG

TATTCTTGTTTATATCGTTACGGCTATAAACACTTTCTTGTTCCTATTAACAAAACATCCTCTTTTTCTT

CGCTCTTCCGGAACCGGTACAGAAATGGGTGCTTTTTCTACGTTGTTTACTTTAGTTACTGGGGGGTTTC

GGGGAAGACCTATGTGGGGCACCTTTTGGGTGTGGGATGCTCGTTTAACTTCTGTATTCATCTCGTTCCT

TATTTACCTGGGTGCACTGTGTTTTCAAAAGCTTCCTGTCGAACCGGCTCCTATTTCAATCCGTGCTGGA

CCGATCGATATACCAATAATCAAGTCTCCAGTCAACTGGTGGAATACATCGCATCAACCTGGGAGCATTA

GCCGATCTGGTACATCAATACATGTTCCTATGCCCATTCCAATCTTGTCCAACTTTGCTAACTCCCTCTT

CTCAACCCGTATCTTGTTCGTTCTGGAAACACGTCTTCCTATTCCATCTTTTCCCGAATCTCCTTTAACG

GAAGAAATAGAAGCTCGAGAAGGAATACC-GCAGGCTAGAAAGATGCTATTTGCTGCTATTCTATCTATT

TGTGCATCAAGTTCGAAGAAGATCTCAATCTATAATGAAGAAATGATAGTAGCTCGTTGTTTTATAGGCT

TTATCATATTCAGTCGGAAGAGTTTAGGTAAGACTTTCAAAGTGACTCTCGACGAGAGAATCCAGGCTAT

TCAGGAAGAATCGCAGCAATTCCCCAATCCTAACGAAGTAGTTCCTCCGGAATCCAATGAACAACAACGA

TTACTTAGGATCAGCTTGCGAATTTGTGGCACCGTAGTAGAATCATTACCAATGGCACGCTGTGCGCCTA

AGTGCGAAAAGACAGTGCAAGCTTTGTTATGTCGAAACCTAAATGTTAAGTCAGCAACACTTCCAAATGC

CACTTCTTCCCGTCGCATCCGTCTTCAGGACGATCTAGCCACCGGTTTTCACTTCTCAGTGAGTGAAAGA

TTTATCCCCGGGTGTACGTTAAAGGCTTCTATAGTAGAACTCATTCGAGAAGGCTTGGTGGTCTTAAGAA

TGGTTTGGGTAGGAGGTTCTCTT-ATGAAAGAGGCGATCAGAATGGTACCCGAATCCATTTATGATCCCG

AGTTTCCAGACACATCGCACTTCCGCTCGGGTCGAGGCTGCCACTCGGCCCTAAGACGGATCAAAGAAGA

GTGGGGAACCTCTCGCTGGTTTTTGGAATTCGACATCAGGAAGTGTTTTCACACCATCGACCGACATCGA

CTCATCCCAATCTTTAAGGAAGAGATCGACGATCCCAAGTTCTTTTACTCCATTCATAAAGTGTTTTCTG

CCGGACGACTCGTAGGAGGTGAGAAGGGCCCTTACTCCGTCCCACACAGTGTACTACTATCGGCCCTACC

AGGCAACATCTACCTACACAAGCTCGATCAGGAGATAGGGAGGATCCGACAGAAGTACGAAATTTCGATT

GTTCAGAGAATCAGATCGGTTCTATTAAAGACAAGTCGTATTGATGACCAAGAAAACTCTGGAGAAGAA-

--ATGGAATTTTCTCCCAGAGCTGCGGAACTAACGACTCTATTAGAAAGTCGAATTACCAACTTTTACAC

AAATTTTCAAGTGGATGAGATCGGTCGAGTGGTCTCAGTTGGAGATGGGATTGCACGTGTTTATGGATTG

AACGAGATTCAAGCTGGGGAAATGGTGGAATTTGCCAGCGGTGTGAAAGGAATAGCCTTCAATCTTGAGA

ATGAGAATGTAGGGATTGTTGTCTTTGGTAGTGATACCGCTATTAAAGAAGGAGATCTTGTCAAGCGCAC

TGGATCTATTGTGGATGTTCCTGCGGGAAAGGCTATGCTAGGGCGTGTGGTCGACGCGTTGGGAGTACCT

ATTGATGGAAGAGGGGCTCTAAGCGATCACGAGCGAAGACGTGTCGAAGTGAAAGCCCCGGGAATTATAG

AACGTAAATCTGTGCACGAGCCTATGCAAACTGGGTTAAAAGCGGTAGATAGCTTGGTTCCTATAGGCCG

TGGTCAACGAGAACTTATAATCGGGGACCGACAAACTGGAAAAACAGCTATAGCTATCGATACCATATTA

AACCAAAAGCAAATGAACTCAAGGGCCACCTCTGAGAGTGAGACATTGTATTGTGTCTATGTAGCGATTG

GACAGAAACGCTCAACTGTGGCACAATTAGTTCAAATTCTATCAGAAGCGAATGCTTTGGAATATTCCAT

TCTTGTAGCAGCCACCGCTTCGGATCCTGCTCCTCTGCAATTTCTGGCCCCATATTCTGGCTGTGCTATG

GGAGAATATTTCCGCGATAATGGAATGCACGCATTAATAATCTATGATGATCTTAGTAAACAGGCCGTGG

CATATCGACAAATGTCATTATTGTTACGCCGACCACCAGGCCGTGAGGCTTTCCCAGGCGATGTTTTCTA

TTTACATTCTCGTCTCTTAGAAAGAGCCGCTAAACGATCGGACCAGACAGGTGCAGGTAGCTTGACCGCC

TTACCCGTCATTGAAACACAAGCTGGAGATGTTTCGGCCTATATTCCCACCAATGTGATCTCCATTACGG

ATGGACAAATCTGTTCGGAAACAGAGCTCTTTTATCGCGGAATTAGACCTGCTATTAACGTCGGCTTATC

TGTCAGTCGCGTCGGGTCTGCCGCTCAGTTGAAAGCTATGAAACAAGTCCGCGGTAGTTCAAAACTGGAA

TTGGCCCAATATCGCGAAGTGGCCGCCCTTGCTCAATTTGGCTCAGACCTTGATGCTGCGACTCAGGCAT

TACTCAATAGAGGTGCAAGGCTTACAGAAGTACCCAAACAACCACAATATGCACCACTTCCAATTGAAAA

ACAAATTCTAGTCATTTACGCAGCTGTCAATGGATTCTGTGATCGAATGCCACTAGACAAAATTTCTCAA

TATGAGAGAACCATTCCAAATAGTGTAAAACCAGAATTATTACAATCCCTTTTAGAAAAGGGTGGCTTAA

CTAACGAAAGAAAGATGGAACTAGATGCATTCTTAAAAGATAGCGCCTTGCCT-----------------

-------GCATCCAACGCAAAGCGGCCTTTCATTCCCTTGTTTCGTCGTGGCACACCCTCCCCACAAGCA

CCCCCCGGCTCAGGGGGGACCAGAAAAGGCCTTTCGTTTTCCCCCCTTCGTCGGCCCTTGTCGCCTTCCT

TAACAAGCCCTCGAGCCTCCTTTGCGCTGCCTTCCTCATAGAAGCCGCCGGGTTGACCCCGAAGGCCGAA

TTCTATGGTAGAGAACGCTGTAATAATAATTGGGCCATGAGAGACCTTATTAAGTATTGCAAAAGAAAGG

GCCTGCTGATAGAGCTGGGCGGGGAAGCGATACTAGTTATCAGGTCAGAGAAAGGCCTGGCCCGTAAGCT

GGCCCCCTTAAAAAGCCATTACTTAATAAGGATTTGTTACGCGCGATATGCCGACGACTTACTACTGGGA

ATCGTGGGTGCCGTAGAGCTTCTCATAGAAATACAAAAACGTATCGCCCACTTCCTACAATCCGGCCTGA

ACCTTTGGGTAGGCTCTGCAGGATCAACAACAATAGCTGCACGGAGTACGGTAGAATTCCTCGGTACGGT

CATTCGGGAAGTCCCTCCGAGGACGAGTCCCATACAATTCTTGCGAGAGCTGGAGAAGCGTCTACGGGTA

AAGCACCGTATCCATATAACTGCTTGCCACTTACGCTCTGCCATCCATTCCAAGTTTAGGAACCTAGGTA

ATAGTATCCCGATCAAACAGCTGACGAAGGGGATGAGCAAAACTGGGAGTCTACTGGACGCGGTTCAACT

AGCGGAGACTCTTGGAACAGCTAGAGTAAGAAGTCCCCAAGTGAGCGTATTATGGGAGACCGTCAAGCAC

ATCCGGCAAGGATCAAGGGAGATCTCGTTGTTGCATAGCTCAGGTCAGAGCAACGTGCCATCGGACGTTC

AACAGGCAGTCTCACGATCGGGCATGAGTGTCCGGAAGTTGTC---ATTGTATACTCTCGCGGGTCGAAA

GGCGGCGGGGGAAGGAGGGGGACACTGGGCGAGATCTATCAGCAGCGAATTCCCCATACAGATAGAAGCG

CCTATTAAAAAGATACTCCGAAGGCTTCGAGATCGAGGTCTCATTAGCCGAAGAAGACCCTGGCCAATCC

ACGTGGCCTGCTTGACGAACGTCAGCGACGGAGACATCGTAAATTGGTCCGCGGGCATCGCGATAAGTCC

TCTGTCCTACTACAGGTGCCGCGACAACCTTTACCAAGTCCGAACGATTGTCGACCACCAGATCCGCTGG

TCTGCAATATTCACCCCAGCCCACAAGCACAAATCCTCAGCGCGGAATATAATCCCAAAGTACTCCAAAG

ACTCAAATATAGTAAATAAAGAAGGTGGTAAGACCAAAGCAGAGTTCCCCAACAGCATAGAGCTTGGGAA

GCTCGGACCCGGTCAAGATCC-GAAAAACAAAGAGCACTCAACTACT------------------ATGGT

CCAACTACATAACTTTTTCTTTTTCATTACTTCCATGGTCGTGCCTTGTGGCACGGCAGCACCCGTACTA

TTGAAATGGTTCGTCAGTAGAGATGTTCCCACAGGTGCCCCTTTTTCCAATGGTACTATAATTCCTATTC

CTATCCCTTCATTCCTTCTTTTGGTCTATCTACATTCCAGGAAATTCATACGCTCCATGGACGGAGCAAA

AACAGGAGTCTTGGTCAGAGCAAGCTGCCCTATTCTAT------TACCAGACATAATTGGGAGAAGCTCA

TCCGAAACTAGAGCGAGAAACGCCTTATTTCGTTTCGTTCCCGTTCTTCATTTTCTTCTTCTCGAATCCA

A------GGGGGACTTCTCATATTTAGAATCTTTTTGCGGTGTGCTCCGTTTACTATTCTTTCGTACTTT

CTTCTCTTTACCACGCGATAGGTCAGCGAAGCGTGAGCGGGCGCGGAGAAGAAAACGCCAAACACTTCGG

CCTAAC------GGGAATGAGCAACGACGAAATGACAAGATAAAGTGCCCCGGGCACCCCCATT------

TAGA---AAGAAGGGTCGAAGGTTTTGGGCCTGTAGCTTTCCCCGTCCCCCCTTCGTCGGGTGGTGCTTG

TGTGGGGGGTGTGCCACCAGAAATCGGGCTTGAATCTCTCGCCTTACCAACGAGCCGACAGCTGATGGCT

GTTGGTCACGACTACTACCAAAAAGTGAACATGAAGATGAATATTTCACATGGAGGAGTGTGCATCTGTA

TGTTGGGTGTTCTTCTG-ACGTACATAGCTGTTCCAGCGGAAATACTTGGAATAATTCTACCACTTCTAC

TAGGAGTAGCCTTTTTAGTGCTAGCTGAACGTAAAGTAATGGCTTTTGTGCAACGTCGAAAGGGTCCTGA

TGTAGTGGGATCGTTCGGATTGTTACAACCTCTAGCAGATGGTTTGAAATTGATTCTAAAAGAACCTATT

TCACCAAGTAGTGCTAATTTCTCCCTTTTTAGAATGGCTCCAGTGGCTACATTTATGCTAAGTCTGGTTG

CTCGGGCCGTTGTACCTTTTGATTATGGTATGGTATTGTCAGATCCGAACATAGGGCTACTTTATTTGTT

TGCCATATCTTCGCTAGGTGTTTATGGAATTATTATAGCTGGTCGGTCTAGT-TTTATTATATACGTTTA

GCGAAAAGAATGTTTTTTGATACACCTAGGACATGGATTCTATATGAACCCATGGATCGTGACAAGTCGT

TACTACTAGCAATGACTTCCTCTTTCATTACTTCATCCTTTCCATATCCTTCTCCCTTGTTCTCAGTTAC

TCATCAAATGGCACTCAGTTTATATCTTTA--TGTCAGAATTTTCGCCTATTTTTCTCTATTTAGTGATC

AGTCCGCTAGTTTCTTTGATCCCACTCGGTGTTCCTTTTCCATTTGCTTCTAATAGTTCGACTTATCCAG

AAAAATTGTCGGCCTACGAATGTGGTTTCGATCCTTCCGGTGATGCCAGAAGTCGTTTCGATATACGATT

TTATCTTGTTTCCATTTTATTTATTATCCTTGATCCGGAAGTCACCTTTTTCTTTCCTTGGGCAGTACCT

CCCAACAAGATTGATCCGTTTGGATCTTGGTCCATGATGGCCTTTTTATTGATTTTAACGATTGGATTTT

TCTATGAATGGAAAAGGGGTGCTTCGGATCGGGAGTAA-AAAGTGTTTCTTACGATTACGCCCAACAGCC

CACTTGAGCAATTTGCCATTCTCCCATTGATTCCTATTCATATAGGAAACTTGTATTTCTCATTCACAAA

TCCATCTTTGTTTATGCTGCTAACTCTAAGTTTGGTCCTACTTCTGGTGCATTTTGTTACTAAAAACGGA

GGAGGAAACTCAGTACCAAATGCTTGGCAATCCTTGGTAGAGCTTATTCATGATTTCGTGCTGAACCCGG

TAAACGAACAAATAGGTGGTCTTTCCGGAAATGTGAAACAAAAGTTTTCCCCTCGCATCTTGGTCACTTT

TACTTTTTTGTTATTTCGTAATCTCCAGGGTATGATACCTTATAGCTTTACAGTTACAAGTCATTTTCTC

ATTACTTTTGGTCTTTCATTTTCTATTTTTATTGGCATTACTATAGTGGGATTTCAAAGAAATGGGCTTC

ATTTTTTAAGCTTCTCATTACCAGCAGGAGTCCCACTGCCGTTAGCACCTTTTTTAGTACTCCTGGAGCT

AATCCCTCATTGTTTTCGCGCATTAAGCTCAGGAATACGTTTATTTGCTAATATGATGGCCGGTCATAGT

TCAGTAAAGATTTTAAGTGGGTCCGCTTGGACTATGCTATGTATGAATGATCTTTTATATTTCATAGGAG

ATCCTGGTCCTTTATTTATAGTTCTTGCATTAACCGGTCTGGAATTAGGTGTAGCTATATCACAAGCTCA

TGTTTTTACGATCTCAATCTGTATTTACTTGAATGATGCTACAAATCTCCATCAAA--------------

----------------ATATGTGGGCACCTGATATCTATGAGGGTTCACCCACCCCGGTTACAGCATTCT

TTTCTATTGCGCCTAAAATCTCGATTTCTGCTAATATTTTACGTGTTTTTATTTATGGTTCCTATGGAGC

TACATTGCAACAAATCTTCTTTTTCTGCAGCATTGCTTCTATGATCTTAGGAGCACTGGCCGCCATGGCC

CAAACGAAAGTCAAAAGACTTCTAGCTCATAGTTCAATTGGACATGTAGGTTATATTCGTACTGGTTTCT

CATGTGGAACCATAGAAGGAATTCAATCACTACTAATTGGTCTCTTTATTTATGCATCAATGACGATAGA

TGCATTCGCTATAGTTTCAGCATTACGGCAAACCCGTGTAAAATATATAGCGGATTTGGGCGCTCTAGCC

AAAACGAATCCTATTTCGGCTATTACCTTCTCTATTACTATGTTCTCATACGCAGGAATACCCCCGTTAG

CCGGCTTTTGTAGTAAATTCTATTTGTTCTTCGCCGCTTTGGGTTGTGGGGCTTACTTCCTAGCCCCAGT

GGGAGTAGTGACTAGCGTTATAGGTCGT-TGTTCGATAGCCCGACCGTAGTGATGTTAATTGTGGTTACA

TCCATAAGTAGCTTGGTCCATCTTTATTCTATTTCATATATGTCTGAGGATCCGCATAGCCCTCGATTTT

TGTGTTATTTATCCATTCTTACTTTTTTTATGCCAATGTTGGTGACTGGAGATAACTCTCTTCAATTATT

CTTGGGATGGGAGGGAGTAGGTCTTGCTTCATATTTGTTAATTCATTTTTGGTTTACACGACTTCAGGCA

GATAAAGCAGCTATCAAAGCTATGCTTGTCAATCGAGTAGGTGATTTTGGATTAGCTCCTGGGATTTCGG

GTCGTTTTACTCTCTTTCAAACAGTAGACTTTTCTACCATTTTTGCTCGTGCGAGTGCCCCTAGAAATTC

TTGGATTTCTCGCAATATGAGATTGAATGCCATAACTCTTATTTGTATTTTACTTTTTATTGGTGCTGTT

GGAAAATCTGCACAGATAGGATCGCATACTTGGTTACCCGATGCTATGGAGGGTCCCACTCCAGTATCCG

CTTTGATTCATGCAGCTACTATGGTTACAGCTGGCGTTTTCATGATAGCAAGGTGTTCTCCTTTATTTGA

ATACCCACCTACGGCTTTAATTGTTATTACTTTTGCAGGAGCTACGACGTCATTCCTTGCGGCAACCACT

GGAATATTACAGAACGATCTAAAGAGGGTCATAGCTTATTCAACTTGCAGTCAATTAGGCTATATGATCT

TTGCTTGCGGCATTTCTAACTATTCGGTTAGCGTCTTTCATTTAATGAATCACGCGTTTTTCAAAGCATT

ACTATTCCTGAGTGCAGGTTCGGTGATTCATGCCATGTCGGATGAGCAAGATATGCGGAAGATGGGGGGG

CTTGCCTCCTCGTTCCCTTTTACCTATGCCATGATGCTCATGGGCAGCTTATCTCTAATTGGATTTCCTT

TTCTAACTGGATTTTATTCCAAAGATGTGATCTTAGAGCTCGCTTACACTAAGTATACCATCAGTGGGAA

CTTTGCTTTCTGGTTGGGAAGTGTCTCTGTCCTTTTCACTTCTTATTACTCTTTTCGTTCACTTTTTCTA

ACATTTCTAGTACCAACTAATTCATTCGGGCGAGACATCTTACGATGTCATGATGCGCCCATTCCTATGG

CCATTCCTTTAATACTTCTGGCTTTCGGGAGTCTCTTTGTAGGATACTTGGCCAA-CTAACACAAAGAAG

ATACAGTTCACTCAACGATTGCCTTTGGGTTCCGAACTCCATATGGGGAAGGAACGTTGTTGTTTGCGAG

GTCTCGATCATTTACATGGACCCACTTCTCATTCAATTTGTGGGAATTTTATGATCTATAAACCGTCCTT

AACGAACGATCGGCTCATGTT------TGAGCATGATGAATCACTTCGTGCCGACCTGTTGTCAATAAAC

TTTTCGGCCTCATATGAGAATGGAAAACTGGAGCATTTTCTGCATCGGTGGATGAAGAATCGCGAACATC

AAAATTTCTGGTTGACCATGTTCCCAGAAAAAAGATACTTTCGAGAAACAACGAGCACGACTGAAGTGGC

TATACATACAAATCCATTTACGGATCTATATGCTTCGATTGGAACTGGAAGTTCCAGAACAGGCGGCTGG

TATACTACCATAATGAAACTGCCTTTTCTTTTTTTTATTCGGATAGGATTTATGTTGGCTTCGTTGGGAG

GCTCGCGTAGTTTGTTACGTCAGCTCCAAAAGGAGAAATTGCGTTGGAATCGAGAAAGTTCCGTAA----

AGTTCATAATTGTATA--CAATTTTTGGGCCAATTCCCTCTTCGTACTACCAAAAAATGAGATTCTTGCC

GAATCCGAGTTTGCTGCTCCAACCATTACCAAACTAATACCTATTCTGTTTAGTACTTCAGGTGCTTCTG

TTGCGTATAATGTAAATCCCGTAGCGGATCAATTCC---------------AACGAGCCTTTCAAACTAG

TACTTTTTGTAATCGACTCTATAGCTTCTTCAATAAACGCTGGTTCTTCGATCAAGTTTTGAATGACTTT

CTAGTCAGATCGTTCTTGCGTTTCGGATATGAAGTCTCATTCGAAGCTTTAGACAAAGGTGCTATTGAGA

TATTGGGCCCTTATGGTATCTCGTACACATTCCGACGATTGGCCGAGCGAATAAGTCAACTTCAAAGTGG

ATTTGTTTTGTTCCATGATCTATGGGTCTACTGGAGCTACCCACTTCGATCAATTAGCCAAGATTTGGAC

CGGATACGAAATCACTGGTGTTCGATCTAGTGGTATTTTTATGGGGATTCTATTTATCGCTGTAGGATCC

CTATTCAAGATCACTGCAGTTCCTTTT-TGGTCTATGCACATCGCTTTCTCCAGGAGGTTGGCCGCCTAT

CCTAGATCTTCCCATTTCCAAGAGGATCCCGGGCTCGATCTGGTTTAGTATCAAGGTGATTCTGTTTCCG

TTCCTATATATATGGGTCCGTGCAGCATTTCCACGATATCGTTATGATCAATTAATGGGACTTGGCCGGA

AAGTGTTCTTGCCTCTATCATTAGCTCGGGTAGTCCCCGTTTCTGGTGTTTTAGTCACCTTTCAATGGCT

CCCTTA-ATGCCTCAACTGGATAAATTTACTTATTTCACACAATTCTTCTGGTCATGCCTTTTCCTCTTT

ACTTTCTATATGATAATATGCAATGATAGAGATGGAGTACTTGGGATCAGCAGAATTCTAAAACTACGAA

ACCAACTGGTTTCACACCGGGGGAACAACATCCAAA------GCAACGACCCCAACTGTTTGGAAGATAT

CTTGAGAAAAGGTTTTAGCACCGGTGTATCCTATATGTACTCTAGTTTATTCGAAGTATCCCAATGGTGT

AAGGCCGTCGACTTATTGGGAAAAAGGAAGAAAATCACTTTGATCTCTTGTTTCGGAGAAATAAGTGGCT

CACGAGGAATGGAAAGAAACATCTTCTATTTGATCTCGAAGTCTTCATATAGCACTTT------------

---TTCCAATCCTGGATGGGGGATCACTTGTAAGAATGAAATAATGCTAATCCATGTTCCACACGGCCAA

GGAAGCATCGTTTTTTAA-----

>Silene latifolia NC014487.1

-TGATACTTTCTGTTTTGTCGAGCCCAGCTTTGGTCTCTGGTTTGATGGTTGTACGTGCTAAAAATCCGG

TACATTCCGTTTTGTTTCCCATCCCAGTCTTTCGCAACACTTCAGGTTTACTTCTTTTGTTAGGTCTCGA

CTTTTCCGCTATGATCTTCTTAGTAGTTTATATAGGAGCTATAGCCGTTTCATTCCTATTCGTTGTTATG

ATGTTCCATATTCAAATAGCGGAGATTCACGAAGAAGTCTTGCGCTATTTACCAGTGAGTGGTATTATTG

GACTGATCTTTTGGTGGGAAATGTTCTTCATTTTAGATAATGAAACCATTCCATTACTACCAACCCAAAG

AAATACGACCTCTCTGAGATATACGGTTTATGCCGGAAAGGTACGAAGTTGGACTAATTTGGAAACATTG

GGCAATTTACTTTATACTTACTATTTTGTCTGGTTTTTGGTTTCTAGTCTTATTTTATTAGTAGCCATGA

TTGGGGCTATAGTACTGACTATGCATAGGACTACTAA------GGTGAAAAGACAGGATGTATTCCGACG

AAATGCTATTGATTCTAGAAGGACTATAATGAGGAGGACGACAGACC-ATGTCAATATATGAATTGTTTC

ATTATTCGTTATTTCCGGGTCTTTTCATTGCATTCACTTACAACAAGAAACAACCACCAGCGTTTGGTGC

AGCACCCGCATTTTGGTGTATTCTTTTTTCTTTCTTTGGTCTTTTGTTCTGTCATATTTCTAATAACTTA

TCCAATTACAACGTATTAACCGCTAATGCACCTTTCTTTTATCAAATCTCAGGGACATGGTCTAATCATG

AAGGTAGTATTTTATTATGGTGTCGGATCCCAAGTTTTTATGGATTCCTTCTTTGTTACCGGGGTCGATC

CCAAAGCCATAATGTCTCAAAACGAGGAGGCCATAGAGAAAGTCTTCTTTTTTCCTTTGTCTTAAACTTC

GTGAAGAACTCCATTCTATCTCTTCCTCGTTACGAACAAAAAAGTAGAGTTCTTCACGAACCCCAGTTGT

ACACTCTCTTCGTTCTACGAA---CTCTTGTTGATTCTGAACTTTGTTCGCGAAGGAAGCGGACTTTTGA

CGGGCCAGCTCTTTTTTACGCGCCGCTTTACCCTGAAAGGAAAATGAGCTTTGCTCTTCTGGCCGCGAGG

CGCTCTCGTGGTTCGCGAGAAGGAAAAAGGACTCATCCTTTGTTGCATCTGGCACGAGATGATAAAGAGA

GAGCTTCGTCTATCGATGAACAGCGGATTGACGGAGCTCTTGGCATTGCTTTGTTTTTCTTTCCTTTCTT

ATCAGCGAGTTCCGATCCTTTTGTTCGAAATTTCTTTGTTCGTACCGAACCGCTTGCAGAATCAAATCCT

GTTCCACAAGATCCTATATCAGCAATACACCCCCCTTGTATTTATGCCGGAGACGTCGCCAGTGCTATGG

GCTTTGGCTTATGTAGATCAAAAATGATGAATGGGATTGTGGCACTCTACTCGCCGCCAATGCGGAAGGA

TGCCGCCGAAAAGAATGGAACGCTACTTTGCTCTGCTGGATGCGTCGGATCCCGTATAACAAGCGAGCTC

TTTACCCTTAAATTCAAACATGTGGGCACCAAATGCTATCCTGCTTTATTGTTGCGTAGCAAAAGAAGCC

TGCTC---ATGCTGCTTCGGCGGCGCTTTTTCGCCTTCTCTTGGCTCTGGACAAGAGCGCTAGTGGACAC

GGGGGGGTTACGGTTCAAGGCGAAGCCTTTCTTTCGTATTCGTAATGGAAAGAAAGAGACCACTACTTTG

CCTCTTTGTTGGACCGCCGGCGCGAACACAGTGGTCTCTGACCAGGACCAGGAAGCTCTTAGAATTTGGA

TCTTGATATGTCGGTGCTTTTTAACCGTAGGCATCTTGCCAGGAAGTTGGTGGGCTCATCATGAATTAGG

TCGGGGTGGCTGGTGGTTTCGGGATCCCGTAGAAAATGCTTCTTTTATGCCTCGGGTATTAGCCACAGCT

CGTATTCATTCAGTCATTTTACCCCTTCTTCATTCTTGGACTTTGTTTCTTAATATTGTGACTTTTCTAT

GCTGTGTCTTAGGAACCTTTTCAATACGGTCCGGATTGCTAGCTCCCGTTCATAGTTTTGCTACAGATGA

TACACGAGGAATCTTTTTATGGCGGTTTTTCCTTCTAATGACCGGCATATCTATGATTCTTTTCTCTCAG

ATGAAGCAGCAGGCATCGGTCCGTAGAACCTATAAAAAAGAGATGGTTGTAGCGCGAAGTACTCTTGTGC

ACTTACG-----------------ATGGCTA---TTCACAATTGCTCCTTGTGATGCAGCGGAACCGTGG

CAATTAGGATTTCAAGACGCAGCAACACCTATGATGCAAGGAATAATAGACTTACATCATGATATCTTTT

TCTTCCTTATTCTTATTTTGGTTTTCGTATCATGGATCTTGGTTCGCGCTTTATGGCATTTCCACTATAA

AAAAAATCCAATCCCGCAAAGGATTGTTCATGGAACTACTATCGAGATTATTCGGACCATCTTTCCTAGT

ATCATCCTGATGTTCATTGCTATACCATCATTTGCTCTCTTATACTCAATGGACGAGGTAGTAGTAGATC

CAGCCATTACTATCAAAGCTATTGGACATCAATGGTATTGGACTTATGAGTATTCGGACTATAACAGTTC

CGATGAACAGTCACTCACTTTTGACAGTTATATGATTCCAGAAGATGATTTAGAATTGGGTCAATTACGT

TTATTAGAAGTGGACAATAGAGTGGTTGTACCAGCCAAAACTCATATACGTATTATTGTAACATCTGCTG

ATGTACTTCATAGTTGGGCTGTACCTTCCTTAGGTGTCAAATGTGATGCTGTACCTGGTCGTTTAAATCA

GACCTCTATTTTGGTACAACGAGAAGGAGTTTACTATGGTCAGTGCAGTGAAATTTGTGGAACTAATCAT

GCTTTTATGCCTATCGTCGTAGAAGCTGTTTCTAGGAAAGATTATGGTTCTCGGGTATCTACTCAATTAA

TCCCCCAAACCGGAGAAGCTTA-ATGAGACGACTCTTTTTTGAACTATATCATAAACAGATCTTTTTCTC

CACACCAATCACGACTTTTTCTTCATTCCTCTCGTATATTGTCGTAACGCCCTTAATGCTAGGTTTTGAA

AAAGACTTTTCATGTCATTTCCATTTAGGTCCGATTCGGATCTCTCCGTTGTTTCCTTTTCCTCCCGCAC

CTTTTCTTCGAAATGAGAAAGAAGATGGTACACTCGAATTGTATTATTTAAGTGCTTATTGCTTGCCAAA

GATCCTACTTCTACAATTGGTAGGTCACCGGGTTATTCAAATAAGTCGTGTTTTCTGTAGTTTTCCCATG

TTACAACTTTTGTACCAATTCGGCCAATCCGGAATGGATCGGTTAAACATTCTATTAGGGAGCCTGGTCT

TGACTCTTCTGTGTGGTATTCATTCTTGTTTGGCTCTTGGAATCACATCCAGCAGTGGTTGGAACAGCTC

GCAAAATTTAACCACTTCACCTACTTCATTGCCCTCAACCGTTTCTCGTACCTCTATTGAAACAGAATGG

TTTCATGTTCTTTCATCGATTGGTTATTTTTCTTCGTTCGTCTCTCTTTTTCCAATTTCGGTCTCGATTA

GTTCACAAGATTG--------TTTCTTTATTACAACCTTCTTTTTTTTTTTGATGTCAAAGACCAAAAGC

TACGCGCAAATTCTCATTGGATCTTGGTTGTTCTTAACAGCGATGGCTATTCATTTAAGTCTTTGGGTAG

CACCACTAGATTTTCAACAAGGTGGAAATTCTCGTATTCTCTATGTACATGTTCCTGTGGCTCGGATGAG

TATTCTTCTTTATATCGTTACGGCTATAAACACTTTCTTGTTCCTATTAACAAAACATCCTCTTTTTCTT

CGCTCTTCCGGAACCGGTACAGAAATGGGTGCTTTTTCTACGCTGTTAACTTTAGTAACTGGGGGGTTTC

GGGGAAGACCTATGTGGGGCACCTTTTGGGTGTGGGACGCTCGTTTAACTTCTGTATTCATCTCATTCCT

TATTTACTTGGGCGCACTGTGTTTTCAAAAGCTTTCTGTCGAACCGGCTCCTATTTCAATCCGTGCTGGA

CCGATCGATATACCAATAATAAAGTTTCCAGTCAACTGGTGGAATACATCGCATCAACCTGGGAGCATTA

GCCGATTTGGTACATCAATACATGTTCCTATGCTCATTCCAATCTTGTCTAACTTTGCTAACTTCCTCTT

CTCAACCCGTATCTTCTTTGTTCTGGAAACACGTCTTCCTATTCCATCTTTTCTCGAATCTCCTTTAACG

GAAGAAATAGAAGTTCGAGAAGGAATA--TGCAGGCTAGAAAGATGCTATTTGCTGCTATTCTATCTATT

TGTGCATCAAGTTCGAAGAAGATCTCAATCTATAATGAAGAAATGATAGTAGCTCGTTGTTTTATAGGCT

TTATCATATTCAGTCGGAAGAGTTTAGGTAAGACTTTCAAAGTGACTCTCGACGAGAGAATCCAGGCTAT

TCAGGAAGAATCGCAGCAATTCCCCAATCCTAACGAAGTAGTTCCTCCGGAATCCAATGAACAACAACGA

TTACTTAGGATCAGCTTGCGAATTTGTGGCACCGTAGTAGAATCATTACCAATGGCACGCTGTGCGCCTA

AGTGCGAAAAGACAGTGCAAGCTTTGTTATGTCGAAACCTAAATGTTAAGTCAGCAACACTTCCAAATGC

CACTTCTTCCCGTCGCACCCGTCTTCAGGACGATCTAGTCACAGGGTTTCACTTCTCAGTGAGTGAAAGA

TTTGTCCCCGGGTCTACGTTGAAAGCTTCTATAGTTGAACTCATTCGAGAAGGCTTGGCGGTCTTAAGAA

TGGTTCGGGTAGGAGGTTCTCT---TGAAAGAGGCGATCAGAATGGTACCCGAATCCATTTACGATCCCG

AGTTTCCAGACACATCGCACTTCCGCTCGGGTCGAGGCTGCCACTCGGCCCTAAGACGGATCAAAGAAGA

GTGGGGAACCTCTCGCTGGTTTTTGGAATTCGACATCAGGAAGTGTTTTCACACCATCGACCGACATCGA

CTCATACCAATCTTTAAGGAAGAGATCGACGATCCCAAGTTCTTTTACTCCATTCAGAAAGTCTTTTCCG

CCGGACGACTCGTAGGAGGTGAGAAGGGAAAAGACTCCGTCCCACACAGTGTACTACTATCGGCCCTACC

GGGCAACATCTACTTACACAAGCTCGATCAGGAGATAGGAAGGATCCGACAGAAGTACGAAATTCCTATT

GTTCAGAGAATCAGATCGGTTCTATTAAAGACAAGTCGTATTGATG------------------------

--ATGGAATTCTCTCCCAGAGCTGCGGAACTAACAACTCTATTAGAAAGTAGAATTAGCAACTTTTATAC

GAATTTTCAAGTGGATGAGATCGGTCGAGTGGTCTCAGTTGGAGATGGGATTGCACGTGTTTATGGATTG

AACGAGATTCAAGCTGGAGAAATGGTGGAATTTGCCAGCGGTGTGAAAGGAATAGCCTTAAATCTTGAGA

ATGAAAATGTAGGGATTGTTGTCTTTGGTAGTGATACCGCTATTAAAGAGGGCGATCTTGTCAAGCGCAC

TGGATCTATTGTGGATGTTCCTGCGGGAAAAGCTATGCTAGGGCGTGTGGTCGACGCGTTGGGAGTACCT

ATTGATGGAAGAGGGGCTCTAAGCGATCACGAGCGTCGACGTGTCGAAGTGAAAGCCCCTGGGATTATTG

AACGTAAATCAGTGCACGAGCCTATGCAAACCGGCTTAAAGGCGGTAGATAGCCTGGTTCCTATAGGCCG

TGGTCAACGAGAACTTATAATCGGGGACCGACAAACGGGAAAAACAGCTATTGCTATCGATACCATATTA

AACCAAAAGCAACTGAACTCAAAGGCCACTTCTGAGAGTGAGACATTGTATTGTGTTTATGTAGCGATTG

GACAGAAACGTTCAACTGTGGCACAATTAGTTCAAATTCTTTCAGAAGCGAATGCTTTGGAATATTCCAT

TCTTGTAGCAGCCACTGCTTCGGATCCTGCTCCTCTTCAATTTCTGGCCCCATATTCTGGATGTGCTATG

GGAGAATATTTCCGCGATAATGGAATGCACGCATTAATAATCTATGATGATCTTAGTAAACAGGCGGTGG

CATATCGACAAATGTCATTATTGTTACGCCGACCACCAGGCCGTGAGGCTTTCCCAGGCGACGTTTTCTA

TTTACATTCTCGTCTCTTAGAAAGAGCCGCTAAACGATCGGACCAGACAGGTGCAGGTAGCTTGACCGCC

TTACCCGTCATTGAAACACAAGCTGGAGACGTATCAGCCTATATTCCCACCAATGTGATCTCCATTACTG

ATGGACAAATCTGTTTGGAAACAGAGCTCTTTTATCGCGGAATTAGACCTGCTATTAACGTCGGCTTATC

TGTCAGTCGCGTCGGGTCTGCCGCTCAGTTGAAAGCTATGAAACAAGTCTGCGGGAGTCCAAAACTTGAA

TTGGCACAATATCGCGAAGTGGCCGCCTTTGCTCAATTTGGCTCAGACCTTGATGCTGCGACTCAGGCAT

TACTCAATAGAGGTGCAAGGCTTACAGAAGTACCGAAACAACAACAATATGCACCACTTCCAATTGAAAA

ACAAATTATAGTCATTTACGCAGCTGTCAATGGATTCTGTGATCGAATGCCACTAGATAAAATTTCTCAA

TATGAGAGAACCATTCCAAATAGTGTAAACCCAGAATTCTTACAATCCCT------AAAGGGCGGCTTAA

CTAACGAAAAAAAGATGGAACTAGATTCATTCTTAAAAGAATGCGCTTTGAATTAC--------------

--------CATCCAACGCAAAGCGGCCTTTCATTCCCTTGTTTCGTCGTGGCACAACCCCCCCGCAAGCA

CCCCCCGGCTCAGGGGGGACCAGACCTCTCCTTTCGTTTTGCCCCCTTCGTCGGCCCTTGCCGCCTTCCT

TAACAAGCCCTCGAGCCTCCTTTTCGCTGCCTTACTCATAGAAGCCCCCGGGTTGACCCCGAAGGCCGAA

TTCTATGGTAGAGAATGCTTTCATAATAATTGGGCCATGAGAGACCTTTTTAAGTATTGCAAAAGAAAGG

GCCTGCTGATAGAGCTGGGCGAGGCAGGGATACTCGTTCTCAGGTCAGATAAAGGCCTGGCCCGTAAGCT

GGCCCCCTTAAAAACCCATTACTTAATAAGGATTTGTTACGCGCGATATGCCGACGACTTACTATTTGGA

ATCGTGGGTGCCGTAGAGCTTCTCATAGAAATACAAAAACGTATCGCCCACTTCCTACAATCCGGCCTGA

ACCTTTGGGTAGGCTCTGCGGGATCAACAACAATAGCTGCACGGAGTACGGTAGAATTCCTCGGTACGGT

CATTCGGGAAGTCCCTCCGAAGACGACTCCCATACAATTCTTGCGAGAGCTGGAGAAGCGTCTACGGGTA

AAGTACCGTATCCATATAACTGCTTGCCACTTACGCTCTGCCATTCATTCCAAGTTTAGGAACCTAGGTA

AGAGTATACCGATCAAACAGCTGACGAAGGGGATGAGCAAAACAAGGAGTCTACTGGACGCGGTTCAACT

AGCGGAAACTCTTGGAACAGCTAGAGTAAGAAGCCCCCAAGTGAGCATATTATGGGAGACCGTCAAGCAC

ATCCGGCAAGGATCAAGGGAGATCTCGTTGTTGCATAGCTCAGGTCAGAGCAAGGTGCCACCGGACGTTC

AACAGGTAGTCTCGCGATCGGGCATGAGTGCCCGGAAGTTGTCGGAAAAAGAGACTCTCGCGGGTCGGAA

GGCGGCGGGGGAAGGAGGGGGACACTGGGCGAGATCTATCAGCAGCGAATTCCCCATACAGATAGAAGCG

CCTATCAAAAAGATACTCCGAAGGCTTCGAGATCGAGGTCTCATTAGCCGAAGAAGACCCTGGCCAATCC

ACGTGGCCTGCTTGACGAACGTCAGCGACGGAGACATCGTAAATTGGTTCGCGGGCATCGCGATAAGTCC

TCTGTCCTACTACAGGTGCCGCGACAACCTTTACCAAGTCCGAACGATTGTCGACTACCAGATCCGCTGG

TCTGCAATCTTCACCCTAGCCCACAAGCACAAATCCTCAGCGCGGAATATAATCCCAAAGTACTCCAAAG

ACTTAAATATAGTAAATAAAGAAGGTGGCAAGACCCTTGCAGAGTTCCCCAACAGCAGAGAGCTTGGAAA

GCTCGGACCCGGGCAAGATCC-GAACAACAAAGAGCACTCAACTACTA-----------------ATGGT

CCAACTACATAACTTTTTCTTTTTCATTACTTCCATGGTCGTGCCTTGTGGCACGGCAGCACCCGTACTA

TTGAAATGGTTCGTCAGTAGAGATGTTCCCACAGGTGCCCCTTTTTCCAATGGTACTTTAATTCCTATTC

TTATCCCTTCATTCCTTCTTTTGGTTTATCTACATTCCAGGAAATTCATACGCTCCATGGACAGAGTAAA

AAGTGGAGTCTTGGTCAGAGCAAGCTGCCCTATTTTAT------TACCAGACATAATTGGGAGAAGCTCC

TCCGAAACTAGAGCTAGAAACGCCTTATTTAGTTTCGTTCCCATTCTTCATTTTCTTCTTCTCGAATCTT

ATAACTTAGGGGACTTACCCTATTTAGAATCTTTTTGCGGTGTGCTCCGTTTACTATTCTTTCGTACTTT

CTTCTCTTTATCACGCGATAGGTCAGCGAAGCGTGAGCGGGCGCGGAGAAGAAAACGCCAAACACTTCGG

ACTAAT------GGGAATGAGCAACGACGAAATGACAAGATAAAGTGCCCCGGGCGCCCCCATT------

TAGA---AAGAAGGGTCGAAGGGTTTGGACCTGTAGCTTTCCCCGTGCCCCCTTCGTCGGGTGGTGCTTG

CATGGGGGGTGTGCTACCTGAAATCGGGCTTGAAGCTCCCGCCTTACCAACGAGCCGACAGCTGATGGCT

GTTGGTCACGACTACTACCAAAAAGTGAACATGAGGATGACTATTTCACATGGGGGAGTGTGCATCTTTA

TCTTGGGTGTTCTTCTG-ACGTACATAGCTGTTCCAGCGGAAATACTTGGAATAATTCTACCACTTCTAC

TAGGAGTAGCCTTTTTAGTGCTAGCTGAACGTAAAGTAATGGCTTTTGTGCAACGTCGAAAGGGTCCTGA

TGTAGTGGGATCGTTCGGATTGTTACAACCTCTAGCAGATGGTTCAAAATTGATTCTAAAAGAACCTATT

TCACCAAGTAGTGCTAATTTCTCCCTTTTTAGAATGGCTCCAGTCACTACATTTATGCTAAGTCTGGTTG

CTCGGGCCGTTGTACCTTTTGATTATGGTATGGTATTGTCAGATCCGAACATAGGGCTACTTTATTTGTT

TGCCATATCTTCGCTAGGTGTTTATGGAATTATTATAGCAGGTTGGTCTAGT-TTTATTATATACGTTTA

GTGAAAAGAATGTTTTTTGATACACCTAGGACATGGATTCTATATGAACCAATGGATCGTAACAAGTCGT

TACTACTAGCAATGACTTCCTCTTTCATTACTTCATCCTTTTCATATCCTTCTCCCTTGTTCTCAGTTAC

TCATCAAATGGCACTCAGTTTATATCTTTA-------GAATTTGCGCCTATTTGTATCTATTTAGTGATC

AGTCTGCTAGTTTCTTTGATCCCACTCGGTGTTCCTTTTCCATTTTCTTCTAATACTTCGACTTATCCAG

AAAAATTGTCGGCCTACGAATGTGGTTTCGATCCTTTCGGTGATGCCAGAAGTCGTTTCGATATACGATT

TTATCTTGTTTCAATTTTATTTATTATCCTTGATCCGGAAGTAACCTTTTTCTTTCCTTGGGCAGTACCT

CTCAACAAGATTGATCCGTTTGGATTTTGGTCCATGATGGCCTTTTTATTGATTTTAACGATAGGATTTC

TCTATGAATGGAAAAGGGGTGCTTTGGATCGGGAGTA-----------------------CCCAACAGCC

CACTTGACCAATTTGCCATTCTCCCATTGATTCCTATGAAACTAGGAAACTTGTATTTCTCATTCACAAA

TCCATCTTTGTTTATGCTGCTAACTCTCAGTTTGGTCCTACTTCTGCTTCATTTTGTGACTAAAAAGGGA

GGAGGAAACTCAGTACCAAATGTTTGGCAATCCTTGGTAGAGCTTATTTATGATTTCGTGCTGAACCTGG

TAAACGAACAAATAGGGGGTCTTTCCGGAAATGTTAAACAAAAGTTTTTCCCTTGCATCTTGGTCACTTT

TACTTTTTTGTTATTTCGTAATCTCCAGGGTATGATACCTTATAGCTTTACAGTTACAAGTCATTTTCTC

ATTACTTTGGGCCTTTCATTTTCAATTTTTATTGGCATTACTATAGTGGGATTTCAAAGAAATGGGCTTC

ATTTTTTAAGCTTCTCATTACCTGCAGGAGTCCCACTGCCGTTAGCACCTTTTTTAGTACTCCTTGAGCT

AATCCCTCATTGTTTTCGCGCATTAAGCTTAGGGATACGGTTATTTGCTAATATGATGGCCGGTCATAGT

TCAGTAAAGATTTTAAGTGGGTTCGCTTGGACTATGCTATGTATGAATGATCTTTTATATTTCATAGGAG

ATCTTGGTCCTTTATTTATAGTTCTTGCATTAACCGGTCTTGAATTAGGTGTAGCTATATTACAAGCTCA

TGTTTTTACGATCTTAATCTGTATTTACTTGAATGATGCTACAAATCTCCATCAAA--------------

----------------ATATGTGGGCACCTGATATCTATGAGGGTTCACCCACCCCGGTTACAGCATTCT

TTTCTATTGCGCCTAAAATCTCTATTTCTGCTAATATTTTACGTGTTTTTATTTATGGTTCCTATGGAGC

TACATTGCAACAAATTTTCTTTTTCTGCAGCATTGCTTCTATGATCTTAGGAGCACTGGCCGCCATGGCC

CAAACAAAAGTAAAAAGACTTCTAGCTCATAGTTCAATTGGACATGTAGGTTATATTCGTACTGGTTTCT

CATGTGGAACCATAGAAGGAATTCAATCACTACTAATTGGTCTCTTTATTTATGCATCAATGACGATAGA

TGCATTCGCTATAGTTTCAGCATTACGGCAAACCCGTGTTAAATATATAGCGGATTTGGGCGCTCTAGCC

AAAACGAATCCTATTTCGGCTATTACCTTCTCTATTACTATGTTCTCATACGCAGGAATACCCCCGTTAG

CCGGCTTTTGTAGTAAATTCTATTTGTTCTTCGCCGCTTTGGGTTGTGGGGCTTACTTCCTAGCCCCAGT

GGGAGTAGTGACTAGCGTTATAGGTTGT--GTTCGATAGCCCGACCGTAGTGATGTTAATTGTGGTTACA

TTCATAAGTAGCTTGGTCCATCTTTATTCTATTTCATATATGTCTGAGGATCCGCATAGCCCTCGATTTA

TGTGTTATTTATCCATTCTTACTTTTTTTATGCCAATGTTGGTGACTGGAGATAACTCTCTTCAATTATT

CTTGGGATGGGAGGGAGTAGGTCTTGCTTCATATTTGTTAATTCATTTTTGGTTTACACGACTTCAGGCA

GATAAAGCAGCTATAAAAGCTATGCTTGTCAATCGAGTAGGTGATTTTGGGTTAGCTCTTGGGATTTCGG

GTCGTTTTACTCTCTTTCAAACAGTAGACTTTTCTACCATTTTTTCTTGTGCTAGTGCCCCTAGAAATTC

TTGGATTTCTTGCAATATGAGATTGAATGCCATAACTCTTATTTGTATTTTACTTTTTATTGGTGCTGTT

GGAAAATCTGCACAGATAGGATCGCATACTTGGTCACCCGATGCTATGGAGGGTCCCACTCCAGTATCCG

CTTTGATTCATGCAGCTACTATGGTAACAGCTGGCGTTTTCATGATAGCAAGGTGTTCCCCTTTATTTGA

ATACCCACCTACGGCTTTAATTGTTATTACTTTTGCAGGAGCTATGACGTCATTCCTTGCGGCAACCACT

GGAATATTACAGAACGATCTAAAGAGGGTCATAGCTTATTCAACTTGCAGTCAATTAGGCTATATGATCT

TTGCTTGCGGCATTTCTAACTATTCGGTTAGCGTCTTTCATTTAATGAATCACGCCTTTTTCAAAGCATT

ACTATTCCTGAGTGCGGGTTCGGTGATTCATGCCATGTCGGATGAGCAAGATATGCGGAAGATGGGGGGG

CTCGCCTCCTCGTTCCCTTTTACCTATGCCATGATGCTCATGGGCAGCTTATCTCTAATTGGATTTCCTT

TTCTAACTGGATTTTATTCCAAAGATGTGATCTTAGAGCTCGCTTACACTAAGTATACCATCAGTGGGAA

CTTTGCTTTCTGGTTGGGAAGTGTCTCTGTCCTTTTCACTTCTTATTACTCTTTTCGTTCACTTTTTCTA

ACATTTCTAGTACCAACTAATTCATTCGGGCGAGACATCTTACGATGTCATGATGCGCCCATTCCTATGG

CCATTCCTTTAATACTTCTGGCTTTCGGGAGTCTCTTTGTAGGATACTTGGCCAAACTAACACAAAGAAG

ATACAGTTCACTCAACGATTGCCTTTGGGTTCCGAACTCCATATGGGGAAGGAACGTTGTTGTTTGCGGG

GTCTCGATCATTTACATGGACCCACTTTTCATTCCATTTGTGGGAATTTTTTTATCTATAAACCGTCCGT

AACGAACGATCGGCTCATCTT------TGAGCATGATGAATCACTTCGTGCCGACCTGTTGTCAATAAAC

TTTTGGGCCTCATATGAGAATGGAAAACTGGAGCATTTTCTGCATCGGTGGATGAAGAATCGCGAACATA

AAAATTTCTGGTTAAGCATGTTCCCAGAAAAAAGATACTTTCGAGAAACAACGAGCACGACTGAAGTGGC

TATCCATACAAATCCATTTACGGATCTATATGCTTCGATTGGAACTGGAAGTTCAAGAACAGGCGGCTGG

TATACTACTATAATGAAACTGCCTTTTATTTTTTTTATTCGGATAGGATTTCTGTTGGCTTCGTTGGGGG

GCTCGCGTAGTTTGTTACGTCAACTCCAAAAGGAGAAATTGCGTTGGAATTGAGAAAGTTACGTAA----

AGTTCATAATTGTATA--CAATTTTTGGGCCAATTCCCTCTTCGTACTACCAAAAAATGAGATTCTTGCC

GAATCCGAGTTTGCTGCTCCAACCATTACCAAACTAATACCTATTCTGTTTAGTACTTCAGGTGCTTCTA

TTGCGTATAATGTAAATCCCGTAGCGGATCAATTCC---------------AACGAGCCTTTCAAACTAG

TACTTTTTGTAATCGACTCTATAGCTTCTTCAATAAACGCTGGTTCTTCGATCAAGTTTTTAATGACTTT

CTAGTCAGATCGTTCTTGCGTTTTGGATATGAAGTCTCATTCGAAGCTTTAGACAAAGGTGCTATTGAGA

TATTGGGCCCCTATGGTATCTCGTACACATTCCGACGATTGGCCGAGCGAATAAGTCAACTTCAAAGTGG

ATTTGTTTTGTTCCATGATCTATGGGTCTACTGGAGCTACCCATTTCGATCAATTAGCCAAGATTTTGAC

CGGATACGAAATCACTGGTGTTCGATCTAGTGGTATTTTTATGGGGATTCTTTTTATCGCTGTAGGATTC

CTATTCAAGATCACTGCAGTTCCTTTT-TGGTCTATGCACATTGCTTTCTCCAGGAGGTTGGCCGCCTAT

CCTAGATCTTCCCATTTCCAAGAAGATCCCGGGCTCGATCTGGTTTAGTATCAAGGTGATTCTCTTTCTC

TTTCTATATATATGGGTCCGCGCAGCATTTCCACGATATCGTTATGATCAATTAATGGGACTTGGCCGGA

AAGTGTTCTTGCCTCTATCATTAGCTCGGGTAGTCGCCGTTTCTGGTGTTTTAGTCACCTTTCAATGGCT

CCCTTA-ATGCCTCAACTGGATAAATTTACTTATTTCACACAATTCTTCTGGTCATGCCTTTTTTTCTTT

ACTTTCTATATTCTAATATGCAATGATAGAGATGGAGTACTTGGGATCAGCAGAATTCTAAAACTACGAA

ATCAACTGCTTTCACACCGGGGGAACGACATCCAAA------GCAAGGACCCCAACAGTTTGGAAGATAT

CTTGAGAAAAGGTTTTAACACAGGTGTATCCTATATGTACTCTAGTTTATTCGAAGTATCCCAATGGTGT

AAGGCCGTCGACTTATTTGGAAAAAGGAAGAAAATAACTTTGATCTCTTGTTTCGGAGAAATAATTGGCT

CAGGAGGAATAGAAGGAAACATATTCTATTTGATCTTGGAGTCTTCATATAGCACTTCGTTCTGGGAATT

CCTTTCAAGGACGGGATGGGGGATCACTGGTAAGAATGACATAACGCTAATCCATGCTCTAAACGGCCAA

GAA--------------------

>Beta macrocarpa NC015994.1

-TGATACTTTCTGTTTTGTCGAGCCCGGCTTTGGTCTCTGGTTTGATGGTTGTACGTGCTAAAAATCCGG

TACATTCCGTTTTGTTTCCCATCCCAGTCTTTCGCAACACTTCAGGTTTACTTCTTTTGTTAGGTCTCGA

TTTTTCCGCTATGATCTTCCCAGTAGTTTATATAGGAGCTATAGCCGTTTCATTCCTATTCGTTGTTATG

ATGTTCCATATTCAAATAGCGGAGATTCACGAAGAAGTATTGCGCTATTTACCAGTGAGTGGTATTATTG

GACTGATCTTTTGGTGGGAAATGTTCTTCATTTTAGATAATGAAACCATTCCATTACTACCAACCCAAAG

AAATACGACCTCTCTGAGATATACGGTTTATGCCGGAAAGGTACGAAGTTGGACTAATTTGGAAACATTG

GGCAATTTACTTTATACTTACTATTTTGTCTGGTTTTTGGTTTCTAGTCTTATTTTATTAGTAGCCATGA

TTGGGGCTATAGTACTGACTATGCATAGGACTACTAA------GGTGAAAAGACAGGATGTATTCCGACG

AAATGCTATTGATTCTAGAAGGACTATAATGAAGAGGACGACAGACC-ATGTCAATATATGAATTGTTTC

ATTATTCGTTATTTCCGGGCCTTTTCATTGCATTCACTTACAACAAGAAACAACCACCAGCGTTTGGTGC

AGCACCTGCGTTTTGGTGTATTCTTCTTTCTTTCCTTGGTCTTTTGTTCTGTCATATTCCTAATAACTTA

TCCAATTACAACATATTAACCGCTAATGCACCTTTCTTTTATCAAATCTCAGGGACATGGTCTAATCATG

AAGGTAGTATTTTATTATGGTGTCGGATCCCAAGTTTTTATGGATTCCTTCTTTGTTACCGGGGTCGATC

CCAAAGCCATAATGTCTCAAAACGAGGAGGCCATAGAGAAAGTCTTCTTTTTTCCTTTGTCTTAAACTTC

GTGAAGAACTCCATTCTATCTCTTCCTCGTTACGAACAAAAAAGTAGAGTTCTTCACGAACCCCAGTTGT

ACACTCTCTTCGTTCTACGAA---CTCTTGTTGATTCTGAACTTTGTTCGCGAAGGAACCGGACTTTTGA

CGGGCCAGCTCTTTTTTACG------TTTACCCTGAAAGGAAAATGAGCTTTGCTCTTCTGGGCGCTAGG

CGCTCTCGTGGTTCGCGAGAAGGAAAAAGGACTCATCCTTTGTTGCATCTGGCACGAGATGATAAAGAGA

GAGCTTCGTCTATCGATGAACAGCGGATTGACGGAGCTCTTGGCATTGCTTTCTTTTTCTTTCCTTTCCT

ATCAGCGAGTTCCGATCCTTTTGTTCGAAATTTCTTCGTTCGTACCGAACCGCTTGCAGAATCAAATCCT

GTTCCACAAGATCCTATATCAGCTATACATCCTCCTTGCATTTATGCCGGAGACGTCGCCAGTGCTATGG

GCTTTGGCTTATGTAGATCAAAAATGATGAATGGGATTGTGGCACTCCACTCGCCGCCAATGCGGAAGGA

TGTCGCCGAAAAGAATGGAACGCTGCTTTGCTCTGCTGGATGCGTCGGATCCCGTATAACAAGCGAGCTC

TTTACCCTTAAATTCAAACATGTGGGCGCCAAATGCTATCCTGCTCTATTGTTGCGTAGCAAAAGAAGCC

TGCTC---ATGCTGCTTCGGCGGCGCTTTTTCGCCTTCTCTTCGCTCTGGACAAGAGCGCTAGTGGACAC

GGGGAGGGAGCG------GGCGAAGCGTTTCT------TTCGTAATGGAAAGAAAAAGACCACTACTTTG

CCTCTTTGTTGGACCGCCGGCGCGAACACAGTGGTCTCTGACCAGGACCAGGAACCAATTCGAATTTGGA

TCTTGACATGTCGGTGCTTTTTAACCGTAGGCATCTTGCCAGGAAGTTGGTGGGCTCATCATGAATTAGG

TCGGGGTGGCTGGTGGTTTCGGGATCCCGTAGAAAATGCTTCTTTTATGCCTCGGGTATTAGCCACAGCT

CGTATTCATTCAGTCATTTTACCCCTTCTTCATTCTTGGACTTTGCTTCTTAATATTGTGACTTTTCTAT

GCTGTGTCTTAGGAACCTTTTCAATACGGTCCGGATTGCTAGCTCCCGTTCATAGTTTTGCTACAGATGA

TACACGAGGAATCTTTTTATGGCGGTTCTTCCTTCTAATGACCGGCATATCTATGATTCTTTTCTCTCAG

ATGAAGCAGCAGGCATCGGTCCGTAGAACCTATAAAAAAGAGATGGTTGTAGCGCGAAGTACTCTTGTGC

ACTTACG----TGATTGTTCGAGAATGGCTATTCTTCACAATGGCTCCTTGTGATGCAGCGGAACCATGG

CAATTAGGATTTCAAGACGCAGCAACACCTATGATGCAAGGAATAATCGACTTACATCATGATATCTTTT

TCTTCCTCATTCTTATTTTGGTTTTCGTATCATGGATCTTGGTTCGCGCTTTATGGCATTTCCACTATAA

AAAAAATCCAATCCCGCAAAGGATTGTTCATGGAACTACTATCGAGATTATTCGGACCATCTTTCCCAGT

ATCATCCTGATGTTCATTGCTATACCATCATTTGCTCTGTTATACTCAATGGACGAGGTAGTAGTAGATC

CAGCCATTACTATCAAAGCTATTGGACATCAATGGTATCGGAGT--------------------------

----------------------------------------------------------------------

----------------------------------------------------------------------

----------------------------------------------------------------------

----------------------------------------------------------------------

----------------------------------------------------------------------

-----------------------ATGAGACGACTCTTTTTTGAACTATATCATAAACAGATCTTCTTCTC

CACACCAATCACGAGTTTTTCTCCATTCCTCTCGTATATTGTCGTAACGCCCTTAATGCTAGGTTTTGAA

AAAGACTTTTCATGTCATTTCCATTTAGGTCCGATTCGGATCCCTCTGTTGTTTCCTTTTCCCCCCGCAC

CTTTTCTTCGAAATGAGAAAGAAGATGGTACACTCGAATTGTATTATTTAAGTGCTTATTGCTTGCCAAA

GATCCTACTTCTACAATTGGTAGGTCACCGGGTTATTCAAATAAGTCGTGTTTTCTGTAGTTTTCCCATG

TTACAACTTCTGTACCAATTCGGTCAATCCGGAATGGATCGGTTAAACATTCTATTAGGGAGCCTGGTCT

TGACTCTTCTGTGTGGTATTCATTCTCGTTTGGCTCTTGGAATCACATCCAGCAGTGGTTGGAACAGCTC

GCAAAATTTAACCACTTCACCTACTTCATTGCCCTCAACCGTTTCTCGTACCTCTATTGAAACAGAATGG

TTTCATGTTCTTTCATCGATTGGTTATTTTTCTTCGTTCGTCTCTCTTTTTCCAATTTCGGTCTCGATTA

GTTCACAAGATTG---------TTCTTTATTACAACCTTC-----TTTTTTGATGTCAAAGACCAGGAAC

TACGCGCAAATTCTCATTGGATCTTGGTTGTTCTTAACAGCGATGGCTATTCATTTAAGTCTTTGGGTAG

CACCACTAGATTTTCAACAAGGTGGAAATTCTCGTATTCTCTATGTACATGTTCCTGTGGCTCGGATGAG

TATTCTTGTTTATATCGTTACGGCTATAAACACTTTCTTGTTCCTATTAACAAAACATCCTCTTTTTCTT

CGCTCTTCCGGAACCGGTACAGAAATGGGTGCTTTTTCTACGTTGTTTACCTTAGTTACTGGGGGGTTTC

GGGGAAGACCCATGTGGGGGACCTTTTGGGTGTGGGATGCTCGTTTAACTTCTGTATTCATCTTGCTCCT

TATTTACTTGGGTGCGCTGTGTTTTCAAAAGCTTCCTGTAGAACCGGCTCCTATTTCAATCCGTGCTGGA

CCGATCGATATACCAATAATCAAGTTTCCAGTCAACTGGTGGAATACATCGCATCAACCTGGGAGCATTA

GCCGATCTGGTACATCAATACATGTTCCTATGCTCATTCCAATCTTGTCTAACTTTGCTAACTTCCTCTT

CTCAACCCGTCTATTATTTGTTATTGAAATACGTCTTCCTATTCCATCTTTTCTCGAATCTCCTTTAACG

GAAGAAATAGAAGCTCGAGAAGG-------GCAGGCTAGAAAGATGCTATTTGCTGCTATTCTATCTATT

TGTGCATCAAGTTCGAAGAAGATCTCAATCTATAATGAAGAAATGATAGTAGCTCGTTGTTTTATAGGCT

TTATCATATTCAGTCGGAAGAGTTTAGGTAATACTTTCAAAGTGACTCTCGACGAGAGAATCCAGGCTAT

TCAGGAAGAATCGCAGCAATTCCCCAATCCTAACGAAGTAGTTCCTCCGGAATCCAATGAACAACAACGA

TTACTTAGGGTCAGCTTGCGAATTTGTGGAACCGTAGTAGAATCATTACCAATGGCACGCTGTGCGCCTA

AGTGCGAAAAAACAGTGCAAGCTTTGTTATGTCGAAACCTAAATGTTAAGTCAGCAACACTTCCAAATGC

CACTTCTTCCCGTCGCACCCGTCTTCAGGATGATCTAGTCACAGGTTTTCACTTCTCAGTGAGTGAAAGA

TTTGTCCCCGGGTCTACGTTGAAAGCTTCTATAGTAGAACTCATTCGAGAAGGCTTGGCGGTCTTAAGAA

TGGTTCGGGTAGGAGGTTCTCTT--TGAAAGAGGCGATCAGAATGGTACCCGAATCCATTTACGATCCCG

AGTTTCCAGACACATCGCACTTCCGCTCGGGTCGAGGCTGCCATTCGGCCCTAAGACGGATTAAAGAAGA

GTGGGGAACCTCTCGCTGGTTTTTGGAATTCGACATCAGGAAGTGTTTTCACACCATCGACCGACATCGA

CTCATCCCAATCTTTAAGGAAGAGATCGACGATCCCAAGTTCTTTTACTCGATTCAGAAAGTCTTTTCTG

CCGGACGACTCGTAGGAGGTGAGAAGGGCCCTTACTCCGTCCCACACAGTGTACTACTATCGGCCCTACC

AGGCAACATCTACTTACACAAGCTCGATCAGGAGATAGGGAGGATTCGACAGAAGTACGAAATTCCGATT

GTTCAGAGAATCAGATCGGTTCTATTAAAGACAAGTCGTATTGATGACCAAGAAAACTCTGGAGAAGAAG

--ATGGAATTCTCTCCCAGAGCTGCGGAACTAACGAATCTATTAGAAAGTAGAATTACCAACTTTTACAC

GAATTTTCAAGTGGATGAGATCGGTCGAGTGGTCTCAGTTGGAGATGGGATTGCACGTGTTTATGGATTG

AACGAGATTCAAGCTGGGGAAATGGTGGAATTTGCCAGCGGTGTGAAAGGAATAGCCTTAAATCTTGAGA

ATGAGAATGTAGGGATTGTTGTCTTTGGTAGTGATACCGCTATTAAAGAGGGAGATCTTGTCAAGCGCAC

TGGATCTATTGTGGATGTTCCTGCGGGAAAGGCTATGCTAGGGCGTGTGGTCGACGCGTTGGGAGTACCT

ATTGATGGAAGAGGGGCTCTAAGCGATCACGAGCGTCGACGTGTCGAAGTGAAAGCCCCCGGGATTATTG

AACGTAAATCTGTGCACGAGCCTATGCAAACCGGGTTAAAGGCGGTAGATAGCCTGGTTCCTATAGGCCG

TGGTCAACGAGAACTTATAATCGGGGACCGACAAACGGGAAAAACAGCTATTGCTATCGATACCATATTA

AACCAAAAGCAACTGAACTCAAAGGCCACCTCTGAGAGTGAGACATTGTATTGTGTCTATGTAGCGGTTG

GACAGAAACGTTCAACTGTGGCACAATTAGTTCAAATTCTTTCAGAAGCGAATGCTTTGGAATATTCCAT

TCTTGTAGCAGCCACCGCTTCGGATCCTGCTCCTCTTCAATTTCTGGCCCCATATTCTGGGTGTGCTATG

GGAGAATATTTCCGCGATAATGGAATGCACGCATTAATAATCTATGATGATCTTAGTAAACAGGCGGTGG

CATATCGACAAATGTCATTATTGTTACGCCGACCACCAGGCCGTGAGGCTTTCCCAGGCGACGTTTTCTA

TTTACATTCCCGTCTCTTAGAAAGAGCCGCTAAACGATCGGACCAGACAGGCGCAGGTAGCTTGACCGCC

CTACCCGTCATTGAAACACAAGCTGGAGACGTATCGGCCTATATTCCCACCAATGTGATCTCCATTACTG

ATGGACAAATCTGTTTGGAAACAGAGCTCTTTTATCGCGGAATTAGACCTGCTATTAACGTCGGCTTATC

TGTCAGTCGCGTCGGGTCTGCCGCTCAGTTGAAAGCTATGAAACAAGTCTGCGGTAGTCCAAAACTGGAA

TTGGCACAATATCGCGAAGTGGCCGCCTTTGCTCAATTTGGGTCAGACCTTGATGCTGCGACTCAGGCAT

TACTCAATAGAGGTGCAAGGCTTACAGAAGTACCGAAACAACCACAATATGCACCACTTCCAATTGAAAA

ACAAATTCTAGTCATTTACGCAGCTGTCAATGGATTCTGTGATCGAATGCCACTAGATAAAATTTCTCAA

TATGAGCGAACCATTCCAAATAGTGTAAAACCAGAATTATTACAATCCCT------TAAGGGGGGGTTAA

CTAACGAAAAAAAGATGGAACTAGATTCCTTCTTAAAAGAATGCGCTTTGAATTACTA------------

--------CATCCAACGCAAAGCGGCCTTTCATTCCCTTGTTTCGTCGTGGCACACCCTCCCCGCAAGCA

CCCCCCGGCTCAGGGGGGACCAGAAAAGGCCTTTCGTTTTCCCCCCTTCGTCGGCCCTTACCGCCTTCCT

TAACAAGCCCTCGAGCCTCCTTTTCGCTGCGTTCCTCATAGAAGCCGCCGGGTTGACCCCGAAGGCCGAA

TTCTATGGTAGAGAATGCTGTAATAATAATTGGGCCATGAGAGACCTTTTTAAGTATTGCAAAAGAAAGG

GCCTGCTGATAGAGCTGGGCGAGGCAGCGATACTAGTTATCAGGTCAGAGAAAGGCCTGGCCCGTAAGCT

GGCCCCCTTTAAAACCCATTACTTAATAAGGATTTGTTACGCGCGATATGCCGACGACTTACTATTGGGA

ATCGTGGGTGCCGTAGAGCTTCTCATAGAAATACAAAAACGTATCGCCCACTTCCTACAATCCGGCCTGA

ACCTTTGGGTAGGCTCTGCGGGATCAACAACAATAGCTGCACGGAGTACGGTAGAATTCCTCGGTACGGT

CATTCGGGAAGTCCCTCCGAAGACGACTCCCATACAATTTTTGCGAGAGCTGGAGAAGCGTCTACGGGTA

AAGCACCGTATCCATATAACTGCTTGCCACTTACGCTCTGCCATTCATTCCAAGTTTAGGAACCTAGGTA

ATAGTATCCCGATCAAACAGCTGACGAAGGGGATGAGCAAAACAGGGAGTCTACTGGACGCGGTTCAACT

AGCGGAAAGTCTTTCCACAGCTAGAGTAAGAAGTCCCCAAGTGAGCGTATTATGGGAGACCGTCAAGCAC

ATCCGGCAAGGATCAAGGGAGATCTCGTTGTTGCATAGCTCAGGTCAGAGCAAGGTGCCATCGGACGTTC

AACAGGCAGTCTTGCGATCGGGCATGAGTGTCCGGAAGTTGTC---ATTGTATACTCTCGCGGGTCGGAA

GGCGGCGGGGGAAGGAGGGGGACACTGGTCGAGATCTATCAGCAGCGAATTCCCCATACAGATAGAAGCG

CCTATCAAAAAGATACTCCGAAGGCTTCGAGATCGAGGTCTCATTAGCCGAAGAAGACCCTGGCCAATCC

ACGTGGCCTGCTTGACGAACGTCAGCGACGGAGACATCGTAAATTGGTCCGCGGGCATCGCGATAAGTCC

TCTGTCCTACTACAGGTGCCGCGACAACCTTTACCAAGTCCGAACGATTGTCGACTACCAGATCCGCTGG

TCTGCAATATTCACCCCAGCCCACAAGCACAAATCCTCAGCGCGGAATATAATCCAAAAGTACTCCAAAG

ACTTAAATATAGTAAATCAAGAAGGTGGCAAGACCCTTGCGGAGTTCCCCAACAGCATAGAGCTTGGGAA

GCTCGGACCCGGTCAAGATCC-GAACAACAAAGAGCACTCAACTACTA-----------------ATGGT

CCAACTACATAACTTTTTCTTTTTCATTACTTCCATGGTCGTGCCTTGTGGCACGGCAGCACCCGTACTA

TTGAAATGGTTCGTCAGTAGAGATGTTTCCACAGGTGCCCCTTTTTCCAATGGTACTTTAATTCCTATTC

TTATCCCTTCATTCCTTCTTTTGGTTTATCTACATTCCAGGAAATTCATACGCTCTATGGACGGAGTAAA

AAGTGGAGTCTTGGTCAGAGCAAGTTGCCCTATTTTAT------TACCAGACATAATTGGGAGAAGCTCA

TCCGAAACTAGAGCTAGAAACGCCTTATTTCGTTTCGTTCCCATTCTTCATTTTCTTCTTCTCGAATTCA

A------GGGGGACTTCCCCTATTTAGAATCTTTTTGCGGTGTGCTCCGTTTACTATTCTTTCGTACTTT

CTTCTCTTTACCACGCGATAGGTCAGCGAAGCGTGAGCGGGCGCGGAGAAGAGAAGGCCAAACACTTCGG

CCTAAC------GGGAATGAGCAACGACGAAATGAAAAGAGAAAGTGCCCCGGGCGCCCCCATT------

TAGA---AAGAAGGGTCGAAGGGTTTGGGCCTGTAGCTTTCCCCGTCCCCCCTTCGTCGGGTGGTGCTTG

CATGGGGGGTGTGCTACCTGAAATCGGGCTTGAAGCTCCCGCCTTACCAACGAGCCGACAGCTGATGGCT

GTTGGTCACGACTACTACCAAAAGGTGAACATGAAGATGAATATTTCACATGGGGGAGTGTGCATCTTTA

TGTTGGGTGTTCTTCTG--CGTACATAGCTGTTCCAGCTGAAATACTTGGAATAATTCTACCACTTCTAC

TAGGAGTAGCCTTTTTAGTGCTAGCTGAACGTAAAGTAATGGCTTTTGTGCAACGTCGAAAGGGTCCTGA

TGTAGTGGGATCGTTCGGATTGTTACAACCTCTAGCAGATGGTTCGAAATTGATTCTAAAAGAACCTATT

TCACCAAGTAGTGCTAATTTCTCCCTTTTTAGAATGGCTCCAGTCACTACATTTATGCTAAGTCTGGTTG

CTCGGGCCGTTGTACCTTTTGATTATGGTATGGTATTGTCAGATCCGAACATAGGGCTACTTTATTTGTT

TGCCATATCTTCGCTAGGTGTTTATGGAATTATTATAGCAGGTTGGTCTAGTA-TTATTATATACGTTTA

GTGAAAAGAATGTTTTTTGATACACCTAGGACATGGATTCTATATGAACCAATGGATCGTGACAAGTCGT

TACTACTAGCAATGACTTCCTCTTTCATTACTTCATCCTTTCCATATCCTTCTCCCTTGTTCTCAGTTAC

TCATCAAATGGCACTCAGTTTATATCTTTAA-TGTCAGAATTTGCGCCTATTTGTATCTATTTAGTGATC

AGTCTGCTAGTTTCTTTGATCCCACTCGGTGTTCCTTTTCCATTTTCTTCTAATACTTCGACTTATCCAG

AAAAATTGTCGGCCTACGAATGTGGTTTCGATCCTTTCGGTGATGCCAGAAGTCGTTTCGATATACGATT

TTATCTTGTTTCAATTTTATTTATTATCCTTGATCCGGAAGTAACCTTTTTCTTTCCTTGGGCAGTACCT

CTCAACAAGATTGATCCGTTTGGATCTTGGTCCATGATGGCCTTTTTATTGATTTTAACGATAGGATTTC

TCTATGAATGGAAAAGGGGTGCTTCGGATCGGGAGTAAAAAAGTGTTTATTACGATTACGCCCAACAGCC

CACTTGAGCAATTTTCCATTCTCCCATTGATTCCTATGAAAATAGGAAACTTGTATTTCTCATTCACAAA

TCCATCTTTGTTTATGCTGCTAACTCTCAGTTTGGTCCTACTTCTGCTTCATTTTGTTACTAAAAAGGGA

GGAGGAAACTCAGTACCAAATGTTTGGCAATCCTTGGTAGAGCTTATTTATGATTTCGTGCTGAACCTGG

TAAACGAACAAATAGGTGGTCTTTCCGGAAATGTTAAACAAAAGTTTTTCCCTTGCATCTTGGTCACTTT

TACTTTTTTGTTATTTCGTAATCTCCAGGGTATGATACCCTATAGCTTTACAGTTACAAGTCATTTTCTC

ATTACTTTGGGTCTTTCATTTTCCATTTTTATTGGCATTACTATAGTGGGATTTCAAAGAAATGGGCTTC

ATTTTTTAAGCTTCTCATTACCTGCAGGAGTCCCGCTGCCGTTAGCACCTTTTTTAGTACTCCTTGAGCT

AATCCCTCATTGTTTTCGCGCATTAAGCTCAGGAATACGTTTATTTGCTAATATGATGGCCGGTCATAGT

TCAGTAAAGATTTTAAGTGGGTTCGCTTGGACTATGCTATGTATGAATGATCTTTTATATTTCATAGGAG

ATCTTGGTCCTTTATTTATAGTTCTTGCATTAACCGGTCTTGAATTAGGTGTAGCTATATTACAAGCTCA

TGTTTTTACGATCTTAATCTGTATTTACTTGAATGATGCTACAAATCTCCATCAA---------------

-----------------TATGTGGGCACCTGATATCTATGAGGGTTCACCCACCCCGGTTACAGCATTCT

TTTCTATTGCGCCTAAAATATCTATTTCTGCTAATATTTTACGTGTTTTTATTTATGGTTCCTATGGAGC

TACATTGCAACAAATCTTCTTTTTCTGCAGCATTGCTTCTATGATCTTAGGAGCACTGGCCGCCATGGCC

CAAACGAAAGTAAAAAGACTTCTAGCTCATAGTTCAATTGGACATGTAGGTTATATTCGTACTGGTTTCT

CATGTGGAACCATAGAAGGAATTCAATCACTACTAATTGGTCTCTTTATTTATGCATCAATGACGATAGA

TGCATTCGCTATAGTTTCAGCATTACGGCAAACACGTGTCAAATATATAGCGGATTTGGGCGCTCTAGCC

AAAACGAATCCTATTTCGGCTATTACCTTCTCTATTACTATGTTCTCATACGCAGGAATACCCCCGTTAG

CCGGCTTTTGTAGTAAATTCTATTTGTTCTTCGCCGCTTTGGGTTGTGGGGCTTACTTCCTAGCCCCAGT

GGGAGTAGTGACTAGCGTTATAGGTTGTT-GTTCGATAGCCCGACCGTAGTGATGTTAATTGTGGTTACA

TTCATAAGTAGCTTGGTCCATCTTTATTCTATTTCATATATGTCTGAGGATCCGCATAGCCCTCGATTTA

TGTGTTATTTATCCATTCTTACTTTTTTTATGCCAATGTTGGTGACTGGAGATAACTCTCTTCAATTATT

CTTGGGATGGGAGGGAGTAGGTCTTGCTTCATATTTGTTAATTCATTTTTGGTTTACACGACTTCAGGCA

GATAAAGCAGCTATAAAAGCTATGCTTGTCAATCGAGTAGGTGATTTTGGATTAGCTCTTGGGATTTCGG

GTTGTTTTACTCTCTTTCAAACAGTAGACTTTTCTACCATTTTTGCTTGTGCTAGTGCCCCTAGAAATTC

TTGGATTTTTTGCAATATGAGATTGAATGCCATAACTCTTATTTGTATTTTACTTTTTATTGGTGCTGTT

GGAAAATCTGCACAGATAGGATCGCATACTTGGTCACCCGATGCTATGGAGGGTCCCACTCCAGTATCCG

CTTTGATTCATGCAGCTACTATGGTAACAGCTGGCGTTTTCATGATAGCAAGGTGTTCCCCTTTATTTGA

ATACCCACCTACGGCTTTAATTGTTATTACTTTTGCAGGAGCTATGACGTCATTCCTTGCGGCAACCACT

GGAATATTACAGAACGATCTAAAGAGGGTCATAGCTTATTCAACTTGCAGTCAATTAGGCTATATGATCT

TTGCTTGCGGCATTTCTAACTATTCGGTTAGCGTCTTTCATTTAATGAATCACGCCTTTTTCAAAGCATT

ACTATTCCTGAGTGCAGGTTCGGTGATTCATGCCATGTCGGATGAGCAAGATATGCGGAAGATGGGGGGG

CTCGCCTCCTCGTTCCCCTTTACCTATGCCATGATGCTCATGGGCAGCTTATCTCTAATTGGATTTCCTT

TTCTAACTGGATTTTATTCCAAAGATGTGATCTTAGAGCTCGCTTACACTAAGTATACCATCAGTGGGAA

CTTTGCTTTCTGGTTGGGAAGTGTCTCTGTCCTTTTCACTTCTTATTACTCTTTTCGTTTACTTTTTCTA

ACATTTCTAGTACCAACTAATTCATTCGGGCGAGACATCTTACGATGTCATGATGCGCCCATTCCTATGG

CCATTCCTTTAATACTTCTGGCTTTCGGGAGTCTCTTTGTAGGATACTTGGCCAAACTAACACAAAGAAG

ATACAGTTCACTCAACGATTGCCTTTGGGTTCCGAACTCCATATGGGGAAGGAACGTTGTTGTTTGCGGG

GTCTCGATCATTTACATGGACCCACTTTTCATTCCATTTGTGGGAATTTGATGATCTATAAACCGTCCTT

AACGAACGATCGGCTCATCTT------TGAGCATGATGAATCACTTCGTGCCGACCTGTTGTCAATAAAC

TTTTGGGCCTCATATGAGAATGGAAAACTGGAGCATTTTCTGCATCGGTGGATGAAGAATCGCGAACATC

CAAATTTCTGGTTAAGCATGTTCCCAGAAAAAAGATACTTTCGAGAAACAACGAGCACGACTGAAGTGGC

TATCCATACAAATCCATTTACGGATCTATATGCTTCGATTGGAACTGGAAGTTCAAGAACAGGCGGCTGG

TATACTACCATAATGAAACTGCCTTTTCTTTTTTTTATTCGGATAGGATTTCTGTTGGCTTCGCTGGGAG

GCTCGCGTAGTTTGTTACGTCAACTCCAAAAGGAGAAATTGCGTTGGAATTGAGAAAGTTACGTAA----

AGTTCATAATTGTATA-CCAATTTTTGGGCCAATTCCCTCTTCGTACTACCAAAAAATGAGATTCTTGCC

GAATCCGAGTTTGCTGCTCCAACCATTACCAAACTAATACCTATTCTGTTTAGTACTTCAGGTGCTTCTG

TTGCGTATAATGTAAATCCCGTAGCGGATCAATTCC---------------AACGAGCCTTTCAAACTAG

TACTTTTTGTAATCGACTCTATAGCTTCTTCAATAAACGCTGGTTCTTCGATCAAGTTTTGAATGACTTT

ATAGTCAGATCGTTCTTGCGTTTCGGGTATGAAGTCTCATTCGAAGCTTTAGACAAAGGTGCTATTGAGA

TATTGGGCCCCTATGGTATCTCGTACACATTCCGACGATTGGCCGAGCGAATAAGTCAACTTCAAAGTGG

ATTTGT--TGTTCCATGATCTATGGGTCTACAGGAGCTACCCATTTCGATCAATTAGCCAAGATTTTGAC

CGGATACGAAATCACTGGTGTTCGATCTAGTGGTATTTTTATGGGGATTCTTTTTATCGCTGTAGGATCC

CTATTCAAGATCACTGCAGTTCCTTTTCTGGTCTATGCACATCGCTTTCTCCAGGAGGTTGGCCGCCTAT

CCTAGATCTTCCCATTTCCAAGAGGATCCCGGGCTCGATCTGGTTTAGTATCAAGGTGATTCTCTTTCTC

TTTCTATATATATGGGTCCGTGCAGCATTTCCACGATATCGTTATGATCAATTAATGGGACTTGGCCGGA

AAGTGTTCTTGCCTCTATCATTAGCTCGGGTAGTCGCCGTTTCTGGTGTTTTAGTCACCTTTCAATGGCT

CCCTTA-ATGCCTCAACTGGATCAATTTACTTATTTCACACAATTCTTCTGGTCATGCCTTTTCTTCTTG

ACTTTCTATATTCTAATATGCAATGATAGAGATGGAGTACTTGGGATCAGCAGAATTCTAAAACTACGAA

ATCAACTGCTTTCACACCGGGGGAACAACATCCAAA------GCAAGGACCCAAACAGTTTGCAAGATAT

CTTGAGAAAGGGTTTTAACACAGGTGTATCCTATATGTACTCTAGTTTATTCGAAGTATCCCAATGGTGT

AAGGCCGTCGACTTATTTGGAAAAAGGAAGAAAATCACTTTGATCTCTTGTTTCGGAGAAATAAGTGGCT

CACGAGGAATGGAAAGAAACATATTCTATTTGATCTCGAAGTCTTCATATAGCACTTC------------

---TTCCAATCCTGGATGGGTGATCACTTGTAAGAATGACATAATGCTAATCCATGTTCTACACGGCCAA

GAA--------------------

>Beta vulgaris NC015099.1

-TGATACTTTCTGTTTTGTCGAGCCCGGCTTTGGTCTCTGGTTTGATGGTTGTACGTGCTAAAAATCCGG

TACATTCCGTTTTGTTTCCCATCCCAGTCTTTCGCAACACTTCAGGTTTACTTCTTTTGTTAGGTCTCGA

TTTTTCCGCTATGATCTTCCCAGTAGTTTATATAGGAGCTATAGCCGTTTCATTCCTATTCGTTGTTATG

ATGTTCCATATTCAAATAGCGGAGATTCACGAAGAAGTATTGCGCTATTTACCAGTGAGTGGTATTATTG

GACTGATCTTTTGGTGGGAAATGTTCTTCATTTTAGATAATGAAACCATTCCATTACTACCAACCCAAAG

AAATACGACCTCTCTGAGATATACGGTTTATGCCGGAAAGGTACGAAGTTGGACTAATTTGGAAACATTG

GGCAATTTACTTTATACTTACTATTTTGTCTGGTTTTTGGTTTCTAGTCTTATTTTATTAGTAGCCATGA

TTGGGGCTATAGTACTGACTATGCATAGGACTACTAA------GGTGAAAAGACAGGATGTATTCCGACG

AAATGCTATTGATTCTAGAAGGACTATAATGAAGAGGACGACAGACC-ATGTCAATATATGAATTGTTTC

ATTATTCGTTATTTCCGGGCCTTTTCATTGCATTCACTTACAACAAGAAACAACCACCAGCGTTTGGTGC

AGCACCTGCGTTTTGGTGTATTCTTCTTTCTTTCCTTGGTCTTTTGTTCTGTCATATTCCTAATAACTTA

TCCAATTACAACATATTAACCGCTAATGCACCTTTCTTTTATCAAATCTCAGGGACATGGTCTAATCATG

AAGGTAGTATTTTATTATGGTGTCGGATCCCAAGTTTTTATGGATTCCTTCTTTGTTACCGGGGTCGATC

CCAAAGCCATAATGTCTCAAAACGAGGAGGCCATAGAGAAAGTCTTCTTTTTTCCTTTGTCTTAAACTTC

GTGAAGAACTCCATTCTATCTCTTCCTCGTTACGAACAAAAAAGTAGAGTTCTTCACGAACCCCAGTTGT

ACACTCTCTTCGTTCTACGAA---CTCTTGTTGATTCTGAACTTTGTTCGCGAAGGAACCGGACTTTTGA

CGGGCCAGCTCTTTTTTACG------TTTACCCTGAAAGGAAAATGAGCTTTGCTCTTCTGGGCGCTAGG

CGCTCTCGTGGTTCGCGAGAAGGAAAAAGGACTCATCCTTTGTTGCATCTGGCACGAGATGATAAAGAGA

GAGCTTCGTCTATCGATGAACAGCGGATTGACGGAGCTCTTGGCATTGCTTTCTTTTTCTTTCCTTTCCT

ATCAGCGAGTTCCGATCCTTTTGTTCGAAATTTCTTCGTTCGTACCGAACCGCTTGCAGAATCAAATCCT

GTTCCACAAGATCCTATATCAGCTATACATCCTCCTTGCATTTATGCCGGAGACGTCGCCAGTGCTATGG

GCTTTGGCTTATGTAGATCAAAAATGATGAATGGGATTGTGGCACTCCACTCGCCGCCAATGCGGAAGGA

TGTCGCCGAAAAGAATGGAACGCTGCTTTGCTCTGCTGGATGCGTCGGATCCCGTATAACAAGCGAGCTC

TTTACCCTTAAATTCAAACATGTGGGCGCCAAATGCTATCCTGCTCTATTGTTGCGTAGCAAAAGAAGCC

TGCTC---ATGCTGCTTCGGCGGCGCTTTTTCGCCTTCTCTTCGCTCTGGACAAGAGCGCTAGTGGACAC

GGGGAGGGAGCG------GGCGAAGCGTTTCT------TTCGTAATGGAAAGAAAAAGACCACTACTTTG

CCTCTTTGTTGGACCGCCGGCGCGAACACAGTGGTCTCTGACCAGGACCAGGAACCAATTCGAATTTGGA

TCTTGACATGTCGGTGCTTTTTAACCGTAGGCATCTTGCCAGGAAGTTGGTGGGCTCATCATGAATTAGG

TCGGGGTGGCTGGTGGTTTCGGGATCCCGTAGAAAATGCTTCTTTTATGCCTCGGGTATTAGCCACAGCT

CGTATTCATTCAGTCATTTTACCCCTTCTTCATTCTTGGACTTTGCTTCTTAATATTGTGACTTTTCTAT

GCTGTGTCTTAGGAACCTTTTCAATACGGTCCGGATTGCTAGCTCCCGTTCATAGTTTTGCTACAGATGA

TACACGAGGAATCTTTTTATGGCGGTTCTTCCTTCTAATGACCGGCATATCTATGATTCTTTTCTCTCAG

ATGAAGCAGCAGGCATCGGTCCGTAGAACCTATAAAAAAGAGATGGTTGTAGCGCGAAGTACTCTTGTGC

ACTTACG----TGATTGTTCGAGAATGGCTATTCTTCACAATGGCTCCTTGTGATGCAGCGGAACCATGG

CAATTAGGATTTCAAGACGCAGCAACACCTATGATGCAAGGAATAATCGACTTACATCATGATATCTTTT

TCTTCCTCATTCTTATTTTGGTTTTCGTATCATGGATCTTGGTTCGCGCTTTATGGCATTTCCACTATAA

AAAAAATCCAATCCCGCAAAGGATTGTTCATGGAACTACTATCGAGATTATTCGGACCATCTTTCCCAGT

ATCATCCTGATGTTCATTGCTATACCATCATTTGCTCTGTTATACTCAATGGACGAGGTAGTAGTAGATC

CAGCCATTACTATCAAAGCTATTGGACATCAATGGTATCGGAGT--------------------------

----------------------------------------------------------------------

----------------------------------------------------------------------

----------------------------------------------------------------------

----------------------------------------------------------------------

----------------------------------------------------------------------

-----------------------ATGAGACGACTCTTTTTTGAACTATATCATAAACAGATCTTCTTCTC

CACACCAATCACGAGTTTTTCTCCATTCCTCTCGTATATTGTCGTAACGCCCTTAATGCTAGGTTTTGAA

AAAGACTTTTCATGTCATTTCCATTTAGGTCCGATTCGGATCCCTCTGTTGTTTCCTTTTCCCCCCGCAC

CTTTTCTTCGAAATGAGAAAGAAGATGGTACACTCGAATTGTATTATTTAAGTGCTTATTGCTTGCCAAA

GATCCTACTTCTACAATTGGTAGGTCACCGGGTTATTCAAATAAGTCGTGTTTTCTGTAGTTTTCCCATG

TTACAACTTCTGTACCAATTCGGTCAATCCGGAATGGATCGGTTAAACATTCTATTAGGGAGCCTGGTCT

TGACTCTTCTGTGTGGTATTCATTCTCGTTTGGCTCTTGGAATCACATCCAGCAGTGGTTGGAACAGCTC

GCAAAATTTAACCACTTCACCTACTTCATTGCCCTCAACCGTTTCTCGTACCTCTATTGAAACAGAATGG

TTTCATGTTCTTTCATCGATTGGTTATTTTTCTTCGTTCGTCTCTCTTTTTCCAATTTCGGTCTCGATTA

GTTCACAAGATTG---------TTCTTTATTACAACCTTC-----TTTTTTGATGTCAAAGACCAGGAAC

TACGCGCAAATTCTCATTGGATCTTGGTTGTTCTTAACAGCGATGGCTATTCATTTAAGTCTTTGGGTAG

CACCACTAGATTTTCAACAAGGTGGAAATTCTCGTATTCTCTATGTACATGTTCCTGTGGCTCGGATGAG

TATTCTTGTTTATATCGTTACGGCTATAAACACTTTCTTGTTCCTATTAACAAAACATCCTCTTTTTCTT

CGCTCTTCCGGAACCGGTACAGAAATGGGTGCTTTTTCTACGTTGTTTACCTTAGTTACTGGGGGGTTTC

GGGGAAGACCCATGTGGGGGACCTTTTGGGTGTGGGATGCTCGTTTAACTTCTGTATTCATCTTGCTCCT

TATTTACTTGGGTGCGCTGTGTTTTCAAAAGCTTCCTGTAGAACCGGCTCCTATTTCAATCCGTGCTGGA

CCGATCGATATACCAATAATCAAGTTTCCAGTCAACTGGTGGAATACATCGCATCAACCTGGGAGCATTA

GCCGATCTGGTACATCAATACATGTTCCTATGCTCATTCCAATCTTGTCTAACTTTGCTAACTTCCTCTT

CTCAACCCGTCTATTATTTGTTATTGAAATACGTCTTCCTATTCCATCTTTTCTCGAATCTCCTTTAACG

GAAGAAATAGAAGCTCGAGAAGG-------GCAGGCTAGAAAGATGCTATTTGCTGCTATTCTATCTATT

TGTGCATCAAGTTCGAAGAAGATCTCAATCTATAATGAAGAAATGATAGTAGCTCGTTGTTTTATAGGCT

TTATCATATTCAGTCGGAAGAGTTTAGGTAATACTTTCAAAGTGACTCTCGACGAGAGAATCCAGGCTAT

TCAGGAAGAATCGCAGCAATTCCCCAATCCTAACGAAGTAGTTCCTCCGGAATCCAATGAACAACAACGA

TTACTTAGGGTCAGCTTGCGAATTTGTGGAACCGTAGTAGAATCATTACCAATGGCACGCTGTGCGCCTA

AGTGCGAAAAAACAGTGCAAGCTTTGTTATGTCGAAACCTAAATGTTAAGTCAGCAACACTTCCAAATGC

CACTTCTTCCCGTCGCACCCGTCTTCAGGATGATCTAGTCACAGGTTTTCACTTCTCAGTGAGTGAAAGA

TTTGTCCCCGGGTCTACGTTGAAAGCTTCTATAGTAGAACTCATTCGAGAAGGCTTGGCGGTCTTAAGAA

TGGTTCGGGTAGGAGGTTCTCTT--TGAAAGAGGCGATCAGAATGGTACCCGAATCCATTTACGATCCCG

AGTTTCCAGACACATCGCACTTCCGCTCGGGTCGAGGCTGCCATTCGGCCCTAAGACGGATTAAAGAAGA

GTGGGGAACCTCTCGCTGGTTTTTGGAATTCGACATCAGGAAGTGTTTTCACACCATCGACCGACATCGA

CTCATCCCAATCTTTAAGGAAGAGATCGACGATCCCAAGTTCTTTTACTCGATTCAGAAAGTCTTTTCTG

CCGGACGACTCGTAGGAGGTGAGAAGGGCCCTTACTCCGTCCCACACAGTGTACTACTATCGGCCCTACC

AGGCAACATCTACTTACACAAGCTCGATCAGGAGATAGGGAGGATTCGACAGAAGTACGAAATTCCGATT

GTTCAGAGAATCAGATCGGTTCTATTAAAGACAAGTCGTATTGATGACCAAGAAAACTCTGGAGAAGAAG

--ATGGAATTCTCTCCCAGAGCTGCGGAACTAACGAATCTATTAGAAAGTAGAATTACCAACTTTTACAC

GAATTTTCAAGTGGATGAGATCGGTCGAGTGGTCTCAGTTGGAGATGGGATTGCACGTGTTTATGGATTG

AACGAGATTCAAGCTGGGGAAATGGTGGAATTTGCCAGCGGTGTGAAAGGAATAGCCTTAAATCTTGAGA

ATGAGAATGTAGGGATTGTTGTCTTTGGTAGTGATACCGCTATTAAAGAGGGAGATCTTGTCAAGCGCAC

TGGATCTATTGTGGATGTTCCTGCGGGAAAGGCTATGCTAGGGCGTGTGGTCGACGCGTTGGGAGTACCT

ATTGATGGAAGAGGGGCTCTAAGCGATCACGAGCGTCGACGTGTCGAAGTGAAAGCCCCCGGGATTATTG

AACGTAAATCTGTGCACGAGCCTATGCAAACCGGGTTAAAGGCGGTAGATAGCCTGGTTCCTATAGGCCG

TGGTCAACGAGAACTTATAATCGGGGACCGACAAACGGGAAAAACAGCTATTGCTATCGATACCATATTA

AACCAAAAGCAACTGAACTCAAAGGCCACCTCTGAGAGTGAGACATTGTATTGTGTCTATGTAGCGGTTG

GACAGAAACGTTCAACTGTGGCACAATTAGTTCAAATTCTTTCAGAAGCGAATGCTTTGGAATATTCCAT

TCTTGTAGCAGCCACCGCTTCGGATCCTGCTCCTCTTCAATTTCTGGCCCCATATTCTGGGTGTGCTATG

GGAGAATATTTCCGCGATAATGGAATGCACGCATTAATAATCTATGATGATCTTAGTAAACAGGCGGTGG

CATATCGACAAATGTCATTATTGTTACGCCGACCACCAGGCCGTGAGGCTTTCCCAGGCGACGTTTTCTA

TTTACATTCCCGTCTCTTAGAAAGAGCCGCTAAACGATCGGACCAGACAGGCGCAGGTAGCTTGACCGCC

CTACCCGTCATTGAAACACAAGCTGGAGACGTATCGGCCTATATTCCCACCAATGTGATCTCCATTACTG

ATGGACAAATCTGTTTGGAAACAGAGCTCTTTTATCGCGGAATTAGACCTGCTATTAACGTCGGCTTATC

TGTCAGTCGCGTCGGGTCTGCCGCTCAGTTGAAAGCTATGAAACAAGTCTGCGGTAGTCCAAAACTGGAA

TTGGCACAATATCGCGAAGTGGCCGCCTTTGCTCAATTTGGGTCAGACCTTGATGCTGCGACTCAGGCAT

TACTCAATAGAGGTGCAAGGCTTACAGAAGTACCGAAACAACCACAATATGCACCACTTCCAATTGAAAA

ACAAATTCTAGTCATTTACGCAGCTGTCAATGGATTCTGTGATCGAATGCCACTAGATAAAATTTCTCAA

TATGAGCGAACCATTCCAAATAGTGTAAAACCAGAATTATTACAATCCCT------TAAGGGGGGGTTAA

CTAACGAAAAAAAGATGGAACTAGATTCCTTCTTAAAAGAATGCGCTTTGAATTACTA------------

--------CATCCAACGCAAAGCGGCCTTTCATTCCCTTGTTTCGTCGTGGCACACCCTCCCCGCAAGCA

CCCCCCGGCTCAGGGGGGACCAGAAAAGGCCTTTCGTTTTCCCCCCTTCGTCGGCCCTTACCGCCTTCCT

TAACAAGCCCTCGAGCCTCCTTTTCGCTGCGTTCCTCATAGAAGCCGCCGGGTTGACCCCGAAGGCCGAA

TTCTATGGTAGAGAATGCTGTAATAATAATTGGGCCATGAGAGACCTTTTTAAGTATTGCAAAAGAAAGG

GCCTGCTGATAGAGCTGGGCGAGGCAGCGATACTAGTTATCAGGTCAGAGAAAGGCCTGGCCCGTAAGCT

GGCCCCCTTTAAAACCCATTACTTAATAAGGATTTGTTACGCGCGATATGCCGACGACTTACTATTGGGA

ATCGTGGGTGCCGTAGAGCTTCTCATAGAAATACAAAAACGTATCGCCCACTTCCTACAATCCGGCCTGA

ACCTTTGGGTAGGCTCTGCGGGATCAACAACAATAGCTGCACGGAGTACGGTAGAATTCCTCGGTACGGT

CATTCGGGAAGTCCCTCCGAAGACGACTCCCATACAATTTTTGCGAGAGCTGGAGAAGCGTCTACGGGTA

AAGCACCGTATCCATATAACTGCTTGCCACTTACGCTCTGCCATTCATTCCAAGTTTAGGAACCTAGGTA

ATAGTATCCCGATCAAACAGCTGACGAAGGGGATGAGCAAAACAGGGAGTCTACTGGACGCGGTTCAACT

AGCGGAAAGTCTTTCCACAGCTAGAGTAAGAAGTCCCCAAGTGAGCGTATTATGGGAGACCGTCAAGCAC

ATCCGGCAAGGATCAAGGGAGATCTCGTTGTTGCATAGCTCAGGTCAGAGCAAGGTGCCATCGGACGTTC

AACAGGCAGTCTTGCGATCGGGCATGAGTGTCCGGAAGTTGTC---ATTGTATACTCTCGCGGGTCGGAA

GGCGGCGGGGGAAGGAGGGGGACACTGGTCGAGATCTATCAGCAGCGAATTCCCCATACAGATAGAAGCG

CCTATCAAAAAGATACTCCGAAGGCTTCGAGATCGAGGTCTCATTAGCCGAAGAAGACCCTGGCCAATCC

ACGTGGCCTGCTTGACGAACGTCAGCGACGGAGACATCGTAAATTGGTCCGCGGGCATCGCGATAAGTCC

TCTGTCCTACTACAGGTGCCGCGACAACCTTTACCAAGTCCGAACGATTGTCGACTACCAGATCCGCTGG

TCTGCAATATTCACCCCAGCCCACAAGCACAAATCCTCAGCGCGGAATATAATCCAAAAGTACTCCAAAG

ACTTAAATATAGTAAATCAAGAAGGTGGCAAGACCCTTGCGGAGTTCCCCAACAGCATAGAGCTTGGGAA

GCTCGGACCCGGTCAAGATCC-GAACAACAAAGAGCACTCAACTACTA-----------------ATGGT

CCAACTACATAACTTTTTCTTTTTCATTACTTCCATGGTCGTGCCTTGTGGCACGGCAGCACCCGTACTA

TTGAAATGGTTCGTCAGTAGAGATGTTTCCACAGGTGCCCCTTTTTCCAATGGTACTTTAATTCCTATTC

TTATCCCTTCATTCCTTCTTTTGGTTTATCTACATTCCAGGAAATTCATACGCTCTATGGACGGAGTAAA

AAGTGGAGTCTTGGTCAGAGCAAGTTGCCCTATTTTAT------TACCAGACATAATTGGGAGAAGCTCA

TCCGAAACTAGAGCTAGAAACGCCTTATTTCGTTTCGTTCCCATTCTTCATTTTCTTCTTCTCGAATTCA

A------GGGGGACTTCCCCTATTTAGAATCTTTTTGCGGTGTGCTCCGTTTACTATTCTTTCGTACTTT

CTTCTCTTTACCACGCGATAGGTCAGCGAAGCGTGAGCGGGCGCGGAGAAGAGAAGGCCAAACACTTCGG

CCTAAC------GGGAATGAGCAACGACGAAATGAAAAGAGAAAGTGCCCCGGGCGCCCCCATT------

TAGA---AAGAAGGGTCGAAGGGTTTGGGCCTGTAGCTTTCCCCGTCCCCCCTTCGTCGGGTGGTGCTTG

CATGGGGGGTGTGCTACCTGAAATCGGGCTTGAAGCTCCCGCCTTACCAACGAGCCGACAGCTGATGGCT

GTTGGTCACGACTACTACCAAAAGGTGAACATGAAGATGAATATTTCACATGGGGGAGTGTGCATCTTTA

TGTTGGGTGTTCTTCTG--CGTACATAGCTGTTCCAGCTGAAATACTTGGAATAATTCTACCACTTCTAC

TAGGAGTAGCCTTTTTAGTGCTAGCTGAACGTAAAGTAATGGCTTTTGTGCAACGTCGAAAGGGTCCTGA

TGTAGTGGGATCGTTCGGATTGTTACAACCTCTAGCAGATGGTTCGAAATTGATTCTAAAAGAACCTATT

TCACCAAGTAGTGCTAATTTCTCCCTTTTTAGAATGGCTCCAGTCACTACATTTATGCTAAGTCTGGTTG

CTCGGGCCGTTGTACCTTTTGATTATGGTATGGTATTGTCAGATCCGAACATAGGGCTACTTTATTTGTT

TGCCATATCTTCGCTAGGTGTTTATGGAATTATTATAGCAGGTTGGTCTAGTA-TTATTATATACGTTTA

GTGAAAAGAATGTTTTTTGATACACCTAGGACATGGATTCTATATGAACCAATGGATCGTGACAAGTCGT

TACTACTAGCAATGACTTCCTCTTTCATTACTTCATCCTTTCCATATCCTTCTCCCTTGTTCTCAGTTAC

TCATCAAATGGCACTCAGTTTATATCTTTAA-TGTCAGAATTTGCGCCTATTTGTATCTATTTAGTGATC

AGTCTGCTAGTTTCTTTGATCCCACTCGGTGTTCCTTTTCCATTTTCTTCTAATACTTCGACTTATCCAG

AAAAATTGTCGGCCTACGAATGTGGTTTCGATCCTTTCGGTGATGCCAGAAGTCGTTTCGATATACGATT

TTATCTTGTTTCAATTTTATTTATTATCCTTGATCCGGAAGTAACCTTTTTCTTTCCTTGGGCAGTACCT

CTCAACAAGATTGATCCGTTTGGATCTTGGTCCATGATGGCCTTTTTATTGATTTTAACGATAGGATTTC

TCTATGAATGGAAAAGGGGTGCTTCGGATCGGGAGTAAAAAAGTGTTTATTACGATTACGCCCAACAGCC

CACTTGAGCAATTTTCCATTCTCCCATTGATTCCTATGAAAATAGGAAACTTGTATTTCTCATTCACAAA

TCCATCTTTGTTTATGCTGCTAACTCTCAGTTTGGTCCTACTTCTGCTTCATTTTGTTACTAAAAAGGGA

GGAGGAAACTCAGTACCAAATGTTTGGCAATCCTTGGTAGAGCTTATTTATGATTTCGTGCTGAACCTGG

TAAACGAACAAATAGGTGGTCTTTCCGGAAATGTTAAACAAAAGTTTTTCCCTTGCATCTTGGTCACTTT

TACTTTTTTGTTATTTCGTAATCTCCAGGGTATGATACCCTATAGCTTTACAGTTACAAGTCATTTTCTC

ATTACTTTGGGTCTTTCATTTTCCATTTTTATTGGCATTACTATAGTGGGATTTCAAAGAAATGGGCTTC

ATTTTTTAAGCTTCTCATTACCTGCAGGAGTCCCGCTGCCGTTAGCACCTTTTTTAGTACTCCTTGAGCT

AATCCCTCATTGTTTTCGCGCATTAAGCTCAGGAATACGTTTATTTGCTAATATGATGGCCGGTCATAGT

TCAGTAAAGATTTTAAGTGGGTTCGCTTGGACTATGCTATGTATGAATGATCTTTTATATTTCATAGGAG

ATCTTGGTCCTTTATTTATAGTTCTTGCATTAACCGGTCTTGAATTAGGTGTAGCTATATTACAAGCTCA

TGTTTTTACGATCTTAATCTGTATTTACTTGAATGATGCTACAAATCTCCATCAA---------------

-----------------TATGTGGGCACCTGATATCTATGAGGGTTCACCCACCCCGGTTACAGCATTCT

TTTCTATTGCGCCTAAAATATCTATTTCTGCTAATATTTTACGTGTTTTTATTTATGGTTCCTATGGAGC

TACATTGCAACAAATCTTCTTTTTCTGCAGCATTGCTTCTATGATCTTAGGAGCACTGGCCGCCATGGCC

CAAACGAAAGTAAAAAGACTTCTAGCTCATAGTTCAATTGGACATGTAGGTTATATTCGTACTGGTTTCT

CATGTGGAACCATAGAAGGAATTCAATCACTACTAATTGGTCTCTTTATTTATGCATCAATGACGATAGA

TGCATTCGCTATAGTTTCAGCATTACGGCAAACACGTGTCAAATATATAGCGGATTTGGGCGCTCTAGCC

AAAACGAATCCTATTTCGGCTATTACCTTCTCTATTACTATGTTCTCATACGCAGGAATACCCCCGTTAG

CCGGCTTTTGTAGTAAATTCTATTTGTTCTTCGCCGCTTTGGGTTGTGGGGCTTACTTCCTAGCCCCAGT

GGGAGTAGTGACTAGCGTTATAGGTTGTT-GTTCGATAGCCCGACCGTAGTGATGTTAATTGTGGTTACA

TTCATAAGTAGCTTGGTCCATCTTTATTCTATTTCATATATGTCTGAGGATCCGCATAGCCCTCGATTTA

TGTGTTATTTATCCATTCTTACTTTTTTTATGCCAATGTTGGTGACTGGAGATAACTCTCTTCAATTATT

CTTGGGATGGGAGGGAGTAGGTCTTGCTTCATATTTGTTAATTCATTTTTGGTTTACACGACTTCAGGCA

GATAAAGCAGCTATAAAAGCTATGCTTGTCAATCGAGTAGGTGATTTTGGATTAGCTCTTGGGATTTCGG

GTTGTTTTACTCTCTTTCAAACAGTAGACTTTTCTACCATTTTTGCTTGTGCTAGTGCCCCTAGAAATTC

TTGGATTTTTTGCAATATGAGATTGAATGCCATAACTCTTATTTGTATTTTACTTTTTATTGGTGCTGTT

GGAAAATCTGCACAGATAGGATCGCATACTTGGTCACCCGATGCTATGGAGGGTCCCACTCCAGTATCCG

CTTTGATTCATGCAGCTACTATGGTAACAGCTGGCGTTTTCATGATAGCAAGGTGTTCCCCTTTATTTGA

ATACCCACCTACGGCTTTAATTGTTATTACTTTTGCAGGAGCTATGACGTCATTCCTTGCGGCAACCACT

GGAATATTACAGAACGATCTAAAGAGGGTCATAGCTTATTCAACTTGCAGTCAATTAGGCTATATGATCT

TTGCTTGCGGCATTTCTAACTATTCGGTTAGCGTCTTTCATTTAATGAATCACGCCTTTTTCAAAGCATT

ACTATTCCTGAGTGCAGGTTCGGTGATTCATGCCATGTCGGATGAGCAAGATATGCGGAAGATGGGGGGG

CTCGCCTCCTCGTTCCCCTTTACCTATGCCATGATGCTCATGGGCAGCTTATCTCTAATTGGATTTCCTT

TTCTAACTGGATTTTATTCCAAAGATGTGATCTTAGAGCTCGCTTACACTAAGTATACCATCAGTGGGAA

CTTTGCTTTCTGGTTGGGAAGTGTCTCTGTCCTTTTCACTTCTTATTACTCTTTTCGTTTACTTTTTCTA

ACATTTCTAGTACCAACTAATTCATTCGGGCGAGACATCTTACGATGTCATGATGCGCCCATTCCTATGG

CCATTCCTTTAATACTTCTGGCTTTCGGGAGTCTCTTTGTAGGATACTTGGCCAAACTAACACAAAGAAG

ATACAGTTCACTCAACGATTGCCTTTGGGTTCCGAACTCCATATGGGGAAGGAACGTTGTTGTTTGCGGG

GTCTCGATCATTTACATGGACCCACTTTTCATTCCATTTGTGGGAATTTGATGATCTATAAACCGTCCTT

AACGAACGATCGGCTCATCTT------TGAGCATGATGAATCACTTCGTGCCGACCTGTTGTCAATAAAC

TTTTGGGCCTCATATGAGAATGGAAAACTGGAGCATTTTCTGCATCGGTGGATGAAGAATCGCGAACATC

CAAATTTCTGGTTAAGCATGTTCCCAGAAAAAAGATACTTTCGAGAAACAACGAGCACGACTGAAGTGGC

TATCCATACAAATCCATTTACGGATCTATATGCTTCGATTGGAACTGGAAGTTCAAGAACAGGCGGCTGG

TATACTACCATAATGAAACTGCCTTTTCTTTTTTTTATTCGGATAGGATTTCTGTTGGCTTCGCTGGGAG

GCTCGCGTAGTTTGTTACGTCAACTCCAAAAGGAGAAATTGCGTTGGAATTGAGAAAGTTACGTAA----

AGTTCATAATTGTATA-CCAATTTTTGGGCCAATTCCCTCTTCGTACTACCAAAAAATGAGATTCTTGCC

GAATCCGAGTTTGCTGCTCCAACCATTACCAAACTAATACCTATTCTGTTTAGTACTTCAGGTGCTTCTG

TTGCGTATAATGTAAATCCCGTAGCGGATCAATTCC---------------AACGAGCCTTTCAAACTAG

TACTTTTTGTAATCGACTCTATAGCTTCTTCAATAAACGCTGGTTCTTCGATCAAGTTTTGAATGACTTT

ATAGTCAGATCGTTCTTGCGTTTCGGGTATGAAGTCTCATTCGAAGCTTTAGACAAAGGTGCTATTGAGA

TATTGGGCCCCTATGGTATCTCGTACACATTCCGACGATTGGCCGAGCGAATAAGTCAACTTCAAAGTGG

ATTTGT--TGTTCCATGATCTATGGGTCTACAGGAGCTACCCATTTCGATCAATTAGCCAAGATTTTGAC

CGGATACGAAATCACTGGTGTTCGATCTAGTGGTATTTTTATGGGGATTCTTTTTATCGCTGTAGGATCC

CTATTCAAGATCACTGCAGTTCCTTTTCTGGTCTATGCACATCGCTTTCTCCAGGAGGTTGGCCGCCTAT

CCTAGATCTTCCCATTTCCAAGAGGATCCCGGGCTCGATCTGGTTTAGTATCAAGGTGATTCTCTTTCTC

TTTCTATATATATGGGTCCGTGCAGCATTTCCACGATATCGTTATGATCAATTAATGGGACTTGGCCGGA

AAGTGTTCTTGCCTCTATCATTAGCTCGGGTAGTCGCCGTTTCTGGTGTTTTAGTCACCTTTCAATGGCT

CCCTTA-ATGCCTCAACTGGATCAATTTACTTATTTCACACAATTCTTCTGGTCATGCCTTTTCTTCTTG

ACTTTCTATATTCTAATATGCAATGATAGAGATGGAGTACTTGGGATCAGCAGAATTCTAAAACTACGAA

ATCAACTGCTTTCACACCGGGGGAACAACATCCAAA------GCAAGGACCCAAACAGTTTGCAAGATAT

CTTGAGAAAGGGTTTTAACACAGGTGTATCCTATATGTACTCTAGTTTATTCGAAGTATCCCAATGGTGT

AAGGCCGTCGACTTATTTGGAAAAAGGAAGAAAATCACTTTGATCTCTTGTTTCGGAGAAATAAGTGGCT

CACGAGGAATGGAAAGAAACATATTCTATTTGATCTCGAAGTCTTCATATAGCACTTC------------

---TTCCAATCCTGGATGGGTGATCACTTGTAAGAATGACATAATGCTAATCCATGTTCTACACGGCCAA

GAA--------------------

>Haloxylon ammodendron NCxxx

-TGATACTTTCTGTTTTGTCGAGCCCGGCTTTGGTCTCTGGTTTGATGGTTGTACGTGCTAAAAATCCGG

TACATTCCGTTTTGTTTCCCATCCCAGTCTTTCGCAACACTTCAGGTTTACTTCTTTTGTTAGGTCTCGA

TTTTTCCGCTATGATCTTCCCAGTAGTTTATATAGGAGCTATAGCCGTTTCATTCCTATTCGTTGTTATG

ATGTTCCATATTCAAATAGCGGAGATTCACGAAGAAGTATTGCGCTATTTACCAGTGAGTGGTATTATTG

GACTGATCTTTTGGTGGGAAATGTTCTTCATTTTAGATAATGAAACCATTCCATTACTACCAACCCAAAG

AAATACGACCTCTCTGAGATATACGGTTTATGCCGGAAAGGTACGAAGTTGGACTAATTTGGAAACATTG

GGCAATTTACTTTATACTTACTATTTTGTCTGGTTTTTGGTTTCTAGTCTTATTTTATTAGTAGCCATGA

TTGGGGCTATAGTACTGACTATGCATAGGACTACTAA------GGTGAAAAGACAGGATGTATTCCGACG

AAATGCTATTGATTCTAGAAGGACTATAATGAAGAGGACGACAGACC-ATGTCAATATATGAATTGTTTC

ATTATTCGTTATTTCCGGGTCTTTTCATTGCATTCACTTACAACAAAAAACAACCACCGGCGTTTGGTGC

AGCACCTGCATTTTGGTGTATTCTTCTTTCTTTCCTTGGTCTTTTGTTCTGTCATATTCCTAATAACTTA

TCCAATTACAACGTATTAACCGCTAATGCACCTTTCTTTTATCAAATCTCAGGGACATGGTCTAATCATG

AAGGTAGTATTTTATTATGGTGTCGGATCCTAAGTTTTTATGGATTCCTTCTTTGTTACCGGGGTCGATC

CCAAAGCCATAATGTCTCAAAACGAGGAGGCCATAGAGAAAGTCTTCTTTTTTCCTTTGTCTTAAACTTC

GTGAAGAACTCCATTCTATCTCTTCCTCGTTACGAACAAAAAAGTAGAGTTCTTCACGAACCCCAGTTGT

ACACTTTCTTTGTTCTACGAA---CTCTTGTTGATTCTGAACTTTGTTCGCGAAAGAACCGGACTTTTGA

CGGGCCAGCTCTTTTTTACGCGCCGCTTTACCCTGAAAGGAAAATGAGCTTTGCTCTTCTGGGCGCTAGG

CGCTCTCGTGGTTCGCGAGAAGGAAAAAGGACTCATCCTTTGTTGCATCTGGCACGAGATGATAAAGAGA

GAGCTTCGTCTATCGATGAACAGCGGATTGACGGAGCTCTTGGCATTGCTTTCTTTTTCTTTCCTTTCCT

ATCAGCGAGTTCCGATCCTTTTGTTCGAAATTTCTTCGTTCGTACCGAACCGCTTGCAGAATCAAATCCT

GTTCCACAAGATCCTATATCAGCTATACATCCTCCTTGCATTTATGCCGGAGACGTCGCCAGTGCTATGG

GCTTTGGCTTATGTAGATCAAAAATGATGAATGGGATTGTGGCACTCCACTCGCCGCCAATGCGGAAGGA

TGTCGCCGAAAAGAATGGAACGCTGCTTTGCTCTGCTGGATGCGTCGGATCCCGTATAACAAGCGAGCTC

TTTACCCTTAAATTCAAACATGTGGGCGCCAAATGCTATCCTGCTCTATTGTTGCGTAGCAAAAGAAGCC

TGCTC---ATGCTGCTTCGGCGGCGCTTTTTCGCCTTCTCTTCGCTCTGGACAAGAGCGCTAGTGGACAC

GGGGAGGGAGCG------GGCGAAGCGTTTCT------TTCGTAATGGAAAGAAAAAGACCACTACTTTG

CCTCTTTGTTGGACCGCCGGCGCGAACACAGTGGTCTCTGACCAGGACCAGGAACCAATTCGAATTTGGA

TCTTGACATGTTGGTGCTTTTTAACCGTAGGCATCTTGCCAGGAAGTTGGTGGGCTCATCATGAATTAGG

TCGGGGTGGCTGGTGGTTTTGGGATCCCGTAGAAAATGCTTCTTTTATGCCTCGGGTATTAGCCACAGCT

TGTATTCATTCAGTAATTTTACCCCTTCTTCATTCTTGGACTTTGCTTCTTAATATTGTGACTTTTTTAT

GCTGTGTCTTAGGAACCTTTTCAATACGGTCCGGATTGCTAGCTCCCGTTCATAGTTTTGCTACAGATGA

TACACGAGGAATCTTTTTATGGCGGTTCTTCCTTCTAATGACCGGCATATCTATGATTCTTTTCTCTCAG

ATGAAGCAGCAGGCATCGGCCCGTAGAACCTATAAAAAAGAGATGGTTGTAGCGCG--------------

----------ATGATTGTTCGAGAATGGCTATTCTTCACAATTGCTCCTTGTGATGCAGCGGAACCATGG

CAATTAGGATTTCAAGACGCAGCAACACCTATGATGCAAGGAATAATAGACTTACATCATGATATCTTTT

TCTTCCTCATTCTTATTTTGGTTTTCGTATCATGGATCTTGGTTCGCGCTTTATGGCATTTCCACTATAA

AAAAAATCCAATCCCGCAAAGGATTGTTCATGGAACTACTATCGAGATTATTCGGACCATTTTTCCCAGT

ATCATCCTGATGTTCATTGCTATACCATCATTTGCTCTGTTATACTCAATGGACGAGGTAGTAGTAGATC

CAGCCATTACTATCAAAGCTATTGGACATCAATGGTATCGGAG---------------------------

----------------------------------------------------------------------

----------------------------------------------------------------------

----------------------------------------------------------------------

----------------------------------------------------------------------

----------------------------------------------------------------------

-----------------------ATGAGACGACTCTTTTTTGAACTATATCATAAACAGATCTTCTTCTC

CACACCAATCACGAGTTTTTCTCTATTCCTCTCGTATATTGTCGTAACGCCCTTAATGCTAGGTTTTGAA

AAAGACTTTTCATGTCATTTCCATTTAGGTCCGATTCGGATCCCTCTGTTGTTTCCTTTTCCTTCCGCAC

CTTTTCTTCGAAATGAGAAAGAAGATGGTACACTCGAATTGTATTATTTAAGTGCTTATTGCTTGCCAAA

GATCCTACTTCTACAATTGGTAGGTCACCGGGTTATTCAAATAAGTCGTGTTTTCTGTAGTTTTCCCATG

TTACAACTTCTGTACCAATTCGGTCAATCCGGAATGGATCGGTTAAACATTCTATTAGGGAGCCTGGTCT

TGACTCTTCTGTGCGGTATTCATTCTTGTTTGGCTCTTGGAATCACATCCAGCAGTGGTTGGAACAGCTC

GCAAAATTTAACCACTTCACCTACTTCATTGCCCTCAACCGTTTCTCGTACCTCTATTGAAACAGAATGG

TTTCATGTTCTTTCATCGATTGGTTATTTTTCTTCGTTCGTATCTCTTTTTCCAATTTCGGTCTCGATTA

GTTCACAAGATTG-ATGTCCGTTTTGTTATTACAACCTTA-----TTTTTTGATGTCAAAGACCAGAAGC

TACGCGCAAATTCTCATTGGATCTTGGTTGTTCTTAACAGCGATGGCTATTCATTTAAGTCTTTGGGTAG

CACCATTAGATTTTCAACAAGGTGGAAATTCTCGTATTCTCTATGTACATGTTCCTGTGGCTCGGATGAG

TATTCTTGTTTATCTCGTTACGGCTATAAACACTTTCTTGTTCCTATTAACAAAACATCCTCTTTTTCTT

CGCTCTTCCGGAACCGGTACAGAAATGGGTGCTTTTTCTACGTTGTTTACCTTAGTTACTGGGGGGTTTC

GGGGAAGACCCATGTGGGGCACCTTTTGGGTGTGGGATGCTCGTTTAACTTCTGTATTCATCTCGTTCCT

TATTTACTTGGGTGCGCTGTGTTTTCAAAAGCTTCCTGTAGAACCGGCTCCTATTTCAATCCGTGCTGGA

CCGATCGATATACCAATAATCAAGTTTCCAGTCAACTGGTGGAATACATCGCATCAACCTGGGAGCATTA

GCCGATCTGGTACATCAATACATGTTCCTATGCTCATTCCAATCTTGTCTAACTTTGCTAACTTCCTCTT

CTCAACCCGTATCTTCTTTGTTCTGGAAACACGTCTTCCTATTCCATCTTTTCTCGAATCTCCTTTAACG

GAAGAAATAGAAGCTCGAGAAGGAATA--TGCAGGCTAGAAAGATGCTATTTGCTGCTATTCTATCTATT

TGTGCATCAAGTTCGAAGAAGATCTTAATCTATAATGAAGAAATGATAGTAGCTCGTTGTTTTATAGGCT

TTATCATATTCAGTCGAAAGAGTTTAGGTAATACTTTCAAAGCGACTCTCGACGAGAGAATCCAGGCTAT

TCAGGAAGAATCGCAGCAATTCCCCAATCCTAACGAAGTAGTTCCTCTGGAATCCAATGAACAACAACGA

TTACTTAGGGTCAGCTTGCGAATTTGTGGAACCGTAGCGGAATCATTACCAATGGCACGCTGTGCGCCTA

AGTGCGAAAAGACAGTGCAAGCTTTGTTATGTCGAAACCTAAATGTTAAGTCAGCAACACTTCCAAATGC

CACTTCTTCCCGTCGCACCCGTCTTCAGGACGATCTAGTCACAGGGTTTCACTTCTCAGTGAGTGAAAGA

TTTGTCCCCGGGTCTACGTTGAAAGCTTCTATAGTAGAACTCATTCGAGAAGGCTTGGCGGTCTTAAGAA

TGGTTCGGGTAGGGGGTTCTCT--ATGAAAGAGGCGATCAGAATGGTACCCGAATCCATTTACGATCCCG

AGTTTCCAGACACATCGCACTTCCGCTCGGGTCGAGGCTGCCATTCGGTCCTAAGACGGATCAAAGAAGA

GTGGGGAACCTCTCGCTGGTTTTTGGAATTCGACATCAGGAAGTGTTTTCACACCATCGACCGACATCGA

CTCATCCCAATCTTTAAGGAAGAGATCGACGATCCCAAGTTCTTTTACTCCATTCAGAAAGTCTTTTCTG

CCGGACGACTCGTAGGAGGTGAGAAGGGCCCTTACCCCGTCCCACACAGTGTACTACTATCGGCCCTACT

AGGCAACATCTACTTACACAAGCTCGATCAGGAGATAGGGAGGATCCGACAGAAGTACGAAATTCCGATT

GTTCAGAGAATCAGATCGGTTCTATTAAAGACAAGTCGTATTGATGACCAAGAAAACTCTGGAGAAGAA-

---TGGAATTCTCTGCCAGAGCTGCGGAACTAACGACTCTATTAGAAAGTAGAATTACCAACTTTTACAC

CAATTTTCAAGTGGATGAGATCGGTCGAGTGGTCTCAGTTGGAGATGGGATTGCACGTGTTTATGGATTG

AACGAGATTCAAGCTGGGGAAATGGTGGAATTTGCCAGCGGTGTGAAAGGAATAGCCTTAAATCTTGAGA

ATGAGAATGTAGGGATTGTTGTCTTTGGTAGTGATACCGCTATTAAAGAGGGAGATCTTGTCAAGCGCAC

TGGATCTATTGTGGATGTTCCTGCGGGAAAGGCTATGCTAGGGCGTGTGGTCGACGCGTTGGGAGTACCT

ATTGATGGAAGAGGGGCTCTAAGCGATCACGAGCGTCGACGTGTCGAAGTGAAAGCCCCCGGGATTATTG

AACGTAAATCTGTGCACGAACCTATGCAAACCGGGTTAAAGGCGGTAGATAGCCTGGTTCCTATAGGCCG

TGGTCAACGAGAACTTATAATCGGGGACCGACAAACGGGAAAAACAGCTATTGCTATCGATACCATATTA

AACCAAAAGCAACTGAACTCAAAGGCCACCTCTGAGAGTGAGACATTGTATTGTGTCTATGTAGCGGTTG

GACAGAAACGTTCAACTGTGGCACAATTAGTTCAAATTCTTTCAGAAGCGAATGCTTTGGAATATTCCAT

TCTTGTAGCAGCCACCGCTTCGGATCCTGCTCCTCTTCAATTTCTGGCCCCATATTCTGGGTGTGCTATG

GGAGAATATTTCCGCGATAATGCAATGCACGCATTAATAATCTATGATGATCTTAGTAAACAGGCGGTGG

CATATCGACAAATGTCATTATTGTTACGCCGACCACCAGGCCGTGAGGCTTTCCCAGGCGACGTTTTCTA

TTTACATTCCCGTCTCTTAGAAAGAGCCGCTAAACGATCGGACCAGACAGGTGCCGGTAGCTTGACCGCC

TTACCCGTCATTGAAACACAAGCTGGAGACGTATCGGCCTATATTCCCACCAATGTGATCTCCATTACTG

ATGGACAAATCTGTTTGGAAACAGAGCTCTTTTATCGCGGAATTAGACCTGCTATTAACGTCGGCTTATC

TGTCAGTCGCGTCGGGTCTGCCGCTCAGTTGAAAGCTATGAAACAAGTCTGCGGTAGTCCAAAACTGGAA

TTGGCACAATATCGCGAAGTGGCCGCCTTTGCTCAATTTGGGTCAGACCTTGATGCTGCGACTCAGGCAT

TACTCAATAGAGGTGCAAGGCTTACAGAAGTACCGAAACAACCACAATATGCACCACTTCCAATTGAAAA

ACAAATTCTAGTCATTTACGCAGCTGTCAATGGATTCTGTGATCGAATGCCACTAGATAAAATTTCTCAA

TATGAGAGAACCATTCCAAATAGTGTAAAACCAGAATTATTACAATCCCT------AAAAGGGGGGTTAA

CCAACGAAAAAAAGATGGAACTAGATGCTTTCTTAAAAGAATGCGC------------------------

-------GCATCCAACGCAAAGCGGCCTTTCATTCCCTTGTTTCGTCGTGGCACACCCTCCCCGCAAGCA

CCCCCCGGCTCAGGGGGGACCAGAAAAGGCCTTTCGTTTTCCCCTCTTCGTCGGCCCTTGCCGCCTTCCT

TAACAAGCCCTCGAGCCTCCTTTTCGCTGCGTTCCTCATAGAAGCCGCCGGGTTGACCCCGAAGGCCGAA

TTCTATGGTAGAGAGTGTTGTAATAATAATTGGGACATGAGCGACCTTTTTAAGTATTGCAAAAGAAAGG

GCCCCCTGATAGAGCTGGGCGAGGCAGCGATACTAGTTATCAGGTCAGAGAAAGGCCTGGCCCGTAAGCT

GGCCCCTTTAAAACCCCATTACTTAATAAGGATTTGTTACGCGCGATATGCCGACGACTTACTATTGGGA

ATCGTGGGTGCCGTAGAGCTTCTCATAGAAATACAAAAACGTATCGCCCACTTCCTACAATCCGGCCTGA

ACCTTTGGGTAGGCTCTGCGGGATCAACAACAATAGCTGCACGGAGTACGGTAGAATTCCTCGGTACGGT

CATTCGGGAAGTCCCTCCGAAGACGACTCCCATACAATTCTTGCGAGAGCTGGAGAAGCGTCTACGGGTA

AAGCACCGTATCCATATAACTGCTTGCCACTTACGCTCTGCCATTCATTCCAAGTTTAGGAACCTAGGTA

ATAGTATCCCGATCAAACAGCTGACGAAGGGGATGAGCAAAACAGGGAGTCTACTGGACGCCGTTCAACT

AGCGGAAAGTCTTTCCACAGCTAGAGTAAGAAGTCCCCAAGTGAGCGTATTATGGGAGACCGTCAAGCAC

ATCCGGCAAGGATCAAGGGAGATCTCGTTGTTGCATAGCTCAGGTCAGAGCAAGGTGCCATCGGACGTTC

AACAGGCAGTCTCGCGATCGGGCATTCATGCCCGGAAGTTGTC---ATTGTATACTCTCGCGGGTCGGAA

GGCGGCGGGGGAAGGAGGGGGACACTGGTCGAGATCTATCAGCAGCGAATTCCCCATACAGATAGAAGCG

CCTATCAAAAAGATACTCCGAAGGCTTCGAGATCGAGGTCTCATTAGCCAAAAAAGACCCTGGCCAATCC

ACGTGGCCTGCTTGACGAACGTCAGCGACGGAGACATCGTAAATTGGTCCGCGGGCATCGCGATAAGTCT

TCTGTCCTACTACAGGTGCCGCGACAACCTTTACCAAGTCCGAACGATTGTCGACTACCAGATCCGCTGG

TCTGCAATATTCACCCCAGCCCACAAGCACAAATCCTCAGCGCGAAATATAATCCCAAAGTACTCCAAAG

ACTTAAATATAGTAAATCAAGAAGGTGGCAAGACCCTTGCAGAGTTCCCCAACAGCATAGAGCTTGGGAA

GCTCGGACCCGGGCAAGATCC-GAACAACAAAGAGCACTCAACTACT------------------ATGGT

CCAACTACATAACTTTTTCTTTTTCATTACTTCCGTGGTCGTGCCTTGTGGCACGGCAGCACCCGTACTA

TTGAAATGGTTCGTCAGTAGAGATGTTCCCACAGGTGCCCCTTTTTCCAATGGTACTTTAATTCCTATTC

TTATCCCTTCATTCCTTCTTTTGGTTTATTTACATTCCAGGAAATTCATACGCTCTATGGACGGGGTCAA

AAGTGGAGTCTTGGTCAGAGCAAGTTGCCCTATTTTAT------TACCAGACATAATTGGGAGAAGCTCA

TCCGAAACTAGAGCTAGAAACGCCTTATTTCGTTTCGTTCCCATTCTTCATTTTCTTCTTCTCGAATTCA

A------GGGGGACTTCCCCTATTTAGAATCTTTTTGCGGTGTGCTCCGTTTACTATTCTTTCGTACTTT

CTTCTCTTTACCACGCGATAGGTCAGCGAAGCGTGAGCGGGCGCGGAGAAGAAAAGGCCAAACACTTCGG

CCTAAC------GGGAATGAGCAACGACGAAATGAAAAGAGAAAGTGCCCCGGGCGCCCCCATT------

TAGA---AAGAAGGGTCGAAGGGTTTGGGCCTGTAGCTTTCCCCGTCCCCCCTTCGTCGGGGGGTGCTTG

CATGGGGGGTGTGCTACCTGAAATCGGGCTTGAAGCTCCCGCCTTACCAACGAGCCGACAGCTGATGGCT

GTTGGTCGCGACTACTACCAAAAAGTGAACATGAAGATGAATATTTCACATGGGGGAGTGTGCATCTTTA

TGTTGGGTGTTCTTCTG-ACGTACATAGCTGTTCCAGCTGAAATACTTGGAATAATTCTACCACTTCTAC

TAGGAGTAGCCTTTTTAGTGCTAGCTGAACGTAAAGTAATGGCTTTTGTGCAACGTCGAAAGGGTCCTGA

TGTAGTGGGATCGTTCGGATTGTTACAACCTCTAGCAGATGGTTCGAAATTGATTCTAAAAGAACCTATT

TCACCAAGTAGTGCTAATTTCTCCCTTTTTAGAATGGCTCCAGTCACTACATTTATGCTAAGTCTGGTTG

CTCGGGCCGTTGTACCTTTTGATTATGGTATGGTATTGTCAGATCCGAACATAGGGCTACTTTATTTGTT

TGCCATATCTTCGCTAGGTGTTTATGGAATTATTATAGCAGGTTGGTCTAGT-TTTATTATATACGTTTA

GTGAAAAGAATGTTTTTTGATACACCTAGGACATGGATTCTATATGAACCAATGGATCGTGACAAGTCGT

TACTACTAGCAATGACTTCCTCTTTCATTACTTCATCCTTTCCATATCCTTCTCCCTTGTTCTCAGTTAC

TCATCAAATGGCACTCAGTTTATATCTTTA-ATGTCAGAATTTGCGCCTATTTGTATCTATTTAGTAATC

AGTCTGCTAGTTTCTTTGATCCCACTCGGTGTTCCTTTTCCATTTTCTTCTAATACTTCGACTTATCCAG

AAAAATTGTCGGCCTACGAATGTGGTTTCGATCCTTTCGGTGATGCCAGAAGTCGTTTCGATATACGATT

TTATCTTGTTTCAATTTTATTTATTATCCTTGATCCGGAAGTAACCTTTTTCTTTCCTTGGGCAGTACCT

CTCAACAAGATTGATCTGTTTGGATCTTGGTCCATGATGGCCTTTTTATTGATTTTAACGATAGGATTTC

TCTATGAATGGAAAAGGGGTGCTTCGGATCGGGAGTA--AAAGTGTTTATTACGATTACGCCCAACAGCC

CACTTGAGCAATTTTCCATTCTCCCATTGATTCCTATGAAAATCGGAAACTTGTATTTCTCATTCACAAA

TCCATCTTTGTTTATGCTGCTAACTCTCAGTTTGGTCCTACTTCTGCTTCATTTTGTTACTAAAAAGGGA

GGAGGAAACTCAGTACCAAATGTTTGGCAATCCTTGGTAGAGCTTATTTATGATTTCGTGCTGAACCTGG

TAAACGAACAAATAGGTGGTCTTTCCGGAAATGTTAAACAAAAGTTTTTCCCTTGCATCTTGGTCACTTT

TATTTTTTTGTTATTTCGTAATCTCCAGGGTATGATACCCTATAGCTTTACAGTTACAAGTCATTTTCTC

ATTACTTTGGGTCTTTCATTTTCCATTTTTATTGGCATTACTATAGTGGGATTTCAAAGAAATGGGCTTC

ATTTTTTAAGCTTCTCATTACCTGCAGGAGTCCCACTGCCGTTAGCACCTTTTTTAGTACTCCTTGAGCT

AATCCCTCATTGTTTTCGCGCATTAAGCTCAGGAATACGTTTATTTGCTAATATGATGGCCGGTCATAGT

TCAGTAAAGATTTTAAGTGGGTTCGCTTGGACTATGCTATGTATGAATGATCTTTTATATTTCATAGGAG

ATCTTGGTCCTTTATTTATAGTTCTTGCATTAACCGGTCTTGAATTAGGTGTAGCTATATTACAAGCTCA

TGTTTTTACGATTTTAATCTGTATTTACTTGAATGATGCTATAAATCTCCATCAAA--------------

----------------ATATGTGGGCACCTGATATCTATGAGGGTTCACCCACCCCGGTTACAGCATTCT

TTTCTATTGCGCCTAAAATTTCTATTTCTGCTAATATTTTACGTGTTTTTATTTATGGTTCCTATGGAGC

TACATTGCAACAAATCTTCTTTTTCTGCAGCATTGCTTCTATGATCTTAGGAGCACTGGCCGCCATGGCC

CAAACGAAAGTAAAAAGACTTCTAGCTCATAGTTCAATTGGACATGTAGGTTATATTCGTACTGGTTTCT

CATGTGGAACCATAGAAGGAATTCAATCACTACTAATTGGTCTCTTTATTTATGCATCAATGACGATAGA

TGCATTCGCTATAGTTTCAGCATTACGGCAAACACGTGTCAAATATATAGCGGATTTGGGCGCTCTAGCC

AAAACGAATCCTATTTCGGCTATTACCTTCTCTATTACTATGTTCTCATACGCAGGAATACCCCCGTTAG

CCGGCTTTTGTAGTAAATTCTATTTGTTCTTCGCCGCTTTGGGTTGTGGGGCTTACTTCCTAGCCCCAGT

GGGAGTAGTGACTAGCGTTATAGGTTGT--GTTCGATAGCCCGACCGTAGTGATGTTAATTGTGGTTACA

TTCATAAGTAGCTTGGTCCATCTTTATTCTATTTCATATATGTCTGAGGATCCGCATAGCCCTCGATTTA

TGTGTTATTTATCCATTCTTACTTTTTTTATGCCAATGTTGGTGACTGGAGATAACTCTCTTCAATTATT

CTTGGGATGGGAGGGAGTAGGTCTTGCTTCATATTTGTTAATTCATTTTTGGTTTACACGACTTCAGGCA

GATAAAGCAGCTATAAAAGCTATGCTTGTCAATCGAGTAGGTGATTTTGGATTAGCTCTTGGGATTTCGG

GTTGTTTTACTCTCTTTCAAACAGTAGACTTTTCTACCATTTTTGCTTGTGCTAGTGCCCCTAGAAATTC

TTGGATTTTTTGCAATATGAGATTGAATGCCATAACTCTTATTTGTATTTTACTTTTTATTGGTGCTGTT

GGAAAATCCGCACAGATAGGATCGCATACTTGGTCACCCGATGCTATGGAGGGTCCCACTCCAGTATCCG

CTTTGATTCATGCGGCTACTATGGTAACAGCTGGCGTTTTCATGATAGCAAGGTGTTCCCCTTTATTTGA

ATACCCACCTACGGCTTTAATTGTTATTACTTTTGCAGGAGCTATGACGTCATTCCTTGCGGCAACCACT

GGAATATTACAGAACGATCTAAAGAGGGTCATAGCTTATTCAACTTGCAGTCAATTAGGCTATATGATCT

TTGCTTGCGGCATTTCTAACTATTCGGTTAGCGTCTTTCATTTAATGAATCACGCCTTTTTCAAAGCATT

ACTATTCCTGAGTGCAGGTTCGGTGATTCATGCCATGTCGGATGAGCAAGATATGCGGAAGATGGGGGGG

CTCGCCTCCTCGTTCCCTTTTACCTATGCCATGATGCTCATGGGCAGCTTATCTCTAATTGGATTTCCTT

TTCTAACTGGATTTTATTCCAAAGATGTGATCTTAGAGCTCGCTTACACTAAGTATACCATCAGTGGGAA

CTTTGCTTTCTGGTTGGGAAGTGTCTCTGTCCTTTTCACTTCTTATTACTCTTTTCGTTTACTTTTTCTA

ACATTTCTAGTACCAACTAATTCATTCGGGCGAGACATCTTACGATGTCATGATGCGCCCATTCCTATGG

CCATTCCTTTAATACTTCTGGCTTTCGGGAGTCTCTTTGTAGGATACTTGGCCAAACTAACACAAAGAAG

ATACAGTTCACTCAACGATTGCCTTTGGGTTCCGAACTCCATATGGGGAAGGAACGTTGTTGTTTGCGGG

GTCTCGATCATTTACATGGACCCACTTTTCATTCCATTTGTGGGAATTTGATAATCTATAAACCGTCCTT

AACGAACGATCGGCTCATCTT------TGAGCATGATGAATCACTTCGTGCCGACCCGTTGTCAATAAAC

TTTTGGGCCTCATATGAGAATGGAAAACTGGAGCATTTTCTGCATCGGTGGATGAAGAATCGCGAACATC

CAAATTTCTGGTTAAGCATGTTCCCAGAAAAAAGATACTTTCGAGAAACAACGAGCACGACTGAAGTGGC

TATCCATACAAATCCATTTACGGATCTATATGCTTCGATTGGAACTGGAAGTTCAAGAACAGGCGGCTGG

TATACTACCATAATGAAACTGCCTTTTCTTTTTTTTATTCGGATAGGATTTCTGTTGGCTTCGTTGGGAG

GCTCGCGTAGTTTGTTACGTCAACTCCAAAAGGATAAATTGCGTTGGAATTGAGAAAGTTACGTAA----

AGTTCATAATTGTATA--CAATTTTTGGGCCAATTCCCTCTTCGTACTACCAAAAAATGAGATTCTTGCC

GAATCCGAGTTTGCTGCTCCAACCATTACCAAACTAATACCTATTCTGTTTAGTACTTCAGGTGCTTCTG

TTGCGTATAATGTAAATCCCGTAGCGGATCAATTCC---------------AACGAGCCTTTCAAACTAG

TACTTTTTGTAATCGACTCTATAGCTTCTTCAATAAACGCTGGTTCTTCGATCAAGTTTTGAATGACTTT

CTAGTCAGATCGTTCTTGCGTTTCGGGTATGAAGTCTCATTCGAAGCTTTAGACAAAGGTGCTATTGAGA

TATTGGGCCCCTATGGTATCTCGTACACATTCCGACGATTGGCCGAGCGAATAAGTCAACTTCAAAGTGG

ATTTGTTTTGTTCCATGATCTATGGGTCTACAGGAGCTACCCATTTCGATCAATTAGCCAAGATTTTGAC

CGGATACGAAATCACTGGTGTTCAATCTAGTGGTATTTTTATGGGGATTCTTTTTATCGCTGTAGGATCC

CTATTCAAGATCACTGCAGTTCCTTTT-TGGTCTATGCACATCGCTTTCTCCAGGAGGTTGGCCGCCTAT

CCTAGATCTTCCCATTTCCAAGAGGATCCCGGGCTCGATCTGGTTTAGTATCAAGGTGATTCTCTTTCTC

TTTCTATATATATGGGTCCGTGCAGCATTTCCACGATATCGTTATGATCAATTAATGGGACTTGGCCGGA

AAGTGTTCTTGCCTCTATCATTAGCTCGGGTAGTCGCCGTTTCTGGTGTTTTAGTCACCTTTCAATGGCT

CCCTTA-ATGCCTCAACTGGATCAATTTACTTATTTCACACAATTCTTCTGGTCATGCCTCTTCTTCTTG

ACTTTCTATATTCTAATATGCAATGATAGAGATGGAGTACTTGGGATCAGCAGAATTCTAAAACTACGAA

ATCAACTGCTTTCACACCGGGGGAACAACATCCAAA------GCAAGGACCCCAAGAGTTTGGAAGATAT

CTTGAGAAAAGGTTTTCACACAGGTGTATCCTATATGTACTCTAGTTTATTCGAAGTATCCCAATGGTGT

AAGGCCGCCGACTTATTTGGAAAAAGGAAGAAAATCACTTTGATCCCTTGTTTCGGAGAAATAAGTGGCT

CACGAGGAATGGAAAGAAACATATTCTATTTGATCTCGAAGTCTTCATATAGCACTTC------------

---TTCCAATCCTGGATGGGTGATCACTTGTAAGAATGACATAATGCTAATCCATGTTCTACACGGCCAA

GGA--------------------

>Aegiceras corniculatum NC056358.1

ATGATACTTTCTGTTTTGTCAAGCCCTGCTTTGGTCTGTGGTTTGATGGTTGTACGTGCTAAAAATCCGG

TACATTCCGTTTCGTTTCCCATCCTAGTCTTTCGCGACACTTCAGGTTTACTTCTTTTGTTAGGTCTCGA

CTTCTTCGCTATGATTTTCCCAGTAGTTCATATAGGAGCTATAGCCGTTTCATTCCTATTCGTTGTTATG

ATGTTCCATATTCAAATAGCGGAGATTCATGAAGAAGTATTGCGCTATTTACCAGTGAGTGGTATTATTG

GACTGATCTTTTGGTGGGAAATGTTCTTCATATTAGATAATGAAAGCATTCCATTACTACCAACCCAAAG

AAATACGACCTCTCTGAGATATATGGTTTATGCCGGAAAGGTACGAAGTTGGACTAATTTGGAAACATTG

GGCAATTTACTTTATACCTACTATTCCGTCTGGTTTTTGGTTCCTAGTCTTATTTTATTAGTAGCCATGA

TTGGGGCTATAGTACTTACTATGCATAGGACGACCCACGGAGCGGTGAAAAGACAGGATGTATTCCGACG

AAATGCTATTGATTTTAGAAGGACTATAATGAGGAGGACGACAGAC-------AATATATTCATTGTTTC

ATTATTCGTTATTTCCGGGTCTTTTCGTTGCATTCACTTACAACAAGAAAGAACCACCAGTGTTTGGTGC

AGCACATGCATTTTGGTGCATTCTTCTTTCTTTCCTTGGTCTTTCGTTCCGTCATATTCCTAATAACTTA

TCCAATTACAACATATTAACTGCTAATGCACCTTTCTTTTATCAAATCTCAGGGACATGGTCTAATCATG

AGGGTAGTATTTTATCATGGTGTCGGATCCTAAGTTTTTATGGATTTATTCTTTGTTACCGGGGTCGACC

CCAAAGCCATAATGTCTCAAAACGAGGAGGCCATAGAGACACTTTTTTTTATTCCTTTGTCTTGAACTTC

GTGAAGAACTCCATTTTCTCTCTCCCTCGTTACGAACAAAAAAGTGGGGCT------GCGCCCAAGTTGT

ACACTCTCTTCGTTCTACGAA---CCCTTGTTGATTCTGAACTTCGTTCACGAAGGAACCGGACTTTTGA

CGGGCCAGCCCTTTTTTATGCGCCGCTTTCCCCTGAAAGGAAAATGAGCTTTGCTCCTCTGGGCGCTAGG

CGCTCCCGTGGTTCGCGAGAAGGAAAAAGTACTCATCCTTTGTTGCATCTGGCACGAGATGATAAAGAGA

GAGCTTCGTCTATCGATGAACAGCGGATTGACGGAGCTCTTGGCATTGCTTTGTTTTTCTCTCCTTTCCT

ATCAGCGAGTTCCGATCCTTTTGTTCAAAATTTCTTCGTTCGTACCGAACCGCTTGCAGAATCAAATCCT

GTTCCACAAGATCCTATATCAGCTATACATCCTCCTTGCATTTATGCCGGAGACGTCGCCAGTGCTATGG

GCTTTGGCTTATGTAGATCAAAAATGATGAATGGGATTGTGGCACTCCACTCGTCGCCAATGCGAAAGGA

TGCCGCCGAAAAGAATGGAACGCTGCTTCGCTCTGCTGGATGCGTCGGATCCCGTATAACAAGCGAGCTT

TTTACCCTAAAATTCAAAGATGTGGGTGCTTCATGCTATCCTGCTCTATTGTTGCGTAGCAATAGAAGCC

TGCTC---ATGCTGCTTCGGCGGTGCTTTTTCGCCTTCTCTTCGCTCTGGACAGGAGCGCTAGTGGACAC

GGGGAGGGAGCA------GGCGAAGCGTGTCG------TTCGTAATGGAAAGAAAGATACCACTACTTCG

CCTCTTTGTTGGACCGCCGGCGCGAACACAGTGGTCTCTGACCAGGACCAGGAACCAATTCGAATTTGGA

TCTTGACATGTCGGTGCTTTTTAACCGTAGGCATCTTGCCAGGAAGTTGGTGGGCTCATCATGAATTAGG

TTGGGGTGGTTGGTGGTTTCGGGATCCCGTAGAAAATGCTTCTTTTATGCCTCGGGTATTAGCCACAGCT

CGTATTCATTCAATAATTCTACCCCTTCTTCATTCTTGGACCTCGTTTCTTAATATTGTGACTCTTCCAT

GCTGTGTCTTAGGAACCTTTTCAATACGGTCCGGATTGCTAGCTCCCGTTCATAGTTTTGCTACAGATGA

TACACGAGGAATCTTTTTATGGCGGTTCTTCCTTCTAATGACCGGCATATCTATGATTCTTTTCTCCCAG

ATGAAGCAAGAGGCATCGGTCCGTAGAACTTATTAAAAAGAGATGGTTGTGGCGCGAAGTACTCTTGTGC

ACCTACGT--------------------CTATTTCTCACAG--GCTCCTTGTGATGCAGCAGAACCATGG

CAATTAGGATTTCAAGACGCAGCAACACCTATGATGCAAGGAATAATAGACTTACATCACGATATCTTTT

TCTTCCTCATTCTTATTTTGGTTTTCGTATCACGGATCTTGGTTCGCGCTTTATGGCATTTTCACTATAA

AAAAAATCCAATCCCGCAAAGGATTGTTCATGGAACTACTATCGAGATTATTCGGACCATCTTTCCTAGT

ATCATCCCGATGTTCATTGCTATACCATCATTTGCTCTGTTATACTCAATGGACGAGGTAGTCGTAGATC

CAGCCATTACTATCAAAGCTATTGGACATCAATGGTATCGGAG---------------------------

----------------------------------------------------------------------

----------------------------------------------------------------------

----------------------------------------------------------------------

----------------------------------------------------------------------

----------------------------------------------------------------------

-----------------------ATGAGACGACTCTTTTTTGAACTATATCATAAAAAGATCTTCCCCTC

CACACCAATCACGAGTTTTTCTCCATTCCTCTCGTATATCGTCATAACGCCCTTAATGCTAGGTTTTGAA

AAAGACTTTTCATGTCATTCCCATTTAGGTCCGATTCGGATCCCTCCGTTGTTTCCTTTTCCTTCCGCAC

CTTTTCCTCGAAATGAGAAAGAAGATGGTACACTTGAATTGTATTATTTAAGTGCTTATTGTTTGCCAAA

GATCCTACTTCTACAATTGATAGGTCACCGGGTTATTCAAATAAGTCGTGTTTTCCGTGGTTTTCCCATG

TTACAACTTCCGTACCAATTCGGTCGATCCGGAATGAATCGGTTAAACATTCTATTAGGGAGCCTGGTCT

TGACTCTTCTGTGTGGTATTCATTCTTGTTCGGCTCTTGGAATCACATCCAGCAGTGGTTGGAACAGCTC

GCAAAATCCAACCATTTCACCTACTTCATTGCCCCCAACCGTTTCTCGTACCTCTATTGAAACAGAATGG

TTTCATGTTCTTTCATCGATTGGTTATTCCTCTCTGTTCGTATCTCTTTTTCCAATTTCGGTCTCGATTA

GTTTACAAGATTG-ATGTCCGTTTCGTTATTACAACCTTC-----TTTTTGGATATCAAAGACCAGAAGC

TACGCGAAAATTCTCATTGGATCTCGGTTGTTCTTAACAGCGATGGCTATTCATTTAAGTCTTCGGGTAG

CACCACTAGATCTTCAACAAGGAGGAAATTCTTGTATTCTGTATGTACATGTTCCTGCGGCTCGGATGAG

TATTCTTGTTTATATCGCTACGGCTATAAACACGTTCTTGTTCCTATTAACAAAACATCCCCTTTTTCTT

CGCTCTTCCGGAACCGGTATAGAAATGGGTGCTTTTTCTACGTTGTTTACCTTAGTTACTGGGGGGTTTC

GGGGAAGACCTATGTGGGGCACCTTTTGGGTGTGGGATGCTCGTTTAACCTCTGTATTCATCTCGTTCCT

TATTTACCTGGGTGCACTGCGTTTTCAAAAGCTTCCTGTCGAATCGGCTCCTATTTCAATCCGTGCTGGA

CCGATCGATATACCAATAATCAAGTCTTCAGTCAACTGGTGGAATACATCGCATCAACCTGGGAGCATTA

GTAGATCTGGTACATCAATACATGTTCCTATGCCCATTCCAATCTTGTCTAACTTTGCTAACTCCCCCTT

GTCAACCCGTATCTTCTTTGTTCTGGAAACACGTCTTCCTATTCCATCTTTTTTCGAATCTCCTTTAACG

GAAGAAATAGAAGCTCGAGAAGGAATAC--GCAGGCTAGAAAGATGCTATTTGCTGCTATTCTATCTATT

TGTGCATCAAGTTCGAAGAAGATCTCAATCTATAATGAAGAAATGATAGTAGCTCGTTGTTTTATAGGCT

TTATCATATTCAGTCGGAAGAGTTTAGGTAATACTTTCAAAGTGACTCTCGACGGGAGAATCCAGGCTAT

TCAGGAAGAATCGCAGCAATTCCCCAATCCTAACGAAGTAGTTCCTCCGGAATCCAATGAACAACAACGA

TTACTTAGGATCAGCTTGCGAATTTGTGGCACCGTAGTAGAATCATTACCAATGGCACGCTGTGCGCCTA

AGTGCGAAAAGACAGTGCAAGCTTTGTTATGCCGAAACCTAAATGTTAAGTCAGCAACACTTCCAAATGC

CACTTCTTCCCGTCGCATCCGTCTTCAGGACGATCTAGTCACAGGTTTTCACTTCTCAGTGAGTGAAAGA

TTTGTACCCGGGTCTACGTTGAAAGCTTCTATAGTAGAACTCATTCGAGAGGGCTTGGCGGTCTTAAGAA

TGGTTCGGGTGGGGGATTTCTCTT-TGAAAGAGACGATCAGAATGGTACTCGAATCCATTTACGATCCCG

AGTTTCCAGACACATCGCACTTCCGCTCGGGTCGAGGCTGCCATTCGGCCCTAAGACGGATCAAAGAAGA

GTGGGGAACCTCTCGCTGGTTTTTGGAATTCGACATCAGGAAGTTTTTTCACACCATCGACCGACATCGA

CTCATCTCAATCTTTAAGGAAGAGATCGACGATCCCAAGTTCTTTTACTCCATTCAAACAGTCTTTTCTG

TCGGACGACTCGTAGAAGGTGAGAAGGGCCCTTACTCCGTTCCACACAGTGTACTACTATCGGCCCTACC

GGGCAACATCTACCTACACAAGCTCGATCAGGAGATAGGGAGGATCCGACAGAAGTACGAAATTCCGATT

GTTCAGAGAATAAGATCGGTTCTATTAAGGACAGGTCGTATTGATGAGCAAGAAAACTCTGGAGAAGAAG

CA-TGGAAATCTCTCCTAGAGCAGCAGAACTAACGACTCTATTAGAAAGTCGAATTACCAATTTTTACAC

GAATTTTCAAGTGGATGAGATCGGTCGAGTGATCTCAGTTGGAGATGGGATTGCACGTGTTTATGGATTG

AACGAGATTCAAGCCGGGGAAATGGTGGAATTTGCCAGCGGTGTGAAAGGAATAGCGTTGAATCTTGAGA

ATGAGAATGTAGGGATTGTTGTCTTTGGTAGTGATACTGCTATTAAAGAAGGAGATCTTGTCAAGCGCAC

TGGATCTATTGTGGATGTTCCTGCGGGAAAGGCTATGCTAGGGCGTGTGGTCGACGCGTTGGGAGTACCT

ATTGATGGAAGAGGGTCTCTAAGCGATCACGAGCGAAGACGTGTCGAAGTGAAAGCCCCTGGGATTATTG

AACGTAAATCTGTGCACGAGCCTATGCAAACAGGGTTAAAAGCGGTAGATAGCCTGGTTCCGATAGGCCG

TGGTCAACGAGAACTTATAATCGGGGACCGACAAACTGGAAAAACCGCTATTGCTATCGATACCATATTA

AACCAAAAGCAAATGAACTCAAGGAGCACCTCTGAGAGTGAGACATTGTATTGTGTCTATGTAGCGATTG

GACAGAAACGCTCAACTGTGGCACAATTAGTTCAAATTCTTTCAGAAGCGAATGCTTTGGAATATTCAAT

TCTTGTAGCAGCCACCGCTTCGGATCCTGCTCCTCTGCAATTTCTGGCCCCATATTCTGGGTGTGCCATG

GGGGAATATTTCCGCGATAATGGAATGCACGCATTAATAATATATGATGATCTTAGTAAACAGGCGGTGG

CATATCGACAAATGTCATTATTGTTACGCCGACCACCAGGCCGTGAGGCTTTCCCAGGCGATGTTTTCTA

TTTACATTCCCGTCTCTTAGAAAGAGCCGCTAAACGATCGGACCAGACAGGCGCAGGTAGCTTGACCGCC

TTACCCGTCATTGAAACACAAGCTGGGGACGTATCGGCCTATATTCCCACCAATGTGATCCCCATTACTG

ATGGACAAATCTGTTTGGAAACAGAACTCTTTTATCGCGGAATTAGACCTGCTATTAACGTCGGCTTATC

TGTCAGTCGCGTCGGGTCTGCCGCTCAGTTGAAAGCTATGAAACAAGTCTGCGGTAGTTCAAAACTTGAA

TTGGCACAATATCGCGAAGTGGCCGCCCTTGCTCAATTTGGGTCAGACCTTGATGCTGCGACTCAGGCAT

TACTCAATCGAGGTGCAAGGCTTACAGAAGTACCGAAACAACCACAATATGCACCACTTCCAATTGAAAA

ACAAATTCTAGCCATTTATGCAGCAGTCAATGGATTCTGTGATCGAATGCCATTAGACAAAATTTATCAA

TATGAGAGAGCCATTCCAAGTAGTGTAAAACCAGAATTACTACAATCTCTTTTAGAAAAAGGTGGGTTAA

CTAACGAAAGAAAGATAGAACCAGATGCATTCTTAAGAGAAAATGCTTTGCCTTACCTATGATGCA----

--------CATCCAACGCAAAGCGGCCTTTCATTCCCTTGTTTCGTCGTGGCACACCCCCCCCACAAGCA

CCCCTCGGCTCAGGGGAGACCAGAAAACGCCTTTCTTTTTCGCCCCTTCGTCGGCCCTTGCCGCCTTCCA

ACACAAGCCCTCGAGCCTCCTTTGCGCCGCCTTCCTCATAGAAGCCGCCG------CCCCGAAGGCCGAA

TTCTATGGTAGAGAAGGCTTTAATAAAAATGGGGCCATGAGAGACTTTTTTAAGTATTGCAAAAGAAAGG

GCCTGCTGATAGAGCTGGGCGGGGAGGCGATACTAGTTATCAGGTCAGAGAGACGCCTGGCCTGTAAGCT

GGCCCCCTTAAAAACCCATTACTTAATAAGGATTTGTTACGCGCGATATGCCGACGACTCACTACTGGGA

ATCGTGGGTGCCGTAGAGCTTCTCATAGAAATACAAAAATGTATCGCCCACTTCCTACAATCCGGCCTGA

ACCTTTGGCTAAGCTCTGCAGGATCAACAAAAAGAACTGCACGGAGTACGGTAGAATTCCTTGGTACGAT

CATTCGGGAAGTCCCTCCGAGGACTACTCTCATACAATTATTGCGAGAGCTGGAGAAGCGTCTACGGGTA

AAGCACCGTATCCATATAACTGCTTGCCACCTACGCTCGGCCATCCATTCCAAGTTTAGGAACCGCGGGT

TTAGTATCCCGATCAAACAGCTGACGAAGGGGATGAGCGGAACAGGGAGTCTACTGGACGCGGTTCAACT

AGCAGAGACTCTTGGAACAGCTGGAGTAAGAAGTACCCAAGTGAGCGTATTATGGGGGACCGTCAAGCAC

ATCCGGCAAGGCTCAAGGGGGATCTCGTTGTTGCATAGCTCAGGTCGGAGCAAGGTGCCATCGGACGTTC

AACAGGCAATCTCACGATCGGGCACTTATGCCCGGAAGTTGTC---ATTGTATACTCCCGCGGGTCGGAA

GGCGGCGGGGGAAGGAGGGGGACACTGGGCGAGATCTATCAGCAGCGAATTCCCCATACAAATAGAGGCA

CCTATCAAAAAGATAGTCCGAAGGCTTCGGGATCGAGGTATCATTAGCCGAAAAAGACCCTGGCCAATTC

ACGTGGCCTGCTTGACGAACGCCAGCGACGAAGACATCGTAAATTGGTCCGCGGGCATTGCGATAAGTCC

TCTGTCCTACTACAGGTGCTGCGACAACCTTTACCAAGTTCGAACGATTGTCGACCACCAGATCCGCTGG

TCTGCAATATTCACCCCGGCCCACAAGCACAAATCCTCGGCGCGGAATATAATCCCAAAGTACTCCAAAG

ACTCAAATATAGTCAATCAAGAAGGTGGTAAGACCCTTGCAGAGTTCCCCAACAGCATAGAGCTTGGGAA

GCTCGGACCCGGTCAATATCC-GAACAACAAGGAGCACTCAACTACTA-----------------ATGGT

CCAACTACATAACTTTTTCTTTTTAATTACTTCCATGGTCGTGCCTCGTGGCACGGCAACACCCGTACTA

TTGAAATGGTTCGTTAGTAGAGATGTTCCCACAGGTGCCCCTTCTTCCAATGGTACCATAATTCCTATCC

CTATCCCTTCATTCCCTCTTTTTGTCTATCTACATTTCAGGAAAATCATACGCTCTACGGACGGAGCAAA

AAGTGGAGTCTTGGTCAGAGCAAGCCGCCCTATTCTATTACCAGTACCAGACATAATTGGGAGAAGCTTA

TCCTTTTCTAGAGCAAGAAAGGCTGAATTTCGTTTTGTTCCTGTTCTTCATTTCCTTCTTCTTCAATCCA

A------GGGGTACTTCTCATATTTAGAATCTTTCTGCGGTGTGCTCTGTTTACTATTCTTTCGTACTTT

ATTCTTTTTACCACGCGATAGGTCAGCGAAGCGTGAGCGGGCGCGGAGAAGGAAAGGCCAAACACTTCGG

CCTAAC------GGGAATGAGCAAGGAGGAAATGACAAGATGAGGTGCCCCGGGCATCCCCATT------

TAGA---AAGAAGGATCGAAGGTTTTGGGCCTGTAGCTTTCTCCGTCCCCCCTTCGTCGGGTGGTCCTTG

TGTGGGGGGTGCGCCACCAGAAATCGGGCTTGAAGCTCTCGCCTTACCAAGGAGCCGACAGCTGATGGCT

GTTGGTCACGACTACTACCAAAAAGCTTCAATGAAGATTAATATTTCACATGGAGGAGTGTGCATCTTTA

TGTTGGGTGTTCTTCTG---GTACATTGCTGTTCCAACTGAAATACTTGGAATAATTCTACCACTTCTAC

TCGGAGTAGCCTTTTTAGTGCTAGCTGAACGTAAAGTAATGGCTTTTGTGCAACGTCGAAAGGGTCCTGA

TGTAGTGGGATCCTTTGGATTGTTACAACCTCTAGCAGATGGTTTGAAATTGATTCTAAAAGAACCTATT

TCACCAAGTAGTGCTAATTTCTCCCTTTTTAGAATGGCTCCAGTGGCTACATTTATGTTAAGTCTGGTCG

CTCGGGCCGTTGTACCTTTTGATTATGGTATGGTATTGTCAGATCCGAACATAGGGCTACTTTATTTGTT

TGCCATATCTTCGCTAGGTGTTTATGGAATTATTATAGCAGGTTGGTCTAGT--TTATTATATACGCTTA

GCGAAAAGAATGTTTTTTGATACACCTAGGACATGGATTCTATATGAACCAATGGATCGTGACAAGTCGT

TACTACTAGCAATGACTTCCTCTTTCATTACTTCATTCTTTCCATATCCCTCTCCTTTGTTCTCAGTTAC

TCATCAAATGGCACTCAGTTCATATCTTTAA-TGTCAGAATTTGCACCTATTTTTATCTATTTAGTGATC

AGTCCGCTAGTTTCTTTGATCCCACTCGGTCTTCCTTTTCTATTTGCTTCCAATAGTTCGACCTATCCAG

AAAAATTGTCGGCCTACGAATGTGGTTTCGATCCTTTCGGTGATGCCAGAAGTCGTTTCGATATAAGATT

TTATCTTGTTTCAATTTTATTTATTATCCCTGATCTGGAAGTAACCTTTTTCTTTCCTTGGGCAGTCTCT

CTCAACAAGATTGATCCGTTTGGATCTTGGTCCATGATGGGCTTTTTATTGATTTTGACGATTGGATCTC

TCTATGAATGGAAAAGGGGTGCTTCGGATCGGGAATAA---------------------------CAGCC

CACTTGAGCAATTTGCCATTTTCCCATTGATTCCTATGAATATAGGAAACTTTTATTTCTCATTCACAAA

TCCATCTTTGTTTATGCTGCTAACTCTCAGTTTGGTCCTACTTCTGGTTCATTTTGTTACTAAAAAGGGA

GGAGGAAACTCAGTACCAAATGCTTGGCAATCCTTGGTAGAGCTTATTTATGATTTCGTGCCGAACCCGG

TAAACGAACAAATAGGTGGTCTTTCCGGAAATGTGAAACAAAAGTTTTTTCCTCGCATCTCGGTCACTTT

TACTTTTTCGTTATTTCGTAATCCCCAGGGTATGATACCTTATAGCTTCACAGTTACAAGTCATTTTCTC

ATTACTTTGGGTCTCTCATTTTCTCTTTTTATTGGCATTACTATAGTGGGATTTCAAAAAAATGGGCTTC

ATTTTTTAAGCTTCTTATTACCCGCAGGAGTCCCACTGCCGTTAGCACCTTTTTTAGTACTCCTTGAGCT

AATCCCTCATTGTTTTCGCGCATTAAGCTCAGGAATACGTTTATTTGCTAATATGATGGCCGGTCATAGT

TCAGTAAAAATTTTAAGTGGGTTCGCTTGGACTATGCTATGTATGAATGATCTTTTATATTTCATAGGAG

ATCTTGGTCCTTTATTTATAGTTCTTGCATTAACCGGTCTGGAATTAGGTGTAGCTATATCACAAGCTCA

TGTTTCTACGATCTCAATCTGTATTTACTTGAATGATGCTACAAATCTCCATCAAAGTGGTTCTTTCTTT

TTTTTAATTGAACAAA-TATGTGGGCGCCTGATATCTATGAGGGTTCACCCACCCCGGTTACAGCATTCC

TTTCTATTGCGCCTAAAATATCTATTTCTGCTAATATTTCACGTGTTTCTATTTATGGTTCCTATGGAGC

TACATTGCAACAAATCTTCTTTTTCTGCAGCATTGCTTCTATGATCTTAGGAGCACTGGCCGCCATGGCC

CAAACGAAAGTAAAAAGACTTCTAGCTTATAGTTCAATTGGACATGTAGGTTATATTCGTACTGGTTTCT

CATGTGGAACCATAGAAGGAATTCAATCACTACTAATTGGTATCTTTATTTATGCATCAATGACGATAGA

TGCATTCGCCATAGTTTTAGCATTACGGCAAACCCGTGTCAAATATATAGCGGATTTGGGCGCTCTAGCC

AAAACGAATCCTATTTCGGCTATTACCTTCTCCATTACTATGTTCTCATACGCAGGAATACCCCCGTTAG

CCGGCTTTTGTAGCAAATTCTATTTGTTCTTCGCCGCTTTGGGTTGTGGGGCTTACTTCCTAGCCCTAGT

GGGAGTAGTGACTAGCGTTATAGGTCGTT-GTTCGATAGCCCGACAGTAGTGATGTTAATTGTGGTTACA

TTCATAAGTAGCTTGGTCCATCTTTATTCCATTTCATATATGTCTGAGGATCCGCATAGCCCTCGATTTA

TGTGTTATTTATCCATTCCTACTTTTTTTATGCCAATGTTGGTGACTGGAGATAACTCTCTTCAATTATT

CCTGGGATGGGAGGGAGTAGGTCTTGCTTCATATTTGTTAATTCATTTCTGGTTTACACGACTTCAGGCA

GATAAAGCAGCTATAAAAGCTATGCCTGTCAATCGAGTAGGTGATTTTGGATTAGCTCTTGGGATTTCGG

GTTGTTTTACTCTCTTTCAAACAGTAGACTTTTCAACCATTTTTGCTTGTGCTAGTGCCCCCAGAAATTC

TTGGATTTCTCGCAATATGAGATTGAATGCCATAACTCTTATTTGTATTTTACTTCTTATTGGTGCTGTT

GGGAAATCTGCACAGATAGGATCGCATACTTGGTCACCCGATGCTATGGAGGGTCCCACTCCAGTATCCG

CTTTGATTCATGCAGCTACTATGGTAACAGCTGGCGTTTTCATGATAGCAAGATGTTGCCCTTTATTTGA

ATACCCACCTACGGCTTTGATTGTTATTACTTTTGCAGGAGCTATGACGTCATTCCTTGCGGCAACTACA

GGAATATTACAGAACGATCTAAAGAGGGTCATAGCTTATTCAACTTGCAGTCAATTAGGCTATATGATCT

TTGCTTGCGGCATCTCTAACTATTCGGTTAGCGTCTTTCACTTAATGAATCACGCGTTTTTCAAAGCATT

ACTATTCCTGAGTGCAGGTTCGGTGATTCATGCCATGTCGGATGAGCAAGATATGCGGAAGATGGGGGGG

CTTGCCTCCTCGTTCCCTTTTACCTATGCCATGATGCTCATGGGCAGCTTATCTCTAATTGGATTTCCTT

TTCTAACTGGATTTTATTCAAAAGATGTGATCTTAGAGCTCGCTTACACTAAGTATACCATCAGTGGGAA

CTTTGCTTTCTGGTTGGGAAGTGTCTCTGTCCTTTTCACTTCTTATTACTCTTTTCGTTCACTTTTTCTA

ACATTTCTAGTACCAACTAATTCATTCGGGCGAGACCTCTTACGATGTCATGATGCGCCCATTCCTATGG

CCATTCCTTTAATACTTCTGGCTCTCGGGAGTCTCTTTGTAGGATACTTGGCCAAACTAACATAAAGAAG

ACACAGTTCACTCAACAATTGCCTTTTGGTTCCGAACTCCATATGGGGAAGGAGCATTGTTGTTTGCAAG

GTCTCGATCATTTACATGGACCCACTTTTCATTCCATTTGTGGGAATTTTTTAATATATAAACCGTCCTT

AACGAATGATCGGCTCATCTT------TGAGCATGATGAATCACTTTGTCCCGACCTGTTGCCAATAAAC

TTTCCGGCCTCATATGAGAATGGAAACCTAGAGTGTTTTCTGCATTGGTGGATGAAGAATCGCGAACATA

ATAATTTCTGCTTGACCATGTTCCCAGAAAAAAGATATTTTCAAGAAACGACGAGTACGATTGAAGTGTC

AATACATACAAATGTATTTACGGATCTATATGCTTTGATTGGAATTGGAAGTTCCAGAACAGGCGGCTGG

TATACGACCATAATGAAACTGCCTTTTCTTTTTTTTATTTGGATAGGATTTATGTTGGCTTCGTTGGGAG

GCTCGCCTAGTTTGTTACGTCAGCTCCAAAAGGATAAGTTGCGTTGG-----------------------

------------------CAATTTTTGGGCCAATTCCCCCTTCGTACTACCAAAAAATGAGATTCTTGCC

GAATCCGAGTTTGCTGCTCCAACCATTACCAAACTAATACCTATTCCGTTTAGTACTTCAGGTGCTTCTG

TTGCGTATAATGTAAATCCCGTAGCGGATCAATTCC---------------AACGAGCTTTTCAAACTAG

TACTTTTTGTAATCGACTCTATAGCTTCTTCAATAAACGCTGGTTCTTCGATCAAGTTTTGAATGACTTT

CTAGTCAGATCGTTCCTGCGTTTCGGATATTCAGTCTCATTCGAAGCTTTAGACAAAGGTGCTATTGAGA

TATTGGGCCCTTATGGTATCTCGTACACATTCCGACGATTGGCCGAGCGAATAAGTCAACTTCAAAGTGG

ATTTGTT-TGTTCCATGATCTATGGGTCTACTGGAGCTACCCACTTTGATCAATTAGCAAAGATTTTGAC

CGGATACGAAATCACTGGTGCTCGATCTAGTGGTATTTTTATGGGGATTCTATCTATCGCTGTAGGATCC

CTATTCAAGATCACTGCAGTTCCTTTTC-GGTCCATGCACATCGCTCTCTCCAGGAGGTTGGCCGCCTAT

CCTAGATCTTCCCATTTCCAAGAAGATCCCGGGCTCGATCCGGTTTAGTATCAAGGTGATTCTTTTTCTG

TTTCTATATATATGGGTCCGTGCAGCATTTCCACGATATCGTTATGATCAATTAATGGGACTTGGCCGGA

AAGTGTTCTTGCCTCTATCATTAGCTCGGGTAGTCGCTGTTTCCGGTATTTTAGTCACCTTTCAATGGCT

CCCTTAAATGCCTCAACTGGATAAATTCACTTACTTCACACAATTCTTCTGGTTATGCCTTTTCCTCTTT

ACTTTCTATATTCCCATATGCAAT------GATGGAGTACTTGGGATCAGCAGAATTCTAAAACTACGGA

ACCAACTGGTTTCACACCGGGAGAACAACATCCGGA------GCAAGGACCCCAAGAATTTGGAAGATAT

CTTGAGAAAAGGTTTTAGCACCGGTGTCTCTTATATGTACTCCAGTTTATTCGAAGTATCCCAATGGTGT

AACGCCGTCGACTTATTGGGAAAAAGGAGGAAAATTACTTTGATCTCTTGTTTCGGAGAAATAAGCGGCT

CACGAGGAATGGAAAGAAACATATTCTATTTGATCTCGAAGTCCTCATATAGCACTTC------------

---TTCCAATCCTGAATGGGCGATCACTTGTAGGAATGACATAATGCTAATCCATGTTCCACACGGCCAA

GGAAGCATCGGTTTTTAA-----

>Chenopodium quinoa NC041093.1

-TGATACTTTCTGTTTTGTCGAGCCCGGCTTTGGTCTCTGGTTTGATGGTTGTACGTGCTAAAAATCCGG

TACATTCCGTTTTGTTTCCCATCCCAGTCTTTCGCAACACTTCAGGTTTACTTCTTTTGTTAGGTCTCGA

TTTTTCCGCCATGATCTTCCCAGTAGTTTATATAGGAGCTATAGCCGTTTCATTCCTATTCGTTGTTATG

ATGTTCCATATTCAAATAGCGGAGATTCACGAAGAAGTATTGCGCTATTTACCAGTGAGTGGTATTATTG

GACTGATCTTTTGGTGGGAAATGTTCTTCATTTTAGATAATGAAACCATTCCATTACTACCAACCCAAAG

AAATACGACCTCTCTGAGATATACGGTTTATGCCGGAAAGGTACGAAGTTGGACTAATTTGGAAACATTG

GGCAATTTACTTTATACTTACTATTTTGTCTGGTTTTTGGTTTCTAGTCTTATTTTATTAGTAGCCATGA

TTGGGGCTATAGTACTGACTATGCATAGGACTACTAA------GGTGAAAAGACAGGATGTATTCCGACG

AAATGCTATTGATTCTAGAAGGACTATAATGAAGAGGACGACAGACC--TGTCAATATATGAATTGTTTC

ATTATTCGTTATTTCCGGGTCTTTTCATTGCATTCACTTACAACAAGAAACAACCACCAGCGTTTGGTGC

AGCACCTGCATTTTGGTGTATTCTTCTTTCTTTCCTTGGTCTTTTGTTCTGTCATATTCCTAATAACTTA

TCCAATTACAACGTATTAACCGCTAATGCACCTTTCTTTTATCAAATCTCAGGGACATGGTCTAATCATG

AAGGTAGTATTTTATTATGGTGTCGGATCCCAAGTTTTTATGGATTCCTTCTTTGTTACCGGGGTCGATC

CCAAAGCCATAATGTCTCAAAACGAGGAGGCCGTAGAGAAAGTCTTCTTTTTTCCTTTGTCTTAAACTTC

GTGAAGAACTCCATTCTATCTCTTCCTCGTTACGAACAAAAAAGTAGAGTTCTTCACGAACCCCAGTTGT

ACACTCTCTTCGTTCTACGAA---CTCTTGTTGATTCTGAACTTTGTTCGCGAAGGAACCGGACTTTTGA

CGGGCCAGTTATTTTTGACGCGCCGCTTTACCCTGAAAGGAAAATGAGCTTTGCTCTTCTGGGCGCTAGG

CGCTCTCGTGGTTCGCGAGAAGGAAAAAGGACTCATCCTTTGTTGCATCTGGCACGAGATGATAAAGAGA

GAGCTTCGTCTATCAATGAACAGCGGATTGACGGAGCTCTTGGCATTGCTTTCTTTTTCTTTCCTTTCCT

ATCAGCGAGTTCCGATCCTTTTGTTCGAAATTTCTTCGTTCGTACCGAACCGCTTGCAGAATCAAATCCT

GTTCCACAAGATCCTATATCAGCTATACATCCTCCTTGCATTTATGCCGGAGACGTCGCCAGTGCTATGG

GCTTTGGCTTATGTAGATCAAAAATGATGAATGGGATTGTGGCACTCCACTCGCCGCCAATGCGGAAGGA

TGTCGCCGAAAAGAATGGAACGCTGCTTTGCTCTGCTGGATGCGTCGGATCCCGTATAACAAGCGAGCTC

TTTACCCTTAAATTCAAACATGTGGGCGCCAAATGCTATCCTGCTCTATTGTTGCGTAGCAAAAGAAGCC

TGCTC---ATGCTGCTTCGACGGCGCTTTTTCGCCTTCTCTTCGCTCTGGACAAGAGCGCTAGTGGACAC

GGGGAGGGAGCG------GGCGAAGCGTTTCT------TTCGTAATGGAAAGACAAAGACCACTACTTTG

CCTCTTTGTTGGACCGCCGGCGCGAACACAGTGGTCTCTGACCAGGACCAGGAACCAATTCGAATTTGGA

TCTTGACATGTCGGTGCTTTTTAACCGTAGGCATCTTGCCAGGAAGTTGGTGGGCTCATCATGAATTAGG

TCGGGGTGGCTGGTGGTTTCGGGATCCCGTAGAAAATGCTTCTTTTATGCCTCGGGTATTAGCCACAGCT

CGTATTCATTCAGTAATTTTACCCCTTCTTCATTCTTGGACTTTGCTTCTTAATATTGTGACTTTTCTAT

GCTGTGTCTTAGGAACCTTTTCAATACGGTCCGGATTGCTAGCTCCCGTTCATAGTTTTGCTACAGATGA

TACACGAGGAATCTTTTTATGGCGGTTCTTCCTTCTAATGACCGGCATATCTATGATTCTTTTCTCTCAG

ATGAAGCAGCAGGCATCGGTCCGTAGAACCTATAAAAAAGAGATGGTTGTAGCGCGAAGTACTCTTGTGC

ACTTACGT---TGATTGTTCGAGAATGGCTATTCTTCACAATTGCTCCTTGTGATGCAGCGGAACCATGG

CAATTAGGATTTCAAGACGCAGCAACACCTATGATGCAAGGAATAATAGACTTACATCATGATATCTTTT

TCTTCCTCATTCTTATTTTGGTTTTCGTATCATGGATCTTGGTTCGCGCTTTATGGCATTTCCACTATAA

AAAAAATCCAATCCCGCAAAGGATTGTTCATGGAACTACTATCGAGATTATTCGGACCATATTTCCCAGT

ATCATCCTGATGTTCATTGCTATACCATCATTTGCTCTGTTATACTCAATGGACGAGGTAGTAGTAGATC

CAGCCATTACTATCAAAGCTATTGGACATCAATGGTATCGGAGT--------------------------

----------------------------------------------------------------------

----------------------------------------------------------------------

----------------------------------------------------------------------

----------------------------------------------------------------------

----------------------------------------------------------------------

-----------------------ATGAGACGACTCTTTTTTGAACTATATCATAAACAGATCTTCTTCTC

CACACCAATCACGAGTTTTTCTCCATTCCTCTCGTATATTGTCGTAACGCCCTTAATGCTAGGTTTTGAA

AAAGACTTTTCATGTCATTTCCATTTAGGTCCGATTCGGATCCCTCTGTTGTTTCCTTTTCCTCCCGCAC

CTTTTCTTCGAAATGAGAAAGAAGATGGTACACTCGAATTGTATTATTTAAGTGCTTATTGCTTGCCAAA

GATCCTACTTCTACAATTGGTAGGTCACCGGGTTATTCAAATAAGTCGTGTTTTCTGTAGTTTTCCCATG

TTACAACTTCTGTACCAATTCGGTCAATCCGGAATGGATCGGTTAAACATTCTATTAGGGAGCCTGGTCT

TGACTCTTCTGTGCGGTATTCATTCTTGTTTGGCTCTTGGAATCACATCCAGCAGTGGTTGGAACAGCTC

GCAAAATTTAACCACTTCACCTACTTCATTGCCCTCAACCGTTTCTCGTACCTCTATTGAAACAGAATGG

TTTCATGTTCTTTCATCGATTGGTTATTTTTCTTCGTTCGTATCTCTTTTTCCAATTTCGGTCTCGATTA

GTTCACAAGATTG--------TTTCTTTATTACAACCTTT-----TTTTTTGATGTCAAAGACCAGGAAC

TACGCGCAAATTCTCATTGGATCTTGGTTGTTCTTAACAGCGATGGCTATTCATTTAAGTCTTTGGGTAG

CACCACTAGATTTTCAACAAGGTGGAAATTCTCGTATTCTCTATGTACATGTTCCTGTGGCTCGGATGAG

TATTCTTGTTTATATCGTTACGGCTATAAACACTTTCTTGTTCCTATTAACAAAACATCCTCTTTTTCTT

CGCTCTTCCGGAACCGGTACAGAAATGGGTGCTTTTTCCACGTTGTTTACCTTAGTTACTGGGGGGTTTC

GGGGAAGACCCATGTGGGGCACCTTTTGGGTGTGGGATGCGCGTTTAACTTCTGTATTCATCTCGTTCCT

TATTTACTTGGGTGCGCTGTGTTTTCAAAAGCTTCCTGTAGAATTGGCTCCTATTTCAATCCGTGCCGGA

CCGATCGATATACCAATAATCAAGTCTCCGGTCAACTGGTGGAATACATCGCATCAACCTGGGAGCATTA

GCCAATCTGGTACATCAATACATGTTCCTATGCTCATTCCAATCTTGTCTAACTTTGCTAACTTCCTCTT

CTCAACCCGTATCTTCTTTGTTATGGAAACACGTCTTCCTATTCCATCTTTTCTCGAATCTCCTTTAACG

GAAGAAATAGAAGCTCGAGAAGGAATA---GCAGGCTAGAAAGATGCTATTTGCTGCTATTCTATCTATT

TGTGCATCAAGTTCGAAGAAGATCTCAATCTATAATGAAGAAATGATAGTAGCTCGTTGTTTTATAGGCT

TTATCATATTCAGTCGGAAGAGTTTAGGGAATACTTTCAAAGCAACTCTCGACGAGAGAATCCAGGCTAT

TCAGGAAGAATCGCAGCAATTCCCCAATCCTAACGAAGTAGTTCCTCCGGAATCCAATGAACAACAACGA

TTACTTAGGATCAGCTTGCGAATTTGTGGAACCGTAGCAGAATCATTACCAATGGCACGCTGTGCGCCTA

AGTGCGAAAAGACAGTGCAAGCTTTGTTATGTCGAAACCTAAATGTTAAGTCAGCAACACTTCCAAATGC

CACTTCTTCCCGTCGCAACCGTCTTCAGGACGATCTAGTCACAGGGTTTCACTTCTCAGTGAGTGAAAGA

TTTGTCCCCGGATCTACGTTGAAAGCTTCTATAGTAGAACTCATTCGAGAAGGCTTGGCGGTCTTAAGAA

TGGTTCGGGTAGGAGGTTCTCTT--TGAAAGAGGCGATCAGAATGGTACCCGAATCCATTTACGATCCCG

AGTTTCCAGACACATCGCACTTCCGCTCGGGTCGAGGCTGCCATTCGGCCCTAAGACGGATCAAAGAAGA

GTGGGGAACCTCTCGCTGGTTTTTGGAATTCGACATCAGGAAGTGTTTTCACACCATCGACCGACATCGA

CTCATCCCAATCTTTAAGGAAGAGATCGACGATCCCAAGTTCTTTTACTCCATTCAGAAAGTCTTTTCTG

CCGGACGACTCGTAGGAGGTGAGAGGGGCCCTTACTCCGTCCCACACAGTGTACTACTATCGGCCCTACC

AGGCAACATCTACTTACACAAGCTCGATCAGGAGATAGGGAGGATCCGACAGAAGTACGAAATTCCGATT

GTTCAGAGAATCAGATCGGTTCTATTAAAGACAAGTCGTATTGATGACCAAGAAAACTCTGGAGAAGAAG

---TGGAATTTTCTTCCAGAGCTGCGGAACTAACGACTCTATTAGAAAGTAGAATTACCAACTTTTACAC

GAATTTTCAAGTGGATGAGATCGGTCGAGTGGTTTCAGTTGGAGATGGGATTGCACGTGTTTATGGATTG

AACGAGATTCAAGCCGGGGAAATGGTGGAATTTGCCAGCGGTGTGAAAGGAATAGCCTTAAATCTTGAGA

ATGAGAATGTAGGGATTGTTGTCTTTGGTAGTGATACCGCTATTAAAGAGGGAGATCTTGTCAAGCGCAC

TGGATCTATTGTGGATGTTCCTGCGGGAAAGGCTATGCTAGGGCGTGTGGTCGACGCGTTGGGAGTACCT

ATTGACGGAAGAGGGGCTCTAAGCGATCACGAGCGTCGACGTGTCGAAGTGAAAGCCCCGGGGATTATTG

AACGTAAATCTGTGCACGAGCCTATGCAAACCGGGTTAAAGGCGGTAGATAGCCTGGTTCCTATAGGCCG

TGGTCAACGAGAACTTATAATCGGGGACCGACAAACGGGAAAAACTGCTATTGCTATCGATACCATATTA

AACCAAAAGCAACTGAACTCAAAGGCCACCTCTGAGAGTGAGACATTGTATTGTGTCTATGTAGCGGTTG

GACAGAAACGTTCAACTGTGGCACAATTAGTTCAAATTCTTTCAGAAGCGAATGCTTTGGAATATTCCAT

TCTTGTAGCAGCCACCGCTTCGGATCCTGCCCCTCTTCAATTTCTGGCCCCATATTCCGGGTGTGCTATG

GGAGAATATTTCCGCGATAATGGAATGCACGCATTAATCATCTATGATGATCTTAGTAAACAGGCGGTGG

CATATCGACAAATGTCATTATTGTTACGCCGACCACCAGGCCGTGAGGCTTTCCCAGGCGACGTTTTCTA

TTTACATTCCCGTCTCTTAGAAAGAGCCGCTAAACGATCGGACCAGACAGGTGCCGGTAGCTTGACCGCC

TTACCCGTCATTGAAACACAAGCTGGAGACGTATCGGCCTATATTCCCACCAATGTGATCTCCATTACTG

ATGGACAAATCTGTTTGGAAACAGAGCTCTTTTATCGCGGAATTAGACCTGCTATTAACGTCGGCTTATC

TGTCAGTCGCGTCGGGTCTGCCGCTCAGTTGAAAGCTATGAAACAAGTCTGCGGGAGTCTAAAACTGGAA

TTGGCACAATATCGCGAAGTGGCCGCCTTTGCTCAATTTGGGTCAGACCTTGATGCTGCGACTCAGGCAT

TACTCAATAGAGGTGCAAGGCTTACAGAAGTACTGAAACAACCACAATATGCACCACTTCCGATTGAAAA

ACAAATTCTAGTCATTTACGCAGCTGTCAATGGATTCTGTGATCGAATGCCACTAGATAAAATTTTTCAA

TATGAGAGAACCATTCCAAATAGTGCAAAACCAGAATTATTACAATCCCT--------------------

----------------------------------------------------------------------

--------CATCCAACGCAAAGCGGCCTTTCATTCCCTTGTTTCGTCGTGGCACACCCTCCCCGCAAGCA

CCCCCCGGCTCAGGGGGGACCAGAAAAGGCCCTTCGTTTTCCCCCCTTCGTCGGCCCTTGCCGCCTTCCT

TAACAAGCCCTCGAGCCTCCTTTTCGCTGCGTTCCTCATAGAAGCCGCCGGGTTGACCCCGAAGGCCGAA

TTCTATGGTAGAGAATGCTGTAATAATAATTGGGCCATGAGAGACCTTTTTAAGTATTGCAAAAGAAAGG

GCCTGCTGATAGAGCTGGGCGAGGCAGCGATACTAGTTATCAGGTCAGAGAAAGGCCTGGCCCGTAAGCT

GGCCCCCTTAAAAACCCATTACTTAATAAGGATTTGTTACGCGCGATATGCCGACGACTTACTATTGGGA

ATCGTGGGTGCCGTAGAGCTTCTCATAGAAATACAAAAACGTATTGCCCACTTCCTACAATCCGGCCTGA

ACCTTTGGGTAGGCTCTGCGGGATCAACAACAATAGCTGCACGGAGTACGGTAGAATTCCTCGGTACGGT

CATTCGGGAAGTCCCTCCGAAGACGACTCCCATACAATTCTTGCGAGAGCTGGAGAAGCGTCTACGGGTA

AAGCACCGTATCCATATAACTGCTTGCCACTTACGCTCTGCCATTCATTCCAAGTTTAGGAACCTAGGTA

ATAGTATCCCGATCAAACAGCTGACGAAGGGGATGAGCAAAACAGGGAGTCTACTGGACGCGGTTCAACT

AGCGGAAAGTCTTTCCACAGCTAGAGTAAGAAGTCCCCAAGTGAGCGTATTATGGGAGACCGTCAAGCAC

ATCCGGCAAGGATCAAGGGAGATCTCGTTGTTGCATAGCTCAGGTCAGAGCAAGGTGCCATCGGATGTTC

AACAGGAAGTCTCGCGATCGGGCATGAGTGTCCGGAAGTTGTC---ATTGTATACTCTCGCGGGTCGGAA

GGCGGCGGGGGAAGGAGGGGGACACTGGTCGAGATCTATCAGCAGCGAATTCCCCATACAGATAGAAGCG

CCTATCAAAAAGATACTCCGAAGGCTTCGAGATCGAGGTCTCATTAGCCGAAGAAGACCCTGGCCAATCC

ACGTGGCCTGCTTGACGAACGTCAGCGACGGAGACATCGTAAATTGGTTCGCGGGCATCGCGATAAGTCC

TCTGTCCTACTACAGGTGCCGCGACAACCTTTACCAAGTCCGAACTATTGTCGACTACCAGATCCGCTGG

TCTGCAATATTCACCCCAGCCCACAAGCACAAATCCTCAGCGCGGAATATAATCCCAAAGTACTCCAAAG

ACTTAAATATAGTAAATCAAGAAGGTGGCAAGACCCTTGCAGAGTTCCCCAACAGCATAGAGCTTGGGAA

GCTCGGACCCGGTCAAGATCC-GAACAACAAAGAGCACTCAACTACTA------------------TGGT

CCAACTACATAACTTTTTCTTTTTCATTACTTCCATGGTCGTGCCTTGTGGCACGGCAGCACCCGTACTA

TTGAAATGGTTCGTCAGTAGAGATGTTCCCACAGGTGCCCCTTTTTCCAATGGTACTTTAATTCCTATTC

TTATTCCTTCATTCCTTCTTTTGGTTTATCTACATTCCAGGAAATTCATACGCTCTATGGACGGAGTAAA

AAGTGGAGTCTTGGTCAGAGCAAGTTGCCCTATTTTAT------TACCAGACATAATTGGGAGAAGCTCA

TCCGAAACTAGAGCTAGAAACGCCTTATTTCTTTTCGTTCCCATTCTTCATTTTCTTCTTCTCGAATTCA

A------GGGGGACTTACCCTATTTAGAATCTTTTTGCGGTGTGCTCCGTTTACTATTCTTTCGTACTTT

CTTCTCTTTACCACGCGATAGGTCAGCGAAGCGTGAGCGGGCGCGGAGAAGAAAAGGCCAAACACTTCGG

CCTAAC------GGGAATGAGCAACGACGAAATGAAAAGAAAAAGTGCCCCGGGCGCCCCCATT------

TAGA---AAGAAGGGTCGAAGGGTTTGGGCCTGTAGCTTTCCCCGTCCCCCCTTCGTCGGGTGGTGCTTG

CATGGGGGGTGTGCTACCTGAAATCGGGCTTGAAGCCCCCGCCTTACCAACGAGCCGACAGCTGATGGCT

GTTGGTCGCGACTACTACCAAAAAGTGAACATGAAGATGAATATTTCACATGGGGGAGTGTGCATCTTTA

TGTTGGGTGTTCTTCTGT-CGTACATAGCTGTTCCAGCTGAAATACTTGGAATAATTCTACCACTTCTAC

TAGGAGTAGCCTTTTTAGTGCTAGCTGAACGTAAAGTAATGGCTTTTGTGCAACGTCGAAAGGGTCCTGA

TGTAGTGGGATCGTTCGGATTGTTACAACCTCTAGCAGATGGTTCGAAATTGATTCTAAAAGAACCTATT

TCACCAAGTAGTGCTAATTTCTCCCTTTTTAGAATGGCTCCAGTCACTACATTTATGCTAAGTCTGGTTG

CTCGGGCCGTTGTACCTTTTGATTATGGTATGGTATTGTCAGATCCGAACATAGGGCTACTTTATTTGTT

TGCCATATCTTCGCTAGGTGTTTATGGAATTATTATAGCAGGTTGGTCTAGTATTTATTATATACGTTTA

GTGAAAAGAATGTTTTTTGATACACCTAGGACATGGATTCTATATGAACCAATGGATCGTGACAAGTCGT

TACTACTAGCAATGACTTCCTCTTTCATTACTTCATCCTTTCCATATCCTTCTCCCTTGTTCTCAGTTAC

TCATCAAATGGCACTCAGTTTATATCTTTA--TGTCAGAATTTGCGCCTATTTGTATCTATTTAGTGATC

AGTCTGCTAGTTTCTTTGATCCCACTCGGTGTTCCTTTTCCATTTTCTTCTAATACTTCGACTTATCCAG

AAAAATTGTCGGCCTACGAATGTGGTTTCGATCCTTTCGGTGATGCCAGAAGTCGTTTCGATATACGATT

TTATCTTGTTTCAATTTTATTTATTATCCTTGATCCGGAAGTAACCTTTTTCTTTCCTTGGGCAGTACCT

CTCAACAAGATTGATCCGTTTGGATCTTGGTCCATGATGGCCTTTTTATTGATTTTAACGATAGGATTTC

TCTATGAATGGAAAAGGGGTGCTTCGGATCGGGAGTAAAAAAGTGTTTATTACGATTACGCCCAACAGCC

CACTTGAGCAATTTTCCATTCTCCCATTGATTCCTATGAAAATAGGAAACTTGTATTTCTCATTCACAAA

TCCATCTTTGTTTATGCTGCTAACTCTCAGTTTGGTCCTACTTCTGCTTCATTTTGTTACTAAAAAGGGA

GGAGGAAACTCAGTACCAAATGTTTGGCAATCCTTGGTAGAGCTTATTTATGATTTCGTGCTGAACCTGG

TAAACGAACAAATAGGTGGTCTTTCCGGAAATGTTAAACAAAAGTTTTTCCCTTGCATCTTGGTCACTTT

TACTTTTTTGTTATTTCGTAATCTCCAGGGTATGATACCCTATAGCTTTACAGTTACAAGTCATTTTCTC

ATTACTTTGGGTCTTTCATTTTCCATTTTTATTGGCATTACTATAGTGGGATTTCAAAGAAATGGGCTTC

ATTTTTTAAGCTTCTCATTACCTGCAGGAGTCCCGCTGCCGTTAGCACCTTTTTTAGTACTCCTTGAGCT

AATCCCTCATTGTTTTCGCGCATTAAGCTCAGGAATACGTTTATTTGCTAATATGATGGCCGGTCATAGT

TCAGTAAAGATTTTAAGTGGGTTCGCTTGGACTATGCTATGTATGAATGATCTTTTATATTTCATAGGAG

ATCTTGGTCCTTTATTTATAGTTCTTGCATTAACCGGTCTTGAATTAGGTGTAGCTATATTACAAGCTCA

TGTTTTTACGATCTTAATCTGTATTTACTTGAATGATGCTATAAATCTCCATCAA---------------

----------------ATATGTGGGCACCTGATATCTATGAGGGTTCACCCACCCCGGTTACAGCATTCT

TTTCTATTGCACCTAAAATTTCTATTTCTGCTAATATTTTACGTGTTTTTATTTATGGTTCCTATGGAGC

TACATTGCAACAAATCTTCTTTTTCTGCAGCATTGCTTCTATGATCTTAGGAGCACTGGCCGCCATGGCC

CAAACGAAAGTAAAAAGACTTCTAGCTCATAGTTCAATTGGACATGTAGGTTATATTCGTACTGGTTTCT

CATGTGGAACCATAGAAGGAATTCAATCACTACTAATTGGTCTCTTTATTTATGCATCAATGACGATAGA

TGCATTCGCTATAGTTTCAGCATTACGGCAAACACGTGTCAAATATATAGCGGATTTGGGCGCTCTAGCC

AAAACGAATCCTATTTCGGCTATTACCTTCTCTATTACTATGTTCTCATACGCAGGAATACCCCCGTTAG

CCGGCTTTTGTAGTAAATTCTATTTGTTCTTCGCCGCTTTGGGTTGTGGGGCTTACTTCCTAGCCCCAGT

GGGAGTAGTGACTAGCGTTATAGGTTGT-TGTTCGATAGCCCGACCGTAGTGATGTTAATTGTGGTTACA

TTCATAAGTAGCTTGGTCCATCTTTATTCTATTTCATATATGTCTGAGGATCCGCATAGCCCTCGATTTA

TGTGTTATTTATCCATTCTTACTTTTTTTATGCCAATGTTGGTGACTGGAGATAACTCTCTTCAATTATT

CTTGGGATGGGAGGGAGTAGGTCTTGCTTCATATTTGTTAATTCATTTTTGGTTTACACGACTTCAGGCA

GATAAAGCAGCTATAAAAGCTATGCTTGTCAATCGAGTAGGTGATTTTGGATTAGCTCTTGGGATTTCGG

GTTGTTTTACTCTCTTTCAAACAGTAGACTTTTCTACCATTTTTGCTTGTGCTAGTGCCCCTAGAAATTC

TTGGATTTTTTGCAATATGAGATTGAATGCCATAACTCTTATTTGTATTTTACTTTTTATCGGTGCTGTT

GGAAAATCTGCACAGATAGGATCGCATACTTGGTCACCCGATGCTATGGAGGGTCCCACTCCAGTATCCG

CTTTGATTCATGCAGCTACTATGGTAACAGCTGGCGTTTTCATGATAGCAAGGTGTTCCCCTTTATTTGA

ATACCCACCTACGGCTTTAATTGTTATTACTTTTGCAGGAGCTATGACGTCATTCCTTGCGGCAACCACT

GGAATATTACAGAACGATCTAAAGAGGGTCATAGCTTATTCAACTTGCAGTCAATTAGGCTATATGATCT

TTGCTTGCGGCATTTCTAACTATTCGGTTAGCGTCTTTCATTTAATGAATCACGCCTTTTTCAAAGCATT

ACTATTCCTGAGTGCAGGTTCGGTGATTCATGCCATGTCGGATGAGCAAGATATGCGGAAGATGGGGGGG

CTCGCCTCCTCGTTCCCTTTTACCTATGCCATGATGCTCATGGGCAGCTTATCTCTAATTGGATTTCCTT

TTCTAACTGGATTTTATTCCAAAGATGTGATCTTAGAGCTCGCTTACACTAAGTATACCATCAGTGGGAA

CTTTGCTTTCTGGTTGGGAAGTGTCTCTGTCCTTTTCACTTCTTATTACTCTTTTCGTTTACTTTTTCTA

ACATTTCTAGTACCAACTAATTCATTCGGGCGAGACATCTTACGATGTCATGATGCGCCCATTCCTATGG

CCATTCCTTTAATACTTCTGGCTTTCGGGAGTCTCTTTGTAGGATACTTGGCCAA--TAACACAAAGAAG

ATACAGTTCACTCAACGATTGCCTTTGGGTTCCGAACTCCATATGGGGAAGGAACGTTGTTGTTTGCGGG

GTCTCGATCATTTACATGGACCCACTTTTCATTCCATTTGTGGGAATTTGATGATCTATAAACCGTCCTT

AACGAACGATCGGCTCATCTT------TGAGCATGATGAATCACTTCGTGCCGACCTGTTGTCAATAAAC

TTTTGGGCCTCATATGAGAATGGAAAACTGGAGCATTTTCTGCATCGGTGGATGAAGAATCGCGAACATC

CAAATTTCTGGTTAAGCATGTTCCCAGAAAAAAGATACTTTCGAGAAACAACGAGCACGACTGAAGTGGC

TATCCATACAAATCCATTTACGGATCTATATGCTTCGATTGGAACTGGAAGTTCAAGAACAGGCGGCTGG

TATACTACCATAATGAAACTGCCTTTTCTTTTTTTTATTCGGATAGGATTTCTGTTGGCTTCGTTGGGAG

GCTCGCGTAGTTTGTTACGTCAACTCCAAAAGGAGAAATTGCGTTGGAATTGAGAAAGTTACGTAA----

AGTTCATAATTGTATAACCAATTTTTGGGCCAATTCCCTCTTCGTACTACCAAAAAATGAGATTCTTGCC

GAATCCGAGTTTGCTGCTCCAACCATTACCAAACTAATACCTATTCTGTTTAGTACTTCAGGTGCTTCTG

TTGCGTATAATGTAAATCCCGTAGCGGATCAATTCC---------------AACGAGCCTTTCAAACTAG

TACTTTTTGTAATCGACTCTATAGCTTCTTCAATAAACGCTGGTTCTTCGATCAAGTTTTGAATGACTTT

ATAGTCAGATCATTCTTGCGTTTCGGGTATGAAGTCTCATTCGAAGCTTTAGACAAAGGTGCTATTGAGA

TATTGGGCCCCTATGGTATCTCGTACACATTACGACGATTGGCCGAGCGAATAAGTCAACTTCAAAGTGG

ATTTGT-TTGTTCCATGATCTATGGGTCTACAGGAGCTACCCATTTCGATCAATTAGCCAAGATTTTGAC

CGGATACGAAATCACTGGTGTTCGATCTAGTGGTATTTTTATGGGGATTCTTTTTATCGCTGTAGGATCC

CTATTCAAGATCACTGCAGTTCCTTTT-TGGTCTATGCACATCGCTTTCTCCAGGAGGTTGGCCGCCTAT

CCTAGATCTTCCCATTTCCAAGAGGATCCCGGGCTCGATCTGGTTTAGTATCAAGGTGATTCTCTTTCTC

TTTCTATATATATGGGTCCGTGCAGCATTTCCACGATATCGTTATGATCAATTAATGGGACTTGGCCGGA

AAGTGTTCTTGCCTCTATCATTAGCTCGGGTAGTCGCCGTTTCTGGTGTTTTAGTCACCTTTCAATGGCT

CCCTTA-ATGCCTCAACTGGATCAATTTACTTATTTCACACAATTCTTCTGGTTATGCCTCTTCTTCTTT

ACTTTCTATATTCTAATATGCAATGATAGGTATGGAGTACTTGGGATCAGCAGAATTCTAAAACTACGAA

ATCAACTGCTTTCACACCGGGGGAACAACATCCAAA------GCAAGGATCCCAAGAGTTTGGAAGATAT

CTTGAGAAAAGGTTTTAACACAGGTGTATCCTATATGTACTCTAGTTTATTCGAAGTATCCCAATGGTGT

AAGGCCGCCGACTTATTTGGAAAAAGGAAGAAAATCACTTTGATCTCTTGTTTCGGAGAAATAAGTGGCT

CACGAGGAATGGAAAGAAACATATTCTATTTGATCTCGAAGTCTTCATATAGCACTTC------------

---TTCCAATCCTGGATGGGTGATCACTTGTAAGAATGACATAATGCTAATCCATGTTCTACACGGCCAA

GGA--------------------

>Spinacia oleracea NC035618.1

-TGATACTTTCTGTTTTGTCGAGCCCGGCTTTGGTCTCTGGTTTGATGGTTGTACGTGCTAAAAATCCGG

TACATTCCGTTTTGTTTCCCATCCCAGTCTTTCGCAACACTTCAGGTTTACTTCTTTTGTTAGGTCTCGA

TTTTTCCGCCATGATCTTCCCAGTAGTTTATATAGGAGCTATAGCCGTTTCATTCCTATTCGTTGTTATG

ATGTTCCATATTCAAATAGCGGAGATTCACGAAGAAGTATTGCGCTATTTACCAGTGAGTGGTATTATTG

GACTGATCTTTTGGTGGGAAATGTTCTTCATTTTAGATAATGAAACCATTCCATTACTACCAACCCAAAG

AAATACGACCTCTCTGAGATATACGGTTTATGCCGGAAAGGTACGAAGTTGGACTAATTTGGAAACATTG

GGCAATTTACTTTATACTTACTATTTTGTCTGGTTTTTGGTTTCTAGTCTTATTTTATTAGTAGCCATGA

TTGGGGCTATAGTACTGACTATGCATAGGACTACTAA------GGTGAAAAGACAGGATGTATTCCGACG

AAATGCTATTGATTCTAGAAGGACTATAATGAAGAGGACGACAGACC-ATGTCAATATATGAATTGTTTC

ATTATTCGTTATTTCCGGGTCTTTTCATTGCATTCACTTACAACAAGAAACAACCACCAGCGTTTGGTGC

AGCACCCGCATTTTTGTGTATTCTTCTTTCTTTCCTTGGTCTTTTGTTCTGTCATATTCCTAATAACTTA

TCCAATTACAACGTATTAACCGCTAATGCACCTTTCTTTTATCAAATCTCAGGGACATGGTCTAATCATG

AAGGTAGTATTTTATTATGGTGTCGGATCCCAAGTTTTTATGGATTCCTTCTTTGTTACCGGGGTCGATC

CCAAAGCCATAATGTCTCAAAACGAGGAGGCCATAGAGAAAGTCTTCTTTTTTCCTTTGTCTTAAACTTC

GTGAAGAACTCCATTCTATCTCTTCCTCGTTACGAAAAAAAAAGTAGAGTTCTTCACGAACCCCAGTTGT

ACACTCTCTTCGTTCTACGAA---CTCTTGTTGATTCAGAACTTTGTTCGCGAAGGAACCGGACTTTTGA

CGGGCCAGTTCTTTTTTACGCGCCGCTTTACCCTGAAAGGAAAATGAGCTTTGCTCTTCTGGGCGCTAGG

CGCTCTCGTGGTTCGCGGGAAGGAAAAAGGACTCATCCTTTGTTGCATCTGGCACGAGATGATAAAGAGA

GAGCTTCGTCTATCGATGAACAGCGGATTGACGGAGCTCTTGGCATTGCTTTCTTTTTCTTTCCTTTCCT

ATCAGCGAGTTCCGATCCTTTTGTTCGAAATTTCTTCGTTCGTACCGAACCGCTTGCAGAATCAAATCCT

GTTCCACAAGATCCTATATCAGCTATACATCCTCCTTGCATTTATGCCGGAGACGTCGCCAGTGCTATGG

GCTTTGCCTTATGTAGATCAAAAATGATGAATGGGATTGTGGCACTCCACTCGCCGCCAATGCGGAAGGA

TGTCGCCGAAAAGAATGGAACGCTGCTTTGCTCTGCTGGATGCGTCGGATCCCGTATAACAAGCGAGCTC

TTTACCCTTAAATTCAAAGATGTGGGCGCCAAATGCTATCCTGCTCTATTGTTGCGTAGCAAAAGAAGCC

TGCTC---ATGCTGCTTCGGCGGCGCTTTTTCGCCTTCTCTTCGCTCTGGACAAGAGCGCTAGTGGACAC

GGGGAGGGAGCG------GGCGAAGCGTTTCT------TTCGTAATGGAAAGACAAAGACCACTACTTTG

CCTCTTTGTTGGACCGCCGGCGCGAACACAGTGGTCTCTGACCAGGACCAGGAACCAATTCGAATTTGGA

TCTTGACATGTCGGTGCTTTTTAACCGTAGGCATCTTGCCAGGAAGTTGGTGGGCTCATCATGAATTAGG

TCGGGGTGGCTGGTGGTTTCGGGATCCCGTAGAAAATGCTTCTTTTATGCCTCGGGTATTAGCCACAGCT

CGTATTCATTCAGTCATTTTACCCCTTCTTCATTCTTGGACTTTGCTTCTTAATATTGTGACTTTTCTAT

GCTGTGTCTTAGGAACCTTTTCAATACGGTCCGGATTGCTAGCTCCCGTTCATAGTTTTGCTACAGATGA

TACACGAGGAATCTTTTTATGGCGGTTCTTCCTTCTAATGACCGGCATATCTATGATTCTTTTCTCTCAG

ATGAAGCAGCAGGCATCGGTCCGTAGAACCTATAAAAAAGAGATGGTTGTAGCGCGAAGTACTCTTGTGC

ACTTACG----TGATTGTTCGAGAATGGCTATTCTTCACAATTGCTCCTTGTGATGCAGCGGAACCATGG

CAATTAGGATTTCAAGACGCAGCAACACCTATGATGCAAGGAATAATAGACTTACATCATGATATCTTTT

TCTTCCTCATTCTTATTTTGGTTTTCGTATCATGGATCTTGGTTCGCGCTTTATGGCATTTCCACTATAA

AAAAAATCCAATCCCGCAAAGGATTGTTCATGGAACCACTATTGAGATTATTCGGACCATATTTCCCAGT

ATCATCCTGATGTTCATTGCTATACCATCATTTGCTCTGTTATACTCAATGGACGAGGTAGTAGTAGATC

CAGCCATTACTATCAAAGCTATTGGACATCAATGGTATCGGAGT--------------------------

----------------------------------------------------------------------

----------------------------------------------------------------------

----------------------------------------------------------------------

----------------------------------------------------------------------

----------------------------------------------------------------------

------------------------TGAGACGACTCTTTTTTGAACTATATCATAAACAGATCTTCTTCTC

CACACCAATCACGAGTTTTTCTCCATTCCTCTCGTATATTGTCGTAACGCCCTTAATGCTAGGTTTTGAA

AAAGACTTTTCATGTCATTTCCATTTAGGTCCGATTCGGATCCCTCTGTTGTTTCCTTTTCCTCCCGCAC

CTTTTCTTCGAAATGAGAAAGAAGATGGTACACTCGAATTGTATTATTTAAGTGCTTATTGCTTGCCAAA

GATCCTACTTCTACAATTGGTAGGTCACCGGGTTATTCAAATAAGTCGTGTTTTCTGTAGTTTTCCCATG

TTACAACTTCTGTACCAATTCGGCCAATCCGGAATGGATCGGTTAAACATTCTATTAGGGAGCCTGGTCT

TGACTCTTCTGTGCGGTATTCATTCTTGTTTGGCTCTTGGAATCACATCCAGCAGTGGTTGGAACAGCTC

GCAAAATTTAACCACTTCACCTACTTCATTGCCCTCAACCGTTTCTCGTACCTCTATTGAAACAGAATGG

TTTCATGTTCTTTCATCGATTGGTTATTTTTCTTCGTTCGTATCTCTTTTTCCAATTTCGGTCTCGATTA

GTTCACAAGATTGA--------TTCTTTATTATAACCTTC-----TTTTTTGATGTCAAAGACCAGGAAC

TACGCGCAAATTCTCATTGGATCTTGGTTGTTCTTAACAGCGATGGCTATTCATTTAAGTCTTTGGGTAG

CACCACTAGATTTTCAACAAGGTGGAAATTCTCGTATTCTCTATGTACATGTTCCTGTGGCTCGGATGAG

TATTCTTGTTTATATCGTTACGGCTATAAACACTTTCTTGTTCCTATTAACAAAACATCCTCTTTTTCTT

CGCTCTTCCGGAACCGGTACAGAAATGGGTGCTTTTTCTACACTGTTTACTTTAGTTACTGGGGGGTTTC

GGGGAAGACCCATGTGGGGCACCTTTTGGGTGTGGGATGCTCGTTTAACTTCTGTATTCATCTCGTTCCT

TATTTACTTGGGTGCGCTGTGTTTTCAAAAGCTTCCTGTAGAACCGGCTCCTATTTCAATCCGTGCTGGA

CCGATCGATATACCAATAATCAAGTTTCCAGTCAACTGGTGGAATACATCGCATCAACCTGGGAGCATTA

GCCGATCTGGTACATCAATACATGTTCCTATGCTCATTCCAATCTTGTCTAACTTTGCTAACTTCCTCTT

CTCAACCCGTATCTTCTTTGTTCTGGAAATACGTCTTCCTATTCCATCTTTTCTCGAATCTCCTTTAACG

GAAGAAATAGAAGCTCGAGAAGGAA----TGCAGGCTAGAAAGATGCTATTTGCTGCTATTCTATCTATT

TGTGCATCAAGTTCGAAGAATATCTCAATCTATAATGAAGAAATGATAGTAGCTCGTTGTTTTATAGGCT

TTATCATATTCAGTCGGAAGAGTTTAGGTAAGACTTTCAAAGTGACTCTCGACGAGAGAATCCAGGCTAT

TCAGGAAGAATCGCAGCAATTCCCCAATCCTAACGAAGTAGTTCCTCCGGAATCCAATGAACAACAACGA

TTACTTAGGGTCAGCTTGAAAATTTGTGGAACCGTAGTAGAATCATTACCAATGGCACGCTGTGCGCCTA

AGTGCGAAAAAACAGTGCAAGCTTTGTTATGTCGAAACCTAAATGTTAAGTCAGCAACACTTCCAAATGC

CACTTCTTCCCGTCGCACCCGTCTTCAGGACGATCTAGTCACAGGGTTTCACTTCTCAGTGAGTGAAAGA

TTTGTCCCCGGGTCTACGCTGAAAGCTTCTATAGTAGAACTCATTCGAGAAGGCTTGTCGGTCTTAAGAA

TGGTTCGGGTAGGAGGTTCTCT--ATGAAAGAGGCGATCAGAATGGTACCCGAATCCATTTACGATCCCG

AGTTTCCAGACACATCGCACTTCCGCTCGGGTCGAGGCTGCCATTCGGCCCTAAGACGGATCAAAGAAGA

GTGGGGAACCTCTCGCTGGTTTTTGGAATTCGACATCAGGAAGTGTTTTCACACCATCGACCGACATCGA

CTCATCCCAATCTTTAAGGAAGAGATCGACGATCCCAAGTTCTTTTACTCCATTCAGAAAGTCTTTTCTG

CCGGACGGCTCGTAGGAGGTGAGAAGGGCCCTTACTCCGTCCCACACAGTGTACTACTATCGGCCCTACC

AGGCAACATCTATTTACACAAGCTCGATCAGGAGATAGGGAGGATCCGACAGAAGTACGAAATTCCGATT

GTTCAGAGAATCAGATCGGTTCTATTAAAGACAAGTCGTATTGATGACCAAGAAAACTCTGGAGAAGAA-

--ATGGAATTTTCTCCCAGAGCTGCGGAACTAACGACTCTATTAGAAAGTAGAATTAGCAACTTTTACAC

GAATTTTCAAGTGGATGAGATCGGTCGAGTGGTTTCAGTTGGGGATGGGATTGCACGTGTTTATGGATTG

AACGAGATTCAAGCTGGGGAAATGGTGGAATTTGCCAGCGGTGTGAAAGGAATAGCCTTAAATCTTGAGA

ATGAGAATGTAGGGATTGTTGTCTTTGGTAGTGATACCGCTATTAAAGAGGGAGATCTTGTCAAGCGCAC

TGGATCTATTGTGGATGTTCCTGCGGGAAAGGCTATGCTAGGGCGTGTGGTCGACGGGTTGGGAGTACCT

ATTGATGGAAGAGGGGCGCTAAGCGATCACGAGCGTCGACGTGTCGAAGTGAAAGCCCCCGGGATTATTG

AACGTAAATCTGTGCACGAGCCTATGCAAACCGGGTTAAAGGCGGTAGATAGCCTGGTTCCTATAGGCCG

TGGTCAACGAGAACTTATAATCGGGGACCGACAAACGGGAAAAACAGCTATTGCTATCGATACCATATTA

AACCAAAAGCAACTGAACTCAAAGGCCACCTCTGAGAGTGAGATATTGTATTGTGTCTATGTAGCGGTTG

GACAGAAACGTTCAACTGTGGCACAATTAGTTCAAATTCTTTCAGAAGCGAATGCTTTGGAATATTCCAT

TCTTGTAGCAGCCACCGCTTCGGATCCTGCTCCTCTTCAATTTCTGGCCCCATATTCTGGGTGTGCTATG

GGAGAATATTTCCGCGATAATGGAATGCACGCATTAATCATCTATGATGATCTTAGTAAACAGGCGGTGG

CATATCGACAAATGTCATTATTATTACGCCGACCACCAGGCCGTGAGGCTTTCCCAGGCGACGTTTTCTA

TTTACATTCCCGTCTCTTAGAAAGAGCCGCTAAACGATCGGACCAGACAGGTGCCGGTAGCTTGACCGCC

TTACCCGTCATTGAAACACAAGCTGGAGACGTATCGGCCTATATTCCCACCAATGTGATCTCCATTACTG

ATGGACAAATCTGTTTGGAAACAGAGCTCTTTTATCGCGGAATTAGACCTGCTATTAACGTCGGCTTATC

TGTCAGTCGCGTCGGGTCTGCCGCTCAGTTGAAAGCTATGAAACAAGTCTGCGGGAGTCTAAAACTGGAA

TTGGCACAATATCGCGAAGTGGCCGCCTTTGCTCAATTTGGGTCAGACCTTGATGCTGCGACTCAGGCAT

TACTCAATAGAGGTGCAAGGCTTACAGAAGTACTGAAACAACCACAATATGCACCACTTCCAATTGAAAA

ACAAATTCTAGTCATTTACGCAGCTGTCAATGGATTCTGTGATCGAATGCCACTAGATAAAATTTCTCAA

TATGAGAGAACCATTCCAAATAGTGTAAAACCAGAATTATTACAATCCCT------AAAGGGGGGGTTAA

CCAACGATAAAAAGAAGGAACTAGACGCATTCTTAAAAGAATGCG-------------------------

-------GCATCCAACGCAAAGCGGCCTTTCATTCCCTTGTTTCGTCGTGGCACACCCTCCCCGCAAGCA

CCCCCCGGCTCAGGGGGGACCAGAAAAGGCCCTTCGTTTTCCCCCCTTCGTCGGCCCTTGCCGCCTTCCT

TAACAAGCCCTCGAGCCTCCTTTTCGCTGCGTTCCTCATAGAAGCCGCCGGGTTGACCCCGAAGGCCGAA

TTCTATGGTATAGAATGCTGTAATAATAATTGGGCCATGAGAGACCTTTTTAAGTATTGCAAAAGAAAGG

GCCTGCTGATAGAGCTGGGCGAGGCAGCGATACTAGTTATCAGGTCAGAGAAAGGCCTGGCCCGTAAGCT

GGCCCCCTTAAAAACCCATTACTTAATAAGTATTTGTTACGCGCGATATGCCGACGACTTACTATTGGGA

ATCGTGGGTGCCGTAGAGCTTCTCATAGAAATACAAAAACGTATTGCCCACTTCCTACAATCCGGCCTGA

ACCTTTGGGTAGGCTCTGCGGGATCAACAACAATAGCTGCACGGAGTACGGTAGAATTCCTCGGTACGGT

CATTCGGGAAGTCCCCCCGAAGACGACTCCCATACAATTCTTGCGAGAGCTGGAGAAGCGTCTACGGGTA

AAGCACCGTATCCATATAACTGCTTGCCACTTACGCTCTGCCATTCATTCCAAGTTTAGGAACCTAGGTA

ATAGTATCCCGATCAAACAGCTGACGAAGGGGATGAGCAAAACAGGGAGTCTACTGGACGCGGTTCAACT

AGCGGAAAGTCTTTCCACAGCTAGAGTAAGAAGTCCCCAAGTGAGCGTATTATGGGAGACCGTCAAGCAC

ATCCGGCAAGGATCAAGGGAGATCTCGTTGTTGCATAGCTCAGGTCAGAGCAAGGTGCCATCGGACGTTC

AACAGGCAGTCTCGCGATCGGGCATGAGTGTCCGGAAGTTGTC---ATTGTATACTCTCGCGGGTCGGAA

GGCGGCGGGGGAAGGAGGGGGACACTGGTCGAGATCTATCAGCAGCGAATTCCCCATACAGATAGAAGCG

CCTATCAAAAAGATACTCCGAAGGCTTCGAGATCGAGGTCTCATTAGCCGAAGAAGACCCTGGCCAATCC

ACGTGGCCTGCTTGACGAACGTCAGCGACGGAGACATCGTAAATTGGTTCGCGGGCATCGCGATAAGTCC

TCTGTCCTACTACAGGTGCCGCGACAACCTTTACCAAGTCCGAACGATTGTCGACTACCAGATCCGCTGG

TCTGCAATATTCACCCCAGCCCACAAGCACAAATCCTCAGCGCGGAATATAATCCCAAAGTACTCCAAAG

ACTTAAATATAGTAAATAAAGAAGGTGGCAAGACCCTTGCAGAGTTCCCCAACAGCATAGAGCTCGGGAA

GCTCGGACCCGGTCAAGATCC-GAACAACAAAGAGCACTCAACTACT------------------ATGGT

AAAACTCCAGAACTTTTTCTTTTTCATTACTTCCATGGTCGTGCCTTGTGGCACGGCAGCACCCGTACTA

TTGAAATGGTTCGTCAGTAGAGATGTTCCCACAGGTGCCCCTTTTTCCAATGGTACTTTAATTCCTATTC

TTATCCCTTCATTCCTTCTTTTGGTTTATCTACATTCCAGGAAATTCATACGCTCTATGGACGGAGTCAA

AAGTGGAGTCTTGGTCAGAGCAAGTTGCCCTATTTTAT------TACCAGACATAATTGGGAGAAGCTCA

TCCGAAACTAGAGCTAGAAACGCCTTATTTCGTTTCGTTCCCATTCTTCATTTTCTTCTTCTCGAATTAA

A------GGGGGACTTACCCTATTTAGAATCTTTTTGCGGTGTGCTCCGTTTACTATTCTTTCGTACTTT

CTTCTCTTTACCACGCGATAGGTCAGCGAAGCGTGAGCGGGCGCGGAGAAGAAAAGGCCAAACACTTCGG

CCTAAC------GGGAATGAGCAACGACGAAATGAAAAGAGAAAGTGCCCCGGGCGCCCCCATT------

TAGA---AAGAAGGGTCGAAGGGTTTGGGCCTGTAGCTTTCCCCGTCCCCCCTTCGTCGGGTGGTGCTTG

CATGGGGGGTGTGCTACCTGAAATCGGGCTTGAAGCTCCCGCCTTACCAACGAGCCGACAGCTGATGGCT

GTTGGTCGCGACTACTACCAAAAAGTGAACATGAAGATGAATATTTCACATGGGGGAGTGTGCATCTTTA

TGTTGGGTGTTCTTCTG--CGTACATAGCTGTTCCAGCTGAAATACTTGGAATAATTCTACCACTTCTAC

TAGGAGTAGCCTTTTTAGTGCTAGCTGAACGTAAAGTAATGGCTTTTGTGCAACGTCGAAAAGGTCCTGA

TGTAGTGGGATCGTTCGGATTGTTACAACCTCTAGCAGATGGTTCGAAATTGATGCTAAAAGAACCTATT

TCACCAAGTAGTGCTAATTTCTCCCTTTTTAGAATGGCTCCAGTCACTACATTTATGCTAAGTCTGGTTG

CTCGGGCCGTTGTACCTTTTGATTATGGTATGGTATTGTCAGATCCGAACATAGGGCTACTTTATTTGTT

TGCCATATCTTCGCTAGGTGTTTATGGAATTATTATAGCAGGTTGGTCTAGTATTTATTATATACGTTTA

GTGAAAAGAATGTTTTTTGATACACCTAGGACATGGATTCTATATGAACCAATGGATCGTGACAAGTCGT

TACTACTAGCAATTACTTCCTCTTTCATTACTTCATCCTTTCCATATCCTTCTCCCTTGTTCTCAGTTAC

TCATCAAATGGCACTCAGTTTATATCTTTA--TGTCAGAATTTGCGCCTATTTGTATCTATTTAGTGATC

AGTCTGCTAGTTTCTTTGATCCCACTCGGTGTTCCTTTTCCATTTTCTTCTAATACTTCGACTTATCCAG

AAAAATTGTCGGCCTACGAATGTGGTTTCGATCCTTTCGGTGATGCCAGAAGTCGTTTCGATATACGATT

TTATCTTGTTTCAATTTTATTTATTATCCTTGATCCGGAAGTCACCTTTTTCTTTCCTTGGGCAGTACCT

CTCAACAAGATTGATCCGTTTGGATCTTGGTCCATGATGGCCTTTTTATTGATTTTAACGATAGGATTTC

TCTATGAATGGAAAAGGGGTGCTTCGGATCGGGAGTAAAAAAGTGTTTATTACGATTACGGCCTTCAGCC

CACTTGAGCAATTTTCCATTCTCCCATTGATTCCTATGAAAATAGGAAACTTGTATTTCTCATTCACAAA

TCCATCTTTGTTTATGCTGCTAACTCTCAGTTTGGTCCTACTTCTGCTTCATTTTGTTACTAAAAAGGGA

GGAGGAAACTCAGTACCAAATGTTTGGCAATCCTTGGTAGAGCTTATTTATGATTTCGTGCTGAACCTGG

TAAACGAACAAATAGGGGGTCTTTCCGGAAATGTGAAACAAAAGTTTTTCCCTTGCATCTTGGTCACTTT

TACTTTTTTGTTATTTCGTAATCTCCAGGGTATGATACCCTATAGCTTTACAGTTACAAGTCATTTTCTC

ATTACTTTGGGTCTTTCATTTTCCATTTTTATTGGCATTACTATAGTGGGATTTCAAAGAAATGGTCTTC

ATTTTTTAAGCTTCTCATTACCTGCAGGAGTCCCACTGCCGTTAGCACCTTTTTTAGTACTCCTTGAGCT

AATCCCTCATTGTTTTCGCGCATTAAGCTCAGGAATACGTTTATTTGCTAATATGATGGCCGGTCATAGT

TCAGTAAAGATTTTAAGTGGGTTCGCTTGGACTATGCTATGTATGAATGATCTTTTATATTTCATAGGAG

ATCTTGGTCCTTTATTTATAGTTCTTGCATTAACCGGTCTTGAATTAGGTGTAGCTATATTACAAGCTCA

TGTTTTTACGATCTTAATCTGTATTTACTTGAATGATGCTATAAATCTCCATCAA---------------

----------------ATATGTGGGCACCTGATATCTATGAGGGTTCACCCACCCCGGTTACAGCATTCT

TTTCTATTGCGCCTAAAATATCTATTTCTGCTAATATTTTACGTGTTTTTATTTATGGTTCCTATGGAGC

TACATTGCAACAAATCTTCTTTTTCTGCAGCATTGCTTCTATGATCTTAGGAGCACTGGCCGCCATGGCC

CAAACGAAAGTAAAAAGACTTCTAGCTCATAGTTCAATTGGACATGTAGGTTATATTCGTACAGGTTTCT

CATGTGGAACCATAGAAGGAATTCAATCACTACTAATTGGTCTCTTTATTTATGCATCAATGACGATAGA

TGCATTCGCTATAGTTTCAGCATTACGGCAAACACGTGTCAAATATATAGCGGATTTGGGCGCTCTAGCC

AAAACGAATCCTATTTCGGCTATTACCTTCTCTATTACTATGTTCTCATACGCAGGAATACCCCCGTTAG

CCGGCTTTTGTAGTAAATTCTATTTGTTCTTCGCCGCTTTGGGTTGTGGGGCTTACTTCCTAGCCCCAGT

GGGAATAGTGACTAGCGTTATAGGTTGT--GTTCGATAGCCCGACCGTAGTAATGTTAATTGTGGTTACA

TTCATAAGTAGCTTGGTCCATCTTTATTCTATTTCATATATGTCTGAGGATCCGCATAGCCCTCGATTTA

TGTGTTATTTATCCATTCTTACTTTTTTTATGCCAATGTTGGTGACTGGAGATAACTCTCTTCAATTATT

CTTGGGATGGGAGGGAGTAGGTCTTGCTTCATATTTGTTAATTCATTTTTGGTTTACACGACTTCAGGCA

GATAAAGCAGCTATAAAAGCTATGCTTGTCAATCGAGTAGGTGATTTTGGATTAGCTCTTGGGATTTCGG

GTTGTTTTACTCTCTTTCAAACAGTAGACTTTTCTACCATTTTTGCTTGTGCTAGTGCCCCTAGAAATTC

TTGGATTTTTTGCAATATGAGATTGAATGCCATAACTCTTATTTGTATTTTACTTTTTATTGGTGCTGTT

GGAAAATCTGCACAGATAGGATCGCATACTTGGTCACCCGATGCTATGGAGGGTCCCACTCCTGTATCCG

CTTTGATTCATGCAGCTACTATGGTAACAGCTGGCGTTTTCATGATAGCAAGGTGTTCCCCTTTATTTGA

ATACCCACCTACGGCTTTAATTGTTATTACTTTTGCAGGAGCTATGACGTCATTCCTTGCGGCAACCACA

GGAATATTACAGAACGATCTAAAGAGGGTCATAGCTTATTCAACTTGCAGTCAATTAGGCTATATGATCT

TTGCTTGCGGCATTTCTAACTATTCGGTTAGCGTCTTTCATTTAATGAATCACGCCTTTTTCAAAGCATT

ACTATTCCTGAGTGCAGGTTCGGTGATTCATGCCATGTCGGATGAGCAAGATATGCGGAAGATGGGGGGG

CTCGCCTCCTCGTTCCCTTTTACCTATGCCATGATGCTCATGGGCAGCTTATCTCTAATTGGATTTCCTT

TTCTAACTGGATTTTATTCCAAAGATGTTATCTTAGAGCTCGCTTACACTAAGTATACCATTAGTGGGAA

CTTTGCTTTCTGGTTGGGAAGTGTATCTGTCCTTTTCACTTCTTATTACTCTTTTCGTTTACTTTTTCTA

ACATTTCTAGTACCAACTAATTCATTCGGGCGAGACATCTTACGATGTCATGATGCGCCCATTCCTATGG

CCATTCCTTTAATACTTCTGGCTTTCGGGAGTCTCTTTGTAGGATACTTGGCCAAACTAACACAAAGAAG

ATACAGTTCACTCAACGATTGCCTTTGGGTTCCGAACTCCATATGGGGAAGGAACGTTGTTGTTTGCGGG

GTCTCGATCATTTACATGGACCCACTTTTCATTCCATTTGTGGGAATTTGATGATCTATAAACCGTCCTT

AACGAATGATCGGCTCATCTT------TGAGCATGAGGAATCACTTCGTGCCGACCTGTTGTCAATAAAC

TTT------TTGCTTGAGAATGGAAAACTGGAGCATTTTCTGCATCGGTGGATGAAGAATCGCGAACATC

CAAATTTCTGGTTAAGCATGTTCCCAGAAAAAAGATACTTTCGAGAAACAACGAGCACGACTGAAGTAGC

TATCCATACAAATCCATTTACGGATCTATATGCTTCGATTGGAACTGGAAGTTCAAGAACAGGCGGCTGG

TATACTACCATAATGAAACTGCCTTTTCTTTTTTTTATTCGGATAGGATTTCTGTTGGCTTCGTTGGGAG

GCTCGCGTAGTTTGTTACGTCAACTCCAAAAGGATAAATTGCGTTGGAATTGAGAAAGTTACGTAA----

AGTTCATAATTGTATA-CCAATTTTTGGGCCAATTCCCTCTTCGTACTACCAAAAAATGAGATTCTTGCC

GAATCCGAGTTTGCTGCTCCAACCATTACCAAACTAATACCTATTCTGTTTAGTACTTCAGGTGCTTCTG

TTGCGTATAATGTAAATCCCGTAGCGGATCAATTCC---------------AACGAGCCTTTCAAACTAG

TACTTTTTGTAATCGACTCTATAGCTTCTTCAATAAACGCTGGTTCTTCGATCAAGTTTTGAATGACTTT

ATAGTCAGATCGTTCTTGCGTTTCGGGTATGAAGTCTCATTCGAAGCTTTAGACAAAGGTGCTATTGAGA

TATTGGGCCCCTATGGTATCTCGTACACATTCCGACGATTGGCCGAGCGAATAAGTCAACTTCAAAGTGG

ATTTGT-TTGTTCCATGATCTATGGGTCTACAGGAGCTACCCATTTCGATCAATTAGCCAAGATTTTGAC

CGGATACGAAATCACTGGTGTTCGATCTAGTGGTATTTTTATGGGGATTCTTTTTATCGCTGTAGGATCC

CTATTCAAGATCACTGCAGTTCCTTTT-TGGTCTATGCACATCGCTTTCTCCAGGAGGTTGGCCGCCTAT

CCTAGATCTTCCCATTTCCAATAGGATCCCGGGCTCGATCTGGTTTAGTATCAAGGTGATTCTCTTTCTC

TTTCTATATATATGGGTCCGTGCAGCATTTCCACGATATCGTTATGATCAATTAATGGGACTTGGCCGGA

AAGTGTTCTTGCCTCTATCATTAGCTCGGGTAGTCGCCGTTTCTGGTGTTTTAGTCACCTTTCAATGGCT

CCCTTA-ATGCCTCAACTGGATCAATTTACTTATTTTACACAATTCTTCTGGTCATGCCTCTTCTTCTTT

ACTTTCTATATTCTAATATGCAATGATAGGGATGGAGTACTTGGGATCAGCAGAATTCTAAAACTACGAA

ATCAACTGCTTTCGCGCCGGGGGAACAACATCCAAA------GCAAGGACCCCAAGAGTTTGGAAGATAT

CTTGAGAAAAGGTTTTCACACAGGTGTATCCTATATGTACTCTAGTTTATTCGAAGTATCCCAATGGTGT

AAGGCCGCCGACTTATTTGGAAAAAGGAAGAAAATCTCTTTGATCTCTTGTTTCGGAGAAATAAGTGGCT

CACGAGGAATGGAAAGAAACATATTCTATTTGATCTCGAAGTCTTCATATAGCACTTC------------

---TTCCAATCCTGGATGGGTGATCACTTGTAAGAATGACATAATGCTAATCCATGTTCTACACGGCCAA

GGA--------------------

>Fallopia aubertii MW664926.1

-TGATACTTTCTGTTTTGTCAAGCCCTGCTTTGGTCTCTGGTTTGATGGTTGTACGTGCTAAAAATCCGG

TACATTCCGTTTCGTTTCCCATCCCAGTCTTTCGCGACACTTCAGGTTTACTTCTTTTGTTAGGTCTCGA

CTTCTTCGCTATGATCTTCCTAGTAGTTTATATAGGAGCTATAGCCGTTTCATTCCTATTCGTTGTTATG

ATGTTCCATATTCAAATAGCGGAGATTCACGAAGAAGTATTGCGTTATTTACCAGTGAGTGGTATTATTG

GACTGATCTTTTGGTGGGAAATGTTCTTCATTTTAGATAATGAAAGCATTCCATTACTACCAACCCAAAG

AAATACGACCTCTCTGAGATATACGGTTTATGCCGGAAAGGTACGAAGTTGGACTAATTTGGAAACATTG

GGCAATTTACTTTATACATACTATTTTGTCTGGTTTTTAGTTTCTAGTCTTATTTTATTAGTAGCAATGA

TTGGGGCTATAGTACTGACTATGCATAGGACTACTAA------AGTGAAAAGACAGGATGTATTCCGACG

AAATGCTATTGATTTTAGGAGGACTATAATGAGGAGGACGACAGACCTATGTCAATATATGAATTGTTTC

ATTATTCGTTATTTCCGGGTCTTTTCATTGCATTCACTTACAACAAGAAAGAACCACCAGCGTTTGGTGC

AGCACCTGCATTTTGGTGCATTCTTCTTTCTTTCCTTGGTCTTTTGTTCTGTCATATTCCTAATAACTTA

TCCAATTACAACGTATTAACCGCTAATGCACCTTTCTTTTATCAAATCTCAGGGACATGGTCTAATCATG

AGGGTAGTATTTTATTATGGTGTCGGATCCCAAGTTTTTATGGATTCCTTCTTTGTTACCGGGGTCGATC

CCAAAGCCATAATGTCTCAAAAGGAGGAGGCCATAGAGAAACTCTTTTTTTTTCCTTTGTCTTGAACTTC

GTGAAGAACTCCATTCTATCTCTTCCTCGTTACGAACAAAAAAGTGGAGTTATTCACGAACCCCAGTTGT

ACACTCTCTTCGTTCTACGAA---CTCTTCTTGATTCTGAACTTTGTTCGCGAAGGAACCGGACTTTTGA

CGGGCCAGCTCTTTTTTACGCGCCGCTTTACCCTGAAAGGAAAATGAGCTTTGCTCTTCTGGGCGCTAGG

CGCTCTCGTGGTTCGCGAGAAGGAAAAAGGATGAGTCCTTTCTTGCATCTGGCACGAGATGATAAAGAGA

GAGCTTCGTCTATCGATGAACAGCGGATTGGCGGAGCTCTTGGCATTGCTTTCTTTTTCTTTCCTTTCCT

ATCAGCGAGTTCCGATCCTTTTGTTCGAAATTTCTTCGTTCGTACCGAACCGCTTGCAGAATCAAATCCT

GTTCCACAAGATCCTATATCAGCTATACATCCTCCTTGCATTTATGCCGGAGAAGTCGCCAGTGCTATAG

GCTTTGGCTTATGTAGATCCAAAATGATGAATGGGATTGTGGCACTCCACTCGCCGCCAATGCGGAAGGA

TGCCGCCGAAAAGAGTGGAACGCTGCTTTGCTCTGCTGGAAGCGTCGGATCCCGTATAACAAGCGAGCTC

TTTACCCTCAAATTCAAACATGTGGGCGAAAAATGCTATCCTGCTCTATTGTTGCGTAGCAATAGAAGCC

TGCTC---ATGCTGCTTCGGCGGCGCTTTTTCGCCTTCTCTTCGCTCTGGACAAGAGCGCTAGTGGACAC

GGGGAGGGAGGA------GGCGAAGTGTGTCG------TTCGTAATGGAAAGAAAGATAGCACTACTTCG

CCTCTTTGTTGGACCGCCGGCGCGAACACAGTGGTCTCTGACCAGGACCAGGAACCAATTCGAATTTGGA

TCTTGACATGTCGGTGGTTTTTAACCGTAGGCATCTTGCCAGGAAGTTGGTGGGCTCATCATGAATTAGG

TCGGGGTGGCTGGTGGTTTCGGGATCCCGTAGAAAATGCTTCTTTTATGCCTCGGGTATTAGCCACAGCT

CGTATTCATTCAGTCATTTTACCCCTTCTTCATTCTTGGACTTTCTTTCTGAATATTGTGACTTTTCTAT

GCTGTGTCTTAGGAACCTCTTCAATACGGTCCGGATTGCTAGCTCCCGTTCATAGTTTTGCTACAGATGA

TACACGAGGAATCTTTTTATGGCGGTTCTTCCTTCTAATGACCGGCATATCTATGATTCTTTTCTCTCAG

ATGAAGCAGCAGGCATCGGTCCGTAGAACCTATAAAAAAGAGATGGTTGTAGCGCG--------------

----------ATGATTGTTCTAGAATGGCTATTCTTCACAATGGCTCCTTGTGATGCAGCGGAACCATGG

CAATTAGGATTTCAAGACGCAGCAACACCTATGATGCAAGGAATAATAGACTTACATCATGATATCTTTT

TCTTCCTCATTCTGATTTTGGTTTTCGTATCATGGATCTTGGTTCGCGCTTTATGGCATTTTCACTATCA

AAAAAATCCAATCCCGCAAAGGATTGTTCATGGAACTACTATCGAGATTCTGTGGACCATATTTCCGTCT

ATCATCCTGATGTTCATTGCTATACCATCATTTGCTCTGCTATACTCAATGGACGAGGTAGTAGTAGATC

CAGCCATTACTATCAAAGCTATTGGACATCAATGGTATTGGACTTATGAGTATTCGGACTATAACAGTTC

CGATGAACAGTCACTCACTTTTGACAGTTATATGATTCCAGAAGATGATCTAGAATTGGGTCAATTACGT

TTATTAGAAGTGGACAATAGAGTGGTTGTACCAGCCAAAACTCATATACGTATTATTGTAACATCTGCTG

ATGTACTTCATAGTTGGGCTGTACCTTCCTCAGGTGTAAAATGTGATGCTGTACCTGGTCGTTTAAATCA

GACCTCTATTTTGGTACAACGAGAAGGAGTTTACTATGGGCAGTGCAGTGAAATTTGTGGAACTAATCAT

GCTTTTATGCCTATCGTCGTAGAAGCTGTTTCTAGGAAAGATTATGGTTCTCGGGTAGCCAATCAATTAA

TCCCCCAAGCCGGGGAAGCTTA-ATGAGACGACTCTTTTTTGAACTATATCATAAACAGATCTTCTTCTC

CACACCTATAACGAGTTTTTCTCCTTTCCTCTCGTATATAGTCGTGACGCCCTTAATGCTAGGTTTTGAA

AAAGACTTTTCATGTCATTTCCATTTAGGTCCGATTCGGATCCCTCCGTTGTTTCCTTTTCCTCCCGCAC

CTTTTCCTCGAAATGAGAAAGAAGATGGTACACTCGAATTGTATTATTTAAGTCTTTATTGCTTGCCAAA

GATCCTACTTCTACAATTGGTAGGTCACCGGGTTATTCAAATAAGTCGTGTTTTCTGTAGTTTTCCCATG

TTAGAACTTCTGTACCAATTCGGCCGATCCGGAATGGATCGGTTAAACATTCTATTAGGGGGCCCGGTCT

TGACTCTTCTGTGTGGTATTCATTCTCGTTCGGCTCTTGGAATCACATCCAGCAGTGGTTGGAACAGCTC

GCAAAATTCAACCACTTCACCTACTTTATTGCCCTCAACCCTTTCTCGTACTTCTATTGAAACTGAATGG

TTTCATGTTCTTTCATCGATTGGTTATTCTTCTCCGTTCGTATCTCTTTCTCCAATTTCGGTCTCGATTA

GTTCACAAGATTG-ATGTCCATTTTTTTATTACAACCTTC-----TTTTTTGATGTCAAAGACCAGAAGC

TACGCGCAAATTCTCATTGGATCTTGGTTGTTCTTAACAGCGATGGCTATTCATTTAAGTCTTTGGGTAG

CACCACTAGATCTTCAACAAGGTGGAAATTCTCGTATTCTCTATGTACATGTTCCTGTGGCTCGGATGAG

TATTCTTGTTTATATCGTTACGGCTATAAACACTTTTTTGTTCCTATTAACAAAACATCCTCTTTTTCTT

CGCTCTTCCGGAACCGGTATAGAAATGGGTGCTTTTTTTACGTTGTTTACCTTAGTTACTGGGGGGTTTC

GGGGAAGACCTATGTGGGGCACCTTTTGGGTGTGGGATGCTCGTTTAACTTCTGTATTCATATTGTTCCT

TATTTACCTAGGTGCACTGTGTTTTCAAAAGCTTCCTGTCGAACCGGCTCCTATTTCAATCCGTGCTGGA

CCGATCGATATACCAATAATCAAGTCTCCAGTCAACTGGTGGAATACATCGCATCAACCTGGGAGCATTA

GCCGATCCGGTACATCAATACATGTTCCTATGCTCATTCCAATCTTGTCCAACTTTGCTAACTCCCTCTT

CTCAACCCGTATCTTGTTTGTTCTGGAAACACGTCTTCCTATTCCATCTTTTCTCGAATCGCCTTTAACG

GAAGAAATAGAAGCTCGAGAAGGAATA-----------GAAAGATGCTAGTTGCTGCAATTCTATCTATT

TGTGCATCAAGTTCGAAGAAGATCTCAATCTATAATGAAGAAATGATAGTAGCTCGTTGTTTTATAGGCT

TTATCATATTCAGTCGGAAGAGTTTTTCTAAGACTATAAAAGTGACTCTCGACGAGAGAATCCAGGCTAT

TCAAGAAGAATCGCAACAATTCCCCAATCCTAACGAAGTCGTTCCTCCGGAATCCAATGAACAACAACGA

TTACTTAGGATCAGCTTGCGAATTTGTAGCACCGTAGTAGAATCATTACCAATGGCACGCTGTGCGCCTA

AGTGCGAAAAGACAGTGCAAGCTTTGTTATGTCGAAACCTAAATGTTAAGTCAGCAACACTTCCAAATGC

CACTTCTTCCCGTCGCATCCGTCTTCAGGACGATCTAATCACAGGGTTTCACTTCTCAGTGAGTGAAAGA

TTTCTCCCCGGGTCTACGTTGAAAGCTTCTATAGTAGAACTCATTCGAGAAGGCTTGGCGGTCTTAAGAA

TGGTTCGGGTAGGAGGTTCTTT---TGAAAGAGGCGATCAGAATGGTACCCGAATCCATTTACGATCCCG

AGTTTCCAGACACATCGCACTTCCGCTCGGGTCGAGGCTGCCACTCGGCCCTAAGACGGATCAAAGAAGA

GTGGGGAACCTCTCGCTGGTTTTTGGAATTCGACATCAGGAAGTGTTTTCACACCATCGACCGACATCGA

CTCCTCCCAATCTTTAAGGAAGAGATCGACGATCCCAAGTTCTTTTACTCCATTCATAAAGTCTTTTCTG

CCGGACGACTCGTAGGAGGCGAGAAGGGCCCTTACTCCGTCCCACACAGTGTACTACTATCGGCCCTACC

AGGCAACATCTACCTACACAAGCTCGATCAGGAGATAGGGAGGATCCGACAGAAGTACGAAATTCCGATT

GTTCAGAGAATCAGATCGGTTCTATTAAAGACAAGTCGTATTGATGACCAAGAAAACTCTGGAGAAGAAG

---TGGAATTTTCTCCCAGAGCTGCAGAACTAACGACTCTATTAGAAAGTAGAATTACCAACTTTTACAC

GAATTTTCAAGTGGATGAGATCGGTCGAGTGGTCTCAGTTGGAGATGGGATTGCACGTGTTTATGGATTG

AACGAGATTCAAGCAGGGGAAATGGTGGAATTTGCCAGCGGTGTGAAAGGAATAGCCTTAAATCTTGAGA

ATGAGAATGTAGGGATTGTTGTCTTTGGTAGTGATACCGCTATTAAAGAAGGAGATCTTGTCAAGCGCAC

TGGATCTATTGTGGATGTTCCTGCGGGAAAGGCTATGCTAGGGCGTGTGGTCGACGCGTTGGGAGTACCT

ATTGATGGAAGAGGGGCTCTAAGCGATCACGAGCGAAGACGTGTCGAAGTGAAAGCCCCTGGGATTATTG

AACGTAAATCTGTGCACGAGCCTATGCAAACAGGGTTAAAAGCGGTAGATAGCCTGGTTCCTATAGGCCG

TGGTCAACGAGAACTTATAATCGGGGACCGACAAACTGGAAAAACAGCTATTGCTATCGATACCATATTA

AACCAAAAACAAATGAACTCAAGGGCCACCTCTGAGAGTGAGACATTGTATTGTGTCTATGTAGCGATTG

GACAGAAACGCTCAACTGTGGCACAATTAGTTCAAATTCTTTCCGAAGCGAATGCTTTGGAATATTCCAT

TCTTGTAGCAGCCACCGCTTCGGATCCTGCTCCTCTGCAATTTCTGGCCCCATATTCTGGGTGTGCTATG

GGAGAATATTTCCGCGATAATGGAATGCACGCATTAATAATCTATGATGATCTTAGTAAACAGGCCGTGG

CATATCGACAAATGTCATTATTGTTACGCCGACCACCGGGCCGTGAGGCTTTCCCAGGCGATGTTTTCTA

TTTACATTCTCGTCTCTTAGAAAGAGCCGCTAAACGATCGGACCAGACAGGTGCAGGTAGCTTGACCGCC

TTACCTGTCATTGAAACACAAGCTGGAGACGTATCGGCCTATATTCCCACCAATGTGATCTCCATTACTG

ATGGACAAATCTGTTCGGAAACAGAGCTCTTTTATCGAGGAATTAGACCTGCTATTAACGTCGGCTTATC

TGTCAGTCGCGTCGGGTCTGCCGCTCAGTTGAAAGCTATGAAACAAGTCCGCGGTAGTTCAAAACTGGAA

TTGGCACAATATCGCGAAGTGGCCGCCCTTGCTCAATTTGGCTCAGACCTTGATGCTGCAACTCAGGCAT

TACTCAATAGAGGCGCAAGGCTTACAGAAGTACCGAAACAACCACAATATGCACCACTTCCAATTGAAAA

ACAAATTCTCGTCATTTATGCAGCTGTCAATGGATTCTGTGATCGAATGCCACTAGACAAAATTTCTCAA

TATGAGAAAACCATTCCAAATAGTGTAAAACCAGAATTATTACAATCCATTTTAGAAAAAAA--------

-TAACGAAAGAAAGATGGAACTAGATGCATTCTTAAAAGAGAGCGCTTTACCTT----------------

--------CACCCAACGCAAAGCGGCCTTTCATTCCCTTGTTTCGTCGTGGCACACCCTCCCCACAAGCA

CCCCCCGGCTCAGGGGGGACCAGAAAAGGCCTTTCGTTTTCCCCCCTTCGTCGGCCCTTGCCACCTTCCT

TAACAAGCCCTCGAGCCTCCTTTGCGCTGCCTTCCTCATAGAAGCCGCCGGGTTGACCCCGAAGGCCGAA

TTCTATGATGGAGAACGCTGTAATAAGAATTGGGCCATGAGAGACCTTATTAAGTATTGCAAAAGAAAGG

GCCTGCTGATAGAGCTGGGCGGGGAAGCGATACTAGTTATCAGGTCAGAGAAAGGCCTGGCCCGTAAGCT

GGCCCCCTTAAAAAGCCATTACTTAATAAGGATTTGTTACGCGCGATATGCCGACGACTTACTTTTGGGA

ATCGTGGGTGCCGTAGAGCTTCTCATAGAAATACAAAAACGTATCGCCCACTTCCTACAATCCGGCCTGA

ACCTTTGGGTAGGCTCTGCAGGATCAACAACAATAGCTGCACGGAGTACGGTAGAATTCCTCGGTACGGT

CATTCGGGAAGTCCCTCCGAGGGCGACTCCCATACAATTCTTGCGAGAGCTGGAAAAGCGTCTACGGGTA

AAGCATCGTATCCATATAACTGCTTGCCACTTACGCTCTGCCATCCATTCCAAGTTTAGGAACCTAGGTA

ATAGTATCCCGATCAAACAGATGACGAAGGGGATGAGCAAAACAGGGAGTCTACTGGACGCGGTTCAACT

AGCGGAGACTCTTGGAACAGCTAGAGTAAGAAGTCCCCA---GAGCGTATTATGGGAGACCGTCAAGCAC

ATCCGGCAAGGATCAAGGGAGATCTCGTTGTTGCATAGCTCAGGTCAGAGCAACGTGCCATCGGACGTTC

GGCAGGCAGTCTCACGATCGGGCATGAGTGTCCGGAAGTTTTC---ATTGTATACTCTCGCGGGTCGGAA

GGCGGCGGGGGAAGGAGGGGGACACTGGGCGAGATCTATCAGCAGCGAATTCCCCATACAGATAGAAGCG

CCTATCAAAAAGATACTCCGAAGGCTTCGAGATCGAGGTATCATTAGCCGAAGAAGACCCTGGCCAATCC

ACGTGGCCTGCTTGACGAACGTCAGCGACGGAGACATCGTAAATTGGTCCGCGGGCATCGCGATAAGTCC

TCTGTCCTACTACAGGTGCTGCGACAACCTTTACCAAGTCCGAACGATTGTCGACCACCAGATCCGCTGG

TCTGCAATATTCACCCTAGCCCACAAGCACAAATCCTCAGCGCGGAATATAATCCCAAAGTACTCCAAAG

ACTCAAATATAGTACATCCAGAAGGTGGTAAGACCCTTGCAGAGTTCCCCAACAGCATGGAGCTTGGGAA

GCTCGGACCCGGTCAAGATCC-GAACAACAAAGAGCACTCAACTACTA-----------------ATGGT

CCAACTGCATAACTTTTTCTTTTTCATTACTTCCATGGTCGTGCCTTGTGGCACGGCAGCACCCGTACTA

TTGAAATGGTTCGTCAGTAGAGATGTTCCCACAGGTGCCCCCTTTTCCAATGGTACTATAATTCCTATTC

TTATCCCTTCATTCCTTCTTTTGGTCTATCTACATTCAAGGAAATTCATACGCTCCATGGACGGAGCAAA

AAGTGGAGTCTTGGTCAGAGCAAGCTGCCCTATTCTAT------TACCAGACATAATTGGGAGAAGCTCA

TCCGAAACTAGAGCTAGAAACGCCTTATTTCGTTTCGTTCCCGTTCTTCATTTTCTTCTTCTCGAATCCA

A------GGGGGACTTCTCATATTTAGAATCTTTTTGCGGTGTGCTCCGTTTACTATTCTTTCGTACTTT

CTTTTCTTTACCACGCGATAGGTCAGCGAAGCGTGAGCGGGCGCGGAGAAGAAAAGGCCAAACACTTCGG

CCTAAC------GGGAATGAGCAACGACGAAATGACAAGAGAAAGTGCCCCGGGCACCCCCATT------

TAGA---AAGAAGGGTCGAAGGTTTTGGGCCTGTAGCTTTCCCCGCCCCCCCTTCGTCGGGTGGTGCTT-

--TGGGGGGTGTGCTACCAGAAATCGGGCTTGAAGCTCTCGCCTTACCAACGAGCCGACAGCTGATGGCT

GTTGGTCACGACTACTACCAAAAAGTGAACATGAAGATGAATATTTCACATGGAGGAGTGTGCATCTGTA

TGTTGGGTGTTCTTCTG-ACGTACATAGCTGTTCCAGCTGAAATACTTGGAATAATTCTACCACTTCTAC

TAGGAGTAGCCTTTTTAGTGCTAGCTGAACGTAAAGTAATGGCTTTTGTGCAACGTCGAAAGGGTCCTGA

TGTAGTGGGATCATTCGGATTGTTACAACCTCTAGCAGATGGTTTGAAATTGATTCTAAAAGAACCTATT

TCACCAAGTAGTGCTAATTTCTCCCTTTTTAGAATGGCTCCAGTGGCTACATTTATGCTAAGTCTGGTTG

CTCGGGCCGTTGTACCATTTGATTATGGTATGGTATTGTCAGATCCGAACATAGGGCTACTTTATTTGTT

TGCCATATCTTCGCTAGGTGTTTATGGAATTATTATAGCGGGTCGGTCTAGT-TTTATTATATACGTTTA

GCGAAAAGAATGTTTTTTGATACACCTAGGACATGGATTCTATATGAACCAATGGATCGTGACAAGTCGT

TACTATTAGCAATGACTTCCTCTTTCATTACTTCATCCTTTCCATATCCTTCTCCCTTGTTCTCAGTTAC

TCATCAAATGGCACTCAGTTTATATCTTTA-ATGTCAGAATTTGCGCCTATTTGTATCTATTTAGTGATC

AGTCCGCTAGTTTCTTTGATCCCACTCGGTCTTCCTTTTCCATTTTCTTCGAATAGTTCGACTTATCCAG

AAAAATTGTCGGCCTACGAATGTGGTTTCGATCCTTCCGGTGATGCCAGAAGTCGTTTCGATATACGATT

TTATCTTGTTTCAATTTTATTTATTATCCTTGATCCGGAAGTCACCTTTTTCTTTCCTTGGGCAGTACCT

CCCAACAAGATTGATCCGTTTGGATCTTGGTCCATGATGGCCTTTTTATTGATTTTAACGATTGGATTTC

TCTATGAATGGAAAAGGGGTGCTTCGGATCGGGAGTA--AAAGTGTTTCTTACGATTACGCCCAACAGCC

CACTTGAGCAATTTGCCATTATCCCATTGATTCCTATGAATATAGGAAACTTGTATTTCTCATTCACAAA

TCCATCTTTGTTTATGCTACTAACTCTCAGTTTGGTCCTACTTCTGGTTCATTTTGTTACTAAAAAGGGA

GGAGGAAACTCAGTACCAAATGCTTGGCAATCCTTGGTAGAGCTTATTTATGATTTCGTGCTGAACCCGG

TAAACGAACAAATAGGTGGTCTTTCCGGAAATGTTAAACAAAAGTTTTCCCCTCGCATCTTGGTCACTTT

TATTTTTTTGTTATTTCGTAATCTCCAGGGTATGATACCTTATAGCTTTACAGTTACAAGTCATTTTCTC

ATTACTTTGGGTCTTTCATTTTCCATTTTTATTGGCATTACTATAGTGGGATTTCAAAGAAATGGGCTTC

ATTTTTTAAGCTTCTCATTACCCGCAGGAGTCCCACTGCCATTAGCACCCTTTTTAGTACTCCTTGAGCT

AATCCCTCATTGTTTTCGCGCATTAAGCTCAGGAATACGTTTATTTGCGAATATGATGGCCGGTCATAGT

TCAGTAAAGATTTTAAGTGGGTCCGCTTGGACTATGCTATGTATGAATGAGCTTTTATATTTCATAGGAG

ATCCTGGTCCTTTATTTATAGTTCTTGCATTAACCGGTCTGGAATTAGGTGTAGCTATATTACAAGCTCA

TGTTTTTACGATCTCAATCTGTATTTACTTGAATGATGCTACAAATCTCCATCAAA--------------

----------------ATATGTGGGCACCTGATATCTATGAGGGTTCACCCACCCCGGTTACAGCATTCT

TTTCTATTGCGCCTAAAATCTCTATTTCTGCGAATATTTTACGTGTTTTTATTTATGGTTCCTATGGAGC

TACATTGCAACAAATCTTCTTTTTCTGCAGCATTGCTTCTATGATCTTAGGAGCACTGGCCGCCATGGCC

CAAACGAAAGTAAAAAGACTTCTAGCTCATAGTTCAATTGGACATGTAGGTTATATTCGTACTGGTTTCT

CATGTGGAACCATAGAAGGAATTCAATCACTACTAATTGGTCTCTTTATTTATGCATCAATGACGATAGA

TGCATTCGCTATAGTTTCAGCATTACGGCAAACCCGTGTCAAATATATAGCGGATTTGGGCGCTCTAGCC

AAAACGAATCCTATTTCGGCTATTACCTTCTCTATTACTATGTTCTCATACGCAGGAATACCCCCGTTAG

CCGGCTTTTGTAGTAAATTCTATTTGTTCTTCGCCGCTTTGGGTTGTGGGGCTTACTTCCTAGCCCCAGT

GGGAGTAGTGACTAGCGTTATAGGTCGT--GTTCGATAGCCCGACCGTAGTGATGTTAATTGTGGTTACA

TCCATAAGTAGCTTGGTCCATCTTTATTCTATTTCATATATGTCTGAGGATCCGCATAGCCCTCGATTTA

TGTGTTATTTATCCATTCTTACTTTTTTTATGCCAATGTTGGTGACTGGAGATAACTCTCTTCAATTATT

CTTGGGATGGGAGGGAGTAGGTCTTGCTTCATATTTGTTAATTCAGTTTTGGTTTACACGACTTCAGGCA

GATAAAGCAGCTACAAAAGCTATGCTTGTCAATCGAGTAGGTGATTTTGGATTAGCTCCTGGGATTTCGG

GTCGTTTTACTCTCTTTCAAACAGTAGACTTTTCTACCATTTTTGCTTGTGCTAGTGCCCCTAGAAATTC

TTGGATTTCTCGTAATATGAGATTGAATGCCATAACTCTTATTTGTATTTTACTTTTTATTGGTGCTGTT

GGAAAATCTGCACAGATAGGATCGCATACTTGGTTACCCGATGCTATGGAGGGTCCCACCCCAGTATCCG

CTTTGATTCATGCAGCTACTATGGTAACAGCTGGCGTTTTCATGATAGCAAGGTGTTCTCCTTTATTTGA

ATACCCACCTACGGCTTTAATTGTTATTACTTTTGCAGGAGCTACGACGTCATTCCTTGCGGCAACCACT

GGAATATTACAGAACGATCTAAAGAGGGTCATAGCTTATTCAACTTGCAGTCAATTAGGCTATATGATCT

TTGCTTGCGGCATTTCTAACTATTCGGTTAGCGTCTTTCATTTAATGAATCACGCGTTTTTCAAAGCATT

ACTATTCCTGAGTGCGGGTTCGGTGATTCATGCCATGTCGGATGAGCAAGATATGCGGAAGATGGGGGGG

CTTGCCTCCTCGTTCCCTTTTACCTATGCCATGATGCTCATGGGCAGCTTATCTCTAATTGGATTTCCTT

TTCTAACTGGATTTTATTCCAAAGATGTGATCTTAGAGCTCGCTTACACTAAGTATACCATCAGTGGGAA

CTTTGCTTTCTGGTTGGGAAGTGTCTCTGTCCTTTTCACTTCTTATTACTCTTTTCGTTTACTTTTTCTA

ACATTTCTAGTACCAACTAATTCATTCGGGCGAGACATCTTACGATGTCATGATGCGCCCATTCCTATGG

CCATTCCTTTAATACTTCTGGCTTTCGGGAGTCTCTTTGTAGGATACTTGGCCAAACTAACACAAAGAAG

ATACAGTTCACTCAACGATTGCCTTTGGGTTCCGAACTCCGTATGGGGAAGGAACGTTGTTGTTTGCGAG

GTCTCGATCATTTACATGGACCCACTTCTCATTCCATTTGTGGGAATTTGATGATCTATAAACCGTCCTT

AACGAACGATCGGCTCATGTT------TGAGCATGATGAATCACTTCGTGCCGACCTGTTGTCAATAAAC

TTTTCGGCCTCATATGAGAATGCAAAACTGGAGCATTTTCTGAATCGGTGGATGAAGAATCGCGAACATA

ATCAATTCTGGTTGACCATGTTCCCAGAAAAAAGATACTTTCGAGAAACAACGAGCACGACTGAAGTGGC

TATACATACAAATCTATTTACGGATCTATATGCTTCGATTGGAACTGGAAGTTCCAGAACAGGCGGCTGG

TATACTACCATAATGAAACTGCCTTTTCTTTTTTTTATTCGGATAGGATTTATGTTGGCTTCGTCGGGAG

GCTCACGTAGTTTGTTACGTCAGCTCCAAAAGGATAAATTGCGTTGGAATCGAGAAAGTTCCGTAA----

AGTTCATAATTGTATA-CCAATTTTTGGGCCAATTCCCTCTTTGTACTACCAAAAAATGAGATTCTTGCC

GAATCCGAGTTTGCTGCTCCAACCATTACCAAACTAATACCTATTCTGTTTAGTACTTCAGGTGCTTCTG

TTGCGTATAATGTAAATCCCGTAGCGGATCAATTCC---------------AACGAGCCTTTCAAACTAG

TACTTTTTGTAATCGACTCTATAGCTTCTTCAATAAACGCTGGTTCTTCGATCAAGTTTTGAATGACTTT

CTAGTCAGATCGTTCTTGCGTTTCGGATATGAAGTCTCATTTGAAGCTTTAGACAAAGGTGCTATTGAGA

TATTGGGCCCTTATGGTATCTCGTACACATTCCGACGATTGGCCGAGCGAATAAGTCAACTTCAAAGTGG

ATTTGT-TTGTTCCATGATCTATGGGTCTACTGGAGCTACCCACTTCGATCAATTAGCCAAGATTTTGAC

CGGATACGAAATCACTGGTGTTCGATCTAGTGGTATTTTTATGGGGATTCTATTTATCGCTGTAGGATCC

CTGTTCAAGATCACTGCAGTTCCTTTT-TGGTCTATGCACATCGCTTTCTCCAGGAGGTTGGTCGCCTAT

CCTAGATCTTCCTATTTCCAAGAGGATCCCGGGCTCAATCTGGTTTAGTATCAAGGTGATTCTTTTTCCG

TTCCTATATATATGGGTCCGTGCAGCATTTCCACGATATCGTTATGATCAATTAATGGGACTTGGCCGGA

AAGTGTTCTTGCCTCTATCATTAGCTCGGGTAGTCCCCGTTTCTGGTGTTTTAGTCACCTTTCAATGGCT

CCCTTA-ATGCCTCAGCTGGATAAATTTACTTATTTTACACAATTCTTCTGGTTATGCCTTTTCTTCTTT

ACTTTCTATATTCTAATATGCAATGATAGAGATGGAGTACTTGGGATCAGCAGAATTCTAAAACTACGAA

ACCAACTGCTTTCACACCGGGGGAACAACATCCAAA------GCAAGGACCCCAACTGTTTGGAAGATAT

CTTGCGAAAAGGTTTTAGCACCGGTGCATCCTATATGTACTCTAGTTTATTCGAAGTATCCCAATGGTGT

AAGGCCGTCGACTTATTGGGAAAAAGGAAGAAAATCACTTTGATCTCTTGTTTCGGAGAAATAAGTGGCT

CACGAGGAATGGAAAGAAACATCTTATATTTGATCTCGAAGTCTTCATATAGCACTTT------------

---TTCCAATCCTGGATGGGGGATCACTTGTAAAAATGACATAATGCTAATCCATGTTCTACACGGACAA

GGA--------------------

>Pereskia aculeata NC067638.1

-TGATACTTTCTGTTTTGTCGAGCCCGGCTTTGGTCTCTGGTTTGATGGTTGTACGTGCTAAAAATCCGG

TACATTCCGTTTTGTTTCCCATCCCAGTCTTTTGCAACACTTCAGGTTTACTTCTTTTGTTAGGTCTCGA

CTTTTCCGCTATGATCTTCCCAGTAGTTTATATAGGAGCTATAGCCGTTTCATTCCTATTCGTTGTTATG

ATGTTCCATATTCAAATAGCGGAGATTCACGAAGAAGTATTGCGCTATTTACCAGTGAGTGGTATTATTG

GACTGATCTTTTGGTGGGAAATGTTCTTCATTTTAGATAATGAAACCATTCCATTACTACCAACCCAAAG

AAATACGACCTCTCTGAGATATACGGTTTATGCCTCCAAGGTACGAAGTTGGACTAATTTGGAAACATTG

GGCAATTTACTTTATACTTACTATTTTGTCTGGTTTTTGGTTTCTAGTCTTATTTTATTAGTAGCCATGA

TTGGGGCTATAGTACTGACTATGCATAGGACTACTAA------GGTGAAAAGACAGGATGTATTCCGACG

AAATGCTATTGATTCTAGAAGGACTATAATGAGGAGGACGACAGACC--TGTCAATATATGAATTGTTTC

ATTATTCGTTATTTCCGGGTCTTTTCATTGCATTCACTTACAACAAAAAACAACCACCAGCGTTTGGTGC

AGCACCCGCATTTTGGTGTATTCTTCTTTCTTTCCTTGGTCTTTTGTTCTGTCATATTCCTAATAACTTA

TCCAATTACAACGTATTAACCGCTAATGCACCTTTCTTTTATCAAATCTCAGGGACATGGTCTAATCATG

AAGGTAGTATTTTATTATGGTGTCGGATCCTTTCTTTTTATGGATTCCTTCTTTGTTACCGGGGTCGATC

CCAAAGCCATAATGTCTCAAAACGAGGAGGCCATAGAGAAAGTCTTCTTTTTTCCTTTGTCTTAAACTTC

TTCACGAACTCCATTCGCACTCTTCCTCGTTACGAGCAAAAAAGTAGAGTTCTTCACGAACCCCAGTTGT

ACACTCTCTTCGTTCTACGAA---CTGAAGTTGATTCTGAACTTTGTTCGCGAAGGAACCGGACTTTTGA

CGGGCCAGCTCTTTTTTACGCGCCGCTTTACCCTGAAAGGAAAATGAGCTTTGCTCTTCTGGGTGCTAGG

CGCTCTCGTGGTTCGCGAGAAGGAAAAAGGACTCATCCTTTGTTGCATCTGGCACGAGATGATAAAGAGA

GAGCTTCGTCTATCGATGAACAGCGGATTGACGGAGCTCTTGGCATTGCTTTGTTTTTCTTTCCTTTCCT

ATCAGCGAGTTCCGATCCTTTTGTTCGAAATTTCTTCGTTCGTACCGAACCGCTTGCAGAATCAAATCCT

GTTCCACAAGATCCTATATCAGCTATACATCCTCCTTGCATTTATGCCGGAGACGTCGCCAGTGCTATGG

GCTTTGCCTTATGTAGATCAAAAATGATGAATGGGATTGTGGCACTCCACTCGCCGCCAATGTGGAAGGA

TGTCGCCGAAAAGAATGGAACGCTGCTTTGCTCTGCTGGATGCGTCGGATCCCGTATAACTTCTGAGCTC

TTTACCCAACAATTCAAACATGTGGGCGCCAAATGCTATCCTGCTCTATTGTTGCGTAGCAATAGAAGCC

TGCTC---ATGCTGCTTCGGCGGCGCTTTTTCGCCTTCTCTTCGCTCTGGACAAGAGCGCTAGTGGACAC

GGGGAGGGAGCG------GGCGAAGCCTTTCT------TTCGTAATGGAAAGAAAGATACCACTACTTCG

CCTCTTTGTTGGACCGCCGGCGCGAACACAGTGGTCTCTGACCAGGACCAGGAACCAATTCGAATTTGGA

TCTTGACATGTCGGTGGTTTTTAACCGTAGGCATCTTGCCAGGAAGTTGGTGGGCTCATCATGAATTAGG

TTGGGGTGGCTGGTGGTTTCGGGATCCCGTAGAAAATGCTTCTTTTATGCCTCGGGTATTAGCCACAGCT

CGTATTCATTCAGTAATTTTACCCCTTCTTCATTCTTGGACTTTGCTTCTTAATATTGTGACTTTTCTAT

GCTGTGTCTTAGGAACCTTTTCCATACGGTCCGGATTGCTAGCTCCCGTTCATAGTTTTGCTACAGATGA

TACACGAGGAATCTTTTTATGGAGGTTCTTCCTTCTAATGACCGGCATATCTATGATTCTTTTCTCTCAG

ATGAAGCAGCAGGCATCGGTCCGTAGAACCTATAAAAAAGAGATGGTTGTAGCGCGA-------------

-----------TGATTGTTCGAGAATGGCTATTCTTCCCAATTGCTCCTTGTGATGCAGCGGAACCATGG

CAATTAGGATTTCAAGACGCAGCAACACCTATGATGCAAGGAATAATAGACTTACATCATGATATCTTTT

TCTTCCTCATTCTGATTTTGGTTTTCGTATCATGGATCTTGGTTCGCGCTTTATGGCATTTCCACTATAA

AAAAAATCCAATCCCGCAAAGGATTGTTCATGGAACTACTATCGAGATTATTCGGACCATATTTCCGTCT

ATCATCCTGATGTTCATTGCTATACCATCATTTGCTCTGTTATACTCAATGGACGAGGTAGTAGTAGATC

CAGCCATTACTATCAAAGCTATTGGACATCAATGGTATCGGAGT--------------------------

----------------------------------------------------------------------

----------------------------------------------------------------------

----------------------------------------------------------------------

----------------------------------------------------------------------

----------------------------------------------------------------------

------------------------TGAGACGACTCTTTTTTGAACTATATCATAAACAGATCTTCTTCTC

CACACCAATCACGAGTTTTTCTCCATTCCTCTCGTATATTGTCGTAACGCCCTTAATGCTAGGTTTTGAA

AAAGACTTTTCATGTCATTTCCATTTAGGTCCGATTCGGATCCCCCCGTTGTTTCCTTTTCCTCCCGCAC

CTTTTCTTCGAAATGATAAAGAAGATGGTACACTCGAATTGTATTATTTAAGTGCTTATTGCTTGCCAAA

GATCCTACTTCTACAATTGGTGGGTCACCGGGTTATTCAAATAAGTCGTGTTTTCTGTAGTTTTCCCATG

TTACAACTTCTGTACCAATTCGGTCAATCCGGAATGGATCGGTTAAACATTCTATTAGGGAGCCCGGTCT

TGACTCTTCTGTGTGGTATTCATTCTCGTTTGGCTCTTGGAATCACATCCAGCAGTGGTTGGAACAGCTC

GCAAAATTTAACCACTTCACCTACTTCATTGCCCTCAACCGTTTCTCGTACCTCTATTGAAACAGAATGG

TTTCATGTTCTTTCATCGATTGGTTATTTTTCTTCGTTCGTATCTCTTTTTCCAATTTCGGTCTCGATTA

GTTCACAAGATTGA-TGTCCGTTTTGTTATTACAACCTTA-----TTTTTTGATGTCAAAGACCAGAAGC

TACGCGCAAATTCTCATTGGATCTTGGTTGTTCTTAACAGCGATGGCTATTCATTTAAGTCTTTGGGTAG

CACCACTAGATCTTCAACAAGGTGGAAATTCTCGTATTCTCTATGTACATGTTCCTGTGGCTCGGATGAG

TATTCTTGTTTATATCGTTACGGCTATAAACACTTTCTTGTTCCTATTAACAAAACATCCTCTTTTTCTT

CGCTCCTCCGGAACCGGTACAGAAATGGGTGCTTTTTCTACGTTGTTTACCTTAGTTACTGGGGGGTTTC

GGGGAAGACCCATGTGGGGCACCTTTTGGGTGTGGGATGCTCGTTTAACTTCTGTATTCATCTCGTTCCT

TATTTACCTGGGTGCACTGTGTTTTCAAAAGCTTCCTGTCGAACCGGCTCCTATTTCAATCCGTGCTGGA

CCGATCGATATACCAATAATCAAGTCTTCAGTCAACTGGTGGAATACATCGCATCAACCTGGGAGCATTA

GCCGATCTGGTACATCAATACATGTTCCTATGCCCATTCCAATCTTGTCTAACTTTGCTAACTTCCTCTT

CTCAACCCGTATCTTGTTTGTTCTGGAAACACGTCTTCCTATTCCATCTTTTCTCGAATCTCCTTTAACG

GAAGAAATAGAAGCTCGAGAAGGAA---------------AAGATTCTATTTGCTGCTATTCTCTCTATT

TGTGCATTAAGTTCGAAGAAGATCTCAATCTATAATGAAGAAATTATAGTAGCTCGTTGTTTTATAGGCT

TTATCATATTCAGTCGGAAGAGTTTAGGTAAGACTTTCAAAGCGACTCTCGACGAGAGAATCCAGGCTAT

TCAGGAAGAATCGCAGCAATTCCCCAATCCTAACGAAGTAGTTCCTCCGGAATCCAATGAACAACAACGA

TTACTTAGGATCAGCTTGCGAATTTGTGGAACCGTAGTAGAATCATTACCAATGGCACGCTGTGCGCCTA

AGTGCGAAAAGACAGTGCAAGCTTTGTTATGTCGAAACCTAAATGTTAAGTCAGCAACACTTCCAAATGC

CACTTCTTCCCGTCGCACCCGTCTTCAGGACGATCTAGTCACAGGTTTTCACTTCTCAGTGAGTGAAAGA

TTTGTCCCCGGGTCTACGTTGAAAGCTTCTATAGTAGAACTCATTCGAGAAGGCTTGGCGGTCTTAAGAA

TGGTTCGGGTAGAAGGTTCTCTT--TGAAAGAGGCGATCAGAATGGTACCCGAATCCATTTACGATCCCG

AGTTTCCAGACACATCGCACTTCCGCTCGGGTCGAGGCCGCCACTCGGCCCTAAGACGGATCAAAGAAGA

GTGGGGAACCTCTCGCTGGTTTTTGGAATTCGACATCAGGAAGTGTTTTCACACCATCGACCGACATCGA

CTCATCTCAATCTTTAAGGAAGAGATCGACGATCCCAAGTTCTTTTACTCCATTCAGAAAGTCTTTTCTG

CCGGACGACTCGTAGGAGGTGAGAAGGGCCCTTACTCCGTCCCACACAGTGTACTACTATCGGCCCTACT

AGGCAACATCTACTTACACAAGCTCGATCAGGAGATAGGGAGGATCCGACAGAAGTACGAAATTCCGATT

GTTCAGAGAATCAGATCGGTTCTATTAAAGACAAGTCGTATTGATGACCTTCAAAACTCTGGAGAAGAAG

---TGGAATTCTCTCCCAGAGCTGCGGAACTAACGACTCTATTAGAAAGTAGAATTACCAACTTTTACAC

GAATTTTCAAGTGGATGAGATCGGTCGAGTGGTCTCAGTTGGAGATGGGATTGCGCGTGTTTATGGATTG

AACGAGATTCAAGCTGGGGAAATGGTGGAATTTGCCAGCGGTGTGAAAGGAATAGCCTTAAATCTTGAGA

ATGAGAATGTAGGAATTGTTGTCTTTGGTAGTGATACCGCTATTAAAGAAGGAGATCTTGTCAAGCGCAC

TGGATCTATTGTGGATGTTCCTGCGGGAAAGGCTATGCTAGGGCGTGTGGTCGACGCGTTGGGGGTACCT

ATTGATGGAAGAGGGGCTCTAAGTGATCACGAGCGTCGACGTGTCGAAGTGAAAGCCCCTGGGATTATTG

AACGTAAATCTGTGCACGAGCCTATGCAAACAGGGTTAAAGGCGGTAGATAGCCTGGTTCCTATAGGCCG

TGGCCAACGAGAACTTATAATCGGGGACCGACAAACGGGAAAAACAGCTATAGCTATCGATACCATATTA

AACCAAAAGCAACTGAACTCAAGGGCCACCTCTGAGAGTGAGACATTGTATTGTGTCTATGTAGCGGTTG

GACAGAAACGTTCAACTGTGGCACAATTAGTTCAAATTCTTTCCGAAGCGAATGCTATGGAATATTCCAT

TCTTGTAGCAGCCACCGCTTCGGATCCTGCTCCTCTTCAATTTCTGGCCCCATATTCTGGGTGTGCTATG

GGAGAATATTTCCGCGATAATGGAATGCACGCATTAATAATATATGATGATCTTAGTAAACAGGCGGTGG

CATATCGACAAATGTCATTATTGTTACGCCGACCACCAGGCCGTGAGGCTTTCCCAGGCGACGTTTTCTA

TTTACATTCTCGTCTCTTAGAAAGAGCCGCTAAACGATCGGACCAGACAGGTGCAGGTAGCTTGACCGCC

TTACCCGTCATTGAAACACAAGCTGGAGACGTATCGGCCTATATTCCCACCAATGTGATCTCCATTACTG

ATGGACAAATCTGTTCGGAAACAGAGCTCTTTTATCGCGGAATTAGACCTGCGATTAACGTCGGCTTATC

TGTCAGTCGCGTCGGGTCTGCCGCTCAGTTGAAAGCTATGAAACAAGTCTGCGGTAGTCCAAAACTGGAA

TTGGCACAATATCGCGAAGTGGCCGCCTTTGCTCAATTTGGGTCAGACCTTGATGCTGCGACTCAGGCAT

TACTCAATAGAGGTGCAAGGCTTACAGAAGTACCGAAACAACCACAATATGCACCACTTCCAATTGAAAA

ACAAATTATAGTCATTTACGCAGCTGTCAATGGATTCTGTGATCGAATGCCACTAGATAAAATTTCTCAA

TATGAGAGAACCATTCCAAATAGTGTAAAACCAGAATTATTACAATCCCTGTTATCAAAGGATGGGTTAA

ATAACGAAATAAAGATGGAACTAGATGCATTCTTAAAAGAATGCGCTTTGCCTTACC-------------

--------CATCCAACGCAAAGCGGCCTTTCATTCCCTTGTTTCGTCGTGGCACACCCTCCCCACAAGCA

CCCCCCGGCTCAGGGGGGACCAGAAAAGGCCTTTCGTTTTCCCCCCTTCGTCGGCCCTTGCCACCTTCCT

TAACAAGCCCTCGAGCCTCCTTTTCGCTGCCTTCCTCATAGAAGCCGCCGGGTTGACCCCGAAGACCGAA

TTCTATGGTAGAGAAGGCTCTAATAATAATTGGGCCATGAGAGACCTTTTTAAGTATTGCAAAAGAAAGG

GCCTGCTGATAGAGCTGGGCGAGGCAGCGATACTAGTTATCAGGTCAGAGAAAGGCCTGGCCCGTAAGCT

GGCCCCCTTAAAAACCCATTACTTAATAAGGATTTGTTACGCGCGATATGCCGACGACTTACTATTGGGA

ATCGTGGGTGCCGTAGAGCTTCTCATAGAAATACAAAAACGTATCGCCCACTTCCTACAATCCGGCCTGA

ACCTTTGGGTAGGCTCTGCGGGATCAACAACAATAGCTGCACGGAGTACGGTAGAATTCCTCGGTACGGT

CATTCGGGAAGTCCCCCCGAAGACAACTCCCATACAATTCTTGCGAGAGCTGGAGAAGCGTCTACGGGTA

AAGCACCGTATCCATATAACTGCTTGCCACTTACGCTCCGCCATTCATTCTTCGTTTAGGAACCTAGGTA

AGAGTATCCCGGTCAAACAGCTGACGAAGGGGATGAGCAAAACAGGGAGTCTACTGGACGCGGTTCAACT

AGCGGAAACTCTTGGAACAGCGTCAGTAAGAAGTCCCCAAGTGAGCCTATTATGGGATACCGTCAAGCAC

ATCCGGCAAGGATCAAGGGAGATCTCGTTGTTGCATAGCTCAGGTCAGAGCAAGGCGCCATCGGACGTTC

AACAGGCAGTCTCGCGATCGGGCATGAGTGTCCGGAAGTTGTC---ATTGTATACTCTCGCGGGTCGGAA

GGCGGCGGGGGAAGGAGGGGGACACTGGGCGAGATCTATCAGCAGCGAATTCCACATACAGATAGAAGCG

CCTATCAAAAAAATACTCCGAAGGCTTCGAGATCGAGGTATCATTAGCCGAAGAAGACCCTGGCCAATCC

ACGTGGCCTGCTTGACGAACGTCAGCGACGGAGACATCGTAAATTGGTTCGCGGGCATCGCGATAAGTCC

TCTGTCCTACTACAGGTGCCGCGACAACCTTTACCAAGTCCGAACGATTGTCGACTACCAGATCCGCTGG

TCTGCAATATTCACCCTAGCCCACAAGCACAAATCCTCAGCGCGGAATATAATCCCAAAGTACTCCAAAG

ACTCAAATATAGTAAATCTTCAAGGTGGTAAGACCCTTGCAGAGTTCCCCAACAGCATAGAGCTTGGGAA

GCTCGGACCCGGTCAAGATCC-GAACAACAAAGAGCACTCAACTACGTTTGACGTCGTCTAGTTGATGGT

CCAACTACATAACTTTTTCTTTTTCATTACTTCCATGGTCGTGCCTTGTGGCACGGCAGCACCCGTACTA

TTGAAATGGTTCGTCAGTAGAGATGTTCCCACAGGTGCCCCTTTTTCCAATGGTACTTTAATTCCTATTC

TTATCCCTTCATTCCTTCTTTTGGTCTATCTACATTCCAGGAAATTCATACGCTCCATGGACGGAGTAAA

AAGTGGAGTCTTGGTCAGAGCAAGCTGCCCTATTTTAT------TACCAGACATAATTGGGAGAAGCTCA

TCCGAAACGTTAGCGTTAAAGGCCTTATTTCGTTTCGTTCCCATTCTTCATTTTCTTCTTCTCGAATCCA

A------GGGGGACTTCCCATATTTAGAATCTTTTTGCGGTGTGCTCCGTTTACTATTCTTTCGTACTTT

CTTCTCTTTACCACGCGATAGGTCAGCGAAGCGTGAGCGGGCGCGGAGAAGAAAAGGCCAAACCCAAAGG

CCTAAC------GGGAATGAGCAACGACGAAATGACTTCATAAAGTGCCCCGGGCGCCCCCATG------

ACGA---AAGAAGGGTCGAAGGGTTTGGGCCTGTAGCTTTCCCCGTCCCCCCTTCGTCGGGTGGTGCTTG

TGTGTGGGGTGTGCCACCTGAAATCGGGCTTGAAGCTCCCGCCTTACCAACGAGCCGACAGCTGATGGCT

GTTGGTCACGACTACTACCAAAAAGTGAACATGAAGATGAATATTTCACATGGAGGAGTGTGCATCTTTA

TGTTGGGTGTTCTTCTG--CGTACATAGCTGTTCCAGCTGAAATACTTGGAATAATTCTACCACTTCTAC

TAGGAGTAGCCTTTTTAGTGCTAGCTGAACGTAAAGTAATGGCTTTTGTGCAACGTCGAAAGGGTCCTGA

TGTAGTGGGATCGTTCGGATTGTTACAACCTCTAGCAGATGGTTCGAAATTGATTCTAAAAGAACCTATT

TCACCAAGTAGTGCTAATTTCTCCCTTTTTAGAATGGCTCCAGTCACTACATTTATGCTAAGTCTGGTTG

CTCGGGCCGTTGTACCTTTTGATTATGGTATGGTATTGTCAGATCCGAACATAGGGCTACTTTATTTGTT

TGCCATATCTTCGCTAGGTGTTTATGGAATTATTATAGCAGGTTGGTCTAGTATTTATTATATACGTTTA

GTGAAAAGAATGTTTTTTGATACACCTAGGACATGGATTCTATATGAACCAATGGATCGTGACAAGTCGT

TACTACTAGCAATTACTTCCTCTTTCATTACTTCATCCTTTCCATATCCTTCTCCCTTGTTCTCAGTTAC

TCATCAAATGGCACTCAGTTTATATCTTTA--TGTTAGAATTTGCGCCTATTTGCATCTATTTAGTGATC

AGTCTGCTAGTTTCTTTGATCCCACTCGGTGTTCCTTTTCCATTTTCTTCTAATAGTTCGACTTATCCAG

AAAAATTGTCGGCCTACGAATGTGGTTTCGATCCTTTCGGTGATGCCAGAAGTCGTTTCGATATTCGATT

TTATCTTGTTTCAATTTTATTTATTATCCTTGATCCGGAAGTAACCTTTTTCTTTCCTTGGGCAGTACCT

CTCAACAAGATTGATCCGTTTGGATCTTGGTCCATGATGGCCTTTTTATTGATTTTAACGATAGGATTTC

TCTATGAATGGAAAAGGGGTGCTTTGGATCGGGAGTAA-AAAGTGTTTATTACGATTACGCCCAACAGCC

CACTTGAGCAATTTGCCATTCTCCCATTGATTCCTATGAAAATAGGAAACTTGTATTTCTCATTCACAAA

TCCATCTTTGTTTATGCTGCTAACTCTCAGTTTGGTCCTACTTCTGCTTCATTTTGTTACTAAAAACGGA

GGAGGAAACTCAGTACCAAATGTTTGGCAATCCTTGGTAGAGCTTATTTATGATTTCGTGCTGAACCTGG

TAAACGAACAAATAGGTGGTCTTTCCGGAAATGTTAAACAAAAGTTTTTCCCTTGCATCTTGGTCACTTT

TACTTTTTTGTTATTTCGTAATCTCCAGGGTATGATACCCTATAGCTTTACAGTTACTTCTCATTTTCTC

ATTACTTTGGGTCTTTCATTTTCCATTTTTATTGGCATTACTATAGTGGGATTTCAAAGAAATGGGCTTC

ATTTTTTAAGCTTCTTATTACCTGCAGGAGTCCCACTGCCGTTAGCACCTTTTTTAGTACTCCTTGAGCT

AATCCCTCATTGTTTTCGCGCATTAAGCTCAGGAATACGTTTATTTGCTAATATGATGGCCGGTCATAGT

TCAGTAAAGATTTTAAGTGGGTTCGCTTGGACTATGCTATGTATGAATGATCTTTTATATTTCATAGGAG

ATCTTGGTCCTTTATTTATAGTTCTTGCATTAACCGGTCTTGAATTAGGTGTAGCTATATTACAAGCTCA

TGTTTTTACGATCTTAATCTGTATTTACTTGAATGATGCTACAAATCTCCATCAAA--------------

----------------ATATGTGGGCACCTGATATCTATGAGGGTTCACCCACCCCGGTTACAGCATTCT

TTTCTATTGCGCCTAAAATTTCTATTTCTGCTAATATTTTACGTGTTTTTATTTATGGTTCCTATGGAGC

TACATTGCAACAAATCTTCTTTTTCTGCAGCATTGCTTCTATGATCTTAGGAGCACTGGCCGCCATGGCT

CAAACGAAAGTAAAAAGACTTCTAGCTCATAGTTCAATTGGACATGTAGGTTATATTCGTACTGGTTTCT

CATGTGGAACCATAGAAGGAATTCAATCACTACTAATTGGTCTCTTTATTTATGCATCAATGACGATAGA

TGCATTCGCTATAGTTTCAGCATTACGGCAAACCCGTGTCAAATATATAGCGGATTTGGGCGCTCTAGCC

AAAACGAATCCTATTTCGGCTATTACCTTCTCTATTACTATGTTCTCATACGCAGGAATACCCCCGTTAG

CCGGCTTTTGTAGTAAATTCTATTTGTTCTTCGCCGCTTTGGGTTGTGGGGCTTACTTCCTAGCCCCAGT

GGGAGTAGTGACTAGCGTTATAGGTTGT-TGTTCGATAGCCCGACCGTAGTGATGTTAATTGTGGTTACA

TTCATAAGTAGCTTGGTCCATCTTTATTCTATTTCATATATGTCTGAGGATCCGCATAGCCCTCGATTTA

TGTGTTATTTATCCATTCTTACTTTTTTTATGCCAATGTTGGTGACTGGAGATAACTCTCTTCAATTATT

CTTGGGATGGGAGGGAGTAGGTCTTGCTTCATATTTGTTAATTCATTTTTGGTTTACACGACTTCAGGCA

GATAAAGCAGCTATAAAAGCTATGCTTGTCAATCGAGTAGGTGATTTTGGATTAGCTCTTGGGATTTCGG

GTCGTTTTACTCTCTTTCAAACAGTAGACTTTTCCACCATTTTTGCTTGTGCTAGTGCCCAACGAAATTC

TTGGATTTCTTGCAATATGAGATTGAATGCCATAACTCTTATTTGTATTTTACTTTTTATTGGTGCTGTT

GGAAAATCTGCACAGATAGGATCGCATACTTGGTCACCCGATGCTATGGAGGGTCCCACTCCAGTATCTG

CTTTGATTCATGCAGCTACTATGGTAACAGCTGGCGTTTTCATGATAGCAAGGTGTTCCCCTTTATTTGA

ATACCCACCTACGGCTTTAATTGTTATTACTTTTGCAGGAGCTATGACGTCATTCCTTGCGGCAACCACT

GGAATATTACAGAACGATCTAAAGAGGGTCATAGCTTATTCAACTTGCAGTCAATTAGGCTATATGATCT

TTGCTTGCGGCATTTCTAACTATTCGGTTAGCGTCTTTCATTTAATGAATCACGCCTTTTTCAAAGCATT

ACTATTCCTGAGTGCAGGTTCGGTGATTCATGCCATGTCGGATGAGCAAGATATGCGGAAGATGGGGGGG

CTCGCCTCCTCGTTCCCTTTTACCTATGCCATGATGCTCATGGGCAGCTTATCTCTAATTGGATTTCCTT

TTCTAACTGGATTTTATTCCAAAGATGTGATCTTAGAGCTCGCTTACACTAAGTATACCATCAGTGGGAA

CTTTGCTTTCTGGTTGGGAAGTGTCTCTGTCCTTTTCACTTCTTATTACTCCTTTCGTTCACTTTTTCTA

ACATTTCTAGTACCAACTAATTCATTCGGGCGAGACATCTTACGATGTCATGATGCGCCCATTCCTATGG

CCATTCCTTTAATACTTCTGGCTTTCGGGAGTCTCTTTGTAGGATACTTGGCCAA-CTAACACAAAGAAG

ATACAGTTCACTCAACGATTGCCTTTGGGTTCCGAACTCCATATGGGGAAGGAACGTTGTTGTTTGCGGG

GTCTCGATCATTTACATGGACCCACTTTTCATTCCATTTGTGGGAATTTGATGATCTATAAACCGTCCTT

AACGAACGATCGGCTCATCTT------TGAGCATGATGAATCACTTCGTGCCGACCTGTTGTCAATAAAC

TTTTTGGCCTCATATGAGAATGGAAAACTGGAGCATTTTCTTCATCGGTGGATGAAGAATCGCGAACATA

ATAATTTCTGGTTAAGCATGTTCCCAGAAAAAAGATACTTTCGAGAAACAACGAGCACGACTGAAGTGGC

TATACATACAAATCCATTTACGGATCTATATGCTTCGATTGGAACTGGAAGTTCAAGAACAGGCGGCTGG

TATACTACCATAATGAAACTGCCTTTTATTTTTTTTATTCGGATAGGATTTCTGTTGGCTTCGTTGGGGG

GCTCGCGTAGTTTGTTACGTCAGCTCCAAAGGGAGAAATTGCGTTGGAATCGATAGAGTTACGTAA----

AGTTCATAATTGTATA-CCAATTTTTGGGCCAATTCCCTCTTCGTACTACCAAAAAATGAGATTCTTGCC

GAATCCGAGTTTGCTGCTCCAACCATTACCAAACTAATACCTATTCTGTTTAGTACTTCAGGTGCTTCTG

TTGCGTATAATGTAAATCCCGTAGCGGATCAATTCC---------------AACGAGCCTTTCAAACGTC

TACTTTTTGTAATCGACTCTATAGCTTCTTCAATAAACGCTGGTTCTTCGATCAAGTTTTGAATGACTTT

CTAGTCAGATCGTTCTTGCGTTTCGGATATGAAGTCTCATTCGAAGCTTTAGACAAAGGTGCTATTGAGA

TATTGGGCCCCTATGGTATCTCGTACACATTCCGACGATTGGCCGAGCGAATAAGTCAACTTCAAAGTGG

ATTTGT-TTGTTCCATGATCTATGGGTCTACTGGAGCTACCCACTTCGATCAATTAGCCAAGATTTTGAC

CGGATACGAAATCACTGGTGTTCAATCTAGTGGTATTTTTATGGGGATTCTTTTTATCGCTGTAGGATCC

CTATTCAAGATCACTGCAGTTCCTTTT--GGTCTATGCACATTGCTTTCTCCAGGAGGTTGGCCGCCTAT

CCTAGATCTTCCCATTTCCAAAAGGATCCCGGGCTCAATCTGGTTTAGTATCAAGGTGATTCTCTTTCTC

TTTCTATATATATGGGTCCGTGCAGCATTTCCACGATATCGTTATGATCAATTAATGGGACTTGGCCGGA

AAGTGTTCTTGCCTCTATCATTAGCTCGGGTAGTCGCCGTTTCTGGTGTTTTAGTCACCTTTCAATGGCT

CCCTTAA-TGCCTCAACTAGATAAATTTACGTATTTTACACAATTCTTCTGGTCATGCCTCTTCTTCTTT

ACTTTCTATATTTTAATATGCAATGATAGAGATGGAGTACTTGGGATCAGCAGAATTTTAAAACTACGAA

ACCAACTGCTTTCACACCGGGGGAACAACATCCAAA------GCAAGGACCCCAACAGTTTGGAAGATAT

CTTGAGAAAAGGGTTTAACACAGGTGTATCCTATATGTACTCTAGTTTATTCGAAGTATCCCAATGGTGT

AAGTCCGTCGACTTATTGGGAAAAAGGAAAAAAATCACTTTGATCTCTTGTTTCGGAGAAATAAGTGGCT

CACGAGGAATGGAAAGAAACATATTCTATTTGATCTCGAAGTCTTCATATAGCACTTT------------

---TTCCAATCATGGATGGGGGATCACTTGTAAGAATGACATAATGCTAATCCATGTTCTACACGGCCAA

-----------------------

>Rhododendron x OM283814.1

ATGATACTTTCTGTTTTGTCAAGTCCTGCTTTGGTCTCTGGTTTGATGGTTGTACGTGCTAAAAATCCTG

TACATTCCGTTTTGTTTCTCATTCTAGTCTTTCGCGACACTTCCGGTTTACTTCTTTTGTTAGGTCTCGA

CTTCTTCGCTATGATCTTCCCAGTAGTTCATATAGGAGCTATAGCCGTTTCATTCCTTTTCGTTGTTATG

ATGTTCCATATTCAAATAGCAGAGATTCACGAAGAAGTATTGCGCTATTTACCAGTGAGTGGTATTATTG

GACTGATCTTTTGGTGGGAAATGTTCTTCATTTTAGATAATGAGAGCATCCCATTACTACCAACCCAAAG

AAATACGACCTCTCTGAGATATACGGTTTATGCCGGAAAGGTACGAAGTTGGACTAATTTGGAAACATTG

GGCAATTTACTTTATACCTACTATTCCGTCTGGTTTTTGGTTTCTAGTCTGATTTTATTAGTAGCCATGA

TTGGGGCTATAGTACTGACTATGCATAGGACTACTAA------GGTGAAAAGACAGGATGTATTCCGACG

AAATGCTATTGATTTTAGGAGGACTATAATGAGGAGGACGACAGAC-----TCAATATATGAATTGTTTC

ATTATTCGTTATTTCTGGGTCTTTTCGTTGCATTCACTTACAACAAGAAACAACCACCAGTGTTTGGTGC

AGCACTTGCATTTTGGTGCATTCTTCTTTCTTTCCTTGGTCTTTCGTTCTGTCATATTCCTAATAACTTA

TCTAATTACAACGTATTAACTGCTAATGCACCTTTCTTTTATCAAATCTCAGGGACATGGTCTAATCATG

AGGGTAGTATTTTATCATGGTGTCGGATCCTAAGTTTTTATGGATTTCTTCTTTGTTACCGGGGTCGACC

GCAAAGCCATAATATCTCAAAACGAGGAGGCCATAGAGAAACTCTTTTTTATTCCTTGGTCTTGAACTTC

GTGAAGAACTCCATTCTATCTCTCCCTCGTTACGAACAAAAAACTGGGGCT------GCGCCCCAGTTGT

ACACTCCCTTCGTTCTACGAA---CCCTTGTTGATTCTGAACTTCGTTCGCGAAGGAACCGGACTTTTGA

CGGGCCAGCCCTTTTTTATGCGCCGCTTTACCCTGAAAGGAAAATGAGCTTTGCTCCTCTGGGCGCTAGG

CGCTCCCGTGGTTCGCGAGAAGGAAAAAGGACTCATCCTTTGTTGCATCTGGCACGAGATGATAAAGAGA

GAGCTTCGTCTATCGATGAACAGCGGATTGACGGAGCTCTTGGCATTGCTTTGTTTTTCTCTCTTTTCCT

ATTAGCGAGTTCCGATCCTTTTGTTCGAAATTTCTTCGTTTGTACCGAACCGCTTGCAGAATTAAATCCT

GTTCTACAAGATCCTATATTAGCTATACATCCTCCTTGCATTTATGCCGGAGACGTCGCCAGTGCTATGG

GCTTTGGCTTATGTAGATCAAAAATGATGAATGGGATTGTGGCACTCCACTCGCCGCCAATGCGGAAGGA

TGCCGCCGAAAAGAATGGAACGCTGTTTTGCTCTGCTGGATGCGTCGGATCCCGTATAACAAGCGAGCTC

TTTACCCTAAAATTCAAACATGTGGGCGCAAAATGCTATCCTGCTCTATTCTTACGTAGCAATAGAAGCC

TGCTT---ATGCTGCTTCGGCGGCGCTTTTTCGCCTTCTCTTCGCTCTGGACAGGAGCGCTAGTGGACGC

GGGGAGGGAGCA------GGCGAAGCCTGTCG------TTCGTAATGGAAAGAAAGATACCACTACTTCG

CCTCTTTGTTGGACCGCCGGCGCGAACACAGTGGTCTCTGACCAGGACCAGGAACCAATTCGAATTTGGA

TCTTGACATGTCGGTGGTTTTTAACCGTAGGCATCTTGCCAGGAAGTTGGTGGGCTCATCATGAATTAGG

TCGGGGTGGCTGGTGGTTTCGGGATCCCGTAGAAAATGCTTCTTTTATGCCTCGGGTATTAGCCATAGCT

CGTATTCATTCAGTAATTCTACCCCTTCTTCATTCTTGGACCTCGCTTCTTAATATTGTGACTCTTCCAT

GCTGTGTCTCAGGAACCTCTTCAATACGGTCCGGATTGCTAGCTCCTGTTCATAGTTTTGCTACAGATGA

TACACGAGGAATCTTTTTATGGTGGTTCTTCCTTCTAATGACCGGCATATCTATGATTCTTTTCTCCCAG

ATGAAGCAGCAGGCATCGGTCCGTATAACCCATAAAAAAGAGATGGTTGTGGCGCGAAGTACTCTTGTGC

ACCTACGT-----------------------------------GCTTCTTGTGATGCAGCAGAACCATGG

CAATTAGGATCTCAAGACGCAGCAACACCTATGATGCAAGGAATCATAGACTTACATCACGATATCTTTT

TCTTCCTCATTCTGATTTTGGTTTTCGTATCACGGGTCTTGGTTCGCGCTTTATGGCATTTCCACTATCA

AAAAAATCCAATCCCGCAAAGGATTGTTCATGGAACTACTATCGAGATTATTCGGACCATCTTTCCTAGT

ATCATCCCGATGTTCATTGCTATACCATCATTTGCTCTGTTATACTCAATGGACGAGGTAGTAGTAGATC

CAGCCATTACTATCAAAGCTATTGGACATCAATGGTATCGGACTTATGAGTATTCGGACTATAACAGTTC

CGATGAACAGTCACTCACTTTTGACAGTTATACGATTCCAGAAGATGATCCAGAATTGGGTCAATCACGT

TTATTAGAAGTGGACAATAGAGTCGTTGTACCAGCCAAAACTCATCTACGTATTATTGTCACATCTGCGG

ATGTACTTCATAGTTGGGCTGTACCTTCCTCAGGTGTCAAATGTGATGCTGTACCTGGTGG---------

----------------------------------------------------------------------

----------------------------------------------------------------------

------------------------TGAGACGACTCTTTCTTGAACTATATCATAAACAGATCTTCCCCTC

CACACCAATCACGAGTTTTTCTCCATTCCTCTCGTATATCGTCGTAACGCCCTTAATGCTAGGTTTTGAA

AAAGACTTTTCATGTCATTCCCATTTAGGTCCGATTCGGATCCCTCCGTTGTTTCCTTTTCCTTCCGCAC

CTTTTCCTCGAAATGAGAAAGAAGATGGTACACTTGAATTGTATTATTTAAGTGCTTATTGCTTGCCAAA

GATCCTACTTCTACAATTGGTAGGTCACCGGGTTATTCAAATAAGTCGTGTTTTCCGTGGTTTTCCCATG

TTACAACTTCCGTACCAATTCGGTCGATCCGGAATAGATCGGTTAAACATTCTATTAGGGAGCCTGGTCT

TGACTCTTCTGTGTGGTATTCATTCTCGTTCGGCTCTTGGAATCACATCCAGCAGTGGTTGGAACAGCTC

GCAAAATCCAACCACTTCACCTACTTCATTGCCCCCAACCCTTTCTCGTACCTCTATTGAAACAGAATGG

TTTCATGTTTTTTCATCGATTGGTTATTCCTCTCTGTTCGTATCTCTTTTTCCAATTTCGGTCTCGATTA

GTTCACAAGATTGA-TGTCCGTTTCGTTATTACAACCTTA-----TTTTTTGATGTCAAAGACCAGAAGC

TACGCGCAAATTCTCATTGGATCTCGGTTGTTCTTAACAGCGATGGCTATTCATTTAAGTCTTCGGGTAG

CACCACTAGATCTTCAACAAGGTGGAAATTCTCGTATTCCGTATGTACATGTTCCTGCGGCTCGGATGAG

TATTCTTGTTTATATCGCTACGGCTATAAACACTTTCTTGTTCCTATTAACAAAACATCCCCTTTTTCTT

CGCTCTTCCGGAACCGGTACAGAAATGGGTGCTTTTTCTACGTTGTTTACCTTAGTTACTGGGGGGTTTC

GGGGAAGACCTATGTGGGGCACCTTTTGGGTGTGGGATGCTCGTTTAACCTCTGTATTCATCTCGTTCCT

TATTTACCTGGGTGCACTGCGTTTTCAAAAGCTTCCTGTCGAACCGGCTCCTATTTCAATCCGTGCTGGA

CCGATCGATATACCAATAATAAAGTCTTCAGTCAACTGGTGGAATACATCGCATCAACCTGGGAGCATTA

GCCGATCTGGTACATCAATACATGTTCCTATGCCCATTCCAATCTTGTCTAACTTTGCTAACTCCCCCTT

CTCAACCCGTATCTTGTTTGTTCTGGAAACACGTCTTCCTATTCCATCTTTTCTCGAATCTCCTTTAACG

GAACAAATAGAAGCTCAAGAAGGAATACC-GCAGGCTAGAAAGATGCTATTTGCTGCTATTCTATCTATT

TGTGCATCAAGTTCGAAGAAGATCTCAATCTATAATGAAGAAATGATAGTAGCTCGTTGTTTTATAGGCT

TTATCATATTCAGTCGGAAGAGTTTAGGTAACACTTTCAAAGTGACTCTCGACGGGAGAATCCAGGCTAT

TCAGGAAGAATCGCAGCAATTCCTCAATCCTAACGAAGTAGTTCCTCCGGAATCCAATGAACAACAACGA

TTACTTAGGATCAGCTTGCGAATTTGTGGCACCGTAGTAGAATCATTACCAATGGCACGCTGTGCGCCTA

AGTGCGAAAAGACAGTGCAAGCTTTGTTATGCCGAAACCTAAATGTTAAGTCAGCAACACTTCCAAATGC

CATTTCTTCCCGTCGCATCCGTCTTCAGGACGATCTAGTCACAGGTTTTCACTTCTCAGTGAGTGAAAGA

TTTGTCCCCGGATGTACGTTGAAAGCTTCTATAGTAGAACTCATTCGAGAGGGCTTGGCGGTTTTAAGAA

GGGTTCGGGTGGGGGGTTTCTCTTATGAAAGAGGCGATCAGAATGGTACTCGAATCCATTTACGATCCCG

AGTTTCCAGACACATCGCACTTCCGCTCGGGTCGAGGCTGCCACTCGGCCCTAAGACGGATCAAAGAAGA

GTGGGGAACCTCTCGCTGGTTTTTGGAATTCGACATCAGGAAGTGTTTTCACACCATCGACCGACATCGA

CTCATCTCAATCTTTAAGGAAGAGATCGATGATCCCAAGTTCTTTTACTCCATTCAGAAAGTCTTTTCCG

CCGGACGACTCGTAGGAGGTGAGAAGGGCCCTTACTCCGTTCCACACAGTGTACTACTATCGGCCCTACC

AGGCAACATCTACCTACACAAGCTCGATCAGGAGATAGGGAGGATCCGACAGAAGTACGAAATTCCGATT

GTTCAGAGAATCAGATCGGTTCTATTAAGGACAGGTCGTATTGATGACCAAGAAAACTCTGGAGAAGAAG

C----------------AGAGCGGCGGAACTAACGACTTTATTAGAAAGTCGAATTACCAACTTTTACAC

CCATTTTCAAGTGGATGAGATCGGTCGAGTGGTCTCCGTTGGGGATGGGATTGCACGTGTTTATGGATTG

AACGAGATTCAAGCCGGGGAAATGGTGGAATTTGCCAGCGGTGTGAAAGGAATAGCCTTGAACCTGGAGA

ATGAGAATGTAGGGATTGTTGTCTTTGGTAGTGATACTGCTATTAAAGAAGGAGATCTTGTAAAACGCAC

TGGTTCTATTGTGGATGTTCCCGCGGGAAAGGCTATGCTAGGGCGTGTGGTCGACGGGTTGGGAGTGCCT

ATTGATGGAAGAGGGTCTCTAGGCGATCACGAGCGAAGACGTGTCGAAGTGAAAGCGCCCGGGATTATTG

AACGTAAATCTGTGCACGAGCCTATGCAAACAGGGTTAAAAGCGGTGGATAGCCTGGTTCCTATAGGCCG

TGGTCAACGAGAACTGATAATCGGGGACCGACAAACTGGAAAAACCGCTATTGCTATCGATACCATATTA

AACCAAAAGCAAATGAACTCAAGGGCCACCTCGGAGAGTGAGACATTGTATTGTGTCTATGTAGCAATTG

GGCAGAAACGCTCAACTGTGGCACAATTAGTTCAAATTCTTTCCGAAGCGAATGCTTTGGAATATTCCAT

TCTTGTAGCAGCCACCGCTTCGGATCCTGCTCCTCTGCAATTTCTGGCCCCATATTCTGGCTGTGCCATG

GGGGAATATTTCCGCGATAATGGAATGCACGCATTAATAATCTATGATGATCTTAGTAAACAGGCCGTGG

CATATCGACAAATGTCATTATTGTTACGCCGACCACCAGGCCGTGAGGCTTTCCCGGGGGATGTTTTCTA

TTTACATTCCCGTCTCTTAGAAAGAGCCGCGAAACGATCGGACCAGACAGGCGCAGGTAGCTTGACCGCC

TTACCCGTCATTGAAACACAAGCGGGAGACGTATCGGCCTATATTCCTACTAATGTCATTTCCATTACGG

ATGGACAAATCTGTTTGGAAACAGAGCTCTTTTATCGCGGAATTAGACCTGCTATTAACGTCGGCTTATC

TGTCAGTCGCGTCGGGTCTGCCGCTCAGTTGAAAACGATGAAACAAGTCTGCGGTAGTTTAAAACTGGAA

TTGGCACAATATCGCGAAGTGGCCGCCTTTGCTCAATTTGGCTCAGACCTTGATGCTGCGACTCAGGCAT

TACTCAATAGAGGCGCAAGGCTGACAGAAGTCTTGAAACAACCACAATATGCACCACTTCCCATAGAAAA

ACAAATTCTAGTCATTTATGCCGCTGTCAATGGATTCTGTGATCGAATGCCATTAGACAAAATTTCGCAA

TATGAAAGAACCATTCTAAGTAGTGCAAAAGCAGAATTACTCCAATCTCTTTTAGAAAAAGGTGGGTTAA

CTAACGAAAGAAAAATGGAACTCGATGCATTCTTAAGAGAAAGTGCTTTGCCTTACTTATGATGCAAGAA

-------GCATCCAACGCAAAGCGGCCTTTCATTCCCTTGTTTCGTCGTGGCACACCCCCCCCACAAGCA

CCCCCCGGCTCAGGGGGGACCAGAAAAGGCCTTTCGTTTTCCCCCCTTCGTCGGCCCTTGCCGCCTTCCT

TAACAAGCCCTCGAGCCTCCTTTGCGCCGCCTTCCTCATAGAAGCCGCCGGGTTGACCCCGAAGGCCGAA

TTCTATGGTAGAGAAGGCTTTAATAATAATTGGGCCATGAGAGACTTTATTAAGTATTGCAAAAGAAAGG

GCCTGCTGATAGAGCTGGGCGGGGAGGCGATACTAGTTATCAGGTCAGAGAGACGCCTGGCCCGTAAGCT

GGCCCCCTTTAAAACCCATTACTTAATAAGGATTTGTTACGCGCGATATGCCGACGACTCACTACTGGGA

ATCGTGGGTGCCGTAGAGCTTCTCATAGAAATACAAAAACGTATCGCCCACTTCCTACAATCCGGACTGA

ACCTTTGGGTAAGCTCTGCAGGATCAACAACAATAACTGCACGGAGTACGGTAGAATTCCTCGGTACGAT

CATTCGGGAAGTCCCTCCGAGGACGACTCCCATACAATTCTTGCGAGAGCTGGAGAAGCGTCTACGGGTA

AAGCACCGTATCCATATAACTGCTTGCCACCTACGCTCCGCCATCCATTCCAAGTTTAGGAACCTAGGTA

ATAGTATCCCGATCAAACAGCTGACGAAGGGGATGAGCGGAACAGGGAGTCTACTGGACGCGGTTCCACT

AGCGGAGACTCTTGGAACAGCTGGAGTAATAAGTCCCCAAGTGAGCGTATTATGGGGGACCGTCAAGCAC

ATCCGGCAAGGATCAAGGTGGATCTCGTTGTTGCATAGCTCAGGTCGGAGCAAGGTGCCATCGGACGTTC

AACAGGCAGTCTCACGATCGGGCATGAGTGCCCGGAAGTTGTC---ATTGTATACTCCCGCGGGTCGGAA

GGCGGCGGGGAAAGGAGGGGGACACTGGGCGAGATCTATCAGCAGCGAATTCCCCATACAAATAGAGGCG

CCTATCAAAAAGATACTCCGAAGGCTTCGGGATCGAGGTATCATTAGCCGAAGAAGACCCTGGCCAATCC

ATGTGGCCTGCTTGACGAACGTCAGCGACGGAGACATAGTAAATTGGTCCGCGGACATTGCGATAAGTCC

TCTGTCCTACTACAGGTGCCGCGACAACCTTTACCAAGTCCGAACGATTGTCGACCACCAGATCCGCTGG

TCTGCAATATTCACCCCGGCCCACAAGCACAAATCCTCGGCGCGGAATATAATCCCAAAGTACTCCAAAG

ACTCAAATATAATAAATAAAGAAGGTGGGAAGACCCTTGCAGAGTTCCCCAACAGCATAGAGCTTGGGAA

GCTCGGACCCGGTCAAGATCC-GAACAACAAGGAGCACTCAACTATTA--------GTCTAGTCT-TGGT

CCAACTACATAACTTTTTCTTTTTCATTACTTCCATGGTCGTGCCTCGTGGCACGGCAGCACCCGTACTA

TTGAAATGGTTCGTCAGTAGAGATGTTCCCACAGGTGCCCCTTTTTCCAATGGTACTATAATTCCTATTC

CTATCCCTCCATTCCCTCTTTTGGTCTATCTACATTCCAGGAAATTCATATGCTCCACGGACGGAGCAAA

AAGTGCAGTCTTGGTCAGAGCAAGCCACCCTATTCTAT------TACCAGACATAATTGGGAGAAGCTCA

TCCGAAACTAGAGCAAGAAACGCCTCATTTTGTTTTGTTCCTGTTCTTCATTTCCTTCTTTTTCAATCCA

A------GGGGGACTTCTCATATTTAGAATCTTTCTGCGGTGTGCTCCGTTTACTATTCTTTCGTACTTT

CTTCTTTTTACCACGCGATAGGTCAGCGAAGCGTGAGCGGGCGCGGAGAAGTAAAGGCCAAACACTTCGG

CCTAACGGGAATGGGAATGAGCAACGACGAAATTACAAGATGAGGTGCCCCGGGCATCCCCATT------

TAGA---AAGAAGGATCGAAGGTTTTGGGCCTGTAGCTTTTCCCGTCCCCCCTTCGTCGGGTGGTCCTTG

TGTGGGGGGTGCGCCACCAGAAATCGGGCTTGAAGCTCTCGCCTTACCAACGAGCCGACAGCTGATGGCT

GTTGGTCACGACTACTACCAAAAAGCTCCAAAAAAAATGAATATTTCACATGGAGGAGTATGCATCTGTA

TGTTGGGTGTTCTTCTGT---TACATAGCTGTTCCAGCGGAAATACTTGGAATAATTCTACCACTTCTAC

TAGGAGTAGCCTTTTTAGTGCTAGCTGAACGTAAAGTAATGGCTTTTGTGCAACGTCGAAAGGGTCCTGA

TGTAGTGGGATCGTTTGGATTGTTACAACCTATAGCAGATGGTTTGAAATTGATTCTAAAAGAACCTATT

TCACCAAGTAGTGCTAATTTCTCCCTTTTTAGAATGGCTCCAGTGGCTACATTTATGTTAAGTCTGGTCG

CTCGGGCCGTTGTACCTTTTGATTATGGTATGGTATTGTCAGATCCGAACATAGGGCTACTTTATTTGTT

TGCCATATCTTCGCTAGGTGTTTATGGAATTATTATAGCAGGTCGGTCTAGTA-TTATTATATACGCTTA

GTGAAAAGAATGTTTTTTGATACACCTAGGACATGGATTCTATATGAACCAATGGATCGTAACAAGTCGT

TACTACTAGCAATGACTTCCTCTTTCATTACTTCATTCTTTCCATATCCCTCTCCTTTGTTCTCAGTTAC

TCATCAAATGGCACTCAGTTCATATCTTTAA------GAATTTGCACCTATTTGTATCTATTTAGTGATT

AGTCTGCTAGTTTCTTTGATCTTACTCGGTGTTCCTTTTCTATTTTCTTCCAATAGTTCGACCTACCCAG

AAAAATTGTCGGCCTACGAATGTGGTTTCGATCCTTTCGGTGATGCCAGAAGTCGCTTTGATATAAGATT

TTATCTTGTTTCCATTTTATTTATTATCTTTGATCTGGAAGTCACCTTTTTCTTTCCTTGGGCAGTCTCT

CTCAACAAGATTGATCTGTTTGGATTTTGGTCCATGATGGCCTTTTTATTGATTTTGACGATTGGATTTC

TCTATGAATGGAAAAGGGGTGCTTTGGATTGGGAATA--AAAGTGTTTCTTACGATTACGCCCAACAGCC

CACTTGAGCAATTTGCCATTCTCCCATTAATTCCTATGAATATAGGAAACTTGTATTTCTCATTCACAAA

TCCATCTTTGTTTATGCTACTCACTCTCAGTTTGGTCCTACTTCTGGTTCATTTTGTTACTAAAAACGGA

GGAGGAAACTCAGTACCAAATGCTTGGCAATCCTTGGTAGAGCTTATTTATGATTTCGTGCCGAACCCGG

TAAACGAACAAATAGGTGGTCTTTCCGGAAATGTTAAACAAAAGTTTTCCCCTCGCATCTCGGTCACTTT

TACTTTTTCGTTATTTCGTAATCTCCAGGGTATGATACCTTATAGCTTCACAGTTACAAGTCATTTTCTC

ATTACTTTGGGTCTCTCATTTTCTCTTTTTATTGGCATTACTATAGTGGGATTTCAAAGAAATGGGCTTC

ATTTTTTAAGCATCTCATTACCCGCAGGAGTCCCACTGCCGTTAGCACCTTTTTTAGTACTCCTTGAGCT

AATCCCTCATTGTTTTCGCGCATTAAGCTCAGGAATACGTTTATTTGCTAATATGATGGCCGGTCATAGT

TCAGTAAAGATTTTAAGTGGGTCCGCTTGGACTATGCTATGTATGAATGATCTTTTATATTTCATAGGAG

ATCTTGGTCCTTTATTTATAGTTCTTGCATTAACCGGTCCGGAATTAGGTGTTGCTATATCACAAGCTCA

TGTTTCTACGATCTTAATCTGTATTTACTTGAATGATGCTACAAATCTCCATCAAA--------------

-----------------TATGTGGGCACCTGATATCTATGAGGGTTCACCCACCCCGGTTACAGCATTCC

TTTCTATTGCACCTAAAATCTCTATTTCTGCTAATATTTCACGTGTTTCTATTTATGGTTCCTATGGAGC

TACATTGCAACAAATCTTCTTTTTCTGCAGCATTGCTTCTATGATCTTAGGAGCACTGGCCGCCATGGCC

CAAACGAAAGTCAAAAGACTTCTAGCTTATAGTTCAATTGGACATGTAGGTTATATTCGTACAGGTTTCT

CATGTGGAACCATAGAAGGAATTCAATCACTACTAATTGGTATCTTTATTTATGCATCAATGACGATAGA

TGCATTCGCCATAGTTTCAGCATTACGGCAAACCCGTGTCAAATATATAGCGGATTTGGGCGCTCTAGCC

AAAACGAATCCTATTTCGGCTATTACCTTCTCCATTACTATGTTCTCATACGCAGGAATACCCCCGTTAG

CCGGCTTTTGTAGCAAATTCTATTTGTTCTTCGCCGCTTTGGGTTGTGGGGCTTACTTCCTAGCCCCAGT

GGGAGTAGTGACTAGCGTTATAGGTCGTTTGTTCGATAGCCTGACCGTAGTGATGTTAATTGTGGTTACA

TTCATAAGTAGCTTGGTCCATCTTTATTCCATTTCATATATGTCTGAGGATCCGCATAGCCCTCGATTTA

TGTGTTATTTATCCATTTTTACTTTTTTTATGCTAATGTTGGTGACTGGAGATAACTTTCTTCAATTATT

CCTGGGATGGGAGGGAGTAGGTCTTGCTTCATATTTGTTAATTCATTTCTGGTTTACACGACTTCAGGCA

GATAAAGCAGCTATCAAAGCTATGCTTGTCAATCGAGTAGGTGATTTTGGATTAGCCCTTGGTATTTTTG

GTTGTTTTACTCTCTTTCAAACAGTAGACTTTTCCACCATTTTTGCTTGTGCTAGTGTCCCCAGAAATTC

TTGGATTTTTTGCAATATGAGATTCAATGCCATAACTCTTATTTGTATTTTACTTTTTATTGGTGCTGTT

GGGAAATCTGCACAGATAGGATTGCATACTTGGTTACCTGATGCTATGGAGGGTCCCACTCCAGTATCCG

CTTTGATTCATGCAGCTACTATGGTAACAGCTGGCGTTTTCATGATAGCAAGGTGCTCCCCTTTATTTGA

ATACTCACCTACGGCTTTGGTTGTTATTACTTTTGCAGGAGCTATGACGTCATTCCTTGCGGCAACCACT

GGAATATTACAGAACGATCTAAAGAGGGTCATAGCTTATTCAACTTGCAGTCAATTAGGCTATATGATCT

TTGCTTGCGGCATCTCTAACTATTCGGTTAGCGTCTTTCACTTAATGAATCACGCGTTTTTCAAAGCATT

ACTATTCCTGAGTGCAGGTTCGGTAATTCATGCCATGTCGGATGAGCAAGATATGCGGAAGATGGGGGGG

CTTGCCTCCTCGTTCCCTTTTACCTATGCCATGATGCTCATGGGCAGCTTATCTCTAATTGGATTTCCTT

TTCTAACTGGATTTTATTCCAAAGATGTGATCTTAGAGCTCGCTTACACTAAGTATACCATCAGTGGGAA

CTTTGCTTTCTGGTTGGGAAGTGTCTCTGTCCTTTTCACTTCTTATTACTCTTTTCGTTTACTTTTTCTA

ACATTTCTAGTACCAACTAATTCATTCGGGCGAGACAGATTACGATGTCATGATGCGCCCATTCCTATGG

CCATTCCTTTAATACTTCTGGCTCTCGGAAGTCTCTTTGTAGGATACTTGGCCAA--TAACACAAAGAAG

ATACAGTTCACTCAACGATTGCCTTTGGGTTCCGAACTCCATATGGGGAAGGAGCGTTGTTGTTTGCGAG

GTCTCGATCATTTACATGGACCCACTTTTCATTCTATTTGTGGGAATTTGATGATCTATAAACCGTCCTT

AACGAACGATCGGCTGATGTT------TGAGCATGATGAATCATTTCGTGCCGACCTGTTGCCAATAAAC

TTTCCGGCCTCATATGAGAATGGGAAACTGGAGCATTTTCTGCATCGGTGGATGAAGAATCGCGAGCATA

ATAATTTCTGGTTGACCATGTTCCCAGAAAAAAGATACTTTCGAGAAACGACGAGTACGACGGAAGTGGC

TATACATACAAATCCAGTTACGGATCTATATGCTTCGATTGGAACTGGAAGTTCCAGAACAGGCGGCTGG

TATACCACCATAATGAAACTGCCTTTTCTTTTTTTTATTTGGATAGGATTTATGTTGGCTTCGTTGGGAG

GCTCGCGTAGTTTGTTACGTCAGCTCCAAAAGGATAAGTTGCGTTGGAATCGAGAAAGTTCCGTGG----

AGTTAATAATTGCATAA-CCATTTTTGGGCCAATTCCCTCTTCGTACTACCAAAAAATGAGATTCTTGCC

GAATCCGAGTTTGCTGCTCCAACCATTATAAAACTAATACCTATTCTGTTTAGTACTTTAGGTGCTTTTG

TTGCGTATAATGTAAATCTCGTAGCGGATCAATTCC---------------AACGAGCCTTTCAAACTAG

TACTTTTTGTAATCGACTCTATAGCTTCTTCAATAAACGCTGGTTCTTCGATCAAGTTTTGAATGACTTT

ATAGTCAGATCGTTCCTGCGTTTCGGATATGAAGTCTCATTCGAAGCTTTAGACAAAGGTGCTATTGAGA

TATTGGGGCCTTATGGTATCTCGTACACATTCCGACGATTGGCCGAGCGAATAAGTCAACTTCAAAGTGG

ATTTGTT-TGTTCCATGATCTATGGGTCTACTGGAGCTACCCACTTCGATCAATTAGCCAAGATTTTGAC

CGGATACGAAATCACAGGTGCTCGATCTAGTGGTATTTTTATGGGGATTCTATCTATCGCTGTAGGATCC

CTATTCAAGATCACTGCAGTTCCTTTTCTGGTCTATGCACATTGCTCTTTCTAGGAGGTTGGCTGCCTAT

CCTAGATCTTCCTATTTTCAAGAAGATCCCGGGCTCGATCTGGTTTAGTATCAAGGTGATTCTTTTTCTG

TTTCTATATATATGGGTCCGTGCAGCATTTCCACGATATCGTTATGATCAATTAATGGGACTTGGCCGGA

AAGTGTTCTTGCCTCTATCATTAGCTCGGGTAGTCGCCGTTTCTGGTGTTTTAGTCACCTTTCAATGGCT

CCCTTA--TGCCTCAACTGGATAAATTCACTTATTTCACACAATTCTTCTGGTCATGCCTTTTCCTCTTG

ACTTTCTATATTCCCATATGCAATGATGGAGATGGAGTACTTGGGATCAGCAGAATTCTCAAACTACGGA

ACCAACTGGTTTCACACCGGGAGAACAACATGCGGA------GCAACGACCCCAAGAGTTTGGAGGATAT

CTTGAGAAAAGGTTTTAGCACCGGTGTATCCTATATGTACTCCAGTTTATTCGAAGTATCTCAATGGTGT

AAGGCCGTCGACTTATTGGGAAAAAGGAGGAAAATCACTTTGATCTCTTGTTTCGGAGAAATAAGTGGCT

CACGAGGAATGGAAAGAAACATATTCTATTTGATCTCGAAGTCCTCATATAGCACTTC------------

---TTCTAATCCTGAATGGGGGATCACTTGTAGGAATGACATAATGCTAATCCATGTTCCACACGGCCAA

GGAAGCATCGTTTTTT-------

>Rhododendron simsii NC053763.1

-TGATACTTTCTGTTTTGTCAAGTCCTGCTTTGGTCTCTGGTTTGATGGTTGTACGTGCTAAAAATCCTG

TACATTCCGTTTTGTTTCTCATTCTAGTCTTTCGCGACACTTCCGGTTTACTTCTTTTGTTAGGTCTCGA

CTTCTTCGCTATGATCTTCCCAGTAGTTCATATAGGAGCTATAGCCGTTTCATTCCTTTTCGTTGTTATG

ATGTTCCATATTCAAATAGCAGAGATTCACGAAGAAGTATTGCGCTATTTACCAGTGAGTGGTATTATTG

GACTGATCTTTTGGTGGGAAATGTTCTTCATTTTAGATAATGAGAGCATCCCATTACTACCAACCCAAAG

AAATACGACCTCTCTGAGATATACGGTTTATGCCGGAAAGGTACGAAGTTGGACTAATTTGGAAACATTG

GGCAATTTACTTTATACCTACTATTCCGTCTGGTTTTTGGTTTCTAGTCTGATTTTATTAGTAGCCATGA

TTGGGGCTATAGTACTGACTATGCATAGGACTACTAA------GGTGAAAAGACAGGATGTATTCCGACG

AAATGCTATTGATTTTAGGAGGACTATAATGAGGAGGACGACAGACC----TCAATATATGAATTGTTTC

ATTATTCGTTATTTCTGGGTCTTTTCGTTGCATTCACTTACAACAAGAAACAACCACCAGTGTTTGGTGC

AGCACTTGCATTTTGGTGCATTCTTCTTTCTTTCCTTGGTCTTTCGTTCTGTCATATTCCTAATAACTTA

TCTAATTACAACGTATTAACTGCTAATGCACCTTTCTTTTATCAAATCTCAGGGACATGGTCTAATCATG

AGGGTAGTATTTTATCATGGTGTCGGATCCTAAGTTTTTATGGATTTCTTCTTTGTTACCGGGGTCGACC

GCAAAGCCATAATATCTCAAAACGAGGAGGCCATAGAGAAACTCTTTTTTATTCCTTGGTCTTGAACTTC

GTGAAGAACTCCATTCTATCTCTCCCTCGTTACGAACAAAAAACTGGGGCT------GCGCCCCAGTTGT

ACACTCCCTTCGTTCTACGAA---CCCTTGTTGATTCTGAACTTCGTTCGCGAAGGAACCGGACTTTTGA

CGGGCCAGCCCTTTTTTATGCGCCGCTTTACCCTGAAAGGAAAATGAGCTTTGCTCCTCTGGGCGCTAGG

CGCTCCCGTGGTTCGCGAGAAGGAAAAAGGACTCATCCTTTGTTGCATCTGGCACGAGATGATAAAGAGA

GAGCTTCGTCTATCGATGAACAGCGGATTGACGGAGCTCTTGGCATTGCTTTGTTTTTCTCTCTTTTCCT

ATTAGCGAGTTCCGATCCTTTTGTTCGAAATTTCTTCGTTTGTACCGAACCGCTTGCAGAATTAAATCCT

GTTCTACAAGATCCTATATTAGCTATACATCCTCCTTGCATTTATGCCGGAGACGTCGCCAGTGCTATGG

GCTTTGGCTTATGTAGATCAAAAATGATGAATGGGATTGTGGCACTCCACTCGCCGCCAATGCGGAAGGA

TGCCGCCGAAAAGAATGGAACGCTGTTTTGCTCTGCTGGATGCGTCGGATCCCGTATAACAAGCGAGCTC

TTTACCCTAAAATTCAAACATGTGGGCGCAAAATGCTATCCTGCTCTATTCTTACGTAGCAATAGAAGCC

TGCTT---ATGCTGCTTCGGCGGCGCTTTTTCGCCTTCTCTTCGCTCTGGACAGGAGCGCTAGTGGACGC

GGGGAGGGAGCA------GGCGAAGCCTGTCG------TTCGTAATGGAAAGAAAGATACCACTACTTCG

CCTCTTTGTTGGACCGCCGGCGCGAACACAGTGGTCTCTGACCAGGACCAGGAACCAATTCGAATTTGGA

TCTTGACATGTCGGTGGTTTTTAACCGTAGGCATCTTGCCAGGAAGTTGGTGGGCTCATCATGAATTAGG

TCGGGGTGGCTGGTGGTTTCGGGATCCCGTAGAAAATGCTTCTTTTATGCCTCGGGTATTAGCCATAGCT

CGTATTCATTCAGTAATTCTACCCCTTCTTCATTCTTGGACCTCGCTTCTTAATATTGTGACTCTTCCAT

GCTGTGTCTCAGGAACCTCTTCAATACGGTCCGGATTGCTAGCTCCTGTTCATAGTTTTGCTACAGATGA

TACACGAGGAATCTTTTTATGGTGGTTCTTCCTTCTAATGACCGGCATATCTATGATTCTTTTCTCCCAG

ATGAAGCAGCAGGCATCGGTCCGTATAACCCATAAAAAAGAGATGGTTGTGGCGCGAAGTACTCTTGTGC

ACCTACGT--------------------------ATTCCTACAGCTTCTTGTGATGCAGCAGAACCATGG

CAATTAGGATCTCAAGACGCAGCAACACCTATGATGCAAGGAATCATAGACTTACATCACGATATCTTTT

TCTTCCTCATTCTGATTTTGGTTTTCGTATCACGGGTCTTGGTTCGCGCTTTATGGCATTTCCACTATCA

AAAAAATCCAATCCCGCAAAGGATTGTTCATGGAACTACTATCGAGATTATTCGGACCATCTTTCCTAGT

ATCATCCCGATGTTCATTGCTATACCATCATTTGCTCTGTTATACTCAATGGACGAGGTAGTAGTCGATC

CATCCATTACTATCAAAGCTATTGGACATCAATGGTATCGGACTTATGAGTATTCGGACTATAACAGTTC

CGATGAACAGTCACTCACTTTTGACAGTTATACGATTCCAGAAGATGATCCAGAATTGGGTCAATCACGT

TTATTAGAAGTGGACAATAGAGTCGTTGTACCAGCCAAAACTCATCTACGTATTATTGTCACATCTGCGG

ATGTACTTCATAGTTGGGCTGTACCTTCCTCAGGTGTCAAATGTGATGCTGTACCTGGTCGTTTAAATCA

GACCTCTATTTCGGTACAACGAGAAGGAGTTTACTATGGTCAGTGCAGTGAGATTTGTGGAACGAATCAT

GCCTTTACGCCTATCGTCGTAGAAGCTGTTCCTAGGAAAGATTACGGTTCTCGGGTATCTAATCAATTAA

TCCCCCAAACC-------------TGAGACGACTCTTTCTTGAACTATATCATAAACAGATCTTCCCCTC

CACACCAATCACGAGTTTTTCTCCATTCCTCTCGTATATCGTCGTAACGCCCTTAATGCTAGGTTTTGAA

AAAGACTTTTCATGTCATTCCCATTTAGGTCCGATTCGGATCCCTCCGTTGTTTCCTTTTCCTTCCGCAC

CTTTTCCTCGAAATGAGAAAGAAGATGGTACACTTGAATTGTATTATTTAAGTGCTTATTGCTTGCCAAA

GATCCTACTTCTACAATTGGTAGGTCACCGGGTTATTCAAATAAGTCGTGTTTTCCGTGGTTTTCCCATG

TTACAACTTCCGTACCAATTCGGTCGATCCGGAATAGATCGGTTAAACATTCTATTAGGGAGCCTGGTCT

TGACTCTTCTGTGTGGTATTCATTCTCGTTCGGCTCTTGGAATCACATCCAGCAGTGGTTGGAACAGCTC

GCAAAATCCAACCACTTCACCTACTTCATTGCCCCCAACCCTTTCTCGTACCTCTATTGAAACAGAATGG

TTTCATGTTTTTTCATCGATTGGTTATTCCTCTCTGTTCGTATCTCTTTTTCCAATTTCGGTCTCGATTA

GTTCACAAGATTGAATGTCCGTTTCGTTATTACAACCTTA-----TTTTTTGATGTCAAAGACCAGAAGC

TACGCGCAAATTCTCATTGGATCTCGGTTGTTCTTAACAGCGATGGCTATTCATTTAAGTCTTCGGGTAG

CACCACTAGATCTTCAACAAGGTGGAAATTCTCGTATTCCGTATGTACATGTTCCTGCGGCTCGGATGAG

TATTCTTGTTTATATCGCTACGGCTATAAACACTTTCTTGTTCCTATTAACAAAACATCCCCTTTTTCTT

CGCTCTTCCGGAACCGGTACAGAAATGGGTGCTTTTTCTACGTTGTTTACCTTAGTTACTGGGGGGTTTC

GGGGAAGACCTATGTGGGGCACCTTTTGGGTGTGGGATGCTCGTTTAACCTCTGTATTCATCTCGTTCCT

TATTTACCTGGGTGCACTGCGTTTTCAAAAGCTTCCTGTCGAACCGGCTCCTATTTCAATCCGTGCTGGA

CCGATCGATATACCAATAATAAAGTCTTCAGTCAACTGGTGGAATACATCGCATCAACCTGGGAGCATTA

GCCGATCTGGTACATCAATACATGTTCCTATGCCCATTCCAATCTTGTCTAACTTTGCTAACTCCCCCTT

CTCAACCCGTATCTTGTTTGTTCTGGAAACACGTCTTCCTATTCCATCTTTTCTCGAATCTCCTTTAACG

GAACAAATAGAAGCTCAAGAAGGAATAC-TGCAGGCTAGAAAGATGCTATTTGCTGCTATTCTATCTATT

TGTGCATCAAGTTCGAAGAAGATCTCAATCTATAATGAAGAAATGATAGTAGCTCGTTGTTTTATAGGCT

TTATCATATTCAGTCGGAAGAGTTTAGGTAACACTTTCAAAGTGACTCTCGACGGGAGAATCCAGGCTAT

TCAGGAAGAATCGCAGCAATTCCTCAATCCTAACGAAGTAGTTCCTCCGGAATCCAATGAACAACAACGA

TTACTTAGGATCAGCTTGCGAATTTGTGGCACCGTAGTAGAATCATTACCAATGGCACGCTGTGCGCCTA

AGTGCGAAAAGACAGTGCAAGCTTTGTTATGCCGAAACCTAAATGTTAAGTCAGCAACACTTCCAAATGC

CATTTCTTCCCGTCGCATCCGTCTTCAGGACGATCTAGTCACAGGTTTTCACTTCTCAGTGAGTGAAAGA

TTTGTCCCCGGATGTACGTTGAAAGCTTCTATAGTAGAACTCATTCGAGAGGGCTTGGCGGTTTTAAGAA

GGGTTCGGGTGGGGGGTTTCTCT-ATGAAAGAGGCGATCAGAATGGTACTCGAATCCATTTACGATCCCG

AGTTTCCAGACACATCGCACTTCCGCTCGGGTCGAGGCTGCCACTCGGCCCTAAGACGGATCAAAGAAGA

GTGGGGAACCTCTCGCTGGTTTTTGGAATTCGACATCAGGAAGTGTTTTCACACCATCGACCGACATCGA

CTCATCTCAATCTTTAAGGAAGAGATCGATGATCCCAAGTTCTTTTACTCCATTCAGAAAGTCTTTTCCG

CCGGACGACTCGTAGGAGGTGAGAAGGGCCCTTACTCCGTTCCACACAGTGTACTACTATCGGCCCTACC

AGGCAACATCTACCTACACAAGCTCGATCAGGAGATAGGGAGGATCCGACAGAAGTACGAAATTCCGATT

GTTCAGAGAATCAGATCGGTTCTATTAAGGACAGGTCGTATTGATGACCAAGAAAACTCTGGAGAAGAAG

C----------------AGAGCGGCGGAACTAACGACTTTATTAGAAAGTCGAATTACCAACTTTTACAC

CCATTTTCAAGTGGATGAGATCGGTCGAGTGGTCTCCGTTGGGGATGGGATTGCACGTGTTTATGGATTG

AACGAGATTCAAGCCGGGGAAATGGTGGAATTTGCCAGCGGTGTGAAAGGAATAGCCTTGAACCTGGAGA

ATGAGAATGTAGGGATTGTTGTCTTTGGTAGTGATACTGCTATTAAAGAAGGAGATCTTGTCAAGCGCAC

TGGATCTATTGTGGATGTTCCCGCGGGAAAGGCTATGCTAGGGCGTGTGGTCGACGGGTTGGGAGTGCCT

ATTGATGGAAGAGGGTCTCTAGGCGATCACGAGCGAAGACGTGTCGAAGTGAAAGCGCCCGGGATTATTG

AACGTAAATCTGTGCACGAGCCTATGCAAACAGGGTTAAAAGCGGTGGATAGCCTGGTTCCTATAGGCCG

TGGTCAACGAGAACTGATAATCGGGGACCGACAAACTGGAAAAACCGCTATTGCTATCGATACCATATTA

AACCAAAAGCAAATGAACTCAAGGGCCACCTCGGAGAGTGAGACATTGTATTGTGTCTATGTAGCAATTG

GGCAGAAACGCTCAACTGTGGCACAATTAGTTCAAATTCTTTCCGAAGCGAATGCTTTGGAATATTCCAT

TCTTGTAGCAGCCACCGCTTCGGATCCTGCTCCTCTGCAATTTCTGGCCCCATATTCTGGCTGTGCCATG

GGGGAATATTTCCGCGATAATGGAATGCACGCATTAATAATCTATGATGATCTTAGTAAACAGGCCGTGG

CATATCGACAAATGTCATTATTGTTACGCCGACCACCAGGCCGTGAGGCTTTCCCGGGGGATGTTTTCTA

TTTACATTCCCGTCTCTTAGAAAGAGCCGCGAAACGATCGGACCAGACAGGCGCAGGTAGCTTGACCGCC

TTACCCGTCATTGAAACACAAGCGGGAGACGTATCGGCCTATATTCCTACTAATGTCATTTCCATTACGG

ATGGACAAATCTGTTTGGAAACAGAGCTCTTTTATCGCGGAATTAGACCTGCTATTAACGTCGGCTTATC

TGTCAGTCGCGTCGGGTCTGCCGCTCAGTTGAAAACGATGAAACAAGTCTGCGGTAGTTTAAAACTGGAA

TTGGCACAATATCGCGAAGTGGCCGCCTTTGCTCAATTTGGCTCAGACCTTGATGCTGCGACTCAGGCAT

TACTCAATAGAGGCGCAAGGCTGACAGAAGTCTTGAAACAACCACAATATGCACCACTTCCCATAGAAAA

ACAAATTCTAGTCATTTATGCCGCTGTCAATGGATTCTGTGATCGAATGCCATTAGACAAAATTTCGCAA

TATGAAAGAACCATTCTAAGTAGTGCAAAAGCAGAATTACTCCAATCTCTTTTAGAAAAAGGTGGGTTAA

CTAACGAAAGAAAAATGGAACTCGATGCATTCTTAAGAGAAAGTGCTTTGCCTTACTTATGATGCAAGAA

-------GCATCCAACGCAAAGCGGCCTTTCATTCCCTTGTTTCGTCGTGGCACACCCCCCCCACAAGCA

CCCCCCGGCTCAGGGGGGACCAGAAAAGGCCTTTCGTTTTCCCCCCTTCGTCGGCCCTTGCCGCCTTCCT

TAACAAGCCCTCGAGCCTCCTTTGCGCCGCCTTCCTCATAGAAGCCGCCGGGTTGACCCCGAAGGCCGAA

TTCTATGGTAGAGAAGGCTTTAATAATAATTGGGCCATGAGAGACTTTATTAAGTATTGCAAAAGAAAGG

GCCTGCTGATAGAGCTGGGCGGGGAGGCGATACTAGTTATCAGGTCAGAGAGACGCCTGGCCCGTAAGCT

GGCCCCCTTTAAAACCCATTACTTAATAAGGATTTGTTACGCGCGATATGCCGACGACTCACTACTGGGA

ATCGTGGGTGCCGTAGAGCTTCTCATAGAAATACAAAAACGTATCGCCCACTTCCTACAATCCGGACTGA

ACCTTTGGGTAAGCTCTGCAGGATCAACAACAATAACTGCACGGAGTACGGTAGAATTCCTCGGTACGAT

CATTCGGGAAGTCCCTCCGAGGACGACTCCCATACAATTCTTGCGAGAGCTGGAGAAGCGTCTACGGGTA

AAGCACCGTATCCATATAACTGCTTGCCACCTACGCTCCGCCATCCATTCCAAGTTTAGGAACCTAGGTA

ATAGTATCCCGATCAAACAGCTGACGAAGGGGATGAGCGGAACAGGGAGTCTACTGGACGCGGTTCCACT

AGCGGAGACTCTTGGAACAGCTGGAGTAATAAGTCCCCAAGTGAGCGTATTATGGGGGACCGTCAAGCAC

ATCCGGCAAGGATCAAGGTGGATCTCGTTGTTGCATAGCTCAGGTCGGAGCAAGGTGCCATCGGACGTTC

AACAGGCAGTCTCACGATCGGGCATGAGTGCCCGGAAGTTGTC---ATTGTATACTCCCGCGGGTCGGAA

GGCGGCGGGGAAAGGAGGGGGACACTGGGCGAGATCTATCAGCAGCGAATTCCCCATACAAATAGAGGCG

CCTATCAAAAAGATACTCCGAAGGCTTCGGGATCGAGGTATCATTAGCCGAAGAAGACCCTGGCCAATCC

ATGTGGCCTGCTTGACGAACGTCAGCGACGGAGACATAGTAAATTGGTCCGCGGACATTGCGATAAGTCC

TCTGTCCTACTACAGGTGCCGCGACAACCTTTACCAAGTCCGAACGATTGTCGACCACCAGATCCGCTGG

TCTGCAATATTCACCCCGGCCCACAAGCACAAATCCTCGGCGCGGAATATAATCCCAAAGTACTCCAAAG

ACTCAAATATAATAAATAAAGAAGGTGGGAAGACCCTTGCAGAGTTCCCCAACAGCATAGAGCTTGGGAA

GCTCGGACCCGGTCAAGATCC-GAACAACAAGGAGCACTCAACTATTA--------GTCTAGTCTATGGT

CCAACTACATAACTTTTTCTTTTTCATTACTTCCATGGTCGTGCCTCGTGGCACGGCAGCACCCGTACTA

TTGAAATGGTTCGTCAGTAGAGATGTTCCCACAGGTGCCCCTTTTTCCAATGGTACTATAATTCCTATTC

CTATCCCTCCATTCCCTCTTTTGGTCTATCTACATTCCAGGAAATTCATATGCTCCACGGACGGAGCAAA

AAGTGCAGTCTTGGTCAGAGCAAGCCACCCTATTCTAT------TACCAGACATAATTGGGAGAAGCTCA

TCCGAAACTAGAGCAAGAAACGCCTCATTTTGTTTTGTTCCTGTTCTTCATTTCCTTCTTTTTCAATCCA

A------GGGGGACTTCTCATATTTAGAATCTTTCTGCGGTGTGCTCCGTTTACTATTCTTTCGTACTTT

CTTCTTTTTACCACGCGATAGGTCAGCGAAGCGTGAGCGGGCGCGGAGAAGTAAAGGCCAAACACTTCGG

CCTAACGGGAATGGGAATGAGCAACGACGAAATTACAAGATGAGGTGCCCCGGGCATCCCCATT------

TAGA---AAGAAGGATCGAAGGTTTTGGGCCTGTAGCTTTTCCCGTCCCCCCTTCGTCGGGTGGTCCTTG

TGTGGGGGGTGCGCCACCAGAAATCGGGCTTGAAGCTCTCGCCTTACCAACGAGCCGACAGCTGATGGCT

GTTGGTCACGACTACTACCAAAAAGCTCCAAAAAAAATGAATATTTCACATGGAGGAGTATGCATCTGTA

TGTTGGGTGTTCTTCTG----TACATAGCTGTTCCAGCGGAAATACTTGGAATAATTCTACCACTTCTAC

TAGGAGTAGCCTTTTTAGTGCTAGCTGAACGTAAAGTAATGGCTTTTGTGCAACGTCGAAAGGGTCCTGA

TGTAGTGGGATCGTTTGGATTGTTACAACCTATAGCAGATGGTTTGAAATTGATTCTAAAAGAACCTATT

TCACCAAGTAGTGCTAATTTCTCCCTTTTTAGAATGGCTCCAGTGGCTACATTTATGTTAAGTCTGGTCG

CTCGGGCCGTTGTACCTTTTGATTATGGTATGGTATTGTCAGATCCGAACATAGGGCTACTTTATTTGTT

TGCCATATCTTCGCTAGGTGTTTATGGAATTATTATAGCAGGTCGGTCTAGTA-TTATTATATACGCTTA

GTGAAAAGAATGTTTTTTGATACACCTAGGACATGGATTCTATATGAACCAATGGATCGTAACAAGTCGT

TACTACTAGCAATGACTTCCTCTTTCATTACTTCATTCTTTCCATATCCCTCTCCTTTGTTCTCAGTTAC

TCATCAAATGGCACTCAGTTCATATCTTTAA-TGTTCGAATTTGCACCTATTTGTATCTATTTAGTGATT

AGTCTGCTAGTTTCTTTGATCTTACTCGGTGTTCCTTTTCTATTTTCTTCCAATAGTTCGACCTACCCAG

AAAAATTGTCGGCCTACGAATGTGGTTTCGATCCTTTCGGTGATGCCAGAAGTCGCTTTGATATAAGATT

TTATCTTGTTTCCATTTTATTTATTATCTTTGATCTGGAAGTCACCTTTTTCTTTCCTTGGGCAGTCTCT

CTCAACAAGATTGATCTGTTTGGATTTTGGTCCATGATGGCCTTTTTATTGATTTTGACGATTGGATTTC

TCTATGAATGGAAAAGGGGTGCTTTGGATTGGGAATAAAAAAGTGTTTCTTACGATTACGCCCAACAGCC

CACTTGAGCAATTTGCCATTCTCCCATTAATTCCTATGAATATAGGAAACTTGTATTTCTCATTCACAAA

TCCATCTTTGTTTATGCTACTCACTCTCAGTTTGGTCCTACTTCTGGTTCATTTTGTTACTAAAAACGGA

GGAGGAAACTCAGTACCAAATGCTTGGCAATCCTTGGTAGAGCTTATTTATGATTTCGTGCCGAACCCGG

TAAACGAACAAATAGGTGGTCTTTCCGGAAATGTTAAACAAAAGTTTTCCCCTCGCATCTCGGTCACTTT

TACTTTTTCGTTATTTCGTAATCTCCAGGGTATGATACCTTATAGCTTCACAGTTACAAGTCATTTTCTC

ATTACTTTGGGTCTCTCATTTTCTCTTTTTATTGGCATTACTATAGTGGGATTTCAAAGAAATGGGCTTC

ATTTTTTAAGCATCTCATTACCCGCAGGAGTCCCACTGCCGTTAGCACCTTTTTTAGTACTCCTTGAGCT

AATCCCTCATTGTTTTCGCGCATTAAGCTCAGGAATACGTTTATTTGCTAATATGATGGCCGGTCATAGT

TCAGTAAAGATTTTAAGTGGGTCCGCTTGGACTATGCTATGTATGAATGATCTTTTATATTTCATAGGAG

ATCTTGGTCCTTTATTTATAGTTCTTGCATTAACCGGTCCGGAATTAGGTGTTGCTATATCACAAGCTCA

TGTTTCTACGATCTTAATCTGTATTTACTTGAATGATGCTACAAATCTCCATCAA---------------

-----------------TATGTGGGCACCTGATATCTATGAGGGTTCACCCACCCCGGTTACAGCATTCC

TTTCTATTGCACCTAAAATCTCTATTTCTGCTAATATTTCACGTGTTTCTATTTATGGTTCCTATGGAGC

TACATTGCAACAAATCTTCTTTTTCTGCAGCATTGCTTCTATGATCTTAGGAGCACTGGCCGCCATGGCC

CAAACGAAAGTCAAAAGACTTCTAGCTTATAGTTCAATTGGACATGTAGGTTATATTCGTACAGGTTTCT

CATGTGGAACCATAGAAGGAATTCAATCACTACTAATTGGTATCTTTATTTATGCATCAATGACGATAGA

TGCATTCGCCATAGTTTCAGCATTACGGCAAACCCGTGTCAAATATATAGCGGATTTGGGCGCTCTAGCC

AAAACGAATCCTATTTCGGCTATTACCTTCTCCATTACTATGTTCTCATACGCAGGAATACCCCCGTTAG

CCGGCTTTTGTAGCAAATTCTATTTGTTCTTCGCCGCTTTGGGTTGTGGGGCTTACTTCCTAGCCCCAGT

GGGAGTAGTGACTAGCGTTATAGGTCGTTTGTTCGATAGCCTGACCGTAGTGATGTTAATTGTGGTTACA

TTCATAAGTAGCTTGGTCCATCTTTATTCCATTTCATATATGTCTGAGGATCCGCATAGCCCTCGATTTA

TGTGTTATTTATCCATTTTTACTTTTTTTATGCTAATGTTGGTGACTGGAGATAACTTTCTTCAATTATT

CCTGGGATGGGAGGGAGTAGGTCTTGCTTCATATTTGTTAATTCATTTCTGGTTTACACGACTTCAGGCA

GATAAAGCAGCTATCAAAGCTATGCTTGTCAATCGAGTAGGTGATTTTGGATTAGCCCTTGGTATTTTTG

GTTGTTTTACTCTCTTTCAAACAGTAGACTTTTCCACCATTTTTGCTTGTGCTAGTGTCCCCAGAAATTC

TTGGATTTTTTGCAATATGAGATTCAATGCCATAACTCTTATTTGTATTTTACTTTTTATTGGTGCTGTT

GGGAAATCTGCACAGATAGGATTGCATACTTGGTTACCTGATGCTATGGAGGGTCCCACTCCAGTATCCG

CTTTGATTCATGCAGCTACTATGGTAACAGCTGGCGTTTTCATGATAGCAAGGTGCTCCCCTTTATTTGA

ATACTCACCTACGGCTTTGGTTGTTATTACTTTTGCAGGAGCTATGACGTCATTCCTTGCGGCAACCACT

GGAATATTACAGAACGATCTAAAGAGGGTCATAGCTTATTCAACTTGCAGTCAATTAGGCTATATGATCT

TTGCTTGCGGCATCTCTAACTATTCGGTTAGCGTCTTTCACTTAATGAATCACGCGTTTTTCAAAGCATT

ACTATTCCTGAGTGCAGGTTCGGTAATTCATGCCATGTCGGATGAGCAAGATATGCGGAAGATGGGGGGG

CTTGCCTCCTCGTTCCCTTTTACCTATGCCATGATGCTCATGGGCAGCTTATCTCTAATTGGATTTCCTT

TTCTAACTGGATTTTATTCCAAAGATGTGATCTTAGAGCTCGCTTACACTAAGTATACCATCAGTGGGAA

CTTTGCTTTCTGGTTGGGAAGTGTCTCTGTCCTTTTCACTTCTTATTACTCTTTTCGTTTACTTTTTCTA

ACATTTCTAGTACCAACTAATTCATTCGGGCGAGACAGATTACGATGTCATGATGCGCCCATTCCTATGG

CCATTCCTTTAATACTTCTGGCTCTCGGAAGTCTCTTTGTAGGATACTTGGCCAA-CTAACACAAAGAAG

ATACAGTTCACTCAACGATTGCCTTTGGGTTCCGAACTCCATATGGGGAAGGAGCGTTGTTGTTTGCGAG

GTCTCGATCATTTACATGGACCCACTTTTCATTCTATTTGTGGGAATTTGATGATCTATAAACCGTCCTT

AACGAACGATCGGCTGATGTT------TGAGCATGATGAATCATTTCGTGCCGACCTGTTGCCAATAAAC

TTTCCGGCCTCATATGAGAATGGGAAACTGGAGCATTTTCTGCATCGGTGGATGAAGAATCGCGAGCATA

ATAATTTCTGGTTGACCATGTTCCCAGAAAAAAGATACTTTCGAGAAACGACGAGTACGACGGAAGTGGC

TATACATACAAATCCAGTTACGGATCTATATGCTTCGATTGGAACTGGAAGTTCCAGAACAGGCGGCTGG

TATACCACCATAATGAAACTGCCTTTTCTTTTTTTTATTTGGATAGGATTTATGTTGGCTTCGTTGGGAG

GCTCGCGTAGTTTGTTACGTCAGCTCCAAAAGGATAAGTTGCGTTGGAATCGAGAAAGTTCCGTGG----

AGTTAATAATTGCATA--CCATTTTTGGGCCAATTCCCTCTTCGTACTACCAAAAAATGAGATTCTTGCC

GAATCCGAGTTTGCTGCTCCAACCATTATAAAACTAATACCTATTCTGTTTAGTACTTTAGGTGCTTTTG

TTGCGTATAATGTAAATCTCGTAGCGGATCAATTCC---------------AACGAGCCTTTCAAACTAG

TACTTTTTGTAATCGACTCTATAGCTTCTTCAATAAACGCTGGTTCTTCGATCAAGTTTTGAATGACTTT

ATAGTCAGATCGTTCCTGCGTTTCGGATATGAAGTCTCATTCGAAGCTTTAGACAAAGGTGCTATTGAGA

TATTGGGGCCTTATGGTATCTCGTACACATTCCGACGATTGGCCGAGCGAATAAGTCAACTTCAAAGTGG

ATTTGTT-TGTTCCATGATCTATGGGTCTACTGGAGCTACCCACTTCGATCAATTAGCCAAGATTTTGAC

CGGATACGAAATCACAGGTGCTCGATCTAGTGGTATTTTTATGGGGATTCTATCTATCGCTGTAGGATCC

CTATTCAAGATCACTGCAGTTCCTTTTCTGGTCTATGCACATTGCTCTTTCTAGGAGGTTGGCTGCCTAT

CCTAGATCTTCCTATTTTCAAGAAGATCCCGGGCTCGATCTGGTTTAGTATCAAGGTGATTCTTTTTCTG

TTTCTATATATATGGGTCCGTGCAGCATTTCCACGATATCGTTATGATCAATTAATGGGACTTGGCCGGA

AAGTGTTCTTGCCTCTATCATTAGCTCGGGTAGTCGCCGTTTCTGGTGTTTTAGTCACCTTTCAATGGCT

CCCTTA--TGCCTCAACTGGATAAATTCACTTATTTCACACAATTCTTCTGGTCATGCCTTTTCCTCTTG

ACTTTCTATATTCCCATATGCAATGATGGAGATGGAGTACTTGGGATCAGCAGAATTCTCAAACTACGGA

ACCAACTGGTTTCACACCGGGAGAACAACATGCGGA------GCAACGACCCCAAGAGTTTGGAGGATAT

CTTGAGAAAAGGTTTTAGCACCGGTGTATCCTATATGTACTCCAGTTTATTCGAAGTATCTCAATGGTGT

AAGGCCGTCGACTTATTGGGAAAAAGGAGGAAAATCACTTTGATCTCTTGTTTCGGAGAAATAAGTGGCT

CACGAGGAATGGAAAGAAACATATTCTATTTGATCTCGAAGTCCTCATATAGCACTTC------------

---TTCTAATCCTGAATGGGGGATCACTTGTAGGAATGACATAATGCTAATCCATGTTCCACACGGCCAA

GGAAGCATCGTTTTTT-------

>Alternanthera philoxeroides MN166292.1

ATGATACTTTCTGTTTTGTCGAGCCTGGCTTTGGTCTCTGGTTTGATGGTTGTACGTGCTAAAAATCCGG

TACATTCCGTTTTGTTTCCCATCCCAGTCTTTCGCAACACTTCAGGGTTACTTCTTTTGTTAGGTCTCGA

TTTTTCCGCTATGATCTTCCCAGTAGTTTATATAGGAGCTATAGCCGTTTCATTCCTATTCGTTGTTATG

ATGTTCCATATTCAAATAGCGGAGATTCACGAAGAAGTATTGCGCTATTTACCAGTGAGTGGTATTATTG

GACTGATCTTTTGGTGGGAAATGTTCTTCATTTTAGATAATGAAACCATTCCATTACTACCAACCCAAAG

AAATACGATCTCTCTGAGATATACGGTTTATGCCGGAAAGGTACGAAGTTGGACTAATTTGGAAACATTG

GGCAATTTACTTTATACTTACTATTTTGTCTGGTTTTTGGTTTCTAGTCTTATTTTATTAGTAGCCATGA

TTGGGGCTATAGTACTGACTATGCATAGGACTACTAA------GGTGAAAAGACAGGATGTATTCCGACG

AAATGCTATTGATTCTAGAAGGACTATAATGAAGAGGACGACAGAC---TGTCAATATATGAATTGTTTC

ATTATTCGTTATTTCCGGGTCTTTTCATTGCATTCACTTATAACAAGAAACAACCACCAGCGTTTGGTGC

AGCACCTGCATTTTGGTGTATTCTTCTTTCTTTCCTTGGTCTTTTGTTCTGTCATATTCCTAATAACTTA

TCCAATTACAACGTATTAACCGCTAATGCACCTTTCTTTTATCAAATCTCAGGGACATGGTCTAATCATG

AAGGTAGTATTTTATTATGGTGTCGGATCCTAAGTTTTTATGGATTCCTTCTTTGTTACCGGGGTCGATC

CCAAAGCCATAATGTCTCAAAACGAGGAGGCCATAGAGAAAGTCTTCTTTTTTCCTTTGTCTTAAACTTC

GTGAAGAACTCCATTCTATCTCTTCCTCGTTACGAACAAAAAAGTAGAGTTCTTCACGAACCCCAGTTGT

ACGCTCTCTTTGTTCTACGAA---CTCTTGTTGATTCTGAACTTTGTTCGCGAAGGAACCGGACTTTTGA

CGGGCCAGCTCTTTTTTACGCGCCGCTTTACCCTGAAAGGAAAATGAGCTTTGATCTTCTGGGCGCTAGG

CGCTCTCGTGGTTCGCGAGAAGGAAAAAGGACTCATCCTTTGTTGCATCTGGCACGAGATGATAAAGAGA

GAGCTTCGTCTATCGATGAACAGCGGATTGACGGAGCTCTTGGCATTGCTTTCTTTTTCTTTACTTTCTT

ATCAGCGAGTTCCGATCCTTTTGTTCGAAATTTCTTCGTTCGTACCGAACCGCTTGCAGAATCAAATCCT

GTTCCACAAGATCCTATATCAGCTATACATCCTCCTTGCATTTATGCCGGAGACGTCGCCAGTGCTATAG

GCTTTGGCTTATGTAGATCAAAAATGATGAATGGGATTGTGGCACTCCACTCGCCGCCAATGCGGAAGGA

TGTCGCCGAAAAGAATGGAACGCTGCTTTGCTCTGCTGGATGCGTCGGATCCCGTATAACAAGCGAGCTC

TTTACCCTTAAATTCAAACATGTGGGCGCCAAATGCTATCCTGCTCTATTGTTGCGTAGCAAAAGAAGCC

TGCTC---ATGCTGCTTCGGCGGCGCTTTTTCGCCTTCTCTTCGCTCTGGACAAGAGCGCTAGTGGACAC

GGGGAGGGAGCG------GGCGAAGCGTTTCT------TTCGTAATGGAAAGAAAAAGATCACTACTTTG

CCTCTTTGTTGGACCGCCGGCGCGAACATAGTGGGCTCTGACCAGGACCAGGAACCAATTCGAATTTGGA

TCTTGACATGTCGGTGCTTTTTAACCGTAGGCATCTTGCCAGGAAGTTGGTGGGCTCATCATGAATTAGG

TCGGGGTGGCTGGTGGTTTCGGGATCCCGTAGAAAATGCTTCTTTTATGCCTCGGGTATTAGCCACAGCT

CGTATTCATTCAGTAATTTTACCCCTTCTTCATTCTTGGACTTTGCTTCTTAATATTGTTACTTTTCTAT

GCTGTGTCTTAGGAACCTTTTCAATACGGTCCGGATTGCTAGCTCCCGTTCATAGTTTTGCTACAGATGA

TACACGAGGAATCTTTTTATGGCGGTTCTTCCTTCTAATGACCGGCATATCTATGATTCTTTTCTCTCAG

ATGAAGCAGCAGGCATCGGTCCGTAGAACCTATAAAAAAGAGATGGTTGTAGCGCGA-------------

--------------------------GGCTATTCTTCACAATGACTCCTTGTGATGCAGCGGAACCATGG

CAATTAGGATTTCAAGACGCAGCAACACCTATGATGCAAGGAATAATCGACTTACATCATGATATCTTTT

TCTTCCTTATTTTTATTTTGGTTTTCGTATCATGGATCTTGGTTCGCGCTTTATGGCATTTCCACTATAA

AAAAAATCCAATCCCGCAAAGGATTGTTCATGGAACTACTATCGAGATTATTCGGACCATATTTCCCAGT

ATCATCCTGATGTTCATTGCTATACCATCATTTGCTCTGTTATACTCAATGGACGAGGTAGTAGTAGATC

CAGCCATTACTATCAAAGCTATTGGACATCAATGGTATCGGAGT--------------------------

----------------------------------------------------------------------

----------------------------------------------------------------------

----------------------------------------------------------------------

----------------------------------------------------------------------

----------------------------------------------------------------------

-----------------------ATGAGACGACTCTTTTTTGAATTATATCATAAACAGATCTTCTTCTC

TACACCAATCACGAGTTTTTCTCCATTCCTCTCGTATATTGTCGTAACGCCCTTAATGCTAGGTTTTGAA

AAAGACTTTTCATGTCATTTCCATTTAGGTCCGATTCGGATCCCTCTGTTGTTTCCTTTTCTTCCCGCGC

CTTTTCTTCGAAATGAGAAAGAAGATGGTACACTCGAATTGTATTATTTAAGCGCTTATTGCTTGCCAAA

GATCCTACTTCTACAATTGGTAGGTCACTGGATTATTCAAATAAGTCGTGTTTTCTGTAGTTTTCCCATA

TTACAACTTCTGTACCAATTCGGTCAATCCGGAATGGATCGGTTAAACATTCTATTAGGGAGCCTGGTCT

TGACTCTTCTGTGTGGTATTCATTCTTGTTTGGCTCTTGGAATCACATCCAGCAGTGGTTGGAACAGCTC

GCAAAATTTAACCACTTCACCTACTTCATTGCCCTCAACCGTTTCTCGTACCTCTATTGAAACAGAATGG

TTTCATGTTCTTTCATCGATTGGTTATTTTTCTTCGTTCGTATCTCTTTTTCCAATTTCGGTCTCGATTA

GTTCACAAGATTG--TGTCCGTTTTGTTATTACAACCTTA-----TTTTTTGATGTCAAAGACCAGAAGC

TACGCGCAAATTCTCATTGGATCTTGGTTGTTCTTAACAGCGATGGCTATTCATTTAAGTCTTTGGGTAG

CACCACTAGATTTTCAACAAGGTGGAAATTCTCGTATTCTCTATGTACATGTTCCTGTGGCTCGGATGAG

TATTCTTGTTTATATCGTTACGGCTATAAACACTTTCTTGTTCCTATTAACAAAACATCCTCTTTTTCTT

CGCTCTTCCGGAACCGGTACAGAAATGGGTGCTTTTTCTACGTTGTTTACCTTAGTTACTGGGGGGTTTC

GGGGAAGACCCATGTGGGGCACCTTTTGGGTGTGGGATGCTCGTTTAACTTCTGTATTCATCTCGTTCCT

TATTTACTTGGGTGCGCTGTGTTTTCAAAAGCTTCCTGTAGAACCGGCTCCTATTTCAATCCGTGCTGGA

CCGATCGATATACCAATAATAAAGTTTCCAGTCAACTGGTGGAATACGTCGCATCAACCTGGGAGCATTA

GCCGATCTGGTACATCAATACATGTTTCTATGCTCATTCCAATCTTGTCTAACTTTGCTAACTTCCTCTT

CTCAACCCGTATCTTCTTTGTTCTGGAAACACGTCTTCCTATTCTATCTTTTCTCGAATCTCCTTTAACG

GAAGAAATAGAAGCTCGAGAAGGAA-----GCAGGCTAGAAAGATGCTATTTGCTGCTATTCTATCTATT

TGTGCATCAAGTTCGAAGAAGATCTCAATCTATAATGAAGAAATGATAGTAGCTCGTTGTTTTATAGGCT

TTATCATATTCAGTCGGAAGAGTTTAGGTAATACTTTCAAAGCGACTCTCGACGAGAGAATCCAGGCTAT

TCAGGAAGAATTGCAGCAATTCCCCAATCCTAACGAAGTAGTTCCTCCGGAATCCAATGAACAACAACGA

TTACTTAGGGTCAGCTTGCGAATTTGTGGAACCGTAGTAGAATCATTACCAATGGCACGCTGTGCGCCTA

AGTGCGAAAAGACAGTGCAAGCTTTGTTATGTCGAAACCTAAATGTTAAGTCAGCAACACTTCCAAATGC

CACTTCTTCCCGTCGCACCCGTCTTCAGGACGATCTAGTCACAGGGTTTCACTTCTCAGTGAGTGAAAGA

TTTGTCCCCGGGTCTACGTTGAAAGCTTCTATAGTAGAACTCATTCGAGAAGGCTTGGCGGTCTTAAGAA

TGGTTCGGGTAGGGGGTTCTCTT--TGAAAGAGGCGATCAGAATGGTACTCGAATCCATTTACGATCCCG

AGTTTCCAGACACATCGCACTTCCGCTCGGGTCGAGGCTGCCATTCGGCCCTAAGACGGATCAAAGAAGA
[truncated: 55,419 more chars]
